# A combinatorial approach of comprehensive QTL-based comparative genome mapping and transcript profiling identified a seed weight-regulating candidate gene in chickpea

**DOI:** 10.1038/srep09264

**Published:** 2015-03-19

**Authors:** Deepak Bajaj, Hari D. Upadhyaya, Yusuf Khan, Shouvik Das, Saurabh Badoni, Tanima Shree, Vinod Kumar, Shailesh Tripathi, C. L. L. Gowda, Sube Singh, Shivali Sharma, Akhilesh K. Tyagi, Debasis Chattopdhyay, Swarup K. Parida

**Affiliations:** 1 National Institute of Plant Genome Research (NIPGR), Aruna Asaf Ali Marg, New Delhi 110067, India; 2 International Crops Research Institute for the Semi-Arid Tropics (ICRISAT), Patancheru 502324, Telangana, India; 3 National Research Centre on Plant Biotechnology (NRCPB), New Delhi 110012, India; 4 Division of Genetics, Indian Agricultural Research Institute (IARI), New Delhi 110012, India

## Abstract

High experimental validation/genotyping success rate (94–96%) and intra-specific polymorphic potential (82–96%) of 1536 SNP and 472 SSR markers showing *in silico* polymorphism between *desi* ICC 4958 and *kabuli* ICC 12968 chickpea was obtained in a 190 mapping population (ICC 4958 × ICC 12968) and 92 diverse *desi* and *kabuli* genotypes. A high-density 2001 marker-based intra-specific genetic linkage map comprising of eight LGs constructed is comparatively much saturated (mean map-density: 0.94 cM) in contrast to existing intra-specific genetic maps in chickpea. Fifteen robust QTLs (PVE: 8.8–25.8% with LOD: 7.0–13.8) associated with pod and seed number/plant (PN and SN) and 100 seed weight (SW) were identified and mapped on 10 major genomic regions of eight LGs. One of 126.8 kb major genomic region harbouring a strong SW-associated robust QTL (*Caq'SW1.1*: 169.1–171.3 cM) has been delineated by integrating high-resolution QTL mapping with comprehensive marker-based comparative genome mapping and differential expression profiling. This identified one potential regulatory SNP (G/A) in the *cis*-acting element of candidate *ERF* (ethylene responsive factor) TF (transcription factor) gene governing seed weight in chickpea. The functionally relevant molecular tags identified have potential to be utilized for marker-assisted genetic improvement of chickpea.

Chickpea (*Cicer arietinum* L.), represented majorly by *desi* and *kabuli* cultivar types, is one of the most cultivated food legume crops in the world. High yield potential but low crop productivity necessitates genetic improvement of yield component and stress tolerant traits of chickpea cultivars. To expedite the marker-assisted breeding for genetic enhancement in chickpea, identification and mapping of informative markers tightly linked to the genes/QTLs (quantitative trait loci) regulating important agronomic traits is essential. In recent years, such marker-assisted trait improvement in a large chickpea genome with narrow genetic base is predominantly attributed to construction of high-resolution SNP (single nucleotide polymorphism) and SSR (simple sequence repeats) marker-based intra- and inter-specific genetic linkage maps, and subsequently fine mapping and map-based cloning of trait-governing genes/QTLs.

Until recently, about two thousand SSR and SNP markers have been validated and genotyped in diverse mapping populations using high-throughput genotyping assays to construct the high-density inter-specific genetic linkage maps in chickpea^1^,^2^,^3^,^4^,^5^,^6^,^7^,^8^,^9^,^10^. By use of numerous such informative sequence-based codominant, multi-/bi-allelic and abundant SSR and SNP markers in combinations, the resolution of constructed inter-specific genetic linkage maps in terms of mean map-density has now increased upto 0.59–1.7 cM in chickpea^8^,^9^. Several efforts have also been made to construct SSR marker-based low resolution genetic linkage maps (map density ranged from 2.5-7 cM) utilizing the diverse *desi* and *kabuli* intra-specific mapping populations^11^,^12^,^13^,^14^,^15^,^16^,^17^,^4^,^18^,^19^,^20^,^21^,^22^. However, only single report on construction of high-density intra-specific genetic linkage maps (with map density varied from 1.74 to 2.16 cM) by high-throughput genotyping of about 1000 SSR and SNP markers in RIL (recombinant inbred lines) mapping populations using automated fragment analyzer and Illumina GoldenGate assay is available in chickpea^23^. Therefore, the combined use of SSR and SNP markers in large-scale validation and high-throughput genotyping of diverse mapping populations using suitable modern advanced genotyping assays can significantly enhance the resolution of intra-specific genetic linkage maps in chickpea.

Using the intra- and inter-specific genetic map information, many QTLs associated with yield component and abiotic/biotic stress tolerance traits have been identified and mapped in chickpea. It includes identification and mapping of QTLs associated with *Fusarium* wilt, *Ascochyta* blight, *Botrytis* gray mold and rust resistance, salinity and drought tolerance, root traits, flowering time, plant growth habit, seed size/100-seed weight, double podding, seed/pod number per plant and harvest index in chickpea^11^,^15^,^16^,^19^,^21^,^22^,^23^,^24^,^25^,^26^,^27^,^28^,^29^,^30^,^31^,^32^,^33^,^34^,^35^,^36^,^37^,^38^,^39^,^40^,^41^,^42^,^43^,^44^,^45^,^46^. In spite of such huge efforts on QTLs identification, most of the markers/genes harboring QTL regions have not been fine mapped and validated across diverse mapping populations and/or environments to be harnessed for efficient marker-assisted selection for chickpea genetic improvement. The available draft assemblies of genome and transcript sequences of diverse *desi* and *kabuli* chickpea have enabled to select numerous chromosome-wise well distributed and informative *in silico* polymorphic SSR and SNP markers for genomics-assisted breeding applications in chickpea^7^,^47^,^48^,^49^,^50^. In this perspective, large-scale validation and high-throughput genotyping of genome-wide polymorphic SSR and SNP markers and their use in construction of diverse mapping population-derived high-resolution intra-specific genetic linkage maps are now feasible in chickpea. It would also accelerate the identification, fine mapping and map-based isolation of genes/QTLs associated with traits of agricultural importance, and thereby, genetic enhancement of chickpea through marker-assisted selection.

Keeping all above in view, the present study was undertaken to validate and genotype genome-wide physically mapped 1632 SNP and 500 SSR markers showing *in silico* polymorphism between ICC 4958 (*desi*) and ICC 12968 (*kabuli*) in a 190 F_4_ mapping population (ICC 4958 × ICC 12968) using Illumina GoldenGate assay, gel-based assay and fluorescent dye-labeled automated fragment analyzer. The marker genotyping and robust field phenotyping information of mapping individuals were utilized to develop a high-resolution intra-specific genetic linkage map for identification of major QTLs associated with pod and seed number/plant and 100-seed weight in chickpea. The relevant high-resolution QTL mapping information was integrated with comprehensive marker-based comparative genome mapping and differential expression profiling to delineate a candidate gene at one of the robust seed weight-governing major QTL region in chickpea.

## Results and Discussion

The large-scale validation and high-throughput genotyping of genome-wide informative sequence-based robust SNP and SSR markers in advanced generation mapping populations is useful in construction of high-resolution genetic linkage maps and identification/mapping of genes/QTLs associated with important agronomic traits, which could accelerate genetic enhancement in chickpea. To expedite such process in a large chickpea genome with narrow genetic base, the use of whole genome SNP and SSR markers showing *in silico* polymorphism (based on repeat-unit variations) in the genomic and transcript sequences among diverse *desi* and *kabuli* genotypes could be an attractive strategy. In our study, we selected genome-wide (physically mapped on eight chromosomes) *in silico* polymorphic genic and genomic 1632 SNP and 500 SSR markers between *desi* ICC 4958 and *kabuli* ICC 12968 chickpea for their large-scale validation and high-throughput genotyping in 190 F_4_ mapping individuals using the gel-based assay, fluorescent dye-labelled automated fragment analyser and Illumina GoldenGate assay to construct a high-density intra-specific genetic linkage map in chickpea.

### Large-scale validation and high-throughput genotyping of SNP and SSR markers

**Figure 1:**
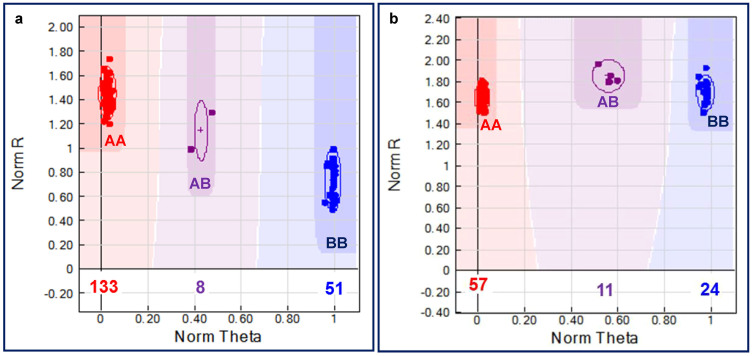
Example of one regulatory SNP (G/A) validated in a *ERF* TF gene by Illumina GoldenGate genotyping assay showing homozygous and heterozygous cluster separation for 190 mapping individuals along with two parental genotypes (a) and 92 *desi* and *kabuli* genotypes (b) based on plotting of normalised R [sum of intensities of the two channels (Cy3 and Cy5)] on the y-axis vs. normalised theta [(2/π)Tan^-1^(Cy5/Cy3)] on the x-axis. A normalised theta value nearest 0 is homozygous for allele A (red), a theta value nearest 0.5 is heterozygote AB (violet) and a theta value nearest 1 is homozygous for allele B (blue).

A selected set of 1632 SNPs with designability scores of ≥0.8 were included to design chickpea “*Ca*-II-OPA” for their genotyping in 190 F_4_ mapping individuals (ICC 4958 × ICC 12968) and parental genotypes though Illumina GoldenGate assay. Reproducibility of genotyping assay was estimated as 100% using two parental genotypes as biological replicates. Of the 1632 SNP loci, 1587 (97.2%) could be genotyped successfully on all 192 individuals showing distinct cluster separation at ≥0.3 GenCall and GenTrain scores. After removal of missing SNP genotyping data, including monomorphic and heterozygous SNPs from parental genotypes, 1536 SNPs (Supplementary Table S1) were found relevant with overall genotyping success rate of 94.1%. Genotype polar coordinate plots [normalized sum of intensities of two channels (Cy3 and Cy5) as y-axis vs. normalized theta {(2/π)Tan^−1^(Cy5/Cy3)} as x-axis] of these 1536 SNPs were used to classify 192 individuals into one of three clusters: (I) homozygous AA (ICC 4958), (II) homozygous BB (ICC 12968) and (III) heterozygous AB (both ICC 4958 and ICC 12968) allele types (Fig. 1a). High reproducibility (100%) and overall experimental validation and genotyping success rate (94.1%) of SNPs obtained in GoldenGate assay is comparable/higher than the estimates (80–92%) determined in diverse crop plants, including rice, maize, barley and chickpea^51^,^52^,^53^,^54^,^55^,^8^,^56^. It suggests the reliability, robustness and utility of Illumina GoldenGate assay in large-scale validation and high-throughput genotyping of SNPs in chickpea.

**Figure 2:**
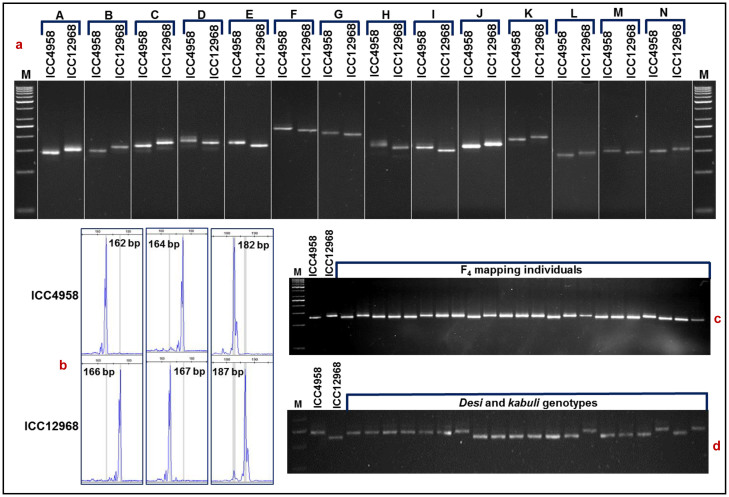
Validation of a representative set of 14 SSR markers (physically mapped on eight chickpea chromosomes) showing *in silico* fragment length polymorphism between parental genotypes (ICC 4958 and ICC 12968) of a F_4_ mapping population (ICC 4958 × ICC 12968) using the gel-based assay (a) and fluorescent-dye labeled automated fragment analyzer (b). (c) Segregation pattern of one selected SSR marker in a representative set of mapping individuals. (d) Amplification and polymorphism profiles of one SSR marker in a selected set of *desi* and *kabuli* genotypes. The fragment sizes (bp) of the amplified polymorphic alleles are indicated. The identities of SSR markers with their detailed information are provided in the Supplementary Table S1. M: 50 bp DNA ladder size standard.

A total of 500 SSR markers showing ≥ 2-bp *in silico* fragment length polymorphism between ICC 4958 and ICC 12968 based on variation in their repeats were selected for experimental validation using gel-based assay. Of these, 479 markers (Supplementary Table S1) produced single reproducible PCR amplicons in 3.5% metaphor agarose gel with an average amplification success rate of 95.8%. Four hundred seventy-two (98.5%) of 479 amplified SSR markers showing *in silico* polymorphism between ICC 4958 and ICC 12968 based on variation in their repeat-length were validated experimentally using both gel-based assay and fluorescent dye-labeled automated fragment analyzer (Fig. 2a, b). Moreover, high experimental validation (95.8%) and genotyping (94.4%) success rate of *in silico* polymorphic SSR markers in mapping individuals as well as parental genotypes (ICC 4958 and ICC 12968) infer broader applicability of these SSR markers in chickpea genome analysis and molecular breeding.

### Polymorphic potential of SNP and SSR markers

A selected 96 genome-wide well-distributed (physically mapped on eight chickpea chromosomes) SNP markers were genotyped in 92 *desi* and *kabuli* chickpea genotypes using GoldenGate assay. Ninty-two (95.8%, mean PIC: 0.43) of 96 SNP markers showed polymorphism among these genotypes (Fig. 1b). Eighty (87%. mean PIC: 0.39) of 92 SNP markers showed polymorphism between *desi* and *kabuli*, while 63 (68.5%, mean PIC: 0.32) and 43 (46.7%, 0.26) markers were polymorphic within 52 *desi* and 40 *kabuli* chickpea, respectively. A set of 96 SSR markers physically mapped on eight chickpea chromosomes were selected to evaluate their polymorphic potential among 92 *desi* and *kabuli* genotypes using gel-based assay and automated fragment analyzer. Seventy-nine (82.2%) markers of these showed polymorphism (with average PIC of 0.69) among *desi* and *kabuli* genotypes (Fig. 2c). Sixty-six (83.5%, mean PIC: 0.65) of 79 markers were polymorphic between *desi* and *kabuli*. Fifty-two (65.8%) of 79 markers showed polymorphism among 52 *desi* genotypes (varied from 1 to 4 alleles with mean PIC of 0.60), while 34 (43%) markers detected polymorphism among 40 *kabuli* genotypes (1 to 3 with 0.51). The 92 SNP and 79 SSR markers overall produced a total of 528 alleles in 92 chickpea genotypes. The number of alleles detected by these markers varied from 2 to 4 with an average of 3.1 alleles per marker.

The intra-specific polymorphic potential detected by SNP (95.8%) and SSR (82.3%) markers among 92 *desi* and *kabuli* chickpea genotypes is much higher compared to that estimated using *in silico* polymorphic SSR markers (50–60%,^50^,^57^). Remarkably, such intra-specific marker polymorphic potential was comparatively much higher than that estimated with random genome-wide SSR markers among *desi* and *kabuli* chickpea genotypes (~35%)^5^,^6^,^57^,^58^,^59^. Therefore, about 2000 highly informative *in silico* polymorphic SNP and SSR markers developed in our study at a genome-wide scale have utility in various high-throughput genotyping applications in chickpea. Furthermore, these polymorphic markers have practical significance in detecting a higher intra-specific polymorphic potential among *desi* and *kabuli* chickpea genotypes than any other random and sequence-based markers and thus, will serve as a valuable resource for expediting the genomics-assisted breeding applications in chickpea.

### Construction of a high-resolution intra-specific chickpea genetic linkage map

**Figure 3:**
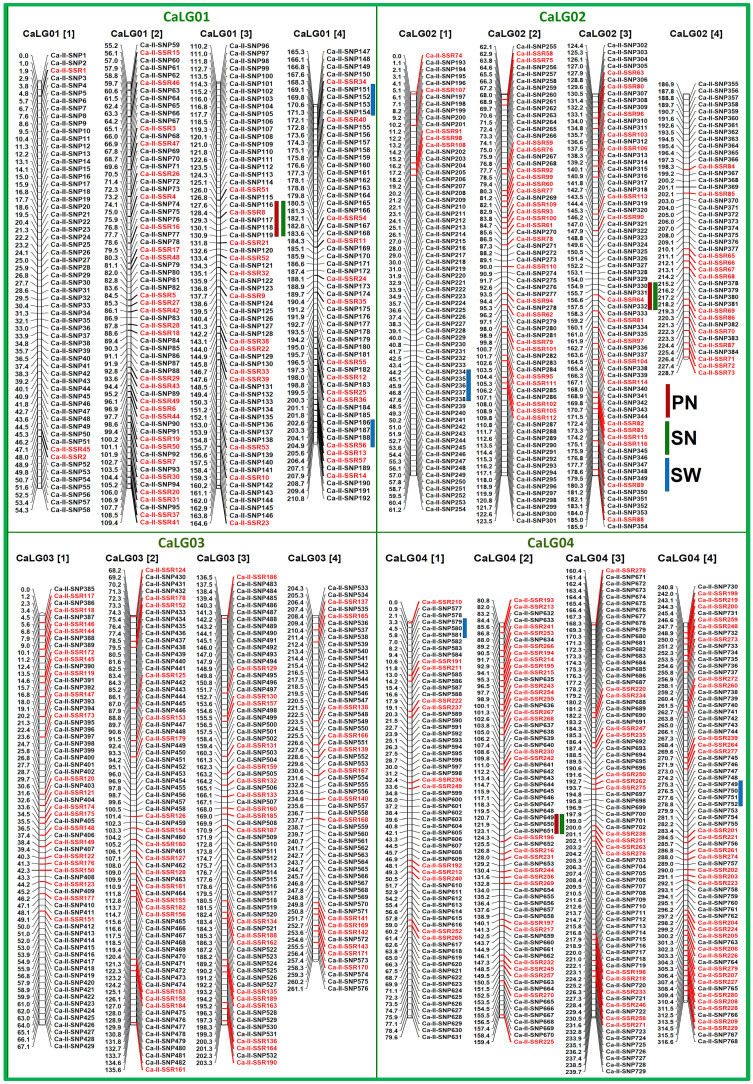
Eight major genomic regions underlying 11 robust QTLs (PVE: 8.5–25.8%, LOD: 6.5–13.8) associated with three agronomic quantitative traits (PN, SN and SW) identified and mapped on four LGs (CaLG01-CaLG04) using a 190 F_4_ mapping population (ICC 4958 × ICC 12968) of chickpea. The genetic distance (cM) and identity of the marker loci integrated on the chromosomes are indicated on the left and right side of the LGs, respectively. Red, green and blue boxes indicate the QTLs regulating PN, SN and SW mapped on eight LGs, respectively. For clear visibility, the individual LG has been divided into four parts; [1], [2], [3] and [4] based on lower to higher genetic positions of mapped markers.

**Figure 4:**
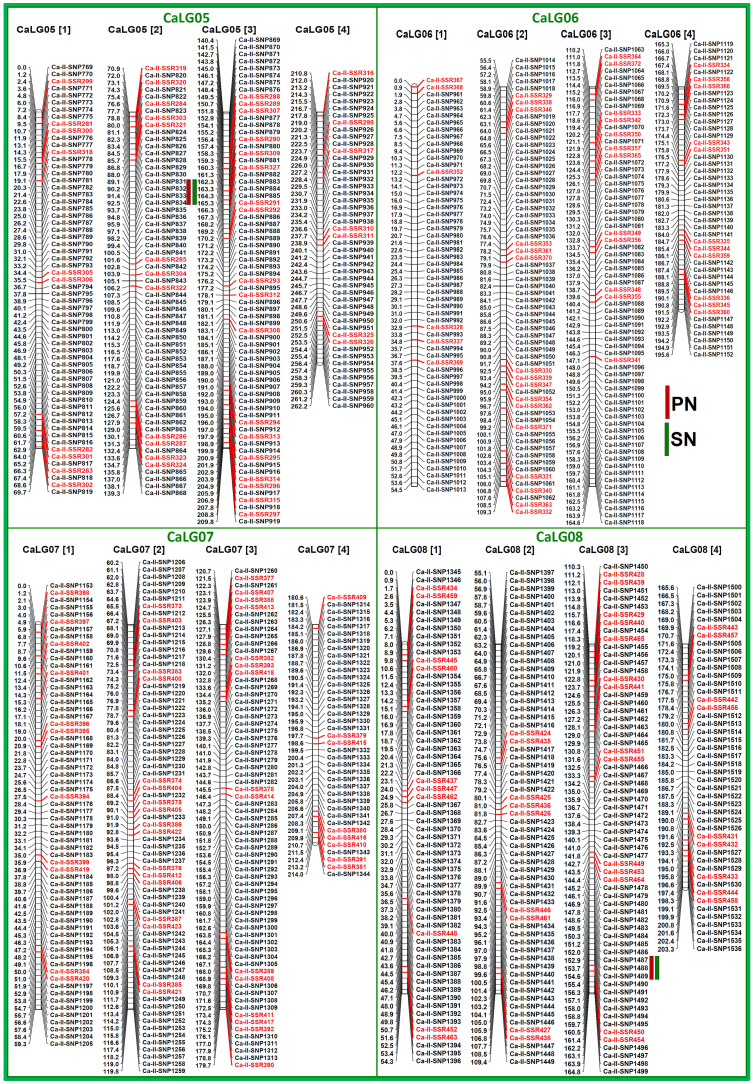
Two major genomic regions underlying four robust QTLs (PVE: 8.8–14.7%, LOD: 7.3–9.6) associated with two agronomic quantitative traits (PN and SN) identified and mapped on four LGs (CaLG05-CaLG08) using a 190 F_4_ mapping population (ICC 4958 × ICC 12968) of chickpea. The genetic distance (cM) and identity of the marker loci integrated on the chromosomes are indicated on the left and right side of the LGs, respectively. Red and green boxes indicate the QTLs regulating PN and SN mapped on eight LGs, respectively. For clear visibility, the individual LG has been divided into four parts; [1], [2], [3] and [4] based on lower to higher genetic positions of mapped markers.

To construct a saturated intra-specific genetic linkage map, 2008, including 1536 SNP (Fig. 1a) and 472 SSR (Fig. 2d) markers showing parental polymorphism between ICC 4958 and ICC 12968 were genotyped among 190 individuals of a mapping population (ICC 4958 × ICC 12968). The linkage analysis using 2008 marker genotyping data mapped 2001 (1536 SNP and 465 SSR markers) marker loci onto eight LGs of an intra-specific genetic map of chickpea (Table 1, Fig. 3, 4). This integrated high-density intra-specific genetic map comprising of eight LGs constructed by us supports the previous similar documentation^11^,^12^,^13^,^14^,^15^,^16^,^17^,^4^,^18^,^19^,^20^,^21^,^22^,^23^. The genetic map comprising eight LGs covered a total map length of 1888.86 cM with an average inter-marker distance of 0.94 cM (Table 1). Longest map length spanning 316.55 cM was observed in LG4, while LG6 showed shortest map length of 195.57 cM. Maximum (282 markers) numbers of markers were mapped on LG4, followed by LG3 (266) and minimum on LG8 (234). The LG6 had the most saturated genetic map (varying from 0.68 to 0.94 cM with an average inter-marker distance 0.82 cM), while LG4 contained the least saturated map (0.91 to 1.21 cM with an average of 1.12 cM) (Table 1).

The average inter-marker distance (0.94 cM) obtained in the presently constructed intra-specific genetic linkage map was much lower and thus highly saturated in contrast to that reported (2.5–7 cM) using diverse *desi* and *kabuli* intra-specific mapping populations^11^,^12^,^13^,^14^,^15^,^16^,^17^,^4^,^18^,^19^,^20^,^21^,^22^. This intra-specific genetic linkage map has remarkably higher map density compared to one of the integrated SSR and SNP marker-based intra-specific genetic maps (1.74–2.16 cM) of chickpea^23^. Therefore, we constructed a more advanced and highly saturated intra-specific genetic linkage map in contrast to all other intra-specific genetic maps reported so far in chickpea. Henceforth, this integrated high-density intra-specific genetic linkage map would be useful for mapping the whole genome and rapid targeted mapping of genes/QTLs governing important agronomic traits in chickpea as well as comparative mapping across legumes.

### Identification and mapping of QTLs associated with agronomic traits in chickpea

We observed a significant difference of three quantitative agronomic traits, PN [37.1–119.0 with 76% broad-sense heritability (H^2^)], SN (43.9–146.4 with 72% H^2^) and SW (20.4–44.5 g with 89% H^2^) in 190 mapping individuals (ICC 4958 × ICC 12968) and two parental genotypes across two years based on ANOVA (Supplementary Table S2). ANOVA results indicated highly significant differences (P < 0.0001) among mapping individuals (RILs) for all three traits despite significant environmental (years) effects on these traits in both seasons (Supplementary Table S3). A significant interaction between genotypes (G) and environment (E) for PN, SN and SW traits was also observed. However, the G × E (58% lower than total mean squares) and E (26.3% lower) variances were found to be smaller for SW compared with PN and SN at significance level of P < 0.001 (Supplementary Table S3). The normal frequency distribution of three agronomic traits in mapping individuals and parental accessions was observed across two years (Supplementary Fig. S1). Remarkably, bi-directional transgressive segregation of traits beyond that of parental genotypes in mapping population was evident. A highly significant positive correlation between PN and SN (r = 0.96, P < 0.0001) and negative correlation of PN and SN with SW (−0.26, P < 0.001) based on Pearson's correlation coefficient estimation was observed (Supplementary Fig. S2). A significant phenotypic variation and normal frequency distribution of three quantitative agronomic traits (PN, SN and SW) among 190 mapping individuals along with parental genotypes indicates the involvement of multiple genes for regulation of these traits, and thereby, suggests the utility of developed mapping population (ICC 4958 × ICC 12968) in QTL mapping.

The QTL mapping using the genotyping information of 2001 SNP and SSR markers mapped on an intra-specific genetic linkage map (Fig. 3, 4) and field phenotyping data of 190 mapping population identified and mapped 18 major genomic regions underlying 28 significant (LOD: 4.6–13.8) QTLs associated (R^2^: 6.8–25.8%) with PN, SN and SW on eight LGs of chickpea (Table 2, Fig. 3, 4). It includes 10 major genomic regions harboring 15 PN, SN and SW-associated QTLs, which were validated and showed consistent phenotypic expression at higher LOD (7.0–13.8) across two years/seasons. These were considered as robust QTLs for controlling PN, SN and SW in chickpea (Table 2). Ten major genomic regions underlying robust QTLs covered (1.7 cM on LG8 to 3.5 cM on LG4) with 37 SNP and SSR markers were mapped on eight LGs (Table 2, Fig. 3, 4). The proportion of phenotypic variation explained (PVE) by individual robust QTL (R^2^) varied from 8.8–25.8%. The combined PVE estimated for all 15 robust QTLs was 31.7%. Ten QTLs associated with multiple traits (PN and SN) were mapped on the five different genomic regions with similar marker intervals of LGs (Fig. 3, 4). The remaining five QTLs associated only with single SW trait were mapped on five different genomic regions of LGs. The mapping and clustering of multiple QTLs controlling PN and SN particularly on a single major genomic region of eight LGs gave clues for pleiotropy and complex genetic inheritance patterns of target traits in chickpea. For PN and SN, five major genomic regions underlying 10 robust QTLs (8.8–19.8% R^2^ with LOD: 4.6–11.4) were identified and mapped on five LGs (Table 2, Fig. 3, 4). The combined PVE of all 10 PN and SN robust QTLs was 23.6%. These QTL regions covered (1.7 cm on LG8 to 3.4 cM on LG5) with 17 genetically mapped SNP and SSR markers on LGs showed positive additive gene effects for increasing pod and seed number with large effective allelic contributions from ICC 4958. For SW, five major genomic regions harbouring five robust QTLs covered (2.2 cM on LG1 to 3. 5 cM on LG4) by 20 SNP and SSR markers, were mapped on three LGs with 8.7–25.8% PVE (6.5–13.8 LOD) (Table 2, Fig. 3, 4). The combined PVE for all five SW robust QTLs was 27.6%. These SW-associated QTLs showed positive additive gene effects for increasing seed weight with major allelic contributions from ICC 4958. The SNP and SSR markers tightly linked to the PN, SN and SW trait-regulating QTLs are mentioned in the Table 2.

To determine the validity of these identified QTLs, the genomic regions harbouring the PN, SN and SW-associated QTLs were compared with that of previous QTL mapping studies involving different intra- and inter-specific chickpea mapping populations^16^,^19^,^20^,^22^,^28^,^37^,^41^,^43^,^44^,^45^,^46^. We were able to detect correspondence of three trait-influencing QTLs (*Caq'PN8.1*, *Caq'SW2.1* and *Caq'SW4.1*) identified by us with known QTLs reported earlier^22^,^28^,^37^,^44^,^45^ based on their congruent genetic or physical positions on three chickpea LGs/chromosomes. It suggests that most of the QTLs associated with three agronomic traits identified in our study are novel and may show population-specific genomic distribution on eight LGs/chromosomes. These 12 novel and robust QTLs underlying seven major genomic regions covered with different informative genomic and gene-based SNP and SSR markers, once successfully validated in diverse genetic backgrounds of populations and/or fine mapped, can be utilized for marker-assisted genetic improvement of chickpea.

### Integration of QTL mapping with comparative genome mapping and differential expression profiling to delineate candidate gene(s) at SW-influencing QTL interval

**Figure 5:**
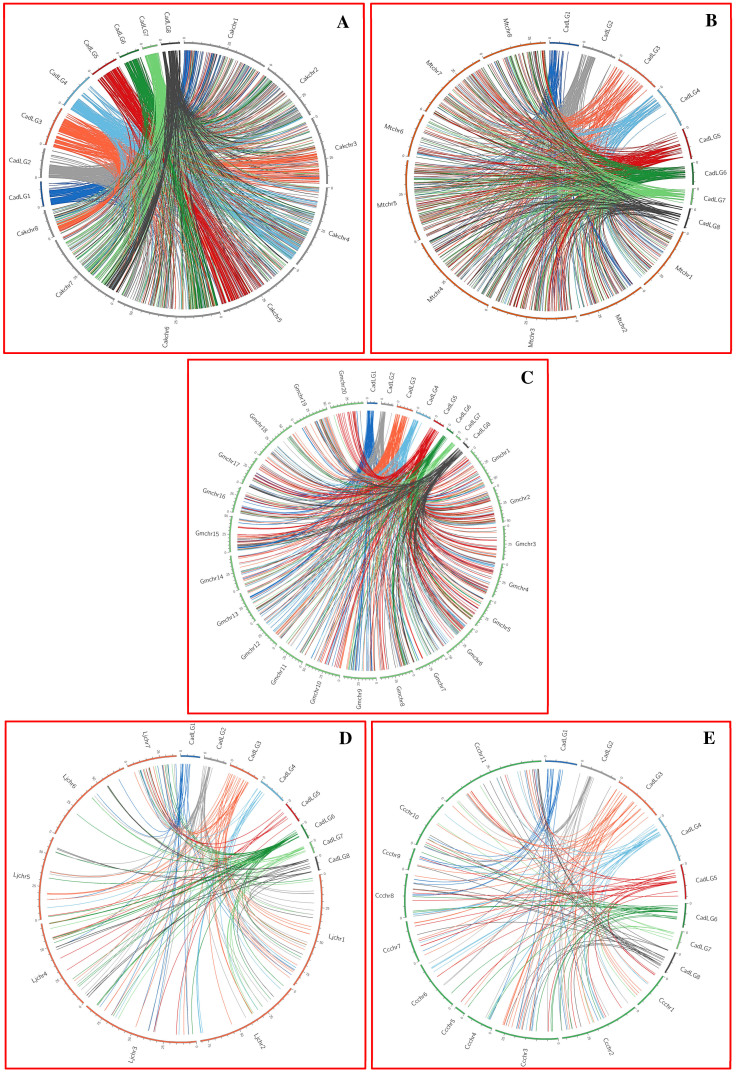
Comparative genome mapping of 2001 SNP and SSR markers genetically/physically mapped on eight *desi* chickpea LGs/chromosomes with their physical position on the pseudomolecules of *kabuli* chickpea (A), *M. truncatula* (B)*, G.* *max* (C), *L. japoincus* (D) and *C. cajan* (E) chromosomes depicted conserved syntenic relationships among five legume genomes, which are depicted in the Circos circular ideogram. A high-degree of conserved collinear synteny among the chromosomes of *desi* and *kabuli* chickpea and *Medicago* genomes was evident. The outermost circles represent the LGs/chromosomes of five legume genomes coded with different colours. The syntenic relationships of each LGs/chromosomes between two legume species are marked individually with different coloured lines.

One thousand six hundred fifty-seven of 2001 SNP and SSR markers genetically mapped on eight LGs of an intra-specific genetic map were physically mapped on eight *desi* chickpea chromosomes with an average map density of 75.0 kb (varied from 41.9 kb in chromosome 7 to 106.8 kb in chromosome 3) (Supplementary Fig. S3). Maximum number of markers were physically mapped on *desi* chromosome 3 (219 markers, 13.2%) and least on chromosome 6 (201, 12.1%) (Supplementary Table S4). The marker-based comparative genomics is useful for evolutionary studies and for transferring information from model crop species to related orphan species^53^,^5^,^54^. The integration of markers into the genetic linkage map of chickpea is expected to serve as a reference for comparative genomics in legumes as inferred from their synteny and conservation of gene order. The comparative mapping of 2001 SNP and SSR marker loci genetically and/or physically mapped (including 1657 markers) on eight LGs (chromosomes) of *desi* chickpea with their physical positions (bp) on the pseudomolecules of *kabuli* chickpea, *M. truncatula, G.* *max*, *L. japoincus* and *C. cajan* chromosomes revealed a significant conserved syntenic relationships among five legume genomes (Fig. 5). Maximum proportion of markers revealed a high-degree of homology with *kabuli* chickpea (98.9%), followed by *M. truncatula* (44.7%), *G. max* (43%), *L. japonicus* (10.3%) and minimum with *C. cajan* (9.6%) chromosomes (Supplementary Table S5–S9), which gave clues to their origin from a common ancestor. A high degree of marker-based conserved syntenic relationships and collinearity among eight chromosomes of *desi* and *kabuli* chickpea genomes was evident (Fig. 5). However, the *desi* chickpea chromosomes 1, 3, 4, 5 and 7 showed conserved collinear synteny with *Medicago* chromosomes 2, 7, 1, 3 and 4, respectively (Fig. 5). The integration of genetic/physical map with comparative genome maps identified many conserved collinear and duplicated chromosomal regions among *desi* and *kabuli* chickpea, *Medicago, Glycine*, *Lotus* and *Cajanus*.

The observed syntenic relationships among the chromosomes of five legume species are similar to the previous marker-based comparative genome mapping studies^5^,^8^,^9^. Striking synteny between chickpea and *Medicago* chromosomes is expected keeping in view their evolutionary closeness as they belong to the same clade Galegoid^47^,^48^,^60^,^61^,^62^,^63^. As compared to *Medicago*, the chromosome of *Glycine* showed a lesser degree of synteny with chickpea, which reemphasizes their distant phylogenetic relationship as *Glycine* belongs to separate clade Phaseoloid^61^,^62^,^63^. The lowest degree of marker-based synteny among chickpea, *Lotus* and *Cajanus* genomes is expected^9^,^42^,^47^,^48^. The comparative genome maps constructed among the chromosomes of five legume crop species thus would guide cloning and mapping of trait-regulatory genes/QTLs in the draft genome sequenced chickpea using the positional information of candidate genes/QTLs from completely sequenced model legume species like *Medicago* and *Glycine*.

**Figure 6:**
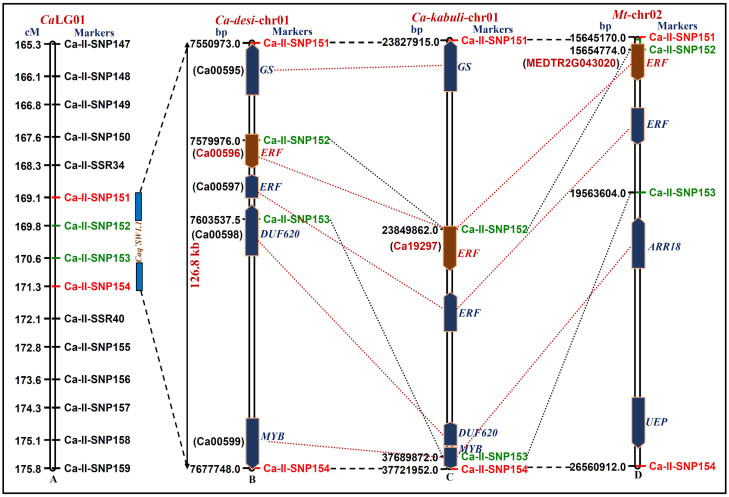
Integration of genetic (A) and physical (B) map identified and mapped one robust SW-governing major *Caq'SW1.1* QTL on 126.8 kb genomic region of *desi* chickpea chromosome 1. The marker-based comparative genome mapping revealed a high-degree of conserved collinear syntenic relationships of five candidate protein-coding *desi* genes annotated at this target genomic sequence interval with *kabuli* chickpea chromosome 1 (C) and *Medicago* chromosome 2 (D). A regulatory SNP (G/A) (Ca-II-SNP152) in a *ERF* TF gene showing strong linkage with *Caq'SW1.1* QTL and conserved synteny with *ERF* orthologous genes annotated from *kabuli* chromosome 1 (C) and *Medicago* chromosome 2 (D), was selected as potential candidate for seed weight regulation in chickpea. The genetic (cM)/physical (bp) distance and identity of the markers mapped on the chromosomes are indicated on the left and right side of the chromosomes, respectively. Red and blue dotted lines represent the gene- and marker-based syntenic relationships, respectively among *desi* and *kabuli* chickpea and *Medicago* chromosomes.

**Figure 7:**
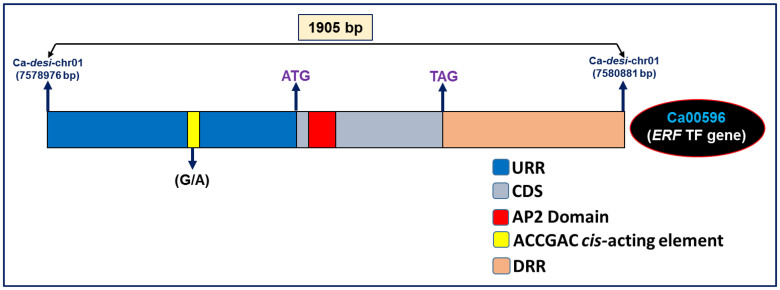
Structural annotation of one candidate SW-associated AP2-domain-containing *ERF* TF gene delineated at a major *Caq'SW1.1* QTL interval by integrating QTL mapping with comparative genome mapping and differential expression profiling. Diverse coding (functional domain) and non-coding upstream (URR) and downstream (DRR) regulatory regions of gene are highlighted. One functionally relevant SNP (G/A) identified in the DRE *cis*-acting element (ACCGAC) of *ERF* gene possibly involved in transcriptional regulation of this gene for seed weight and development in chickpea is indicated. CDS: coding sequences.

Considering the comparative genome mapping potential of SNP and SSR markers, one strong (PVE 25.8% with highest LOD 13.8) SW-associated robust QTL (*Caq'SW1.1*) region [Ca-II-SNP151 (169.1 cM) to Ca-II-SNP154 (171.3 cM)] genetically mapped on *desi* LG1 (Fig. 3, 4, Table 2), revealing conserved collinear syntenic relationships with *Medicago* chromosome 2 (Fig. 6), was selected to delineate candidate gene(s) regulating seed weight in chickpea. The integration of genetic linkage map information of markers flanking the *Caq'SW1.1* QTL with that of physical map of *desi* chickpea genome defined a 126.8 kb genomic region (spanning 7550973–7677748 bp) harbouring such major QTL on chromosome 1 (Fig. 6A and B). This target 126.8 kb *Caq'SW1.1* QTL interval in *desi* chromosome 1 corresponding to 13.9 Mb (spanning 23.8–37.7 Mb) and 11 Mb (15.6–26.6 Mb) conserved collinear genomic regions of *kabuli* chromosome 1 and *Medicago* chromosome 2, respectively (Fig. 6C and D) was structurally and functionally annotated. Five candidate protein-coding *desi* chickpea genes identified in the *Caq'SW1.1* QTL region showed conserved collinear syntenic relationships with five and four gene orthologs annotated that from *kabuli* and *Medicago* genomes, respectively (Fig. 6C and D)*.* The detailed SNP and SSR marker-based gene synteny in the *Caq'SW1.1* QTL interval among *desi* and *kabuli* chickpea chromosomes 1 and *Medicago* chromosome 2 was performed to narrow-down the possible candidate gene(s) regulating seed weight in chickpea. One SNP (G/A) (Ca-II-SNP152) in the *cis*-acting dehydration-responsive element (DRE) (ACCGAC) binding site of upstream regulatory region of AP2-domain containing *ERF* (ethylene-responsive factor) transcription factor (TF) *desi* gene (Ca00596) (Fig. 7) showing tight linkage with SW-governing *Caq'SW1.1* QTL (based on high-resolution QTL mapping, Table 2) and orthology with that of *kabuli* (Ca19297) and *Medicago* (MEDTR2G043020) *ERF* genes (known to regulate seed development and seed size/weight in crop plants, including dicots) was primarily selected (Fig. 6C and D) as potential candidate for seed weight regulation in chickpea. Interestingly, this identified SNP showing transition substitution of ‘G' nucleotide in the *cis*-acting element (ACCGAC) of *ERF* TF gene of a high seed weight mapping parental genotype (ICC 4958 with SW: 35.4 g) by another nucleotide ‘A' resulted in creation of the non-functional *cis-*element (ACCAAC) in the corresponding *ERF* gene of a low seed weight mapping parent (ICC 12968, 20.8 g).

**Figure 8:**
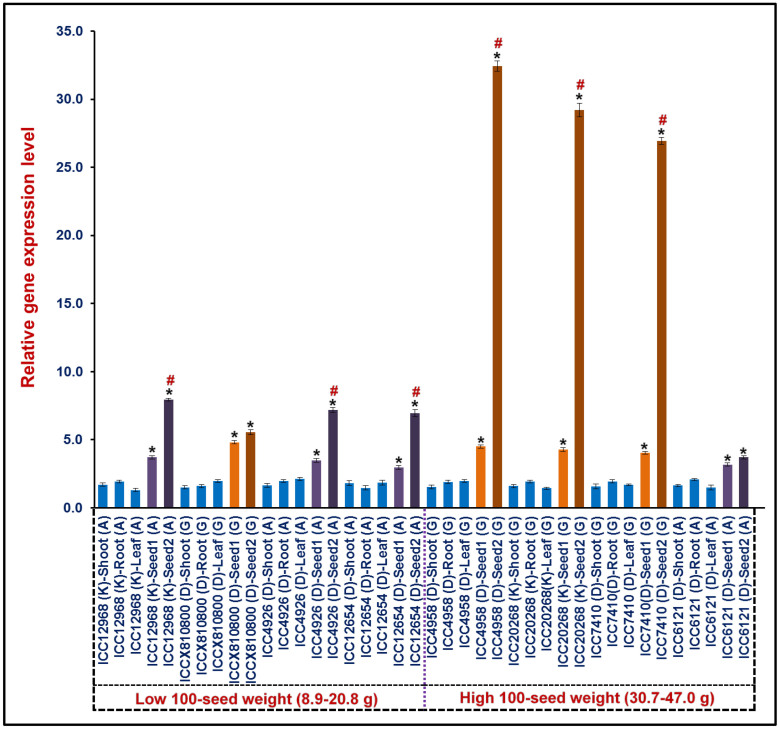
Differential expression profiling of a strong SW-associated regulatory SNP-containing *ERF* TF gene in three different vegetative (shoot, root and leaf) and two seed developmental stages (S1 and S2: Seed development stages 1 and 2 occurring at 10–20 and 21–30 days after podding, respectively) of eight low (ICC 12968, ICCX-810800, ICC 4926 and ICC 12654 with 100 seed weight: 8.9–20.8 g) and high (ICC 4958, ICC 20268, ICC 7410 and ICC 6121 with 30.7–47.0 g) seed weight contrasting chickpea genotypes as well as parents of mapping population using quantitative RT-PCR assay. The elongation factor-1 alpha gene was used as an internal control in the RT-PCR assay to normalize the expression values across different tissues/developmental stages of chickpea genotypes and mapping parents. The bars indicate mean (± standard error) of three independent biological replicates with two technical replicates for each sample used in RT-PCR. *Significant differences in gene expression at seed developmental stages of genotypes as compared to leaf at p ≤ 0.01 (LSD-ANOVA significance test). ^#^Significant differences in gene expression between S1 and S2 seed developmental stages of genotypes at p ≤ 0.001 (LSD-ANOVA significance test). The ‘G' and ‘A' SNP-alleles identified in the *cis*-acting element of *ERF* TF gene possibly regulating seed weight in *desi* (D) and *kabuli* (K) chickpea genotypes are represented.

To understand the differential regulation pattern of upstream regulatory SNP-carrying *ERF* TF gene, the expression profiling of five selected *desi* chickpea genes (including *ERF* gene) annotated in the 126.8 kb major genomic region harboring robust *Caq'SW1.1* QTL was performed. The RNA isolated from three different vegetative tissues (root, shoot and leaf) and two seed developmental stages (early cell division and late maturation phase occurring at 10–20 and 21–30 days after podding, respectively) of eight low [*kabuli*: ICC 12968 (SW: 20.8 g), *desi*: ICCX-810800 (11 g), *desi*: ICC 4926 (7.4 g) and *desi*: ICC 12654 (8.9 g)] and high [*desi*: ICC 4958 (SW: 35.4 g), *kabuli*: ICC 20268 (47 g), *desi*: ICC 7410 (32.5 g) and *desi*: ICC 6121 (30.7 g)] seed weight contrasting chickpea genotypes as well as parents of mapping population was amplified using the gene-based primers through semi-quantitative and quantitative RT-PCR assays (Supplementary Fig. S4). An *ERF* gene of these selected five genes in the *Caq'SW1.1* QTL region showed seed-specific expression as well as pronounced up-regulated expression (~4-fold) in seed developmental stages as compared to vegetative tissues (root, shoot and leaf) of all eight low and high seed weight chickpea genotypes and mapping parents (Supplementary Fig. S4, Fig. 8). Notably, the ‘G' allele-containing *cis*-acting element (ACCGAC) of *ERF* TF gene exhibited its pronounced up-regulated (~6.5 fold) pattern of expression specifically in seed developmental stages of three high seed weight *desi* and *kabuli* chickpea genotypes (ICC 4958, ICC 20268 and ICC 7410). In contrast the ‘A' allele-carrying *cis*-element (ACCAAC) of *ERF* TF gene revealed its ~3-fold lower differential up-regulation in seed developmental stages of three low seed weight *desi* and *kabuli* chickpea genotypes (ICC 12968, ICC 4926 and ICC 12654) compared to that of high seed weight genotypes. However, no significant differential expression of the ‘G' and ‘A' SNP alleles-containing *cis*-acting elements of *ERF* genes in remaining two low (ICCX-810800) and high (ICC 6121) seed weight *desi* chickpea genotypes, respectively during seed development was observed. The seed-specific pronounced differential up-regulation of this *ERF* TF gene expression particularly in high seed weight contrasting chickpea genotypes than that of low seed weight genotypes during seed development further ascertained its potential as candidates controlling seed weight in chickpea.

Comparing our present and past reports of seed weight QTLs mapped especially on chromosome 1, we observed that one regulatory SNP revealing ‘G' (high seed weight mapping parental genotype ICC 4958) to ‘A' (low seed weight mapping parent ICC 12968) transition substitution in the *cis*-acting element of *ERF* TF gene delineated at a major SW-governing *Caq'SW1.1* QTL was absent in the corresponding *ERF* gene of another low (G-allele in ICCX-810800) and high (G-allele in ICC 20268) seed weight contrasting chickpea genotypes [used earlier as mapping parents to identify a major SW QTL (*CaqSW1.1*) in chickpea]. This indicates that the two major seed weight QTLs identified in our present (*Caq'SW1.1*) and past (*CaqSW1.1*) studies using two different intra-specific mapping populations are altogether dissimilar. Therefore, TF genes harboring these two distinct major SW QTLs validated by us in two different studies using an integrated approach possibly involved in discrete transcriptional regulatory pathways governing seed development as well as seed weight in chickpea. The novelty and population-specific characteristic of this presently identified SW QTL (*Caq'SW1.1*) is further evident from its non-congruence (based on genetic/physical positions of markers flanking/tightly linked to the QTLs) with one of our earlier mapped *CaqSW1.1* QTL on the chromosome 1 of chickpea.

Collectively, the integration of QTL mapping with comparative genome mapping and expression profiling were able to delineate one regulatory SNP (G/A)-containing candidate *ERF* TF gene in a major SW-governing robust QTL (*Caq'SW1.1*) region for controlling seed weight in chickpea. Such integrated approach of high-resolution genetic/QTL mapping and marker-based comparative genome mapping (specifically between chickpea and *Medicago*) for narrowing down the QTL region into specific functionally relevant candidates have been recently implemented in chickpea for isolation/fine-mapping of a nodulation gene^64^. Three TF genes harboring a known major QTL (*CaqSW1.1*) regulating 100-seed weight mapped on chromosome 1 (on which *Caq'SW1.1* QTL identified in the present study) have been validated recently by integrating association analysis with QTL mapping, differential expression profiling and gene-based molecular haplotyping in chickpea^20^. The identified regulatory SNP-containing *ERF* gene harboring a major SW-regulating robust QTL has significance in controlling diverse transcriptional functions during seed development and determining the seed size/weight in crop plants, including legumes^65^,^66^,^67^,^68^,^69^. The SNP marker-based allelic variations in the upstream *cis*-acting elements of genes is known to regulate gene expression for controlling diverse traits of agricultural importance in crop plants^70^,^71^,^72^. In this context, novel SNP-based allelic variants identified within a functional genomic element in the upstream regulatory region of *ERF* gene is significant for understanding the seed weight regulation in chickpea. The validation of this candidate TF gene delineated at trait-influencing QTL interval is required through fine mapping and map-based cloning for its subsequent use in marker-assisted genetic improvement of chickpea. An integrated strategy established in our study for identification of seed weight candidate gene in chickpea can be applied to diverse crop plants for narrowing-down the trait-specific QTL intervals and in rapid isolation/positional cloning of functionally relevant candidate gene(s) regulating many useful agronomic traits for crop genetic enhancement.

In conclusion, high experimental validation, genotyping success rate (94–96%) and intra-specific polymorphic potential (82–96%) of 1536 SNP and 472 SSR markers showing *in silico* polymorphism between ICC 4958 (*desi*) and ICC 12968 (*kabuli*) in 190 advanced generation mapping population (ICC 4958 × ICC 12968) as well as 92 diverse *desi* and *kabuli* genotypes have suggested their immense use in large-scale genotyping applications of chickpea. An intra-specific 2001 marker-based genetic linkage map comprising of eight LGs constructed by us is highly saturated (mean map density: 0.94 cM) in contrast to previous documentation of intra-specific genetic maps in chickpea. Fifteen robust QTLs harbouring 10 major genomic regions associated with three agronomic traits, PN, SN and SW (PVE: 8.8–25.8% with LOD: 7.0–13.8) were identified and mapped on eight chickpea chromosomes. Positive additive effects of all these QTLs for high seed and pod number and seed weight were evident. An integrated approach of high-resolution QTL mapping, comprehensive marker-based comparative genome mapping and differential expression analysis have been utilized to delineate one of the strong SW-associated major genomic region (126.8 kb) underlying robust QTL (*Caq'SW1.1*). This led to identify one potential SNP (G/A) in the *cis*-acting element region of a gene encoding an ethylene responsive factor, which presumably regulate seed weight in chickpea. The functionally relevant molecular tags (markers, intra-specific genetic linkage map, high-resolution PN, SN and SW QTLs, and genes/novel alleles regulating seed weight) identified have immense utility in diverse genomics-assisted breeding applications for chickpea genetic improvement.

## Methods

### Development of an intra-specific chickpea mapping population and their phenotyping

An intra-specific F_4_ mapping population (consisting of 190 segregating individuals) derived from the bi-parental crosses between *desi* ICC 4958 [high pod (101.6 ± 2.2) and seed (137.2 ± 2.1) number/plant and high 100-seed weight (35.4 g ± 2.2)] and *kabuli* ICC 12968 [low pod (46.7 ± 2.3) and seed (54.2 ± 1.8) number/plant and low 100-seed weight (20.8 g ± 2.1)] chickpea genotypes was generated by single seed descent method. The mapping individuals along with their parental genotypes were grown (planted in a single row with 35 × 10 cm spacing) in the experimental field according to randomized complete block design (RCBD) with at least two replications for two consecutive years (2012 and 2013) during crop season at New Delhi (latitude 28.6°N and longitude 77.2°E). The mapping parental genotypes (ICC 4958 and ICC 12968) sown after every 10 rows of the RILs served as reference in field experimental design to test the homogeneity of mapping population across two seasons. The mapping population was phenotyped for three yield component traits (pod number and seed number per plant and 100-seed weight) in two experimental years/environments (environment I: 2012 and environment II: 2013). The pod number (PN) and seed number (SN) was measured as average number of fully formed pods and seeds per plant from 10–12 representative plants (selected from the middle of each row) at maturity in each of the 190 mapping individuals along with parental genotypes. The 100-seed weight (SW) was estimated by taking the average weight (g) of 100-matured seeds at 10% moisture content from 10–12 representative plants (selected from the middle of each row) of each mapping individuals and parental genotypes. The diverse statistical measures, including mean, standard deviation, coefficient of variation (CV), least square difference (LSD), analysis of variance (ANOVA), frequency distribution and Pearson's correlation coefficient of three agronomic traits in a mapping population were estimated using SPSS v17.0. The inheritance patterns of three traits under study were determined by estimating the effects of genotypes (G), experimental years/environments (E) and G × E interactions based on two-way ANOVA. The broad-sense heritability [H^2^ = σ^2^g/(σ^2^g + σ^2^ge/n + σ^2^e/nr)] was estimated using σ^2^g (genetic), σ^2^ge (G × E) and σ^2^e (error) variance with n (number of experimental years/environments) = 2 and r (number of replicates) = 2.

### High-throughput genotyping of SNP and SSR markers

A set of 1632 genomic and genic SNPs (physically mapped on eight chromosomes of ICC 4958) differentiating ICC 4958 and ICC 12968^47^,^50^ were selected for their validation and high-throughput genotyping using Illumina GoldenGate assay. For this, the chromosome-wise physically mapped 2000 SNPs (between ICC 4958 and ICC 12968) along with their 60-bp either side flanking genomic and transcript sequences were analyzed using the Illumina Assay Design Tool (ADT) to design the custom oligo pool assay (OPA). The custom made OPA, “Ca-II-OPA” contained one locus-specific oligo (LSO) and two allele-specific oligos (ASO) designed for each 2000 SNPs. The physically mapped 1632 SNPs with oligo designability ADT score ≥0.8^8^,^56^,^73^ were selected for synthesis of a custom Sentrix Array Matrix (SAM) by Illumina (San Diego, CA, USA). The GoldenGate SNP genotyping assay was performed according to the standard manufacturer's protocol with minor modifications as described earlier for crop plants, including chickpea^8^,^56^. The allele-specific oligonucleotide hybridization, allele-specific multiplexed primer extension and ligation reaction and hybridization of fluorescent dye-labeled (Cy3 and Cy5) PCR products onto a decoded SAM using the genomic DNA of 190 mapping individuals and parental genotypes were performed by Illumina BeadArray Express Reader. The intensity data for each SNP was normalized and cluster positions were assigned using Illumina GenomeStudio Genotyping software V2011.1. Minimum GenCall and GenTrain cut-off scores of 0.3 were used to assign valid genotypes at each SNP locus and for measuring the reliability of SNP detection based on distribution of genotypic classes. The cluster separation score provided by GenCall software module for 190 mapping individuals and parental genotypes was optimized manually based on degree of separation between homozygous and heterozygous clusters as normalized θ value [(2/π) Tan^-1^ (Cy5/Cy3)] in each SNP locus. 

Additionally, 500 genomic SSR markers (physically mapped on eight chromosomes of ICC 4958) showing *in silico* fragment length polymorphism between ICC 4958 and ICC 12968 based on variation in their repeats were acquired^47^. The synthesized SSR markers (normal and/or fluorescent dye-labeled) were PCR amplified in the genomic DNA of parental genotypes and 190 mapping individuals using touchdown thermal cycling profiling and standard PCR constituents as described by Jhanwar et al.^49^ and Kujur et al.^19^. The PCR products amplified by each SSR markers were resolved on 3.5% metaphor agarose gel and automated fragment analyzer. For automated fragment analysis, the amplified three fluorescent dye (FAM, VIC and NED)-labeled PCR products were multiplexed (based on different dyes and amplified fragment size) with ABI GeneScan-600 LIZ size standard (Applied Biosystems, IL, USA) and resolved in automated 96 capillary ABI 3730 xl DNA Analyzer. The electrophoregram containing trace files were analyzed using GeneMapper V4.0 following Kujur et al.^19^.

### Assessment of polymorphic potential of SSR and SNP markers

To determine the polymorphic potential of designed markers, the Illumina GoldenGate assay, gel-based assay and automated fragment analyzer were employed (following aforementioned methods) for genotyping of genome-wide physically mapped 96 SNP and 96 SSR markers (showing polymorphism between ICC 4958 and ICC 12968) in the genomic DNA of 92 *desi* and *kabuli* chickpea genotypes (Supplementary Table S10). The genotyping data of markers were used to estimate the average polymorphic alleles per marker, percent polymorphism and polymorphism information content (PIC) among *desi* and *kabuli* genotypes.

### Construction of an intra-specific genetic linkage map

The genotyping data of parental polymorphic 1536 SNP and 465 SSR markers assayed in 190 F_4_ mapping individuals (ICC 4958 × ICC 12968) were analyzed using the χ^2^-test (p < 0.05) to determine their goodness-of-fit to the expected Mendelian 1:1 segregation ratio. The linkage analysis among the markers was performed using MAPMAKER/EXP 3.0 and classified into different linkage groups (LGs). To eliminate spurious linkage among markers, the genotyping data of markers grouped by MAPMAKER were further analyzed using JoinMap 4.1 at higher LOD threshold (3.5–8.0) with Kosambi mapping function. The SNP and SSR markers were allocated into defined LGs according to their centiMorgan (cM) genetic distances and an intra-specific genetic map was constructed using MapChart v2.2. The LGs with genetically mapped markers were designated (LG1 to LG8) based on the corresponding marker physical positions (bp) on the chromosomes.

### QTL mapping

For QTL mapping, the genotyping data of SSR and SNP markers genetically mapped on eight LGs of chickpea and field phenotypic data (SN, PN and SW) of 190 mapping individuals and parental genotypes were correlated using single marker analysis, interval mapping and composite interval mapping functions of QTL Cartographer v2.5 and MapQTL v6.0. The LOD threshold score of more than 4.0 at 1000 permutations was considered significant (p < 0.05) to identify and map the major QTLs on LGs governing PN, SN and SW traits in chickpea. The positional genetic effects and phenotypic variation explained (PVE) by QTLs were evaluated at significant LOD. The multiple-trait composite interval mapping (MCIM) of QTL Cartographer was employed to detect pleiotropic QTLs. The additive effect of marker loci harboring the QTLs was determined using QTL Network v2.0. The confidence interval (CI) of each significant major QTL peaks was measured by using ±1-LOD support intervals (95% CI).

### Comparative genome mapping

The genomic and transcript sequences flanking the SNP and SSR markers that were genetically mapped on the eight LGs of chickpea were BLAST searched (E = 0) against the chromosome pseudomolecules of draft genome sequences of *desi* (ICC 4958) chickpea^47^ to determine the physical positions (bp) of markers on eight chromosomes. For comparative genome mapping, the markers genetically and/or physically mapped on eight LGs (chromosomes) of *desi* chickpea were BLAST searched (1e ≤-10) against the pseudomolecules of *kabuli* chickpea^48^, *Medicago truncatula, Glycine* *max*, *Lotus japoincus* and *Cajanus cajan*^74^ chromosomes. Reciprocal best hit method^75^,^76^ of OrthoMCL was used to define orthologous relationships of marker sequences among five dicot genomes. The marker-based syntenic relationships among chickpea and five other dicot genomes were visualized with visualization blocks using Circos 0.55.

### Differential expression profiling

To determine the differential expression patterns of genes annotated at the SW-regulating major genomic region harboring a robust QTL, suitable primer-pairs from these genes were designed for expression profiling. The gene-based primers along with internal control elongation factor 1-alpha (*EF1α*) were amplified using the RNA isolated from three different vegetative tissues (shoot, root and leaf) and two seed developmental stages [(early cell division at 10–20 days after podding (DAP) and late maturation phases 21–30 DAP as defined by Kujur et al.^19^] of eight low [*kabuli*: ICC 12968 (SW: 20.8 g), *desi*: ICCX-810800 (11 g), *desi*: ICC 4926 (7.4 g) and *desi*: ICC 12654 (8.9 g)] and high [*desi*: ICC 4958 (SW: 35.4 g), *kabuli*: ICC 20268 (47 g), *desi*: ICC 7410 (32.5 g) and *desi*: ICC 6121 (30.7 g)] seed weight contrasting chickpea genotypes as well as parents of mapping population using semi-quantitative and quantitative RT-PCR assays. The expression level of genes was compared with each other and along with control (vegetative tissues of respective genotypes) following Kujur et al.^19^.

## Author Contributions

D.B. conducted all experiments and drafted the manuscript. D.B., Y.K., S.D., S.B., T.S. and V.K. involved in genotyping, sequencing and data analysis. H.D.U., S.T., C.G., S.S. and Sh.S. helped in constitution of association panel and mapping population, and performed their phenotyping. S.K.P., D.C. and A.K.T. conceived and designed the study, guided data analysis and interpretation, participated in drafting and correcting the manuscript critically and gave the final approval of the version to be published. All authors have read and approved the final manuscript.

## Supplementary Material

Supplementary Information Supplementary information

## Figures and Tables

**Table 1:** Markers mapped on eight LGs of an integrated high-density intra-specific genetic linkage map of chickpea

Linkage groups (LGs)	Genomic and genic SSR + SNP markers mapped	Map length covered (cM)	Inter-marker distance (cM)		
			Minimum	Maximum	Average
LG1	57 + 192 = 249	210.80	0.75	1.38	0.85
LG2	59 + 192 = 251	225.37	0.85	1.29	0.90
LG3	74 + 192 = 266	261.13	0.90	1.15	0.98
LG4	90 + 192 = 282	316.55	0.91	1.21	1.12
LG5	47 + 192 = 239	262.20	0.97	1.21	1.10
LG6	45 + 192 = 237	195.57	0.68	0.94	0.82
LG7	51 + 192 = 243	213.99	0.81	1.16	0.88
LG8	42 + 192 = 234	203.25	0.83	0.89	0.87
**Total**	**465 + 1536 = 2001**	**1888.86**	**0.68**	**1.38**	**0.94**

**Table 2:** Significant QTLs associated with pod and seed number/plant and seed weight identified and mapped on eight chickpea LGs/chromosomes using an intra-specific mapping population (ICC 4958 × ICC 12968)

QTLs	LGs/chromosomes	Marker intervals with genetic positions (cM)	Markers associated with QTLs	2012			2013		
				LOD	PVE (R^2^%)	A	LOD	PVE (R^2^%)	A
*Caq'PN1.1 & Caq'SN1.1*	CaLG(Chr01)	Ca-IISNP18 (16.8) to Ca-IISNP20 (18.6)	Ca-IISNP18	NS	NS	NS	4.6	12.7	8.9
*Caq'PN1.2 & Caq'SN1.2*	CaLG(Chr01)	Ca-IISNP32 (29.5) to Ca-IISNP35 (32.1)	Ca-IISNP34	5.1	7.8	4.5	NS	NS	NS
*Caq'PN1.3 & Caq'SN1.3*	CaLG(Chr01)	Ca-IISNP116 (127.6) to Ca-IISNP119 (130.9)	Ca-IISNP116	8.5	12.4	6.3	7.8	14.7	5.4
*Caq'PN2.1 & Caq'SN2.2*	CaLG(Chr02)	Ca-IISNP330 (154.9) to Ca-IISNP332 (157.5)	Ca-IISNP331	10.5	10.8	11.4	9.8	12.4	8.9
*Caq'PN3.1 & Caq'SN3.1*	CaLG(Chr03)	Ca-IISNP398 (24.7) to Ca-IISNP401 (27.7)	Ca-IISNP399	6.4	9.4	10.9	NS	NS	NS
*Caq'PN4.1 & Caq'SN4.1*	CaLG(Chr04)	Ca-IISNP649 (12.7) to Ca-IISNP651 (123.1)	Ca-IISNP649	11.4	19.8	12.9	10.2	18.5	9.5
*Caq'PN5.1 & Caq'SN5.1*	CaLG(Chr05)	Ca-IISNP831 (89.1) to Ca-IISNP834 (92.5)	Ca-IISNP832	8.5	12.5	6.5	7.3	14.7	5.7
*Caq'PN6.1 & Caq'SN6.1*	CaLG(Chr06)	Ca-IISNP1104 (154.8) to Ca-IISNP1108 (157.6)	Ca-IISNP1106	NS	NS	NS	4.8	8.5	5.1
*Caq'PN7.1 & Caq'SN7.1*	CaLG(Chr07)	Ca-IISNP1332 (199.5) to Ca-IISNP1335 (202.2)	Ca-IISNP1335	5.7	6.8	10.2	NS	NS	NS
a*Caq'PN8.1 & Caq'SN8.1*	CaLG(Chr08)	Ca-IISNP1487 (152.9) to Ca-IISNP1489 (154.6)	Ca-IISNP1487	9.6	9.5	3.1	8.5	8.8	2.5
*Caq'SW1.1*	CaLG(Chr01)	Ca-IISNP151 (169.1) to Ca-IISNP154 (171.3)	Ca-IISNP152	13.8	25.8	10.7	12.6	24.5	8.9
*Caq'SW1.2*	CaLG(Chr01)	Ca-IISSR55 (196.5) to Ca-IISSR25 (199.5)	Ca-IISSR55	8.5	11.6	3.9	NS	NS	NS
*Caq'SW1.3*	CaLG(Chr01)	Ca-IISNP186 (202.6) to Ca-IISSR56 (204.9)	Ca-IISNP186	9.5	13.4	5.1	8.7	16.3	4.6
a*Caq'SW2.1*	CaLG(Chr02)	Ca-IISNP234 (44.2) to Ca-IISNP238 (47.6)	Ca-IISNP236	7.6	9.7	4.7	7.0	9.5	5.1
*Caq'SW3.1*	CaLG(Chr03)	Ca-IISNP465 (115.6) to Ca-IISNP468 (118.5)	Ca-IISNP466	NS	NS	NS	7.2	10.6	8.6
*Caq'SW4.1*	CaLG(Chr04)	Ca-IISNP579 (3.3) to Ca-IISNP581 (5.8)	Ca-IISNP579	8.5	10.3	4.7	7.8	11.4	3.8
a*Caq'SW4.2*	CaLG(Chr04)	Ca-IISNP749 (275.3) to Ca-IISNP752 (278.8)	Ca-IISNP751	6.9	9.8	7.1	6.5	8.5	6.7
*Caq'SW6.1*	CaLG(Chr06)	Ca-IISNP972 (13.2) to Ca-IISNP975 (16.0)	Ca-IISNP974	4.7	8.7	8.9	NS	NS	NS

*Caq'PN1.1 (*Cicer arietinum* Q**TL for pod number on chromosome 1 number 1), *Caq'SN1.2* (*Cicer arietinum* QTL for seed number on chromosome 1 number 2) and *Caq'SW2.1* (*Cicer arietinum* Q**TL for 100-seed weight on chromosome 2 number 1), PVE: Percentage of phenotypic variation explained by QTLs, A: Additive effect; positive additive effect infers alleles from ICC 4958 with increasing trait values. Details regarding Ca-IISSR and Ca-IISNP markers are provided in the Supplementary Table S1.

*^a^*known Q**TLs from previous studies by Cobos et al. (28,44), Hossain et al. (45), Varshney et al. (22) and Gowda et al. (37). NS: non-significant QTLs.
