# Supplementary material for: A combinatorial approach of comprehensive QTL-based comparative genome mapping and transcript profiling identified a seed weight-regulating candidate gene in chickpea
Source: Sci Rep. 2015 Mar 19;5:9264. doi: 10.1038/srep09264 (PMC4365403; doi:10.1038/srep09264)
Supplement: Supplementary Information [file srep09264-s1.pdf]

A combinatorial approach of comprehensive QTL-based comparative genome mapping and transcript profiling identified a seed weight-regulating candidate gene in chickpea

Deepak Bajaj[1], Hari D. Upadhyaya[2], Yusuf Khan[1], Shouvik Das[1], Saurabh Badoni[1], Tanima Shree[1], Vinod Kumar[3], Shailesh Tripathi[4], C.L.L. Gowda[2], Sube Singh[2], Shivali Sharma[2], Akhilesh K. Tyagi[1], Debasis Chattopdhyay[1], Swarup K. Parida[1]*

[1]National Institute of Plant Genome Research (NIPGR), Aruna Asaf Ali Marg, New Delhi 110067, India

[2]International Crops Research Institute for the Semi-Arid Tropics (ICRISAT), Patancheru 502324, Andhra Pradesh, India

[3]National Research Centre on Plant Biotechnology (NRCPB), New Delhi 110012, India

[4]Division of Genetics, Indian Agricultural Research Institute (IARI), New Delhi 110012, India

*Corresponding author

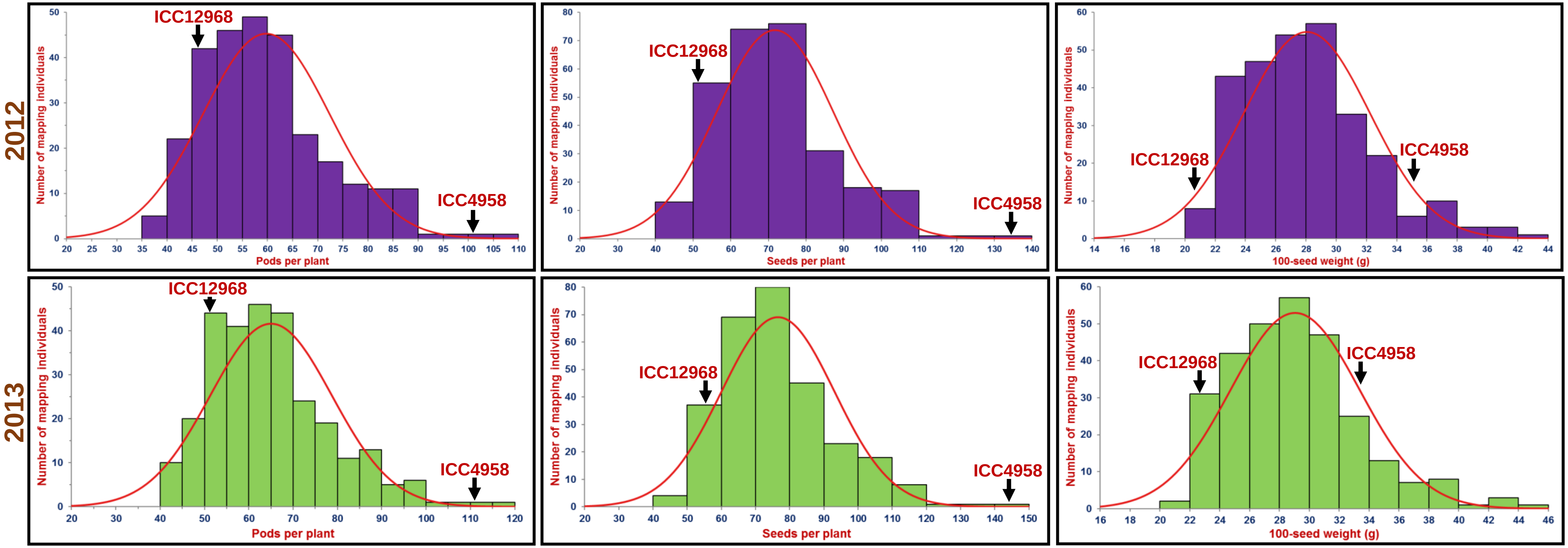

Supplementary Fig. S1: Frequency distribution of three quantitative agronomic traits, pod number per plant (PN), seed number per plant (SN) and 100-seed weight (SW) in 190 F_4 mapping individuals (ICC 4958 x ICC 12968) and parental chickpea genotypes across two experimental years (2012 and 2013) depicted a goodness of fit to the normal distribution.

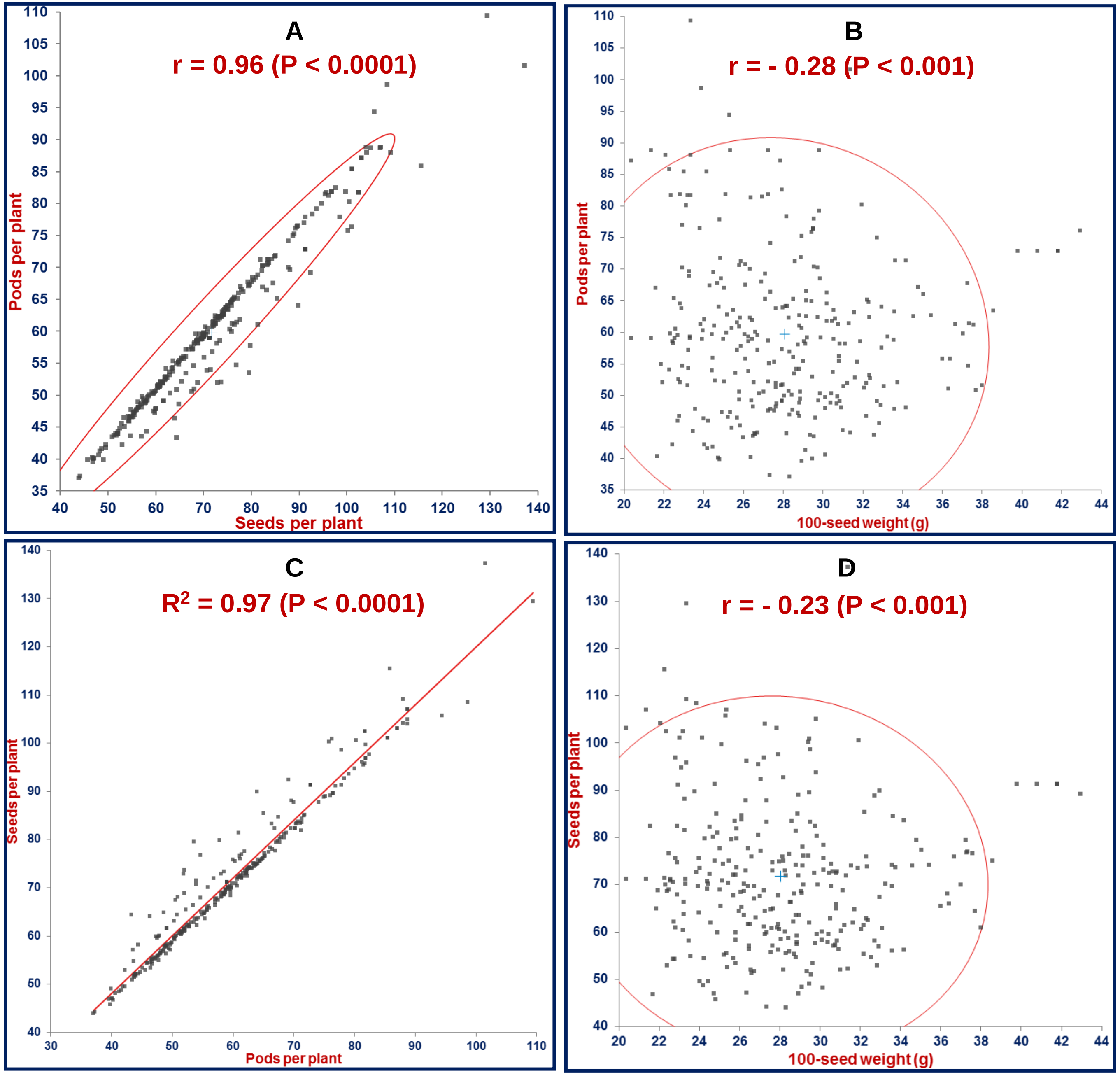

Supplementary Fig. S2: Pearson's correlation coefficients estimated among three quantitative agronomic traits (PN, SN and SW) in a mapping population (ICC 4958 x ICC 12968).

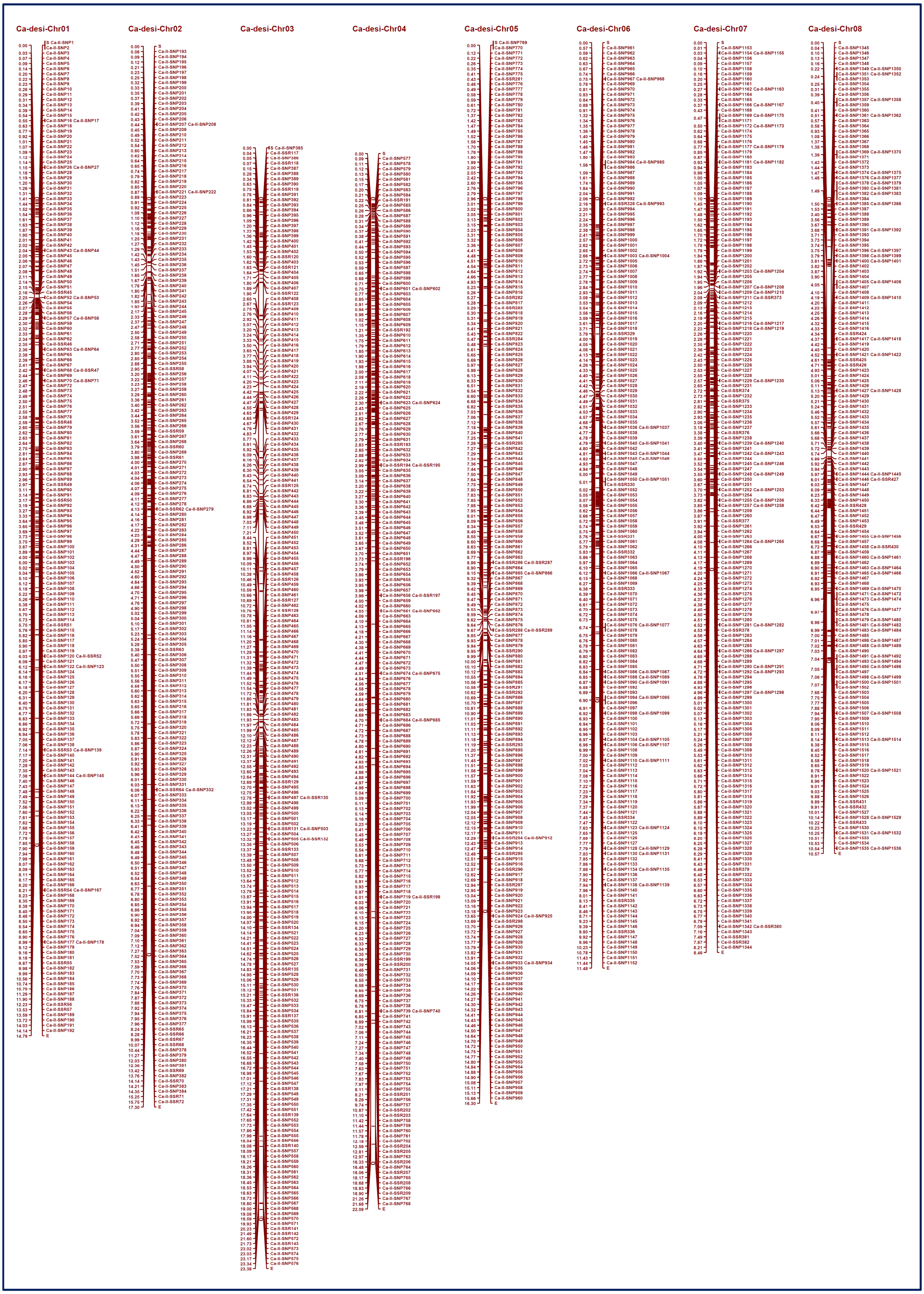

Supplementary Fig. S3: A physical map of *desi* chickpea constructed using 2008 markers (including genic and genomic 1536 SNP and 472 SSR markers) showing their genome-wide physical distribution over eight chickpea chromosomes. The physical distance (Mb) and identity of the marker loci integrated on the eight chromosomes are indicated on the left and right side of the chromosomes, respectively. The identity of markers mentioned on the right side of chromosomes that corresponds to the marker IDs “Ca-II-SNP” and “Ca-II-SSR” as mentioned details in the Supplementary Table S1. The size of the chromosomes (Mb) is based on the draft genome sequence (Pseudomolecule V1.1) of *desi* chickpea (Jain et al.[47]).

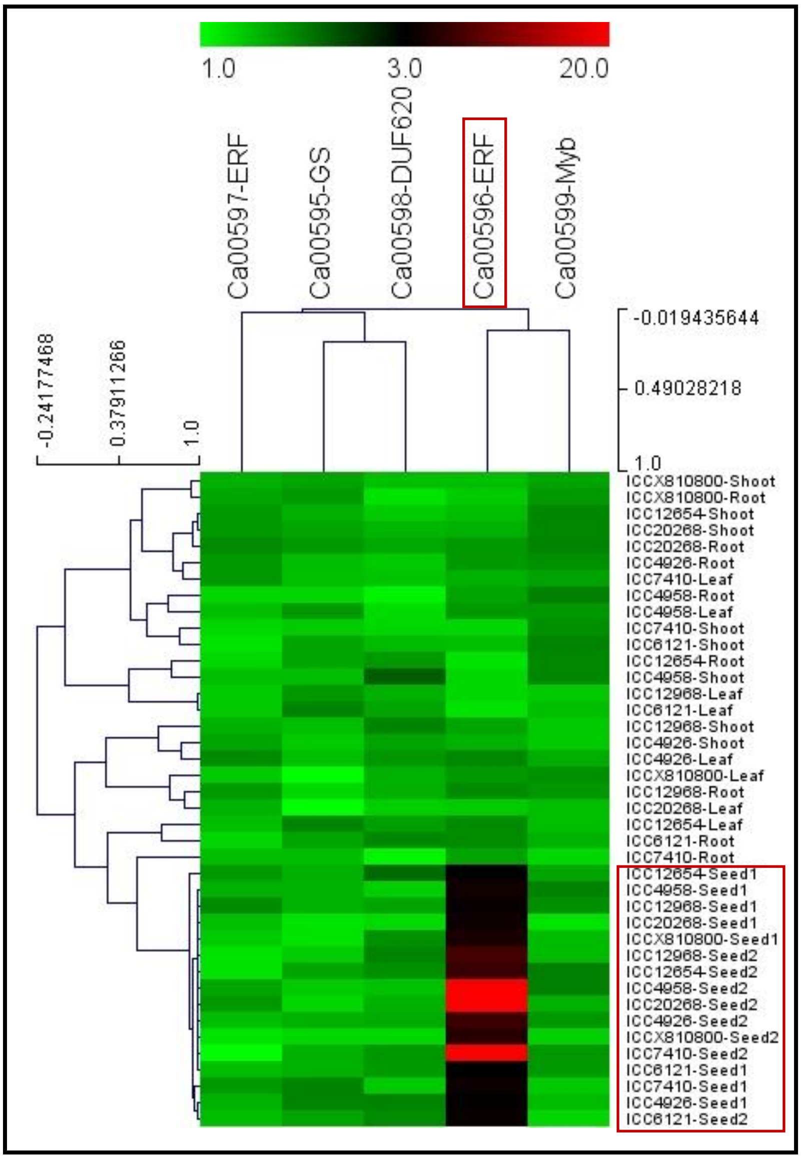

Supplementary Fig. S4: Hierarchical cluster display illustrated the differential expression profiles of five selected *desi* genes underlying a major SW-associated QTL (*CaqSW1.1*) exhibiting pronounced seed-specific expression at two seed developmental stages (S1 and S2: Seed development stages 1 and 2 occurring at 10-20 and 21-30 days after podding, respectively) compared to vegetative (shoot, root and leaf) tissues of eight low (ICC 12968, ICCX-810800, ICC 4926 and ICC 12654: 100 seed weight 8.9-20.8 g) and high (ICC 4958, ICC 20268, ICC 7410 and ICC 6121: 30.7-47.0 g) seed weight contrasting chickpea genotypes as well as parents of mapping population. The colour scale with green, black and red colours at the top signifies the low, medium and high average log signal expression values of the genes, respectively. The genes and tissues/seed developmental stages of the chickpea genotypes and mapping parents used for the expression profiling are mentioned on the top and right sides of the expression map, respectively. Details on the genes are provided in the Fig. 5. The endogenous control elongation factor-1 alpha was used in quantitative RT-PCR assay for normalization of gene expression values across different tissues/developmental stages of genotypes used. One seed-specific *ERF* TF gene revealing a higher differential up-regulation in eight chickpea accessions during seed development are indicated with red boxes.

Supplementary Table S1: Summary of SNP and SSR markers mapped on an intra-specific chickpea genetic linkage map (ICC 4958 x ICC 12968)

Marker IDs	Genetic positions (cM)	Chromosomes	Physical positions (bp)	Flanking sequences/Forward primers	ICC 12968	ICC4958	Flanking sequences/Reverse primers	Annealing temperature (0C)	Amplified Product size (bp)
Ca-II-SNP1	0.00	Ca-*desi*-LG(Chr)1	262	TTTTGAGATACTTGAGTATGATCGAAAATATTGATCTTTTGCTTGGTGCTGATTGTTGTGTTTTGTTCTTCAACTTGCAGCTGCATTTATAGACTCCAAG	A	G	TTGATGCAGCTCATCAATCTTGCTTTTGGCAGAACTTATCCAACAACTCTTTTATGACAGTTGGTCCCTTTCCAACTGACACATGAGCTGTCCTAAGCCT	NA	NA
Ca-II-SNP2	0.95	Ca-*desi*-LG(Chr)1	509	GTACTTTGTGATTTGTGTCAGTATGTCAGCCTTGAGTATAGGATGAATATTGTTTCCAGGATTGATCTTTGTTTTCCAGAACTGCTTCAAGATCAATCTG	C	T	TTGAGTGTGTTAAAACAAGATAGGAATCAAACATCACAAATCAGATCAAAGAAAGCAATTCTGGAAAACGCAGATTGATCTTGAAGCAGTTCTGGAAAAC	NA	NA
Ca-II-SNP3	2.85	Ca-*desi*-LG(Chr)1	27315	TCCGCAGAAGTCAGGCAGCTAGTAGACCACCTCAGTGAGCAAGCCAGGGAGGTGGCCAGTGAAGCTTCAATGGAAAGAACACCCAGAGCTCTTGCAATGT	A	G	TTTCCCAGAAATTCCAGGCTCGTATTAATAACCAGTATAACCCCGGACCTTTTGAGCAAGCGCTATGGAGATAGCGAATGCTAGCGTTAGCCCAGCCATC	NA	NA
Ca-II-SNP4	3.80	Ca-*desi*-LG(Chr)1	66474	GTAGAACTCTGATACAAGTTGTCTATACTAGGTTTGTCTGGGCTATTTTATCTTGGGACTTCGTGGTTAATAATCTGTCTTGCACAATTTGAGCAGTGCC	G	A	TGTTTTTGAGTTTCAAAAAATAATTTCAAAATAAATTTGTGCAATCAAAGATATTATTGGGTTGAGTCGGAGACTAAGTTGCTTTCTTTAAGACGTTTTG	NA	NA
Ca-II-SNP5	4.75	Ca-*desi*-LG(Chr)1	93741	CAACTACAGATGGGTCCCTAAATGCAAACTAAAAGTTTGGAAGGAGCCCTTACCTTCGCGGTAGCTCTAACGTGCGATTACGGCACCGTGAGTAAATCCG	A	G	TAAACGTCGTTTCAAGAAGTGATAGAAGGGAAGGTTGCTCACCTCCGTGCTACGGTGCTAGTGAACTAATTTGGAAGCGGCGTTGCGAGCGGAGATTTTA	NA	NA
Ca-II-SNP6	5.70	Ca-*desi*-LG(Chr)1	137009	TTCTTTTGGGTTGCGTTTATTTTTAAACCGGAAGACTGTACTACTTTTGTTATGTTAATATTTTGAATTCATGGATTGAGAACTTTTTCCGCTGCTTGAA	T	A	TTTTCAAAGCGAGGGTGTACTTTTTTTTCAAAAGAAATTAAAATAAAACAGAGAAATAAATAAGGTAACACCTTTGGATAAGTAATTAAGTCATTATAAT	NA	NA
Ca-II-SNP7	6.65	Ca-*desi*-LG(Chr)1	199705	AGAACTAACATATTTTTTTTATGAAGGTTCATTTTATCTCGTTGATTCTGGGTATCCATATACTAGAGGTTTGCTTCCTCCTTATAGGGGTGAAAGATAT	T	C	AAACAACGTTCAATTGTCATCCGTAGAGATGAGTGTCTGTAGTTAAATAACTCTTCGGGACTCCTAGGTTGTCTACCTTGGCCTTTATATTTTTGGGCAT	NA	NA

Marker IDs	Genetic positions (cM)	Chromosomes	Physical positions (bp)	Flanking sequences/Forward primers	ICC 12968	ICC4958	Flanking sequences/Reverse primers	Annealing temperature (0C)	Amplified Product size (bp)
Ca-II-SNP8	7.60	Ca-*desi*-LG(Chr)1	216154	GCACTTGGTTTGACTCCAATTGGACATCTACAACT<br>CAAGATATGGCTGAAATACTCTACATATGTCATGT<br>TGATTTCTCACCAAAATTCAGCACTGCACT	G	A	ATATTTTGTTGATTTTTGTTCTTTGAGTTAATAA<br>AATGTTTTGTGTTTTATTTCATTGATTAAGTATG<br>AATTTTTCGTAATTTTACATTGCGTTGTGTTT	NA	NA
Ca-II-SNP9	8.55	Ca-*desi*-LG(Chr)1	232647	CATGCTTCACATATTTTATCTTTATCAAAATTGAGT<br>TTGGGTAATCCTCTAACATGATCTAACTTTGAGAT<br>TTTTGAAATTGTTTTCATGCTGATATGTC	T	C	TAGAAAGAAAAATTTATACGTTTTATATCTTGAA<br>GAACTACCTAATGAATCTTGTTTTATGTCAATCA<br>ATAAAGATAAATGGATATGGCATAAAAGAGCT	NA	NA
Ca-II-SNP10	9.50	Ca-*desi*-LG(Chr)1	263358	TGCAAACTAGAAGTTTGGAAGGAGCCCTTACCTTA<br>GCGTTAGCTTTAACGTGGTTTTCCGGTACCGCGA<br>GTAATTCCGGTAAAATCTTCGCTCGCAACGC	C	A	ACCCCTCCGTTTCTTCTAGATCCAAACCTCATT<br>TCTCAAAGAGATAGAAGGGAAAGTTGCTCACCA<br>CCACACTACGGTGCTAGTGAACTAATTTGGAAG<br>C	NA	NA
Ca-II-SNP11	10.41	Ca-*desi*-LG(Chr)1	290259	TTGAAAAAGAAAAATTTGTGCATCATCTTCTCAACT<br>CTCTAAATCCGTTATTTGTTTGAAGATCTATGTGA<br>CATACTCTGATCATGATATCCTCTACTCT	A	C	GATATTGATTCGCCATTAAACCTTGTCCAACAA<br>TAGTCCTTGTAATTGATTATGTGTATCTTGATAT<br>GGATGATTTTGTGTATCTTGATATGGAGATAGA	NA	NA
Ca-II-SNP12	11.32	Ca-*desi*-LG(Chr)1	307564	GTTTTTACTACTACACGGTTTTTGTTGTTATTACTA<br>GTTCAGTTTTATGTTGATGATTATATATTAAGTTTT<br>TTCCATGATTATCCTTTTATATGTTGCC	A	T	AATATAAAAAAAGTATATGACCAAGACGTTCTCT<br>CCACTGAATTAAAAATAAAATAAATAAATTGAAG<br>ATTTCAACTTGGGTTATACTTGTTTGATATGA	NA	NA
Ca-II-SNP13	12.23	Ca-*desi*-LG(Chr)1	342387	TGTTTGTGTTTGACTGGCTTGGCCTTGCGTTTGC<br>GAAATCGCAGTTATTACAAGTTAATGAGTCTTGAA<br>GTTCAAGTTTGAGAGAAACCTCGCTCTGATA	A	G	TTAATTCCAAAAATTTACTAGTACTAAGGTTTTC<br>ATATAAAAAGTCATTTGTAATTACATAACTAAAA<br>TATAAATTACAACTCTAAATTCCTGGTATCAC	NA	NA
Ca-II-SNP14	13.14	Ca-*desi*-LG(Chr)1	388218	CCATATGCTTCCTTGTATATGCCTTTAAGCAAGTG<br>CCACAAGTGAGATTGAACGTCGTCCAAACAATGTT<br>TTTCAATCATACATTGTGCAACAGCGGAGC	G	A	AAGGTGTTCAGATAACATTATGAGACGGGATAT<br>GGACAGGCATTGTATAACTGTTTGTCCAATGAA<br>GCTTGTAAATTGTCCTTTTTATGCAGTCGGTTG<br>T	NA	NA
Ca-II-SNP15	14.05	Ca-*desi*-LG(Chr)1	544347	TGCAAACTAGAAGTTTGGAAGGAGCCCTTACCTTA<br>GCGTTAGCTTTAACGTGGTTTTCCGGTACCGCGA<br>GTAATTCCGGTAAAATCTTCGCTCGCAACGC	G	A	CTGCAACATCCGCAGAGCAGTGTATAACTTAGC<br>TGACAGATTCAAGTCTACTTCAGTTTGATGTTG<br>GTCAGGAATGTCAAAGGGAAGATTCAGCTTCC<br>CAGATGTCAGAGAACCTTTGGCTGGAGTTGGA<br>TCACATTTCCCTGGCTTTGATGCTTTCACAAGA<br>GACGGGAATCTCAATATAATTCTTTGCCCACCA<br>AACAGCACATTCTGAACCTTTGCTTCATTTACC<br>[illegible]	NA	NA

Marker IDs	Genetic positions (cM)	Chromosomes	Physical positions (bp)	Flanking sequences/Forward primers	ICC 12968	ICC4958	Flanking sequences/Reverse primers	Annealing temperature (0C)	Amplified Product size (bp)
Ca-II-SNP16	14.96	Ca-*desi*-LG(Chr)1	547496	TTGAAAAAGAAAAATTTGTGCATCATCTTCTCAACTCTCTAAATCCGTTATTTGTTTGAAGATCTATGTGACATACTCTGATCATGATATCCTCTACTCT	G	A	GCACGACTCCAGCAGAGCCAAATAGACAGCATTGAACACCCATGCCAAATAATTACTTGGGTGTAGAGGGGTGGCTTCTGACAATTTTGGGTGTAGAGGGGTGGCTTCTGGTTTGGGCTTTATTTTAAGTTTGCTCCATGCTATTGTCTGTTATGGAAATGTTGATGGTAGAAGAGCTCATATGTCCAACCTCCCTAGTGATATACAAATTCAAG	NA	NA
Ca-II-SNP17	15.87	Ca-*desi*-LG(Chr)1	549097	CAGTCATTGAAAGTCCATTTTTCTGCCACTGTTCTAACAGCACATCTATAGCTTCCATTTCTGCATGTCTAGTAGCCTGTAAAAATTGATTTAGCAATAA	C	T	ATATTTCATCAATACTTTATGCAATTCACTAATCATGCATTTGTTTTAGATTTTCTTCATTACTATTATGTCTTTTTCGTACCTGTGAATTTCTTCTGTT	NA	NA
Ca-II-SNP18	16.78	Ca-*desi*-LG(Chr)1	627392	TTCTGACTGTTCTCTCCTAAATAGTGAATGTTTGGTTGAGCTTGTATGGGCTTAACTTATTGCATAAATTTGCATACATGCTTGAAAAAACTTATGAAAA	G	C	AAATCCAGCATAAACTGAAAACAACTTATGACAATAAGCAAGCAAACTGGAGGAGCTTTCTGAAAATAAGTTGAAAACAACTTATGGAATATCACAAGCT	NA	NA
Ca-II-SNP19	17.69	Ca-*desi*-LG(Chr)1	770437	GCTCATTTTCTCATTCAGAATGGTTACATTAAGGTTCCTCCATTTTTTTAATTGAATTGTCACCTTCACTCTTCTATTCTAGTAATTAGTAACGATTAAT	T	A	TTTATTTCCAAACGCTGTCTAGGATAACTCTTTATAACAACTAATAGCTTATATGAAAATTGTTTGACTTAATGATTTATATGATAAGCACTTATAACTT	NA	NA
Ca-II-SNP20	18.60	Ca-*desi*-LG(Chr)1	923863	GACTTTGTGATGGCACTAATTGATTCTTCTTTTTCATCTCTTTACTTTTACCCCATACAACCGCATACAAGCCACATACTATTAACACTGCTCCAATGAT	G	A	TTGTTTACCTAATTTTTTAGTTGAATAAAAAAGTTATTATGGATCAAATTGTATTGATTTTAATGGCTCACTTGAACTTTATTGATTTTGTTAATTTCAG	NA	NA
Ca-II-SNP21	19.51	Ca-*desi*-LG(Chr)1	1009711	CTTATGAAATATTAATTCCATGTGTTAAATTTTCTGATGAAATCATTTATTTTTTCCTTCCATCTATGTCTGCCAATTTTCAGTGTAATTGTTGAAGCCT	T	C	ACAGGATGCAAATCAATACCACAATCAACTTAATTTTCTTACAACTCTTCGTAGAATAGGCTCCAGTGACGAGCAAAGTTTCTGCAGACTGTCCACCTTC	NA	NA
Ca-II-SNP22	20.42	Ca-*desi*-LG(Chr)1	1066513	GACTTTGTGATGGCACTAATTGATTCTTCTTTTTCATCTCTTTACTTTTACCCCATACAACCGCATACAAGCCACATACTATTAACACTGCTCCAATGAT	A	G	AAAGGATAAAGATAGATAGGTCCCAAAGACAATTGATATACTCTTCTCACTTCTTAGCAAGGAAACTCAATTCGCCAAGTGGAAAAGAAAGAAAGAAAAACTGATGATTTCTCTACAACCTCATAGATCATGTCTTCAAACAACATTTCTATTCAAGAATTCCAACACTACTATACAACATAGACAAACACAAACAAGCCGAATAGCAGAAAGAAAGAGTAAAGTACATAGA[illegible]	NA	NA
Ca-II-SNP23	21.33	Ca-*desi*-LG(Chr)1	1089631	TAGTTTTTTGTTGTTTTTTGATGTTGAAACGGTCTTTCTTTACCCATGGGCAATGAGTCCTCCTCTTTCCGGACAACACATACAAAGAGACCCGCCAACA	G	T	TCTAGTGAATAACTAAAGAAAATAGTAGAAAAGATAGATGGGAGATAGAAGAGAAACAATTTAATATAAACATCTCCCATAAGTTCACTAATTATGTTTT	NA	NA

Marker IDs	Genetic positions (cM)	Chromosomes	Physical positions (bp)	Flanking sequences/Forward primers	ICC 12968	ICC4958	Flanking sequences/Reverse primers	Annealing temperature (0C)	Amplified Product size (bp)
Ca-II-SNP24	22.24	Ca-*desi*-LG(Chr)1	1120966	TGTTTAAATTATTTTTATGTCTTTTTTTTTTTGTTGG<br>TGGAAATTGCATTCATTCAGGTCACTTTAATGACA<br>TTAACACCCTCTAATTTTGAGAAAACAA	C	T	ATCAAACATGTTTTTTGTCACACTTATATATATT<br>TCACTATGTTCTTAATAGCACAATTAATATATAT<br>ACACAATTTTTTTAATAAGTATAGAAAAGGTC	NA	NA
Ca-II-SNP25	23.15	Ca-*desi*-LG(Chr)1	1136888	GTCCTACTAACATTAGTCCTCTGAGTTTGTAGCAG<br>GGGAAACCATGGTTAATTAGGCTCATTGTCACATC<br>ATGTCTTTCTCTTGCTTCAAAAATGAACAA	T	C	CAATATTATAGTTTGCAGTGTATCTGTGACCTC<br>AACTGCAAATTAGAACTATGATTATTATTCGAAG<br>ATTTACCTGCAAGTTGGAAAATGATAAATGTGT	NA	NA
Ca-II-SNP26	24.06	Ca-*desi*-LG(Chr)1	1145410	TTTTGCAAAATACGTGCCCACATGAACTGAGGGC<br>CACAAGTGACATTGTGTTGGTGAGAGTGGTTTTG<br>CAGCTAGGACAAATGTAGGTCCTATCAAGAGC	A	G	CTACCAGTACTCTCTGTCCAGAAGGTAACGAGA<br>AACTGAAGTTAAAGACACTTTTCCCTGTCCAGT<br>TCACTGAAGATACTAGTGAGCAGAAAAAATCCA<br>A	NA	NA
Ca-II-SNP27	24.97	Ca-*desi*-LG(Chr)1	1147254	TTGGGACGTGAAGTGGAGAAGTGGAGTATCTCAT<br>GAGAATCATTGAGTTAGTCATGGGCAGAGCAGAA<br>AGATCTGTTCTCTAAATGAGCCTAGCTCTGGG	A	T	ATATATATATATATATATATCTGATGTATTGCCA<br>ACATATTACAATTCAATAAATATATTTTTACTCTA<br>ATTCTTGAGACAGAGGAATGAATATTCCTGC	NA	NA
Ca-II-SNP28	25.88	Ca-*desi*-LG(Chr)1	1184107	TACTGGACTGGACAAGGCGATTTAACATTATATGT<br>GGAATAGCTAGAGGGCTTCTTTATCTGCATCATGA<br>TTCAAGACTCAGGATTATCCATAGAGATCT	C	T	ATGTATTTGCTTCTGTTTGATCTTTGCCGAATAT<br>TCTAGCCATCCCAAAGTCTGATATTTTAGGGTT<br>CATTTCGCTGTCAAGTAGAATGTTGCTTGCCTT	NA	NA
Ca-II-SNP29	26.79	Ca-*desi*-LG(Chr)1	1188582	GTCCTACTAACATTAGTCCTCTGAGTTTGTAGCAG<br>GGGAAACCATGGTTAATTAGGCTCATTGTCACATC<br>ATGTCTTTCTCTTGCTTCAAAAATGAACAA	G	A	GGCGTCGAGATTCGAGTAACATCATGGAAAAG<br>CCAAGATGATCCATCAAGTGGTGATGGTTATTT<br>CAGTCTTGATTATCATGGTATTCCAGATATTTAC<br>TTGTGGAACAAGCAACAAAGGGTGTTTAGAAGT<br>GGATCATGGAATGGTGAGAGTTTTGGTGGAGT<br>ACCAATACTAAAT	NA	NA
Ca-II-SNP30	27.70	Ca-*desi*-LG(Chr)1	1203450	GTTCACCTTATGGTTCAGGAACATATGCAGGGGA<br>TGGCTCAAGACAGCCTAGTGAGTTAGAATTGGCT<br>CAAGCTTTTCATCAGGGGAAGTACTTTGCTGG	A	T	ATACATAATTATAAATGACAATATGTTAAGTGGA<br>TACATAATACTTAATGAAAGATATGAAGGACATA<br>AATCATTGTGGTCCCTTGAGCTTCTTAGCAAT	NA	NA
Ca-II-SNP31	28.61	Ca-*desi*-LG(Chr)1	1262058	TTGGTGTTAGGGCTTTGGCTTCTTATCCACTTAGG<br>TCCAATAAAAAAGGTACTGGTGAAAAGCATGTTAC<br>TGTCTATGTTGGAGGGACTTTTATTCGTCA	T	A	GTGAAAGCACATTTGAGCTCTGGTAGAGTCTA<br>GTATTTCAGATAGACAACTCAAACTTAGAAACA<br>ATGATACCATCATTATCTGCATAGAGCCATTCT<br>CC	NA	NA

Marker IDs	Genetic positions (cM)	Chromosomes	Physical positions (bp)	Flanking sequences/Forward primers	ICC 12968	ICC4958	Flanking sequences/Reverse primers	Annealing temperature (0C)	Amplified Product size (bp)
Ca-II-SNP32	29.49	Ca-*desi*-LG(Chr)1	1306596	GGCAATCGATTGAGTGAGTATTGAGCTCCCAGGTTTTAAGCCAATCGATTTCCAAATCGATTTGGTCGAATATGAATGAGTCCCTGACTTAAGGCCCAAT	C	T	TGGATGGTAGACAGGGACTTGGCCAAATGGACAAAATCGATTAGCCAATCGATTTTGTAATCAGTTTTCGAAAATCATTTACGAAATCGATTGCCCAATC	NA	NA
Ca-II-SNP33	30.37	Ca-*desi*-LG(Chr)1	1413807	AATGGTCATTTTTTCTTCTTTCTTTTATGGCTACCTTTTTGAGATTATTTACTGCTACAACTTGTAAGATTTTTTTAGAGAGCTAATGAAAACAATTTAT	G	A	TAAAATAAAGTCATACTTTTCTTATATAAACTATAAGTTGTTTTTATTAACTATCCTAGAGAGATTGTAAAAATAAATTGAAAACAACATATTGACATGT	NA	NA
Ca-II-SNP34	31.25	Ca-*desi*-LG(Chr)1	1440076	ATTTCTCAAACTCGTGTGATGTATACTCACTACCTCCATTTGTCCTTAGCAATTTGATAGTTTTTCCAATTTGTTTTTCAATTTTCACTTTGAAATTCTT	G	A	CACTGGGAGGTAATAAGTACTTCATAATATTTGTTGATGAATTTAGTAGAATATTGTTGATTTATTTGATAAAGACCAAAGATAAAGTATTTGAAATATT	NA	NA
Ca-II-SNP35	32.13	Ca-*desi*-LG(Chr)1	1524692	AATGGTCATTTTTTCTTCTTTCTTTTATGGCTACCTTTTTGAGATTATTTACTGCTACAACTTGTAAGATTTTTTTAGAGAGCTAATGAAAACAATTTAT	G	A	GCGGATTTTTACCAAACCAGTTCACTGGCCCTCCTGTCCTGCCTACGATCTCAACTGTTCTGTTTGCTAAAAACCATGTGGCAAGAAAATGAAATGATAGAAAGCAGAACTTGGGGAGTAAAGGCAGATTTGGGTAAATCTAAAACATTGCAGCGTCTATTTGGTTTCAAAAAAATTATTTCATTTTCTGTTATTGCTTTTATTTGCGATACA	NA	NA
Ca-II-SNP36	33.01	Ca-*desi*-LG(Chr)1	1536229	CTATGAGTCTTGTCCATACTATCCTTTTTCTCCTTTTCTGGTAGTGCGGGTGTCATGTCTTTCTCTCCTCCCAGTGCTCCTGCCAAACCTTGATGAACCA	A	G	TATTCGATTCAAAGGAAAGATGACATAAACAAAGTATGTACTTGAAAAATTCGATGGGAAGAATGATTTTGTGTTACGGCGTCTCAAGATGAAAGCGTTG	NA	NA
Ca-II-SNP37	33.89	Ca-*desi*-LG(Chr)1	1609264	AAACTCAAATGTTAATTTTTTTCCCCTAATCTGTAATCATCTATTCCTACATCTGTTACTATTACACAAATTCATGCTCTCTATGATGTCATAGGTGGTT	C	T	TTTACCTTTTCGGGTTAAAATTGTCGACAAGAAGGTCCTCATCCCAAAGAAATATATAATTGTAGTCGGCAACTATATCTGGATGCAAAAACCGTTTTGC	NA	NA
Ca-II-SNP38	34.77	Ca-*desi*-LG(Chr)1	1628335	TCGGATTAAGATGTCAAATATTGATTTCCCTCCCTTAGTCAATCTATTTTCTGCATTTCACTTCTTTTATTAATTTCCCCCTTTTCCTTGTTTTTTATTC	A	T	TGTGTTGACACAATATTAATATGAATCCTTCATGAAACAATACATAAAAATGTTAGAAGTGTATGATGCTAATGGTATAGCCAAAAAAGGAGTCAGACAT	NA	NA
Ca-II-SNP39	35.65	Ca-*desi*-LG(Chr)1	1870704	AATCGATCGGTGGGTTTGTGAGTCATTGCGAGTGGGACTTGGTGTTGGAAGGTATGGTTGCTAGAGTGCCAATGATTGCATGGCCATTGTATGCAGAGCA	G	A	AAAGTCTTCAGTTGAACTTCCAGTTTGAATGGCCATATATGTAGAGTATGTAAAATGTAAGAAGTGATGTAAAATTAGGCTACAAGTCCATGATAGCATG	NA	NA

Marker IDs	Genetic positions (cM)	Chromosomes	Physical positions (bp)	Flanking sequences/Forward primers	ICC 12968	ICC4958	Flanking sequences/Reverse primers	Annealing temperature (0C)	Amplified Product size (bp)
Ca-II-SNP40	36.53	Ca-*desi*-LG(Chr)1	1946438	GCCTCATTCCCACTACCTCTAGAACATATTTTATTTATTTTAATTTGATTGTGAGTGAATAATGTCATGAGATTTATTTCATATATTATGTGCACTTCTT	G	C	TGTACACCACCATATGAAGCCATGAACTCAACAGGAAACACCCACAACCAACATTGCATTACATCTGACCTCCTTTTCATCATCACACATACCCTTGTAT	NA	NA
Ca-II-SNP41	37.41	Ca-*desi*-LG(Chr)1	2008908	TGCTGTGATTGCAGAATGTGGTGATGCTTGGTTTAATACTCAGAAATTGAAACTTCCACAAGGATGCGGGTAACCATTTTTTTATTCAAATTTCATTAAG	A	G	ACCAATCGACCAACCTAATGATGCATATTGCAGTTGAGCTTCAAACCTATAAAATAAAATAAAAAGCAATGAAATATTGGCATGATTTATATTACTATAT	NA	NA
Ca-II-SNP42	38.29	Ca-*desi*-LG(Chr)1	2019308	AAGGCCATTGATATTTCTGGTGTCTCTATGCTGGTAGGTTTTCTTTATAATGTTCCATGTAGAGAAGTCTTTCTTCTTTTGGCTACTATCATACACTTAT	C	G	AACATGAAGTTAGAAGATAAAAAAAGTTTGAAATGTGCTTGCAAGTTCCTGTTTAAGATGGTTGCTTTCAGTCAAAATTATTTCACCAATAATAACAACG	NA	NA
Ca-II-SNP43	39.17	Ca-*desi*-LG(Chr)1	2036194	AACCACATTTCATTCCTGAGACTGAAAATAGTTATGGCTTATATGGCCATGATGACTCTGATCAACTTCCTTCCACCACCACTGCCACTTACCATCCTTC	C	A	GGTTATTCTCAAAAGTGTCCTTGTTGTAATACTTTGTGTTATATGCATCATTATTGTAGTACTTGTTGTTGTATTTGTTGGTGTCTTCAAATTGAGTCTT	NA	NA
Ca-II-SNP44	40.05	Ca-*desi*-LG(Chr)1	2040493	TATTTCAAAAATTAGTCCAATATTTTCTTTGAAAAAATGGGGCAGCCCGAAGTCTTGAGGGTTAGCCCGACCCGACCCATTTGTAAACTTGATCAATTGG	A	G	ATATGTAGAGCTATCAAAATGGTGTGGTCTATTAGCTCACATTATAGGTCATCAGATTGTAAAAAAGTTGATCCAAAAATAAAAATCTTTTCTAGTCCAA	NA	NA
Ca-II-SNP45	40.93	Ca-*desi*-LG(Chr)1	2050795	TCACCCTGATATTGGAATAGATGGCCCTGGTTTTCAATGTGATTTAACTATGCTTTAGACAAAGTAGCTAATCCAGACTATCACTCAATTGGTTGCTTTC	A	C	TCGTTCTTGAGCAAATAATCCTAACCCAATCAAAATTATATAGAGAAGTTCCATTTTCTTGCAATAAGTTACTCAAAGTCAATAGGGTCCTCATCATCTT	NA	NA
Ca-II-SNP46	41.81	Ca-*desi*-LG(Chr)1	2056298	TTTCTATTTAAGTACCAAGCTAGGTGTTTGACTTTGTTTTTATTTAGTATTATTACTTTATTAATATATTACCATTATATAAATTATAGTCCTTATGTGT	T	C	GATATTGAATTTAATAATATCATTTATGAATTGAAGGATAGCTGAATGACTTCTTTTTCTTGAAAGGTATACCTGAGTATTGGATGAACTCATTTTTTCT	NA	NA
Ca-II-SNP47	42.69	Ca-*desi*-LG(Chr)1	2073370	TCTAATCCATAATTCCTTGAAAGTCGTAGAGTTTGGATTTAATCTCATCAGTTTACGACATATTCTCTCAACCCTAATTCCTTGAAAGCTGTAGAGTTTG	G	A	CATATAAAGACAAAGAAAGAGACATAAACACGATTCAAGGTGTTCAAAGGGAGTAATTTACACACTTGAGAGAATATGTCGTAAACTGAGGAGATTAAAT	NA	NA

Marker IDs	Genetic positions (cM)	Chromosomes	Physical positions (bp)	Flanking sequences/Forward primers	ICC 12968	ICC4958	Flanking sequences/Reverse primers	Annealing temperature (0C)	Amplified Product size (bp)
Ca-II-SNP48	43.57	Ca-*desi*-LG(Chr)1	2084773	CGCGAATCTCAGCTGCATAGCGTCCCCATGGTCGCTTTCTAACACCTCTGTAGTGTCCTTCGCGGTTAGCTGAAGAAGATGAAGATGAAGAATCCATTAA	C	G	CGGCCAGCCGACAATTACGGGAGCAAATGTGGGTCCCATCACTTTTATAAACATCCATCCATTCCAACACAATCACTTCAAATCTCTTTTTCTTTTCTTT	NA	NA
Ca-II-SNP49	44.45	Ca-*desi*-LG(Chr)1	2109647	GTTGTCGCATCGACAATTTGGTCCATATATATCTATAGAGGTGTCTACATATTGGTGGATATTACCAAGGTAGTATTATCATAGTATAGCATCTCTATCT	T	G	TGGGAAACCTCACGTAAGCCCAAAAGACAACCGCAACAAAGACAAAAATTTAAGATATAACTAGTCTTTTTTAGGCTCATTAAGATTTGACAATGACCTT	NA	NA
Ca-II-SNP50	45.33	Ca-*desi*-LG(Chr)1	2140927	ATGGCTGTGTATATGTTTATTCATGCGGGTATTGACCGGCCCAAAAGAAGGTTAATATTTGGCAGAATTAAATGGTACACCTGCGATGATAAATGTGTGA	T	C	TGGTACTCTTGTTGCAACGATCAAATATTAACAATAAGACATGTCAAACAATAAACCTTCCTTTGGGTCGATTTACTGCTCACATGTATACCAAATAATT	NA	NA
Ca-II-SNP51	46.21	Ca-*desi*-LG(Chr)1	2148209	ATCATTCCTCGTACCTTTCTCTTGCCTTTTTGCTACTTTTCTTCAAAGACCTTTCTTTAAACATTTTTAATAGTATTTTGTCTTTATTGAATGAATCAAA	C	T	CTCGCTGATTTTAAATTCATTAAACAAAGGCATAATAGTATTAAAGAATGAATTATTTGAATTCATTCAACAAGGGCAGAATAGTATTAAAGAATGAATT	NA	NA
Ca-II-SSR45	47.09	Ca-*desi*-LG(Chr)1	2162549	CTGTGAGAAAATGGTTGCCA	(CT)9	(CT)10	TCCAAAAGCAAAGCAACAAA	59.69	138.00
Ca-II-SNP52	48.87	Ca-*desi*-LG(Chr)1	2249502	TATTTTGGGGAATCCAACCACCTTCCCTTGTCTCAAAGAGCCACAAAAATTACTTTGATAACGTTGCAACTATTTTTCTCTGTATACGGGCACTTCTAAG	A	T	AAAAGTGTCCCGTGTGGTACAATAGTTGAAAATTGTTCAAACAAAAATATGTGAAATCAAACAATTTTTAAAGTTTGACTCATCGTAGGACATACAATAC	NA	NA
Ca-II-SNP53	49.77	Ca-*desi*-LG(Chr)1	2251306	TGTTTGGGTGCTAGGTTTGGGCCTAAGCCCAATAAGGGGGGTGAGTACGGAAGTGAGGGGAGAAATGCTTATGGTGCAGAAGGGGGTAAGCATCTGTATC	A	G	AGGAACCCAAATCAAGGTGAAATTGATAAATTGTATTATTGATTGAAAATAAGGAGAATGTATTACAAGAGGATCTCTCCTGTAAACTGTAACTCCTGAA	NA	NA
Ca-II-SNP54	50.67	Ca-*desi*-LG(Chr)1	2261743	GGTTCTGCACCAGAGCCAATGTTCCGTCACGCTCAAGGGAAGTTTGAAATTGCTCTAATACCTTGACAATTTGCCAGAAATCAGGCCTCTTATCTGGTTG	T	C	CTGTTATGTGATAATGTACTTTTCTTCAGAAATTAAGGCCGGTAATTCCTTCAGACTGTCCACCTGCAATGCGAGCTTTAATCGAGCAATGTTGGTCGTT	NA	NA

Marker IDs	Genetic positions (cM)	Chromosomes	Physical positions (bp)	Flanking sequences/Forward primers	ICC 12968	ICC4958	Flanking sequences/Reverse primers	Annealing temperature (0C)	Amplified Product size (bp)
Ca-II-SNP55	51.57	Ca-*desi*-LG(Chr)1	2272101	AACACTGATGTTGGGTGACCCAAAACCTTCGATTCATCTCCTCATTGGTATGAGTTTGATCCTTTTCTTCATCATCATCATCATCGAAATCCATTTGAAG	G	T	ACACTAGTAGTACTATTGCTGATGTTGAGTTTCAATTTCTTGATGATGATGATGAGGTATTATCTTGTCAAAGTTCTAGTAGTGATGATCAGTGTCATTC	NA	NA
Ca-II-SNP56	52.47	Ca-*desi*-LG(Chr)1	2282239	TAATTTTTTTTCTTCGTATTGTTTGTTGTGATCTCTTCGTTTGAATGCGTTTTTTTTTTGTCCGGATTCACAAAGTAACTTTGATCAATTAAAGTTTTCA	A	T	ATACTAACTTAATAATGTAAAACAACAATATATCTACATATATAACAAAATTCAAGTGTCTGTTTCAGATATGTAACGTTGACGATTTCAGAGTCATTGG	NA	NA
Ca-II-SNP57	53.37	Ca-*desi*-LG(Chr)1	2289645	TTAACTATTTTTTTTCTTTCATTGGATCCTTTGATTAATACTACATGTACCACTTTCAATTCAATATTTTTTACTTTGCATTAATAATAATGATGAATTT	T	G	TGATTGTCTCAATATTTAATCTTTTAATATAGATTAACAAAAATGTATAAAATAAAATGGAGAATAATAATGTTGCGAAAATATCATTATCAAGTATTGA	NA	NA
Ca-II-SNP58	54.27	Ca-*desi*-LG(Chr)1	2293371	GGTTTGTGTGTGTTTGGGAGTACTTGAAATGATGAAAATTTTGATGAGGGGGTGTGAAATGCAATGAGAGGAGGGTATGATCGTGAGGGTTTAGAGATAA	G	A	ATTGCTTTCTTGCTGTTTGAATTGGTGACAAACCATTCTGTAGCTGTTTCTCCAAAGATCCATAACTACTAGAACGAATACCCAATGACCCACCACTCAT	NA	NA
Ca-II-SNP59	55.17	Ca-*desi*-LG(Chr)1	2304660	ATTTTATGCACATATTTAGGGTTGAATTTCTCTGATGGAAGAGGAAGTTTTCCATTGCACCATCTATAACTGTTCGGAAACCTTGAAACTTGGAAGTGGT	A	T	CACACATGCAAAGGGATATAATATATTCATCTAAAATTTTAAGATATTTGAAATATGAGACTTCTCTCTTGTACCATAGCCAATGCGGTATTCTATTTTT	NA	NA
Ca-II-SNP60	56.97	Ca-*desi*-LG(Chr)1	2317643	ATTTTTTTTAAATGGTTTTCTAGTTTTATTTGATCAAAACGTCGCAATCCCTTTAGATTTTTAGTTCTTTTTGGTTCTTGCCTCATGAGTTTGGATATAT	C	T	GACTAGTGAGTACTTTCTCTAATCATTTTTCAATAAGAAAAGCAAATGAAAACAACAAAATTTGAAGATAGTCATAAACTAAGCTGATAATATTTTGAAA	NA	NA
Ca-II-SNP61	57.87	Ca-*desi*-LG(Chr)1	2325015	CGATTAAGCTCACAATGAGATTTCCTGCTGTGTTGTCTTTGAGAAACAGGAGTACTGTTTTGGGCCAGATATGGAGTGAGAAACTTGTTGGTGAATCTGG	C	A	AAGATTTTCTTACTCTGTCAATTTCAGTATACTTGTATTGTAAACCAGGGAAACCTCTTGATAACTTCCAGTTACCCTCAAATCCAACACTACCAAAGTA	NA	NA
Ca-II-SNP62	58.77	Ca-*desi*-LG(Chr)1	2340803	AAGGAGATTGAAATTTTTTTAAATAATTTTTATGTTATTTTAATTCTTGTGTAGTACACCTAGGGTGTGTGTGTGTGAGTTTGATAGAAAACTTTGTGCT	A	G	ACATATATTGGATTTGAATCATTTATCAAAATCATCATCCACTCAGCTTTTTTGTGCACCCAATTTAAGAGAATTTTGTATTATTATGAATAAAAATAAA	NA	NA

Marker IDs	Genetic positions (cM)	Chromosomes	Physical positions (bp)	Flanking sequences/Forward primers	ICC 12968	ICC4958	Flanking sequences/Reverse primers	Annealing temperature (0C)	Amplified Product size (bp)
Ca-II-SSR46	59.67	Ca-*desi*-LG(Chr)1	2351367	GTTGCAGTTACTGGGCTTCA	(TTA)6	(TTA)5	AAGCATCAAGATGGCAAACC	58.93	136.00
Ca-II-SNP63	60.57	Ca-*desi*-LG(Chr)1	2358985	GACATGTGTGGACTTTGATGGCCTACACATGCTTTTTGGTGCCGCTTTGTTTTTAGTTCTTTCTTTTTTGAAATTTAAGGAGTTATTTACTTTGTAAAAA	C	T	TGTTTCTAAAAATGTCGACAAATTATTCACTTAAAAAATATATTTCTTTTTTATTAACGAATAAAATTGACATAAATTTTTAAATATGAAAATATGAAAT	NA	NA
Ca-II-SNP64	61.47	Ca-*desi*-LG(Chr)1	2363519	CTGGGACTTGGTGGAATTTTTCGTAGACTGGACCGACGACGGTCTTAACAGCTTCTTCGACGGTGTCGACGCCGGGTTTGAGTGGACCCGAACGTTCTTT	A	G	AGGAAGAAACTAACCAAGAGCAAGAACTCAAGTACCTTGAATTCGTGCAATTCGCAACAATTCAAGCTGTGATGCGTTGCGCGATTCTCTATTCCTACGC	NA	NA
Ca-II-SNP65	62.37	Ca-*desi*-LG(Chr)1	2381955	CATTGGGAAATTTTGAGTCGAAATAATAATCCTTGTGTTGTGGTGTGATTATGCTTGCATGTAAGTTTGGTATCATATTGACTATTAAACAAAGGGTCCA	C	T	GTAGGAATCAAATTCATTATCTTGAAGTTATAACACTCACACACCGTTCATCAGTTACTTATGCTAAGGCTTATGTTAGTCCTAATTTTTGGTTTCAGCA	NA	NA
Ca-II-SNP66	63.27	Ca-*desi*-LG(Chr)1	2392790	TGTTCCAAAACGCGGGTGAATCGGAACAAAGCGAGGTAGAAATGGGAGATGTTGAGCGTCTGATGCAGAAGTCGTGAGTTGGTGGCACACAAATTCTTCG	C	T	TGCAACCAAATGAGTATACTACTTGCTATATACTAACAAAATACTCGTGCGTATGCACAAGTCGTGCAACCTTAGTTACCATATCTAGAAGCTTAGTACG	NA	NA
Ca-II-SNP67	64.17	Ca-*desi*-LG(Chr)1	2407032	ATTTTAGAGTACCTTTGTTAATTAGTTTTAAAGTATTCCCTAGGATATTTTAGGTTGAAATTATGTTGGGTTATGTTGTAGCGTTCTATTAGAAAATCTT	G	A	GAGCATAAAATCTAAGATATAATTCTATTCTAGTTAGCTCATGATTTGTGTACTCCTTTCGTACTTTGCAGCAATGTGTTAGAATGTTACACCATTTCAT	NA	NA
Ca-II-SNP68	65.97	Ca-*desi*-LG(Chr)1	2422961	TACGCGGGCAAGTGCTGATGATGCTGTTACTGCTTCTAATGATGTGAATACTTCTGGAAATAGTCATCTGGACCAGCCACTGAAATTAGTGGACGACGTT	C	T	GAAGCTAGTGGAAAAATTTATCATGAAGAATCAGACCTCTCTTTTCTTTTACCAGTCAAGGAAGAACTATTCTTCTCAATGACTTGGGAATCTAAGCTGC	NA	NA
Ca-II-SSR47	66.87	Ca-*desi*-LG(Chr)1	2424978	GAGGATTTTGATGGCGAAGA	(GAA)5	(AGA)8	GTCTGAGACGAATCCGGAAA	60.16	214.00

Marker IDs	Genetic positions (cM)	Chromosomes	Physical positions (bp)	Flanking sequences/Forward primers	ICC 12968	ICC4958	Flanking sequences/Reverse primers	Annealing temperature (0C)	Amplified Product size (bp)
Ca-II-SNP69	67.77	Ca-*desi*-LG(Chr)1	2435676	CCTTTTATGTGGCTTTCTTGTGGGACACTTCTTGTTATATTGTTAAACATTAACTTTAGTTTTTAGCTTTTCAGTTTATTGTAGTGCTTTAATGCAACAC	A	G	TTGATCCTTCAAGTAGCCTACACTTTATCCTGTCTAGACTCTGCAATCATACAATACATAGTTGTTTTAAGCCCTGATAGCAGCAGCAAAAAGCCACTTC	NA	NA
Ca-II-SNP70	68.67	Ca-*desi*-LG(Chr)1	2455899	CTCCTTACAAATCTTTCCAATTTCCTCCATAGGTTGAAAAACTCCAATCCCGTAGTTCACAACAATAACATCGTGTTTGATGAGGGATGCTACTTGGCAA	C	A	CTAATCATAGTTGTCTTGCATATTAAGCACAGTCTCTTCAATTTAATTCGTTTATTTATTTACTTATTTATGGCTTGCAACAACGCCGTCGAACTCGCTC	NA	NA
Ca-II-SNP71	69.57	Ca-*desi*-LG(Chr)1	2464479	CGCTTATATTTGGTTCGGATAATGGGTTTGGCAGTCTCAAAAGTCCAATAAAGTTTCATTATTCATCTTCTTTACATTTTTAGCTTCTTGAAGCCGCGCC	G	A	CTAGAATTTTAAATACAATACACAATCTCAGTAGCACACCAAATGGACTGCTGAGAAGCTGAATTTTAAATACAATACATAATCTCATTAGCACACTAAA	NA	NA
Ca-II-SNP72	71.37	Ca-*desi*-LG(Chr)1	2472975	CTTAGTTTTGAATTGTGTGTTCTAGTGGTGATCATTGCCCTTTCACCCTTTAGTGAAGTATGTTGCTCTTTAGATGTGAGGAAGGAGAATTCATGAAGAG	G	T	GCAAAGAAATCTAATAAATTTGTTGCTTTCTAATCACTGTAATCACGTACTAGTATATCCTATAAAAGTCTACATATTATTTCTCCTAAGTTAAGAAGAG	NA	NA
Ca-II-SNP73	72.27	Ca-*desi*-LG(Chr)1	2476682	GCCTAGTTTGAAAAATCTTCCCTTTTGATTTGTGTAGGTCTTTATCTCCTTTTCAAATTTCTTCCACTTTTAATTTAATATGTTTAGTTTACAAATCATA	T	C	GTCAAAAGAATAAAATAACTTTTTGAACTACAATATAATAATTATCAAAATATGTGTGACTATGAAATACTCGGTTCAAATGATGAAGAGATGGATGGGG	NA	NA
Ca-II-SNP74	74.07	Ca-*desi*-LG(Chr)1	2487961	AATTTGTTGGGTTTTATTCGTCCATGAGGTGTCAGAGCTTTTGATCTTGTATGCTCAAGGGACGGAATTCAAATCCCTTCGTAGATGATTGTAAAACGAC	C	T	AAAAAAAAACAAGTTCAATTCAATGGTTAATCACGAGTAAACCTAACAATTTTTCTTTTAAATAGGAGAAGGAGGAATTGTTAATTAAAGTGACACTAAT	NA	NA
Ca-II-SNP75	74.97	Ca-*desi*-LG(Chr)1	2495031	TAAGTGCCACGGAAAAACATACTTACCTGGATGGGGTCTATGGATGATCAATAAGGTCCATGTCCTAAGTTGGTGACCTCCATTGCACTTTGGAGGGGTG	T	C	TATAAATTTTGAACTGAATAGGAAGGGGGCCGCGCGAACGCTGGCCCCCACTGCAACAAATTATGATGTAGGCTGTTCTACCTGAGAACACCTTAAGCGG	NA	NA
Ca-II-SNP76	75.87	Ca-*desi*-LG(Chr)1	2506596	TTAAGTATAGAATCCAATTTTTGGTAGAGTGATGCTGCCATTCTTATAGCTTCTAGCTTTTTGGAGGGGAAACCTTCAAACCGTGATAAAACCTACACAG	A	G	ATACAATACATAGCATTTATCATATAAATTAATCAACATTATTTTCAATAATTAAATACCATAACATAGACATATAAATAAATATTCATTTATGTATGTT	NA	NA

Marker IDs	Genetic positions (cM)	Chromosomes	Physical positions (bp)	Flanking sequences/Forward primers	ICC 12968	ICC4958	Flanking sequences/Reverse primers	Annealing temperature (0C)	Amplified Product size (bp)
Ca-II-SNP77	77.67	Ca-*desi*-LG(Chr)1	2547484	TACGACATGAAATTTCAAATTTTATGACTACGACTTTTTATTTCTCCTATCCATTGCATTCACATGCCATATTTTCTCTTATTTTCTATTTATTTTTAGT	A	C	AAAATTTGTAACAAAAAACTTCACTATTGAAGGTTGGGATATACCATTGGTGGCACGACATGCATCGGAGGATACTCACGGAGGTGCATTGGTGGTGTGC	NA	NA
Ca-II-SNP78	78.57	Ca-*desi*-LG(Chr)1	2577406	TCTCAACTTTGTTCTCTCTATCGAATTTCACAAAACAGTGTGGCTTTTCACCTTCAAAATTGACCAAGCTAGGTTTCTTCCATTTGGGTGTTTCCGACAA	C	A	ACGATTGTGAACAATCAAGATCTAGCATTTCACAAGTGAAATCATCAGAAAACAGAGCACCTCCTACTCTTCCTTTGGAAGAAGAAACAGTGAAAGAAGT	NA	NA
Ca-II-SSR48	80.30	Ca-*desi*-LG(Chr)1	2590753	ATAGTGGACCCCACAATCCA	(CT)9	(CT)7	TCGCTGTTGTGTTTTTGCTT	60.05	144.00
Ca-II-SNP79	81.13	Ca-*desi*-LG(Chr)1	2597468	GGCAATGTCATGTTATAAAGGAGGGTTATTTGTTGTTGTTGGGCAGCAAGTATAAATCAACAACAATGTGTCACTCTTCAAAAGATATTGGTCATTTTTT	A	T	TTCGAGACATAGCATTTCTGTCCAAACAACCAATATCTTAAACAAACCCAAAAGGCAAAAACAACCAATAATTTTTGGGGTTACAAACCAATATATCCGT	NA	NA
Ca-II-SNP80	81.96	Ca-*desi*-LG(Chr)1	2615225	ATATATGTAGTTTTGAATTGTATAAAGCCACTGAAAAGTGAATATTTTTATATTCTATTATTGCAGTTGCTGTTGTGCGAGGAAGGACCCGTTCTCTGGT	T	G	CTTCATTACAAATTTTTCCACCTGTTTCATTTATTGTGGGTAGGGATGGACTAATTTGATCTTCGCTGATTTGAACTCTTTCCCTTCTTTCAAACCTCTC	NA	NA
Ca-II-SNP81	82.79	Ca-*desi*-LG(Chr)1	2626185	AGGATGTTAGATATCTTAGTCGGTTAATTAGTTAAGTTAGTTAGTTAATTACACTAGTCAGTTAGTTATTTTACACAAGTTAGTTGTTTATCTAATTGAC	A	T	TTGAGTTAAGGTGTTGATAAAAGAGAAATAGAGATAAGTAAATTGAATGATATTGATAATTGAGAGAGTTAATTACATCAAGGGTTACAAACACTTATAT	NA	NA
Ca-II-SNP82	83.62	Ca-*desi*-LG(Chr)1	2639056	CAAGGTATGAATAATGCTTTTGTTTCTCTCCCTCTGCTGTTTTTTTTTTGAAAAACATATTATTTGTATAAGAAAGAATCAATATAACCTCGTGCTTTTG	C	T	TAGCTCTAATTGTAATTCTATGATGTATCTCTGTTGAATTTGGGTCAAGAGCTGGTTGTGATATCTCCAAACCTTCTTCCTTCACAATTTTAACGTATCT	NA	NA
Ca-II-SNP83	86.94	Ca-*desi*-LG(Chr)1	2752133	TGACTTCTGTGTGGACGCCTGTGTGGCTGGTTATATCCGGATCATATATGTTTAGGAGCATGCATGACTTGCATACATTTTTATGTTATTTTATTTTGAT	T	C	ATTTTAAAAAAATTAGATTCTTTAATAGTTCTTAAAGATGTATCAAAAGTCATAGAATGTATCTAAATAAATCAAGTAGCAATCGTATAGCAAATAATTA	NA	NA

Marker IDs	Genetic positions (cM)	Chromosomes	Physical positions (bp)	Flanking sequences/Forward primers	ICC 12968	ICC4958	Flanking sequences/Reverse primers	Annealing temperature (0C)	Amplified Product size (bp)
Ca-II-SNP84	89.43	Ca-*desi*-LG(Chr)1	2808533	TTATATTTTTTGAATCTGTTATTATAATACTTTGTATATAGTTTTGGATTTCAATTATGGATAAATTAGGAGATGGGATTTTTCCAGATGAGGTTGTTAT	G	T	ATAATTGAATGAAATAATTATCCAAACTCAACCTATTCCACAATTTGCACACAGTTTTGGTCCTAAAAAGGGATTTCACTGGTAACCTAGCAAGAATTTG	NA	NA
Ca-II-SNP85	90.26	Ca-*desi*-LG(Chr)1	2843332	TCCATCCTAAAAAAAACTAGTGACTTAACTCATTCTTTTACTCTCCTTTATGACAAGATTTTATGACAATTCATGGTTTTTCTTGGCATAATGTGCATTA	A	G	AAAAAGGAGAATAGTCATCTCTTAATTGCACAAAAAAAGAAACTCATAATTAAATTTATAATCTTCCTTTAAAAAATAAATTAAAGGTGTTAAAAATGAG	NA	NA
Ca-II-SNP86	91.09	Ca-*desi*-LG(Chr)1	2865546	AATGATATCAATTTTCTTCTTGCATCTTATGTAATTCCTTATGTTGGTCTTACTGGCTATGTTGGTGCCAATCCACTCTTGCAAAATGCTACTTCAAGGA	G	A	CAATAAGATCATAAGATCACAAGTACATGTCTACTTTTTGATAAATACTTTTTCTTTTAGTTTGAAAAGTTTTCAAAGATTGAGCTTGAGATTATATACC	NA	NA
Ca-II-SNP87	91.92	Ca-*desi*-LG(Chr)1	2898518	TGCGTCCATAATTGGTTGGAGCCCAAAGTTGTGTGGTGCGTCCATAATTGGTTGGAGCACACAGATCCTTGCGGGAATTTTGAGTTAGTGGTGTTGCTTG	A	G	AAGATACAAATCAACCAAAATGCACAATCTATTTGTTAGACTATTTGTCAATGCATGATTCCGGAATATCACATCCTCCGCAAGGGTTTCGTGGGTCCTT	NA	NA
Ca-II-SNP88	92.75	Ca-*desi*-LG(Chr)1	2927971	AGCGCTGTAATATGCTTCTCATTGCGAACCATACGAACAATATTGTCTTGTTACTAACCCTCATTACGTAAACTTCATACGCTTGTTTCCTCTTCATTCT	T	C	TTACGGCGCAGAAATACGAACAAAGAAGAACAACGCATAAATACGAATGGTTCGTAGGAGAGTAGGGTTCGCAGAAACAAAGATGGTTCGCAATGACAAG	NA	NA
Ca-II-SNP89	95.24	Ca-*desi*-LG(Chr)1	2958413	TTTGTTGAATCGTTGAATGTTGTGGTGGCCTTAGACATTTATTGATTGCTTTAGTTCATTATTGTGTTTTCTCCTTGAAGTGCTGTGTGGAAGGAGCTTT	T	G	GTAGAGGAATTATATTGCAGAAGATAGTGAGGGTGCGAATAAGAATAGATAGCATGAGGAACACACACCAATCTATTATCCTATAATCAAAACTCAAAAT	NA	NA
Ca-II-SSR49	96.07	Ca-*desi*-LG(Chr)1	2973501	GTTCCGTTCTCCACCTTCAA	(TAT)8	(TAT)6	TTTTGCGAGAGGTGTGTGAG	60.09	146.00
Ca-II-SNP90	98.56	Ca-*desi*-LG(Chr)1	3109215	TCCATCCTAAAAAAAACTAGTGACTTAACTCATTCTTTTACTCTCCTTTATGACAAGATTTTATGACAATTCATGGTTTTTCTTGGCATAATGTGCATTA	A	G	GAGAAATAATAGTAAGTGAGAATCTCTACTCTTTGCATGCTCGATCACCCGAAAATTACACAACACTCCCCCTTCATTCATTAATTACCAAGCTTTTCCCTTACTATATGGTTCATACCCCACTTCTTTTTCTTTTTCCTTTTTCT	NA	NA

Marker IDs	Genetic positions (cM)	Chromosomes	Physical positions (bp)	Flanking sequences/Forward primers	ICC 12968	ICC4958	Flanking sequences/Reverse primers	Annealing temperature (0C)	Amplified Product size (bp)
Ca-II-SNP91	99.39	Ca-*desi*-LG(Chr)1	3141981	AATGATATCAATTTTCTTCTTGCATCTTATGTAATTCCTTATGTTGGTCTTACTGGCTATGTTGGTGCCAATCCACTCTTGCAAAATGCTACTTCAAGGA	T	C	TGTACATGAGAATTTGTTTAAGCACAAAAACAAAAGAGACACTCTCTCTTACATAGAAAACATCAAACCAATTAAAACCACACCCTTCTCCTCCATCAACCAAAACAAAACAAAACACCTCCATTTTCGTTTTTTCATTCATCGATGGCGGACGTGCTTTCTCAAAAGGCAAAAACACCATCAAAGAAAGAAACATCGAGTCTTTTACTTGGTCGATTCGAAATTGGGAAGC[illegible]	NA	NA
Ca-II-SSR50	101.05	Ca-*desi*-LG(Chr)1	3165102	CTGTTTTCCTCGTTGCACCT	(TA)12	(TA)11	TCCATAAAAGAACCAAGCATATCA	60.29	134.00
Ca-II-SNP92	101.88	Ca-*desi*-LG(Chr)1	3192156	TAGGATAAGAGGCCATGTAATTGATTGCACAAACTCCATATTTTAAATTAGTATTCCTTCTTATATAAATGTACCCTTCTATTCCCCAATTTGTTCCCCA	A	G	ATGGAGATTGCTCAAGTGATCCAGATGATATTGATCATGCAGTTTTAATAGTAGGTTATGGATCAAAAGGTGATGAAGATTACTGGATTGTAAAGAATTC	NA	NA
Ca-II-SNP93	103.54	Ca-*desi*-LG(Chr)1	3270298	TGCGTAGCATCTCCCTTCACCTTTCAATCCTTAATAAGTATAGTCTTACTACTGTTACGAACCATTAGACTGACCGCTTTAAAATGTATATTACTCCCTC	A	T	GTTTAATTATTGAGAGTATTATTGAAAAAAAATTAGATATTCTAAAGTGAAAATTATTTGAGATGGAGAAATATTCCAAACATGACATTTATAATGATAC	NA	NA
Ca-II-SNP94	105.20	Ca-*desi*-LG(Chr)1	3329312	ATCCTAGATTAGTAATTCCACTTAATGTTCACAATTCATGATAACTGTAGGGCCTAAAATGTGCTCATGCAATCATTTCATTTTACTATAATTCGTTCTG	T	C	TTTATGAATTTGTGGTGAAAAGTAATATGATGACCCGCACAAATGTCCCCAATAACACACATGTCTCCATCTACTCAATGCAAGGAAATAAAAAATGCAA	NA	NA
Ca-II-SNP95	107.69	Ca-*desi*-LG(Chr)1	3635583	TCACAGCAGGACTAGTTATAGCATTTAGTGCTAATTGGATCCTATCTTTTGTGATTCTAGCTGTGTCACCCTTGTTGCTCATACAAGGATACATTCAAAC	A	C	AAAAAAACAATATATAAGTATACTTTCTATTGATGGAATCGACGAAAAAATTGTTCATTAGGCCAACCTTTGCATCAGAACTGAATCCTTTGAGAAACTT	NA	NA
Ca-II-SNP96	110.18	Ca-*desi*-LG(Chr)1	3654198	ATCCTAGATTAGTAATTCCACTTAATGTTCACAATTCATGATAACTGTAGGGCCTAAAATGTGCTCATGCAATCATTTCATTTTACTATAATTCGTTCTG	C	T	GGAAGACAAAACTACGACAACATCAAAGAACTAGAGTCTTCTGTTAAAAACAGAGGAAATTCTTAGACCCAAAAGATAGTGGCATTAAAACTAAATGGCTAAGTTTATTTCAAGTTATGGTACTCAAAATTTCGTTCATGGGAGTGTTCTTTTCTGGGTTTTTATTTTATTATCACATATTTCCTTCCAACTTGCAACATGTGGCTCCGTAGTAAAGTTTCTTCCTGG	NA	NA
Ca-II-SNP97	111.01	Ca-*desi*-LG(Chr)1	3693177	AGTTCGTCCTCCAGCGTGTAATTATTTAACTTCCAGTTTTTTGTGGGTTCACCGTTGGAGTTGTGTCAAGATGTCATCACGGTATTGTCAGGAAGGAACG	G	A	GTTAACGGCTATAAATTAATAAAATATGATAAATTTATTATTATTAATAAATTAATAAAAGGTAGCGGTTATAATTTTATTATTATTAAATTGTGAAGCA	NA	NA

Marker IDs	Genetic positions (cM)	Chromosomes	Physical positions (bp)	Flanking sequences/Forward primers	ICC 12968	ICC4958	Flanking sequences/Reverse primers	Annealing temperature (0C)	Amplified Product size (bp)
Ca-II-SNP98	111.84	Ca-*desi*-LG(Chr)1	3734425	TTTTTTTGGTTTGTATTATTATTAATTAACTATAAG ACAAGTCTCCTTACATTCTCTTTCACTCAACTTATT TTCTTCCTTCCTACATCTCCTTAGCTTC	T	C	ACATAAACAAAAAACAAAACAGTACCTTTTTTTT TTTTGTATGTTTCATATGTAACTGTTTCAATTAA TTCCAATGCCTAACGTTACCCTTTTGAATGAAT GAAGTATAGAAAGTGGAAAGTTAGAAGCTTTAA TTGTTATTGAAGGTGAAAGGAAGAGTGGTGGTT GGATTTGAATCAAACCCAGATGGATTTTTCTTC TTTCTTAACCTCTCTTGGTACTTCCTTTTTGATA [illegible]	NA	NA
Ca-II-SNP99	112.67	Ca-*desi*-LG(Chr)1	3754453	TGCAGAAGCAGAAGCTGACAAAAACATTGTTGGA ATCCATGCTGCAATGGGAAGTGCAACTGGCATGA ATATCTTCCTTCGCAGTTTCCCTTCAAGATGC	G	A	TGGGAGACCTAGAAAAAATGGGAAGAGAACTC AAATGTCCAATCTGTTGGAGTTTACTACATTCT GCGGTTTCACTCACATGCAATCATCTTTTCTGC AACTCGTGTATAATTAAGTCCATGAAATCTTCAT CTTCTTGTCCTGTTTGTAAAATACCCTTCACTC GTCGAGAGGTTCGTCCTGCTCCTCACATGGAC AACTTGGTTACCATCTACAAAAACATGGAAGCT [illegible]	NA	NA
Ca-II-SNP100	113.50	Ca-*desi*-LG(Chr)1	3867644	AGAACTGTTGAGGACTGATCCTTACAGAGTGGAA GACATGGATATGTACTCCAATGTGCTATATGCTAA GGAATGCTTTTCGGCTTTGAGTTACCTTGCA	C	T	AAATATACAACTGACTTTTCATGTTGCCCCTTCA AACTGTAATAATTCCCAATAATACAACAAGATTC AGGTCTGTATTTATCAGTCATGAATACTCTAT	NA	NA
Ca-II-SNP101	114.33	Ca-*desi*-LG(Chr)1	3888320	TTTTTTTGGTTTGTATTATTATTAATTAACTATAAG ACAAGTCTCCTTACATTCTCTTTCACTCAACTTATT TTCTTCCTTCCTACATCTCCTTAGCTTC	T	C	GATCAAATAATCCCAAACTGAAATGCTAGTTAT TTGCTTCAGTTTTTGGTATAAGTTACAAACAAAT CAGTAGAAGAAAGTGAATTTAATCATAGCAATA	NA	NA
Ca-II-SNP102	115.16	Ca-*desi*-LG(Chr)1	4001436	TGCAGAAGCAGAAGCTGACAAAAACATTGTTGGA ATCCATGCTGCAATGGGAAGTGCAACTGGCATGA ATATCTTCCTTCGCAGTTTCCCTTCAAGATGC	T	A	TGCAAGAAGGATGAGTAAAGTGCACAAAAAGG CTTAAGACCTTCACAAGCCAGACCTGCAGCAAA TGTAACAGCATGCTGTTCTGCTATCCCGACATC AA	NA	NA
Ca-II-SNP103	115.99	Ca-*desi*-LG(Chr)1	4998121	AATCATGTTCCTCTTTGGTTGGATATTTGGAGCAT TCTTCTAAAATTATGTTTTCTATCATACTTCTCAAT TATGTGATTTACTTTCTCAAAATAAGGAA	G	A	CACTTAACAAGAGTCACTCGTAGAGAAAAAATT GCATAGTTGCACTCCTTCCAAATTTCCCAAAAA GAAGCTAATCAAATCACTTGCAAGTAATTCTTG A	NA	NA
Ca-II-SNP104	116.82	Ca-*desi*-LG(Chr)1	5016574	ATATCCCTTCTTAGATCCTTTCTTTGTAATATTTCT TTCAAACCTTGCCACCTTCCTGTCTGCTACGCGC CTTGATGTTGTCTATAATTAAATCCAACAT	G	C	TATCTAATTAAAAATTAAGATGAAAAATTAAGAT GAAAGACATATATATTCAAATATATTTTTATTTAT ATTTTGGTGATTTTTGTTGTGTTTGACGTAA	NA	NA
Ca-II-SNP105	117.65	Ca-*desi*-LG(Chr)1	5041011	CTAACTAATTGTATCTCTTAGTTTGAGAATTGTGAA TCTGTAAGTACTGTGTTTTGGAGTGTTATACTGCA ATACTAAATGGAACTTCATTTACTGTGTA	G	T	ACTACACACTCAAATAGTGGAATTAAACCTCTT GCAAATGCAAGGTAACACAAAATACAATATATA GATTCAACCTTTCCATATGATGCATGGTTATCA A	NA	NA

Marker IDs	Genetic positions (cM)	Chromosomes	Physical positions (bp)	Flanking sequences/Forward primers	ICC 12968	ICC4958	Flanking sequences/Reverse primers	Annealing temperature (0C)	Amplified Product size (bp)
Ca-II-SNP106	118.48	Ca-*desi*-LG(Chr)1	5101106	ATCATATTGTTTTTACTATTTTCTTACATATTTCTTA<br>GCTGTTATTCTCACCATTCTTTAGTACTTTAATCAA<br>TATAGAGCCTTTTAATGGGACCCTTGT	T	C	ATAGACTTTGATCAATAGCATGCCAAAGCAACA<br>TTTTAAATAACACCTCCATTTTTGGGTGCGCAT<br>GTGAAACCCCAAGGGAAATACTGGTGATTCTTT<br>T	NA	NA
Ca-II-SNP107	119.31	Ca-*desi*-LG(Chr)1	5117310	ATTTGGAAACAAGTCTAGCTTTGAATTTATCAATG<br>CTAGCATCAACTTTCATTTTCTTCTTAAAGATCCAT<br>TTACAACTTATTGGTTTGGATCCAGGTGG	G	A	AAGAGGAAATAAAGTCTCAAGACTTTTCCTTTT<br>GGAAAGAAGTCATCCAAGAGGAAATGGACTCA<br>ATTATGGGGAATAAAACTTGGAAATTAGTAGAC<br>CT	NA	NA
Ca-II-SNP108	120.14	Ca-*desi*-LG(Chr)1	5178186	TGCTTTGTCAAGGAAGGGGAATACATATGAGAATC<br>CTCTGGCTGCAAATGAAGAACACCAAACCACTCCT<br>TCTCACCCACCAGGCTTTACCCCGACTACC	C	G	TGTGATTGGTTTGGGTTTTGGGGGTTGACGAC<br>TAAAGGTGGAGTATAACTGGGTGGAAGACCAT<br>ACATAGGAAATTGCATAGTTGTTGTATGAGGTT<br>GTT	NA	NA
Ca-II-SNP109	120.97	Ca-*desi*-LG(Chr)1	5217919	TCTTAACTTCTAACTCAACTGATAAATGTCGATACT<br>GTTAAGTTAGATGTCTTGTTTCAGATTCGAATCCG<br>AGACCTCGCAGTTGTGTGAATTAATTTTG	A	C	TGGAACAGCAAACAAAAAGAAAAAAATAATCTT<br>ATTTAAATAATAACTAAGGCTTGTTTGTATAAAT<br>TATATATATATTTAGAAGAGATGATAAATTCCA	NA	NA
Ca-II-SNP110	121.80	Ca-*desi*-LG(Chr)1	5260586	TGGTTAGTAAGATAACCATTGTAACAACCGTTTAT<br>TATAGGCAGCATTGGTCATGTCCTAATCCTATCAT<br>TTGGCAAATGGTTTTGTCAGGTAAACCGGT	C	T	CTTCTTCTTAGTTCTTACTTACAAACTATTCTTT<br>TCACTCTTCACATTTTTCCCATCTCTTTTCACTC<br>ACTCACCAAATCTATGGACCCCACATCCTCCTA<br>TCACCGCCGTTTTCCACCCTCCTCCGACCGCT<br>TACTCGCTGCTTTCAATTCCTCTCCTTCCTCCA<br>CCGCTGCCACCACCTCCTTCGCCGAAGAGCTC<br>AACGAAGCCGAACTCTTTTGGTCATCCGATAGC<br>[illegible]	NA	NA
Ca-II-SNP111	122.63	Ca-*desi*-LG(Chr)1	5382171	CACTGAGTTTCATTGTGAAATGGAAGGGAATTGA<br>GTGATTTTCTCAGAGACTCCGCTGTACTTGCATTC<br>CCATCAAGCGGAGCTGCTCTGAGGTACATGT	T	G	TTTTTTGTGAATTACAGACCAGCATGTTTCATTT<br>CTTGTTATGTGGATGATGCAGTTGCTCTCCTGA<br>CTGAGCGTTTGCTTTGGACCGCTCTCTCCCAA<br>CACCACTCATGTTTTTCAGGCAAACCTCCATCA<br>ATTCAAACAAAAGTAAAGCTACCACTAGAGATA<br>TGCCT	NA	NA
Ca-II-SNP112	123.46	Ca-*desi*-LG(Chr)1	5667198	GAGACAGTGGGTCCACCGGGTAGTTGACGATTCG<br>GGTTGTCTCTCAATTGGCTCTAGATACCATGTTGA<br>ATATAAAGAGTATTGAGAGACATTGAATAAC	T	C	GGGCTGTAAGTATTCCGGCCCGTTAGTATTACT<br>GTAAATTAGGGTTGCTTAATACTCTTTGTCTATA<br>TAAAGAAATTCCGTTGTGTAGTTGAAACACACG	NA	NA
Ca-II-SNP113	124.29	Ca-*desi*-LG(Chr)1	5701969	TATTGTATGGATTTGCTACTATTTTGAAGTTTTTTA<br>GTTGTTTTTATTGTTTTGGAGACTGTTCTGGAGGT<br>GTTTAATTGTTTTTAGGTGTGATTTGCTC	G	A	AGGACCAGGGAAGATTCACAAGATCATGAATC<br>GTGAGATCGTAAAATCCTACCAAATTTGGGGTC<br>ATTAGATTCATAAAACAACTAAAACACTGAGAA<br>CA	NA	NA

Marker IDs	Genetic positions (cM)	Chromosomes	Physical positions (bp)	Flanking sequences/Forward primers	ICC 12968	ICC4958	Flanking sequences/Reverse primers	Annealing temperature (0C)	Amplified Product size (bp)
Ca-II-SNP114	125.12	Ca-*desi*-LG(Chr)1	5732484	AATCCTTTGTGACCATAATGACGAGTTCATATGTGGTTTCTACTGCAATGTGGCAATCTGTAGCTCCTTATTTTCTGAAATGTGGACTTTTTGCAGTGGT	A	G	AAAGTAAACTGAGTATAACATTTCTTGATCATGGAAGGGAGGGTTAGAGAGCCAGTTTAAAAAATAACATTACAAATCCCTTTATCACGAGCCAATAAAA	NA	NA
Ca-II-SSR51	125.95	Ca-*desi*-LG(Chr)1	5754943	CCTCAATGAAGGGAGTTCAAA	(TAA)11	(TAA)10	TTGTCTGGTCAGCTTTCTGC	59.16	186.00
Ca-II-SNP115	126.78	Ca-*desi*-LG(Chr)1	5759582	GGATCTGGAAGTGGCAGTGATGGAATATATTAGATATATCCAAATTGTCTCAAACACCTCTATGGTCGATATTTCACTACCGTGCTGCATAATCGAAGGT	A	G	ACATATTATTGATGCATTGACACTTGCGAATGCTATATGGAGACCATGGGAGAATCATAGGGGTGTCATTCCTTTTGATGATATCATGCTTTATACTGGT	NA	NA
Ca-II-SNP116	127.61	Ca-*desi*-LG(Chr)1	5815163	ATCAGACAGAGAAGGAAAACAAGGAAATATTTTTTTTTACAAAGTTTTCTCGTGTTGTTGATTCCTAAGCGCAGTGCTTCTTCCCCCATGCCGCCTATTG	A	G	GACACAGTGAAACATGATTACGACTAGTTCGTTGTAAAACGACATTTTAATCTATCAAATGAATATTTACTGCTGCTTGAAAAGTTATTTACCACTAGTA	NA	NA
Ca-II-SNP117	129.27	Ca-*desi*-LG(Chr)1	5854373	TGGTTAGTAAGATAACCATTGTAACAACCGTTTATTATAGGCAGCATTGGTCATGTCCTAATCCTATCATTTGGCAAATGGTTTTGTCAGGTAAACCGGT	T	C	GTTTGATATTACATTACAATTTATTTACACAATGCAAAGATAAATAAAAAGCTGAAAAACAGCTGTAGTTAAACAAAGTAGCAGCAGAACATGATAATCA	NA	NA
Ca-II-SNP118	130.10	Ca-*desi*-LG(Chr)1	5898347	CACTGAGTTTCATTGTGAAATGGAAGGGAATTGAGTGATTTTCTCAGAGACTCCGCTGTACTTGCATTCCCATCAAGCGGAGCTGCTCTGAGGTACATGT	A	T	TGCAATAAATAAATGCCAAGCACCAACAAGAACAAAAAGACATAATACCTCGGAGGCCGAATAACGAAATTGATGAATTGATCAATCATATTCTTGCTTC	NA	NA
Ca-II-SNP119	130.93	Ca-*desi*-LG(Chr)1	5970900	ATAAGAAGATACATGCCTTTTCAGATACCGATGAAGACAGAGACGGTGGTTCGCGGTGGAAGTTGAAACGGCGGTGATGCTTTCTGTTTCTTCTTCTTCT	T	C	AAAAACTCTATAAATACATCCTTTTTTTACAAAAGGGGGAGACATTTTTGAGGGGGAGAAATCGGAGATTAATACTGATAAGAAACACATAAAAAAGAAA	NA	NA
Ca-II-SNP120	132.59	Ca-*desi*-LG(Chr)1	6029621	TTTTTTTCCCTCTCCATGTCTTGACCGCCTTGGTGCCTCGTCTTCGCTTTCACTATCTTCATTCCTGAAGTTGTTTGCAGGAGCCCCACTTTGCTGATAC	T	C	CAAAGAACAAATGATCGGAATGATAGGTACAGTTCGGGACAAGAGCAAGGTAAGAGTCAAGACATGCTCAGTCAAAGTGGGGGTCCTGACGATGAAATGC	NA	NA

Marker IDs	Genetic positions (cM)	Chromosomes	Physical positions (bp)	Flanking sequences/Forward primers	ICC 12968	ICC4958	Flanking sequences/Reverse primers	Annealing temperature (0C)	Amplified Product size (bp)
Ca-II-SSR52	133.42	Ca-*desi*-LG(Chr)1	6033499	ATACATAATTGGGCCGATGC	(AT)11	(AT)10	GGAACCAGAATTAGAGTTGAAGAA	59.65	150.00
Ca-II-SNP121	134.25	Ca-*desi*-LG(Chr)1	6088473	GATTTTCTTATTCAAATTGGTGGTGCCGGTGCATTTTATTCCTAGACTATAAGACCCAACTATCAAGTGGATGCACTTGCCCCCAAAATTTGGAGACAGT	G	A	GGCTTAAAATTGCAACTACACTTCCAATCTCTACTGATCAGAAATGGGACTTCTTGGAAACAATGATTAGCAGAGTACCAAACAAAGGCTATGAACACAA	NA	NA
Ca-II-SNP122	135.91	Ca-*desi*-LG(Chr)1	6115794	TTATTTATTTTTCTCGCTTCTTTTTTAAATAATTTGCTTTTTTTTATTGGCATTGTAAAGTTCTCATTAGTTATTAGTTATTGAGCATATATTCTACTTC	C	G	CATTAAAAAAAAATTGATAAATAAGATCAGGAGAATAATGATTTTATCATATTATTTTTATTAGTCTTTGGTTTATTTAAATATGACTATTATCTTAGGA	NA	NA
Ca-II-SNP123	136.81	Ca-*desi*-LG(Chr)1	6122870	CATTTTTATTCAAAAACAAAATACTTGTTGTTTTGACCTAATCACATTTTATGTGTAGGATGATTTGGGTCACTTATATAGAATATCTCTTTTTATCTTT	C	T	GTTAAACTATTGATCTTTTATGATAAAAAAATATATATTTTTAATATAAAAAAAAATAATGTATTTATTCAAAGCTAACATATCAAATATTTTTTCATGA	NA	NA
Ca-II-SNP124	138.61	Ca-*desi*-LG(Chr)1	6140856	AGATGAAAGTTCGTGTATCTTCTGCTTCAAATTCTCAATTGTCTTTTTATCCTCTACTAACTCCTGAGACATCTGATCAGCAAGACGTTTTGCTTCCATG	G	A	GTTGAAAAACAGAAGACTGCTAGGGAAAATCGGCGTGCAGATGAGGAGCATGTTAAAGTAGAGGAGCAGAAGAAGCTTGCTGAAGATAACAGGAAAAGCG	NA	NA
Ca-II-SNP125	139.51	Ca-*desi*-LG(Chr)1	6161052	ATTGGTGGCGTATATATTTTGATTGGGGGTGAGATGTTGAGGGTGATGAGTGTAGATGTCATTGTAATATGTTTCTTTGGGAGAATGAGTTGGTGGTAAA	A	G	AATCATATTTCACATAGTACACACCAATAGTAGAATTTTTCCAAAGCAACAAATCATTCATGTGATCCTGCAAGTGAACGTTAGCTAACAAAAACACACG	NA	NA
Ca-II-SNP126	140.41	Ca-*desi*-LG(Chr)1	6173569	TTTAATACTACGGTAGTTTTTTTTAAGTCTCATAATTTATTGTATTTATGTATAAACGATACAAGTTTTATTGAGTGAGTGGATAAGTTACTGCGTCCTT	T	C	AATTGGCGCATGGATCTATTTGACAGATGAAATTCAAATTAAAGGACGATTTTCCTATTTCAATAAATTCAAATATTAAATTGATTGATAATTACAAATT	NA	NA
Ca-II-SNP127	141.31	Ca-*desi*-LG(Chr)1	6184142	CATATTGGCGGCTGAATTGATTACTGACTCAAAGATGCGTGACCTTACCTTCTGCAGTTTTTTTTACTGAATTATATTAGATGTTATCGAAACTATTTTC	T	A	CAGATAATTTTAAATTATATTATAGATACAAAATAACTGATCTCCTTCGAGTATGCTATGTTAGATTTTTTTAGTGAAAATTTTTTTGATGATTAAGATA	NA	NA

Marker IDs	Genetic positions (cM)	Chromosomes	Physical positions (bp)	Flanking sequences/Forward primers	ICC 12968	ICC4958	Flanking sequences/Reverse primers	Annealing temperature (0C)	Amplified Product size (bp)
Ca-II-SNP128	142.21	Ca-*desi*-LG(Chr)1	6200464	ATTAATATACCATCAGTATCAATGGCTCAAATATTAATGGTTCAATTATTGGTGCCAATCCTACTTGTGTTGTTGGTAGGGGCAGGGGCTAAACCTGAGC	C	G	TCTACAATCCATAGTACAATCATAAGGACAATCTTTAGGACAAGCATACACCACATTGTAGCAATATTTGTACTTCTTTATCTCGCACTTTGCATACCCT	NA	NA
Ca-II-SNP129	144.91	Ca-*desi*-LG(Chr)1	6310910	TATACTGAACTCTGCAACGGTGGCTATGATGCCAAAGACTTTTCGTGTGCTTTTCGACACTATTACTCAGGAATGGACGAGCCTCTTGACCACTAAATTA	A	T	TAACAACACACTTTTTATCCAGTTTCATTGCATCAAATTAATGGTCTTACCATACAAGAAAACAAATACATAAAGAAGTTACATGATCTATAAGAATACT	NA	NA
Ca-II-SNP130	145.81	Ca-*desi*-LG(Chr)1	6397062	GCAAGTAATCATTAATAATTCACATGCTTTTTTTTTCTTTACAGGTTTCTGCTGCAACTACCACAGACAATAGCTGTGAGTCTGTGGTAATGAGTGGTCA	A	T	CATAACATAAAATCAATTCATGATGCAAAAAAAGTTTGACTGACCCAGCTGGGTTGTTGGCATCCCTTTGAGGATGCTGAGTTGGGTTTTGCTGTTGGTT	NA	NA
Ca-II-SNP131	148.51	Ca-*desi*-LG(Chr)1	6745622	ATGATGTTTTAACATGTGGTTTCCAATGTTTATTTTTTGTTGTTTTTGTCTTAGGTTCTTCCTGAAACGCTTGATGGCAAGTCTTTTGATTTTCCTGCGG	C	T	ATGATACATTTGCTAATTAAACAACCAATAGTCCTTATAATGTACTAGTAGTAATAACCATAGAATCTATGCTTAAGAACAGCAAAAATGATCACCACAT	NA	NA
Ca-II-SNP132	149.41	Ca-*desi*-LG(Chr)1	6773488	AACGAGACCCAATAGCACGTTTTCCAGGAGGAAGAGGTACTAATTCCCAAATATTGGTTTTGTGCAATGCAGAAAGTTCTTCTGCCATAGCCTGCTGCCA	T	A	TTGTCTATTCCACTTACTCAACTTCATTTGTTTCTTTCTTAACCTCTATTCACAGTTTGTCTGAGCCCTCTTCCTATAAAGAGGTTGTTCTTGATCCTCT	NA	NA
Ca-II-SNP133	150.31	Ca-*desi*-LG(Chr)1	6829116	AGCTAACATGTAGGTCATGTTGCTAAATTTTGATCCCTACTTTAACTCTCTACACGAATTGAAGGATCTTTTTATCACTTGTGACTTGCATTGTTATTGA	A	C	TACACTCACCTCCTTAATATGAATGAATAAACACAAACATACCAATTATTCTCTCATTCATTGATAGAAGTCAACCCCAATGAATTGATAACCAACGAAA	NA	NA
Ca-II-SNP134	151.21	Ca-*desi*-LG(Chr)1	6862783	TTGATTTATTTAGGAAAATTTTGCTTGAGCAGGAGAAGGATTTGCCATGATGTCTGGAACTAGCATGGCTGCCCCACACATTGCTGGAATTGCTGCTCTT	T	A	TGTGCTAGAATTGGGCTTCCTGCTCTGTCTAGAGTTGTTGATGTTGTAAGCAAGGCTGATTTAATGGCTGCAGGGCTCCAACGTGGATGTTTCTGCTTTA	NA	NA
Ca-II-SNP135	152.11	Ca-*desi*-LG(Chr)1	6918109	AAATTCTCGCATTCTACATCACAGGTAAGAACTTCTTGATTCGCCATCGTGACATTCTCCTTCTTTATGGTTGTGTGTTATTTTTCCATTTACTTCTCAT	T	G	TTGAAACTTTCCCATGGCTTGCCTGTTGCAACAAAAACATACACAGAGGAAGAGTTAGGGTTTCAAAATAAAATTGGGGTTTTAGTTTTTTTAATCAATT	NA	NA

Marker IDs	Genetic positions (cM)	Chromosomes	Physical positions (bp)	Flanking sequences/Forward primers	ICC 12968	ICC4958	Flanking sequences/Reverse primers	Annealing temperature (0C)	Amplified Product size (bp)
Ca-II-SNP136	153.01	Ca-*desi*-LG(Chr)1	6942682	TTGATTTATTTAGGAAAATTTTGCTTGAGCAGGAGAAGGATTTGCCATGATGTCTGGAACTAGCATGGCTGCCCCACACATTGCTGGAATTGCTGCTCTT	T	C	ACTGATAATGTATAATATAAAAGAAAACTATGGGGCCTAGGTTAGAGCTCCTAAAGAATGAGACAAAAGGGTAAATTTACAAATCAGGAGAAAGAACTAGTATACAAAAAGAATGACACTAAACCACAATTTCTAATCTACAAATCGGGTATATTTTGGGAGCATATACAAAAGGGTGACAGACATAATAATTT	NA	NA
Ca-II-SNP137	153.91	Ca-*desi*-LG(Chr)1	7046558	AGAAACGACGAGCAGTGACAACGGCGCGCAGTGACGACGGCGAGCAGTGGCGACGGCGAGCAGCGACCACGACGAGCAGCGACCACGGAGAGCAAGTGAC	C	T	AAGAATAAAGATTGATGAATGAAACACATAGAAATAAGATTAAAAACTTGAACCTGCGCCTGACGTTATCCTGGTCGTCGTCGTTGTCATTGCTCGTCCT	NA	NA
Ca-II-SNP138	154.81	Ca-*desi*-LG(Chr)1	7062230	CGCCACCCTGTAACCATTCTTGTATAGATTAGCTCGTTCTTTTCGTTTGTGATGACGGTTGTTCCCCATTTTTTGGTACCACTTGAACGGGGCTCACCCA	G	T	AACACCAAAGAAGATGGAATCTGACAATGAAATAATTGATTAGGAAAAAAGTGGTAAAGCTCATAGAAGCTGGTCTCATTTACTCCATCTCAGATAGCTC	NA	NA
Ca-II-SSR53	155.71	Ca-*desi*-LG(Chr)1	7137592	AACCTTCTCATGCACAAAATCA	(AT)14	(AT)17	TGGAGGTTTGTCTTTTCTTTTGA	59.62	176.00
Ca-II-SNP139	156.61	Ca-*desi*-LG(Chr)1	7141328	CAAAAAAAAATACTTCTTTTCTCAATAGCCTAGTACCTCTTTCATCGGGAAGTAACTTTTATCAACAAGTAGTTTGGCATTCCACCCTCCAGTGGCGGAT	C	T	TATAGAAGGTTTTATGGCAGCTTAAAATAAAAATAATAGTATTATCCAAAGGATTTTTCGTTTTCTTGTGGGGGCAAGAGCCCCCATTAGTTACACAGTG	NA	NA
Ca-II-SNP140	157.51	Ca-*desi*-LG(Chr)1	7166394	CGCAGATGACAATAAACCACTTAGTATCTTCCTTGTCAAGCAATCCCTATCTCTTTACCTGTTATTGTGCTAGGGCTTCCCTTCTAACAGCAAATAGGCC	T	A	GTTGCAGTGTTCTTTGATATTTCAAATTAGAAAATAAAAGTTACATTGTACAAGTGTTTTTTAGGAAAGATTTTTGCTCGAAAAGACTTAGCGCTTGGTC	NA	NA
Ca-II-SNP141	158.41	Ca-*desi*-LG(Chr)1	7204364	TCGTCTTCTGCTGCTGCACCTATTATTGTCTGACAGTAATGGCCATTATGGTCATAAACTGCTCATTTTCTGCTGAACCGTTTTCCTCCTATGAAATTCT	G	T	TAGTAAGAGATTGTGAGTAGGAATGTGTGTGTAAAACTGAGTATTTTGGTAATTTTTATAGTCCTAGGTGCCTTCATGAGCTTTAATTACAAGAAACCAT	NA	NA
Ca-II-SNP142	160.21	Ca-*desi*-LG(Chr)1	7282702	GGCAGGCATGTTGGGGTCGTAATCCCTCAGGTGGGAGAAATACAAGCAGCAAAATGGCCAAACAAGCTGCATTGGGATTTGTTAAACGGACATTGGAGCG	G	A	CTAGACGAACAATACTAAGCTGGGAAGAAGCAGCAAAGAACATATCCTTGAATGAAGGTTCGTTGAAGCAGCTCTTGCCTGTATCTTCAAATTGACGGCA	NA	NA

Marker IDs	Genetic positions (cM)	Chromosomes	Physical positions (bp)	Flanking sequences/Forward primers	ICC 12968	ICC4958	Flanking sequences/Reverse primers	Annealing temperature (0C)	Amplified Product size (bp)
Ca-II-SNP143	161.11	Ca-*desi*-LG(Chr)1	7349728	TCTATCAGTCACAATAACTTTAGAAATTAAAGTCTCAGATTTGAACAACTTTCGTACCTTTTCAAATGCCCAGATGAAGTTATCTTGTGTCGCTCTTGTT	A	C	TGATAGCACATACAAAACAAATAGATATTGGTTACCACTACTTGAAATTGTCGGTGTCACATCTACTAGTTTGATATTTTCGGTTGGGTTTGCTTATTTG	NA	NA
Ca-II-SNP144	162.01	Ca-*desi*-LG(Chr)1	7380996	TTGATTTGGGGAAACATCGAGTTGATCTCACGAGGTTGCTTCCTCTCACTCTAGAGGAGCTTGAGGAGGAGAAGAGCTCGGGGAAGTGGACTACTAGTTT	C	T	GAGCATTAAGTCCATCTCTTGTCGAGCTGGTGTTTTCACCAACAACCGTATACCCGAAACTGACATTCATCACAGCACCCTTAGCCTTTCCTGATAACCT	NA	NA
Ca-II-SNP145	162.91	Ca-*desi*-LG(Chr)1	7383209	TTATACCACCAGTCCAGATCAACCTTGATTTGTTACAATCTTCACCTTTCAACCTAGAAAGGATTTTTACAATCACCTTTCAGCCTTGAAATGATTGTTA	C	T	CGATGAAGTTGATGGGATAATGGAACTATCATTAACTCTTGATGAGTGGATGAGACTAGATTCGGGTGAGATTGATGATATAGATGATATCAGTGAACATACTTTCAAACTTCTTGCAGCCCATCATGCCAACTCTTTCGACGTTGTCCGTGAGAGTTCAAAAGGAAGGAAGAAAAAAGGCAAAGGTCATGGCAGGAAATGTGGTTTGCTAGGAAAC	NA	NA
Ca-II-SNP146	163.81	Ca-*desi*-LG(Chr)1	7404716	AAATTTTTGGTTATTTTTCTCTGTTGGAGAGATATGTATTTTATAGTCGCACCCTTTAATTAATTTCCTTAATCCCACAAGGTACATTTGGATCACCATG	T	C	AAAAATTGGTATTTCGTCTATATTTTATCAATGAATACAAAGTTAATATGAATTATATGCAGGTTAAAAATCATGTTTTATATGGAAAATAAAATCTCTC	NA	NA
Ca-II-SNP147	165.31	Ca-*desi*-LG(Chr)1	7432885	AGATAAGTCCCTAATGCAAACTAGAAGTTTGGAAGGAGCCCTTACCTCAACGTTAGCTTTAACGTGCGTTTCCGGTACCGCGAGTAATTCCGGTAAAATC	C	T	TCTAGATCAAAACCTCATTTCTCGAAGAGATAGAAGGGAAAGTTGTTCACCACCACGCTACGGTGCTAGTGAACTAATTTGGAAGCGGCGTTGCGAGCGG	NA	NA
Ca-II-SNP148	166.06	Ca-*desi*-LG(Chr)1	7481763	TTATACCACCAGTCCAGATCAACCTTGATTTGTTACAATCTTCACCTTTCAACCTAGAAAGGATTTTTACAATCACCTTTCAGCCTTGAAATGATTGTTA	T	C	TTTATCTCAAACAAGTGTTGAGAAGTTTAAGATCCCAAACACTTTTATCATACACATCATTTAACTCAAGTCTATCTTTTATGTTAGATTGAGTGTGTTT	NA	NA
Ca-II-SNP149	166.81	Ca-*desi*-LG(Chr)1	7490836	TTGAGATCACAAGGAAGTTTATGAACAAACCTGTGAGGATTCTTGTGAAGCGTGATGAGCTCACCTTGGAGGGTATTAAGCAGTTTTATGTCAATGTTGA	T	G	GCCAATCAACCTTCCGTCTGGTGTTCACAAAAATAACACTCTGGGTGATTGCCAATGTCTCATAAAGATCACAGAGGGTGTCAAGCTTCCATTCCTCTTT	NA	NA
Ca-II-SNP150	167.56	Ca-*desi*-LG(Chr)1	7515845	GCGATTTGTGTTTTAAATAATATTATTTTTGTGGTTGTCTAAGTGGCTTGTGATTTGTGTTTTGAATATTTTATCTATATGGTTGTCTAAGTGATTTGGT	C	T	CCAGCCACTTAGGCAACCACAAAGATAACAATATTTAAAACACAAATCACCAGCCACTTAGGCAACCACAAAGATAAAAATGTTTAAAATACAAACCACC	NA	NA

Marker IDs	Genetic positions (cM)	Chromosomes	Physical positions (bp)	Flanking sequences/Forward primers	ICC 12968	ICC4958	Flanking sequences/Reverse primers	Annealing temperature (0C)	Amplified Product size (bp)
Ca-II-SNP151	169.06	Ca-*desi*-LG(Chr)1	7558735	TCGATAATGTAATCCGAGACTCATTGTCCTTTTAAGGTATCTCATGACTCTTTCGATAGCGTGCCAATGCTCCATACTAGGTCTACTAGTAAACCTGCAC	C	A	GGTGAAGGTGTTAGACAAAATGAATATGCGAGCATCATTGGCAGCCTCAAGTATGCCACTGACTGTACTAGACCCAACATTGCCTATGTCGTTGGATTGT	NA	NA
Ca-II-SNP152	169.81	Ca-*desi*-LG(Chr)1	7595734	ACGAGTTTTAATGTGTTTTGATGCGCGTCGCGCACTTCAAGCGTGGATTGGCAGTGGCTTCAGCACGAAATGCGTTTCAGGCGCGCTTCAAGCGCACAAT	A	G	AAATGAAAGCTAGGATCCATAGATACAACTTTTAGGAAGACCAAGAAACCAAATTCGGAATCCAAAGAGGAGAAAAATGTACTACACTCCAGACAAAAGA	NA	NA
Ca-II-SNP153	170.56	Ca-*desi*-LG(Chr)1	7609528	TGGAAATATCAAAATTGTTCTTCGTAATTTTACATCATTAGATATTGAATCTTTTTTTGTTGTTGAGAATTTTGGTCTATTGAATCAACAAGTGATGGTA	C	T	AATGAGATGTCATCATTATTACGATACACGAGAGAAATAATGAATTCAGAAAATATCATATGATTTAGTGTTTCTTCCTGAACTTTATGCAATTTCTCTT	NA	NA
Ca-II-SNP154	171.31	Ca-*desi*-LG(Chr)1	7623150	TTTGTGGCGGCCTTTGTAGCCACAAAATGCAAAGGCTCAGGCTTTTGTGGCGGTCTTTGCAGCCACAAAGTGTAACATCCCTTCAGGAATTTGTGGCGGC	C	A	TGATTTTGTGAGGGCTTAGGCAGCCACAAAATCCACCTAAATTTTTAAGATTTTTGTGAGGGATTTTTCAACCACAAAATCTAAATTTTGTGAGGGTAAA	NA	NA
Ca-II-SNP155	172.81	Ca-*desi*-LG(Chr)1	7684333	TTTGTGTATTTTGCTATTTTTTAGTATTTTATGTTTTGTTTATATACTGTTTTTAGGCCTTCGCAATAGCGGTCATCCCGCTATCCCATTTTTGGGATTC	G	A	GTGTTGGTGCCGGACATTAGACACATCTTTAATCTAAAGTGTCAATATTACATAGAAGATAAGTCTATGTTGTCAAATCTTAGATAGAAGCGCGGAGCAC	NA	NA
Ca-II-SNP156	173.56	Ca-*desi*-LG(Chr)1	7721091	TAGTTCTAACACCATTGAAGGAGTTTTATCAAGAATTATTCATGAATCTTTCTTGTAATTCTTCTTTTATCTCTATCTTTTGTATCTCTGTCATGAGTAA	C	T	TGAGAAAAATAATTCAATAAATTCATTTATATAAATAGAAATCAAATCAGACAAATAAGAGTTTCATCTTGTTTCACTCATCCCTAACAAATAGAGTTTA	NA	NA
Ca-II-SNP157	174.31	Ca-*desi*-LG(Chr)1	7780059	ATCCCAATTATAAAATTTTAATATCAAATGGCCCATTAGCTCAGTTGGTTAGAGCGTGGTGCTAATAACGCCAAGGTCGCAGGTTCGAGACCTGCATGGG	T	C	ACGCGGGTGACCCGGGTTCGATCCCCGGCAACGGCGTTTGTATTTTTTATTTTATTATTATTTTATCAGAACTTAGAATTAAAAAATGAAAAACAATTTG	NA	NA
Ca-II-SNP158	175.06	Ca-*desi*-LG(Chr)1	7851353	GCCCCTGTGTTAAAGCCAATCGATTGGGAAAATCGATTTCCTGAGGGTTTTTCGTGAAAACTGTACTCTGGGCTTAATTCAATCGATTGGGAAATCGATT	A	G	CGATTTGGCAATCGATTGGATGCATTACAGGAGCTCAGCCATTTTGACAAAATCGATTTGGAAATCGATTGGCTTTAATACAGGCGCTCAGCTATTCATT	NA	NA

Marker IDs	Genetic positions (cM)	Chromosomes	Physical positions (bp)	Flanking sequences/Forward primers	ICC 12968	ICC4958	Flanking sequences/Reverse primers	Annealing temperature (0C)	Amplified Product size (bp)
Ca-II-SNP159	175.81	Ca-*desi*-LG(Chr)1	7904001	TGGATGCATGGTATGATATATGTATGCAGGGTGATAATGCTGATTTTTTTATCGATAATAGTGATGTTATAAAATTGTGATGAGTTTATATGATGATTAC	A	G	CAATATTCTCATCACAATTTTATAACGTCATTAATCATACATAATCATAATCATCGTATAAACTCATCACAATTTTATAACGTCACTAATCATACATAAT	NA	NA
Ca-II-SNP160	176.56	Ca-*desi*-LG(Chr)1	7944510	TTCAATTGCAATTATGTTGTTGTTTTATATTGTCATTTTGTTGTGTTGTTGAACTAAAGTAAAATCATTTTTATACTTAATTCTGGAACAAATGTCTTGC	T	A	GTAACGATCAAAAGTGAAACAACAAGCATCGCACAAGTTCAACCATATCAATTCAGAACTAAACAAAATTCAAACAAGATAATGTTTCAATTCATACTGA	NA	NA
Ca-II-SNP161	177.31	Ca-*desi*-LG(Chr)1	7981054	TTTTTCTTGCATGAATTTAGTTTGCTGTTTTAAAAAATTAAAATTAGAAAGCAGTTTAATTTAATTATTTTGGCAGGTCGAAGCTGTTTGAGATGGAGCC	G	T	AGCACCAATATCAAAATTGTTTTACTTGTCATAGTAGATCAATTTTGCCACTCTCTACAAGTGGCTTTATTGATTTGTAACATATGAAGTAAAGGACATC	NA	NA
Ca-II-SNP162	178.06	Ca-*desi*-LG(Chr)1	8074774	CAGTCCTCACACTGTGAGATTTTCCACTAAGTGTTTAAAACAAAGAACCTTCTTGATCTTACAACACCTAGTCTAGATCAACCTTGATCTGTTTCAATCT	T	C	AACTCAAACTATAAGAGTTTCTTTATAGTTTGTTTAGAGTACATTGAATCCTATCAAAGTTAGATAGGTACTGTTATTGCTTTCTGGGTGGAAAGTAATA	NA	NA
Ca-II-SNP163	178.81	Ca-*desi*-LG(Chr)1	8092533	TGTTGTGTAAATGTTTTGGATTTATAATTGGAGAAACTTTTCCGCTGCTTGTAATTTATAATGACTCAATTATTTATCCAAATGCATTTTCTGATTTAAA	C	T	AAGTTAGTCAAACATGAACTTGTTGAAGGCTGTAACACCCCGTTTTTCAAAGCGAGGGTATATATATATTTTTTTTTAAAGTAATTAAAAACGAACAAAG	NA	NA
Ca-II-SNP164	179.78	Ca-*desi*-LG(Chr)1	8111129	TAACATTATAATGTTTTGTAAAATGAAAAATTATATTTGACATGTTGGCACATTCATCTTATTTTTAGCTTCCCTATATGCGCTCACTCTCTCTTGTTCA	A	C	TCCAGTTTTAAGAAAAGTAACATTTTCTAAAAAACTCCCCCTTAATATTTTCAAAAAGATATCCTTCAAAATTCAAAAACACAAGTAAGATAAGATGGAT	NA	NA
Ca-II-SNP165	180.54	Ca-*desi*-LG(Chr)1	8169336	GCACTGGCCCCATCTTCTTCCCAAATGCACTGGTATCGAATAGGTCTCAGCACACTTTCAATAGTTTTGGTAGTCCCTTTGCCCAACCATCGTCAAGGTT	A	G	CATCCAAATGAAACTGCAACAGTATTGGACTAGATGATGTCCTTCACTTGAAACAATCAACTTAAGTGGGACATGTCATCTGTGACTTGCACCAACGACT	NA	NA
Ca-II-SNP166	181.30	Ca-*desi*-LG(Chr)1	8196164	ACCATTTGAACACACGAGCAACTTCACTATCAGATCTACTATTGCTCTTCTCTTCTGCATTTGAATGCTTTGCTCGATGACTCCAGTTTGTTGGGTAATA	C	G	AGATTCACAACCATAGTCAACAAAATACCTTCTCTTGAGAGATGTCGGAATGAGTTGGTATTTCAAAATAAGATTGATAATAGTCACACAATGGCTGCCA	NA	NA

Marker IDs	Genetic positions (cM)	Chromosomes	Physical positions (bp)	Flanking sequences/Forward primers	ICC 12968	ICC4958	Flanking sequences/Reverse primers	Annealing temperature (0C)	Amplified Product size (bp)
Ca-II-SSR54	182.06	Ca-*desi*-LG(Chr)1	8205909	CTTTTAAAGCAATGTTTACCTTTTC	(AT)14	(AT)11	GCGACCTAGTTCTCGAAACATC	57.06	136.00
Ca-II-SNP167	182.82	Ca-*desi*-LG(Chr)1	8210526	TTCATACTCGAAAGAGAAGAAACATGCAATATGCACCGGACATCAGATAATGAGCGCTTGGAGCGCGTCTGAAGCGCTTTTTCCAGCATCTGCTACTGCC	T	C	ATAGTGAAAAAGATCCCTAAAACAGTAAAAATCTGTGTTTTTATGGTTGCTGGTGCGGGTCGCGCTCCACAGGCGCGGAAAACGCGCTCCAGACGCGTTT	NA	NA
Ca-II-SNP168	183.58	Ca-*desi*-LG(Chr)1	8232371	TAATTTAGTGAATTTTTTTGTTGCAGGAATTAAATTATTCATTTAATTATTGTTAGGGACTTAATTGTCTATTTTTAATTATTATCAAGGACCTAATTGT	C	T	TTTATGTGAACCACAGTTAAAAAATTGAGATTTTTTTTACTAATTTGTCTGATGACTCGTCAACTTGATTTTCCCACGTGTAATTTCTATATAAAAAAAG	NA	NA
Ca-II-SNP169	185.10	Ca-*desi*-LG(Chr)1	8345900	AGTTTTGACATAGAGAATGGACCCAAGGCTGCTGAGTATATTCAGGTTTGTTTTTATTATTTCCACTACGGGAAATGCTGATTGTTACTAGAGATGTTTG	C	T	GGGTTTGGACTTATAAGCAATCGGCTGACCATACATGCGAGAGTGGGTAGTTATTATTTATAAGTGTTAGATGTTGCCACATATTATTCTTCTCTTTCTT	NA	NA
Ca-II-SNP170	185.86	Ca-*desi*-LG(Chr)1	8383527	TCGCCCACACAATAAGCGAAATCTTCACCATCGGATTTGATGTACTCATAGTTGATATCAATGCCATCTATTATGTTAAAGCTCCAAGGATCCTTGACTT	T	A	CGCACTATTTTGTATTCATAAGCAACATTTTGGGTGGGAATTGCAATGCTGCTGCTTGGCATCAATATACACAAAATGTATCCATTCTACAATTGTCCCAATAAAGCATTACCTTACATGGCTTTTTATGGAAACAAAAACAAGTCACCTTATATAGATAGAGCATCTAGTAATTACACAAAAGCCATATTTATTAATGTATATACCCTATGACTTATTCCATTTCTCTTCATT[illegible]	NA	NA
Ca-II-SNP171	186.62	Ca-*desi*-LG(Chr)1	8453603	AAATCAAGTATGTGAACCAAGGGTGGCCTTTGTTTGTGAGTGAATTTGGGGGTGACTTAAGAGGTACCAATGTCAATGGCAGTGAATTTGGGGGTGACTT	G	A	CCTGACATTCTTGTGTATTTGTTAAAGAGTATTCAAATCAGATAAAATTCACTCACAAACAAAGGCCACCCTTGGTTCACTAAGTAACATTGGTACCTCT	NA	NA
Ca-II-SNP172	187.38	Ca-*desi*-LG(Chr)1	8477054	TCGCCCACACAATAAGCGAAATCTTCACCATCGGATTTGATGTACTCATAGTTGATATCAATGCCATCTATTATGTTAAAGCTCCAAGGATCCTTGACTT	T	C	CGGTGGTCGTGATGATAAACATCCATTCCATCCTGCTTCAAAAGTAGATTGGGCTGACAATGCTGTAAACTCACTCAAAAAGATCATCCAACAATACAAC	NA	NA
Ca-II-SNP173	188.90	Ca-*desi*-LG(Chr)1	8501184	GGCATCGGGAGTCTGTTTTAAATATATATAAAGACTTATTTACGCTTTAAACCCCTCATTAATCTATTCTTGTGTGTGTGACACTATACGTTTCCGAAAT	A	G	CGAATCACATGACATTCTCGTCACTTGATTAGTAGTGATGCATTGCTAGATAATGTTCGTTTTTTATAATATTAAATAGATGATATAATATTATTATCAA	NA	NA

Marker IDs	Genetic positions (cM)	Chromosomes	Physical positions (bp)	Flanking sequences/Forward primers	ICC 12968	ICC4958	Flanking sequences/Reverse primers	Annealing temperature (0C)	Amplified Product size (bp)
Ca-II-SNP174	189.66	Ca-*desi*-LG(Chr)1	8543377	AATGAAGTATATGCTCCAGTAGCTCGATGGGACACAATTCGGATCTTGTTGGCTGTGGCAACTCAAAAGGGATGGAATGTGTATCAGCTTGATGTGAAAA	T	G	TTTGTGCAGCCTATATACCTTGTCTTCCTCTCCCTGCTTTATGAAGCCCTCAGGTTGATCTACATACACTTCTTCCTTCAGTTCTCCATATAAGAAGGCA	NA	NA
Ca-II-SNP175	191.18	Ca-*desi*-LG(Chr)1	8653369	TATAATTTCGTCGTCTCGATGCGACGTTTTTTTGTTTTAATATGGCATTTTTATTTTTTCTGTCTGTGGTGCGGAGTGTTCGTTTTGATTCAGCTATGCA	A	G	TATGATAAAATTTAAATATTCATAATATATATGTTTTCAGATTTATAACGTTAACGACGTCAAAGTTATTGATTGAAATCTATAATAGATTAAAACTACT	NA	NA
Ca-II-SNP176	191.94	Ca-*desi*-LG(Chr)1	8896611	ATGACTTCTCAATTGATGACCATATTAATTTTTAATTTTTTTTTTTTTGCATCATTATAGACTTGGGATGGCCCAATCGGTCATGTCAAACAACAAACT	A	T	TTGTACTACACTTTTATGCTTCTCTTATGAATTGTACTACACTTATATCATTACAATTGAAGCGCATGTAATTATAAACCATAAGTTTACAAATGATGAT	NA	NA
Ca-II-SNP177	192.70	Ca-*desi*-LG(Chr)1	8987902	CTTTTCTATCTGTTTCCCATTAACACAAACTTTTCTTTTTTTCAACAATGGCTCCAAGAGACAGACCCACCGCACTACCCGGGCCCAGCCCATCAGCCCA	C	A	AAGTACCAAGCCAAACTCGTGTTTTTTTGCCGGGGTCACGGATCTCAGCGGCGTAACGACCCCACGGACGTTTCCTAACGCCTCTAAAACGAATCTCTTT	NA	NA
Ca-II-SNP178	193.46	Ca-*desi*-LG(Chr)1	8988178	ATTTTCCAACACCGTCGGAGATGCTTAATCTTAACATTAGTAACAACAGCACACGCAGCCCAAGTCAGAGTAGCACCCTTGAATCACCGTCACCGCCGCC	G	A	GTGCAAACGCGTCGAAAAATAAAACAGGACGGGATACCGGAAAAGCCAAAGAAACAGCACCGACGCCGGTGGCGGAAAATGGTGTCAGAGTTAGATCAAG	NA	NA
Ca-II-SNP179	194.22	Ca-*desi*-LG(Chr)1	9116083	TCAGAATTGAGTGAATGAGTACTCTTTGGAAAGCTTATGGTGTCTACTTTATAGATAAAATTTTATTTTATTTTTATTTAATTCCAATTCTTGCAGTTTA	C	T	AGTCGTGATGATTGATCGAGTATTGATGTTGAAAAATGGGTTACAAGCTGGACCAGAGTTGCTGTTACTGTTTTGTTTTATGTCATTTGTGTTTATGTTA	NA	NA
Ca-II-SNP180	194.98	Ca-*desi*-LG(Chr)1	9161984	ACCGGAGAGAAAGTCTCTTTATAATCAATCCCTTCCTTTTGAGTATAACCCTTAGCTACAAGACGTGCCTTATATCTCTCCACATTACCATTGGAATCCC	T	G	TGAGGAGTACAAGTCTATGCAAGACAATTCAGTTTGGGAACTTATCCCATTACCCGAAGGAGTGAAACCCATTGGTTGCAAATGGATTTTTAAAACCAAG	NA	NA
Ca-II-SNP181	195.74	Ca-*desi*-LG(Chr)1	9182446	TGTTGTGTAAATGTTTTGGATTTATAATTGGAGAAACTTTTCCGCTGCTTGTAATTTATAATGACTCAATTATTTATCCAAATGCATTTTCTGATTTAAA	A	C	TGCCATTACAGTGTTCTACTTCTATCAATCAATCAATGCTATTCTACATAAAAGTTTACAACAGGATGTTAACAAGCAAAGAAACAAAAACAAAACTGTGTAACCTGCTAACTTAATTATGCTCCTCTATAATCCCAATATCCGGCCAG	NA	NA

Marker IDs	Genetic positions (cM)	Chromosomes	Physical positions (bp)	Flanking sequences/Forward primers	ICC 12968	ICC4958	Flanking sequences/Reverse primers	Annealing temperature (0C)	Amplified Product size (bp)
Ca-II-SSR55	196.50	Ca-*desi*-LG(Chr)1	9870501	GGACCAAAACGGCAATTAAA	(TAT)15	(TAT)19	TAGGCATGTCTGGCATTTTG	59.81	144.00
Ca-II-SNP182	197.26	Ca-*desi*-LG(Chr)1	9983100	TAACATTATAATGTTTTGTAAAATGAAAAATTATAT<br>TTGACATGTTGGCACATTCATCTTATTTTTAGCTTC<br>CCTATATGCGCTCACTCTCTCTTGTTCA	T	A	CAGATGTCCAATTGAGAGTTGCCCTGTTGACTG<br>TATTCATTGGACATCTGCTGCACAACTATCATT<br>ACTCGAAGATGAAATGCACCGTATAGAAAGAGT<br>CAACGTTGCTCTAATGCTTTCAGGAATGGGATC<br>AGCTTCATTTGATGTTTTCAGAATGGCGAGTTC<br>GCGATGGGAAAAGAGACAGTCAAAAGTCTTGA<br>GGATGAAGTTAAAGAAAGAGCAAAAAGAGCTG<br>[illegible]	NA	NA
Ca-II-SNP183	198.78	Ca-*desi*-LG(Chr)1	9986169	GCACTGGCCCCATCTTCTTCCCAAATGCACTGGT<br>ATCGAATAGGTCTCAGCACACTTTCAATAGTTTTG<br>GTAGTCCCTTTGCCCAACCATCGTCAAGGTT	A	C	GAAAAAGGTGAGTCATCATACTCAAAAAACGAA<br>GCTCAGAAAAGGAAAAAGTACCTCTAGGAGTTT<br>GTCGGAGCTCGAGTTTAAGGAGCTAAAAGGGT<br>TTATGGATTTAGGATTTGTGTTTGCTGAGGAAG<br>ACAAGGATTCAAGATTGGTTTCTTTGATACCAG<br>G	NA	NA
Ca-II-SNP184	201.06	Ca-*desi*-LG(Chr)1	10562402	TCTATCAGTCACAATAACTTTAGAAATTAAAGTCTC<br>AGATTTGAACAACTTTCGTACCTTTTCAAATGCCC<br>AGATGAAGTTATCTTGTGTCGCTCTTGTT	G	C	GGCGGTTGGGGGTGCGATTAGAAGAAGAGGA<br>AGAAGAAGAAGGTTGGGAAGATTCGTAACTGA<br>AAACGACGTCATCTTCGGTTAGTTCGAGGGGC<br>GTGGAATTAACGAGAGGCGACGGCGCGTGGG<br>GTTGGATGCCGAGGTAACGCTCCGCCT	NA	NA
Ca-II-SNP185	201.82	Ca-*desi*-LG(Chr)1	10739203	TTGATTTGGGGAAACATCGAGTTGATCTCACGAG<br>GTTGCTTCCTCTCACTCTAGAGGAGCTTGAGGAG<br>GAGAAGAGCTCGGGGAAGTGGACTACTAGTTT	C	T	TCTCAATACAAATTAGTCTCTCCATTATTATTTT<br>TTTGAATAGATACAACCCAACCCCTCATATACA<br>TAAAGAAAAACATACATATATATATGTCTTTAAT<br>AATCACCAATAACCACCAACCCTTACTCTTCAT<br>GTGGCCTTGCGTAAGATAGTGAAGGAACTACC<br>ATCCCCTTTAATGAAATCGTACCTACGAAACAA<br>ATGTGCCACAATCAATGATGCGGTGAAGGTTAC<br>[illegible]	NA	NA
Ca-II-SNP186	202.58	Ca-*desi*-LG(Chr)1	10785548	TTATACCACCAGTCCAGATCAACCTTGATTTGTTA<br>CAATCTTCACCTTTCAACCTAGAAAGGATTTTTAC<br>AATCACCTTTCAGCCTTGAAATGATTGTTA	A	G	GTACAAGATGCACAGATTGATGAAGATCTCAAA<br>GATCTTAAAAACTCAAATAGGCAGATCTTGCTG<br>GAGATAATAGCCAGCTGCTATGGGTGTGATTTC<br>CCACATTTTTGTTTTGCTCTTCTTCATTTCTTCT<br>TCATTATTTCCTTCATTTGTATTTTCAGTCCTAA<br>ATGAGTGAGCAAAAGCTTCTCTAGTCTTTTT	NA	NA
Ca-II-SNP187	203.34	Ca-*desi*-LG(Chr)1	11291737	AAATTTTTGGTTATTTTTCTCTGTTGGAGAGATATG<br>TATTTTATAGTCGCACCCTTTAATTAATTTCCTTAA<br>TCCCACAAGGTACATTTGGATCACCATG	C	T	TTTGCACCCCCACCAAATTGTATTGGTATTTCA<br>AATACAGAATATCAAATACAGAATATCAAATTCA<br>GGAAGGATTACAAAGAATAAAATAAATAAAAGG<br>TTGCCAATTGCCATTACAGCACACCAATCAGAA<br>AACACCTACTAGCCTCGTGGGAAAAAAATAACA<br>TCCTGCTACTTGTGATTTATTCTCATTCAACACA<br>AGCTCATCAATGCGTTTGATTGCGTGCTTTTTC<br>[illegible]	NA	NA
Ca-II-SNP188	204.10	Ca-*desi*-LG(Chr)1	11899768	AGATAAGTCCCTAATGCAAACTAGAAGTTTGGAAG<br>GAGCCCTTACCTCAACGTTAGCTTTAACGTGCGTT<br>TCCGGTACCGCGAGTAATTCCGGTAAAATC	T	C	CAGCAACTTTGCAAGCAATCATTTCGTTTGTTG<br>AAAATGGGGGTGACCGTGTCAAATTGGTGAGG<br>GCTGGAAAGCATCAGGTGGTTTTTCTCGTGAAA<br>GGACCAATTTACTTAGTCTGCATCAGCTGCACC<br>GAAGAGCCTTATGAGTCCTTACGGAGGCAATTA<br>GATCTCATTTATGGCCAGATGATTGTCATACTG<br>ACAAAATCAGTAAACAGATG	NA	NA

Marker IDs	Genetic positions (cM)	Chromosomes	Physical positions (bp)	Flanking sequences/Forward primers	ICC 12968	ICC4958	Flanking sequences/Reverse primers	Annealing temperature (0C)	Amplified Product size (bp)
Ca-II-SSR56	204.86	Ca-*desi*-LG(Chr)1	12228747	CGTCCAATCCTACCAAGTATTTC	(AT)6	(AT)7	TGAAGACAAGAAAATGGCCC	58.94	169.00
Ca-II-SSR57	206.38	Ca-*desi*-LG(Chr)1	13530166	TCATCTTCTATGGCTACTACTTTCAA	(TAT)5	(TAT)6	TTCAAACATTCTCTTTACTAAGTGTCA	57.88	210.00
Ca-II-SNP189	207.14	Ca-*desi*-LG(Chr)1	13591156	TTATACCACCAGTCCAGATCAACCTTGATTTGTTA<br>CAATCTTCACCTTTCAACCTAGAAAGGATTTTTAC<br>AATCACCTTTCAGCCTTGAAATGATTGTTA	G	C	CCATTTTCAATGATTCATTTATAGGTGAAACTTT<br>TTAAATTAAGTTGAAGCTCAACCATTTGAATAAC<br>CATGATCTGATTCCTCGATCCTACGCATTCGTtA<br>AGGTTTTGAGATGGCATGGTTTAATGCGAAAAA<br>TGCATGGGGGAACTTCCCTGATTTGGCGGGAG<br>CCGTGAATAAGCTTCAGGAGAGTGTGAAGAGT<br>ATTGAGAAGAATTTTGATACTGCTCTTGGATTT<br>[illegible]	NA	NA
Ca-II-SNP190	208.66	Ca-*desi*-LG(Chr)1	13721651	TAACATTATAATGTTTTGTAAAATGAAAAATTATAT<br>TTGACATGTTGGCACATTCATCTTATTTTTAGCTTC<br>CCTATATGCGCTCACTCTCTCTTGTTCA	A	G	TTAGGAAATTCTAATGAGCAATCATCATCATCA<br>GCTCTTCAAAGTTCTCAAGGTGATATAGATCTG<br>CAATGTAAAGATAATGTGACCTCTCAAAGTAGA<br>AATCTTGAGATTGTTGGACAAAAAGACAATCTT<br>CCTGCTGATTCAACTCCTACTGCTGATGGAACC<br>CAAGGATTAGCATTCCTTGCTCAGGTCAGAGAA<br>AAACTCAGTGCTGCAGAATATATAGACTTTGTG<br>[illegible]	NA	NA
Ca-II-SNP191	209.42	Ca-*desi*-LG(Chr)1	14029689	GCACTGGCCCCATCTTCTTCCCAAATGCACTGGT<br>ATCGAATAGGTCTCAGCACACTTTCAATAGTTTTG<br>GTAGTCCCTTTGCCCAACCATCGTCAAGGTT	C	G	TGATAATTCAGTCTCCTAAGAAGATCTCGAACG<br>ATAAAGGGAAAACGTGCTCGTCCTTCGTGTAAA<br>GAGATGCAAAAAGTTGAGGAGGGGCAAGTGAT<br>CTGTTACATTGAACAGCTTGGTGGGGAGCTGC<br>CAATTGAGTGTGATGTGTCCGGGGAGGTTATC<br>AAGATTCTAAGAAAGGACGGGGATCCGGTTGG<br>TTATGGTGACGCACTTGTAGCAGTATTGCCGTC<br>[illegible]	NA	NA
Ca-II-SNP192	210.80	Ca-*desi*-LG(Chr)1	14135090	ACCATTTGAACACACGAGCAACTTCACTATCAGAT<br>CTACTATTGCTCTTCTCTTCTGCATTTGAATGCTTT<br>GCTCGATGACTCCAGTTTGTTGGGTAATA	G	A	GCCGGACCCAAAAGTTTTTAGAGCTTTGATAAA<br>GAATTTTCAATCTATAATATTGAAGGTTTTTCAA<br>ATATCAATCTTAAGAACCAAATTTCTACTAAAGC<br>TTATATTATAAAGCATATTAATAGCTTCAAATGG<br>TAATGATATTTATTCACAGCCTATCGGGAGAAG<br>AAAGAGGAGGACACACGAGCATCCTTCCAACA<br>CCCATAAATTCAGCAGCAAATTCTTCGTCATTTT<br>[illegible]	NA	NA
Ca-II-SNP193	1.08	Ca-*desi*-LG(Chr)2	75212	TTTGTGTATATAAATGGTATTTTAGCTGAGATAATT<br>TGTATAGTGCTTTGGCTGAGATGATTTGTACTGTG<br>TTTTGCAAAGATAATTTTGTTTTATTTTC	C	T	GCAACATGTTGACTGACACAAGCAAAAAACAGT<br>GTTTCACCTCAGTAGAAGGTAAACAGCATGGCA<br>TGACATAAAGTAAAACGTTTAAATCAAATATGGT	NA	NA
Ca-II-SNP194	2.09	Ca-*desi*-LG(Chr)2	89296	AAACCAGCCTCTCGCTTCCTGCATAAGCCTTTCCA<br>TAAGCAGTGATGTGGTCGGTGAGATGCAAACAGG<br>TGAATTACCACTTTGTGCGGCCGAAAGAGCG	A	G	AAAAAAACTCTTCAGCCACTGGTTTTAGGTAAT<br>TGGTTATTGATTATGGCTACTTATGAAGATTTTT<br>TTCGGTGGGAAGAGTATTTTCAGAACTCAAGTT	NA	NA

Marker IDs	Genetic positions (cM)	Chromosomes	Physical positions (bp)	Flanking sequences/Forward primers	ICC 12968	ICC4958	Flanking sequences/Reverse primers	Annealing temperature (0C)	Amplified Product size (bp)
Ca-II-SNP195	3.10	Ca-*desi*-LG(Chr)2	123972	AGGAACATTAAACACTTTACCCAGAATGTCCTTGCAACTAGCTTTGTTGCCCTCTTGATTTTCCGAACTTGGTTCGATATTCTCCTAAAATACATGTAGG	G	A	ACTTTAATATTTTTTAATATTATATTTTATCACTTTAATATTATCACTTTAATATTTTTAATATTGTATTTTAATAATGATATTGAACTTATCTTTTTAA	NA	NA
Ca-II-SNP196	4.11	Ca-*desi*-LG(Chr)2	208901	GTATCGTACTTACGTTTACTAGACTTAATATTGCTTATGTTGTTCATATTGTCGGTCAAAAATAATATTGATTTCAAGTACAAAAGATTCTACTTGTTTT	G	C	ATATCATAATATCCTACAATGTTGTTTGTGGTCTTGGAACTGCGCACTTAAATAAACATTAGTGTCTAAAATTGTTTCCACAAACTGATTTTATTATCAT	NA	NA
Ca-II-SNP197	6.13	Ca-*desi*-LG(Chr)2	232256	ACATGTCGTCCAAAGTAATCGTCATTTCACCAATAGGGAGATGAAAGCTACTGGTGTCACGATGCCACCTCTCTACGAATGCAGAAATGATCCCTTTGTC	A	G	GGAAAAAATTAAAAGAAGCTGTTATTGAAGACGAAGAGGTTGAGCAACTAGTTAAAAGCTCGGGATTGTATTCGTTACTAAAATGCAGCTATGAAATGAT	NA	NA
Ca-II-SNP198	7.14	Ca-*desi*-LG(Chr)2	249953	GTAGGGATTTTTCAGAGTTTTGTGTTGTTACTTTGTTTTAGTGAAGTGCTGAATTTTGGTGAGAAATCAACATGACATATGTAGAGTATTTCCGCCATAT	C	G	GAGTCTGAATTTGGTTCTGGTGTCTTCATGAAAAATGTAGCTATGGATCTTGGCTTTCATTTTCACTTGGTTTGACTCCAATTGGACATCTATAACTCCA	NA	NA
Ca-II-SNP199	8.15	Ca-*desi*-LG(Chr)2	315499	AAATTTTATTTGAGTCTTGGTATTTAGTTACTTCAAGTCTTAGAGGCTTTAACTTGTTTATTTCCTTGACTTTTGTTGGTATTTACATCTATCTGAAGAC	G	T	TTCCCCCTTGATAGCTAGCTTTTAAGGGAGGGTTCCCCAAGTTCTTAAATACTTATCACAAAGTTCATGAAATATTCATAATTAGCATTAAAAAGCCACA	NA	NA
Ca-II-SNP200	9.16	Ca-*desi*-LG(Chr)2	325842	AAGTCCAAGGGTTACCTCATGAGTGGCTTACAATCATCCATCAATAGCCGAGTGAGGACATTACACTCTAAGCTCTCGGCTTTAGTTCATACAGCTAGTC	A	G	CTCACTTCGAACAATTTGTTCCCTTCCCTTTCATTTCCAAAAACGACTATGTATTGTCTCACCTCCTACTTCCTTATCATTCATCTCCTCTTTATATCTC	NA	NA
Ca-II-SNP201	10.17	Ca-*desi*-LG(Chr)2	354586	CCTAATGAAGCTGGTGCGATGGACCTATGTTATGGACTTAAATCCTTAATTATATGTATGAAAACAATTGAACTATTCTGGCTTTGACCTATTGGTTATG	G	T	TCGACCCAACTTTAAATACGTCAAACTAAATTGGATTAAACCTACATTTTTTAGATGCAAATTATTTTAGTTTAAAAAAAAATGCGTAAAAGAGTGACTC	NA	NA
Ca-II-SNP202	14.21	Ca-*desi*-LG(Chr)2	371023	GACATGATTAATGATGTTCCCGGAAGTATATCCCTAGCATCTAAGAAATGAGCAGTGAAAAAGATGCTCTGTGTAGTTGTGTTGTGAACTGCATGTCATT	C	G	AACTATATAAATGTTAATCCCATTTTAAAATTAAAGCAGCAACTAGTAGCCTATAGTAGTGAAGTACATTACTACTGTAATATTATTTAATAGTGATAAT	NA	NA

Marker IDs	Genetic positions (cM)	Chromosomes	Physical positions (bp)	Flanking sequences/Forward primers	ICC 12968	ICC4958	Flanking sequences/Reverse primers	Annealing temperature (0C)	Amplified Product size (bp)
Ca-II-SNP203	15.22	Ca-*desi*-LG(Chr)2	392964	GCCTTACTGTTTGTATACCAATTGACATGATGGGTAATGTGACATTATGTAGCTCACTGTCATTTTAGGTACACTATTTGGGCTGGCACTCAGAACAAAA	A	C	TGTCTGGAGGACCCATAATTGTCGCTTGCCAATGAAACATATCTTCAGCAACAGGACCTGTAAAGTTTTGAAACAAAGACAAAACAAGATATCAGCACAA	NA	NA
Ca-II-SNP204	16.23	Ca-*desi*-LG(Chr)2	413593	GTGTGTGAATATATTAATCAAGTTTATGTGAAATGGTGAAGGGTTGAGTCACCTCTTGCAGGCGGTGATTGCGGTCGTTACCACTTGGTGGGTCGTGTGA	G	A	CCGACTCACTAACAATCTAACTAACCATGCTCCTATAAAGTTTAATTAACTATATTATTATATATTTGGAAGAAGAAAAATTGAGACCTCGATTATCGAA	NA	NA
Ca-II-SNP205	17.24	Ca-*desi*-LG(Chr)2	420492	TTATTTAGTTTATTAGTAGTTAATGTAAGACTTTAACCACTCACATTTGAACTTAACAATCTCTCCCTCAAGTGTGAGTCTTTCGCATCATATATTTGTA	C	T	GGTCTTATTGTCGTAACGTTTCCGTTTGATGGAGGAGCGGAAATGGATCATGATATTGGATCCTTGTATCAAAATACTTCTTGTCAGTGTTGTAACAACG	NA	NA
Ca-II-SNP206	18.22	Ca-*desi*-LG(Chr)2	429417	CATTACCTTTTGGTTGTCCATAAACTTCAGGTGAAGCAAGGCAGCTGGCTTCATGTACATCTCCAACAAAAGCAAGAGCTTCTTCAATGACTTTATATTT	C	T	CCATTCAGACTCAGCAGCGCGTTGAGAGAGATGCTGCAATTGCACGTTTGGAACAGAGTAGAATTGTTCTTGCAATGAGACTGGCAGAACACCGTGGAAA	NA	NA
Ca-II-SNP207	19.20	Ca-*desi*-LG(Chr)2	436660	AGTTAATCGGAACTAATTGCATACCCTCGACCATCCGGCCATATGATTGTTCTCCCGTCAGGTGCAACACCATGACTAGAATTGGAGCATCATGAGCCTT	G	T	ACTGCTACAAAAAAAACTTTTATGATTTGTTAACTGCTACAAAAAAACCCTTATATGAGGGGGCTTCTGATTCAAGGTTATCGCTACTGGTAATAAGACT	NA	NA
Ca-II-SNP208	20.18	Ca-*desi*-LG(Chr)2	441416	TTCGAGCTGAATCTAGTGCTTCTAGGACTTCTAATGCCATTTTGGAAAAATTAAGACTCTCAATTTGATTTATGGAATTGGTTTAGTTATTTATGTGTTT	G	C	AGCTAGCAAATCCCAATTTTTCTTGTTCACATCTCCTAAGATAACTTAGGATTAAACGTCCATTACAACATCAACATAGTTATAATAAGATATATAAGAG	NA	NA
Ca-II-SNP209	21.16	Ca-*desi*-LG(Chr)2	451398	CTTAATGTTCCACCTTTAGTCCAATATCTTCTACAAGATTTGCAGAAATATCTTGGTTGAGAAAGACTGTAATTGTTGTAGTAACAGAATTTGGTGTTGG	A	T	CGAATCATTGGAGAATATGTTGGCTTGTTCGAAAGCACAACAAGACAAAAAACCAAAACCTCAGCCAGAACAAGCTCTAAAATGTCCAAGATGCGACTCA	NA	NA
Ca-II-SNP210	22.14	Ca-*desi*-LG(Chr)2	493799	ACACAAGAGTTGATACACTCCTACAATGGTATAAACCACTTAATTTGTGCTCTTCCAAAATGGTATTTCAATTGGCTCAATGCTTTGTCTCTGTTCATCA	G	A	GGAGCTGTCAAGTGCACTCCGTTTGAAACAGTTTACGGCAGACCCTGCCATCATTAACAAGGTTTGTACCTAGGGAAGCTTAGAAGCAGTGGTACAAGAC	NA	NA

Marker IDs	Genetic positions (cM)	Chromosomes	Physical positions (bp)	Flanking sequences/Forward primers	ICC 12968	ICC4958	Flanking sequences/Reverse primers	Annealing temperature (0C)	Amplified Product size (bp)
Ca-II-SNP211	23.12	Ca-*desi*-LG(Chr)2	519934	TCTTATTGGATTGTAGGATCTAGTTTTATAGTGAGATAGAAGGCACAATGTCCTTTTCTTCGTTCAATCTATTTTTCAAATAAAAAATTCTCTAAACCTC	C	T	ATAAAAAATAAAAATAAAAAAAATTTACCCTTAGATGATTATGAGCCGATGAATTGAAAAGTGAAGTAAGAAATCAAATTGAATTGAATTTGAAAGAAAT	NA	NA
Ca-II-SNP212	24.10	Ca-*desi*-LG(Chr)2	539372	CGATATATGTCATCAAGTGTATTGGTGTGTTAGTCACGGGATTTAGTTGCATTATATGTCAGTAACGACCGACGCAGTGGTCCCATTATTGATGTATGTG	G	A	TTTTCACATGGGTAATATATTTTAATAATTATATCAGAATTCTTTAGACGCACATCTGAATGCTATCTATTTTTAGATTTCTCCTTTACAATCTGGTCCA	NA	NA
Ca-II-SNP213	25.08	Ca-*desi*-LG(Chr)2	604529	AAAGCTTCTTAAAGATTTGCAGGATCCTCTTCTAAAGTATAGGTCGCATATTTTGATCCATAATTTTTAGCAACTCTGACTCTCTTACTTCTTCAAGTTT	C	A	TGACTTCTATGAAAATAAATTCCCTTTCAAATGGAGAAATAGTGGGGGCAGCGAATCAAGTCACGTTCCTATATCTATAAGCACCAAACAAGGCGAATCA	NA	NA
Ca-II-SNP214	26.06	Ca-*desi*-LG(Chr)2	616399	TATGTTATATGAATATTTTTTTTTAAAGCCTTTTACAACGCTTTTTCAAATAAGCACTCTCAAAGGCCTCCTTATTTTATTTTTAAAAGGGTCTTTTACA	A	G	CTGAACTGAATATAAATTAGTAATTTACATGAATTGAAAAAGAGCCTTAAATATAAAAATAAAACAATGAGGTTTTTTACAGCGCTTATTTGAAAAAGGG	NA	NA
Ca-II-SNP215	27.04	Ca-*desi*-LG(Chr)2	642761	TTCCTGATAGTGCAATTAACATTGATGGTGATCTCCTAAATATAGTATTATTAGCAGAAGCTGAACCTGTGTCGCTAGAGGAAGCACTGGCTCACCCTCA	T	C	ACTTCACTTCTATAGCCTTCTTATTTCTTGGCAAGCTTACCATTTCCCAGGTTTTGTTCTTGTGAATTGATCTCAATTCCTCATCCATAGCATCCTTCCA	NA	NA
Ca-II-SNP216	28.02	Ca-*desi*-LG(Chr)2	651196	TTTAACAAGCGACTACATCACCGCGATTAATTCCGGTAAAATTTCCGCTTTCGACGTCGCTTCCGAAGTTGGCTCACTAGCACCGTAGCAGGGAGATGAG	A	C	AAGAAAAACGGTATTAGGGGTTTTTAAAACCGTCAAACACAAACCTCCACTTTTCTTCGACGTTCGAGCTTCGTTTCGAGAAGTGATAGAAGGGAAAGTT	NA	NA
Ca-II-SNP217	29.00	Ca-*desi*-LG(Chr)2	741960	ATTCTCATTTTATTTTATTCTATTTTGTACAGATTTTTTTATTTTTCCTTCAGATTTTGTGCAAATATTACTTTAAATAAATTTTAATTTTTTATGATGT	C	T	AAAATCTAGTAAAAAGAATTTAAACTCGTGTTTAATCGAATATTTAATTGAAGTTCATTAAAATTTTGAATTCAAATATTTATTTAATTAAAATTAAGAG	NA	NA
Ca-II-SNP218	29.98	Ca-*desi*-LG(Chr)2	794468	TGCAACCTCTAATGTTGCACTGACATTGATTATTAAACAATGTGATTTGCCCAAAGCCACTAATTCTAAGTCATGCAATTCTGCCTTCGACTTATATTAT	C	T	GGTTAAATCTTGTTTCCATAATTAATTTTTTCAATCAAAGCTTTTTACTTTCCATTAGAGTCATTGACTTTCTCGTTTGAGTGAGATGTTGTGTTTTTTG	NA	NA

Marker IDs	Genetic positions (cM)	Chromosomes	Physical positions (bp)	Flanking sequences/Forward primers	ICC 12968	ICC4958	Flanking sequences/Reverse primers	Annealing temperature (0C)	Amplified Product size (bp)
Ca-II-SNP219	30.96	Ca-*desi*-LG(Chr)2	824124	GAGTTTGAGGAAGGAGATCATGTTTTTTTGAGAGTTACCCCCACCATTGGCATTGGAAGAGTGCTAAAGGTCAAGAAGTTAAACCCTCGTTTTGTTGGAC	C	T	CTTTAACTGGGAGACGTGAAAAACATTGTGTAGATTGGACAGTTTGGGTGGCAAGGCTATTCTGTATGCAGAGGGACCAATCCTCTGCAATATTTGAAAG	NA	NA
Ca-II-SNP220	31.94	Ca-*desi*-LG(Chr)2	851921	CTATTAAAACGGGCTACCCAACCCATATAAGGTCGGTCCTAATGGGTTGCACGCTTCTTAGGATCATGCCAAAAAACCCTGTTTTAATATGGGCTGATAA	A	T	AAGAAATTAAAAATATAACGAGTCAAAAAAAAATATTGTAAAAAATGTGCAGGTTACCCGCTCAATTTGAAACTCGCCTAGGACCAGACACGATTAGTAT	NA	NA
Ca-II-SNP221	32.92	Ca-*desi*-LG(Chr)2	865647	CAAGACACTAGCTTTTATCAAGATTCGTAATCTTATGCTCACAATAATATTTATTTTTTTCAGTTTTAGAGACTTTCAATTTTTATTGTTTGGTTTCCTT	G	A	AAAGAAAAACAAGTAGTGAATTTGTTGTTATTTTGACTACCAACAAATTCTTAAGTAATGTCATAACTTATAAATATTTTTATATACAAAAATAAGATTG	NA	NA
Ca-II-SNP222	33.90	Ca-*desi*-LG(Chr)2	874315	TAGTCAATATATGTAATTACTTGTTAATTTTGTTTGAAGAATGAGTTGAATTCATAACTTTGCTGTCACAAGACTTGAGATAGATTAGTCTTTGTAGTTG	T	C	GAGAGACCTGACTATTAAAATTAATTTGGTTCATTAACATATCAGATTGTAAGAAATATTTTAGTTGAATTTTTTAGTGTAAGTTGGGTATTTTTCATAC	NA	NA
Ca-II-SNP223	34.88	Ca-*desi*-LG(Chr)2	886887	AACAAAACCCGTAGAGCTTGACAATGTTACGATGTTGAATTTCTATCAGAGTTTTGATCTCATTTGTGAAAGCTTTCATAGTAGACATTTCTTCATTTGG	C	T	TTGATGACAAATATCTCATTGGAGTTGGAGGGAATGGAAGTGTTTACAAAGCAGACTTACCCACGGGTCAAGTTGTTGCTGTCAAAAAAATGCATTCAGT	NA	NA
Ca-II-SNP224	35.73	Ca-*desi*-LG(Chr)2	909741	GTATCATTGAATGGTTTTTCTTCACATAAATAAAACACGGTACTTGTCAGGTCCGCACGGTTCTGTTTTTGTGTTTAAATTGTTTGATTGTATACAAACA	A	G	AAATTAAGGACCTCTTATACTAAAACTATTTCAAACAAATATACAAGCACTTATGTCATAATACGAGTTCAAATGAGCTATGAATAAGCTCTTTAAAAAG	NA	NA
Ca-II-SNP225	36.58	Ca-*desi*-LG(Chr)2	922816	GGAGTCTATAATACGTGTGGAAAGTCATGTGTTGAGTCGTGGTTATGGGTTATTATGTATGGGTGTCATGTAAGCATCTGTGATTAATTATTAATATAAT	G	A	GAATTTGATGCATTAAAACGATAAGAACAGAACACAAGATATTTGAAATATCTCAGTGAACTCTTAGGCTTACCAAACAAGTTTACGCACAATATTTAAA	NA	NA
Ca-II-SNP226	37.43	Ca-*desi*-LG(Chr)2	1081581	AAAGTTACCTCTCTCTGTGACTCAGCTGACACACCCTGTCCTTGTGTAGAAACTGAGACCAGTCCTGAACCCATACCATACTGCAGGGGGCCGCTGCGGG	T	C	CTCATTGACCGGTTCACACTCACATGATCTTGGATCATTCGAAAATGGATTCATTGGTGGAACTGAAGACAAGTTGCTTGCTCCACAGACCTCATTCAAG	NA	NA

Marker IDs	Genetic positions (cM)	Chromosomes	Physical positions (bp)	Flanking sequences/Forward primers	ICC 12968	ICC4958	Flanking sequences/Reverse primers	Annealing temperature (0C)	Amplified Product size (bp)
Ca-II-SNP227	38.28	Ca-*desi*-LG(Chr)2	1100915	CCTCTTGACTCATTTATAAGCAAAATTAGCTACTTTCACACTTATTAATGATGATAGCCAAGTGCATTTAGGGTTTTTTTGGATTAACTTATTTGAGCTT	A	T	GGATAGCTTATGAAAATTAGCTGAAAACACCTTATTGACATGCCATAAGCTGTTTCCATAAGTTTCCCCAAACAGTTTCGCAAGTGTTTATGTTGGTAGA	NA	NA
Ca-II-SNP228	39.13	Ca-*desi*-LG(Chr)2	1122632	TGAGGGAACAAATGGGTTTGTTGTAGATGTTGATGCTAATGTTGGTATGGTGAGTTTTGCTGCTGCTGCTATGGGGTTCCGTGTTTTGGCGTTTGAGCCT	A	G	ACTTCATCACCAACAACAACAACACTACCAACATTAGCACCAACATCCCCAACTCTATTAAAGTAAATTCCTTCACAAATCTTCTGCAAATTCTCAAAAA	NA	NA
Ca-II-SNP229	39.98	Ca-*desi*-LG(Chr)2	1156903	CTTTTGATGATATCACGCTTTATACTGGTCACATTCGATTATGCAGCACTGTAGTGAAATATCTACCAGAGAGGTGTTTGAGACAATTCGGATATATTCA	A	G	ACTTGTGAAATAGCTCTGTCACTGGCATCACATATCCCAACCATTCCACATCTACATCAAATAATCTAGCATGATCTGGAAGTGGCGGTGAAGGAATATA	NA	NA
Ca-II-SNP230	40.83	Ca-*desi*-LG(Chr)2	1181100	CATATTTTTTAATATTTATTTATAATAAATATTTTTTTGTATTTTTTTTAATATTTATAAGTGACAAATATTTGTATTGTGTTTTTTTATGTATTAGTGA	A	T	ATTAAAAAAATATGATTAGTAAATATCTATAATTGATGCATGAAAAGCGCATGACAAATATTCAATAATAATAAATATAAAAAAATTACGAGACAAATATTT	NA	NA
Ca-II-SNP231	41.68	Ca-*desi*-LG(Chr)2	1245390	CGACGACGACGGCGCTGCGGTCGTGGTGATGGCGGCGTGCAAGAAAGTGAGAGTCGATAGAGAAGAGGGACAGCGGCTGATATGGTTGCAGAGAACAATC	G	C	AAACTTTTAAAACAAATTTGAACTGGGCTGGGCCACACACGTGCTACAATGGGAAGCAAGGCTGTAAGGCGGAGCGAATCCGGAAAGATTGCCTCAGTTC	NA	NA
Ca-II-SNP232	42.53	Ca-*desi*-LG(Chr)2	1272206	TCTACTTCATAGTGTGGTACCATTATAACTCTAAAGTACTTGGTTAGGAAGTGTGATACTATGTCACCATCCCGGTGAACAGACAATTCAAATTGTCCAT	G	A	GGAGGTCGCAATCCAAATGTCGGGAGCTATCATGCAACACACGTCTACCTCGATTGTCGCGTATATGTGTCTCGGGTTGAGTTAAGGAGATCTATGAGGA	NA	NA
Ca-II-SNP233	43.38	Ca-*desi*-LG(Chr)2	1288366	ACAGTGAAAGGGGTGGGATGGGATGTTTCTGCTATTGGAAATGGTTAGTATTCTTGTCTGCATTATTGTTCAAGAACCGTAAGACTGAAGGAGCTTCATG	C	T	GGATAAATTGGTGCTTTTTTTTTGTTTCCAATTCAAAATAATAAAATTGTAATTTTCATTAGTTCCATCTCATTTCATTGACTACTAGATATCCAAATACA	NA	NA
Ca-II-SNP234	44.23	Ca-*desi*-LG(Chr)2	1424536	TCGGTTATCGATAATTGTCCCAAAAAATATTCTCCATTTTCTTTCAAATTTCAGTATGTTGTTTCACTCATTTAATTATTCAATTAAACACACTTTCACG	A	G	ACTCAAATTAGACTTACGATGAAGAATAACGGTGTGCAATAACGAATTCCATAAACTTATAAGTTGAGTATTTTATAGAGAAATTGAGATTGTTTAAAAT	NA	NA

Marker IDs	Genetic positions (cM)	Chromosomes	Physical positions (bp)	Flanking sequences/Forward primers	ICC 12968	ICC4958	Flanking sequences/Reverse primers	Annealing temperature (0C)	Amplified Product size (bp)
Ca-II-SNP235	45.08	Ca-*desi*-LG(Chr)2	1432725	TATGTTTATGGAACAAGAATTGTTGTCCCCCTCCTCCCGCTTCCATAAAATTTTAAATTTTTTTTCTCTTTGGTTAGTTTACAATAATACTCATGAGTTT	G	C	AAAGTATTATGTAAAGTTCAAAAACAAAACAAAAAACAACAACAATCGTTTATTTATTTATTTTTATAATTCAATTATTACATATAAAATGAGTCTTTTT	NA	NA
Ca-II-SNP236	45.93	Ca-*desi*-LG(Chr)2	1450638	GTTACATTGTGGTATTAGAGCACTATTTCTCGGACCTTGGTGGGATGGGACGCTTCGTGTATGTCGCATGTTTTTCTTGTGATATGTTATTCATGTTAAT	T	C	TGTAATTCTTAAAAATTCAAATTCAAGAAGGTTACATAATTAATGACATATAATGGACATTTAAATAATTAGTAAATCTCAAATAATTAGAAAATTATAC	NA	NA
Ca-II-SNP237	46.78	Ca-*desi*-LG(Chr)2	1506588	TTTTAAAAGTTTGATGTGTGAGAAAGATTTAATATTGGGAATTTCGTTGTGGTTGGATTAGTTTTATTGTGAACAAATTGTTATAAATATTTATGTTTAT	A	G	CCATAAATCACCACAACAACATTAATATATCAGAGTTCTCCCCATCACTAACTCGGTGCTAATGGTCTCGATTCCTTTTCGAGACTCAATGAGCTAACTA	NA	NA
Ca-II-SNP238	47.63	Ca-*desi*-LG(Chr)2	1649749	GAAGAGGCGTCATCAATTGCATTCACTTCTTCCTTGCATAGCTTCGTAGACATCCTATAGATAGAAGTGAAACGTTGCTCCTTTGCAACCATTAGAGGTC	C	A	ATGTTCTTGATATTCGTCTCAAGTTGATTTTATTGAAAGCTTTGGATATTAAGGGTTATCAACTTACTTTGGTAGTGGTGGTATATGTAAAATCACTAAA	NA	NA
Ca-II-SNP239	48.48	Ca-*desi*-LG(Chr)2	1673041	CGACGACGACGGCGCTGCGGTCGTGGTGATGGCGGCGTGCAAGAAAGTGAGAGTCGATAGAGAAGAGGGACAGCGGCTGATATGGTTGCAGAGAACAATC	T	C	GTAGATAAGGTAGAATAGTTGCAACGGTTGTGTTGTGTTGTGTTTGTGATAATGGCTTCTTCCACATCAATTCATTGCTCTGTTTCACTTCCTTCACTTCCCAAACTCACCAATTTCAAAACCAATAATTTCTTCTCTTTCAATCCAACAAACCCTAACTCCATTCTCTCATCATCCCTCTCCACCGTCAGATCCTCCGCTTCTTCCTCTTTCTCCTCAACAGCATTCGAAGA[illegible]	NA	NA
Ca-II-SNP240	49.33	Ca-*desi*-LG(Chr)2	1781378	GATTATCTCATTTGTTGTAGTTTTATTCGTAATATATTTGTTTGTAGTTTTGTTTGTAATATATAATTGACCCCCACAATGGTAAATTATTCTCTAAATT	C	A	TTGTATATATTTACTTTATATTCTACTTTACAAAATATTGATTTTTAGTCTAAAAACAAAGATTTTTTTTGCTTATAGTGTATTTTTCTCTTCAAATTTA	NA	NA
Ca-II-SNP241	50.18	Ca-*desi*-LG(Chr)2	1801000	CTGGACTATCACCTTCTTTTCCTAATCAAGTATGTAAACTTGTTAAATCTCTTTATGGCCTGAAGCAGGTTAGTAGACACTGGCATGCAAAACTTTTATA	T	G	CATCAACATATATGGCCAAGGCATTAAACGACTTGCTAGTATGTTTAATCAATAATAAATGGTCTGGTGTGGCTTGCTTATAACCAATAATTAATGAAAC	NA	NA
Ca-II-SNP242	51.03	Ca-*desi*-LG(Chr)2	1818420	TCAAGTCATTTAAAGTTTTTTTTCTCGATTAGGTTTGGTAAGTTATATTTAGTTTACATTTTAGTTTTTTGTCTCACTTTCACCGAGGATTAATGCATTT	G	A	TGTAGATTGTTTATAATGTTTAATGAATGTTTTTTGAATAATTAATTTGAGGAACATATTGTTGCAGCCTTTGACAGAGTTTCTAATAACCACATGCTTT	NA	NA

Marker IDs	Genetic positions (cM)	Chromosomes	Physical positions (bp)	Flanking sequences/Forward primers	ICC 12968	ICC4958	Flanking sequences/Reverse primers	Annealing temperature (0C)	Amplified Product size (bp)
Ca-II-SNP243	51.88	Ca-*desi*-LG(Chr)2	1854096	TAAGCCTGCATTTCTGTAATATATGTATGTATTGTGTATTATGTTTATTACTCCCTAGTTAACTCGACAGCTTAACACGAATAACGCGCGAGCTTTCCTC	A	G	AAATGAGGTATTTTTCTTTTGGGAAGCGAAGCGACCTAGTCGAGCTATTGTCAAGTTAAGCAGTTCCTCTTGTCGAGCTAGACTCTACTCGCACTATAGT	NA	NA
Ca-II-SNP244	52.73	Ca-*desi*-LG(Chr)2	1918740	TCATGGATCTCATGGAAGAATGGTTGGAAAAGTTGGATCAAACGAATAGCGTTTTTCGCGTTTCGTTTGTTGGAAGAGAAGAATTTGTGTTTGAAATTGT	T	C	CTTCCTCTTTCGCGCCTCTGCAACCACTGTAGAACCAACTAGAGATGAATACACTTGTCGGAGAAGAAAACGCCGACGGCGAAGCCAGCACAAGAAGAATCCAAGTCCGTTTCATTACTAAGTTATCCGAACCTTTCAAAGTTCCATCCTCAGCCATCGCCATCCCCGCTGATCTCACCAGATTCGGCCTCTCATCTCTCGTTAACGCCCTTCTCCAATCCAAATATGAT[illegible]	NA	NA
Ca-II-SNP245	53.58	Ca-*desi*-LG(Chr)2	2170610	TGCTGATCTATGAGAGATTGCACCTTGGTAACTAGTGTTGGTCTGTGAGAGGCTGCACTCTAGAAAATCGTGTTGATTTGTGAGCGATTGCACGCTAGTA	T	C	GCTTTCTTATGTGACAAAACTTTATTGAATAAGCACTTATTTAAATTGTTTATTCAAGTACACCTCATATCACACCGCACACAACACGAGAATTCTATCACATGGATTGAATTCCAAATCAGTGATTAGCCTAATCTACTTCCCAAATTCCATTTTATTTATGATCAGATTCAATCTTTAGATTCATCATCTTCTTTCCATTCTACTATTGCCAAAGTATGGCGGCCGCCACAA[illegible]	NA	NA
Ca-II-SNP246	54.43	Ca-*desi*-LG(Chr)2	2325857	GCAGAATCTACAGGTCTCCTCCCGACATGCCCAGACCACTTAAGCTGAGTTTCTACCATCTTTTCTACTATAGGTGCTACCTCAACTCTCTCTCTAATGT	T	A	TAATTAATTGGTTAAACATTAGGATAAAATAAGTGTAGCAGAGTTGCGAATGTTGTATTGGATGTGTGGAAAGACTAGACAAGATAGGATTAAGAATGAC	NA	NA
Ca-II-SNP247	55.28	Ca-*desi*-LG(Chr)2	2366447	CGAGATTTTTACAATTTTACAATGTACGTGAACTCAGTTCAAGTATGTTTACGTGAACTCAGTTCAAGTATGTTTACGTGAACTCAGTCCACGTACACCA	A	T	TGTAATATTACGTGAATTGAATTCACGTATATTGTAAAATTGTAAAAATCACAGGTAGTACGTGATTTAAGATTGCGTAATTAATGGAGTTTGAGACTTA	NA	NA
Ca-II-SNP248	56.13	Ca-*desi*-LG(Chr)2	2470350	TTCCAATCGATTGTTCAGTATGAGTTTGGTTCTGTGAATCACAAAATCGATTACTCAATTGATGTGCAAATCGATTGTCCAATTGATTTGTTTAAACACA	T	G	ATCGATTGAACAATCGATTGCCAGACTCACAGAATGAAGGTTTCACTAAGTTAATCGATTGGGAAATCGATTACTTAAAGGTTTTATCAAAACCTATGTT	NA	NA
Ca-II-SNP249	56.98	Ca-*desi*-LG(Chr)2	2502854	TTTGCTCGAACCGACCTATCAGCTCAAGCTTCCGTTCAGATTTTTTTCTGGGCTTTCCCCACATACCACTGCTTTTTGTCAAATTTCTTTCCATTCAATG	C	T	CATTACTAGTGCTGTTTTGATGAGAGATGCAGACGGTAGGTCAAAGTGTTTTGGTTTTGTCAATTTCGAAAATCCAGACAATGCTGCTAATGCTGTGGAG	NA	NA
Ca-II-SNP250	57.83	Ca-*desi*-LG(Chr)2	2576058	TGTTGGCATCCATTTAAATCTTATCCAAATATGCCTTTAATTTGTCAGGAGTTTTGTTTGGAGATTAACACTTGACATTACTTAATCTGTGTCTTCTTTG	T	C	TTCTGTATAATTAAGAATGCCAAATTTAGTTATTAACATCTATAAACACATACTAATATGAAACCACATTTGACAGAAACACATGCTCAAAAACAGAAGG	NA	NA

Marker IDs	Genetic positions (cM)	Chromosomes	Physical positions (bp)	Flanking sequences/Forward primers	ICC 12968	ICC4958	Flanking sequences/Reverse primers	Annealing temperature (0C)	Amplified Product size (bp)
Ca-II-SNP251	58.68	Ca-*desi*-LG(Chr)2	2705575	GCCACTTTTTCCCACAGCCACATTCTTTTCTTCTATGTGCCGTACCTTGTTTGAGGTTCATACAGCCAAGCCTTTACATTGATTTCCTCTTTCAACCCAT	G	T	CAAACAAGACAACTCTGTTCAAGAATTTCTCACAAAATTTGAGAAATATGCAGGATCTTTCAAGGGGATTGATGAACATTATTTGATAGAGATCTTTATA	NA	NA
Ca-II-SNP252	59.53	Ca-*desi*-LG(Chr)2	2765925	TCATGGATCTCATGGAAGAATGGTTGGAAAAGTTGGATCAAACGAATAGCGTTTTTCGCGTTTCGTTTGTTGGAAGAGAAGAATTTGTGTTTGAAATTGT	C	A	GTCGGGTCGTCGGATATTAACATTTCCGGCGGTTCATCCCTGCGTGAACATTGCTCCTTCCACTCTTCTCTCTTCCCTCATCACTCTCTCCGACAGAATT	NA	NA
Ca-II-SNP253	60.38	Ca-*desi*-LG(Chr)2	2803005	TGCTGATCTATGAGAGATTGCACCTTGGTAACTAGTGTTGGTCTGTGAGAGGCTGCACTCTAGAAAATCGTGTTGATTTGTGAGCGATTGCACGCTAGTA	T	A	ACTAGCGTGCAATTGCTCACAGATCAGCACATTCTAGAGTGCAGCCTCTCATAGACCAACACTAATTACCAAGGTGCAATCTCTCACATATCAGCACGAT	NA	NA
Ca-II-SNP254	61.23	Ca-*desi*-LG(Chr)2	2848860	ATTTGATCTTATCTATTGGCATAAATACTTGTGGGACTGTTTTGAAGAGTTTGTGAAAATAACTTATGACATTTCCATAAACTGCTTACAACTTATTTTC	A	T	CTTATGTATAAGTTATTCCTATTAGTAAAGATAAAAATTATAGTCAATATTTTTTCATATAAACTAAAAGTTGTTTTCATAAGCTATTCTAGAGAGCTTA	NA	NA
Ca-II-SNP255	62.08	Ca-*desi*-LG(Chr)2	2883759	TTAAGCTGGTTTCAATGGTGGGAGAGGTGCAATCCAAATCCTTCATGGGATGCTTTCAAGGTGGCTGTCATAAGGAGATTTCAACCATCAATGATTCAAA	G	A	TTGATCAATTTCTCTTAGAGCGCCAACATACTTCTCAAATTCCTCCACATACTCTTCCACTGTACCAACCTGTTTCAAAGATAGCAACAATTCAAAAGGA	NA	NA
Ca-II-SSR58	62.93	Ca-*desi*-LG(Chr)2	2952192	CAATGTGCTAGATTCGCAGTG	(AT)8	(AT)6	AGGGAAGTTGGATTCCATGC	59.50	116.00
Ca-II-SNP256	64.63	Ca-*desi*-LG(Chr)2	3195855	TTGGTAACCTTCCCCTTTCTTGTTAGTCTTTTTAATCGAATAAAATAAATAAATGTATACTTGGTGTGTCCATGTTTTTGCATTGTATTTACATTTTTAG	T	A	TCATTCTATGTTTCAATCCTTTCTACTGTATAGAAATCTTCCAAATAATTAAACTAGTTTCTACATAATCAAAAATATACAAGATAGTTCTCCATACTAC	NA	NA
Ca-II-SNP257	65.48	Ca-*desi*-LG(Chr)2	3220112	CGCTTACTGATCTACGTGTATCAGGACAGGATGCCCAGTCTGAGTCAGAAAAGGCCTTCAGTTGGAGGGCAGAATTTGCAGACAGAAAAATGCCTAATGT	A	C	ATCAGTTTTTTGTAGAACAACTCAGCCAACATATGAATTCTCCTTCAATCACTCACTACGAGGCAGCTATAAGAGTATTGAAATATCTCAAATCTTCTCC	NA	NA

Marker IDs	Genetic positions (cM)	Chromosomes	Physical positions (bp)	Flanking sequences/Forward primers	ICC 12968	ICC4958	Flanking sequences/Reverse primers	Annealing temperature (0C)	Amplified Product size (bp)
Ca-II-SNP258	66.33	Ca-*desi*-LG(Chr)2	3229500	AAGCCACATCTACAGCCTCACATCGTTGCCGCCG TCACTATCACCGTCTTCACCATCGTCGTCTCACCC TACCCTTCACCATCGCCGCCGTCTTGCCCAT	C	T	CTTTGAAGAGTTAATATGTTTTTTGAGCCGTTTT TCATAAATAAATAAAGTTTCTAATGGATCAAATC CAAATACAAAAGAGTAACTTCGACGAGACCCA	NA	NA
Ca-II-SNP259	67.18	Ca-*desi*-LG(Chr)2	3268614	TAGACTCAAATCACTCTCGTAATTTTTTCATATTTT TTTGTCTCTATTATTTCTCTTCTACTCTCTTTCCAT TATACGAATAAGATTATATATTTTTTTC	A	G	ATTATTTAAAATTATCTTTGTAATAATTCTTAAAA ATATCATTTTGACATGTGTTAAGAGTAAAAACAT AACTACATATTTTTATAGAAATGAGGAGAAA	NA	NA
Ca-II-SNP260	68.03	Ca-*desi*-LG(Chr)2	3293949	ACCTATTATAGAAGATTCATTGATCACTTCCTTTAC CATACTAGAATCAGTCCATATTGTAGTTTTTAGCT CAACAGTTGAGCCAGTTCATGCAAGCTC	C	A	TAGAACAAAAGACTAAGGCCAAGGATAGAGAAT GTCTTGTACTAGTGCAGATTTGATATACTTCAA CACACGAGTAGCTGCAGAAAATTCGTCTTCGGT G	NA	NA
Ca-II-SNP261	68.88	Ca-*desi*-LG(Chr)2	3356963	AAGATTATAACATTTTATGGTGGGTGGTGACACCT GATAGGCTCCAACCACTGTCAGAGGCTCAATGGT GGAAAACTTGTTATTTGACTATTTATTTCAG	G	A	CCAAAATATTTTTATATTATACCATAACCTTTAA AAATTTACATTGATACCCTTAATTTTGTATATTA AATACCTTAAAATTTTATATTTGCATCTTATA	NA	NA
Ca-II-SNP262	69.73	Ca-*desi*-LG(Chr)2	3404662	AATTTGGCGATTTTCTTTCCTTTCTTTTTCTTTCCT TAGTTTTTCCTTTCTTCCTTTTTCCTTCCTTCCTTT TATACTATTTTCTTTTCCTTTTCCTTCC	T	C	ACGGTAATTTAACAAATATCTAGATATTTGGAG ATATTTGTTAAGTTATATTTATTATCTATATATGT TTGGTTGGAGGGAAAAAGAAAGGAAAACTAGA	NA	NA
Ca-II-SNP263	70.61	Ca-*desi*-LG(Chr)2	3423711	GACAAGTTTTATATTTATTTTTTATAGAAGTAACCA GTCATTTTGTGCTTATTTTGCTAAGTTCTCTTATTT ATTTCTTTCACATTTTATCGGAGCAATT	C	A	ATAAAAGGAAAGAAGAGATTAGAAGGAAACTCA TATGAGATATTTTTATTTTGAATATAATTGGAGG TTGAACTACATCCAAAACACATAAGAGAATGCA	NA	NA
Ca-II-SNP264	71.49	Ca-*desi*-LG(Chr)2	3435770	AATATGGCCGCAACTCGGATCTATCGAGTTATATG TCACATTTGAGGATGGTAATCCATTATACGATATT GGTCAACCATCATCCTCAACCACACCTCAC	C	T	TTGGCCTGATCAAAGTATGGTTCGGGTTGGTAT GGGTCGGGCTGGTAAGATTCATAATTTTGAAAA GAATAAGTATTTTGTGTTTCAAAATGGTATTTAG	NA	NA
Ca-II-SNP265	72.37	Ca-*desi*-LG(Chr)2	3524491	AGTTTGTTCTCTTCTGCAATGTGATATGATCCTAA TAGTCATAGTTCACTTTAAAGATCGTTCTAGGTCG GAAACTTACAAATTTGAGTGAAAATCGCGT	A	T	TTCAAAAGCCTAACTTGCGTTTCAGAACGTCAT CTTTCACCTCGATTTGGAATCGGCCGAATCGG AGTTTCGCAGTGCCGTTTATCACGTTCTCCAGC CA	NA	NA

Marker IDs	Genetic positions (cM)	Chromosomes	Physical positions (bp)	Flanking sequences/Forward primers	ICC 12968	ICC4958	Flanking sequences/Reverse primers	Annealing temperature (0C)	Amplified Product size (bp)
Ca-II-SNP266	73.25	Ca-*desi*-LG(Chr)2	3559005	ACTGAAGGTTTATATGTCTGTCATTGACGAAATGTAATGCGGAGTCGGTATTTATGCAGGTGTATGTACATGTTATGGTGGTGGATCATAATATAGAATG	T	C	TCCTCATGTTCACTCAACCCCTAATCAACCTCACAAGAACAAACCATGCACCCAACGACATAATCGTCTTCATAATCGCCTCACCTACATATTGAACCGT	NA	NA
Ca-II-SSR59	74.13	Ca-*desi*-LG(Chr)2	3592267	TGTGTCCCCAATAGACATTTTG	(CT)6	(CT)7	TCAAAAGCTGTGCAGTGGAG	59.72	179.00
Ca-II-SNP267	75.89	Ca-*desi*-LG(Chr)2	3610564	TGATAATACACAGTTTGTAATCATCCTCGGGTTGAGGATCGATCTGTTTGAAAGTTAGTGAAATCTCACAAGGGTGTGAGGACTGGACGTAGCCATCTTT	A	G	CTCGCTGAGGATGAAATTTTTCAAATCGTTAGATCTAAGTTGAGCAAAAGAGTAGGAGCAGAATATGAGAGACAGACACACAATTTTATACTGGTTCACC	NA	NA
Ca-II-SNP268	76.77	Ca-*desi*-LG(Chr)2	3641530	TTATTCATATGTTCATACACTTTTTTTTTTTGCTTTAGTATATTTATTTATCATTTTTTTCATGAATACTTAAGGTACATTAATTAATTTTAAAATGCGT	C	T	TTAAAATGATTTTTAATCTATTTGTATACAATTTTATAAAAGTGATTTTTAAATTACAAAATTACCATAAAAGACATGACTTTTTTAATTATTATTTTTA	NA	NA
Ca-II-SSR60	79.41	Ca-*desi*-LG(Chr)2	3785925	TTGATGAGTTAAGGAAATCTCAAAAT	(AT)9	(AT)10	ATGTTTTAAGGAATAGATGATGTTTTT	58.73	148.00
Ca-II-SNP269	81.17	Ca-*desi*-LG(Chr)2	3799571	TGCTTTGCATTTCACTTCAATTTAAATTACAATTACAATTGCAATTTCTCTCTCATTTAGCGCACCTTTTGATCTATTTAAAGCTTGCTTCGCAACATGA	C	A	TTTTTTTTTTGTTCTAAATTTAGTGAAAGCTTGATTAACTCAATTTCATTTTAGGTGTTTAAAATGTATAGAAAAATGTAAACCATATCCAAATACATGTTTGTCCTCAAAAAAGCTATCGGCAGAACCAAGTAGTCTCCTTATCACTTGCTCTTCTTTGGTTCTTTTGTATATTGAGCTATCAGAGCTTCGACGTCCTTCCAGAGGAGGCCCTCAACTAAGACTCCATCCTTA[illegible]	NA	NA
Ca-II-SSR61	84.69	Ca-*desi*-LG(Chr)2	3978441	CGATTCACACCTTGTGTTCA	(TC)10	(TC)11	AGGCTCTGAAATGAATGATGC	58.08	146.00
Ca-II-SNP270	85.57	Ca-*desi*-LG(Chr)2	3999295	GATGATTGACTCTCCTTTCTATTTAACTGCCTTTAAACCTTATATTATGTTTACTATGTATGTGCAAGATTTCAGTTTGACCATAAAGAATCTACTTTAA	T	C	CAGCATCACAGAAACCTACTAACCTGTACTCAAGGATGACTTATAACACATGTCCAAATTAGTAGTTTGTCTTAGATATCTAAAGATCCTTTTAACAATA	NA	NA

Marker IDs	Genetic positions (cM)	Chromosomes	Physical positions (bp)	Flanking sequences/Forward primers	ICC 12968	ICC4958	Flanking sequences/Reverse primers	Annealing temperature (0C)	Amplified Product size (bp)
Ca-II-SNP271	87.33	Ca-*desi*-LG(Chr)2	4012391	CTCCCTCTATTTATAGACACATGTTCATGTTTTATCTCATTCGATGTGGGATTCTTTAACACACCTATGACTGAACATTTGGAATGTGATTCGAGGATCT	C	T	AGATAAAGTCTGAACATATGTTATAAGTAAGAATAATCTTTACTTTAAAAGTCAATTTTGTAAGGATAAATTAAACTCAACTCAAATTCTAAGATAGTAT	NA	NA
Ca-II-SNP272	88.21	Ca-*desi*-LG(Chr)2	4031210	TGATCAATCCCCCTTCCCATTCCTTAAAAAAATCTGGAAACTAATGAAGCAGGGCTATCTTCTGGTTATTTTTCTGTTGTGGAGCAATCTTAAATACATA	T	A	AAATCAATATAGCTTTACCTGTGATTGCCATGTACCACTAAAGTCATGTATAACATAGAATAAATGTATGTTGGCACATCAACGTAGGCATATTAGTTTA	NA	NA
Ca-II-SNP273	89.09	Ca-*desi*-LG(Chr)2	4040689	TATGACAAAAACTTATTTAATCATTAGAGTATTCGGTATATTATATGGTATTTTGTTCTACAATACTACTATTTTGTTATTGATCCTCTTTCCTATGTTC	A	G	GAAGAGATTGAGAAAAAGAGAGAACTGACAGTGCCGCCGCGGCCTCCGAGGGTGATATTGTTGAAGAGATGACCGTCAGTCATTATTCAAAAACTGCAAA	NA	NA
Ca-II-SNP274	90.85	Ca-*desi*-LG(Chr)2	4055778	TGCTTTGCATTTCACTTCAATTTAAATTACAATTACAATTGCAATTTCTCTCTCATTTAGCGCACCTTTTGATCTATTTAAAGCTTGCTTCGCAACATGA	A	G	CGAAGTGAGAACCAGAGAAGATTCCAGAAGCAAAGCAAAGAAGAAGAGAGAAAAACGGTTACGGAGAGAGAATGAAATGAATTGAAACGGTTTTGGGGTT	NA	NA
Ca-II-SNP275	91.73	Ca-*desi*-LG(Chr)2	4073623	CACATTCGTCTTTTTTTTTTGTTACCAGAAGGAATTCATTAATAGTAAGTAGTTAAAAATTTGTTGGTATACACGTACTTATCATATCACCTTACACTTT	A	C	ATACACAATTTACAAGAAAAATTATGTCATAGCCTAAACTACCTCTTTGAAACTAATATTCCCACTATTAATAATCTAATCATAATTATAAGCTTTTCCT	NA	NA
Ca-II-SNP276	92.61	Ca-*desi*-LG(Chr)2	4094950	TATTAAAATATTCTACTTTTTTAACTTTTAAGAGGTCGCATCTCTACCCTACGATTATCTGAAAGATGATTTTGTTTTTTATCGTATGAGGTCGCATCAT	G	A	GTGACCACGGATAATTTTTTTATTTATAAACTTTTAATTTTTTATTTTAACACCTCCGAAATGTTAAGATTAAACTGAAGCTTATAGATGATCGCATTTC	NA	NA
Ca-II-SNP277	93.49	Ca-*desi*-LG(Chr)2	4106081	CCATATTCATTCGAGAAGTGGAATTGTCTTAATGGATTAAGATTTTAGTCATTTTAGTGACATAAGTATTTCTAACTCTTTAGTTTTGCCACATTTATAT	A	G	TTAAATTTAATATTGTTAAAATTAAAAAAAGATTAATCTGCGATTCCGCGAACAATTTCGTAACATTTAAATTAATAGCATATATAAAACAGAAAGAATA	NA	NA
Ca-II-SNP278	95.28	Ca-*desi*-LG(Chr)2	4120640	AATTTTTGTTGTTAATTGTGGCGGTTTGTTTAATTGTTTTACTTAATTATTAAAATTATAAAATGGATAAGGAGCTTCTAGCATATCGATCTTAATTGTA	G	T	AATAAATAAATAAAATACAGAAGATATCTTTACCCAAATATAAGCAGGTTTTGCAATAATTGATTGTATATAAACATATTAACAATATCTTACTTAATGT	NA	NA

Marker IDs	Genetic positions (cM)	Chromosomes	Physical positions (bp)	Flanking sequences/Forward primers	ICC 12968	ICC4958	Flanking sequences/Reverse primers	Annealing temperature (0C)	Amplified Product size (bp)
Ca-II-SSR62	96.19	Ca-*desi*-LG(Chr)2	4129489	CGAAACCAAGGTAACGTGAAA	(ATA)5	(ATA)11	TGCTATGAGCTATGAATGGGAA	60.02	181.00
Ca-II-SNP279	97.10	Ca-*desi*-LG(Chr)2	4131868	GTTCACGCGCTATGTTGTTTTGTGAGATTCACGCGCCTTGTTTAGGTATTTTTTTTCTTCTTCTTTTTGTGGTTGTGTATATTGTTATATTAGATTTAGA	C	T	AAAATCAAAACACAAAAGCAAGAGTTTACAATTTGAGAATTCAAACTATATAGTAACAATTCAAGTACATAAACAGAGTTTCTTCACAAGAACAAACACA	NA	NA
Ca-II-SNP280	98.01	Ca-*desi*-LG(Chr)2	4139873	TTCTTTTAGTGTGTTCCCTTAGTGTGCAAGAGGCTTCCTAACACTTTCTTATTTACTCATAATGTAATTAAGTGGGTTCCCTTTTGAGTTGCAATTAAGT	T	G	GGAACATGGCTATGAACCTCTTCAATATGAAACCAGGAACCTGCTCTTTCTACTTATTGTTGTCATAAAAGTGACAAGTGCAAAAGCACCAAAAGAAATA	NA	NA
Ca-II-SNP281	98.92	Ca-*desi*-LG(Chr)2	4158262	GATTATTCAAGACATCCTTCATTGTCAAGTTATCCTTAGTTGGGAGTCGATTGTTCAAGAGACGCCTCACAAAGAGTGACACTTTCAGAAGAATCATTTT	G	A	GTCATGCTTTTAAATAGCAATACTCAGTAAAATGTGTGTATCACTATTTGGTAGAGGATGATAACATAGTTGAATCTCCATCGCGTGACTTCTTTTTGAA	NA	NA
Ca-II-SNP282	101.65	Ca-*desi*-LG(Chr)2	4173568	AATTTTCTACATTTTTAGATTGAAATTTTTTACCCCATATCCCCTGATTATACCTATACCTCCAAATATGCAAGAAAAAATGACGTTTTTGTCCTCGTTTA	A	T	ACTTGGATTCAAGTTCTCCGTAAGTTTAGAAAAAAAAACTCACTTTTGATTTTCGGAAAGGTTGAAAGGAGAAATGGTAATTTTTTTGGAGTTTTATTAT	NA	NA
Ca-II-SNP283	102.56	Ca-*desi*-LG(Chr)2	4221811	TCATAACTATTCAATTTCAAACATCACTTATCACTTATTTTGTCTTCATACTTTCTCAAAAGAAAACGACCATGCACTTTTTGTTACCCTTTCTTTTTCC	T	A	GTAGTTATCTCTTTGAGTCTCGAAAAGAAATAAAAAACATTGACATCAAGTTAGCGATGGGGAGAGTTGTGAAATATTTATGTTGTTTGTGTGAAATTTT	NA	NA
Ca-II-SNP284	103.47	Ca-*desi*-LG(Chr)2	4234380	CACTTTAAGTCTTAGGTTAATGTATGTTTAGATTCATGTACTTATGGAGTTGGAACTTGTATTTATGTCTCCTTGTAATCATAACATGTAATTAATTGTA	A	G	ACCATCAATGGCAAAAGAAGTTGTGAAAAATAGGAAGAAAGAATTTTTCTGAATGTGCTTGAGTGTTAAAAGCACTCAGCAACTTTGATATTTATATACA	NA	NA
Ca-II-SNP285	106.20	Ca-*desi*-LG(Chr)2	4291559	TAGACACACGACGTTCCACTTCTGGTTTCTGTTTTTATCTTGGCTCCTCTTTAGTCTCTTGGAAAAGCAAGAAACAGCCCACCGTTTCACGCTCTTCCTC	C	T	TCTGCACAGGTGCTGAATGAGAGATCTGGAAATCAGCTAACAAATATAACAACCATTGAGCTTCGCGTGTGGCGTGAGCAAGAGCACGATACTCCACTTC	NA	NA

Marker IDs	Genetic positions (cM)	Chromosomes	Physical positions (bp)	Flanking sequences/Forward primers	ICC 12968	ICC4958	Flanking sequences/Reverse primers	Annealing temperature (0C)	Amplified Product size (bp)
Ca-II-SNP286	107.11	Ca-*desi*-LG(Chr)2	4305455	AAAGGAGAATTTCCTTTTTTCCCCTCATTTTTATTCTTCCCAAGAAAAATACTAATTCATTATCTCATTTTTCCCTTCTCCTTCATATAACCAGACTGAA	G	A	ATTTGGGGGACTTCAGTAAACTTCTAGTCTTGGAAGATTTGGAAGAGAAGAAAAAATGGACGATTTGGAAGATAAGGTGAACATGAACAATGGAGTTATC	NA	NA
Ca-II-SNP287	110.75	Ca-*desi*-LG(Chr)2	4444895	AACCCATTTTAGGTGAACTAGTATAAATTTGTGTGTGTCCTTTTATATGTTTTATTATTTCTGTTACAAACGTTCATCCTCATAAAATTTGTATTTCATC	C	A	CGTGTGAAGTAACTGAAATATCACTAGAAGAGGGTTGAATAGAGATATTGTAGTTTTAAAATTTTCTAGTGAGCTTTTTAAAAATGTGTCCTCTACGAGA	NA	NA
Ca-II-SNP288	111.66	Ca-*desi*-LG(Chr)2	4468063	TTCCTTCCCACACAACCACAGATAAGTCCCTAATGCAAACTAGAAGTTTGGAAGGAGCCCTTACCTTAACGTTAGCTTCAACGTGGTTCTCCGGTACCGC	A	G	TCGCGAAGAGTTAGAAGGGAAAGTTGCTCACTACCACGCCACGGTGCTAGGTGACCAATTTGGAGGCGACGTTGCGAGCGAATATTTTACCGGTTTTACT	NA	NA
Ca-II-SNP289	112.57	Ca-*desi*-LG(Chr)2	4492319	CTTGAGTTGCAAGCTTTTGTTCCAAGTGTATGGTGTCTGCTTGTTTGCCTGAAATGTTTCTCAATTCAAATCATTAGATCAATCAAATACTTCATATGTA	T	C	CTCAAGGTTAAACTGTGTGTTTGTCATTAAATCAAAAAGGGGGAGTTTGTAAGTCATGAGCTTCCCTAATCATGTTTTTGATTTAATTCCCAAACATACA	NA	NA
Ca-II-SNP290	113.48	Ca-*desi*-LG(Chr)2	4503024	TCGTGAACTGGTATTCACGTGTGAAAGCTTCGAACTGGTATTTCACGATTTTCATACAATTTGATAATAGTCGGTCATATTTCACGATTTTGAAAAGTTA	C	T	CATTTCTTATTACTTTCAATTTATGTCAATCAATTATATGAGTAATATTTTACGTTACCAATCCTCCTTTCACCTTCATACACAATTACATCCCTTCGCG	NA	NA
Ca-II-SNP291	114.39	Ca-*desi*-LG(Chr)2	4522978	TTGAATATTTAACTTTACCCCTTGGGGAATTTTCTTGCAAGAACTAACATTACCCCTTAATCCTATTTGGTGTCTCTTTGCTCTAGTAATACCTCCAGAA	T	G	TTAAATCCATTGGGAATAACCTTGACCCAACTTGGGATGGAATAGCCTAAAGATATCAATAATAAGAGGAATGATACATGCGATTTTTGTCTCCATACAT	NA	NA
Ca-II-SNP292	115.30	Ca-*desi*-LG(Chr)2	4600246	TCCAACAAGATATCTAAATATGCGTTTTACTGCTGTTAAATGGGATTCTTTTGGTGCTGATTGAAATCGGGCACATAATCCTACTGCAAACACGATATCT	G	A	CCATCCACTTCTTTAGAAAAAGATGAAAATGGAAAATATGTTTCTGAAAAAGAGTATCGAGGAATGATTGGTTCTCTGCTGTATCTAACTGCTAGTAGAC	NA	NA
Ca-II-SNP293	116.21	Ca-*desi*-LG(Chr)2	4642315	TGAAAATACCTATTCTAGTTTTATCATGAGCTATGGGTTCGAATTTAAGCAAAATTCTTGAGAACGTTTGCTTCCTGAAATTTTCTTGTCTTTTCTCAAT	T	G	TTTTTTAATTCCGTACATAAAGATTCAGGAAATACAAGCAAATTATCGACAAAACTTCAAAATGACATTTTCTTTTTACCCTATATTTTTCCCTCCTTTT	NA	NA

Marker IDs	Genetic positions (cM)	Chromosomes	Physical positions (bp)	Flanking sequences/Forward primers	ICC 12968	ICC4958	Flanking sequences/Reverse primers	Annealing temperature (0C)	Amplified Product size (bp)
Ca-II-SNP294	117.12	Ca-*desi*-LG(Chr)2	4662459	TCTTCTAGTAAATAATTCATATCTTCTTGTGATAAATCTTGATCACATCTTCTTGTAAATAATTCTTATCTTCTTGTAGATAAATCTTGATCAAATCTTC	C	T	TAATTGAAGAAAATCTTTGACCAAGTTGAAAAGAAGATATGGTCAAGATTTGAAGAAGATATGATCAAGATTTATCACAATAAGATATGAATTATTTACA	NA	NA
Ca-II-SNP295	118.03	Ca-*desi*-LG(Chr)2	4699467	CTCTCTTATATTCCCTTGCCTTGATACTTCATGGAAATTAATTCTGAAGCAAAGAACTTGTTCAGCCTTATCACTTTTTCAAAGCGTTTTTTCCATCTCA	A	G	CGCTCAAATCGCATAAGTCTTATGATCATAAAGCATGGCATTCCTGAGGTCTTTAGAGGTGCTGTCTCGGATGAGATAGTTACAGCTAAAAATTTCCTTG	NA	NA
Ca-II-SNP296	118.94	Ca-*desi*-LG(Chr)2	4713211	TAACCATAACATTATTCATTTCTCAGGTTTGATCTTGCTTCTTTCAAATGATCTTGCTTCTGATTTATAGCATAATGTGATCATTGATTGTTGTATGAAT	C	G	AATGAAATAATCTTAAAAGGAATGTATCATATATTAATCATTCAAGAGAATGATTATTACATCTTCAAGATAACATCTTCATGAAGAAGATGATCAAATA	NA	NA
Ca-II-SNP297	119.85	Ca-*desi*-LG(Chr)2	4758614	TCGCGTAAACCATTTATCGCGCAACTCAACATCGTTCAAGGTTTCGTTCAATGTTTGAGGATTCTGTCTCGCGTAAACCATTTATTCCGACTGTGTTTTT	A	G	GTGGATTATAACACTAATTCGTAGCAAAATTAAAACTAATAGCAGAGCAAAATAGGATTCGAAAAATTAGGGTTTTAGAGCAAAGATACAAAATACATAC	NA	NA
Ca-II-SNP298	120.76	Ca-*desi*-LG(Chr)2	4904211	TGAATAGCAGTTATATAATAGTTATATAGTCAATTACGTAGCAGTTAATGGCTTTCCTTTCTGTTTTGTTTAAGTTGTTGATGTGGCTTCACATTCAGTT	A	T	AGTTGAGAGAAAACTCAGAGAGAAAACAATAATCTTATTCATCTTATTTTGAGGCTATTCATCACCACCATTATATAGGTGCATGAAATAACAAAAATCA	NA	NA
Ca-II-SNP299	121.67	Ca-*desi*-LG(Chr)2	5015705	ATCCTCTTTATGATGTTTTGCATGACCAGATTGCATCCACAAATCCATGTTGAATACATTTGGGGACATAACCTGGTGATTTCAAGCAAGTTGTACTAAG	C	T	TGGACTTCATTCGAAATCAATACAGGGATAGGGGCTATCAAGAGGTAAAGTTTCACTATAGAAGTTTCTTGATATTACTAAAAACAATCTTTTTCCAAGT	NA	NA
Ca-II-SNP300	122.58	Ca-*desi*-LG(Chr)2	5036904	TTTTAAAAAAGCGCTGTAATAGGTGCATATATAGCACGCGTGTTATACTAGTTTTACCGCGCTTCTTTCAAAAAACGTTGTAATAAAGGTCTTTTTTTAT	G	A	TTCAGAACTTCAAAAGACTTTAATAGCACATCTCAACCAAAAACCGTAAAATATTAGGTATATGAATCACCTTTTTTATATATCCAATATTTAATTCATT	NA	NA
Ca-II-SNP301	123.49	Ca-*desi*-LG(Chr)2	5097670	ATGATGTTGTTGTGATTGTGTTATAAGTGCTAAGTGTTATGTTTTGTTTGGAATTGTGTGATAGCATGTTTGATTGCAAAACTATTTCGAAGCTCATGAT	A	G	TTTCCCTGAATATTTTTCTAAAAATACATAAACTAACTCAATCACATTCACACACAATTCCAACACATAACACTTAGCACTTATAACACAATCACTACAA	NA	NA

Marker IDs	Genetic positions (cM)	Chromosomes	Physical positions (bp)	Flanking sequences/Forward primers	ICC 12968	ICC4958	Flanking sequences/Reverse primers	Annealing temperature (0C)	Amplified Product size (bp)
Ca-II-SNP302	124.40	Ca-*desi*-LG(Chr)2	5166682	ATGTTTGCTTCTTCCTAAAGGATGATGATCATATGGTTGATTCTATTTGATCTACCCTTTATAAGCATTGTTGGATTTTGTCTACATTTTAATGAAGGAT	G	A	CACAAGTTATCGGTGATGTTTTGTAAAGCAAAACAACAAAGGTTAAATGACAACAAGTTGAAGTTCTGCAACATAACATGGATTGGAGGAGACACTCAAG	NA	NA
Ca-II-SNP303	125.27	Ca-*desi*-LG(Chr)2	5229329	AGTACTCTTAAAGCTACATAAAAAGGTATGTTTGTTGGTCTGGTTTTGAATTCCAATTTTTTTTCTTCTAAATCTGGGGATGTTACGTGAACTCAGTTCA	C	T	AAACAACGTACGTTTACGTTAATTAAGATTGCGTACTTTCTGAAATTTTTATTTTTTTAAAACATACGTGAATTCAGTTCACGTAACAACACAAATGTAC	NA	NA
Ca-II-SNP304	126.14	Ca-*desi*-LG(Chr)2	5240432	TTCATTTTAAACACTTAGTGGAAAATCTCACAGTGTGAGGACTAGACGTAACCCACGTTGGGTGAACCAGGATAAATCTTTGTGTGGTATTCTCTTTCCT	T	A	TTCTTGGTTTATGTTATAAAACAGAGAACGTTCTTAAATTAGCAGAAAATCAGTTTCTATTTTATCTTAAATGATTAATGCAGTATAATAAAGAAGGAGA	NA	NA
Ca-II-SNP305	127.01	Ca-*desi*-LG(Chr)2	5275879	TAGTTTTTGCATTGAAGATCTGGAGGCACTATTTATATGGATGCACGTTCACAGTGTTCAGTGACCATAAAAGTTTGAAATATTTGTTTGATCAGAAGGA	G	A	TCAACGCATCAGCCACTACATTGGCCTTCCCAGGCTGGTACTACAGTGTGAAATCAAAATCCTTGAGAGTCTCCATCCATCATCTCTGACGCATGTTCAA	NA	NA
Ca-II-SSR63	127.88	Ca-*desi*-LG(Chr)2	5381402	TTTTCGAAGTATATGTGCACGG	(AG)10	(AG)9	TTTCTATGCTCAACCAAACTGAA	60.02	126.00
Ca-II-SNP306	128.75	Ca-*desi*-LG(Chr)2	5398410	ATATTTTTTAGGGAGACTTAAAGTACTTGCTTTTCTAGTTTGGGCCTTAAGCCGGCGGCCCTGCCTATCTTCTGTCAACCTATTCTACATGTGAGGGACT	A	C	ACCCTCCCTTAAAAGCTAGCTATTAAGGGGGAAGAACCGAGTGTCCCATACTGCTCTCAATGTGGGAGTTAGGCACCCCATAATACCCAAGCCCCTATCA	NA	NA
Ca-II-SNP307	130.49	Ca-*desi*-LG(Chr)2	5472382	TTTTGCCCATCTTCTCGGCGTGAGACTCGCGAATATATATCATTGTAGGTGTGCGTCTGGCAAGGAATAACACTAATTAGAAGCAATGTATTAAGATGAA	G	T	TGAGTTTGTTTCGTTGATGTTTTTCTTAGTTATTTATCTTTAGTAATTTTAGTAATATTGGAGGATTGCTAATCCTCACAACTCTTTGTAATTATATCAT	NA	NA
Ca-II-SNP308	131.36	Ca-*desi*-LG(Chr)2	5495838	TTACGACTTCACTCCAGTCACTAGCCCTACCTTCGGCATCCCCCTCCTTGCGGTTAAGGTAACGACTTGGGCATGGCCAGCTCCCATAGTGTGACGGGCG	A	G	CCTGTCCTCAGTTCGGATTGTAGGCTGCAACTCGCCTACATGAAGCCGGAATCGCTGGTCAGCCATACGGCGGTGAATTCGTTCCCAGGCCTTGTACACA	NA	NA

Marker IDs	Genetic positions (cM)	Chromosomes	Physical positions (bp)	Flanking sequences/Forward primers	ICC 12968	ICC4958	Flanking sequences/Reverse primers	Annealing temperature (0C)	Amplified Product size (bp)
Ca-II-SNP309	132.23	Ca-*desi*-LG(Chr)2	5510517	TTCCACCGCCCTATGTTCTCAAAAGCCTCCAAAATACACCTCCGGATTAGCCAATGTGGATGTGTTTTTTGTCAATATACACCTTCGGATTGGCCAATCC	G	A	GGACTTCATTACTCTTATTAAATTTTAGGAGCTTAGATTTAAGTATTTTACTATTGCTAAATTGTTTTGAGAGTTTCTTCTCCCACCCCCCAATGCTCTC	NA	NA
Ca-II-SNP310	133.97	Ca-*desi*-LG(Chr)2	5547268	CTCAACCTGTAGTTCAGGAGAAGTTCCTCATCTTGAAGAGCCCTAGTTGCTACCAAAACAACAGTTTTCAGAGTAGGAACATGTGATTCACTATTTCTTG	G	A	GATTGAAAACAACATGAGAGCTTACATTCCTAATATATTATATGGAAATGCAGAAGTAAATATGAAGAAATTTGGCAGTTTTTGGTTCAAATCTAGAGTT	NA	NA
Ca-II-SNP311	134.84	Ca-*desi*-LG(Chr)2	5561407	TATATTATGATGTGACAGCCTTTAAAATTCGTCATGTTGCCACTCTTCTCTAAGACTACATTACATTCATTCTTCCATTAACTTATATTTCCTTTTTATT	A	C	ATAAATGGGAGTTACCAAATTTATTATATGATATAATTATGTAATAATGAGTTGGGAGAGAGTTGGTTTTGGTTATAAAATTGGTGCAAGAACAAATAAA	NA	NA
Ca-II-SNP312	136.58	Ca-*desi*-LG(Chr)2	5581383	CTTAGCGTAGTTGTCTAGCTTTATAATTTAAAAGAAATCAAGATTCCTTAACTTTGAATTTCTCTATCTTTAGTGACTTTGTTTCTGTCTTCCACTTAAC	C	T	AATCACTTAATTTTAGTATTTGGTAACTTTAGTATATTTTAGAGAGAAATTATTGATTAAAATAAATTAATAAAAATTATTTAGATGAGAGGAAAATTTT	NA	NA
Ca-II-SNP313	138.32	Ca-*desi*-LG(Chr)2	5602332	AGTTGTTTAGTGAATCAATGCTAGTACATCTTATCTTTTTTTTTATAAAATTTAATGTAGCATTTATGAGTTTAACTTTTATATACTTTCAGTGTAAATC	A	G	GGTTAATTACAACTATTGGTCATATGAAATATTTGACTTTTATTTTGATCATCTCTAAAGTCACAAATATGATTAGTTGTAATAGACCGATAGTGTATAA	NA	NA
Ca-II-SNP314	139.19	Ca-*desi*-LG(Chr)2	5616669	TTGCTCATTTTGTAGGGATATTTTTTTTAATTATATTCTTTTTTCAAATTCTTTTCCATATATTTTATTTTGAATTTTTTATATGTTATATTTTTTTTTA	C	G	TATTTTTTATTTAATATATTTTTTCTATTTAATTTTTTATTTAATATTTTAGTTTTTTTAATTATTTTTTTATATTTTATTTATTTACAAACTTTTAAAG	NA	NA
Ca-II-SNP315	140.06	Ca-*desi*-LG(Chr)2	5627315	AATTTTCCAATCGATTGTGCTGATCAAAGTGACTCAGACATTTACGTCAATCGATTTGCCAATCGATTGTGCTTGTTACAGAACCTCAGCTATTTTGTCA	G	A	AGATTTCTTAAACTACAAGTTTGTGGCAATCGATTAGGCAATCGATTGCAGTTAATCATTTATCAGAACCACTTTGAATAAATCGATTGGCACATTGATT	NA	NA
Ca-II-SNP316	140.93	Ca-*desi*-LG(Chr)2	5645696	TATATTATGATGTGACAGCCTTTAAAATTCGTCATGTTGCCACTCTTCTCTAAGACTACATTACATTCATTCTTCCATTAACTTATATTTCCTTTTTATT	A	C	CTTCCAAGTTCCTTATATAAACAACATCTATTCCAAAGTTTGAACTACTTCAACTCGGGAATTTCCTTGGTGAAAGCTAAGCTTTTGAAACCATGGCTCTAACTACCAACCCTTCATCCCTCCTTTGTGTTTCCCTCATCGCCATTGTCCTCTACTTTGCTGATTCACAATCCTTCATCGGAGTAAACTACGGCCAAGTCGCCGACAACCTTCCGTCACCGGACGCCACGG[illegible]	NA	NA

Marker IDs	Genetic positions (cM)	Chromosomes	Physical positions (bp)	Flanking sequences/Forward primers	ICC 12968	ICC4958	Flanking sequences/Reverse primers	Annealing temperature (0C)	Amplified Product size (bp)
Ca-II-SNP317	141.80	Ca-*desi*-LG(Chr)2	5657560	TTTACACACACCTATCTATGGGGCCGTTTAATTCCAATCACGATAACACAAACTCAGGCTGCTTGAATCAACATATCTTGACTTGATTGAACTAGCCGCT	T	A	GGTTGTCTTTTGGACTTGATGGTGCAAAAGATGTTGCAATGAAACTGGCTGTCAGATCCGATGATGAAACTGTCAGCGATGCCATCCATGAGATTGTAGC	NA	NA
Ca-II-SNP318	142.67	Ca-*desi*-LG(Chr)2	5685363	TTTCTTTATAGTTTGTTTAAGATTGCTTCCTATCAAGGTTAGATAGGTGTTGTAATTGCTTTCTGGGTGGAAAGTAATTAAGAAAGAAATAGATCAAGGT	C	T	TCACCCAACGTGGGTTACGTCCAGTCCTCACACTGTGAGATTTTCCACTAAGTGTTTAAAATGAAGAGCCTTCTTAATCTTACAACACCTAGAATAGATC	NA	NA
Ca-II-SNP319	144.41	Ca-*desi*-LG(Chr)2	5717662	TATATTGGTATCTTTTTGCGTTAAAATTTTATGTAATATTTTGTTGTTTCAGACAAAGGGATGAAGAGTTGGATGCTGTTATGCTTTCTGCTCAGGCACT	A	G	CAGATACAAAGAGTGCTAGTAGTGATGCTGTCTGCAATTTATCAACTTCACCCCGACTCCCAGGCGTGTTCAAACCATTTAATTGCTTCAAATTAGAGAC	NA	NA
Ca-II-SNP320	145.28	Ca-*desi*-LG(Chr)2	5753868	TAATTTTCTTTCCTTCCTTTTTCCTTTCTTTGTTTTTCCTTCCTTTCTATTTCCTTCTTTCCTTTATATAACCTTTTCTTTTCCTTTTCCTTCCTTCTAA	A	T	TATTTTGACACAGTTTAGAATTACTCGTCGATAAATAAATTTAAATTAAGTGCGGTAAATCCATTACGGAGAATTTAATGCGTTACGGGTGAAATGGTAA	NA	NA
Ca-II-SNP321	147.02	Ca-*desi*-LG(Chr)2	5783837	TGTACTTACATATCTAATATATTATTGTTTTGGGATATTTGTCCTTTGATGGAGTGCCTTGCTAGTTTTGGATTTTTTGTGTTGCTGTTTCAGCAAGATT	C	G	AACAGAACAAAAAAAAATGACAAACCAACTTATCCTTAATTCCACATCCTCGGTGAGGGAAAAAGGTACACTCTAGAATCTTGTATGCTTCAACACTCTA	NA	NA
Ca-II-SNP322	147.89	Ca-*desi*-LG(Chr)2	5834516	TATGGTAGCATCTTCAAGCACAAAGCTCTCATGGAATAACTTGAGGTGTTGTCCATTTACTTTGAACACTTTATCGGTGTTTTCACTTTTTATTTCCACT	G	A	AAAGTTTTACTGTTCAACTCCCGTCTAAAAATTATGGATGGGAAACCTTGATCCAAGTGGATTGGCCCTTTTGCTGTTACTAACACTTTTACTCATGGTG	NA	NA
Ca-II-SNP323	148.76	Ca-*desi*-LG(Chr)2	5857541	TTCAGTAAGAGTTCTATTCTTTCTTTCAGCTTTACCATTCATTTCAGGTGAATATGGTGCAGTTGTTTCATGTACAATTCCTTGGGTTTTATAAAACTCA	A	T	CATGTTCAAAATATTTGTGACTGAAATAGAAAATCAATTTAACAAAAAGATTAAGAGGTTTCGTAGTGATAGAGGAACAGAGTATGATTCAAACTTTTTA	NA	NA
Ca-II-SNP324	149.63	Ca-*desi*-LG(Chr)2	5874928	AAACTGTCCTTGGGCAAGGCCTTGGTGAAAATGTCAGCCAACTGATCAGTTGTATTGACATATTCCAGCCTAATCTTGCCCTGTTGGTATTGGTCTCTAA	G	T	TACAGTGCCCATTATGTGTGATAATACTAGTGCTATTAATATCACTAAGAACCCTGTCATGCATTCTAGGACTAAGCACATAGAGATTAGGCATCACTTC	NA	NA

Marker IDs	Genetic positions (cM)	Chromosomes	Physical positions (bp)	Flanking sequences/Forward primers	ICC 12968	ICC4958	Flanking sequences/Reverse primers	Annealing temperature (0C)	Amplified Product size (bp)
Ca-II-SNP325	150.50	Ca-*desi*-LG(Chr)2	5898120	ACCTCAGAAGGCAAAATTGGCTGATCAATGCATGGACTACCCAATAACATTGCAAGTGCTCTTTCCAAAGATGTTGTTACTTTGTCCATTGAAGGTCTAT	T	C	AGCTGCAATTTTGGATCCAACTCTTGAGCCACCTCATGATGTTGAAGCATTGAAAAGGATAGCTAATGTAGCTTGTAAATGTGTGAGAATGAGAGGGAAA	NA	NA
Ca-II-SNP326	151.37	Ca-*desi*-LG(Chr)2	5914388	TGCCCCACAATCATTGGCAGCACCATCCTCTTCCTCTTCCTCTTCCTCTTCCTCTTCCTCTTCTCCCGAATCCTATCAATGAAGTCAGTCTAGAAATTAA	G	A	TACATACCCAAAAAAACCAACCCTTGTCCTAAAACCAAGGAAAATGAAAGAAAAAAAAACACAAATACTTCCTGTTATAGTGGTAGTGTGACCCCTATTCCTGACCATTAGTATCACATTACATTAATGTAAAACCAAGTGATAATCTAACCTGACTTAGATTCACCATGTTGAGTAGATCTAAGTTGTTGAATAGAGATTGAATGACTCATCTCATCTCAAAGTGCCCAAT[illegible]	NA	NA
Ca-II-SNP327	152.24	Ca-*desi*-LG(Chr)2	5923861	TGTAGGAGCATATCCGCTCTTGTGTATCACTTTCTTTGGAGTAATTCTCTCCTATTATTTCTTATCATTTTTCTAAATATTAGTGCCCTTCCTCTAAATC	C	T	ACTTCTTGGCCCACCAATCCTACATTTCCATGAAAATTGGCACTCCCTCGAGATCCGGCAGGTCGAACCAATTGTTAGGAACCCAATAATTTGATTCCAA	NA	NA
Ca-II-SNP328	153.11	Ca-*desi*-LG(Chr)2	5933735	CTGGGAGTGATAAAATTGATGATGCTTCTGCTCTCTCCTTGTGGCACCCCAATTTGAAGTTGCTCCTTGTCACTCTTGGTGAAAATGGTTCCAGATACTA	T	C	TACTACTGTACCATACATAAGCTAAGATTGAAAAACTATGAAGCTTAATAAAGAAATAGGAATAATAAGTAAAATTGAAGATTAGGCCTTTTACCTTGGT	NA	NA
Ca-II-SNP329	153.98	Ca-*desi*-LG(Chr)2	5957298	AGTTGTAGGCAATTATACTGCTACAACTCAACTACCTAGCAGTTTGGAGTCTGACACTAGAGCTATTATTCCTGCTAAGGTTTTTGTCATGGAGGGATAA	C	G	ATTGACTCTTCAGCAGCAGTTCCTTTTCCCAGGTAGCATCTCCAACATCCATGCCTTCCCATTGAATCAACCATTCTCGCTTATGCTGCCCACCATCCAA	NA	NA
Ca-II-SNP330	154.85	Ca-*desi*-LG(Chr)2	6012474	TGTAACCACCACCACACTCAATATCCTGCTCAAATTTAATAGAATACTGAACCACCAGGGTTCTGTTCTTATTGGTCAACTCTGGTATCTTTGCAGATAT	G	T	GTAATTAGTGATTTTAGTTATGGTGTTATAGGCTTGATTATCACTAGTGTTCTACAATTCTTGCAGGTATTCAGACATATAATGATGCCAAGCATTTTTC	NA	NA
Ca-II-SNP331	155.72	Ca-*desi*-LG(Chr)2	6034855	AAAATTTGAAATAATTTTGATAACAATTAATTGTTTTAAGGCTTAATTTCAGTTTTGGTCCCCCTATTTTAGTTGAATCGCGAAAGTAGTCCCTCCATTT	G	T	AAATCTATGATCATAAATGGTGTAGTACGATTTTAAAAAATAAAATTTCATCAAATTTTGGATCAAAATTCTGTTTGGGGGACCAAAACTGGGGAGAAAC	NA	NA
Ca-II-SSR64	156.59	Ca-*desi*-LG(Chr)2	6056181	AATCAGAAGATGCCCCACAA	(TCCTCT)9	(TCCTCT)6	GCATTGAGAATGCTCTAGTGGAA	60.46	118.00

Marker IDs	Genetic positions (cM)	Chromosomes	Physical positions (bp)	Flanking sequences/Forward primers	ICC 12968	ICC4958	Flanking sequences/Reverse primers	Annealing temperature (0C)	Amplified Product size (bp)
Ca-II-SNP332	157.46	Ca-*desi*-LG(Chr)2	6056256	TGCCCCACAATCATTGGCAGCACCATCCTCTTCCTCTTCCTCTTCCTCTTCCTCTTCCTCTTCTCCCGAATCCTATCAATGAAGTCAGTCTAGAAATTAA	C	T	CTTAAATTGAATTCACTGACTTGGGGGATACAATTGTTTTTACTTTCGTTTCATGTGAGATATGTATGTCAGTTTGCATTGAGAATGCTCTAGTGGAAAT	NA	NA
Ca-II-SNP333	158.34	Ca-*desi*-LG(Chr)2	6067444	ATCATTGATGAATGACTTATGCTTTCACTGAGGCTAATATCTGATTGTATAAATAGATACCCTTCCATCTCATCACACGCCTTTGGGACACATATCTGGC	T	C	AAATATATTATTTTAGTGAAGAGGCCAGAGTAAGCTAGCTCACCGTTAGAAGGAAACTGGCAAATATATACACTAAGAAGTCTGGTAAGGCATCTCCTTC	NA	NA
Ca-II-SNP334	160.10	Ca-*desi*-LG(Chr)2	6105613	ACATACTTTTTCGAATTCATTTCCCTTTATCTGTTATTAATTTGCAAGAGTGCATTGATGTGCAGCCTACTTTGTCATATCCCATAATTAGACAAGCGTT	A	G	TTTTCACCAACCATGATCTTATAATTCATAATAAAATTCGAGTCACAATTCATAAGAAAACTGTTCCAATTCTTAGTTTGAATCACAATTTCGTAACTAT	NA	NA
Ca-II-SNP335	160.98	Ca-*desi*-LG(Chr)2	6133438	GGCTTTGGAACGTCGTTTAGCTTCAACTTCTCGAAATCCTGAGACTCCTCATGAAGCTTGGTTCCAAGGAGAGTATAAAGACCAACTGAAATCAGCTATT	G	T	GATTTCCTCTGGTAAGAAGCCAAGGATGCAGCAATACCATCAAAAGGCCGCCATACATCCCCTAGAGCTGAAGAAGATGGTGGAGGAGTCTTGAAAGATA	NA	NA
Ca-II-SNP336	162.74	Ca-*desi*-LG(Chr)2	6220860	AGTGTTTGAAATCTGGCGCGGAACCCGAATCAAAGTTATAAATTTTAGGGAAGAATGCTATTATGGTCTCGCGGTCCTCGATTTCATTTGGTCTTTTCGG	A	G	ATAACTATGTTTCATGTTTCGCGCCAGATTTCAAACACTAGCCATCGTTTGGTCCAAAAATACAGATGTTGGTCCATGTTTGTCGAGGGGTACGAAAACG	NA	NA
Ca-II-SNP337	163.62	Ca-*desi*-LG(Chr)2	6246652	GTCCATATGTTTGTGTTTTTAGTGTATTTCAAAAGTGTCTTGTTCTATAAGTCCCACAATCATATATCGCTCATGAGAACAAGGACGAAGCTCTAGCTTC	T	G	AATGACAAATATTTTGAAACAGTTTGTTTTTTCTTTTTAATTGATATTTATTCTGAGATAGAGGAAATGTCAACGGTGGTAGATGTCACTAATTAAAACT	NA	NA
Ca-II-SNP338	165.38	Ca-*desi*-LG(Chr)2	6328392	CCTTTCTCTTCACGGAAGGAAGAACCTCCCTGTTTTCTGTAAGATAGCACTGTTAGACTCTAGCTATCTTCACTATGAGAACACGGTCATCGGGACTGTC	C	T	ACTTGGACTTTTAGCCGTGTTGGCAAGGTACTATCGTTAAGACTTACGTTGTAATTTAGGAAGATAGTGAGAACTACGCTTCCTGCATGTAGTGAAGTCA	NA	NA
Ca-II-SNP339	166.26	Ca-*desi*-LG(Chr)2	6414438	CAATACAACTTTTTAGAACCAAGAGGCGGCTTTATCTCATTGATAGCCCTCTCCTCCTAAGGGTGGCCTTTCCCTTAGGGGCGGCTCCATCTAACGGATG	A	G	CCTTACGAGAGAGGTCATCCGTAGGAGGAGAGGGCTATCAACGAGATGTAAGCTCCCTAATTGATATGTGCTGTTGCGTTGCGGAATTGAGGGGAGAGGG	NA	NA

Marker IDs	Genetic positions (cM)	Chromosomes	Physical positions (bp)	Flanking sequences/Forward primers	ICC 12968	ICC4958	Flanking sequences/Reverse primers	Annealing temperature (0C)	Amplified Product size (bp)
Ca-II-SNP340	168.02	Ca-*desi*-LG(Chr)2	6423896	TTGTTCATAGCCTTAGTGTATAACACTCAATCCTTTTACCCTTATCTTATATTTGTGTTATGCTTTTTAAATAATCAATAGTTAAATCTTTTTGTTGTTT	T	C	TTTATCTTAAAACCATTGAAAAATTGAAAGTGGGACAATGACATCAAACAATTATAAGATATATATTTTTTAAAGGAATTATAAAATATATTGAAGAGAA	NA	NA
Ca-II-SNP341	168.90	Ca-*desi*-LG(Chr)2	6434776	GTTGGACTGACATTAATTGGTTTTGTTCCATTAAAGACTAGCATTGGCTACTATGTAGGATTGCAATCGTTAGGAACAAGAGAATCATCCTTAATTGGTT	C	T	TAATTTTGCATCCTTTAGTAAGAAATGTTCATCTTGAATGCTTCTGGTGTGGATTATCACTTTGCATCACTATTCTGCTACTGCTACCAATCTTTATGGA	NA	NA
Ca-II-SNP342	169.78	Ca-*desi*-LG(Chr)2	6445277	TGTGGTAGCCGATCTTCAAATTTATCAAACGTGGTATTTCCTTTGTAGGAATCATTTCCAAATTTATCAAACTATTGACTTGTAGATATGATTCCCTCAA	G	A	GGAGTGGTTGGACCTATCAAGGAACCAGTTGACAGGAGAGATTCCTCTGGCTTTGACAAATTTGAATTTCTTGTCTTTCTTGAACCTTTCACAAAACCAG	NA	NA
Ca-II-SNP343	170.66	Ca-*desi*-LG(Chr)2	6455481	ATAATCATTATTTTTATTTTTATTTTACTTTTTATCTTGAGTACTATAATTTCTATATTCTTTTTTTACTTAAACTAATATTTCTTATATTTTATGTTTC	A	G	CCAAAAAAGATAATTAAATCAAAATATTTGTGAAAAAAACTGGTGGCATAATATCTCTAATAAAGAATTAGTTATCGGAAGTTTAAGTGAAAATGAAACA	NA	NA
Ca-II-SNP344	171.54	Ca-*desi*-LG(Chr)2	6478674	CATCGATTCAAAATAACATGTGCAATTATACTTTGTCGGTTTCTGTTCATAACTCAGGAGTTTTGGGTTAGCATTACTCATGAATTTTATAATCTCAACA	G	C	GTCAAATTTAAGGATGTTTGCATACGAGAATTAAGAATGTAGCTTTTGAAACAGAGATACTTCAATGGTCAATTCACCGAACTGACGGCTACAAAGAAAA	NA	NA
Ca-II-SNP345	175.94	Ca-*desi*-LG(Chr)2	6488476	CTATCTGAACAATGGATTTTGCCACCAAAAAACCTGTTGAGAAATCAAACTATCTCTTGTTGTGTAAAGGGTTGGATCCTGTAGGCACTTTTCGTTTTGC	T	A	GGATCAAATTCTCAAGTCCTTCTACTTCAAATCATCACCAAAATTTTCATATTCCATTTGTTAATTCTCCGGTAAGAAGCAACGGTAGAGAATTCAAATC	NA	NA
Ca-II-SNP346	176.82	Ca-*desi*-LG(Chr)2	6501665	AACATTAATGACTCTTTCAACAGGGTTAATGTTCCATTCCCTTGTGATTGCCTCAATGGAGAGTTTCTTGCTTACACTTTTCAGCATAAGCTTCAGTTGA	C	G	TTCAGGAAGATTACTTGGTGAATAAACATTGACTCTCTCTATCCATTCCTCAGTGGTCAAATTGCTAAAAATAAAATCAGCAATTGAGCCATAAGTTTCA	NA	NA
Ca-II-SNP347	177.70	Ca-*desi*-LG(Chr)2	6513092	TTTGTATGTAACGTCATATTCTTATTCATAAGAATTCTTTCTCTCACATCTCTACTACCGATTTTTTTTAATTCTTTTTGCTAATGGTGATGTTTTAATC	C	T	CAACTGCAACCACAATTTAAAATCATAGACCTCACCCTCTATATAACTTTAACTTTTTAAAATGTTCAATCATTATCCTCCAATAGTTGAATCTATATAT	NA	NA

Marker IDs	Genetic positions (cM)	Chromosomes	Physical positions (bp)	Flanking sequences/Forward primers	ICC 12968	ICC4958	Flanking sequences/Reverse primers	Annealing temperature (0C)	Amplified Product size (bp)
Ca-II-SNP348	178.58	Ca-*desi*-LG(Chr)2	6524433	GAGCTTCATTTGGAAGATGTGAATATGTCTTCCATAAGAGAGAGTTCTTTGTTGCTTCTAAAGAACTTGTCGTCCTCTTTGGTCTCTCTTAATCTAGCAT	T	C	TTGACCGCCAAGTTTATTATCATAAGATGATGACAAATCTAGTAATTTTAGATTAGGTAAAGAGAAGAGATCACTAGACAAATTTCCTTGCAACTCAGTG	NA	NA
Ca-II-SNP349	179.46	Ca-*desi*-LG(Chr)2	6539044	TAACCCTTTCATTTTCTTTTCCTCTTCAAAATTATGTGTTCCAACTGTGCTTTTCCATTCCAGCTGTGTGTATACTATTCTAGTGCAACCCACCACCACA	G	A	AAATTGGTGGCCAAATGTGTCACCGTATTCGCCGGAGTTTGGCAGCGCAAAAATAACAAAAAAAAATGTCATTGTGTTTGTCGGAGGATTGCGCGACGGT	NA	NA
Ca-II-SNP350	181.22	Ca-*desi*-LG(Chr)2	6625748	ATGATTTATGATTGGCTATTAGGGGTCATTTGATCCATGCCAGTAGGAAAAGGCATTGGTTATTGTAAATTTATTACTCGAGTAGTGCTAGCAACACTCT	T	C	TACATGTGGATCCCACCACTTTATATGAGACTCATTTCCCAAATGGTGGGACCTACATGTATTTCATCCAATAAAAGAATGAATGTTGGAAAGAGAGACA	NA	NA
Ca-II-SNP351	182.10	Ca-*desi*-LG(Chr)2	6765331	TCTCCAGAAAGTTCAAATTGTCAAAACCAGATTGATAATCAATCTTCGTTTCTGCCGAATTTCAGTTTTGATCTCCTTACTTCTGCAGAGAATTATAATC	A	G	GTTTGCCTTCGAGTTTAAAAAGCTTTGAAGCTTCTCATTTAATCATTGATGATACAACTTTGATCTGGAGCCTTGAATATGTCCAAAACAGTTTGATGAA	NA	NA
Ca-II-SNP352	183.05	Ca-*desi*-LG(Chr)2	6775211	CCAATCGATTGTGTTTAAGACAGTGACTTAGCTATTTTCAAGTTAATCGAAGTGCCAATCGATTTGGTAGTATTTGCCTGAAAAGTTCTTACCAGATGTT	T	C	ATAACTTGACCTGTGACAAACACAATCGATTGGACAATCTATTGTGAAAATTTCAATCATGTTAAGTTTGGTTTTAATCGATTGGGAAATCGATTGAACT	NA	NA
Ca-II-SNP353	184.00	Ca-*desi*-LG(Chr)2	6797334	TGCGTTTGAATTACAATATGAAGGATCTCCTGTATACACTAAGGCTTTGTTTGGCAAGGCCGAACTGCTAGCTTATAGTGTATAAGCTCGTATAATCAAA	G	A	AAAATGCTAAAAAATAATTTATAAGCTCAAACGCTACTTGAGGTAGCATATCAAAAAACACACTAAGCTCGTGAGAGAAAACCGTTACCAAACAACTCTA	NA	NA
Ca-II-SNP354	185.90	Ca-*desi*-LG(Chr)2	6847648	TTTTATCCTGGTTCACCCAACGTGGGTTACGTCCAGTCCTCACACTGTGAGATTTTCCACTATGTGTTTAAAAACAAAGAACCTTCTTGATCTTACAACA	C	T	TGTTTAAGATTATATTCTATCAAGGTTAGATAGGTGTTGTTATTGCTTTCTAGGTGGAAAGTAATAGAGATTGAAATAGATCAAGGTTGATCTAGACTAG	NA	NA
Ca-II-SNP355	186.85	Ca-*desi*-LG(Chr)2	6862520	GTGTTCTTCTTACTTATATTATACTATTAACATGATTTCAAAACTCACCTCCTTCGTTGTTTGTGTTTGACTGGCTTGACCCTTCATTTGCGAAATCGCA	A	G	AGTAAAAGTAAAATACAATTTACAATTCTTATTCCCGGTATCACCTATCAGAGCGGTGTTTCTCCCAACTTGAACTTCAAGACTAATTAACCTGTAATAA	NA	NA

Marker IDs	Genetic positions (cM)	Chromosomes	Physical positions (bp)	Flanking sequences/Forward primers	ICC 12968	ICC4958	Flanking sequences/Reverse primers	Annealing temperature (0C)	Amplified Product size (bp)
Ca-II-SNP356	187.80	Ca-*desi*-LG(Chr)2	6904261	AAGGAATGCCCCATCTGTTTGATCTGAGAGGCTCACCACAAAACCAGAATACCGAGGTTCGACTTTGGGGTCATGATGGTCCGGTTACTTCACTTGCTTT	C	A	CCCAGTCACTATGCCTCAGCACCACCATACATTTCATCGAGTGTCGATCCCACACACGAACAGTCGTATCCCAAGAGCCACTGTAAATCCTCGTCAAATC	NA	NA
Ca-II-SNP357	188.75	Ca-*desi*-LG(Chr)2	6923340	GTTGTTTGTAGTTTGTATGTCAACTATAAGTTCTCCCTCCGCTTCATATTATGAATTATCTTTTAAGGTTATGCACACTTCTTAAGAAAATCATCGAGTG	T	C	AGACCATTATTTATTGAATCTCAACTCACCTAACTACTCCACTAAATACTATTAATAGTGTACTTTAGTAAGTGAAACTTATTTTATCATAGAAATTAGT	NA	NA
Ca-II-SNP358	189.70	Ca-*desi*-LG(Chr)2	6939183	ATCAAAGAATAAGACTTCACCATCCTCCGCACGTTGTGGAGGATTTTCTTCAACTGCATCCTCAACTGAAGGAGCGGCAGTATGTGTTGATGGATTGTTA	T	C	ACTGCCTCAAAGAAACATAAGTCTGCCTCTCCACTATCTCATTCTCGCACCCCCTCATTTAACTCCGCTGGCTCAACACTTCCTCAATCTCCACATGTCA	NA	NA
Ca-II-SNP359	190.65	Ca-*desi*-LG(Chr)2	7044437	TAGGGTTGTGAACTTTTTTACTTTTGAGGAGTTTGCAATCATTGTTAGGTTTTCAGGTGATTTTTAAGCACTGATTCGTGGTGAACCATATGTTGTGTTG	G	A	TAAGTATAGCTTAATATTCCAACAACTGAAGACAAAATAATTATAAAAATAATAATAATATCAACTCTAATAATTATAAATAGGCGAGTGATTCGGTGGA	NA	NA
Ca-II-SNP360	191.60	Ca-*desi*-LG(Chr)2	7089449	TTGGAGACTTGTTTAATTGTCCATAGTCATTAATTTGATTTACAAAAGTGTCATTCAACTATCCTTGTAATACATATGCTTAGGAGGATGAGTTTGCAGT	C	T	CAAGAAAAAGCAAGACTCTACAGTTCTTGAAAAAGGAAAAAGTCAAAAGCACAAGTCATGTCCTAATTTATGGAAAACCCAATCCTCATTTTCGATGATG	NA	NA
Ca-II-SNP361	192.55	Ca-*desi*-LG(Chr)2	7100347	GTCAACAGGAATTGCATGCAAGTCGCAATAATGGCCTTGATTGAGTGTGTGAAATTTGCCGACGTCTATTTGATATTATATTGAATGCAATAATGTCCTC	G	A	TTTTCATGAAACATGTGCAACTTATTGACATTCCAAAGTCCAAACAGCATATCACAAAACTCGGTTGAGAGGACGTTATTGAATGCAATGCCCACTCAAT	NA	NA
Ca-II-SNP362	193.50	Ca-*desi*-LG(Chr)2	7124069	CAAGATCAACAAAATCCAGTGACTTCTTCGAGTGAATACAGGTTACCTCTTGTCTGGATTGACTTGGAAATGACTGGTAAGCATGTAGAGTGTGTTATTT	T	C	TCCAATATTCTATCCACTTCAATATTCAAACCTGCAAACACACAAAAAAAAAAGTTTAACCGGGAGGTTCAATGTACTGATCGAGAGCTTTGTCACTCTC	NA	NA
Ca-II-SNP363	194.45	Ca-*desi*-LG(Chr)2	7267988	TGGTGGCCGCCGTGGCAAGACGTTCTCTTCTTCTAGCAGCAGGTACCCAAGTACTCTTGACATGAGTAGGGCAATCAAAACCTCTACTTTTACAACAAGT	T	C	GTGGTTTAACTGGTTCAGGTACAAGTAGTGGTGGAGGAACAACAACGTGTCAAGATTGTGGAAACCAAGCGAAGAAAGATTGTACTAATAGAAGATGTAG	NA	NA

Marker IDs	Genetic positions (cM)	Chromosomes	Physical positions (bp)	Flanking sequences/Forward primers	ICC 12968	ICC4958	Flanking sequences/Reverse primers	Annealing temperature (0C)	Amplified Product size (bp)
Ca-II-SNP364	195.40	Ca-*desi*-LG(Chr)2	7520753	TTTCCATAGCTAACTCGTGCGACATAATGTTGTGCGTGTTCAATGATAAAGTTAGAGATTTTGTTATGTTGTGCAACCCAATCACATCAAAGTATATGGA	C	A	ATTGAAGACCTTCAATCAATTAGTCCCTCCTTCATCTAACAGGGTCAATAATGTTTGAGTTGGCAAAACATTTAGGGCATGCTTAGTTTTTGAAAAATTGTTTAAGCTTTTGTTGATCTCTATTTCGCAATTCAAATTCATCACAA	NA	NA
Ca-II-SNP365	196.35	Ca-*desi*-LG(Chr)2	7527128	AAATGTTCTACTTATCTTTTGAACCTTCCCTCTAGACATTCTATGTATCCTCTAAACCTTCCCTTTGGACGTTCTATGTATCGCATGAACATTCCTTCTA	G	T	GTCCAAAGGGAATATTTAGAGGATAAGCAGAACGTCAAGAGGAAAGGTTCATAGGAGAGTAGAACAATCAAAGGGAAGGTCCAAATGATGCGTTGAACGT	NA	NA
Ca-II-SNP366	197.30	Ca-*desi*-LG(Chr)2	7585433	ATATACTCTCTATGTCTCATTATAAGTGCCTTCTTTCAAATATTTGTCTCAAATTATTTGTTACTTTAGAATAACCATGCAATATTTATTAACTTTTTCC	T	A	TTTTAGTGAAATTACGTGTTAAGAATAAAATACCACATTAATTGGAGTGGTCGAAAGTAAGAGTAGAGACTTAGTTATTTAAATAAAATGAGTGTATTTT	NA	NA
Ca-II-SNP367	199.20	Ca-*desi*-LG(Chr)2	7701830	CCACTTTGGATTTCTCTAGAGATAGAATTCCTTTTCTTTATTTAGGGGTTCCAATTTTTAGAGGGAAACCAAAAGCTAACCATTTAATACCCATTGTTGA	C	T	CAACATAAAATTCCGAATACAAGTATCTAAAGAATTAAGAATGTTAGTCCATGATCGAAAGCATGGACCCTTTCTAAGAGGAAAACTTGTTTTTGATGCG	NA	NA
Ca-II-SNP368	200.15	Ca-*desi*-LG(Chr)2	7726596	TCAAGGGTAATGTCAAGTTTCTATTTCATCTTAATTACTCTCATGCTGCTTTTTATTTGTTTTGTTTTGTTATACTTAGTAAATTACCTTTTCTTATCTA	C	G	GGCACCAATGACTCCAAGACTAGCAAGCCTCAAAAACTCATATGGTGCTGACATGTTTGTGGTTCGAAGGATCGGATACAAATAGAGAGGTATATAAGCT	NA	NA
Ca-II-SNP369	201.10	Ca-*desi*-LG(Chr)2	7736715	TTTCCATAGCTAACTCGTGCGACATAATGTTGTGCGTGTTCAATGATAAAGTTAGAGATTTTGTTATGTTGTGCAACCCAATCACATCAAAGTATATGGA	C	A	CCACCTTATACTGTTTACGTTGGTTGTCACGGCAGAAACCAAATCCAACAAAAATAAAATCACAAGAGAGAGGTGACTTTCAGCATTTTTCATTTGTTAC	NA	NA
Ca-II-SNP370	203.00	Ca-*desi*-LG(Chr)2	7757375	AAGAAGAAGGGGTTAGGGCTCATACGATTTCTGGGTATGAAGGGGTTAGGGAAGAAGGCGATTTCTGGGTTTACCTTGTACAATTTCTGGGTATAATCTG	A	G	TATAAAAAATTTCTGTTTAATTTTTTAAAAAAGGTCCCCAATAGAAACTAAATTAATTTTATTAATTTTAGTAGTGTTAACATAGGCTGCCTTCCAAAAC	NA	NA
Ca-II-SNP371	203.95	Ca-*desi*-LG(Chr)2	7839853	TAGGACATGACTTGTACTTTTGACTTTTTCCTTTTCCAAGAGTTGTAGTGTCTTGCCTTTCTTGTCAAGTACAACACTTTTTTCTATTATGGTACTTTTG	A	G	AACAGTAAAAGGATAAACAAATCAAGAGGATTCAAGACAGAGGATGAATAAATGAAAGGATGAACTTCTGGTGTCATTCATCCTCTTACATAGGGAATGT	NA	NA

Marker IDs	Genetic positions (cM)	Chromosomes	Physical positions (bp)	Flanking sequences/Forward primers	ICC 12968	ICC4958	Flanking sequences/Reverse primers	Annealing temperature (0C)	Amplified Product size (bp)
Ca-II-SNP372	204.97	Ca-*desi*-LG(Chr)2	7865097	CTTTCAGTTTTTTGGTTCTTTATTTTTGAATGCGGCCCAATTAAAAATCTTTCACTGAAATTAAATGCGGCCCAAGTTTTGAAACATATACTAAAGCCCA	A	G	TTGATGTCATGTGTTTGTTCTTTTTTTTCGAATAATTTAATTTAATGTCATGTGAAATTAGTATATGTTTCAATTTCAGTGGAAGTTTTGGGTGGGCTTTA	NA	NA
Ca-II-SNP373	205.99	Ca-*desi*-LG(Chr)2	7884187	GATATTTTCTTGAAAAGCCATGAACGGCTCCTTTTTCCTCTTTTGTATTGGCCAACTCTCTTATGTTGATTTTCAATACTTCAGATATTTCATCCAAACT	C	A	TTCAAGACTTTCAGTTGGCCCTGACATTAGAGGAATTTGAACGAATATTGGGTTACCCCTTAGAGAAAGGAAATCCTTATCGATATACTGGGCAACCACC	NA	NA
Ca-II-SNP374	207.01	Ca-*desi*-LG(Chr)2	7916698	ACGATATCAAATACATTGTAAGTTCACTACTTCGCACAATATCGCCAATTTTTTTTCTTTTTTTTTTTTTTAGATTCAGAATCCTTTTTATATTTGTGAA	C	T	AATTGCTATTTGCCTGGGTCGAAAAAGTCTTTCTCATCAGCTAAACCTACTAACACTCTCTATAGCGTCGCATTTAAAAAAAAAAAAAGGGCAAAATACTC	NA	NA
Ca-II-SNP375	208.03	Ca-*desi*-LG(Chr)2	7936003	CTCGTTGATGGATCATCGGAACTCTAAGGTATGCTCTTGAAAGTGCAAACACGAGAATGGAGTTGAATTGTGTTTGACTAAGTTGATATATTTTTCATTA	T	G	AAGCAGGATAAATTACTTATGTCACTTTATTCTTGCACTCCTTGGTTTTGTTATTGCTTCTACTCCAAGTAAATCATCAAGTCTGAACTAGTTTAATCAA	NA	NA
Ca-II-SNP376	209.05	Ca-*desi*-LG(Chr)2	7949370	TCCAGGATGAGTTGTCAAGCATAAAGAAACAACTTGCACAGGGGTTTTCTCCTGATGATGCATATCCACTGGGACCTCCATTGTTTATGGAGACACCAAG	A	G	TGAAACAGAGTTTTCAAAGCCACTTAAATGCCGATACAGTTATAAAGGATTTTACCTCATCAAAATCTGGAAACTCAATCTGAGCAAGTGGAGAACATGG	NA	NA
Ca-II-SNP377	210.07	Ca-*desi*-LG(Chr)2	7962223	TTCGCTGCTGGTTTTATGTAACTACTTATTGATTATGGCTACTTATGAAGATTTTCTTCGGTGGAGAGAGTATTGTCAGAACTCGAGTTCCGCTGTTTCC	A	G	AGAGTCTTATTACCAGTAATGTGATCAGATGCACTAGAATCGAGAATCCATGGGCTATGAGAAAGTGAGATGCAAACAGGTGAATTACCACTTTGTGCGG	NA	NA
Ca-II-SSR65	211.09	Ca-*desi*-LG(Chr)2	8238017	TTATAATAAGCATGTGTGCATGTG	(AT)13	(AT)17	TGAAAAGGAGAGATGATCTACATTG	57.79	138.00
Ca-II-SSR66	212.11	Ca-*desi*-LG(Chr)2	8278851	CAACAAAACAGACACGCACG	(AT)10	(AT)11	CATTTGAGTGACCAATGTTTGAA	61.35	123.00

Marker IDs	Genetic positions (cM)	Chromosomes	Physical positions (bp)	Flanking sequences/Forward primers	ICC 12968	ICC4958	Flanking sequences/Reverse primers	Annealing temperature (0C)	Amplified Product size (bp)
Ca-II-SSR67	213.13	Ca-*desi*-LG(Chr)2	9991894	CATAATTGAATTGTTTCCGCTG	(AT)7	(AT)8	TGTGTGAGGTTGAGTGAATGC	59.49	130.00
Ca-II-SSR68	214.15	Ca-*desi*-LG(Chr)2	10069811	TGGAAAGGAAGAGAGCAACAA	(TCA)29	(TCA)21	GAAAGCATCAGCACTTGCAT	59.98	205.00
Ca-II-SNP378	215.17	Ca-*desi*-LG(Chr)2	10438749	TTTTATCCTGGTTCACCCAACGTGGGTTACGTCCAGTCCTCACACTGTGAGATTTTCCACTATGTGTTTAAAAACAAAGAACCTTCTTGATCTTACAACA	G	A	GTCATATCATATCAATCCTATATATATTTTTCTTTTTATGCAAAATCGTTGGTAGTTAAATTAACATTTTTACACCTTCACCTGGGTTTGAACCTGGTCTTTCGACTCCTGCACTCCTTCAACCCTCGGCTCAAAGTAGTTGAGCTACCCAATCCCCCATATCAATCCTATATGGTTTGCCTAGGG	NA	NA
Ca-II-SNP379	216.19	Ca-*desi*-LG(Chr)2	11268840	GTGTTCTTCTTACTTATATTATACTATTAACATGATTTCAAAACTCACCTCCTTCGTTGTTTGTGTTTGACTGGCTTGACCCTTCATTTGCGAAATCGCA	G	C	AAAGACATTAGGATACTCGGCGCGAAAGGCTGTCATACTATTGCTTCCATAGATAGGGCCTGCAACAATGTAGATTGTTGTAGTTGAAGGGTATCCCATGGCCTTGAGAAAAATGGCTGCTTCTCTTGGTGACATAGGGCATCCTCCTTGTAATCGCCTTTCGACGCTATCGATCTCTTTTTCTTTCCAATGCTTGACTCCATACCTCATAACCCTTAGTTCCTCAGCTT[illegible]	NA	NA
Ca-II-SNP380	217.21	Ca-*desi*-LG(Chr)2	12031041	AAGGAATGCCCCATCTGTTTGATCTGAGAGGCTCACCACAAAACCAGAATACCGAGGTTCGACTTTGGGGTCATGATGGTCCGGTTACTTCACTTGCTTT	T	C	CGCGCAGACAAAAGCCGAACTCGGTTACTTTAGTTTGTGTATTATCTGCATGTGCTAGAATAGGAGCTTTGACATGTGGAAAAGAGATTCATGCTCATGCATTGAGAATTGGAGTGAGTTATGATGGTTTTGTACCTAATGCAATCTTGGACATGTATGTAAGGTGTGGAAGAATGGAATACGCATGGAAACAATTTTTCTCAACTGATCA	NA	NA
Ca-II-SNP381	218.23	Ca-*desi*-LG(Chr)2	12357547	GTTGTTTGTAGTTTGTATGTCAACTATAAGTTCTCCCTCCGCTTCATATTATGAATTATCTTTTAAGGTTATGCACACTTCTTAAGAAAATCATCGAGTG	G	A	TTTAGACTTTGAAACAAACAAATGCAGTGTCTCTGATCTCATTCCTTTGTTTCTCACCCCTTTTCCTTGCCGACCCATTTTCACTTACTCAACTCAACGTCACGTGCCTCCACATGCTTCTCTATTCTTTCTCAGATCAAGTAATTTTGGGTTTTCCTGTACCATGGATGGCATTCGTGGGGGAGATTCTCGGATGCAGATAAGCGACGGACAGCATCCTGTACATGTGC[illegible]	NA	NA
Ca-II-SSR69	219.25	Ca-*desi*-LG(Chr)2	13422213	ACCTGCAAAATGCACTAAAAA	(AAT)10	(AAT)12	AGCATGGGGAACAAAAACAA	57.53	175.00
Ca-II-SNP382	221.29	Ca-*desi*-LG(Chr)2	13759775	ATCAAAGAATAAGACTTCACCATCCTCCGCACGTTGTGGAGGATTTTCTTCAACTGCATCCTCAACTGAAGGAGCGGCAGTATGTGTTGATGGATTGTTA	T	C	CTCATATGAATAATGTTATAAAGTGACAGAAAACCTATAAAAAAACTTCTTTGAAGATTTAAACCATTCAATATTAATTACAATGGACCTAAAGTACTACAAAAGTCAGGCCACTCAAGATTCATGAAAATTTTGGTTTAGGCACAGGACCACTGCTTTGATGTGAAGGACTGAGCTTTTGAATCCAATGGTGAAGCCCCTTTTTATGATCTTGGCAAAAAGGGTTTTGCA[illegible]	NA	NA

Marker IDs	Genetic positions (cM)	Chromosomes	Physical positions (bp)	Flanking sequences/Forward primers	ICC 12968	ICC4958	Flanking sequences/Reverse primers	Annealing temperature (0C)	Amplified Product size (bp)
Ca-II-SSR70	222.31	Ca-*desi*-LG(Chr)2	14142854	TTCTTTGTACCGTTAGCAATCAT	(ATT)22	(ATT)28	GAGCGATAAATATTCCCCAAA	57.91	238.00
Ca-II-SNP383	223.33	Ca-*desi*-LG(Chr)2	14207756	CAATACAACTTTTTAGAACCAAGAGGCGGCTTTATCTCATTGATAGCCCTCTCCTCCTAAGGGTGGCCTTTCCCTTAGGGGCGGCTCCATCTAACGGATG	G	A	CAGATGGTTGCTCTTCTTTCCACATTTCTCTGCTTGATTTTATTTCATTCATTCAACATTGTTGCTTTCAAGGACCAACAATGGATGAAAGCCACTGCAACTTATGCTAAAGACACAGAAGGATCACTAATTACAGAAGGAGCTTGTGGTTATGGAGATCTCCACAAAGCAAGCTATGGAAAACACAGTGTTGGATTAAGCACTATTTTGTTCAATAGAGGGAGTACATG[illegible]	NA	NA
Ca-II-SNP384	225.37	Ca-*desi*-LG(Chr)2	14348649	TTGTTCATAGCCTTAGTGTATAACACTCAATCCTTTTACCCTTATCTTATATTTGTGTTATGCTTTTTAAATAATCAATAGTTAAATCTTTTTGTTGTTT	G	A	GTCTCTCCACCGCCGCCACAACAAACACAATCGGCGTCAACTACGGCACCGTCGCCGACAACCTCCCACCGCCATCCACAGTAGCCACCTTCCTCAAATCCCAAACCACCATCAACCACATCAAACTCTACGACGCCAACCCAGACATTCTCCGCGCCTTCGCCAACACCGGTA	NA	NA
Ca-II-SSR71	226.39	Ca-*desi*-LG(Chr)2	15249055	TTCTAAGTTTGGGGTTGCTCA	(ACA)9	(ACA)10	CCAAAGTGCACAAACTGATTT	59.73	114.00
Ca-II-SSR72	227.41	Ca-*desi*-LG(Chr)2	15754194	TGGATTTGGATTGCATGTTG	(TAA)23	(TAA)22	GTCTCGCACTTAAGCGGTTC	60.32	178.00
Ca-II-SNP385	0.00	Ca-*desi*-LG(Chr)3	4709	AAGAGTGGCTTGTACGAAACTTATTTCTCGACTTTTTTTTCCCTTCGATGAATTTCTTTTATAATCCGTTTTCGTTAACATTCGAAGTTTTTATTTGTTT	A	C	CAGTGGATTAGAGAGTTATCTGCAACAAAATATTATAAAATACAGTAGATTATTATATTATTACGTACGTACATAGGAAACCCGTTAAAACACATGCATT	NA	NA
Ca-II-SSR117	1.15	Ca-*desi*-LG(Chr)3	41679	CGAGTACGTTTGGCAACAGA	(GT)6	(GT)7	CCAGGTAAGCAAGTTCCAGC	59.90	163.00
Ca-II-SNP386	2.27	Ca-*desi*-LG(Chr)3	45404	GTGTAGAGGACCCGGGCTTTCAAATATAACTGAAGAACAGATACTCTGGTTCATACTTTTTGAGCTAGATTTTATTTTGGATGCATTATTCCAATTAATG	G	A	GTGATCTTAGAAGATCAATTCGTTAACTAGTATTGTCAATGAAGTAGTAATGTTTAAGCATTCTCATTACTAAAAGACAATGCACTTTATGCTACGTGAACTGAAGGAAGGTTCAATTGTTGTGGCGTACAATGTTAAATTTT	NA	NA

Marker IDs	Genetic positions (cM)	Chromosomes	Physical positions (bp)	Flanking sequences/Forward primers	ICC 12968	ICC4958	Flanking sequences/Reverse primers	Annealing temperature (0C)	Amplified Product size (bp)
Ca-II-SSR118	3.39	Ca-*desi*-LG(Chr)3	70241	TGTTGTGTGGTTTCCTCTCTTT	(AT)11	(AT)12	TGAGAGGCAAGAAGAGAAATG	58.74	115.00
Ca-II-SNP387	4.51	Ca-*desi*-LG(Chr)3	145155	GATTTGGTTGAGTATATGAGTTACAGGGAGACATTAGCCCATTGCCAAATCGATTATGTTTGTAAACATGAATCGACTGAGTAATCGATTATTTGATCTT	A	T	TTTGCTAATCGATGTTTTTAATGCATAAATCAGAAATTTGATGCTCAGTTCATCTCAGAGTCGATTGAGTAATCGATTTATATTTGTTTTGTTCAAAGTG	NA	NA
Ca-II-SNP388	7.87	Ca-*desi*-LG(Chr)3	276679	ACTCCTCTTCCTCTCTCATACCCTTTTCTCTCTGTTCACCGAACCCAGAAAGAGAGTCTACTCACCGAATCTAGAGAGACTCTTCTTTTTCTTTCTCCTC	C	G	CTTATAATTAAGAAGTAAATAAGCAAGAAGAAAAAACCCTAACTAATCGGTGTAATGAAGTAGAATGAGAGAGAGAAATGAAAATACCGTGAAAATGGAT	NA	NA
Ca-II-SNP389	8.99	Ca-*desi*-LG(Chr)3	339706	CCTCTTCTACTACAGCTGCTGCCCACTCAAGTTGTTGAAGGGCTTCTTGAACATTTCTTGGAATATATGTGTTATCAAGTGCTGCTACAAATGCCCTGTA	T	G	TCAACTAACAAAGACGTTTCTATTTCACTAAGAAAAGGGAAGAGAACTTTTACTCAACACCAATTGAAATATTTGTGTCGTATGGAAAATTATCACAAAA	NA	NA
Ca-II-SNP390	12.35	Ca-*desi*-LG(Chr)3	628033	GTAACGAATACAATCCCGAGCTTTTAACCAGTTGCTCAACCTCTTCGTCTTCAATAACAGCTTCTTTTAATTTTTTTCCATTGCTGAACACCCTTAATTC	G	T	GGATGGCGAGGTAATTTTCTCAATCAAATTAAGTTAAATTATATTTTTTAACATTTATGTTTAACATATATTTTTTGCTTAAATAATTTCAGGATCGGGG	NA	NA
Ca-II-SSR119	13.47	Ca-*desi*-LG(Chr)3	748034	GTTCACTCGTATCCGAAGCC	(TCT)12	(TCT)11	TTGCTGTAGGAATGGAATTGG	59.70	155.00
Ca-II-SNP391	14.59	Ca-*desi*-LG(Chr)3	776626	TTAATCATTCCATCGATGTTTACACTTTTCATTCAAGCACGCGTAAAATATAGTTGCCGGTTCATCAGCTGAACGAGTCTGAATTTCCATATAAGCAGCT	G	T	AGAGATATTGAGTAACTATGTAATCTTTGGTATATTTTTATTCATTGTTTCCATTTTCCCTTTCTTTCTGTAGTACCATGCCCAAATTGCCGCCATGATA	NA	NA
Ca-II-SNP392	15.71	Ca-*desi*-LG(Chr)3	811886	GTGTAGAGGACCCGGGCTTTCAAATATAACTGAAGAACAGATACTCTGGTTCATACTTTTTGAGCTAGATTTTATTTTGGATGCATTATTCCAATTAATG	T	C	GCTTATTTGGAGATTTATTTCTTGTTAATTTAGTGATGGTCGTGAATGATTATGAAGCATAATCATGTATTAGAGAGATTAACAGGGTAGTTGTGGAAAGAGTCATCCCATGGATTACCCCTCCAATCATCTAGAAAAGGCATATCACGACAAGCCTTCCAAACAACACTATTACACTTAAAACCTGACCCGAATGCAATTTGCCACACCCTATCTCCTTTTGAAACTCTAC[illegible]	NA	NA

Marker IDs	Genetic positions (cM)	Chromosomes	Physical positions (bp)	Flanking sequences/Forward primers	ICC 12968	ICC4958	Flanking sequences/Reverse primers	Annealing temperature (0C)	Amplified Product size (bp)
Ca-II-SNP393	17.95	Ca-*desi*-LG(Chr)3	839950	TTAGATCAGTATGGCGGCTATAGCCGGTTTTATATGCTTTGTAATGTTTCTAATTGGGTCATCATGAGATTCTCAATTTTGGTATGTGTCATCAAAGTAT	A	C	GATTTATTTTCTTCATTCTGCTGAGGCGTGCAGTTACACAACCAACAAGATGTGTGAATTTATTCAATAAGAATAAATTAATGTCAAAATGTTATACACT	NA	NA
Ca-II-SNP394	19.07	Ca-*desi*-LG(Chr)3	859723	TGGCCAGAGGTGTCACACGGGGTTGGTGGAGGCGTGTGGGTCTCACGCCCAAATAAGGAGGTGGCGCGTCCGTATGAGTTTGGCAGCGCAAAAAATGTCA	A	C	CTGCCATATATTTGTGTGCTCATTTTAGTCTCCTCCATAATTGTTTCTCAACTTCTCTTCAAAAGCTTCAACATTTCCCCAACAAGCAAACACCATGGCTAACCCACCCCCGGTTCGTCCATGGTTCCGCTTAACCTCCATTCGCCCCGCACCTGCTCCTGCTCCTGCACCTGCACCTGCACCAGCTCCAGCTCCAGAGCCACGACCAACGTTGATTCTACCTACATCT[illegible]	NA	NA
Ca-II-SNP395	21.31	Ca-*desi*-LG(Chr)3	955191	CACACAAGAGTGTTTGAGTGCGTAGAATGTTCTTCTCAATAATCTGATATTCCAACTCTATCTCTTTGTTCACGTGGATGGCAAATACTTGAGCTATAAA	C	T	ATGATGTTCATCCTCAGAAGAGAAAGAGACTATGGGGTTTAATTGTTTCCCATCACATTACTCCCAGGTTTGTTCCCTTCCCTATTAGGTATGCTTGTGA	NA	NA
Ca-II-SNP396	22.43	Ca-*desi*-LG(Chr)3	1088367	GTGTAAAACTAGTTCACACTAACTGTGCATGTCTCATTTTTCTCATAGAATTCATGTTGGCCATACCCAAAATCAGTTGTTTGCTTTAGTGTTTTGTTTT	C	T	TGGGAGAATCTTCCAGTAGTTGGATCAACAAGATCCCCAGGTCTAGTGATGTCACCTTCATTATACATTAGAGACTGGAAAATGATAAAATGTAAAGACA	NA	NA
Ca-II-SNP397	23.55	Ca-*desi*-LG(Chr)3	1201004	GGTTTCCTGTCTCTGTAAACCTTGGCACTAGCTCTCTTGATTACCCATGGATGCAACAAGATTCACATCAGTCAAGTCCACCTCAAACAAGTGGAAATTC	A	G	ATGAGCACGGGTGCAACAGAAAATCCAGAATAATGCTGCAATGGATAAATACCTGAGCTTCACCAGTGGAACTAGAAGGGGACTGGGCAGATGCAGAATC	NA	NA
Ca-II-SNP398	24.67	Ca-*desi*-LG(Chr)3	1219565	AAGAGTGGCTTGTACGAAACTTATTTCTCGACTTTTTTTTCCCTTCGATGAATTTCTTTTATAATCCGTTTTCGTTAACATTCGAAGTTTTTATTTGTTT	C	T	CTTAAATTCCCAAAAGTTTCCTTCGGCCCACAGCTCTTCAGTGAAAGGTGTCTGGACCGACGGATCTTGGGTGTTTTCGACTGGACTTGATCAGCGGGTCAGATGCTGGTATCTTCACCAAAGTAAACTAATTGAACGAGCACACCTAATAGTTAGTGTTCCGGAGCCAGAAGCGTTATCTGCTAGGGCTTGTAGCAGGAACCATTATCAGATTGCTGTTGCTGGAAG[illegible]	NA	NA
Ca-II-SNP399	25.79	Ca-*desi*-LG(Chr)3	1356024	ATAAAAAAGCACATTTTAATAAAGGGGTGCACCGACCTTGGAAAGGGGCTGTTTTTAACGGCTTTCTGACCGTACTTTCTCCATCTGTACCCATCTTCCA	G	A	TGTGGTTGGTATATATTAAAGGTTGAAAGTTAAGAAGACAAATGAGAAGAGACAGAGAGAAGCTAGAATAGCTTTCATGACGAAAAGCGAGGTGGATCAT	NA	NA
Ca-II-SNP400	26.76	Ca-*desi*-LG(Chr)3	1416213	GATAGTTTCTTTTTTCCTTTCATCATGTGACACTTAATAGGAATTTGCATTGATTTATTCCAGGTCCCTTGGGAATAAATTGTCCTTTGGATTGGGACAC	T	C	ATATGTTGGTTGCCTTGAAATCTCTGTGTATTACACACGGCTGTGAGTCTTCGTGCAGGTACGAAAGTCCTCTTGCAGCATCAAGAGCAATCTTCATTCT	NA	NA

Marker IDs	Genetic positions (cM)	Chromosomes	Physical positions (bp)	Flanking sequences/Forward primers	ICC 12968	ICC4958	Flanking sequences/Reverse primers	Annealing temperature (0C)	Amplified Product size (bp)
Ca-II-SNP401	27.73	Ca-*desi*-LG(Chr)3	1447555	CGTATAGGCAAATATGTGTGGCTTTTGTCTCCTGT<br>TAAGTGTTAACTGCCATTGCTTTTTTTATTTGGATG<br>ACTATTGCTGCTTTATTCTGATTCAATCC	T	G	CCCAAACTCCATCCATTACTTGGAGAGTCATCA<br>AATGATGCTGTTGGAACCTTGTCCATCAAGATA<br>AATCAGTTGAAAAAGCAAATCCAGGTGGAGAGA<br>ATTGTATCCATTCATGAAAAAATTCAGAGGAAC<br>CAGAAGAAGCTACAATGTCATATTTCAGGAATA<br>ATGTCAGCGGTATCAACCAGGGGCTCTTCACA<br>GATGGAGGAAAATAAGACACTTTCAATGCTTTC<br>[illegible]	NA	NA
Ca-II-SNP402	28.70	Ca-*desi*-LG(Chr)3	1525532	TTTTTCATATTCGTAAATAGGGGTGTACATGGATG<br>CAGGTCGACCCGTCAATCTGATCACCAAATTTGAT<br>TTTTATCCGTATTCGATCACTTTTGGGTCC	A	G	TTTAAAAATATGTAACTAATTATATCGGGTCGAC<br>GGATTCAAATTGTTAAACCCATTATCCGAACCA<br>AATATAAGCGGATTTCACCGGGTTGAGTTGAGT	NA	NA
Ca-II-SSR120	29.67	Ca-*desi*-LG(Chr)3	1602316	TGAGCAGCACACACTTAGGC	(GGC)7	(GGC)5	CCCAAGCCCATTTCATTTAC	60.21	100.00
Ca-II-SNP403	30.64	Ca-*desi*-LG(Chr)3	1618294	TTCTTGGTGCAATCTTCTCTAACCCTTAACTCCTTT<br>GATTTACTATATTGCTATATTTGTATATGTGATTAA<br>AACTAGATGCATGTTAAACATATTCTGT	C	T	CATGAGATTCTTAACCAGATAGTTTATGTTTAGT<br>GATTTAGTCATTTTTCAGTACTAGTTCATATAAA<br>ACCCTTTTGTCAGGCAGTTGATAATTGTCTGTC<br>GTTTGTATTCTATTTTGATTTGATTTGATTTAAT<br>TCTTGCCCTGAAAATAAATTAGATGCAAAAAGA<br>AGAAGATGGTTCTTCTGGGATGTGTTTCTTGTA<br>CACAAGTGGAATGTAATAATATAATAGAATTTG<br>[illegible]	NA	NA
Ca-II-SSR121	31.61	Ca-*desi*-LG(Chr)3	1632339	TTCTCGAGGATCCCTAATCC	(AG)6	(AG)7	TTTTAGATCATGGACTCAAAAGACA	58.13	103.00
Ca-II-SNP404	32.58	Ca-*desi*-LG(Chr)3	1639432	AAACGTGATGAGTTGCGAGTTGACGAAACAAACG<br>AATCGCGAATAGAAGGATGATAGCGACATTTTTCT<br>CTGATAGGGTTCTTAGTTTTTCAATTCTTTC	T	C	ATTATTTTTCAAAATAAGAAGTTGTGTATTATTG<br>TTTTAAAAAACTAACTTATTTTTCATTTCAAAAAC<br>AAAAAGTAGACTCAAGTCAAAATAAAAAGAG	NA	NA
Ca-II-SNP405	35.49	Ca-*desi*-LG(Chr)3	1705484	AGTCGGTTAGTCGGAGAAAGCTTCTACAGGCTGA<br>TGTTGGTGATGATGTTGTGGTGAGTAATATTGTGA<br>CAGTTAGTCAAGATGGAAGTGGAAACTTTAC	G	A	TAACATACTCTTCATAAACACCAGCCGTAACAT<br>AAATCAAAAAGTATCCATCACTAGAAAGAGATT<br>TATTCGGAGCAGCAGCAATAGCATCATTAATCG<br>T	NA	NA
Ca-II-SNP406	37.43	Ca-*desi*-LG(Chr)3	1803646	CGAATATACAATTTTTTCCATAAATCCATTACTTCT<br>TCTTGTCTATCCTTTTTCACATATTGTTTGCATCTT<br>GCCCCAACATTTTTTTCAATATGAAAAC	A	G	ATATGAGACTTTAATTTCTAAAGTTATTGTGACT<br>GATAGAGATCTTGCCATGATGAATGCCATTAGT<br>GTTGTGTTTCCTACTTCAATACATTTGCTATGT	NA	NA

Marker IDs	Genetic positions (cM)	Chromosomes	Physical positions (bp)	Flanking sequences/Forward primers	ICC 12968	ICC4958	Flanking sequences/Reverse primers	Annealing temperature (0C)	Amplified Product size (bp)
Ca-II-SNP407	39.37	Ca-*desi*-LG(Chr)3	1893159	GTAGATGAATAAGGAATGGTCGAATTTGCTGTGATGGAAGCCTATGGTGGAGACATAGTCAGCAAACCTCTGGTACCACGCTCGAGGAGCCTGTTTGAGG	T	C	CACGGCAATCTTCGTGAGACCATCTATATGCATCAGCCCATGTGTTTCGGCGATCCTGTTCATCCAGACTATGTCTGTCTTCTGCACAAATCATTATACG	NA	NA
Ca-II-SSR122	40.34	Ca-*desi*-LG(Chr)3	1900350	GAGCAGACTGTGAAGATTCTTTGA	(AAT)16	(AAT)19	TGGAAAAACCATAAAGAAATTGAA	60.06	176.00
Ca-II-SNP408	43.25	Ca-*desi*-LG(Chr)3	2647451	TCCAGAAGAAGCTACTACAATGTTAGATCGAATTCTTTCTCTTCTTACCTCCTCAACTGTTCTGTCGTTCAAGATCAACAAAAACTTGGATCCTATACAC	C	T	ACCAGGCATCTAAAAATATTTTATCATTAGTTAATGCTAACAAAGGAGCTAGTGAAAGACCATCAGAATTAGGAGCAAAGAATCTAGCAGCAGGAGCCAT	NA	NA
Ca-II-SSR123	44.22	Ca-*desi*-LG(Chr)3	2668158	CAAAGCCTGGTTCTTCATCA	(AAT)12	(AAT)11	CCGAGTCATAGCTACAGCGG	58.85	176.00
Ca-II-SNP409	45.19	Ca-*desi*-LG(Chr)3	2746674	TCGTGACGTGATCCATTGTGATTGTCATGGCATATGAAACGAGTCAGTCTCTCTATGTCACATTTCTACAAATGTAGATATGAGACCAATATCAACTATC	A	G	GGCTTACAGCGGAAGAAGTTTGTTGACGTACTTGTATCAAATGTTGTACAACATTGGATTCAATAATTTGGGCTGATACATTTGTCTTCAGTTTATTTGA	NA	NA
Ca-II-SNP410	47.13	Ca-*desi*-LG(Chr)3	2757852	AAGCAAAGAGTTCTGATGTTTCTAACTCTACTGGGATGCAGATACTTCAAAAAATGATTTCGCTTGAGCGAAATGAGTCTGGTTTGTCTCTGGATGGAAG	C	T	CCTCCAGCTCTTTATACAAAGCACTCATAAGTTTCCTGTCATGATCAACTTGTCTTTTAAGCTTGTCAACCAGACTTTCTCCTTCAATTTCGCTGACAAT	NA	NA
Ca-II-SNP411	48.10	Ca-*desi*-LG(Chr)3	2921225	AAATTTTACGTGAAAAACAACCTTCTCTTTGTTTGTTTCACTATATAAGTTTTTATTTCACTTGCAATATTACATATATTCTTAGCACCTCTCACCTGCC	A	G	AGAAACAAAATGGGGGGACTACTTGTGCGATTCAGCTAAAATAGGGAGACTAAAACTGCAATTAAACCAAATAAAAAAGAAATACAAGGACCGATGTTAA	NA	NA
Ca-II-SNP412	50.04	Ca-*desi*-LG(Chr)3	3074678	ATTTTCTTATCATGATTGACAATGTTTATTTTGTTTTCAAGGAAATATTGATTGTGTGTTTGTTATTTTATGAAATTCTATATCTTCTAAGTTATTTTTT	C	T	CCATCCATCTATATGTTCCAGTTTCTGCAGTCATCACTCCTCCCTGATTTAGGAACCGGGCAACTCCAAAATCTGCCACTTTAACAACCTAATTGAAAAC	NA	NA

Marker IDs	Genetic positions (cM)	Chromosomes	Physical positions (bp)	Flanking sequences/Forward primers	ICC 12968	ICC4958	Flanking sequences/Reverse primers	Annealing temperature (0C)	Amplified Product size (bp)
Ca-II-SNP413	51.01	Ca-*desi*-LG(Chr)3	3200806	CAAATTATTTAAACATACTGGTTGTGACTTTTGTGGGGGAGCACATAAAAATGGAGATTGTGAAGTACCGGATGATGATGCTTGTTCTTGTCCAATTAGG	A	G	CCCTAATTGGACTAGAATAAACCTAGAAAGGACGAAAATGACCTAAATGTTAATCCAATGACACAAATGAACTTATTGGATGCACAAGATTGGTTAAAAA	NA	NA
Ca-II-SNP414	51.98	Ca-*desi*-LG(Chr)3	3240315	TCTTCAGAAGATACATGTATTCACCTTTGAGATGTTGTGCAACTCCCGCTAATCTTTCATTTTTCATGCGTGGGACTAATTTACAAGAATTTGACTTTCA	C	T	GTTGTATTCAGTTGGCGTGCTGGGGGCAACAGGAACAAAACCCCTGCCACCAAAAACTGGACGCATAAATGCATCATCAAATTTACGCCATAAGTGATGAACAGTGTGTGTTGGAGTAGTTAGTAAATCACGAATGCTGCTTGGACCTTGTGAATCATTGACATCAGTTTCAACTTCTGAATCTTGGTTATCTCCAAGGAATGGCG	NA	NA
Ca-II-SNP415	52.95	Ca-*desi*-LG(Chr)3	3325103	TGAACACAATTTTAAACACAAATACATGTGACCTCCACATAATCTCTCTATTCATTTAATTTCTTTTTCTTTTATATTTCTTTTATTTATTTATCTTTTT	A	C	CGTAATAATAATTCTTTCAAAATGGTAGTATATGATAGATGGTGCATAAAGGTTTAGGTAAACAACTTGTCCATTTATTTATGATATACAAACAATGGGTTTGGTTGAACCTGCACAACCAGTTGCTATAAATTCAACCTCATTGAAGGAAGTAAACAGTTGCGG	NA	NA
Ca-II-SNP416	53.92	Ca-*desi*-LG(Chr)3	3495973	TGGCCAGAGGTGTCACACGGGGTTGGTGGAGGCGTGTGGGTCTCACGCCCAAATAAGGAGGTGGCGCGTCCGTATGAGTTTGGCAGCGCAAAAAATGTCA	T	C	CACTGCAACGCAAACCTTTAATATGGAGTCCTTTCAAACTGAAAGGCATGAAAAGTAGCAGAAAGTCATCACTCTTGCCATCTCGGCCTTCCATCCAAATGCAATGATCTTTGTTTTCTAAAACAATGACATATACTTGTAGTGTTTGGAATTAAATTGATTACTGACGAGGGAACGAAATCTGTGTTGAGG	NA	NA
Ca-II-SNP417	54.89	Ca-*desi*-LG(Chr)3	3654605	TCGCATTTCGCTACGTTCTTCATCGATGCAAGAGCCTAGATATCCGTTGCCGAGAGTCATTCTATATTTCATGTGTCAAAAACACAACCCGCACGAAAAC	T	C	CCCAATTGTGTTCTCCTCTGCGCCAAAACTCAAACCCCGACGCTGAATGCGTCAAGGAATTAAAACTTTGTGATGTGCGCACCTGCATGGAACCGGAGAC	NA	NA
Ca-II-SNP418	55.86	Ca-*desi*-LG(Chr)3	3765014	AAAATTCTTTTATATATAGAGGATTCTACTCGATTCATAAATTACACTAAAACTACTAAATAAATTCGAGTTTTCTCTTTTCTCCCTTTTTTGCTTTGTC	C	T	ATTATGGCGATTAAGATTACTCGTCGTAATATAGCACTATGACCGCTCGAAAATTTGAATGGACAGTTTTTTTTTCAATAAGGAACAAAAAGAAAATTTG	NA	NA
Ca-II-SNP419	56.83	Ca-*desi*-LG(Chr)3	3879405	CGTGTGTGTTATTTGTCTAACTTCTTTCATCAAAAAATTCCCCTCTACTCATTTAATTCTCAAATATATTTTTAGTATCGAAAAGTTGCTTAGCATTAGG	G	A	AAAAAGGTCAGGTCAGACTAAAAAAAAAGTCTATGATAGACTGTAAGCCTAAAAAATAAATCGTAAGACTCAGACATTTCAAAGTCTGGCCTATTCCCAC	NA	NA
Ca-II-SNP420	57.86	Ca-*desi*-LG(Chr)3	3943513	ATTAAGAATTTGAAATTGAATTGGAATTAGAAATGGAATCCTCCCTCTTTTTTTGTGGTTTTTATTTTTATTTATTTTTAATGGTAATTATGTATGTGAT	C	A	GTAAAGGCAGCACACAGTATGTTGTTAAGGGATCACAGAAAGAGTTTCCACAAGAGCTTCTTGAGGAGTTGAAAATCATCTGTAAGGATAACATATCACTTGATTATGACGAGAGATATATCCATGGAAAACCACAAAACAGTTTTCACAAGGCTGTTAATATCCCAGATGTGATTGTTTATCCAAGATCTGAAGAGGAGGTCTCCAAGATTGTCAAAGTATGCAACAGTT[illegible]	NA	NA

Marker IDs	Genetic positions (cM)	Chromosomes	Physical positions (bp)	Flanking sequences/Forward primers	ICC 12968	ICC4958	Flanking sequences/Reverse primers	Annealing temperature (0C)	Amplified Product size (bp)
Ca-II-SNP421	58.89	Ca-*desi*-LG(Chr)3	4071550	GCGGGATAGGAGAGGGAGCAGGAATGACACGAG<br>AAGTTAGTATAGTGTTTGTGTGCCCCACTGTAAGT<br>TTTGTCTCTGCATCAACTACAACACACTCAAA	T	C	CAGGAGCCATTACACAACGAAATGGCGGTGAA<br>ACTTGGTTTGAAGTTGGACGATCAAAGACATAT<br>ACCCCATCAGCTGATGACATTGGTCATGTCCTT<br>AA	NA	NA
Ca-II-SNP422	59.92	Ca-*desi*-LG(Chr)3	4112099	CCAAAAGCTATTAAACCATGTCAATTTTTTTGGCC<br>TAATGATGTGATTTGTGATGAATCATTATTTGCAG<br>TGTTAGTGTGTTTGGTGTCTCAGCAGAATC	A	T	TGGTTACTACCTTTAGGCCTTCTTGTGAGTATA<br>ATCTCTCTTGCATTAGTAGCAGAATTGTGGGGA<br>CGCTGTTTCCTTTTGAATCATACGAACATTGCA<br>T	NA	NA
Ca-II-SNP423	60.95	Ca-*desi*-LG(Chr)3	4198788	TTCGATTTACAGCTGTGTGGTATATGTAGTTACTC<br>CTAGTTTTTGAATTTACCTGGAACTGTTTCTGTTAA<br>ATAATGGTCTGTATGTTGTGACAGAAACG	G	T	CATTGTAGACGCCAATAATGCTACCAATCATCT<br>CAGGCACAATAATCATGTTGCGGAGGTGAGTC<br>CTCACTGGTTCTGGCTTCTCACCAGCTGGGGC<br>CTC	NA	NA
Ca-II-SNP424	61.98	Ca-*desi*-LG(Chr)3	4227862	ATAGAGCATTAAGTTTACTTAATTTTTTGGTGAATG<br>ATTTGAAGATTTTGTTAGCAATATGAGGCTTCACA<br>AGCTCCTGTTTCATAGAAACATGATCTTT	C	T	GAGCAATTTCTCCTAGAGATAACTATGCATCAG<br>TAAGAAGAGAGAACAGAGAGCTGAAACTTGAA<br>GTTGCAAGAATGAGAATGAGATTGAATGATTTG<br>GA	NA	NA
Ca-II-SNP425	63.01	Ca-*desi*-LG(Chr)3	4416213	AACTTGTCGCTGGATCATGGACTTATTCAAGGACC<br>ACAACTTTGTGGCCCAACATTGTATACGGATACAA<br>AGATATTTGTACCATGTACCCCCACCTATT	G	C	ATAATATGTTTGAATATATTGGTTTTCACGGCAA<br>AAAGTCACGGCAATGTGAAAGCTACAGCTAGAA<br>GCTTCACTATCATCGTACATTTAGAATTGGGTG<br>CTCCAATCAATTTACCAAACACATACTTAATCCC<br>TGATCCCAAAAGTCATGCACGCTTTATAATAAC<br>CAAGTATCCAG	NA	NA
Ca-II-SNP426	64.04	Ca-*desi*-LG(Chr)3	4441610	TCTATTACGCTTCTATATTCATACAGATCTATTGTA<br>TATCTTCAACAAATTTATACTCTCACATATCTCTTC<br>TGTGCGAGAATTTGTGCATGCGGTTTTT	G	T	ATCAGAAAAATAAATACCTTTTCTCTCCCCCCAA<br>AATCAAAACCCTAACCTTCGAAGCTCTCACGGA<br>AACGCCACCGATCCCCATTTCCTCACCAATCCA<br>AGAAAAAAATGAGTAGACATTCGAGCAGAACTG<br>TCTATGTTGGGAATCTACCTGGCGATATCCGTG<br>AAAGAGAAGTTGAAGATTTGTTTATGAAGTATG<br>GACACATTACTCATATTGACCTAAAGGTGCCAC<br>[illegible]	NA	NA
Ca-II-SNP427	65.07	Ca-*desi*-LG(Chr)3	4469690	GTCATTGTTCAAACAACCGTTATCTCTCCTGTTTT<br>GGACGCATTTTCTTACTTTGATGATCATGGCTTCA<br>TTTCGTTAGAGTTGCGCTGCTTCTTCTTTG	G	A	AATTCATATGTGGAAAAAGAGACACCACCAGAA<br>TGATCGTCGGTGGGAATAAAAGCCACTGTTGG<br>AATCTATTAGTAAGAACTAAATTGCATGACAAAC<br>A	NA	NA
Ca-II-SNP428	66.10	Ca-*desi*-LG(Chr)3	4548319	TAAAACCATTTTGTTTTTCAGCAATTATCTTGAACT<br>TTTTAAGTATTTCAAGCACTTCATTCTTTCTCTTGA<br>TTAGATATACCCAAATTTTCCTTGTAAA	G	A	GATCTTTAAGAATGATGGAGATAATCTACTCTG<br>ATGTTTGTGGTCCTTTTGAAGAAGTATCAATAG<br>GAGGTAATCATTACTTTGTATCATTCATTGATGA	NA	NA

Marker IDs	Genetic positions (cM)	Chromosomes	Physical positions (bp)	Flanking sequences/Forward primers	ICC 12968	ICC4958	Flanking sequences/Reverse primers	Annealing temperature (0C)	Amplified Product size (bp)
Ca-II-SNP429	67.13	Ca-*desi*-LG(Chr)3	4631479	CAAACTTTTGATGCCCTTTAACATGCATGTGTTGTGTATAGCCAAGTCCAGTTTTAACATTTAGATTTCTTTGATTTCTAAGAAGTCTATCTAAGTTGTT	T	C	AGATTAATGTGAAGCATGAGTCTCTTAGAGACATAAATGAAGAATTGAAAAAAGAAGCATGTGCATTGAAATGTGATGTAGCTAAGTTTAATTGTGGTAA	NA	NA
Ca-II-SSR124	68.16	Ca-*desi*-LG(Chr)3	4650196	AATCCCTTCACACACACAAAA	(TA)12	(AT)14	TGATTCCGTGTTTCTTTTCG	57.97	114.00
Ca-II-SNP430	69.19	Ca-*desi*-LG(Chr)3	4673307	GATTGAGTCAAATTCATGATACTTCTTTAGTTCTTTGTAAAATACAATTCTTTGTAACTGAAGGTTTAGAAAATCCAGTTTGGAAACTGGTGGCTATCTT	T	C	CTCTCTCTATGCTATTCGGCGACCACTGTATCCCAGAGTTCCTCTACAAACTCTTCCAAGATCACAGTAAAACTTCTTCTTGATGAGTCCTCTCAACAAC	NA	NA
Ca-II-SNP431	70.22	Ca-*desi*-LG(Chr)3	4785882	CCAAAGAAGCTCTTTCATATGTCCTCCAGGATACTTTTTTTTCCAATTCCCATACAAGTGTTTAACACATAATCGGTGTTCCACATGGGCCTCAATGTTG	A	T	CAGGGATCAAATCAACATGAAAGAAAGCCGTATATTGTCTACATGGGAGAACTACCAACTGCAACTGCAACAACATATACTACGGAACAACACCACCACAACATGTTGGAGACTGCGATTGGAGACAAGCAGTTAGCAAGAGAGTCCAAAATACATAGCTATG	NA	NA
Ca-II-SNP432	71.25	Ca-*desi*-LG(Chr)3	4832402	TAGAGTTCCAAATAAAATTAGGGTTTCAATGAATACCGGCGGTGACGAATCGGCGATTGTACTGCATACGCTTGTGAGCACGGCCACGTGGCTTCTTCTT	G	C	GTTTGCACGGCTTGATATTTAAGACTTGAGGTCTTCTCTAGTTCCGGTGGTATCATAGCAGGATTAGAGGATAAAACTGTAACTGATGCTGGAATTAGTGGAGTAACATCAACAGTCAAGTCAATTTCGCCGACCAAAGAGCACTTCTCCAATAACTGAAAGAAACCGGTTGTCCTGATATTCAAAAGAAAAGCTGCTTTTAATATTCGTAAAGAAGGAAAGGACTGGCTG[illegible]	NA	NA
Ca-II-SNP433	74.34	Ca-*desi*-LG(Chr)3	5774493	CAACACATCTTTGATCAAGGAATGACCACCATCCCTTCTTATGATCAAAACATCCCCGATACCGTCGGCCGCTAATGTCGTGTCATCCGCGAATTTCACT	A	C	GCCCAAGGGAGAAATGAGTGGTACTTGGACTCAAGTTGTTCGACACATATGACGGGAAGAAATGATTGGTTCGTGAATATCAATCAAGCCACGTGAAGTA	NA	NA
Ca-II-SNP434	75.37	Ca-*desi*-LG(Chr)3	5913806	GTCTCTTCGTTAATTTTAGGGAGCATCTCCAGCAGAAATGATGATGTATTATGTATTGGGCATACCTCTACTCGACGTGCATATCAACTATCTAAAACTA	A	G	CCCTTTTCAACGCGTTTTTCTTTTATCCATTCTTCGCTTCTTCATCGCCTTCATCACAATCATCTATTATGGATTAATTCTCATTATAGATTATCTATTCCTATTCTTCTCTTTATAGATCTATTTCGCATGTTCTTCGATCTTCGATCGGTTACGATCGAACAGAGAGTTTTCGATTTTTGTTCTTTCTTAACAATTAGGGTTTCGACGAGCACAAAATGATGCCTACCGTGC[illegible]	NA	NA
Ca-II-SNP435	76.40	Ca-*desi*-LG(Chr)3	5916065	ATCTAACAGCGGTTTGGATCTCCCGAGAAGTAATTGTAGGTTTTTTGTTATACCTAGCCAAACGGGAAGATTCTTGAGCGAGTTTCTCGAATATATCGTT	G	A	GCGTTGAGACTTACAAGATTTACATCTTTAAGGTCTTGAAGCAGGTTCACCCTGATATCGGGATCTCCAGCAAGGCTATGGGAATCATGAACAGTTTCAT	NA	NA

Marker IDs	Genetic positions (cM)	Chromosomes	Physical positions (bp)	Flanking sequences/Forward primers	ICC 12968	ICC4958	Flanking sequences/Reverse primers	Annealing temperature (0C)	Amplified Product size (bp)
Ca-II-SNP436	77.43	Ca-*desi*-LG(Chr)3	6090281	TTTTAGTTTTGGCAGTTATGATCTATAATGGGCATGCGGTGTCCCACTGTCTATGTACGTGGCTGTCCTCTTGTGGTTTAGGTACACACAGACAGGCACA	T	G	TTGAATATAACTAATGGAAGTAAAATTAAGGGATAAAAAATGAAGAGACTAAAACTACAAGAGCACATGTGATATATAAAAGCCAAGGAGTGGTAAGCAC	NA	NA
Ca-II-SNP437	78.46	Ca-*desi*-LG(Chr)3	6159447	CTATTTCAGGTTTCTTGCTGGAAGAAAATTCTACTTCGTACTCTTCGTTGTCTTGTATTACTCCTTCACACCACTCCTCAATCAGCTCCTCCAGGTAGTA	G	A	AGCTGCAGTCTGCATTGATGCTACATATAAGAAAATGGTCAGAATAATTGCTTCAGTTATGAACTAAGTAGTGTATGGGTTATAGGTACAAACCCAATGA	NA	NA
Ca-II-SNP438	79.49	Ca-*desi*-LG(Chr)3	6259404	ACAGTTGTTCAAATCTGAGACTTTAATTTCTAAAATTATTGTGACTGATAGAGATCTTGCCATGATGAATGCGATTAGTGTTGTGTTTCCTACTTCAATA	G	A	CAACACAAGAGTCACTGAGTGAGTGAAGCGTTACACCCAAAAAAAATAAAACTTCCATTTTCCCTAACCCTTCAAATCAAATTTCGTAATATCAAAATTACACTCTTCGATTTCCTCACATCTTTAACAAATCTCTTCTCGATTCAAACCTAATCAAATAAAACCCTCAAACACCTGTAGATCTGTACAAAACACCCTCTCATCCTCCTCAAATTTTCCTCCTCCAAATTTTCTG[illegible]	NA	NA
Ca-II-SNP439	80.52	Ca-*desi*-LG(Chr)3	6298304	TCCTACTCTGAAACAATATTCTCTGAATGCCCTACTTTTTCGTTCCTTCAGGAAGTCGTTCCCTGGGGAAGCGACCTCTTGAAGAGGAAATTTTTTAATG	C	T	TAAATTAATAATTCTAATAATAAACTAACTTTGAAATATAACAAAAAAAAAAAACAAAACCTAGTGGGAGGTCGCTTCCCCAGGAAACGACCCCATGTTA	NA	NA
Ca-II-SNP440	81.55	Ca-*desi*-LG(Chr)3	6427132	CTCATGTGTTTGCAAAAGTTTATTTTTTTAAAGTGCGAACGTTATGCATGCAGGTATCTTCTATTGTTCATAATCACCTGTCTCAAGCAAGGAGGGTCCC	T	G	ATGAAACACAAGCATAAAATAAAAATTCAAAACATTAAAAAATTCAAAGCCTTAAGAAATACTCCGTAGCGGGACAAAGAGGCCCCTCAAATATGAAAAG	NA	NA
Ca-II-SNP441	82.45	Ca-*desi*-LG(Chr)3	6541733	TGGTTTGTCCATCAGACCAATGAGTTATGCTATGTGTTTGTTGTTGTTCTGCCTAATTTGTTATTCGTGTGCAATTGCTTGACATTGTGTGAAATTGTCT	A	G	ATTGAAACAAACAAATAGAAAATGACTCACAAAGTTTTAAACAATTCATATAGTTTTATAACAATTTCACACGACTACGTGATATATAACACACAAACAT	NA	NA
Ca-II-SSR125	83.35	Ca-*desi*-LG(Chr)3	6739764	GGTGAAACAAACTCAAACTTGG	(AG)6	(AG)7	TCCATCCAAACCTACTGTCCA	58.65	142.00
Ca-II-SNP442	84.25	Ca-*desi*-LG(Chr)3	6811546	TTACTCTTGTCCTTGTTTTGTGCCAAATACAAGAACCCAAATGCACCAACTATAGCCCCGAGTTTCCCTGCTGCTGATGATATTCCATGACAAGTAGATC	G	T	AGGATTTGTTGCATTGTATTCATTGACCTTCTTCTTTGCAAATTTTGGGCCAAATGCTACCACGTTTGTTGTGCCCGCAGAGATTTTTCCTGCTAGATTT	NA	NA

Marker IDs	Genetic positions (cM)	Chromosomes	Physical positions (bp)	Flanking sequences/Forward primers	ICC 12968	ICC4958	Flanking sequences/Reverse primers	Annealing temperature (0C)	Amplified Product size (bp)
Ca-II-SNP443	85.15	Ca-*desi*-LG(Chr)3	6830375	TTCCCGATTTTGTTAAATCAGTCTCACAAAAACAT CGATTGCGCATTCATTAAGCGTTGGATCGGGCTG ATTTTTGGACAGAAGGTTCGCAAAATTCAGG	C	T	ATCACCGAGACAAAATAAATTAGGGATAATCTG GTGCTGTGCGAGCCCGATTTCTCCTCCTACAG ACGTCGTTTCGCCGATCCGACCGTTGAAGACG AAG	NA	NA
Ca-II-SNP444	86.05	Ca-*desi*-LG(Chr)3	6859122	GGAATTAGCATATAATTTTAATTGTCCTTAACTTAA TTTCCGGAACTATGATATTGCACCTTACTAGTTGT GGAGCGATTAAGTTCATGATTGACCCGAA	C	T	TATGATTAAAAATATTAAATTTAAATCTACGACA TTTGATTTTGATCAAATGGCTACTAATATCTCGA CTTTAAGAATGCAAAAAATCCCTGCTATAGGA	NA	NA
Ca-II-SNP445	86.95	Ca-*desi*-LG(Chr)3	6877359	AACACGAAGTAACATATCTTGTTCACCTTGCTGCA AGGACCACCCCGAAAGGTCTTCATTGTCTTTCTAT GCAACTAACTTCTGATTACTTTGCCCTGAG	A	G	CAACCACCGCACATGCCAGAACATTGTCAGAG AAGACAGCATAATGATGAAGTTCCGTATCATGA ATCTTATTTTCATTTGGCAGCTTTCTATCCTCG GG	NA	NA
Ca-II-SNP446	87.85	Ca-*desi*-LG(Chr)3	6921110	TAATGTATTGGACAGGTGAATATTCAGAAAGGGAA GGGTACAATGGATCTGAATATGCTCCTACTTATGA GGACAAGGATGGTGATTGGATGCTTGTTGG	C	A	ACTAATTAATTTACTGATTTAATTAAAAGAAGCT GATTTAATTATTTATATATGATGATGAGGGGAG AAATCAATTAAGAACTTACTTCCATGGAACATC	NA	NA
Ca-II-SNP447	89.65	Ca-*desi*-LG(Chr)3	6978618	CCCCTTAAAGTTGACACTAAATCTGGGTGTCACTC AAAGTATTATTGACCTTTACCTACAGTTTATAACTT TTAGTGACACATTTTGCATGTCACGATAC	A	G	TTCGGAAAACTTATGGTTTTAAATTTTTCAGTAA TGGTTGTGTTTCGAAAATGTTGAATTTTATTTTA TTTTTTATTATAGTTCACTACAAAAAAATTGA	NA	NA
Ca-II-SNP448	90.55	Ca-*desi*-LG(Chr)3	7029469	TGACTTACTCGTACAAAGAAGGATTTGATTTGTTA AATCAGGAGGTAGATAGAGCATATCTTGTTCTCCA TGCTTCTTTCTCTTCTACAAGTGTTGTTTC	C	T	CTCAGAAATTTCAACTCTCTATTTATTATAATAT ATAATAAATCGCATGATTGATAATTATTATTATT ATAGAAACTAAATCATGTGGATCAAAAAGAAG	NA	NA
Ca-II-SNP449	92.35	Ca-*desi*-LG(Chr)3	7114370	GTATATAGACACCAATTGATGCAGACTCAACCTAA AACCTATGTGGCAACTCCTTTTATTTCAACATATTT CTAGTCGTGTCTAGGATGGTTCTATTTCT	T	C	AATATACATCACATGAGTTTGAGGAATTTTGTAT CAAAAATGGGATTGAACATCAAGTGACTGCCTC ATATACACCTCAACATAATGGATTGGTTGAAAG	NA	NA
Ca-II-SNP450	93.25	Ca-*desi*-LG(Chr)3	7207094	ACAGTTGTTCAAATCTGAGACTTTAATTTCTAAAAT TATTGTGACTGATAGAGATCTTGCCATGATGAATG CGATTAGTGTTGTGTTTCCTACTTCAATA	G	C	TAATAATTGTCTGTTAAAATGACGACGGTTCCT GTATTAGCGGTGCCAATTTCTGTCCTTCTCCGA CGACCTTGTTCTCCTCTTCTATGTCTAACAACT CAGTCTCACAACCATCATTTCTGCAATCCCATT TTCTCAAATTCGTCAAACAAGAAAATAACCGTA GTAGTGTTTGGTGTGAATCCCAGTAACTCAAGA CAAGAAAATCCTCTCTTCTTGGACGAAAACAAT [illegible]	NA	NA

Marker IDs	Genetic positions (cM)	Chromosomes	Physical positions (bp)	Flanking sequences/Forward primers	ICC 12968	ICC4958	Flanking sequences/Reverse primers	Annealing temperature (0C)	Amplified Product size (bp)
Ca-II-SNP451	94.15	Ca-*desi*-LG(Chr)3	8442587	CTATTTTGGAACTTTTGCTAGGAAGGAATCTTGTATATTGGAACAAAAGGCTTTGTGATTATTTTATATTTTGAAATATTTGCTCACAATATATATTTTG	C	T	ATATACTTGAGAAAATTGTTATGCTACTAAATAAATATATATTATAAATGAGCTAAAAAATATATATTATTAATAAGCAAAAACTGTATATTATTAATGA	NA	NA
Ca-II-SNP452	95.05	Ca-*desi*-LG(Chr)3	8518037	AGGGGCAAGCATAAACTCATACGTGAACTGAGTTCACGTTCAACAACCTACGTGAACTGTGTTCACGTACTTGAACTCCATCACCTACGTGAATTGAGTT	C	T	AACTCAATTAACGTAGTCTGATGGAGTTCACGTACGTGAACTCAGATCACGTAGGTGGACGTGAACTCAGTTAACGTAGGCTGATGGAGTTCACGTACAC	NA	NA
Ca-II-SNP453	95.95	Ca-*desi*-LG(Chr)3	8807727	CATTTCCTCTTGGATGGCTTCTTTCCAAAAGGAAGAGTCTTGAGACTTTATTGTCTCTTCATATGTGTTTGGGTCACTCTCTAAATTGTAGCAAATTGGT	T	C	GAACCCAAAAGAAGTAAAAGAGGCAGAAAAGAAAAAGATTATGGCCTAGACTTCTTTATGTTCCTTGTAGAAGGTACAAATGATTCCAGTAACAGTTATG	NA	NA
Ca-II-SNP454	96.85	Ca-*desi*-LG(Chr)3	8974312	ACCATTGCCACGAGTTTCTTGTTTCTTGTTCATCTTTACTTATTGTAGCTATTAACTGGCCACCATAGTCTCCCTTCAAGAAACATGCATCCAACCCAAT	G	A	CTATAAAGGCTGAAATTAATGTGGCTACAATGTTGATTATGAATCCTATTCCCATTCTTTGTATGCATGTGATTCCAGAAGGATTGTTTGTGAATCTACGAGTGAACGGGACGAAAAGGCGTTCGTATAGAATAACTCCTATCATTGTAGTTATGACACTGAAAATTGACATGTTGGCTGGTGAAATT	NA	NA
Ca-II-SNP455	97.75	Ca-*desi*-LG(Chr)3	8989700	GTTCGTATAGAATAACTCCTATCATTGTAGTTATGACACTGAAAATTGACATGTTGGCTGGTGAAATTTGGAATGAGTGAGAGAGGTGACGATCCATTGT	T	A	AACTAAAATCAATCATAAGAATCCTTCCAATTTCATCATCAGGAATATTACTCATAGCTGCTTCAGCACACCTACCTAGTTTCGTCATTGAACAAGCTCG	NA	NA
Ca-II-SNP456	98.65	Ca-*desi*-LG(Chr)3	10086662	TTGGAACTATTTCTCCATCCTCGGAGTCCTGATCCTTGAGGGATCAAAGAATTGGCAATTTTAGTCGAATCATTTTTATCATTAGCCTTGCTGCTCCCAT	T	C	TTCAACACACGTCAAAGGATGCAAGTAATAAAAGTACAAAACTATCAAGATGAAGGAAGATCCACCAATATGGTAGATGCAAATTTGTATGTTCTAAAGGAGAAGATAGAGATGGTGAAGGTGAAGGAAAGATTGGAAAGGTGTTGCAAATGTCAACATGGTTGGAATTATGTCCCACTTTCTATTAATGATTATCAAAAAACCAAAAGAAACAAAGAATTGAACAACTTC	NA	NA
Ca-II-SNP457	99.55	Ca-*desi*-LG(Chr)3	10106267	AATCCATGTATGGCCTCAGACAAGCATCGCGACAATGGTTTTGCAAGTTCTCCACCACCTTGCTTGCTCATGGTTTTGCTCAGTCTAGAAACGATTATTC	C	G	GAGTCTCAACAATTTTAGTGGAATCTGGGCCTGTCAACACAATATCATCTACGTAAACAAGTAGAATGATCAGGGAGGGATCTGAACCAGAAGTAAATAA	NA	NA
Ca-II-SNP458	100.45	Ca-*desi*-LG(Chr)3	10384363	ACAAGTTACATAGTTTTTTACAATTAAGTGCTATGTGAATTTGTTTGTGCTTTGCCTGATTGCAATGTGAATTATGCTTTTTCTTTAACTCTCTGATTTT	C	G	TGGTTCGAAAGAATCTGAATTTAAATATTGGTCGGAATAATTTTTTGTCAGACTATTTACCTCTCGATCAAATTCTGCATTGTGAGAACCCCAATACTCT	NA	NA

Marker IDs	Genetic positions (cM)	Chromosomes	Physical positions (bp)	Flanking sequences/Forward primers	ICC 12968	ICC4958	Flanking sequences/Reverse primers	Annealing temperature (0C)	Amplified Product size (bp)
Ca-II-SSR126	101.35	Ca-*desi*-LG(Chr)3	10464064	TTCCCCACAAATTGAAACTACA	(TATT)5	(TTTA)5	CTCTCTCCTGCACATCTCCC	59.36	201.00
Ca-II-SNP459	102.30	Ca-*desi*-LG(Chr)3	10490580	CTCCCCAGAATGTTTTAGGCAGCCCTACACTCAAGATCATACATCTGACCATTTCCAAGATTGTTATGTTCATCCTTTCAGCAATACCATTTTGTCAAGG	A	G	AATGTTTAAGAACTGACAATGGCCTGAAGTATCTTTCAGAGCAGTTTAATAAGTATTGTAAGGACTTAGGTATTCAAAGACATAGAACAGTTGCAGGTAC	NA	NA
Ca-II-SNP460	104.20	Ca-*desi*-LG(Chr)3	10590044	GGAGGGAGAACGCAAGCTAATTGATCACCACACACGCGCGCGCCGCCGTCCATCCGGCCTGGCCTGGCTTGGCTTTTGCTTTGTGGTGTATTATTATTTT	T	G	ATATGACTAGGTGGAAGGCAGTTACGTAATTAACGTTTCAATTAGAGATGGAACGCAAGTAATCAGATAATTAATTAAAATTGATTAATTAATTTTCAGG	NA	NA
Ca-II-SNP461	106.10	Ca-*desi*-LG(Chr)3	10664843	ATATTGATGAATTAGGTTATTCCCTCTGACTTATGGTCAAGTCATACATATACCGTGTATGTTTGGTTTCACCGTGAGAATGGCAAAATCCCGGCGACTC	A	G	GGGGGTTTCTTAACAGTATGATGCCAATTAATGTTATATTTGCCTGTAAACAAAAGGAGTATATGCTTAGTTAGTTTCACGGTGGGATTACAAATCATGG	NA	NA
Ca-II-SSR127	107.05	Ca-*desi*-LG(Chr)3	10687153	GAAGATGTGGGGGACTACGA	(AAT)19	(AAT)15	GGAGGAGAAGAAGAACAAGAAGAA	59.93	232.00
Ca-II-SNP462	108.00	Ca-*desi*-LG(Chr)3	10723365	TGTTCCAAAATATTTTTGGGAAGAGGTTGTCCTCACATCAACATATCTTATTAATCAATAACCTTCTAGAGTCTTGGGGTCCAAGAGTCCTATGGATGTT	A	T	GAAAATGATAATGGACATGAATTTGATGATGAAAAAAGGGAAATTGCTCAGAAAAAGTTTGAGCAATCTCATGTCATCAAAAAAATGTGACACAACGTAATGGTGGTTTTATGGTACATGATTATGAATTCTCTTGTAGTAATAGCCCAAATCCTGTTTTTTTCAACATGCCCAAAAGGAAGCACCATTTC	NA	NA
Ca-II-SSR128	108.95	Ca-*desi*-LG(Chr)3	10755951	AAAATAGAGTCGTTGGATTCAAAA	(AT)11	(TA)11	TTATGTTATCAAAATCCGTCAAAA	58.33	142.00
Ca-II-SNP463	109.90	Ca-*desi*-LG(Chr)3	10776247	TACATAGATTGACTAGAATTGTCTGTAACATTTGGACAATATTTTTTAGTATGACTATTTAACCGACATAGTCCGCATTTTTTTGACATTTTTTCTCTAA	C	T	AGAATGATATTGTCCCTTTGAAACAGTGTATCACAATACTGAAATGCGAATAATTAAAACAAGTCGTCCAAATTGTATTCGTATTAGAACTGAAATGGAC	NA	NA

Marker IDs	Genetic positions (cM)	Chromosomes	Physical positions (bp)	Flanking sequences/Forward primers	ICC 12968	ICC4958	Flanking sequences/Reverse primers	Annealing temperature (0C)	Amplified Product size (bp)
Ca-II-SNP464	111.80	Ca-*desi*-LG(Chr)3	10809526	GATTTAAAAGGCTTTAACATTTATATATGCAGATAGAGATATAGCTTTAACATTTAGATTCTTGTGATGTGCTCTCTTTCTCTCACTGGTCATTACATTA	C	G	GTGTGTGTGTGTGACAGTGAAATGATAAAATATGAGTTTTAGAAGTGGAAGAAAATGTTTTCTAAATATTGCTTTTGGAGGTTAAAAAGGGAAAAAAGGT	NA	NA
Ca-II-SNP465	115.60	Ca-*desi*-LG(Chr)3	11051176	CTGTTGGTGTGGCAGCTTTGCCATCTGGAGATCCTGTTTCGGAAACGAAGAGGAAGTTGGAATATCTTGAAATGCAAGAGGAGCTAATCAAGGTTAATTT	G	T	CAAATCACTTTAAAAATATAATAATAGAAAATGGAAATCACCAACTCATGCCTCCTTCGCAAAACATAACTATCTCAGATGATGGCAAATGTGATGCATA	NA	NA
Ca-II-SNP466	116.55	Ca-*desi*-LG(Chr)3	11138620	TTGTTCAATTTTTTAGATTGGCCAGCAAAGATGACAAGGGTGGACATTGATATTATTTGTATTGATTTTGTATAAGGACATTGGTCATTTAGTTTAAGAT	T	C	ACTCCACCAATTTATAATTGAAATGCAAATATATTTATAATTTTAACCATTAAAATGTAAACTTAATATACATTGTACATTCATGTTTATAAAATCTAAA	NA	NA
Ca-II-SNP467	117.50	Ca-*desi*-LG(Chr)3	11161639	TGTTTTGTGTATACACTTGTCTGATTTTGAAAGAACTTGATTATGTTAAACTGATTTGGTTAATATTGAGTTTGAAGGAGAACAATTTATGTTTGGATAT	C	G	ACATTATGGCATAAGATATGGTATATAACATTATGGCATCATTTGGTTCATGTAGCCGAATCAACTTAGTGGGATAAGGCTCAGTTGTTGTTGTAGTATA	NA	NA
Ca-II-SNP468	118.45	Ca-*desi*-LG(Chr)3	11201949	TGGTTTGTCCATCAGACCAATGAGTTATGCTATGTGTTTGTTGTTGTTCTGCCTAATTTGTTATTCGTGTGCAATTGCTTGACATTGTGTGAAATTGTCT	A	G	TTAGCATTCATGCTTTCCAGTCTGTTGAAAACTTCAAATTTGGCCTTTGGCATACAATTAGCATCAATTTTTTTGATAATAACGTGTTCCGGGATGGAATTCTAGATTCAGCTGCTAATTTTGGGTTACTCGGCTAGGGTTTTTTGAGTCTCCTATGTGTTCGGGCTCCTTTTGGCCTATCTTGCCTGGGACCCTCAGACTTTGGTCAGAGCAATCGGGCTACAGTGTAAT[illegible]	NA	NA
Ca-II-SNP469	119.40	Ca-*desi*-LG(Chr)3	11267039	CACATTTAAAACTTTTTTCTATTAATGGTAGGGGCGTACAAGACCCGGTTCGTCCCGCGAATCCGACCTGACCCATAAGCAACGGGTTGTGTCTGACCCG	A	G	TGTATTTTGGGATGGGACAAGTAACCCGGAGGATGACTCAAACCCGATCATTTTTTTACCTTGTCTATGTCCTGTCTTTTTTTTGTCCTTGTTTTTTTTTA	NA	NA
Ca-II-SNP470	120.35	Ca-*desi*-LG(Chr)3	11294090	GGTAATTCCTTTTCCTCTGTTTTATGTGCTTTCCTTTTGTTTGGTTTAACTTGTTTACTATTTAACGGTCAATATTATTGGTCCCTGGTTCAAAGTTTAC	A	G	TTAAGTAGCATTAAGAATATTTTATAATTCATTTCATGAAATTCACATTAACCATCATATGGCTATCAAGACAAGAAGAGTATGTTTATATCCAACTGTC	NA	NA
Ca-II-SNP471	121.30	Ca-*desi*-LG(Chr)3	11306404	AAATATTAATTTTTGTCCTTTTCTTTGTCGGTAAATTGGATGGTACGTACACTTGCATGTGTGCTGTATGTTTAACGGCAAATTATTTGACTCAAAATAA	T	C	GTTGCAACAAATATGTGGGAAGTGACGTTGACTTTATCTTAATTCCATTGTTGGAAGGAAAAACTCATAAGTTGATAAAACTCATTTGCAATGATTTGCC	NA	NA

Marker IDs	Genetic positions (cM)	Chromosomes	Physical positions (bp)	Flanking sequences/Forward primers	ICC 12968	ICC4958	Flanking sequences/Reverse primers	Annealing temperature (0C)	Amplified Product size (bp)
Ca-II-SNP472	122.25	Ca-*desi*-LG(Chr)3	11324258	ATACATTTCTAAGGCATCATCCTTCATAATCACCTGAAATCCTTTCTGAATTAGTTGTCATATGCTCAACAAATTGTTTTTCATTCTTGGCACATACAAC	A	G	AGCAAGGTCAAGTTTGCTGATGACAGAACCTTGAAAGCTGAGGAAGTTGACAACATAGTGATCAAGAGAAGAAATGGCAAAACAGCGGTGATTGAAAATG	NA	NA
Ca-II-SNP473	123.20	Ca-*desi*-LG(Chr)3	11391242	TGGCCAGAGGTGTCACACGGGGTTGGTGGAGGCGTGTGGGTCTCACGCCCAAATAAGGAGGTGGCGCGTCCGTATGAGTTTGGCAGCGCAAAAAATGTCA	C	T	CCCTTCCTTTCCTTTTTTTTTTTTTTTCTTCTTCAAAATTCTGTGTTCCAGCTGTGCCACCCACCACCACAGACCACCGCGCAATCCTACGGCGAGCACC	NA	NA
Ca-II-SNP474	124.15	Ca-*desi*-LG(Chr)3	11437128	GTGTAGAGGACCCGGGCTTTCAAATATAACTGAAGAACAGATACTCTGGTTCATACTTTTTGAGCTAGATTTTATTTTGGATGCATTATTCCAATTAATG	T	A	CTTTTAAAGATGAAGCTGAGGAATATGCATATTTCATCAAGGCAAAGGAATGAAAACACTCATCATGGAGTTGACTATTCAGCAAAATGTTCAAGTCAGC	NA	NA
Ca-II-SNP475	127.95	Ca-*desi*-LG(Chr)3	11485180	GTCTCTTCGTTAATTTTAGGGAGCATCTCCAGCAGAAATGATGATGTATTATGTATTGGGCATACCTCTACTCGACGTGCATATCAACTATCTAAAACTA	T	C	TTATTTTTTTGGGTCTGATTGTAATATGCGGTCTTCTTAGCAGATATGGTTGTTTGGGGATTTTTTTGTATAAATATACTGTAGTTGCAATTTAATTTTA	NA	NA
Ca-II-SNP476	128.90	Ca-*desi*-LG(Chr)3	11517067	ACAGTTGTTCAAATCTGAGACTTTAATTTCTAAAATTATTGTGACTGATAGAGATCTTGCCATGATGAATGCGATTAGTGTTGTGTTTCCTACTTCAATA	G	C	TTTTTCCATAAATTCATTGCTTCTTCTTGTCTATCCTTTTTCACATATTGTTTGCATTTTGCCCCAACATTTTTTTCAATATGAAAACGACATAGCAAAT	NA	NA
Ca-II-SNP477	129.85	Ca-*desi*-LG(Chr)3	11540671	ATTAAGAATTTGAAATTGAATTGGAATTAGAAATGGAATCCTCCCTCTTTTTTTGTGGTTTTTATTTTTATTTATTTTTAATGGTAATTATGTATGTGAT	T	A	TTTTATCTTTTGCTACAGAAATCCTTAATACATAAGCACTTACATAATTAGTGCTTATTATAAGCCCTTAATCAAGTTGTTCAATTCAACAGTTACCACC	NA	NA
Ca-II-SNP478	130.80	Ca-*desi*-LG(Chr)3	11719694	AAGTTGGAAGTTGAGAAGATTTGACTTGTAGGTCAATGTGTTGTAGTACCTCAACGGTTTGTAGATTTCAGAATGGAAGTTGAGAAGACTGACTTGTAGG	A	G	TAGTTCACTACCAATGTGGCAGCTACGTCTAATCCTCTCACATAATCCCCTCACATAGAAGGATTTATCCACTAATTCAAACCTAAATTACAACACCTGA	NA	NA
Ca-II-SNP479	131.75	Ca-*desi*-LG(Chr)3	11802421	TTGGAATCACCAGTTAAGAATGCAGTCATTACTGTACCAGCTTATTTCAATTATTCTCAGAGAAAAGCCACCATAGATGCTGGTGCTATTGCTGGCCTTA	T	A	GTCAAAGACAAAAATATTTCGTTCTCCCATGCAGTTAGGTCTCTTGTCAAGTCCATAAGCAATAGCTGCAGCTGTAGGCTCACTCATTATCCGCATAACA	NA	NA

Marker IDs	Genetic positions (cM)	Chromosomes	Physical positions (bp)	Flanking sequences/Forward primers	ICC 12968	ICC4958	Flanking sequences/Reverse primers	Annealing temperature (0C)	Amplified Product size (bp)
Ca-II-SNP480	132.70	Ca-*desi*-LG(Chr)3	11810843	ATACATTTCTAAGGCATCATCCTTCATAATCACCTGAAATCCTTTCTGAATTAGTTGTCATATGCTCAACAAATTGTTTTTCATTCTTGGCACATACAAC	A	T	TGCACCATAACCTATTGGAAGCAGATAATCTTATGTTTTAGACCTAACTCAGCCTCACAATACCAACACCCTCTTGCGGTTAGGGTCTACTGGGTTTGGTGTGTGGACATTTCGTTGGCCAAATAACGATTCTAGTATATAGACTTTTTGTACC	NA	NA
Ca-II-SNP481	133.65	Ca-*desi*-LG(Chr)3	11828812	AAATTCTCTCCGGAATTGGGAGGGGCTGCAGCAAGCCCATCGGTGACGATGCAACGTACTTTTGTCGTTGGCACACATCACAACTTCTAACAAATTCTTG	C	T	AAGAATTCAATTCCTCTCAAAACGGTAGTCATTCCAGTTTCCTATGAACATATCGAAGAATAGCAAGTAATCTTTATTGGATAGGTATGACAAAAACGGC	NA	NA
Ca-II-SNP482	134.60	Ca-*desi*-LG(Chr)3	11858356	ACACTATTTCTCTGTAGTTAAATTTGATGTGAAAAGTTGATAGTTTTCTTAGCATAAATTATTATAGGGTAGATGCTACAGTTGCTTTATTGGTGGGGGC	A	G	AATATTTGTGTTCAATTTGTAATTGAAACAACCTTTAGGCCATTAGTTAACTTTTAAACACTAATCTATAATAAACCGCTTAGAAAGATTGTCCACGACA	NA	NA
Ca-II-SNP483	137.45	Ca-*desi*-LG(Chr)3	11925952	AACTTTTGATAAATATCAAAGTTTCGAAATGCTATTTTTCTGGATCCTCTGAAACTTTCGAAACTTTGCATGAATTTGAAAGTTCTGAAGTGTGATTTTC	T	A	ATTTTTTGAAATTGAATTACTTCGGAAATTTGAAATTTTCGAGCATTACGTTTCAGAAGTTTCAAATTCATCAAAATTTTCGAAAGTTTTGATAATTTCT	NA	NA
Ca-II-SNP484	138.40	Ca-*desi*-LG(Chr)3	11974306	CTTTGACGATCAGCAGTTGTTTTTGATTGACTATTGACAGTTCTAATCCCATTGCAGCATGGTTCTGGTACAGATGGACCTGGGCTCCTTAGATAACCAA	C	T	TACATGCATGGCTATGATATGTTTTGTTATGGGAAGTAGTATTATGAAGGCCAATGGTGAAATACCATGTGGTCAAGTGCAACTATATGTAGCATCATGC	NA	NA
Ca-II-SNP485	139.35	Ca-*desi*-LG(Chr)3	11993781	GTTAGTAGCTCCCACAAAACAATCCCAAAGCTATACACATCAACCTTGTTGTTATAGTGCTTTTTCTCTCCCTGGCGCAATGTTACGGTACTGTACAACT	G	A	TCTTTTTTGGAGCGTGATTTTATTGTTTTACCGACAACTATCCAAAAAAGTGCACCAGTGAGTCAGTGTGTTTTATCGACTGAATAGAGCTTTGTTTTTA	NA	NA
Ca-II-SNP486	140.30	Ca-*desi*-LG(Chr)3	12104449	TGGAGTTCTTAAGCCGTCATGACTTTTGTCCATCTTTGAACCCTTTTAATTTTGAGATCAAATAGCCTTTTTGAGCTTGGATGAATCCTACTTGTTCCTT	T	G	CAGCCTCAAAAAGTTCATCACAACATGTTGTTCAAAGTTTATCTACATCATTAACAAAGAATCTGTCCTGGAAATTTGTTCATATCTAATCAGTACATGG	NA	NA
Ca-II-SNP487	141.25	Ca-*desi*-LG(Chr)3	12119959	CGGAGGCTCCGACCCGAATCGGACGAGTTGAGTCCAGCTCCGAACACGTTCAGGTGCTTCTTCCTCAATCGCATTTCGCTCCCATTTTCTTCTTCTCGTT	A	T	ATAAAGAAAGAGAATGTTTTTGAGCTTCAGCAATGGAGATTGTGGCATGAATCGAAATAGAAATGGAATCGAACGCTTACAGATTCGGTGACGACGGCGA	NA	NA

Marker IDs	Genetic positions (cM)	Chromosomes	Physical positions (bp)	Flanking sequences/Forward primers	ICC 12968	ICC4958	Flanking sequences/Reverse primers	Annealing temperature (0C)	Amplified Product size (bp)
Ca-II-SNP488	142.20	Ca-*desi*-LG(Chr)3	12234970	AAACTTTATATTTTGTGGTACATGTATGTGTATCATTACACATTAATATTTTGATTTTTTTTTTTTTAAGTTTTCCGGTATATAAACTGAAGGTGATCAAA	C	T	TTTGATCCTTTAAAATATTTAAGTCACCTGCACTTGAATTAGTTTAAGAATTCAAAAAACAATAAAATTTAGACAAACACAATTCAACTTTTCTTCTCGT	NA	NA
Ca-II-SNP489	143.15	Ca-*desi*-LG(Chr)3	12256632	CGCTCACTCTAATGTGTCGAATGGGTGCCTTTTCGCGTTGCATGATTCTCTCTCAGCTATTGTTATGTTATCTCACATGCATAGTTTGTATTTATCTTTA	T	A	CTCATAAATATTAATTTTTAGCTGCACCATCTTAATATTTTTTTAATAATTTTATATTAATAACGTCTATATAATACTTGCATTAGAATTGAAATTGACA	NA	NA
Ca-II-SNP490	144.10	Ca-*desi*-LG(Chr)3	12310829	TGAACATAATTGGGTTTCAATTGATTAGTTCGGATTATACCTGAACTTGAATGAGTTGGGTCAAAATGCGAGTTAGATTGGATTGGTGACCGGGTTCGCG	A	G	CCAAAAAAATGTTTGTTCCTTAACTTCTATCAACACCTTGTGTCTCTTTCATCATATTCAATAAGCTATTAGTTAGGGGTGTTCATGTGTACGGTTAAAC	NA	NA
Ca-II-SNP491	145.05	Ca-*desi*-LG(Chr)3	12367109	CAGGGCGACACTTGATTTTTACGTCTTATGTTGTTCATTTATTCATAATGATAGATTAACTTTAATCTAATATAGACACATTCAATTACTTCAGCATCTT	C	G	ACTCTCAACATTTAAATTAATAGTATAAAATAATAAAATATCTATAAATTTGATAAAATTAAAGTGATATGAATATATATGTCTCTAGATTTTCAATGTT	NA	NA
Ca-II-SNP492	146.00	Ca-*desi*-LG(Chr)3	12551743	AGAACAAACTACTATGGTGTTAAGGAAACAACTGAGTCATTTCTTCCCCTTCTCCATTTATCCAATTCTCCCAAGATTGTTAATGTTTCCTCCCAAGCAG	G	A	CAAAGATTAAGTTGACAACGCTTTCAATTTCAGTAACTAAATTAGAGAGTAGTTTAAAAGTTTTGTTTTGGCTCGTACTCTTGTTTGTACCTTTAATAAC	NA	NA
Ca-II-SNP493	146.95	Ca-*desi*-LG(Chr)3	12599684	CGAATACAGACATGCTGTTTTTGCTGCTGACTTATGTGTAGTCGAATACAAAAGGCTGTATTCGAATACAGACATGTTGTTTTTGCTGCTGACTGCTGAA	G	A	ATATGTTAAACATGCATAATCAAATTTGAAAAATGAAGTTTTTCACAAAACTGCACTGTTGTATTCGAATACAACATCTCTGTATTCGAATACAAGGCTG	NA	NA
Ca-II-SNP494	147.90	Ca-*desi*-LG(Chr)3	12640565	GATGGCTCAGGCCGCCACAAAATCCCTCCTTTGTGAGGGCTTAGGCCGCCACAAATGACCTCCAGATTAAAAAAAAATCTTTTGTGAGGGCTTAGGCCGC	T	C	TACAATTTAAATTTATATTAATTTATTATATATAAAATTATAATACATAAGTAATTTTGAATTTAAAAAAATTAATTTTTTAATATGCGTAATATTTTGT	NA	NA
Ca-II-SSR129	148.85	Ca-*desi*-LG(Chr)3	12686141	CTTGATCGACGCACAAGCTA	(AT)9	(AT)8	AACTGAATTGAGAATTTAACATGGA	60.16	189.00

Marker IDs	Genetic positions (cM)	Chromosomes	Physical positions (bp)	Flanking sequences/Forward primers	ICC 12968	ICC4958	Flanking sequences/Reverse primers	Annealing temperature (0C)	Amplified Product size (bp)
Ca-II-SNP495	149.80	Ca-*desi*-LG(Chr)3	12697957	GATTAGAAAATCTGCAATTTAGGCCCAGTAAAGTTTGGCATTAGACTTTTATTCCAACACAAAAGTTGTAGCTCTGATTTTTATCTTTCCAACGCTTGCT	T	C	ATTGCTCTTTGATAGTTTGCCCCAGCATTTTTCAATCCAAGTGACTCTTGCTCGCTCTGTAGGCCATAACTTGAGCTCTAGATATCCGATTGGTGCGTAC	NA	NA
Ca-II-SNP496	150.75	Ca-*desi*-LG(Chr)3	12752594	TCTGAGACTTTAATTTCTAAAGTTATTGTGACTGATAGAGATCTTGTCATGATGAATGCGATTAGTGTTGTGTTTCCTACTTCAATACATTTGCTATGTC	A	G	GAATATACAATTTTTTTTCCATAAATCCATTACTTCTTCTTGTCTAACTTTTTTCACATATTGTTTGCATTTTGCCCCAACATTTTTTTCAATATGAAAA	NA	NA
Ca-II-SNP497	151.70	Ca-*desi*-LG(Chr)3	12781161	TATTTTATTGTAATTTTTTTTTAAGTCAACTTATTTTTTTAGTGTCAACTTATTTTTTTAAAGTAACTTATTTATTAAAATCGTTGTTTCCGTGCGACGC	C	A	TTAACTCATATATTATATAAGAAACATTACGGGTTTGTTGTTTGGGAGTTCAGGAGGAGTCTCGATAGAATTGCTTTAAATGTAACTAGTCTGTAACCCG	NA	NA
Ca-II-SSR130	152.65	Ca-*desi*-LG(Chr)3	12784679	TTGACCATATAACCATATAGGAATGA	(AT)8	(AT)7	TCAAAAGAGATAGACGTCAACACA	57.92	103.00
Ca-II-SNP498	154.57	Ca-*desi*-LG(Chr)3	12991606	TTTGTAAATTTTTGTTCTTTGAGTTGATAAAATGTTTTGTGTTTTTATTTCGTTGATTAAGTAGGAATTTGTCGTAATTTTACATTGCGTTTTGTTTTAG	C	T	TTTTCACTTTGTTTGACCCCAATTGAACATCTACAACTCCAGATATGGCTGAAATACTCTACATATGTCATGTTGATTTCTCACCAAAATTCAGCACTGC	NA	NA
Ca-II-SNP499	155.53	Ca-*desi*-LG(Chr)3	13021945	TATTGTTGTTGGAGCGAGTAATTGATGATGAACAACCATCTTGTTTAACTATAAATACTAGAACTATTTGGGTGATGGTTTACAATCTCCCTTTGAAGCT	T	C	TCATACACAAAAAACAATTAGTTATGTGTGATTCCTTTGGAACTGTTTCCTCATGTGTGCCGATGATTCTTCTTAGCTTCTTTGACATGGCCTCTGATCA	NA	NA
Ca-II-SNP500	156.49	Ca-*desi*-LG(Chr)3	13047533	CTTGGAACCATTTTGATGCAAAGTATCCAAACTTCGCAAATGAACCAAGAAATGTAAGGCTTGGTTTATGTTCTGATGGCTTCACGCCATACATTCAGGC	C	A	TGAGACAGGTCAAAAACATGAATGGTTTAGTCATGCACATTTCGGGCGGGAGATTATACGGGGTCACAACAACAGGCCAACAAGAATATGGGGTTGAAGA	NA	NA
Ca-II-SNP501	157.45	Ca-*desi*-LG(Chr)3	13165372	AATGTTTTTATTGTTTTATGTATCAATCCATGGTTTTATTGTAAAAATTTAATTAAATTCTTATTGGTTTGAAATTGACTTTATATTGGCTTGAGAATTG	T	G	TTTTTGAATTAATCAAAATGATATTTATAATTATTATTAAAGAGATTTGAAATCAGTACTTCGAAATTACAAAATTAAACTCTTAACAAATGATCTGTAC	NA	NA

Marker IDs	Genetic positions (cM)	Chromosomes	Physical positions (bp)	Flanking sequences/Forward primers	ICC 12968	ICC4958	Flanking sequences/Reverse primers	Annealing temperature (0C)	Amplified Product size (bp)
Ca-II-SNP502	158.41	Ca-*desi*-LG(Chr)3	13193734	TTTTTGTCATCAATAGATTAATTACCCTGTTTTGAAGTTCAAAACATCTTTTGAATTGTTTTATAACATGCCACTCACTTTTGTTGATTTAAAGGTTTTT	T	G	CTTTAGTACCTAACTTAAAACCAAGAAAAAAACATTTTTCGAGCCTGATGATCTAGTTTGGATCTTTGACGAAGTAAGGTAGAGGCAAAAGATAAACAAC	NA	NA
Ca-II-SSR131	159.37	Ca-*desi*-LG(Chr)3	13219360	ACACCGACTCATTGACACCA	(AT)9	(AT)8	TGGTTGGGTTGCCATTAGAT	60.01	149.00
Ca-II-SNP503	160.33	Ca-*desi*-LG(Chr)3	13220353	AAATATTAATTTTTGTCCTTTTCTTTGTCGGTAAATTGGATGGTACGTACACTTGCATGTGTGCTGTATGTTTAACGGCAAATTATTTGACTCAAAATAA	C	G	AAAAAATGAATTTGACTTACTTTCATTCATTTCAATCTCTCAAAGTTCTTTTGTTTGGTTTTTGTGTTATAACATTGTTTCCTGAGCTTGTAGTGAGCATAACAAGGCACTATACATTTAATATTGAGTACTTGAATGTCACAAGATTGTGCCACACAAGAAACATTCTTAGTGTAAATGGGAAATTCCCAGGACCTCGTTTAGTGGCAAGGGAAGGTGATAGAGTTTTGGT[illegible]	NA	NA
Ca-II-SNP504	161.29	Ca-*desi*-LG(Chr)3	13265079	GATTGAAATAGCTAATTGATTGCTTGAATTGTTGGACTGTCCCATTTCAATTTCCCCTTAAATCCTACACATTGCTTCATTGGGAGCTTTTGTTGAATTG	A	G	AATTTAATTGTTAAAATTAAGTTAAAAGAAAATGAAAAACATAAGCAGCAATGAGAATTATGATTCAACAAAAGCTTGAGCTCTAACTAAAAAAACAGCC	NA	NA
Ca-II-SNP505	163.21	Ca-*desi*-LG(Chr)3	13318452	TTAGTATGAAGTTCAGAGTCAGATGCTTGCTTTGAGTTGCTTTATTGGAGGCAATTCATTAGAACATGTTACACTTGACTTCATAGTCTCATCTTTAGAG	G	A	AACACGCACCCTCTAAAGCAGATGCCTCTAAGGATATGCCCCTACCTCTAAGTAACCGAGTCTAAGAATTTGAATCTGATGATCTCCCTCCAAGGTTTTT	NA	NA
Ca-II-SSR132	164.17	Ca-*desi*-LG(Chr)3	13319724	AATCAATTAACAAGGCTCGCA	(TA)16	(TA)10	GGAAAAGATTGGCCAAGGTAA	59.73	108.00
Ca-II-SNP506	165.13	Ca-*desi*-LG(Chr)3	13345747	ATACAAAATGTGAAGTTTTAATGACATACCTATTAGTCGTAGTATTTCCTAGATGCAATACTCGATTGGTCCATGCTTCAACAAATCTTTCCTTATGCGG	C	A	GTTCTGAAGAGAAGGAGTACGAGGAACTTTTGCAAATTTTTGAGCAAGCATGTGTCGATAATATTATTTTCTTTGACTACGTCAGAGACACATGGTTGAA	NA	NA
Ca-II-SSR133	166.09	Ca-*desi*-LG(Chr)3	13372497	TGGATCCACAACACGACAAT	(TA)19	(TA)15	TCCCTTTAAGCGCAGAAAAC	59.81	179.00

Marker IDs	Genetic positions (cM)	Chromosomes	Physical positions (bp)	Flanking sequences/Forward primers	ICC 12968	ICC4958	Flanking sequences/Reverse primers	Annealing temperature (0C)	Amplified Product size (bp)
Ca-II-SNP507	167.05	Ca-*desi*-LG(Chr)3	13385275	TGTAGATATGGATCTTAGCTTTCATTAGCACTTGG AGTGACACTAATTGGACATCTAGAACTCCAGATAT GGCTGAAATACTCCACATGTATCATGTTGA	C	T	TAATAAAATGATTTTTGTTTTTATTTCGTTGATT AAGTAGGAATTTGTCGTAATTTTACATTGCGTTT TGTTTTAGTGCAGTGCTGAATTTTGGTGAAAA	NA	NA
Ca-II-SNP508	169.93	Ca-*desi*-LG(Chr)3	13478676	TTTATCTAATGCTAACAGACTTAATTGGTGTAAGA GGTATGCCATTGCTTTAGGAACAGCTAGAGTGTTA GCTTATTTACATGAAGAATGTTTGGAATGG	A	G	TTGTTTCTATTTATTAACTTAGAAAGACCAAAAT CAGCTAACTTGGGATGCAAGTTAGAATCAAGAA GTATATTTTGTGGCTTAATATCACAATGCAAAA	NA	NA
Ca-II-SNP509	171.85	Ca-*desi*-LG(Chr)3	13503033	GATTATCTATTGGTTCGGAATCTTGGAAGGGTCG GAGAATGGGTCCTTCATTTCTCTGTTTGTGGTTTC TTATTTCAAAGACATTTCCCTTCCAAATAGG	C	G	ATGATCAAATTCTTGATCCCAATAATAATGAAG GGCCACTTGAAATGCCACAACATAATGACTCAT ATATAGAGACACAAAGCAGGTTTATTACAATTG A	NA	NA
Ca-II-SNP510	172.81	Ca-*desi*-LG(Chr)3	13520714	TTGGACAATTATTTTTGTTGTTATATCCCACGGTC CAACCCAACGTATATCATTTTGATTGACCATATAC AAATACGGCTAAGTTTTGGATGATTCTTGA	G	A	TCAAATATAAGCCAAAACAATTATTAATCAAACT TTACTTATCTTTAAATTAAACTTTAAATTATTAAA TTTTCATTCTCCTAAGACAGTGAACAAAGTT	NA	NA
Ca-II-SNP511	173.77	Ca-*desi*-LG(Chr)3	13570118	CATCTATTTTTAACTTTCTTGCTCTTGGACCTTTAT AGAGTACAAGTGTCACCATTCAATTGGAGAACAG GAGCAATGCTCTTCCCGCTGGACTTGTGGA	A	G	TTCCATGGATAGGCTCCCTGTTGGACTTATTCT CTCCCTCCATGTCCAGAATGTAAAAATCTACAG GAAATATCAATTCATTAACTCGAACAAGTACATC	NA	NA
Ca-II-SNP512	174.73	Ca-*desi*-LG(Chr)3	13635021	TTCTTTCCTTACAATTGGCAAGAAAATGTTGAAATA CGTCTTCATGCTTATTAGAGTTACATGTCGAATTT TCAATTGATGTTGAACTATGATTACTTTT	C	T	AATAAATCAATGTTTTAAAGAATCAAAGTGTTGA AGTAAAAATAGAAAATGCAACTCTGAGAACTGA TATTGTTGAACTTAAGGTTGAAAATCTAACTCC	NA	NA
Ca-II-SNP513	175.69	Ca-*desi*-LG(Chr)3	13739225	CGTGTAACACTTTTTCTCTCAACAACTCAACATCG TTCAACCTTTGAGTATTTTTGCATCTTGAGAGACT TCCACCGTATTTTTGGTATGTATTTTGTAT	C	A	ATAACAACCCAGTTTGATTATTAGAACCAACTT CTTTTTTAATGGTATGAGTTTTATCACCCTTTTA ATGTAGGAAAAATAAATCTAATAGCAGAGCAAA	NA	NA
Ca-II-SNP514	176.65	Ca-*desi*-LG(Chr)3	13794278	AAACTGGGTTTCCACTGTGACTTATCCAATCGATT TCCCAATCGATTTATTTCAATCAGGTTTACTTTGTG AATTGTATAATCGATTTGTCAATCGATTA	A	C	AAGGAAACACAACTGTTTGTGTTGGTATAATCG ATTGGGCAATCGACTGACTGAATTAGAAATTGA AAATGCATAAGTCAGTGGCACCCCAGTTTTGAG G	NA	NA

Marker IDs	Genetic positions (cM)	Chromosomes	Physical positions (bp)	Flanking sequences/Forward primers	ICC 12968	ICC4958	Flanking sequences/Reverse primers	Annealing temperature (0C)	Amplified Product size (bp)
Ca-II-SNP515	177.61	Ca-*desi*-LG(Chr)3	13874492	CTTTTCCTCTCAATGTGCGATTACGATTTCTTGAAGCTTCAGGAAGAAAACGGGATTCAGGAAGATTCAGATCATTAATTTACGATTTCACGGAGATTCA	C	G	TTCCTGTTTTCTTATCTCATCTCCACTAATTTCCCTCTTCTCATCTCCATCGTTTTCCTCTGCGTCTTCCGTGAAATGCTATCGTCTTCGCGTTTTCTTC	NA	NA
Ca-II-SNP516	178.57	Ca-*desi*-LG(Chr)3	13914902	CAGATTCACGCCCCGAGGATGAAGAAACGGCGAGCGGCGACAATGGCGGGCAGGGACCACGGCGAGCAGTGACGACAGCGAGCAGTGACGACGGCAAACG	A	G	ATTAAAAACTTGAACCTGCGCTTGAAGTTATCCTGGTCGTCGTCGTTGTCATTGCTCGTCCTGGTCGCTTGCTCTCCGGGGTCGCTGCTCGCCGTGGTCG	NA	NA
Ca-II-SNP517	179.53	Ca-*desi*-LG(Chr)3	13936315	TACATCTAGTCTTTTAAATTTATCTCAATTTCCGTTTAGGTCATTTAAGTTTACTTTTTCTTAATTTGGTCTTTAACGTTACATTTTGTTTGTATTATTA	A	G	GTCACTACGATGTTTAGTTTGAATTTTAACATTTAATGACACTAATCCGTTAAAATTGTCAAATATTAAAGACTATATATAATTTAATATATCTTAAAAG	NA	NA
Ca-II-SNP518	180.49	Ca-*desi*-LG(Chr)3	13949576	CCTTTAGTGATTTTGACTATTTAATGTGATATTTTCTTGATGGAATGTTTGTTTAATGATACGGTTCTATTATGATTGTTTTGTAAGACATGTGCTTCCC	C	T	TCAGAACTCTCTTGCCATTGCTATGTGAAGAATGAAACGTGTGTTGAGGAAAGCATAAAAAAGACCTAGCTGGGAGAAACCTGTTATGATGCTAAATGTGCAAGTGCATGGGAAATTGAAAATGTGATTCTTTTTAGGTTCCGCATGTTATGCATCACAGTTCTTCACTCACAAACATGTCTTAATATCACAAAAGAAGAAAAGATATCTACTAAGC	NA	NA
Ca-II-SNP519	181.45	Ca-*desi*-LG(Chr)3	14004716	GGTGATATTTTTCCTATTGCAAAAAATGCGACTTGAAGTGATAGTAGTTCTTTTAATGTTTCAATGTCTGCCCTTTGGCATTATAGGTTTGGACATGCCT	A	C	GCCCTGTTGCCACTCAATAAAAAATGTCAATCTACAAATCAAATATTTATTAACAACAATAATAGGAAAGTATTTGTGAAGCATATCTAACTTTGTAGAA	NA	NA
Ca-II-SNP520	182.41	Ca-*desi*-LG(Chr)3	14069380	AATTCCTTTATTGAATTATTACCAAATTCTCTTTATTATTCTTGCCCTAATGTCTTAGTGACATAACCTTTATTTCTAAAGTAACCCATAATTCCTTAGT	A	G	AGGGATAGATGCGGGTCATGAAACCAATTAAAGTTGTTGCAAAGGCAATAGTTTTAAAAGAGAAATCTTGAACTGTAAAACACAATTCTAACCTTAAATT	NA	NA
Ca-II-SSR134	183.37	Ca-*desi*-LG(Chr)3	14096792	TCTGTTTTGGTTGGTGGTGA	(AT)8	(AT)7	CAGGCCGACCTACTTCCTTT	59.98	128.00
Ca-II-SNP521	184.33	Ca-*desi*-LG(Chr)3	14141483	TGTGGTAAGAAAAAAGTTGTTAAACCACGTGTTGTTAATGACCCATCTCTCATGCCGATGCCGACGATTGGTACTAGTGGTGGAGCTATTCCTCTATATC	A	C	ATACCCATCTCCTTCTGGAATGATGACAATGCGGTTGTAGCAATCAACACATCCGGGTTCCATTACTTCGTCTATGGTATAAGGCTCTATAGGGACCTCA	NA	NA

Marker IDs	Genetic positions (cM)	Chromosomes	Physical positions (bp)	Flanking sequences/Forward primers	ICC 12968	ICC4958	Flanking sequences/Reverse primers	Annealing temperature (0C)	Amplified Product size (bp)
Ca-II-SNP522	187.21	Ca-*desi*-LG(Chr)3	14208354	TTTAAAGGCATATATTTCAAACCTTTATTGGAATCCATGTGATGTTATTAGACTTTTGTTTCGACATTGTAGCACCTCTTTGAGCTCGAAAAACTTGTAA	A	G	ATGTTCGACGCGTAAGTAATATTCATTTATTTCTTACATATATATATAAATAATGTGTTTGTTAATGCTATAATAATCACATTTATTTATTCGATAGTGC	NA	NA
Ca-II-SNP523	188.17	Ca-*desi*-LG(Chr)3	14405537	AGTTTGGAAGGAGCCCTTACCTTAACGTTAGCTCTAACGTGCGATTACGGCACCGTGAGTAAATCCGGTAAATTTTCCGCTCGCAACGCCGCTTCCAAAA	T	C	TCAAACACAAACCTCCCTGTTACACCTAGATCTAAACGTCGTTTCAAGAAGTGATAGAAGGGAAGGTTGCTCACCTCCGTGCTACGGTGCTAGTGGATCA	NA	NA
Ca-II-SNP524	189.18	Ca-*desi*-LG(Chr)3	14513401	ATTTGCGTCACATCGTTTATATTCTTATGTGGCACCCTTCATATGTTTCTATTATTCATCTCACATTTTCTATTCCTCAATTTAGTCTCTGCATCTCACA	C	T	AAGAATTGGATGAGCCGCAAAAGTTGATTGAAGTGAGAAGATGATAGAATTATTTTTATGATGAGCTTTTTTGAAAGGGTTTTTAACTTATGTATATGAT	NA	NA
Ca-II-SNP525	190.19	Ca-*desi*-LG(Chr)3	14615636	AAGAGTGGCTTGTACGAAACTTATTTCTCGACTTTTTTTTCCCTTCGATGAATTTCTTTTATAATCCGTTTTCGTTAACATTCGAAGTTTTTATTTGTTT	T	C	AAACCAATAATCTTTGGTCATCTTCAATACAGAAACCGATCAATTTAACGAGATTCGAGTGGACAATATCACCAAGAATGTCTAACTCAGCCTGGAAAGG	NA	NA
Ca-II-SNP526	191.20	Ca-*desi*-LG(Chr)3	14739635	ACCATTGCCACGAGTTTCTTGTTTCTTGTTCATCTTTACTTATTGTAGCTATTAACTGGCCACCATAGTCTCCCTTCAAGAAACATGCATCCAACCCAAT	T	C	ATATTAAGTGTGGTATGTCTTGTGTTGGACTAGTTTTTGAGAGAATATATGTATGTTTGGAGACTTGTAAGGTTGCATTTGCTAATACATGTAGACCTTT	NA	NA
Ca-II-SNP527	192.21	Ca-*desi*-LG(Chr)3	14776422	CCTTTAGTGATTTTGACTATTTAATGTGATATTTTCTTGATGGAATGTTTGTTTAATGATACGGTTCTATTATGATTGTTTTGTAAGACATGTGCTTCCC	G	T	GTTTGATTATTTTCCTGAAATGTTTCTCAATTCAAACCATTAGATCAATCAAATAATTCATATGTATTGTATGTTTGGGAATTAAATCAAAAACATGATC	NA	NA
Ca-II-SSR135	193.22	Ca-*desi*-LG(Chr)3	14825740	GCTCAACATTGGTTGCCTTT	(CT)8	(CT)9	CCATTGGAGACCAAATGACA	60.12	183.00
Ca-II-SNP528	196.25	Ca-*desi*-LG(Chr)3	14949792	ATACATTTCTAAGGCATCATCCTTCATAATCACCTGAAATCCTTTCTGAATTAGTTGTCATATGCTCAACAAATTGTTTTTCATTCTTGGCACATACAAC	A	T	AAAAATGGGTGAAGAAGTTCATATTCAAAATCAACAACAACAAGAACAAGAATCCTTTTTCATCCCAAATTCTTCAATCTCTCACAAACAAAAACAATCCATTATTATTCCAGCTTCATCAATGCTTGATCTTGATCATCATTGTAATCATAATCATCATCATCATCAACAAGAACAATCAATTAACAACAATAACAACGATTTTTCATCAATGCTGCATCTCAACAGCACAAC[illegible]	NA	NA

Marker IDs	Genetic positions (cM)	Chromosomes	Physical positions (bp)	Flanking sequences/Forward primers	ICC 12968	ICC4958	Flanking sequences/Reverse primers	Annealing temperature (0C)	Amplified Product size (bp)
Ca-II-SNP529	197.26	Ca-*desi*-LG(Chr)3	15061743	TAGAGTTCCAAATAAAATTAGGGTTTCAATGAATACCGGCGGTGACGAATCGGCGATTGTACTGCATACGCTTGTGAGCACGGCCACGTGGCTTCTTCTT	C	T	TAATCAACAATGGAATCTATTTTGTAGGTAAGGTTCACGGTTCACTTGCTCGTGCCGGAAAAGTGAGAGGCCAAACACCAAAAGTGGCCAAGCAGGACAA	NA	NA
Ca-II-SNP530	198.27	Ca-*desi*-LG(Chr)3	15111898	TTGGAACTATTTCTCCATCCTCGGAGTCCTGATCCTTGAGGGATCAAAGAATTGGCAATTTTAGTCGAATCATTTTTATCATTAGCCTTGCTGCTCCCAT	G	C	GAGGACTGGTCTTCAAAAAGAAGGATCAAGAGTGATTTTTGGAATTCCTAAACCTGGGAAGAAAAGGAAGTTTATGGAAGTAAGCAAGCATTATGATGCA	NA	NA
Ca-II-SNP531	199.28	Ca-*desi*-LG(Chr)3	15124677	CTATTTCAGGTTTCTTGCTGGAAGAAAATTCTACTTCGTACTCTTCGTTGTCTTGTATTACTCCTTCACACCACTCCTCAATCAGCTCCTCCAGGTAGTA	T	C	AGAGAGGGAAAGGAATTGATTATATCTACTTGTAACTTCTCTCTTGATATCATGTGTTTCACTAAAGCAACCTAGCCTACTTACAAAATGTACTTTATTGTGGGATCGGAGAGGCAATTCCTGAAACAACCTTCATCAATAATGCAAACTTTTAGAAGCATCTTAAGTGAAATCACAACGGAAATAGTAGAAAATAGTTATACATGAAAAAGAAAAACAAATGGGTTTGGAA[illegible]	NA	NA
Ca-II-SSR136	200.29	Ca-*desi*-LG(Chr)3	15207234	GGATTGAAAAACCCTGGAAA	(AG)8	(AG)9	GCTACTCCTCCTCAACCCCT	58.86	102.00
Ca-II-SNP532	202.31	Ca-*desi*-LG(Chr)3	15327978	TCTTCAGAAGATACATGTATTCACCTTTGAGATGTTGTGCAACTCCCGCTAATCTTTCATTTTTCATGCGTGGGACTAATTTACAAGAATTTGACTTTCA	A	T	TGGAAAAAAAGGAATTATTAAATTATAGTGAATGATTTAGTTTTTACAATGTGATCAATTAATCATAAGCTATTTTTTCATCATCTTTAAATTAGATATA	NA	NA
Ca-II-SNP533	204.33	Ca-*desi*-LG(Chr)3	15465535	TTTGTTTGTACTTTCTTACAGGTAAATCAACAAGAAAAAAATGTTCAGGTTATGACATTTGGACAACCTCGTATTGGGAATGCTGTTTTTGCATCTCTCT	A	G	TTGGTATGTCTTTTGCGGTAAATAGTAATAGTATGGAGGCAAATGAGGAACAATATCATGCTCATGTGTGACTCGGATTGTATTTGGAACTAGTTTGCTG	NA	NA
Ca-II-SNP534	205.34	Ca-*desi*-LG(Chr)3	15837875	AACTTGTCGCTGGATCATGGACTTATTCAAGGACCACAACTTTGTGGCCCAACATTGTATACGGATACAAAGATATTTGTACCATGTACCCCCACCTATT	C	T	TTCGAATGGGGGCGTGTGGAATGGAGTGGTAAATTTTTTTTTTTTCATAAAAGGTAAAAAAGGACAATATAGTCTTTTCCATTGAAATGGGGGGTGGGAG	NA	NA
Ca-II-SSR137	206.35	Ca-*desi*-LG(Chr)3	15914984	TGCACACAACAAGTGTGCTT	(AT)12	(AT)13	TCTACAAAATGTTTAAGATACGCACT	58.92	179.00

Marker IDs	Genetic positions (cM)	Chromosomes	Physical positions (bp)	Flanking sequences/Forward primers	ICC 12968	ICC4958	Flanking sequences/Reverse primers	Annealing temperature (0C)	Amplified Product size (bp)
Ca-II-SNP535	207.36	Ca-*desi*-LG(Chr)3	15992284	TGTTCCAAAATATTTTTGGGAAGAGGTTGTCCTCACATCAACATATCTTATTAATCAATAACCTTCTAGAGTCTTGGGGTCCAAGAGTCCTATGGATGTT	G	C	TTTCCCCAATCATGACCATGAACATGGACAAATGAGACACACCCAAAGATTTGTGGTGGAAGATTAATGGTAGTGGACAAGTTCAGATAGAATGAAGAAA	NA	NA
Ca-II-SNP536	209.38	Ca-*desi*-LG(Chr)3	16125396	CGTATAGGCAAATATGTGTGGCTTTTGTCTCCTGTTAAGTGTTAACTGCCATTGCTTTTTTTATTTGGATGACTATTGCTGCTTTATTCTGATTCAATCC	G	A	AACAACCCAGGGTTCACATTCACCATTTACACTCTTTGATGTCAATGCATTCTCTTCAAGAATTTCACAGACCTATAACATCAACAACAAAAGTTCAACA	NA	NA
Ca-II-SNP537	210.39	Ca-*desi*-LG(Chr)3	16211241	TTCCCGCGTTACCCTTCCCTCATACTTATAACAGAAAATCCCTTAAAATCATGGAAGACTCTAACCGACTATTATTCCCTCGCTTTCTAGAAACTGTTAG	A	G	TGTATCATTTTCTTTGCAGAGATTCTCTCCCATTATTTCTTATATTTTTTTCAAATATTAGTGTCTTTCCTCTAAATCCTTGGAATCAAATTCTTGGGTT	NA	NA
Ca-II-SNP538	211.40	Ca-*desi*-LG(Chr)3	16234484	AATGCATCCAAAACAGGAGAGACAGTGGTTGTTTCGGAAAACTTTTCTAGCAGCGACGGCAGGCAGCAGCAGCAAACGGCGGCGGCGGCAGTAGCAGACG	T	G	ATTAACTCTAGCTTCTTTGTTGTATCTTTTGGAGCCACTTTACAAAGAGATTGGACAAAGTGCAATTTGGGCTGTTATGACTGTTGTGGTTGTGCTTGAGTTCACTGCTGGGGCAACTTTATGCAAAGGGCTCAACAGAGGATTAGGGACACTGATAGCTGGATTACTAGCATTCTTTGTTGGCTATATAGCAAATGCGTCATCTGATAGAGTCTCTCAAGCAGTTATC[illegible]	NA	NA
Ca-II-SNP539	212.41	Ca-*desi*-LG(Chr)3	16346213	CTGAAAAAACTCTCACAAAAGACTTCAAATTTAGTTGGATTTTGTGGTTGCCTTAGCCCTCACAAAATCACAACGTTGTTATTTTGTGAGGGCTTAGGCC	A	G	CGGATCAATGCTTTTGTGGCGGCCTTTACCGTCACAAATGGTTGATTTTGTGGCTGAAAAAGCCCTCACAAAGGACTTAAATTTTAGCTGGATTTTGTGG	NA	NA
Ca-II-SNP540	213.42	Ca-*desi*-LG(Chr)3	16443044	AAATCTCATTTGTGTCCCTTAGGTTTGTTTGGATTGAGACTTAATTAAGCCTTAGGCTAGGTTTGGATTGATGGTATGAGATGGAATAAGTTTATGTTCC	A	T	AATTGGAGGGGAACAAAAGTGAGGGTAAGTGATGGAATAGGATGGAAAGTATTCCATTATATTCCACTTCATTTCATTCTCTTTTTGACAATCCAAACAA	NA	NA
Ca-II-SNP541	214.43	Ca-*desi*-LG(Chr)3	16522022	TGATTTTACTTAGATGATGTCTTTCCAAAAATAATTGTTATGATTGATGAGTAAGAATGTGTAACACCCCGGCCATTACCGTTACCGACGGTGCCACCTC	A	G	GGAGGCGGTATTATCCCCTAGGTACCGGGCTCGAATCCCACGGAAGGCAAAAACTCGTCAGATTTCCATTGGGGGCGGAAAGAGGGGGACGAGAAGGTCG	NA	NA
Ca-II-SNP542	215.44	Ca-*desi*-LG(Chr)3	16545023	TTACTCTTGTCCTTGTTTTGTGCCAAATACAAGAACCCAAATGCACCAACTATAGCCCCGAGTTTCCCTGCTGCTGATGATATTCCATGACAAGTAGATC	T	G	GCGGCAAAGAGTGGAAAGGGAGGGAAGAGGAGGAGGTGTGTGAAGAAGAAAGAAGGAAAGGTGAATTTGGATGCAACGAAGCCTCTAGAAGATCTTTCTTTCTTTCTATCTCTCTCTACAGAGGAATCGTAGATCTGATTTCTTTCTTTCTTTCTTTTCGATCAATTCTTTTGTTAATCAGAATTTACTTCAAGAAAAACAATGGGATTGTGGGAAGCTTTTCTGAATTGG[illegible]	NA	NA

Marker IDs	Genetic positions (cM)	Chromosomes	Physical positions (bp)	Flanking sequences/Forward primers	ICC 12968	ICC4958	Flanking sequences/Reverse primers	Annealing temperature (0C)	Amplified Product size (bp)
Ca-II-SNP543	216.45	Ca-*desi*-LG(Chr)3	16680965	ACTTTGAAAAGGTTAAAAATGTTAACTCTTCCCATATGTATGTATCCTCAATTCTTTCATGGTTTTTTGTGGCTCCAAAATCACACTAGTTGGTTCTTAA	T	G	CAACCACCAAATATCAAATATTATTGGTCAGAAATATTATGAATGTACAGGACATAAAACCAAATATGGCTATGGCCAGTCTCTGACATTTGATTCAGGG	NA	NA
Ca-II-SNP544	217.46	Ca-*desi*-LG(Chr)3	16719937	ACTCTGCTGTATAAGACCACCAACTGCGTGACACCGTGTACACCTAGTATCGGGACCAGCATCATGACTAGGACCAGCATCAGGACCAGGACCAGGACCA	G	A	GAGTGTGTTGAGGGATACATTCAGTGGTATTATAAGATATCTCATCCTCGGATGATCCCTTATGTTGCTGCATATGCTTGTCCTTGTTATGATGCTGATG	NA	NA
Ca-II-SNP545	218.47	Ca-*desi*-LG(Chr)3	16978563	GTAATTTTATCGTTACTCTAAATTATTTTGCTACCTTTGTTGAGAAATTTCTGATACTAACATTAATTACTTGGAATTGTTGTTATCTTCTAAATTGATG	T	A	ACCGATACTAAAACAAGAGACTTCTGAATTTGGATTCCAATTCGAATTCTAAGTGCATATCATCGTATTAGAATTACAAAATTCCAATTTTAAAAGCTAA	NA	NA
Ca-II-SNP546	219.48	Ca-*desi*-LG(Chr)3	17008520	AATTGGTCATTCCATTTCTCATGTAATAGAAGTTTTATTTTTGTCTTAAAAAAAATCTTTTATTTTTAATTTATTTTTCTTATTTTTTAATTGGCCATTA	G	A	ACGATCTTTGTCCACGATACAATTTAATGGTCAATTAAAAAATTAAAAATAAAATACAATACACCACGACTTACAGTTTTTTCATGTATCCGTTGAGAAG	NA	NA
Ca-II-SNP547	220.49	Ca-*desi*-LG(Chr)3	17116615	CACATTTAAAACTTTTTTCTATTAATGGTAGGGGCGTACAAGACCCGGTTCGTCCCGCGAATCCGACCTGACCCATAAGCAACGGGTTGTGTCTGACCCG	T	C	TTTTTTTTTATGCAGACACTACCTCTCATTTCTGAAAATAGACAATTTCAAAAATCTGCATTACAAATATGAAAGTTAATCATTCATCATACTTAAAAATAAAGTTAAACTCTGAAAGAAATAGTTTCAAAAAGTTCAGGTCGCCAATCCCGCTGCCACCAAAAGCTGCTTAACGATTCGCCTTAGCAACTCTCTCAATTTCATCTTTTTTCTTGATTGCATAGCTGTTGGATG[illegible]	NA	NA
Ca-II-SSR138	221.50	Ca-*desi*-LG(Chr)3	17212326	TTGGCATCATCAAACCATGT	(AT)9	(AT)11	GTGCCAATTCCAGTCCTTGT	59.78	114.00
Ca-II-SNP548	222.51	Ca-*desi*-LG(Chr)3	17288468	TAAAGCCTACTATCCCAAAATGTTCTACCTTTACCTAATCCCCATTACAACCCGAAAGTCCTCCAAAAGTGTATGTGTTTGTTGCATTGTGATTGCTAAC	T	A	ATCTCACCAAAATTTCTCTCAATTGTTTAGCAAAGGTTATGAATTTATCACTCAAATCCTAAAACATGCATCACACTACCATTGCCATAAAAAAAATTCT	NA	NA
Ca-II-SNP549	223.52	Ca-*desi*-LG(Chr)3	17313806	AATCTCACGGTTGTGAGGATTAGATGTAACCCGGGTTAGATGAACCAGAATATATCTCTGTGTGATATCTTTTCCCTTATCTCTATTTATTTCTAGTCTT	A	T	TTTAACTCAAGATTAATCTTGATTATGAAAATAGAAAACGGAGAACGTTATTGGTTAGTGAAAACACACAAATTAACTTATCTATTTAATTTGATAATCA	NA	NA

Marker IDs	Genetic positions (cM)	Chromosomes	Physical positions (bp)	Flanking sequences/Forward primers	ICC 12968	ICC4958	Flanking sequences/Reverse primers	Annealing temperature (0C)	Amplified Product size (bp)
Ca-II-SNP550	224.53	Ca-*desi*-LG(Chr)3	17346792	GGATTAATCTAATAGCTCCAAAGTGTAAGGTTGTGTATCCTTCATTTTTCCAATGATTTATAAGATTCTGTGAAATCTCTAAATCAACATACTGCTCTTG	G	A	TCACCAGATCTATCAGAAACCTAATCACAACTAGCCGTCCTCACATGAAAGAATATGTACAATCTTCACGACTTGATCAGTGCAGCCTCAGAGCGACAAA	NA	NA
Ca-II-SNP551	226.55	Ca-*desi*-LG(Chr)3	17415276	TTATAGGGTTGTTATTGTTGATTGATATTCCTTATTGTTATTATGAAGACTCTCGGTGTACCCATTAATTATTATAGTGGAAGTTTTACTGGACTAGGTC	C	T	ACAATTCCAAGAGCAATAATAATTGGCAGAAAATTAAATATGAGACAAACACAATTTTAACGTGGAAAACTTCCCTCAAATTGAGAGAATAAAAACCACG	NA	NA
Ca-II-SSR139	227.56	Ca-*desi*-LG(Chr)3	17637329	TCATCAGCAAAATTAACACATTAAAA	(TA)9	(TA)10	GGAGGGAAGAAAAATAAACAAACA	59.09	142.00
Ca-II-SNP552	228.57	Ca-*desi*-LG(Chr)3	17645419	TTTGTTTGTACTTTCTTACAGGTAAATCAACAAGAAAAAAATGTTCAGGTTATGACATTTGGACAACCTCGTATTGGGAATGCTGTTTTTGCATCTCTCT	G	C	GACAGATTGGTTCGGAGCGAATTATACAGGCTCGAGTGGACTACCCTCCGCTTCGGGTCCAAACCAACTGGGTCCGATTCCACATCAGCAGTGTACACCCACGAACCACCAATTTGATCTCCTTGCTCGAAAACCACCACGCGATGTCCTTCTCTTCGGAGCTCGCGCGCCG	NA	NA
Ca-II-SNP553	229.58	Ca-*desi*-LG(Chr)3	17729360	AGATTTGTCAGTCATCCTATTTGTTAGGGATTTTTGGGAGTTCACCAGCAATAATAAGTCAATTGTTTCAAGATCTATAATAGACTTTGACACCACATCT	T	C	AATTGTCAAATTTCAAGTGTGAGTGTTTAGTCTCATCTCAACAATGAACAGATATATAAAATATGTAACTTATATATTTAATGCCTTAAGATTTTGGATG	NA	NA
Ca-II-SNP554	231.60	Ca-*desi*-LG(Chr)3	17860954	AAATATTAATTTTTGTCCTTTTCTTTGTCGGTAAATTGGATGGTACGTACACTTGCATGTGTGCTGTATGTTTAACGGCAAATTATTTGACTCAAAATAA	G	A	CCAGGAAGCATTTTGCAGGAATTTAGGTGAGACTACTAGAGACTGGAACAACTCTCATAAGAGGATGCATCAGTCCTATTTGCCTTGAATTAACTTCTCCTTCAACAAGGTATGTGCTCTGTAGTTGATCAGAAGAAGATTACTGGATTGGTTGAGGTCTGCAGGCCAAAAAGGGATACACTAACAATGTTGCTAGTTGTAGAAATTGATAGATGGAACGTAAG	NA	NA
Ca-II-SNP555	232.61	Ca-*desi*-LG(Chr)3	17992109	CCAAAGAAGCTCTTTCATATGTCCTCCAGGATACTTTTTTTTCCAATTCCCATACAAGTGTTTAACACATAATCGGTGTTCCACATGGGCCTCAATGTTG	C	G	TGCGTGCTGAAGTCTATGATGAATAATATGTTTTTAAGCAATCTGTTTTTAAGCAATTTGTTTGTAAATATTCTGTTTCAGGAATTGGTACCAGCCATTG	NA	NA
Ca-II-SNP556	233.62	Ca-*desi*-LG(Chr)3	18039669	TGAACACAATTTTAAACACAAATACATGTGACCTCCACATAATCTCTCTATTCATTTAATTTCTTTTTCTTTTATATTTCTTTTATTTATTTATCTTTTT	G	T	AATTATTTCTGTCCAAAGGAAGAAGAAAGGAGAAATTATGGAAGAAAGAAAAGATGAATGTGTGAGGTTTATTTTTGTAGAGGAAGACAAAAAAAAAGTG	NA	NA

Marker IDs	Genetic positions (cM)	Chromosomes	Physical positions (bp)	Flanking sequences/Forward primers	ICC 12968	ICC4958	Flanking sequences/Reverse primers	Annealing temperature (0C)	Amplified Product size (bp)
Ca-II-SSR140	234.63	Ca-*desi*-LG(Chr)3	18078319	GTAGTGGGGGCACAAAACAT	(TAA)9	(TAA)8	TGGGTGGGATTCCATTTCTA	59.72	167.00
Ca-II-SNP557	235.64	Ca-*desi*-LG(Chr)3	18086914	TATTAAAATTTACAGTTTAACTTTTTGTATTATATG CATGTATGGTTGTGTTAGCTCAATCCCTTTTGTGC TTCTATTTTTCATAGTGTTGTCAAATAGC	C	T	AATTTCTTCAATTGAGGAAGCTAATAAGCTAGC CTTATATATGGCTCAAGATCAATGTGGAGGAGA TATATCAAATTCAAAGGGAAGCTTATCAGAGTC TGTTTCACCACAGGGTCCTTTAACTCCTCTTCT CATTGGAGGATCTGTT	NA	NA
Ca-II-SNP558	236.65	Ca-*desi*-LG(Chr)3	18170249	TCTATTACGCTTCTATATTCATACAGATCTATTGTA TATCTTCAACAAATTTATACTCTCACATATCTCTTC TGTGCGAGAATTTGTGCATGCGGTTTTT	T	G	AAGTCAACAAAACTATTTTTTAACAGAAAAATAA TTTATAATTTATATAAAATAATAAAAATAAAAGAT TCTCCACAAAAATTTAAACAGCTTCAATTTC	NA	NA
Ca-II-SNP559	238.67	Ca-*desi*-LG(Chr)3	18207408	GGGTACATTTGGGTTTGTACACTCTTCTCTGTCTT TTATTTGTCCTATTCCTGCATAAAAGGACTGTATAT ATGACTTATGAGTTGTCAAGCCAATAATT	T	C	ATAATAAGAATTCCAAAACTATATACATCACTTC GTTCGTTCAACATTCCAGTACTTGCATATTCAG GAGCAACGTAGCTGCCAAAATAAGAATAAGTAT	NA	NA
Ca-II-SNP560	239.68	Ca-*desi*-LG(Chr)3	18262067	TATTAAAATTTACAGTTTAACTTTTTGTATTATATG CATGTATGGTTGTGTTAGCTCAATCCCTTTTGTGC TTCTATTTTTCATAGTGTTGTCAAATAGC	G	C	ATTGAAATAGTGGCCGCTATTGTCCTAAAATCG AAACTAGGATGGCGTCACGGTTTTCATAGTGG ATTCATCAAAATCCGCTATGCTATAGCGCTATC GC	NA	NA
Ca-II-SNP561	240.69	Ca-*desi*-LG(Chr)3	18313268	TTCTTGGTGCAATCTTCTCTAACCCTTAACTCCTTT GATTTACTATATTGCTATATTTGTATATGTGATTAA AACTAGATGCATGTTAAACATATTCTGT	G	A	TTAAATTATGGCATACAAGATTAGCAAGTTTTAT TAGATTGATATGAGATTAACCAAGATGCATACA AGTCAAATTAACAGATTAAACAATATTACTTAT	NA	NA
Ca-II-SNP562	241.70	Ca-*desi*-LG(Chr)3	18363296	ATTAAGAATTTGAAATTGAATTGGAATTAGAAATG GAATCCTCCCTCTTTTTTTGTGGTTTTTATTTTTAT TTATTTTTAATGGTAATTATGTATGTGAT	G	T	CGCGCATCAACACTCTCTCTCACACACCACCAC ACCTACCTTCTCTTTCTCTCTCTTTCTGAAAAAT CGCAGAAAGCACTAACACCTGAAAAGTGATTGT GAGGTGTGTGGAATCGCAGTCTCAGAAGCACG TACTTGCTGAGTTAAAATCAATGGAGATGGTTC AGGTAGGTGTCAGGACACGAGCTCAAGCGGCA TTAGCCATGGAAGCTTCTAGTTCAGCTGCACGA [illegible]	NA	NA
Ca-II-SNP563	242.71	Ca-*desi*-LG(Chr)3	18445099	AATGCATCCAAAACAGGAGAGACAGTGGTTGTTT CGGAAAACTTTTCTAGCAGCGACGGCAGGCAGCA GCAGCAAACGGCGGCGGCGGCAGTAGCAGACG	G	A	TTCCCAACAGTTTGGGAATATAATCCTAGTTTC TCTTTTCTAGCATGATCATTCCTTTTTCTGATGA CTATCACTTCCGTCCACAATCGTCGCTGCTGCC	NA	NA

Marker IDs	Genetic positions (cM)	Chromosomes	Physical positions (bp)	Flanking sequences/Forward primers	ICC 12968	ICC4958	Flanking sequences/Reverse primers	Annealing temperature (0C)	Amplified Product size (bp)
Ca-II-SNP564	243.72	Ca-*desi*-LG(Chr)3	18525106	TGAGTTTTAAACTACAATTAGAAAATCGATTGTTCATCCTTCTGTTTCTTAAAATCACTTCTGATTCTTTCATCAACTCAATGTATTTCTTCCTCACCCT	A	C	ATCCCCATATGTTTTACCTGTCTTTCAGGAATAAAACGTGTACAGTCATTCTTAAGGAAGCTTACTGCGATGAAGCAGCTATCGAAAAACATCCTCTATT	NA	NA
Ca-II-SNP565	244.73	Ca-*desi*-LG(Chr)3	18630974	CTGACTCTCAGTGTTTTTTTTTCCTTCTGTGAGATGGCTTATTTTTACTTCTTTCATGTTTGGCCGCGTCTGTCCTTTTTCTGCATTCCTTCTCTTTCAG	A	C	ATAAGTGATCAGGATTTGGTCAAGGAAGTTACTAAAAACTAACCTAATTTAATAAATGAAAAGGCAAGGCAAATCGTACCCTAAAACAAACATAAGCGCC	NA	NA
Ca-II-SNP566	245.74	Ca-*desi*-LG(Chr)3	18732969	GGAACATATTCTCAAACTTCTGGTATATAACTTGAGAAATAACTTGTATTTATCTTGTTCCCTTTAGTTGATTCGACCAATTCCATATCGAGGTTAAATG	C	T	ACTGTCCTGGTTAAATTGGCAAATTTTTCCCAGAGCAGAAACTGCATTGTCATATGCCATTACATTATCTGAATGAAGTGCATAAGGATGTTGGATCACA	NA	NA
Ca-II-SNP567	246.75	Ca-*desi*-LG(Chr)3	18804984	CTAGTTAGGACTTAGGACTACTATGAATTATTAGTTATTTTGAATTGGGATTTAGGGCTGTAGGCAATATACACTTTCAAATTTCTATTTAGGGCTGTAG	A	G	AAAAAGGCTCATATTAAAATCAAAGCAAAAAACCTCACATTAAAATCAAAGCAAAAAACCTCAATAATTCAAAACAACTAATAAAAGATGTTGCATCCTA	NA	NA
Ca-II-SNP568	247.76	Ca-*desi*-LG(Chr)3	18996239	AAGCTGCGAACGACACTAATGAAAATAGGCACTCTAATGCTAGTTACTTCGTCGACGACACATCTGAGATCGTCGTTTCGATGTCCGTCATGTCATGTAA	A	C	ATTATTAGTATTATTGTAACCCTCTAATTATTTGAAAAATAAAATAAAATTAGTAAATAGAAACCTTTTTGCCCATAAAATAAGTAGAGAGTATCTATGC	NA	NA
Ca-II-SNP569	248.77	Ca-*desi*-LG(Chr)3	19078905	GGTAATTCCTTTTCCTCTGTTTTATGTGCTTTCCTTTTGTTTGGTTTAACTTGTTTACTATTTAACGGTCAATATTATTGGTCCCTGGTTCAAAGTTTAC	T	A	CTTCCCTTCTTCGTCGTCCCTGCCCCATCCCTTCCTCTATTTCCCTCTCTCTTAATCATGTACACCTCAACACGCTTTTCTATCTCTCTCTCTCTCTTCATTCAAATGTGAATGATAATTTCTCTCTTATATCCAAATTTTTCCCTTGCTATTGATCCACCATTTCATTATTTTATTTTATTTTAATTAATCCCAATCATGGGTATATGTTTCTCTTCAACAAAGGTTGTTAGCGG [illegible]	NA	NA
Ca-II-SNP570	249.78	Ca-*desi*-LG(Chr)3	19594047	AGATTTGTCAGTCATCCTATTTGTTAGGGATTTTTGGGAGTTCACCAGCAATAATAAGTCAATTGTTTCAAGATCTATAATAGACTTTGACACCACATCT	C	T	GTTGCTTTAGCCTTATTAGTAACATTTTGTTAGAACAAAACCCTAGTTCAAAAGAGTATAGATACAAAAAACGCAAAAAAACACGCTAGATTTGACCGCGGAGTTTTTGTGTCCCGTATGCTTATCAATAAACTAGAGTCTTATATCCAAAAAAAA	NA	NA
Ca-II-SNP571	250.79	Ca-*desi*-LG(Chr)3	19929129	TCCTACTCTGAAACAATATTCTCTGAATGCCCTACTTTTTCGTTCCTTCAGGAAGTCGTTCCCTGGGGAAGCGACCTCTTGAAGAGGAAATTTTTTAATG	G	A	AAACAATGGCGGGACCTGACCCCTCTCCTTCTCCAAAGATCCTTCTAGCAAAGCCAGCACTCGTCACCGGAGGTACAGTCGCCGGTAAATTCAGTCGTGGCGGCGCCGCCGAAGAAGATTCTTCGCAGCTCCGTTCCCGTCTTCCTTCCGTCGCATCCCTCAACCTTCTCTCCGATTCCTGGGATTTCCACTTCGATCGCTTTCTCCCCTTTTTGACTGAGAATACTG [illegible]	NA	NA

Marker IDs	Genetic positions (cM)	Chromosomes	Physical positions (bp)	Flanking sequences/Forward primers	ICC 12968	ICC4958	Flanking sequences/Reverse primers	Annealing temperature (0C)	Amplified Product size (bp)
Ca-II-SSR141	251.73	Ca-*desi*-LG(Chr)3	20233763	TCTTTTTCTCTTATAAATTGTTGACCT	(AT)12	(AT)11	CTTTCTCAAAATAAAGAGATCTCACA	57.42	118.00
Ca-II-SSR142	253.61	Ca-*desi*-LG(Chr)3	21486044	TCGTTCGAGTTCGATTTTCC	(AG)7	(AG)9	CCTTGCGTTGGTTCAATTTT	60.19	128.00
Ca-II-SNP572	254.55	Ca-*desi*-LG(Chr)3	21603729	GGGTACATTTGGGTTTGTACACTCTTCTCTGTCTT TTATTTGTCCTATTCCTGCATAAAAGGACTGTATAT ATGACTTATGAGTTGTCAAGCCAATAATT	G	C	TAGCACTTATTTTGGTGTAGTGCTATGTATTTT GGTTGAATCTCCATGTAATGTATTATTATAATTG GAGCAGATGACATTCTTGTCCCATCTGCGCCAA TAAGGAAGTCATGTATAGTTCGAGCGGGAATTA AAATGTAATAGTTCAATTAAATAGACTCAGTTCC TGTTATTACTTCAGGAAAAAGTTCAAGTTCTTA GTTAAAATAGCATGTTTAGTAATAGTGGTTTAC ATACTTCTCTTACATCAAAT	NA	NA
Ca-II-SSR143	255.49	Ca-*desi*-LG(Chr)3	21727270	GGGTTGAGAAATTACGGGTG	(TG)14	(TG)12	GGGAGGAAACATTTTCGTTTT	59.29	174.00
Ca-II-SNP573	257.37	Ca-*desi*-LG(Chr)3	23020307	TTTTAGTTTTGGCAGTTATGATCTATAATGGGCAT GCGGTGTCCCACTGTCTATGTACGTGGCTGTCCT CTTGTGGTTTAGGTACACACAGACAGGCACA	A	G	GCAAAAATGGTAAATATTACAATATTAGCCCAA AACCTGAGGGGAATATGATATGAACTACATTAA GTGATAATATATACAGCTTCTCATGGATGGGAA TGTGGTTCTTATTTTCAGCTGTGTACATAAAAG TTGGAAGACTAAGCCTTCAAACAACTAACTAGT TAAACTCGCTGATAACTAAAAATTCCTCTAGGA GCAACTACAATCTTACTTTACATTCCCATCAGTA GCTTTTCCACATCCATTTCCACCCTCCAATCCA	NA	NA
Ca-II-SNP574	259.25	Ca-*desi*-LG(Chr)3	23034508	TTCCCGATTTTGTTAAATCAGTCTCACAAAAACAT CGATTGCGCATTCATTAAGCGTTGGATCGGGCTG ATTTTTGGACAGAAGGTTCGCAAAATTCAGG	G	T	TTAAACAACAATGTGGCTCCTGTTATTGAATTAT AAATAATATTGCTCCTGCTAAGAATGCCATCAT CTCATCTCATACATACATCTCTCACACATGACT ACTACAACTACTACCTATACTATTATCAAGCACT CTTACCAAGAACATCATGGACAAACCCCTCATG AATAGGATTAGTGGTAATGCTTTTATTAACCATA TGCAAATCTCAAATAATAAGACACTGACTGAAC TATAAAATACAGGATACTAATCTGTAAGCAATCT	NA	NA
Ca-II-SNP575	260.19	Ca-*desi*-LG(Chr)3	23165300	CTCATGTGTTTGCAAAAGTTTATTTTTTTAAAGTGC GAACGTTATGCATGCAGGTATCTTCTATTGTTCAT AATCACCTGTCTCAAGCAAGGAGGGTCCC	A	G	ATGAAGTCAATAAGCATACATGAAAGAAAAAAC CCTAGCTCTCTCACCTAATAATAATAATAATAAT AATAATAAACAGCTCTCCACATAGCAACATTTT GTACAGTCATCGCGTCAACTTATAAACTACTAG CTCATATCACCACCGTTTCATGAAAAACTTTAC ATTCTTAAAAACCAAACCTTGCTTCTTGTATTAG TCATTTTTATCTTCCACTGCAGGGGACCTCACC TCACTTAAATTCTATCTATAAACTACACAATCTA	NA	NA
Ca-II-SNP576	261.13	Ca-*desi*-LG(Chr)3	23338444	GTCTCTTCGTTAATTTTAGGGAGCATCTCCAGCAG AAATGATGATGTATTATGTATTGGGCATACCTCTA CTCGACGTGCATATCAACTATCTAAAACTA	A	G	CTAAACTTCTGAGAATCTGTAACATATTTCCAA GAAATACCAAACCAAACCAAAGCTTATTCAAAG AAATCTATCCCAATACAACAGAAAGTTTGTACA GACAGAGAGACCTGAATCAGGAATCATGAGTTT CTCGACTAATGATTGCAGGCAGATACATCATTT TATAACCATCCACTGAGCAAAAACAAACTATGC CGTGGATTCACCTTCTTCCCTTGCGATCAATTC CATCCTACTACCACCCCATCTCCACCCCTCTC	NA	NA

Marker IDs	Genetic positions (cM)	Chromosomes	Physical positions (bp)	Flanking sequences/Forward primers	ICC 12968	ICC4958	Flanking sequences/Reverse primers	Annealing temperature (0C)	Amplified Product size (bp)
Ca-II-SNP577	0.91	Ca-*desi*-LG(Chr)4	86182	CTCGTCGCGATCTCTATTTCGCGTCCCTTTCTATCTGTTGAGGTTTGGATTGATAGAAAGAATACGGTGTTATTCTTGACGAAAGTAGAAAGAAGACTGG	C	G	TTATTGATACATTTATTATATTAATATATTACTCTTGCTATGTGTTTTTCACTAAAAGTGTGAGTCCCTACTCACACTTTTATTTGTAAATATAATAAAA		NA
Ca-II-SNP578	2.12	Ca-*desi*-LG(Chr)4	107312	TACAATAAATATTTATCGGTAGAACTAATCTTATTCGCCAGAACATTATAGCCTTGTCTCGCCTTGTTCTATTATTCTCATAAGTAGCGTGTTTATGTGT	G	A	ATGGAGCACGAACATAGGTTGGCATGCATACGTGGATAAAGGAAACATCGCGATATAGAGGAAAATCAGACCAAACGTGAAGCGCCACGTACACTCAGTT		NA
Ca-II-SNP579	3.33	Ca-*desi*-LG(Chr)4	115020	AATTTTATCTATATTGCAAAAGTTTTGTCCTCTCATAAATTTTGACTCGGTCAAGTTGCTTTAGATATGTTTTTATGATTTAATAGACTCATTTTTAAAA	T	C	ATAAAGGTGTATAAAGAGTTACAATAATATATAACTTTTTAATGCATTTTTGGCTCCCTATTTCATTTTATATTTAAAAAAGAATGAGTTGTCAATTGTT		NA
Ca-II-SNP580	4.54	Ca-*desi*-LG(Chr)4	130269	AATTTAGTGTTTATCATTTTCATAGTTTGGTATACAAGTATATGTATTTTATCGAATCACAATTCTATTCTATCCTTTTTATTTTCAATTAGCAGTTATA	T	A	TGACACAAATTTGCAATACCATGATGAAAGATATCTATCTATGTGTATTACATATCACAATTTATTCGGATTTGATTCCTTTAAAGAGGATAAAGTGAAT		NA
Ca-II-SNP581	5.75	Ca-*desi*-LG(Chr)4	152022	ATGCTGATTGTTGTCTTCCTTGTTTGTTGTGTGATTTCTTTGCATGTGTGGTGGAAGGTGAATTGCTTCCAGCAGATAGAGGATTCAGCAAGCCTCCTTT	A	G	CTAGACAAGCAACTAGTGTAGAATTATCAAAGAGAAAATCTTTAAAAGATTCAGCCTCAGGAAAGCCTCCTCTAAGCACACATCAGGGTGCTGTTGAGAA		NA
Ca-II-SNP582	6.96	Ca-*desi*-LG(Chr)4	173850	GGTTTAGGGGATAGAGCTGTGATTAAAAATGTCTACAGTTTGTGGGAATAAGACTGCCACAAATTATCTCTTTGTGAGGGCTTAGACCGCCACAAATGAT	G	T	ATATAATATTTAATTTAATTTAATTTTCAAATTATATAAATATTAATTTTTTATATATAATATTAATTTTGAAGTTTATAAAATTATTATTTATTTATTA		NA
Ca-II-SNP583	8.17	Ca-*desi*-LG(Chr)4	197709	AACCTGCATATATGATCATGCCCAAGGATGTTTCATTTTCTTTATTTAAATGGATTTTATGTGGAATGACAATATTTGCTGATTGTCAATATAGTAATTC	A	C	CTGTGCAGAGATCATTGAACAAGACTAGATAAAAAGAAATCACTTGTTCTTTTCTTTTGTTGGTTAACATTAATCATTCTAGAGTTGAACAACAAAAGTT		NA
Ca-II-SNP584	9.38	Ca-*desi*-LG(Chr)4	212618	TTTTATCTTAAGTCTTTGTGAACTCTGCTCTAAGTATGTAGTTCAACACATTGTATTCATTTTTTTCAATTTTAATAAATTAGGTATATTTAGTACATTT	C	A	GTTAAGATCTAAAAAAATTGGTTAGATTAAAATTTATGATGGATTATAGTTAATATATATAATCTGTTTCTTACTGTTATTACATAAAGAAAGTTTGGAAA		NA

Marker IDs	Genetic positions (cM)	Chromosomes	Physical positions (bp)	Flanking sequences/Forward primers	ICC 12968	ICC4958	Flanking sequences/Reverse primers	Annealing temperature (0C)	Amplified Product size (bp)
Ca-II-SSR191	10.59	Ca-*desi*-LG(Chr)4	217545	TCCAAACAAAGTGGAGATGC	(AT)6	(AT)8	TTTATAAGTGTAGGCAATGACCTG		58.70
Ca-II-SNP585	13.01	Ca-*desi*-LG(Chr)4	246041	GGTTTCTGGAAATTGTCCTGATGATTGGAGTTTGGCTCAGAAATTGATCTTGAAAGGGTGTGAGCCTTTACCTAGAAGAAGGTGTTTTGCTAAAACTGTT	C	T	TCAAAACTCTTACAATTTAAACCACTCCAATTAACACTCTTATTATTATTACCAACACTTTTCCAAAGAGAAATAGGAAAAGGAAAAAGACCAACCTTTG		NA
Ca-II-SNP586	14.22	Ca-*desi*-LG(Chr)4	258002	AATGTGCATACTGTCAGCTGAAAGTACCGACGAAGGCAGCGTTGTCTGTTGACGGTTAGCACGCCTTACACCAACTCGCAATTGCGATTTCTCATCCCTT	A	G	ATAGGTAGAATAACTTCCGATCGGTAACTTATTATTTTAAAATTTGACAAGTATCTTTACGCAATTTACTTCAAACTAAAGTGTGATGTGTCAATTGTTA		NA
Ca-II-SNP587	15.43	Ca-*desi*-LG(Chr)4	282233	TCTTACTTATATATTGTTCAACATTCGCTTTTTCAATCAATGCGAAACTTTTTACTCACACTTGATACCAATAAATCTTTGTGAGTACATCCATTCATAT	G	C	AATGGAGTTCGGACAACGTGTCTTGTTGATGCAGCTTACGATGGTGCAAGTGAATGAAGATGGACAAAAAAAAAATCTTTTATTTAAGGGAGAGCCTACC		NA
Ca-II-SNP588	16.64	Ca-*desi*-LG(Chr)4	307143	CTATCAATTCGACAACCTATTTTATTTTCCATGTTATCAATTTACGCTGTTATTTTAGCTTTTTTTCAGTGGTATACATAAAAGTGGTCAAGGACAACAA	G	A	TTTATTTTTAAATAAAAACTATTAAATGTTATTTCTTGAGAAAACTATAATTTTGAGTTCCACTACAGTTATTTAATTTAAACATTAACTCGAGTTTTTA		NA
Ca-II-SNP589	20.27	Ca-*desi*-LG(Chr)4	337765	ATAGTGAAGGGGACATTCTTGAAACAGAGTGGGCAACAAAAAGTTCACGTCCCTGTAGTTTCTCATGTTCATAGAAATAGGATGTTTGATTGCTTGCTCT	G	C	CATCATCTCCTCTTACTTGTGCCTTCTGACGCTTGATTCTTTCCACTTGTCGCTGAAGAGAGATTAAATAACAGTTAAAAACACAAAGCAAGAATCACTT		NA
Ca-II-SNP590	21.48	Ca-*desi*-LG(Chr)4	365713	ATTTCTTCACTTCTTCATCTTCGTTTTCTTTTTCTTTCTGTGTTAATGTTAGTTTCTCTTTGGTTTTGCGATTTGTAAATCAAATCAAACGGTCTCAGGG	C	A	TAATAGAAGCAAAAAATGAAGAAATCGAGGAAGGAACTAGGGCGAAGCATGATGAATGTCAAAGATCAAACTCAAATTTGTAACGATAACAACATCTAAT		NA
Ca-II-SNP591	22.69	Ca-*desi*-LG(Chr)4	376968	GAGGCCTTATCAATAATCTCTATGAAAAGTGTTGTCCAGCAGCAAGTATCGTCGAGGTTTAAATCTTTGGGCTAACATCAAATCTCAAACTTATGTTTCG	C	G	TTTCCTTTATTGTTCGTATGCATCCATAATGTACTCAAGTTTTGGTATCAATCTCACATTGTTATGCTGGAAATTACTCTCGTCAAAGCCTTGTACAAAT		NA

Marker IDs	Genetic positions (cM)	Chromosomes	Physical positions (bp)	Flanking sequences/Forward primers	ICC 12968	ICC4958	Flanking sequences/Reverse primers	Annealing temperature (0C)	Amplified Product size (bp)
Ca-II-SNP592	23.90	Ca-*desi*-LG(Chr)4	405477	GTTCGAGTTGTGTACTTCGAAAAGAACTCCACACT<br>TTGATCCTTCTTTGTAACATTCATGAAGCTTAGATT<br>CTTTGAAAACTCCTTGCTTTGGTACTTCC	G	A	CAATAATTAACTGTGCAGATTTCTGAACCTGTT<br>ACTACTCATAAAAGATCACAGAAAAGGTGTGCA<br>ACTCATGTTTACATCAGTCATACAATCCAGACT<br>T		NA
Ca-II-SNP593	25.11	Ca-*desi*-LG(Chr)4	440093	AGTCAATGCCAGCTTGTTGTATGTATCCTTTTGGG<br>ACAAGCCCGACTTTATATTTGTCTAAGCTGCCATT<br>AGCCCTATATTTAGTCTTGTATATCCATCT	G	A	AGAAACGGCGAAAGGCTATGGCAGAAGAGTTG<br>ACAACTTTAGAAGTCAATCAAACTTGGAGCATA<br>GTGCCTCTACCTAAAGGAAAAAAGGTTATGGGA<br>TG		NA
Ca-II-SNP594	26.32	Ca-*desi*-LG(Chr)4	496502	TTAGATAGTCAATCGCGATGGTTCACATCGCAATC<br>GAGGTGGAGTGTGGCGATAAGGGCATGGCCACG<br>ACGATGCAATGCAGCGCGGGGAGGAGACAGGT	C	G	CGCGAGCATAGACCATAGTTTTTCTTCACATCT<br>CTCTCTTTTTCCTCTCTCTCCCCCCAAAAAAGG<br>GTACTATCTTGTTGATCATAAGGGGGTTGCTTG<br>GTTTAATCAAATGATTTGAAAAGGGAGAGTCAA<br>ATCATAACCAAGTTCTTTCAATCTATCTCATGTT<br>TTTTTTATCTATTCATTCACAGTTGAAGTGGAAA<br>TTGAAGAGCTTATTCCTCTTTGCAAGTTGAAGA<br>[illegible]		NA
Ca-II-SNP595	27.53	Ca-*desi*-LG(Chr)4	520748	TGCTTGGCATGCGAGTCATGCTTGTTATACTATGA<br>TGATTGTATATACATACTCAGTTGTTGTTGTTATC<br>GTGCCGTAATTGCTTTGAGGTGTGAATTTG	A	G	ATTAACACCAAATTAATCTCCGCAAATTCACAC<br>CTCAATCACACATCAAACATTCATAACATCAATT<br>ATGAACACATAATTACATCGAAACTTAATCAAC		NA
Ca-II-SNP596	28.74	Ca-*desi*-LG(Chr)4	533777	ATTACTGGCCATCTTACTGAATTTAATTATTTCGTT<br>GAGGGTGTTTTTCGATGTTTAGTTTATTATAAAGT<br>TGTTATAATATATAGTATGGAGAAACTAT	G	A	GAATGGATGAAATTGAAGTTCCACAGTATTTCA<br>TTTGTCCAATCTCACTCCAAATAATGAAAGATC<br>CTGTTACTGCCATAACAGGCATAACATACGATC<br>GAGATAGCATTGAACATTGGTTATTCACAAACA<br>AAAACACTACTTATTGTCCTGTCACAAAACAAC<br>CTCTTCCATTAGATTCTGATTTAACACCAAACCA<br>TACACTTCGTCGTTTGATTCAATCTTGGTGCAC		NA
Ca-II-SNP597	29.95	Ca-*desi*-LG(Chr)4	592505	AATAATGGTCCCTCAAATATTTTACATTTAGAATTT<br>TGGTGTTTGCTTTTAAGTCAACTCATGCGTATCAT<br>TTGAGTTTTTGAATGATTTTTTACAATTA	T	A	TGAATTTTTAAGCACCAAAAGTTAAAAACTTCAT<br>ATTTAATTAATCCCTTATTAAAAATTTCCAAATTT<br>TTATAGGGAAGATTCTTAAAATGTCCTGAAT		NA
Ca-II-SNP598	31.16	Ca-*desi*-LG(Chr)4	646933	GAGCAAGGTCTTCAGGGTGTTTTGCTGAGGAAGA<br>TCACCCAACCCATTGGCTCTATCATCATCATCCCA<br>GGATTTCATCAATGGCAATTCCCACAGGTAC	G	T	AGACAATAACCGAATTTCATCATGAATTAAACAT<br>GGTAGGCTCTGATACCACTTGTTAACTATCCAT<br>GCCAATTACATGATGAATAACCAAGGGCGGAA<br>G		NA
Ca-II-SNP599	34.79	Ca-*desi*-LG(Chr)4	678912	TAGTTTTTTTTAATCTTCGTTTTGTTCTTGTTAAGT<br>TTGCACTTAACTTTACAACTTATCTAATATTAGACA<br>TTAATCGTTAATGATTATAATTATGCAA	T	C	TCTTTTATTAAATATTTGTTTTGATTATTTATCTT<br>TTTTAAGTTCATTTTATTTTTTATAAATAATTAAT<br>GTTAGTCCTTTATCATAACCATTTCAAATT		NA

Marker IDs	Genetic positions (cM)	Chromosomes	Physical positions (bp)	Flanking sequences/Forward primers	ICC 12968	ICC4958	Flanking sequences/Reverse primers	Annealing temperature (0C)	Amplified Product size (bp)
Ca-II-SNP600	36.00	Ca-*desi*-LG(Chr)4	706953	GAGCAACCATCAAAGTTTGTTGCTTAGCGGGAGCCTTCCACCATGGTTTGTCGGCTATACAGGTCCCTTTATAGTCTTAAATAGTCTTTCCTAGAGCTTG	A	G	AGATAAATGCATCTTTGGCTGAGTGACGATAAAATACAGAGTGATCGGCTTCACTACGAATTATATTAAATTCTTGTACTACTGTGCTGAATCTGCCAAA		NA
Ca-II-SNP601	37.21	Ca-*desi*-LG(Chr)4	745785	TTAATCTTCTACAGTCTGGATCTCACCCATCATGATCCGGTTACTCACAACATTAAATCCCAAAACTAAAGGGCTAACCTTTTTCCCCTTCTTGCCCTTC	T	C	AACGGACTTGGTGTCGCTGCTGGTGGCATGGCATCAGCTAAAGCTGCAGATATACAGGATGTGGACCAAACTGAAGGATCCTCCAAAGGAGGTGGAAAGA		NA
Ca-II-SNP602	38.42	Ca-*desi*-LG(Chr)4	754303	CTGCTGGAATTATGTATACTCCTGCCTATCCAACGCTTTTGCAGCATGTTATCCTGCATAACTGCAACCTCTGAACTTTGTGTACTTTATAGGTTGCTGC	T	A	TGACTTGACAATGAATTCAATTACGGTAAATCACAATATCTCATCATGACGAAATGTCAAAGCAACTAAATTTAGAGAACAAAATTGGTTAAGTTTGATCTAAATGCTGCTACAGTCTGTGTAGGAAATAAATATCCTAAATCGAACAAAAGCATTTACAATGTGCGACCAAAATGTATGTACATCTACCGCAACAACCTCATGCATAAAATTTAATTGTAATCTTATCTTAA[illegible]		NA
Ca-II-SNP603	39.63	Ca-*desi*-LG(Chr)4	769894	TTATCATAATTTTAAGCATCAATTTATGCTAGTTATTTGACTTTTGAGGGAAGGCACCTTGAATTTAGGAAGAAATTCCCCTGCCTACCCATCTTTATCC	G	C	CAAAGTCCCAACATTCAATATGGCAATCTTGGTTTCTTGCTCCTCATTTCTTCTACACTTCCTGTCCCTCTTTCTTGCATTCAACCTTGCAAATTCTGAACCATTCCTCGGCGTGAACTATGGACAAGTCGCCGACAATCTTCCACCACCGTCAGCCACCGCGAAACTACTACAATCAACTTCAATAGAAAAAGTCCGATTATACGGAACCGATCCAGCCATAATCAAAG[illegible]		NA
Ca-II-SNP604	40.84	Ca-*desi*-LG(Chr)4	846652	AGCGGGGCCAAACTTAAATACCTCTAATTTTGCCGTTGGTTCCGTTGCTGTTCGGATAATTCATGTGTTGTGTTAATACATGTTTTTTGCGTATATTTCT	T	A	GATTAACTACATGTTCTAATCTAGACAAAATTTCCCTAGGCTATGAAACTTTGTAAATCATGATGAAAGCATGCATAACACCAAAAGAAGAGAAAATGAA		NA
Ca-II-SNP605	42.05	Ca-*desi*-LG(Chr)4	909120	ATTGCAACCTCCAAGGAGTAGGACAAGGTTGTTTTTGAGGGGGGGGGGGTGTATTGCTAGGATACATATTAAGAGAGTTAGTTTCTAGTAGTTAGTTGG	C	T	GAAACTCTACTCAATTCAACAAATGTTTACAATCTTCAATGATATCCAATCCAACTACAAAACTCCCTATTTATACACAATAATCTTAACAAAGCACATA		NA
Ca-II-SNP606	43.26	Ca-*desi*-LG(Chr)4	937976	GACTTTGTAAAGATTGCTCTATCTCCCAACAAGCCATCACTGCTTCCATTGCATGGACTCTGACATGTTGTTGTAGTTGTTCTTTGAAGGAACACAGCAA	T	C	ATATGTATCTGCGAACAAGTTCACAGCATCCAAATTATTAAATACGATTGAATTGCAATTTTGATGTTTCGCGTTTGATGCTGCAGTCGCACTATGTTTT		NA
Ca-II-SNP607	44.47	Ca-*desi*-LG(Chr)4	984185	GGAACAAGCTGAGTTAAGTCAGCTACAATCAACTGCAGTTGCTTCACTACCTAACTCTAATGGTTCACGGATTCCTCCGTGTAAGTCTATTATGCCCATC	A	G	ATTAGAGTGAGACAAGGAACCTGATATGCTCTTAAAAATAACTTTTAAAAGACTGGAAAACAGAAATAATAAGTTCACTGCAAAGGGATTAAAACAATGT		NA

Marker IDs	Genetic positions (cM)	Chromosomes	Physical positions (bp)	Flanking sequences/Forward primers	ICC 12968	ICC4958	Flanking sequences/Reverse primers	Annealing temperature (0C)	Amplified Product size (bp)
Ca-II-SNP608	45.68	Ca-*desi*-LG(Chr)4	1018306	CTGCTGGAATTATGTATACTCCTGCCTATCCAACGCTTTTGCAGCATGTTATCCTGCATAACTGCAACCTCTGAACTTTGTGTACTTTATAGGTTGCTGC	T	A	TTCCTCCAAGCTCAACAGGTACAAGAGGGATGAGGGAGTGTAGAACATCTGCAATCAGGGGGCGGTAACATGGCTCTGGTTGCACACATAACACAGCCAC		NA
Ca-II-SNP609	46.89	Ca-*desi*-LG(Chr)4	1147249	CCAAGGCTTAACAGATTGTTCTTCATGTTTGGCACATACAACACATCACATATGAATGATTGTTTGTCATCTCTCCTTTGAATCATCACTTTTCCTACTC	C	A	TTCAAATCACACGACTGACCATAAATATTGGTTTGTGAGCATTGATGAGAAGGTGAAAAGGGAGATCAGATTTGCAGATAATAGTTCAATAACTACAGAA		NA
Ca-II-SSR192	48.10	Ca-*desi*-LG(Chr)4	1213958	TGGTCGTGCTGCTGTTTAAT	(TA)16	(TA)14	TTCAGCTGTTTTCTTCCACG		59.35
Ca-II-SNP610	51.73	Ca-*desi*-LG(Chr)4	1346021	TTTTTTTGTGATTTCGTTGTCTGAGCGCGACGTTTTTATTTTTTTATTTTTTTGTCATAGTGCGATGCTTTTGGGTTTAGATTGACGAAGTAGTTTTAGT	T	C	AAAGGCACTCCTCCAAACTCTCTAACATGTCCTTGTAAATACTTATGTCTTCGGATTTGTAATGTTGATGACGTCGAAGTCATTGATTGAAATTTGTAAT		NA
Ca-II-SNP611	52.94	Ca-*desi*-LG(Chr)4	1749663	GATGATGTCGCTGAGCCTGCACAGATGGAGGAGGATGTCCCTGAACCTACACAGATGGAGCAGACTGAGGAGCAGTATGTTGAGCCTGCACATGATGATG	T	C	ATTGTGTCAACACAGATATCACCACCGATCCTCCGGGGAATCCGGGTTACTGATTGTGCTTAGGCTCAACATCATCAGCAACATCATCATCTCAACATCA		NA
Ca-II-SNP612	54.15	Ca-*desi*-LG(Chr)4	1785296	GGATGAGGACGGGTTATAATTATCGTGAGCTTGGGCATTTTGGATGACTAGGGGTATCCGGATGAGTGTTGATGGTGACAGTAATGAACTTACTCAGCGT	A	T	ACTCCTTCAGCTACTCCATTACACTTCACTTCATCACCCTCGTGTAAACCGTGTTCCTTACCATCAACTCCTTCTTTTAAGTTGTTTTTCCCATCTTGCA		NA
Ca-II-SNP613	55.36	Ca-*desi*-LG(Chr)4	1850658	CGATTTTTATGTAGGAGTAATGGTTTTTCAAACTTCAACACCACTGATTTAACAAATTTGTTCCACCACGATTTGACGAGTTTTTGTAAAAGTGTGTAAT	A	G	TCATTAATTTTTCATTTCTTTTGAATTTACATGATATTTAATAATAAATTTAGAATGTAAAATGTATAATAGATTAATAAAATAGTAGTTTTAAAAAAAT		NA
Ca-II-SNP614	56.57	Ca-*desi*-LG(Chr)4	1895597	CTCCATTCAAACGAGTTTTAGATATGCCAAAATTTACAAATTTCTGGTGCTTTATTGTCTGAGTTGGCTAGTAGGTGGGATGGACGTAGTGGGGGATTTA	A	G	TTCCTCATTTTCATATTCTTTCAAAGACTCACCTACTATTGGTAGTCCTATAGCTAAACATACGTCACTTGGAGTAAAATTAACTATCCTACCTCTAATC		NA

Marker IDs	Genetic positions (cM)	Chromosomes	Physical positions (bp)	Flanking sequences/Forward primers	ICC 12968	ICC4958	Flanking sequences/Reverse primers	Annealing temperature (0C)	Amplified Product size (bp)
Ca-II-SNP615	57.78	Ca-*desi*-LG(Chr)4	1931418	AATAATACTTTAAGTTTGTATCTTACTTAGTTTAAATATTTGCTTTGTAAGAGAGTGGTTGTACTGGGGTGTTTGGTGCTATTTTTCCCAATAGTATACT	C	T	CATGTAATTCCGTTAAATACATTATTCTCAAACACACAAGATTCATCATACCAAGATGGCGTGACTGCTTCAAATGCGTGGAGGCTGCAATTCTAAGAGATCTGTTGATCCCCTTCTCAAGAATCTTGCTTTCTTACACAATGGCATCGGTAGCAGTCACCAAGATATATCCAGAAAACAAGGAATATCTTTTGCCATTCA		NA
Ca-II-SNP616	58.99	Ca-*desi*-LG(Chr)4	1983063	TGCGGCGGTGGAGGCTTCGTCGGCAGACAATGTGCCGTAGCGTTTAGAAAGGACTGAGGGAGTGGAAAATGTTTGGATCAAGCGGTTTATGACGGCATCC	C	T	CAATACTCCATACCCGTTCCACCACCATCAACGGAGGCTCCACCACTGCAACCAAACCTTACCGCCTCCTTCAGTATATGGCCTCCCACACAGCGCACTC		NA
Ca-II-SNP617	62.62	Ca-*desi*-LG(Chr)4	1995574	CCTGGGGCGGTTTTAATCAACCGCCGTCGTCGTTTAGGGCATAAACGTGTTTTATTTTTCCAGATTTTGTAAGTTCATTCCTCACTCTCTCTTTCGTTTA	G	C	TCTTCGATTTCGTTTCCTTCAGAAGTTTTAGGGTTGACAGATTTGGGGGTTTTCAGAAATTTTAGGGTTGTGAAAGGATGTGTACGGGGATAACAAAGCA		NA
Ca-II-SNP618	63.83	Ca-*desi*-LG(Chr)4	2083734	AATCGCTCGTAGGAAAATATTTACCGACGGACAAGCTGTGGGACACACTTACCTAGGGATTGTCCGTGGGTAATATGACCCACTGAGTATCTGTGGGTAA	T	C	AAATAGTAGACTTGTCACGGATGATCCGTGGGTAACGTAACCTACGACTTTTCCGTCGATAACTATCTACTATTTTATCTACGGCAAATCTATGGGCAAC		NA
Ca-II-SNP619	65.04	Ca-*desi*-LG(Chr)4	2111259	CACACATATTCCCTCTCTTTCTTGTGGAAATTCTAAGCTTTCGTTTCTTCATCTTTATGAGATTTTTTGAGGGCTATATAGTGTTACGTGGAAGGAAAAC	A	G	CATTTTTCTTTTAAGACATCCAGACATCATATCAATATCTTACGAACCGCTAATTTAATATCTAGCCTAACTCTGCATGAGGTAAAGTCCTTACCTTGAA		NA
Ca-II-SNP620	66.25	Ca-*desi*-LG(Chr)4	2192586	ATTATTTTGCATAAATTATGGAGAGACAAGTGCAGATGAGACTTCTACTTTTGGTGGAGTCCAAAGTTAGTGAAGATGAGACTTCTACTTTTTGATGCAG	G	T	AAACTCATAAATCTATTCCAATAAATCTCATTGATATTTTAACAAAGTGTCCAATTTAAGCTTCCATGTTTCCCTCCTTTTGAATCTTCACCAACTTTAC		NA
Ca-II-SNP621	67.46	Ca-*desi*-LG(Chr)4	2220409	TTTGGCAACATGAGCAAAAGTCTTTGAGTAATCAATGCCATATGTGTGAGTAAATCCTTAGCAACCAAGCGTGCCTTGTATCTTTCAACGGTGTTAACAT	A	G	GATGAGAGCTCTTGAGAAGCATCAAACTTGGAGAAGCATGACGTTACCTACATGAAAGAATATTGTTGGCTATAAATGGATATTTACTATGAAATATAAT		NA
Ca-II-SNP622	68.67	Ca-*desi*-LG(Chr)4	2259951	GCTCACGCCACATGCGAAGCTCAATGGTTGTTATATTTGTTAGCTGATTTCCAGATCTCTCATTCAGTACCTGTGCAGATTTTTTGTGATAATAAATCAG	T	C	ATGAATGACCCCAACTTGCATTTTGTCGCGAACAATTTGGCAATCCATCTCAATATGTTTGGTGCGCTCGTGAAATACCGGATTGGAAGCAATATACATG		NA

Marker IDs	Genetic positions (cM)	Chromosomes	Physical positions (bp)	Flanking sequences/Forward primers	ICC 12968	ICC4958	Flanking sequences/Reverse primers	Annealing temperature (0C)	Amplified Product size (bp)
Ca-II-SNP623	69.88	Ca-*desi*-LG(Chr)4	2295325	AAACTTTTTGTTTTTAGTTCCTATAAACGTTTTACTTTGTTTTAGTCCTTCCCCACTTTTTGATTTTGGACTTTGTTTTGTTTTAGTTCGTGAAATATTT	A	G	GTTGGAATTGATCCTTGTAATATTCATTTTAGTCCTTATGTAGCTCATATTGGAGGGACTAAAATCAAATGTTCTACATGGGGATCAATTCCAACACAAA		NA
Ca-II-SNP624	71.09	Ca-*desi*-LG(Chr)4	2301546	GATAATTTGTTTATTTGTTTTACTAATCTTTCATTTTAAAAAACAATTTTTTTTTCAAAATTCTGGTGCCGACTTGTTGGGTGTGTGGTGGGCTCAATA	T	C	CGTGTTATGAAAGAGTTCTAAACGAACAAGAAAGCCCACCCGGTCCAATCCTTCCAACATTGGTGGGCCCATTATGGCCCGCATCAAGCAACCTGAACGT		NA
Ca-II-SNP625	72.30	Ca-*desi*-LG(Chr)4	2432885	TTCTACGGTTATTGATCTTGACTCCCCTTTTTTTCGTCTTTTTAAAATTCAGCCTGATTACAGTGATTTACATACTTTTGGATGTGTGTGTTTTGTTCAC	T	C	GTTGTCAAATGTTTGGATTTTGGAAGATTTATGTAAATTATTTGAAGGGTGGTAACTGATAGATAGAATTTATCTGTCATGCTGTGTCTATTTTCTTTGAGAGCAAGATAGAGATGAATGGTGTTGTGAAAGCACGTTGAAGTGCCAAGT		NA
Ca-II-SNP626	73.51	Ca-*desi*-LG(Chr)4	2510477	CCCAGATCTATCCTCACTTCTCTCTTCAACATTTTTGTTCATCTTCTTCTTTTTCTGATCTGTCTTCACCTCTTCAGCATTTAATTTATTCTCACTCCCC	G	A	GAGATAGAAAAAAAGATAAGCAATGGTGTTATTGATGGGGAAAATGGAGGGCTAGTTGCTTCTCGAGATATAGAAATTAAGTCGAAAAAGAGATACGAAG		NA
Ca-II-SNP627	74.72	Ca-*desi*-LG(Chr)4	2618893	CAATCCGCTCCGTCGACCGTTCTGGATGTCGCCGTGATGATCGTTATCTCTTAAGGTAAGTCACTGAATCGACTAATTTTCTAATATCTTTTCATTTTCT	A	G	TGACTTCATTCAGTATGACTCCATTCAGTGAAAATGGTTAGGGTTTTCAATACAAATTGGGGCTTTTTCAGTAAGACTCGATTTCGGTAAAAATTGATTT		NA
Ca-II-SNP628	75.93	Ca-*desi*-LG(Chr)4	2673525	CTATTTCACATAATGTAAAGTTAAATTTGTTTTTTACCATTTTCTCATTTTTAGAGCAGTAAACAATATTTCCAGTAGGTAAGAGTTTTCCGGTAACTTC	T	C	GATTTTTAAGTTTTTCGGAATTTACATTCTGTACCGGAGAACTTAAGAAAAGTTTTTCGGAAGTATGCTGTGTACCGGAAATCTTGTTTTTGAGTTTTCC		NA
Ca-II-SNP629	77.14	Ca-*desi*-LG(Chr)4	2761588	TAATAGGTATTTTTCTTGTTTTTAATGTTAAATTTGAAATACCCACTACCTGCACTAGTCCCATTTGGCCTAGTTTCAGGTTGCAGCTGGTTGGTGAAGT	T	C	CCAAGTTGACATCTAAGGGAGAATTAATATAAGTGGTAGATTGTGTAGCATTTTGTTTTTCTTGGATTAAATATTTGTCATTTTGTCTTGTACCCATGAC		NA
Ca-II-SNP630	78.35	Ca-*desi*-LG(Chr)4	2774441	TTTCCAGAACTGCTTCAAGATCAATCTTCGTTTTCCAGAACTGCTTTCTTTGATCTGATTTGTGATGTTTGATACCTATCTTGTTTTAACATACTCAAAA	G	A	TGTTGAGACAAAATCTCAATTTAATGTTTTGATGAAAACAAACAAAAAGTTAATTAAGAATTCTAATTATGATTCTAAGTGTTTTGATATTTAACATGTG		NA

Marker IDs	Genetic positions (cM)	Chromosomes	Physical positions (bp)	Flanking sequences/Forward primers	ICC 12968	ICC4958	Flanking sequences/Reverse primers	Annealing temperature (0C)	Amplified Product size (bp)
Ca-II-SNP631	79.56	Ca-*desi*-LG(Chr)4	2792460	TAGTGGAAATCTAATGGTTGTGAGGACTGGACTAGCCCAAATTGGGTGAACCAGTATACCTTTTTTGTGTCATTTTCCTATCCTTATCTTTAATTATTTG	T	C	CGATTATGGAAGTGTTCATGGTTTGAAGAAGGTGGAAGAGGTTGACAAAATATGGAACAATAGAGCAGCAAACGAGAGAGGAAAAGCAGAAAGGTAAGACTAAGAAGAACAAGAACGTCACTCTCCTAGTGCATATCTTTGTTTCTT		NA
Ca-II-SSR193	80.77	Ca-*desi*-LG(Chr)4	2828033	CGATTCTTTTGTCCATTTCCA	(AATA)6	(AATA)8	CCCATTTTGTGCAGATGTTG		59.92
Ca-II-SNP632	83.19	Ca-*desi*-LG(Chr)4	2851816	ACGGATTTCGGTCCATTTTTGTCAGGTTGTACAAAAATCGCTCGAAAAGCCCAGATGATAGCATTCTTCTCCAAAATTTATAACTCTGTTTCGGGTTTCG	T	C	TTTCATCTGGGCTTTTCGGTCGATTTTCAGACCACCAAGACTAATATGATCCGAAATTCGTATTTCCGAAAAAGACAATTGTTAATGTTTAAAATCTAGT		NA
Ca-II-SNP633	84.40	Ca-*desi*-LG(Chr)4	2879232	TTGTGTTATTTGCACCATTTTGCCCTTGAGAGCCAACTTGTCCTTGAACTGGGAAATCTATGAAGGGAATGTTCAACTCCTGACAGATGACCGTCTGAAT	A	C	TCTCTCTCTCTCTCTTTAATCTAGTCCTTTAACATAGTTATTTTCTATTAAGGCATGGGTATGGGAGGTGGCAAGCTATTGTTGATGATAAGGATCTGAA		NA
Ca-II-SNP634	88.03	Ca-*desi*-LG(Chr)4	2919277	GTGGTGCCAGATTCCACAGGTAGAACGGCAGGTAATGTTTTCGTTTGCTGCGGATTTACATGTGACTATATATCCGCAGCAAACTTCACAATTTTCCTGA	T	G	ACTATTTTATTGGTATGACAATTCGCAAGAAAATCCGCAGGAAATTACCTGCGGAATTCGCTGTGAATATTTACAGCGGGGAACATTCCGCAGATAAATC		NA
Ca-II-SSR194	90.45	Ca-*desi*-LG(Chr)4	2986218	AAAACACGAATGCTCGCAC	(CA)8	(CA)9	TTCTGAATTGCATGTGAATGG		59.86
Ca-II-SSR195	92.87	Ca-*desi*-LG(Chr)4	2993847	TCACTGACTAAGTGCATGTCCA	(TAA)5	(TAA)6	TATTTCAACCTTGAAGGGGC		59.37
Ca-II-SNP635	95.29	Ca-*desi*-LG(Chr)4	3050876	GAATCATAATTAACTTTCTTAATTAACATTTGTTTATTTTCCTCAAAACTTTACATATGGAGTTTGTCTCAACACTCTCCCCCTTTTTGATGATGACAAA	T	C	TTTAAATTCTGTCAATTTAAAAACTGCATAAATTGAGGGGGAACTTTTAAGCTCTTTAAATACTTGATATTAGAATCAGAATTAAAATCTAAATTAATAT		NA

Marker IDs	Genetic positions (cM)	Chromosomes	Physical positions (bp)	Flanking sequences/Forward primers	ICC 12968	ICC4958	Flanking sequences/Reverse primers	Annealing temperature (0C)	Amplified Product size (bp)
Ca-II-SNP636	100.13	Ca-*desi*-LG(Chr)4	3089120	AATGGTTTAGGAGGTTTGATGAGGGTGTTTTGGAGCACAGAGGTCCTAGTGATGACACACTGTGAATACCATGTCTCAGATAGGCAGTGGGCTCCTTCAT	T	C	TTCAAAAGGATCTAAAGGCATGACAAATTTCAGTATGGTACTGAAAGGGGTTCATATGGAAATTAATCCCACCACTTTATGTCAAATACTTGATATACGT		NA
Ca-II-SNP637	103.76	Ca-*desi*-LG(Chr)4	3142206	ATATTAATGACATACCTATTAGTCGTAGTATTTCCTAGATGCAATACTCGATTGGTCCATGCTTCAACAAATTTTTCCTTATGAGGATTCAACCATGTGT	T	C	GAAAATTGTTTATTGTTCTGAAGAGAAGGAGTACGAGGAACTTTTGCATATATTTGAGCAAGCATGTGTCGATAATATTATTTTCTTTGACTACGTCAGA		NA
Ca-II-SNP638	104.97	Ca-*desi*-LG(Chr)4	3202194	AACCAACCTCCGGCCTTGATGCTAGAGCAGCTGCAATTGTAATGAGAACAGTAAGGAACACCGTGGACACCGGACGTACCGTGGTGTGCACGATCCATCA	G	A	ATATTGAAGCAATAAATTCTTTAATATGTAGTACTTAAAACAAAATGATGAGACTGAGAGTTCTTACCTCATCAAAGGCGTCAAATATGTCAATACTTGG		NA
Ca-II-SNP639	106.18	Ca-*desi*-LG(Chr)4	3220236	TGCTTTCTCCCTCTCCAGACCTCTTTTCTCCGATCCGACCGTTGAATACGAAGACCTAAATGTTGGAAACCTGCTGTCCAAAAATCAGCTCGATCCAACG	G	A	TAGGAAAGACAACCACCAAAAGAGAAAAGATCAGAAGAGTAACCCTATTCCCAATTTTGTTAAACCAGTCTCAGAAAAACATCGATTACGCATTCGTTAA		NA
Ca-II-SNP640	107.39	Ca-*desi*-LG(Chr)4	3270115	GTGGTTGATTGTACCCTTCAAAACATATGTGAGTTTCAATAGTCCATTGACTCTACTCAATATTTCTTCTAATGATAACTTATGGGCATATCATCTATTT	C	T	TATTGCATTGAACATACAAATGAGTTTACCTTGAAATACAGAGGAAAAAGTTTGTGGTTTGATCGTAGGTTCTTACCAGAAAATCATGAGTTTAGAAATA		NA
Ca-II-SNP641	111.02	Ca-*desi*-LG(Chr)4	3301962	TACCATCCTTCATGTTTTACATCTTTGCAACTCAATGGGAAGGGAAGGAAACAACTTTTTGTGTTATACGTGATAGTGATGGTCAAGAACATTTTTGTAT	A	G	AAAAAATGATAAAATTTGTTAAAATACAAGAGTCATAGAGTGGTGATATTGTGTGACTACTGATCACAACTTTTTATAGAAATAGTTGCTTGTTCATAAT		NA
Ca-II-SNP642	112.23	Ca-*desi*-LG(Chr)4	3363537	ATCTCATTATTTCAAGCAAGGTTCTTTTCATCCTTTCAGCCATTCCATTTTGCTGAGGTGTGTTTTTTAAAATCCTATGTCTATTCACACCATGAGTTTT	G	T	GATTGGACGTCTATAGTTGAGAATAAGACTGAAAAAGGATTAAAACTTTAAGAACAGACAATGGTCTTGAATTTTGCAGTGAAGAGTTCACTAGTTATTC		NA
Ca-II-SNP643	113.44	Ca-*desi*-LG(Chr)4	3390135	TACTATTCCTAACAGAATCATATTGGTTCTGGTTAGGTATTGTTCATTGATTCAATTCAGCTTAGTCATAAATACCAGTGGTTCTATCAAATAGGGATAG	A	C	AGGTAGCCTTGCTGATAAAATTTATCATTAGAGAAGAGTGAAAGAATCTAAAGAATATAATCAAAACATGATTAACTGTATTATTGATTTGCATCATAGT		NA

Marker IDs	Genetic positions (cM)	Chromosomes	Physical positions (bp)	Flanking sequences/Forward primers	ICC 12968	ICC4958	Flanking sequences/Reverse primers	Annealing temperature (0C)	Amplified Product size (bp)
Ca-II-SNP644	114.65	Ca-*desi*-LG(Chr)4	3450900	TGTGATGGTTCATAGTGTTGTGATGGTTCATAGTGTTGTGATGGAGAATATAAGTCGAGTTGATAAGGTATGTTTGAGGCCGATGGTTGGTCAATGTTTG	C	T	CGACGACAAAGATGTTGAGTTGATGTTTGATGTGTATGCACAATGGTCACAACTCGGCACCATCTAGTTATATGTTACCTTTGAGTATGGTCCTCCATCA		NA
Ca-II-SNP645	115.86	Ca-*desi*-LG(Chr)4	3480006	CGATTTTTATGTAGGAGTAATGGTTTTTCAAACTTCAACACCACTGATTTAACAAATTTGTTCCACCACGATTTGACGAGTTTTTGTAAAAGTGTGTAAT	G	A	AAACGCAACGCAATGATGTACAAAAATCATCCATGGCTGCTAAAGTTCATAAAAAACACAGCTCTTTCTCAACAGATGATCAGGTGTGCGTTTACTCATTTCATAGACTCAGTGCGAATGGCAGAAAAAACCTCTCTATGATGCATCAGTGAAGATGCCAGATCCAAAGAGTTCTCCACCAGATTCTAACTCTAAAGAATTTTCAAATGGTGCAATGGATTTCTCTAGCTCCTCGTCAATCAT		NA
Ca-II-SNP646	117.07	Ca-*desi*-LG(Chr)4	3510460	CACCCGCAGCCCGGATTAAACCGAGCAGCCCGTAGCCCAATTTCTATAACGGGCGGGTCAAAAGCGGGTACACGGGTCTACGGATAAATAAACATGGATT	C	T	TATAGTTAGATGATTTTTTTTCCATATAATTTGAATCGGGCGGGTGCGGATATCCGCAGTCCAAAAATCTTTAACCGAACCCGCCCGTTATAAACCATGT		NA
Ca-II-SNP647	118.28	Ca-*desi*-LG(Chr)4	3524948	ACAAAGGCAGACGTTTGTGACGGCTTTTGCCCTCACAAAAGGCAACGTACTTTTCAAACTTTGTGGCCGCCACAAATGTTTGTGACCACCGTATTTGTGG	C	T	GAGTCTTCACAACAAAAAAACAAGGATTTTGTGAGGGCCAAAAGCCCTCACAAAGGGGAAGAAGCAGCCACAAAGGGGGAATTTGTGGCGGCCTTAGACA		NA
Ca-II-SNP648	119.49	Ca-*desi*-LG(Chr)4	3612793	ACGACATGTGTGTCGCTGTCCACCGACTTGTATCAGACATCAAACACGTTTTCAATCTAGGTGTTACATAGGTTAGGACTTCCACTAATAGACTAGTCAT	T	G	CTCATTCACCTGAATCCTAGAAGATTTCTTCACACAAAAATACTATTTTCATGTTACTTAACTTGTAAGGAGCATAAGCCCAAAAAAAGTGTTCTACCCA		NA
Ca-II-SNP649	120.70	Ca-*desi*-LG(Chr)4	3645705	GTTTATGTCCAAACTCGATATGGTCATTAGGAATTAGTCAAGTTGATTTTCAAGTACTCGAAGAGGTACAATAGATCTTGGTATCATATTTGATTGCGGT	T	C	TCATCTTGCAAGAGTAAATACATATCTTGTTGTAGATTTCTTATCATTCATGTCACCAACATAGTATAATCAACATATCATATAATTGAACGATCACCTT		NA
Ca-II-SNP650	121.91	Ca-*desi*-LG(Chr)4	3666024	TTGCTTCATATATTTGAATGAAAAGTGTGACTTTATCTTTATTTCGCTCTTTCGACACACTCAAAATGAATCTTGGCACAAACTTATACTTTGTCATATT	G	A	TTAGTTTTTGATGTGGAACACACAATCATGATGGAGTGGATACTTTAGACGGTCATTCATATTTGGGGCATTTAAATTAAAATGATTGAAAATTTGTTAA		NA
Ca-II-SNP651	123.12	Ca-*desi*-LG(Chr)4	3702341	AATTGAGTCATTGGCCAATACTTATTTTATATGTTATTATCTTTTATCCATCTTTTGTTTGTTGAATTGAAAGACTTGAAGTTCTATATCTGATAATAAT	C	A	TTTATAAATATTTATCTAATCTTCTTTTTTAGTTTTCTAGTGTTCTAACAGCCATTAAATTATATATTTATACATAATGAAATCAATCTTCTTAACGATA		NA

Marker IDs	Genetic positions (cM)	Chromosomes	Physical positions (bp)	Flanking sequences/Forward primers	ICC 12968	ICC4958	Flanking sequences/Reverse primers	Annealing temperature (0C)	Amplified Product size (bp)
Ca-II-SSR196	124.33	Ca-*desi*-LG(Chr)4	3728850	AATTTAACGGTTGTGATTAACTGAT	(AT)6	(AT)7	CCGACACAACGTTATTGAATG		57.34
Ca-II-SNP652	125.54	Ca-*desi*-LG(Chr)4	3735411	TTTAGTTTGACCGAATTTGGTTCAAACCGGTCTGATATGATTTAGTCAAATTTGGTTCGAATCATCTAGTCCAATTTGGTTTGATTAGTTCTGATTCCAT	A	C	CTTCTCGTGTTTGCACCTTCAAACCAATCACACTAAATCAGACCAAATCAAATCAAATTGAATCATATCATACCATGCCAAATCAAATCAGGTTGGGTCA		NA
Ca-II-SNP653	129.17	Ca-*desi*-LG(Chr)4	3789814	GAGCTGCCTGAAACCAGTTCATCACAACTATTGTGAGGGCCAAGCCAAAAGTGTATCCTAAAAATGCACTCTTGAAATATTGGGGCTGTTTTCCTCTAGA	A	T	CTTACATTTAATAATTTGGTTGGCACTCACTTGTATTTATTTTGATGGTAAGTGTTTTCCCTTCAGGTATCTTTGTAGCATTGGCCTTGCGTTTTGATGT		NA
Ca-II-SNP654	134.01	Ca-*desi*-LG(Chr)4	3863231	TTCTACGGTTATTGATCTTGACTCCCCTTTTTTTCGTCTTTTTAAAATTCAGCCTGATTACAGTGATTTACATACTTTTGGATGTGTGTGTTTTGTTCAC	C	G	GAAACATCATAACACACAAAACCCTTATGAGAGTGACTATATCCCATAAATGCGCATTGAGCAGACTGCGCTCTAAGCTTATGTCTTTCAAATGGAGGTA		NA
Ca-II-SNP655	135.22	Ca-*desi*-LG(Chr)4	3932848	ATTACTGGCCATCTTACTGAATTTAATTATTTCGTTGAGGGTGTTTTTCGATGTTTAGTTTATTATAAAGTTGTTATAATATATAGTATGGAGAAACTAT	A	G	CGTATATAAAAGAACATTAAAAGAGTTTCTTGGTGCATAAGACATTAATGTTTGAGGGTATTTACTTTACCCTCACAATACATAAAAACACTTGAAAAAA		NA
Ca-II-SNP656	136.43	Ca-*desi*-LG(Chr)4	3954869	ACATGTATCTACCAGCTGCTACTGCAGCTAATGGGATCAAATTCCCTCTTCCTCAATTCAAGGCTGGAGCAAACGCAGGAAATATGGCTCATATTGGAAT	T	C	ACTGAGACACAGCAAGATCTTCATTTCCCGTGGAGCTTCCAGTATTCACTGTCGGACTAGGAGCGTATCCAACAGGTGGAGTGATAAATGATCCAGAAGG		NA
Ca-II-SNP657	137.64	Ca-*desi*-LG(Chr)4	3975881	TATCTTCTATTTCCATTTACGATTTTCAAGTCTTGTTGTGTAGATTTCAGGTTTTTATTGTTATGTGAAATGTGTTTTCTGTTTGCACTTTGGAGATTGT	T	A	GCAGCAACCATATAAATTAACACTTACATAATAATCTCCAAATAAGCCACTGTCACATACACAAATTAGAGGTGTATAAATTAAAATAACTACTTGACAT		NA
Ca-II-SNP658	138.85	Ca-*desi*-LG(Chr)4	3989112	CATGAGACACCTCCTTGATCTTCTTCTTTTTCTTCTCCTCCTTTTCCTTTTCTTCATCTACTTCCTCAACTTTACCCTCCTCTTCCTTCTTCTCTTCCTC	A	G	GTCTTAAGGATTTGATCAAGAAGCATTCTGAATTCATCAGTTATCCAATTTCTCTTTGGATTGAGAAAACCATTGAGAAGGAGATTTCTGATGATGAGGA		NA

Marker IDs	Genetic positions (cM)	Chromosomes	Physical positions (bp)	Flanking sequences/Forward primers	ICC 12968	ICC4958	Flanking sequences/Reverse primers	Annealing temperature (0C)	Amplified Product size (bp)
Ca-II-SSR197	140.06	Ca-*desi*-LG(Chr)4	3990820	GGGTTTCAACTTTTCTAGATCCTTC	(AAT)5	(AAT)8	CTCCCGAGGTAAGTGGACAG		59.91
Ca-II-SNP659	142.48	Ca-*desi*-LG(Chr)4	4003197	GTCTCTAAAATGTTTGAGATGACTCTGTCTTCCACCGAACCGAACTATGACCGGAATACTACTTACTTATTAGGAAGTTCGGAGGTGTAGGGTAACAACG	G	A	GGTAAATGTTGGATAAGAAAAGTACTCATATACTTATTGTTTTAAGATTTTGGGTTGAAATGTTTTACCAAAATCTTTTGTGGATTATAGAACATTTGAT		NA
Ca-II-SNP660	143.69	Ca-*desi*-LG(Chr)4	4020449	TTTGTTTCCTTCTAAACTCTTGATATCTGAAACTAGCGTGATATTTTAACTAATTTGAGTTGAAATTGATAGGCATTCTTAAGAGGGGAAAATCCTTCCA	T	A	AACTCCTTTTCTATGATGAACAAAGTATAGCAATAAATGAAACCTATTGAATTTAATATAATTTTGTAGAAAACAAAATGAAATAAAATATATAAAAATG		NA
Ca-II-SNP661	144.90	Ca-*desi*-LG(Chr)4	4027639	ATTAGCTTTTGTCATTGATCACACACGCACAATATTTGACATGTAGTTTATGTGTTTACTTATTGAAGCGTTGGTAGTGTCTCTGTTTAGTCCACACGAA	T	C	GCAACAAATGCCACCCAAGAACAAATATATGCCGATACTCAACCATTGGTTAGATCTGCTTTAGATGGATATAATGCATGCATCTTTGCATATGGACAGACTGGCTCAGGGAAGACCTACACCATGAGTGGACCGGATCTGATGACTGAAGAGACATGGGGTGTCAACTACAGGGCTTTG		NA
Ca-II-SNP662	146.11	Ca-*desi*-LG(Chr)4	4030857	TTGAAATTAATCTATGCATATACATAATATTACTATAATTCATACCTGCTTCCTCTGCTTTCCTGTGAGCCAATTCATGATCGTTGATTTCCTCTGTTAC	G	A	ATACTAGTATGCCTCAGGAGAGTAGCTTACCAAACTCAATTTTCACATCTCCATCGAAGAGAATATTGAACTTGAAGGCTTCAGTTTTTAATAACAGTGA		NA
Ca-II-SNP663	150.51	Ca-*desi*-LG(Chr)4	4085353	GATGTGGAGGAATTTGATGGAACTCATCTTGTGTTCATTGGGGCTGGATCAGATCCTTATGAAGTTATAACAAATGCTGTCAAGTGAGTTTCACAATACC	T	A	TCACTTCCAAAGATTAAAACATGAAGCCTAATCATAGTAATTGAAAAAACTCAAAACTAACTTTTTAAGCAAGAACAAAGAAGAATTGAAAAGGTAAAGC		NA
Ca-II-SNP664	151.50	Ca-*desi*-LG(Chr)4	4101088	CCAACAGTGGTTTTGTGAATAATTTCTACCAAATGAAACTGCCTCTATTGCCAGCTTTTCAGCTGAACGTGTTAAGGATATGTTTTAGAACAACTTATGT	A	G	CTGGAAAATATATAGTCAATGGCCAAAATCCAAAAGCTCCTAGCACTCCTAGAACTTGATTGAAGTAAGGAAACATGATTGCAATTCCAGTTGTGGAAAT		NA
Ca-II-SNP665	153.48	Ca-*desi*-LG(Chr)4	4131111	ATATAATTTAAAAGAGTTATGTTGCATACCACCGTTAGTCGTCGAGTTCTATCTGCCACCATCGAGAAACTTATCGAAGCTTCTCCGTCCATGGAGGTTG	C	T	GGTTAAATCAGATTTATTGTAAATTTGAGAATGAAAAGAAATGGATCTAATAATTAAATAACAGAGAGGATGGTTGGTCGCAGCCGACGGCGGAGCTCTG		NA

Marker IDs	Genetic positions (cM)	Chromosomes	Physical positions (bp)	Flanking sequences/Forward primers	ICC 12968	ICC4958	Flanking sequences/Reverse primers	Annealing temperature (0C)	Amplified Product size (bp)
Ca-II-SNP666	154.47	Ca-*desi*-LG(Chr)4	4176869	CACAAGAACCTGATTCAATATCTACATGCTTATGT<br>AGACCTGATCGCGGGGAGATATTCATTCTGGAAC<br>CGAACCGGAGGGGCTGATCATTTTCTTGTTG	G	C	TTCTGACCACAATATAAAGGTTTGGACATCTTC<br>TCATTGTGATCATAATAGATTTCCAACAAATAGA<br>CAACGAGAAGAAGTCATTACCCAATCATGGCAA		NA
Ca-II-SNP667	155.46	Ca-*desi*-LG(Chr)4	4207427	GAGTAAAGAGATGTCTCATCTAGTTATTCCGTTTA<br>AAAATATTTTTTATTATTTTTGATTTTTTATTTGAGT<br>TTTAAAGCCACCAAGTGGTACAACTTGT	A	G	CTCTCTATGTGTATTTGTTTAATTTAAAAAAAAA<br>CATTTACAATAAATAAATATTAGATTAATAAGAT<br>GTGACATCTTTTTAATCTTTGAGTTGTTAAAA		NA
Ca-II-SNP668	156.45	Ca-*desi*-LG(Chr)4	4299087	GGGTACCGTCATTTTCTCCATTTTTGGAAATCAAG<br>AGGTGGGTTTCCCTTTTTCTATCTTTCATTCATGC<br>ATGTAGGAAATAATGTGTTCCATAGGTTAG	A	G	ATTTACTCAATTCTACACTAATCTATTTCAAAAT<br>CCCTAAATCCAACCAAGACTAAAATAAAAATCAT<br>TTTCTTCATTTCAAACTCTTAAACCCCAAATC		NA
Ca-II-SNP669	157.44	Ca-*desi*-LG(Chr)4	4379030	TTTAAGATTACATTCTATCAAGGTTAGATAGGTGC<br>TGGTATTGCTTTCTGGGTGGAAAGCAATAGAGGT<br>TGAAATAGATCAAGGTTGACCTAGACTAGGT	A	G	AGAGTTTATCATGGTTCACCCAACGTGGGTTAC<br>ATCTAGTCCTCACACAGTGAGATTTTCCACTAA<br>GTGTTTAAAACAAAGAGCCTTCTTGATCTTACA<br>A		NA
Ca-II-SNP670	158.43	Ca-*desi*-LG(Chr)4	4414547	TTTCTTTTTGTTTATGTATGATGATGCATTCACATG<br>GAAATTTGAAAAGATTAAAACTAATTCACTTTTAAC<br>ATTTCTTGAGAAAGTTGTCAGTGGGTTG	A	G	CATTCAAATAATATTTGACTGTATTTGAAAACAT<br>AAAATAATGTTGATTTTGTTTTGGAATCTGCAAA<br>ATATGATCAATTGGTCCACACAATGACTTCTT		NA
Ca-II-SNP671	161.40	Ca-*desi*-LG(Chr)4	4442373	TTTCTCTTTCTTCCTCTCTCTTTCAGCCTTCCTCTC<br>CCTCTCCCTCTCCCTCTTGCTTGCAAGTTACAGCC<br>TTCCTTTCTCCTTCCTCCAGAATCAAATT	T	C	ACGACGAACGGCCACAGAGGCGGTCGCAGGC<br>CCGCAGCGAGCAGAGGAGCGATCTGGACTGG<br>AGGCAGAGTGAGGCGGTCGCACGTTTTTCATG<br>ATCCGT		NA
Ca-II-SNP672	162.39	Ca-*desi*-LG(Chr)4	4466488	TTATTTATTCTGTTTTGTGCTTATATTTCAACTTAA<br>GTTTTAGATATTACATATTTGATCGGTACATTATAG<br>TGTATTTTGGCACCCTGGGTATTCACGT	C	T	ACACAACTACGAACAATGGCAGAACTTGACTAA<br>AAAGCTTTCCACCGAATTGGAGTACGGAATCAT<br>CTCCAAAGATCTCGGTCAAAGCAGGCATATGC<br>CA		NA
Ca-II-SNP673	163.38	Ca-*desi*-LG(Chr)4	4494394	GTATCAGTAAGGTACATTAGGCTTCCTATCATTTG<br>CTTGTAAAGGGTAATGTCAACATCTGCTCCTCCTT<br>CTTTCTTAGAGGGTTTCACTCTTGGTACTA	A	T	GATGCAGACAAAAGAGGGAATTTACATGTGTCA<br>ACGAAAGTTCACAAGAGACATTCTCAACATATT<br>TAGTATAGATAACATCAATGGAGTAAGCAATCA<br>C		NA

Marker IDs	Genetic positions (cM)	Chromosomes	Physical positions (bp)	Flanking sequences/Forward primers	ICC 12968	ICC4958	Flanking sequences/Reverse primers	Annealing temperature (0C)	Amplified Product size (bp)
Ca-II-SNP674	164.37	Ca-*desi*-LG(Chr)4	4507431	TATGCAGCCTTTCATTTTTAGTTCCAATTCCTAATTTTATATGATATTATTATAAATTTCAATCATTCAAACATGAGGAGAGGGCTCTTTCTTCATGGAC	A	G	AGCAAAAATATGAACACACTTTTCTTTTGTTCGACTCAATCAAAGCTACCAACTTTTTCCCCTTAATTAACTTAATCTCGTTTAGCTTCTTAATTAGCTA		NA
Ca-II-SNP675	165.36	Ca-*desi*-LG(Chr)4	4514512	TTTTTTAGGCTCTATAAATAGAAGATCAACATTGTTTTCAATGTCAACATTTTTAATGCGAATTTATGGGCCATCATTGTCAAAATGATGTTGTATTCTT	C	A	ACGTTTCTAACACAGTTCAATTCTACGACAGGATTGATTAACAAGGAGATTATGTGATCGACCAACACAAGCTAGAAATCACTTGTTGAAGTATCTAGTG		NA
Ca-II-SNP676	166.35	Ca-*desi*-LG(Chr)4	4540552	AATAAGAAAGTTTCTTCTTTATCAACTGAACAAATGTTGGTTTGTAGTGTTTATCAATGGAGCAAATATTGTTTGTGCATTTTCAATTTATACCTGTTAT	G	T	AGGGAATCATATATGGCTTGCTGTGACAATTTTAACACATAACACTACATATATCATATCAAACTTATTATAATAATTGTTTGCATATTGATACAACATA		NA
Ca-II-SNP677	167.34	Ca-*desi*-LG(Chr)4	4563041	ATAGGTTATAAGTTATAAGCGTGTTTTTTTTAACATTCCAAATAGATGCATTCAAGTAACTTATGAGCTTATCATTTTTCTTCCAATTTTACTTTTATTA	T	A	AGTAAAAGATCAAATTGAGAAAACAAAATATAAATGACTAAAACTTTAACTGAAATTAAGTTAAGGGATCACAGATGCTATTTATCCATTTTTTTATTAA		NA
Ca-II-SNP678	168.33	Ca-*desi*-LG(Chr)4	4580896	AAGAGCTCTGGTTCGACTTGGTGGGGTAGTGAGAGTAGATCCTGGTGTATTTGGTCCTTGTATCAAAAGTTTTTCTAGCAGTGTAGTTAGGAGTCTGGTC	A	C	CCTAGTCGATGTGGGATTAACCATCACATTTAAACCCAATAGTCTCCCCCTCAAATGTGAGTCACCCATATTTTGTACTTGTACCACCATTATTACCAAA		NA
Ca-II-SNP679	169.32	Ca-*desi*-LG(Chr)4	4590227	CAAAAAAAAAATCTTAATATATGAACGAGTCATGAAGAGGTTGTCTTGTTATAATCTCTGTCACTTGGTCAAATGCTTTAGCTCAGTTTTTTTCTTTCTC	T	C	AGTAGAAAGTAGAGAAGAGAGATTATGCACCTGATGCAACTAGAAATGAAGAACCAAAGCAATATTCTTAGCTGGTAAAAATTGATTGAAATAGAAATAT		NA
Ca-II-SNP680	170.31	Ca-*desi*-LG(Chr)4	4635664	GCTAGTGCGGGGGCAGTATTGGAGTTGCGGGTGCGGGGTTGGTAATGCTTAAAACCGTCCATGCTCCGCCTCGTTGCCATCCCTAACTTGAATGTGTGTT	T	C	TTTTTTGATATTCTTCAGGAAATTTATTTGATATTTGATTGATGAATATATATGGTAAGCCAATTTCAACAAAGGCTCCACTGAGTAACAAGAGAGTACT		NA
Ca-II-SNP681	171.30	Ca-*desi*-LG(Chr)4	4654094	TTCCTTGTGCACGACCTAAATAGTTTGCCAAGTACTTTCGCTTAGACAGAGGCAGAGGCTGGACCATGGTAGACCCAGCCCTGGTCATACCATCTTCGAC	G	A	CTTAAAGTTTGTTCTTTGGCAAACCAATGTAGGGGGATCGCACTGATAAGAGAGATACTAGTGCCTTTAATACATGGAAAGATATCATCATTCCAGGTAA		NA

Marker IDs	Genetic positions (cM)	Chromosomes	Physical positions (bp)	Flanking sequences/Forward primers	ICC 12968	ICC4958	Flanking sequences/Reverse primers	Annealing temperature (0C)	Amplified Product size (bp)
Ca-II-SNP682	172.29	Ca-*desi*-LG(Chr)4	4663824	GACTTGTTGATTTTTGAGACAACAATCTGGTTTTGGTGCAAGGATAATTGTTTGAATTTTGTTTGTATTTGTATTTGTTTTGATAATTGCTCATTATATG	G	A	AAAATCGAACTTTGTCAAACTTTGCCAAACACAATTCAACCATCCCCCTTCTTGTGATTGCACATTCAATCCACAACTATTATGATATTGATTTTAGTAC		NA
Ca-II-SNP683	173.28	Ca-*desi*-LG(Chr)4	4688141	AAACGCAGGTATATGTTGTTGTATAGTGCATACCCATGCTACCTTTCAGGTATTTGCTGTACGTTTTGTCCCCGACCAGGGTCAGTGTCTTTGTCGTATT	G	C	ATTTGTGTAATTGTTGAGTTAAATGTATAAAAGAGCTCTGTGAATCATGTAAGAAATAAATGATGGTGAATAAATTATGACAGTAGCAATGAATGCTCTT		NA
Ca-II-SNP684	174.27	Ca-*desi*-LG(Chr)4	4698858	GAACTCTATTTCCTCTCTTCTAATTTTTTTAGGAAAGAACTAACCTCAAACCAAATTTTGGTTCTCAATTTATATGCTACAATGATGAATCGTTGCGTCT	A	T	CACCAACTTTGCACTTCACCAAAAAATAGAAGTCTCATCTTCACTTGTCACTTCATAATTTAAACAAAACAATACAAAAATCGAAATTCAATTTATCTCA		NA
Ca-II-SNP685	175.26	Ca-*desi*-LG(Chr)4	4702919	GGTTATGATATTTTATATTTTGTTTGGTGCTTAGCTGTGAAAATAATGAATGTTGTGGGAGATATATGTAGGAAATTGGATTAGTGATTACCCTTTTTGT	G	T	TAAAATCTTACAAATTTCCACCCTCTCACAACCAATCATATCAATATCAATATCAATAAATTAATAAAAAACATAAAATGGCTTTTCTGTAAATAATTCA		NA
Ca-II-SNP686	176.25	Ca-*desi*-LG(Chr)4	4710255	CACACTACTACTTTTAACTACATTCCCTCATGGTTATGGTCCCTTCAACTATTAATTCTTAGATCGGTTTTCACTCGGAGTGAGTATTGTAAATTCTAAA	C	A	TGAACTTAAATATATCCAATCATTGCCAAAATATTGAAAACTAATTAAAAAACTGTACAATTACAATATATCATTTTTACAGAGAAGAATATAATTCATA		NA
Ca-II-SNP687	177.24	Ca-*desi*-LG(Chr)4	4719601	TAGTGGAAATCTAATGGTTGTGAGGACTGGACTAGCCCAAATTGGGTGAACCAGTATACCTTTTTTGTGTCATTTTCCTATCCTTATCTTTAATTATTTG	C	G	TAATTAGTAAGCTCTTAATTGATGAGAACATTATGTACAATTTAAATGAAATCAAGAGGTTGTAAATAAAAACAATGATAATGAAGTGTGATATGTGTGT		NA
Ca-II-SNP688	180.21	Ca-*desi*-LG(Chr)4	4732975	ATTAGCTTTTGTCATTGATCACACACGCACAATATTTGACATGTAGTTTATGTGTTTACTTATTGAAGCGTTGGTAGTGTCTCTGTTTAGTCCACACGAA	A	T	GAAAGAAACACTATATATGATACATTGTGTCTCGTCTTAACCTAACCATAGAATCACCTTAAGTGATCGTCAGCTAACACTGACACATTCCGATACAACC		NA
Ca-II-SNP689	181.20	Ca-*desi*-LG(Chr)4	4778314	CCTTATCTCCTTTTTATTTTTCACCTGCACAACACTAATTATTTTGTGAAGTTTTTATTGTTACATTCTAACAAAGAACATTATGTCTTTATTAAATCAA	A	G	GTAATAGAAATGACAATGTTGATTACAAAAAATGGGGGTTTGAATTGTGATTCCCTTTTTAAAATTAATTTATGTATTAATTTGTTAATTTAAAATTGAT		NA

Marker IDs	Genetic positions (cM)	Chromosomes	Physical positions (bp)	Flanking sequences/Forward primers	ICC 12968	ICC4958	Flanking sequences/Reverse primers	Annealing temperature (0C)	Amplified Product size (bp)
Ca-II-SNP690	182.19	Ca-*desi*-LG(Chr)4	4795454	AATAATACTTTAAGTTTGTATCTTACTTAGTTTAAATATTTGCTTTGTAAGAGAGTGGTTGTACTGGGGTGTTTGGTGCTATTTTTCCCAATAGTATACT	G	T	TGGTTATTCCTTCCTCCAGCTGATTTCATGTAAATATCGTGTATGCTAAAAGGATTGAAGGAACATCAAAGTTTTTATTAATATCCCTTTGGGTTTCAAG		NA
Ca-II-SNP691	183.24	Ca-*desi*-LG(Chr)4	4809998	TTATCATAATTTTAAGCATCAATTTATGCTAGTTATTTGACTTTTGAGGGAAGGCACCTTGAATTTAGGAAGAAATTCCCCTGCCTACCCATCTTTATCC	G	T	TCCATTTGTGAACCAACAAAACGCTTAAGCCCACACAATAAATAACATAATAGAATATGTTATGATATGACATCCAGCAATCAATCCATTATGAAGGCTG		NA
Ca-II-SNP692	186.39	Ca-*desi*-LG(Chr)4	4821489	TTAGATAGTCAATCGCGATGGTTCACATCGCAATCGAGGTGGAGTGTGGCGATAAGGGCATGGCCACGACGATGCAATGCAGCGCGGGGAGGAGACAGGT	C	A	AGGGTAAAATTGGTCTTTAAAAAAAAAAGAAATCACATTTTTAGAGTTTTGAGAACCCAGGACCCGAAACACGATATGCGAACGCGTCCAAATTGCACGC		NA
Ca-II-SNP693	187.44	Ca-*desi*-LG(Chr)4	4831174	CTCGTCGCGATCTCTATTTCGCGTCCCTTTCTATCTGTTGAGGTTTGGATTGATAGAAAGAATACGGTGTTATTCTTGACGAAAGTAGAAAGAAGACTGG	G	A	GATAATTTTTTTATAAAGAGTAAATCATTAATAAAATAAAATAATCATTTGTTTTTAAAGTAATTTGAGACGAAACGGATAACTTGAGAGGTAACTTAAA		NA
Ca-II-SNP694	188.49	Ca-*desi*-LG(Chr)4	4847066	CTCTTCCAATCTTTTGTAGAACTTCCAAGCTTGATACCAAAAGAGTACGTACCTCTCTTTCTTTTTTTGAAATTTTGTTAGATATATAGGTGTTTGTTTT	C	A	TTCCACTTGAGATCAACAAATTGCACTATCTACTAGAACATGTTCTTATGATTTAAGTAGTAAATAAACAAATAAAATTTAAGTTCTCACTTTCACCTTA		NA
Ca-II-SNP695	189.54	Ca-*desi*-LG(Chr)4	4864461	GATGCACTTACCGTTATGAGTGTTGTTATTAATGTTCTTACGCAGCGGTCTTTGCTCCAAAAACATTACACATTCACAAATTAATTTACTACTCATTTAG	T	C	GAGAATTGATATCTTTTAAAAATATAGAGAAATGACGTGTCTTAAAACCTTAAGCCAACACCAAACCTTGAATATGAATTAGAGATAGATGAGTTTTGAC		NA
Ca-II-SNP696	190.59	Ca-*desi*-LG(Chr)4	4880696	TACAACTAGACTTATAGAGTGTGAGTTTGTAGGACTAGACATATGGGAGTGGTCTAATGGATCTAAGAGCATCTCCAACGGTTGCAAAATGAGTTCGTCC	G	A	ATGAAAGTACCGTTCCTTCATGAGAGAACCGTGATGACAGCTTGACACATCTCCAATAGTGAACTCGCCAAAAACTGAATTTTACGTAACTACACGCTGG		NA
Ca-II-SNP697	194.79	Ca-*desi*-LG(Chr)4	4971795	TTTTGTTGACTATTTTCTCTTATATTTAAAACTGAAGAATATTTATTTTAGCTGAGTTCGTTCCCCAATTCGACCTTTATCATAAATATCACTAGTTACT	G	T	TGTATATTTTTTATATCAACAAGATTGTGTATAAATGTGTGTATCTAGCATTGTTTCTACCAAAAACCAGTGTTTTTATTTTTTATTATGTATTGAATGA		NA

Marker IDs	Genetic positions (cM)	Chromosomes	Physical positions (bp)	Flanking sequences/Forward primers	ICC 12968	ICC4958	Flanking sequences/Reverse primers	Annealing temperature (0C)	Amplified Product size (bp)
Ca-II-SNP698	195.84	Ca-*desi*-LG(Chr)4	4981638	TTTTGTTAAAGAGGTGGAGAATGATTGGTCAATATGCTGTGATGAACATTCTTGAAATTGGAGATCATTCTCTGTTTTTATGCAGAATGTTCTCATTGCT	A	G	TAATTAAGAAACTTAATTATGATTCTAAAATGTTAAGTTAATTTAACTTGTGTTTTTGAGTGAATTTAATAAAGAACAAAATCAAGCAAAGCAGAAAAGA		NA
Ca-II-SNP699	196.89	Ca-*desi*-LG(Chr)4	5035097	AATATACCTATTGTTCTTTAAGTTTAGTCTAATTCTTCCTTAGGTCATTTAAGTTTATTTGTCTTCGAGTTGATTCTTTAAGTTACATGCATAACCTTAA	A	G	AGATTTAGTCGAAACATCGCAATAAATTGATTGTCCATTACTTTTCAAATGAAACGAGTTAATGACATTGATTATATGGATATATTTTTATTACTATGTT		NA
Ca-II-SNP700	197.94	Ca-*desi*-LG(Chr)4	5089894	AATTTATGAATGTCTGTGCAGGGACTTGATTTTATCAAAGATGATGAAAATGTGAACTCCCAACTACTTATGTGTTGGAGAGACCATTTCTTATTTTGTG	T	C	ACAGAACTCTTTTATCATTTCTACACATGTGTCTGCAGTAGCATATAAATAATGTCCTTTAATTTCACTTTTTTCAACCTGTGATTAATAAATTGCTTCT		NA
Ca-II-SNP701	198.99	Ca-*desi*-LG(Chr)4	5111270	CCCATTGATGCACCTTATGCCATCCGTAATGTATACAACCGCCTCATCAAATGAACTTCATTTGAGGCTCACGGTTTTTTGTGCATGCTAGAAAGAGTTG	G	A	ATAGATTGATGATGGTATGTATCAGAAAAACTTGTATTAACGTTATCTCATAATGTTGCTTTTAACATTGCAAAGAAAAAAAACCATAATAGGTCTTATG		NA
Ca-II-SNP702	200.04	Ca-*desi*-LG(Chr)4	5128637	GTGTGGCGAAGACTTGACTTGTAGGGTCAAAGTATTTGTTTGCTTAAAAAAGTATGTAGGTGTTAGGTTGTTTTGAGAAGACTGACTTATAGGGTTAGGT	G	C	TCTAACATCTTTGTCATGGTTTACCACCAACTTTGTAGCTACGACTAGTCCACTCTCTCGGAAGAATTTAATCCATTAATTCAAAACTTGAATTACACAT		NA
Ca-II-SNP703	205.29	Ca-*desi*-LG(Chr)4	5144601	TCTAAATGTTTCCCTTTCTCTATATTTGGACATTCTGATTTGATAAGTCCAAATTTGTTGCATCCATAGTAGATGATGCTTTTCTTGTCGTGTTTATCAT	C	T	AGAGGAATTGGCTTTCATATGTTAAAAAATTTAGAAGATGTGGCACAACAGAAAAAATAAATTTCCCCAAAGATATTCCAAGCAATCCAATAGAGGAAAT		NA
Ca-II-SNP704	206.34	Ca-*desi*-LG(Chr)4	5164229	GAAGGGAGCGGAGCAACCTCTTGGTCAGTTTTAGCTCTCCAAAAGTGGGAAGTTTTAGGTGAGAATATTTGTTTAAAATTTGAACTTAAATATCCTTTTA	T	G	TGGTTCGACGAGGGGAGGGGAGGGAAGAGATTTTAATAGATGAAAGGTATGAACTATGAATTTACTTTTTCCACCCTTGTAATATTTACTATCATATTAA		NA
Ca-II-SNP705	207.39	Ca-*desi*-LG(Chr)4	5228872	ATTGCAGAAAGTGATGTTGCCTCTGTTTTTGCAGAATTTCAAGCTTAAGTGAGATTGTATATTGTTGGTTACTTAGCACTTGAATTTTTTGCCTTCATAC	G	T	TTCTAAAATAATGTTTTAGTCAAAAAAAGCATGGCCTCTCCAACAGCTATTTCGTACTTATCCAGTGCATTCTCAGCTTATAAATAGAGGGATTCAATCT		NA

NA

Marker IDs	Genetic positions (cM)	Chromosomes	Physical positions (bp)	Flanking sequences/Forward primers	ICC 12968	ICC4958	Flanking sequences/Reverse primers	Annealing temperature (0C)	Amplified Product size (bp)	
Ca-II-SNP706	208.44	Ca-*desi*-LG(Chr)4	5405499	ATGTTTCTTTGTTTTATCATGTTTGTCTTCATATCATCAAGTACAACTTTCTCTTCCTGAGACAACTGACCTAAGAAAGTATGATCAATAAGTTATTTGG	T	C	TAGAAAATGTGAATGTCCATTTAGATTGAGAGGAACACCATCAAGTGTTGGTGATAGATGGTATCTACATGTAATATGTGGTGTCCATAACCACAAATTG		NA	NA
Ca-II-SNP707	209.49	Ca-*desi*-LG(Chr)4	5451471	TGGATTATGGTTTCAAAGGTGCTGAAATTGCAATGGCTTCTTATTGTTCTGAGTTGCAATATCTTGCTAACCCTGTCACTACACATGTTCAAAGTGCTGA	A	G	CAATCAAGAAAGTTGAAGACATCAACTTTAAGATCTCAATAGCCTCATTTGTTTTTCTAGATGAAATCAAACCCAAAGAGTTAACATCTTGGTTATGTTG		NA	NA
Ca-II-SNP708	210.54	Ca-*desi*-LG(Chr)4	5483523	ACTTTATTCTCTAAAGTTAGTTGCTCATTTTAGAGGAGTCAAATCCTAAATTATTATACCAACATTTTAGGCTTGTTTGAATTGACTTATTTGAACTTAT	C	T	ACTTATGAAAATAAACTGAAAATAGCTTATGGTCATTTCATTAGTTGTTTAGCTGTTTCATAAACTCTCCTGAATAGTCTCACAAGTGTTTATGTTAATA		NA	NA
Ca-II-SNP709	211.59	Ca-*desi*-LG(Chr)4	5528932	GGTCTGGAGGATTCACACGATCAGGTACAGTGATTCCAATGCCAGCAAAGTATTCTTCTACTTTTTTAACAGGGCCGTGATATGCCGTAAGGCCACCTTT	T	G	TGTTCTTCAAACATTCCAACATAATTTAAAATCTTACTTTTGATTTATTGTGATACAGCTATACTTTGTTCAGAATGTTTGATGATATAATATTTCTAGC		NA	NA
Ca-II-SNP710	212.64	Ca-*desi*-LG(Chr)4	5544750	AATCAATTTGCTGACACCATATGGTTACTTAATGCTAACTGGTTTTTACTTTTTGAATTAGTGAAGTGTTTGGAAAGTCTTCATCATATATCACCTTCAA	T	C	AAACACTTCATCGCAACCTTACCAGAATCACACTGCATTTTCACAAAATTGAAATAAAACTAAGAAGCATAAAGTCATTGAAAATCAACACAGCATCTAA		NA	NA
Ca-II-SNP711	213.69	Ca-*desi*-LG(Chr)4	5611371	GGACACATGGAGCAGAACCACGAGCTTTGTCAAAAATTTCCCTCACATTAGCCTCACTTTCTCCAAACCACATTGTGAGTAGCTCAGGACCTTTGATACT	A	G	GAATGTCACCTTCAAAGGGGGTTCTGTTCTATGGTCCTCCTGGTTGTGGTAAAACCCTTTTGGCCAAAGCTATTGCCAATGAATGTCAAGCAAACTTCAT		NA	NA
Ca-II-SNP712	214.74	Ca-*desi*-LG(Chr)4	5678633	TGCGGCGGTGGAGGCTTCGTCGGCAGACAATGTGCCGTAGCGTTTAGAAAGGACTGAGGGAGTGGAAAATGTTTGGATCAAGCGGTTTATGACGGCATCC	A	G	CGGCGATGGCGCTGCGTGATGTTTTGTTGAGGGGGATGAAGGTTAGGGCGAAGGAAGCGGTGGAAATGGGGATTGTGAACACGGCGCACGATAGTGAGGATGGGACAGTAGAGGCTGCAATGCGCATGGGGGAGGAGTTAGCAAGGAAGAAGTGGGTGGGTGACGTTTATGGTGAAATCAGGAAGAGATTGTATCCTCAAGTTTGTGGTGTTTTGGGTTTAAC[illegible]		NA	NA
Ca-II-SNP713	215.79	Ca-*desi*-LG(Chr)4	5726782	TTTTGAAACGCTTATCGACAAACTGAGTTTCATTTGTAGCTTCATTTTGGATGATGCACTCATCTTTATTAAAAACTATCTTGTATCCTTTGTCACAAAG	C	T	AAATTTTAGGCATTGGAAAAGTCGGTACAACACCATCCCCCTCGATTGAAGATGTCTTATATGTCGAAGGTCTAAAACATAATCTCATAAGCATCAGCCA		NA	141

Marker IDs	Genetic positions (cM)	Chromosomes	Physical positions (bp)	Flanking sequences/Forward primers	ICC 12968	ICC4958	Flanking sequences/Reverse primers	Annealing temperature (0C)	Amplified Product size (bp)	
Ca-II-SNP714	216.84	Ca-*desi*-LG(Chr)4	5767017	GTTGACCTGTTTCATTACTCCTTGTGTAATAGTCATTTGGTGCCTGCTGAAAACGACCAGTGTTTTGAGTAAGGTTCCCTTTCTGTCCCTGATACTCTAA	G	A	GCGTTGGAACGCATCCTAATGCTTTTTATGAGAAACATAATTGGAGACTAGATTCTGGAGAAAAGGTTAAGTACCCCAATGCATATAGTTTACCTAGGCC		NA	NA
Ca-II-SNP715	217.89	Ca-*desi*-LG(Chr)4	5817107	CACCTGAGGATACACCATCACCACTTATTCCAGAGCCAACCTCTCCATTGCGTAACTCTCCTGATACTACTCCTTCTCATGCCTCACTTGTCCCTACAAC	C	A	CATCTTTCATATGTTGAGGAGAAGTTGATTTTGAGGCATCCTCAGGGATAGGATCGGCTTCACGAAGGGGAGTCAGTTTGTGGATTGGTGTAGACTGAGT		NA	NA
Ca-II-SNP716	218.94	Ca-*desi*-LG(Chr)4	5907659	GGTCGGTTACGGTTGCCGATGGATCCAATTTCTTCATCCGAACCCGACCACGGTAGACCGTGTATATCCATCGACGTTCACCCGCGAGATCCGACAACCC	G	A	GTGTTAGTCATAATCAAAATTTTTAGGGGTGGACAGATTTTCCACACCCGCCCATAATCGTCCAACCCATACGACCCACAAGCAAACGGGTGGTTTGGAT		NA	NA
Ca-II-SNP717	219.99	Ca-*desi*-LG(Chr)4	5934551	ATAAACCAGAATGTTCTTCTGATCAATCTGTCATCAATCTGGAGATCAATATGCATGTTTGTTTTTGCAATTATGCCTTCAAATCTTGAATTTTAATTGC	T	C	TTATTCAATTAAAGAACAATTGACAAAATGGATCTTTTTAGAATAGCAACATGTAGTAAATCAGCTGGCATTTCGCACATGGTCTTCCCAACATTTAATG		NA	NA
Ca-II-SNP718	221.04	Ca-*desi*-LG(Chr)4	5971039	CGTAGTCAATCAGCAGACTCACACAAAATCAAAGTACGGCTCCTTCTTGATGAGGCCTCTCACCCAAGAAGATCGCCACCAGTTTCTAGTTGGATTTCTA	T	G	TAACCAGATTCGTGAGAAGACACATTGTTCAAGATTGAAGACTTCTTTTACTACTTCTTTTATTCTGTTTGAAATAAATACTTTGTAGAGAAAACGACTG		NA	NA
Ca-II-SNP719	222.09	Ca-*desi*-LG(Chr)4	6005969	GTATTCAAACTTGGAATGGAGAACATCTATTCACCAGCTTGAGGGGGAGTGTCAAAATCTGTTTCTGTTTAATATCCCTCAAGTATTGGGATTCAGTTGA	C	A	CCTATTAGTACTGATATATAACTACATCCCTATTAGTACTCATATATAACTAAATCCCTATTAGTACTCATATATAACTAAATCCCTATTAGTACTCCTA		NA	NA
Ca-II-SSR198	223.14	Ca-*desi*-LG(Chr)4	6006092	ATCAGTACTAATAGGATTTGGTTGTTT	(TA)7	(TA)6	CAACCCCTTAAACCTACACATTG		57.11	NA
Ca-II-SNP720	225.24	Ca-*desi*-LG(Chr)4	6031929	TTATCTTTAGTTAGAGGCAAATGATCATTGTAACGTTCTTTTCCTTTGTAATCAGATGATCATTTCTTGACTTATATTTATTTTGTCTTTAATGCTTTGA	C	T	TTACCTAATAAACAAACAATTATGTTTACTAATTATTACAATAAGTCATGATATAGAAATAAGAAGCGTTGCAACAAGCATATGATAATCTATAGTTCGA		NA	NA

Marker IDs	Genetic positions (cM)	Chromosomes	Physical positions (bp)	Flanking sequences/Forward primers	ICC 12968	ICC4958	Flanking sequences/Reverse primers	Annealing temperature (0C)	Amplified Product size (bp)	
Ca-II-SNP721	227.34	Ca-*desi*-LG(Chr)4	6062333	TTGTTTGAGATTGGTTTGTTTGTTTTTAAAAACAATTTCTAGATTGATTTGTTTGCATTTGAAACTAGTTTACAGATTGATTTGTTTGCATTTAAAACTA	T	G	AAACCAATTATCAACAAACCAATCTGAAAACTAGTTTTAAATGCAAACAAATCAACTTCTTTACTTCAAACAAACCAATCTGGAAACAAACCAATTGTAA		NA	NA
Ca-II-SNP722	229.44	Ca-*desi*-LG(Chr)4	6098301	GCAATTTGTGGATCTCAGGTTGTGTTGAGAAGACTTGACTTGTAAGGTTAATTGAGTAGTTTGCATCTAACAATTTGTAGGTTTCAGGTTGTGTAGAGAA	G	A	GAACACAACCACCTTGACTCTACTAGCCAAGTCTTCTCCACACAATCTAACAGCCCCAGATTGTTGAAGGCATACAACACCTTGACCCTACAAGTCAAGT		NA	NA
Ca-II-SNP723	232.79	Ca-*desi*-LG(Chr)4	6134862	TTTCAATATACGTGACTTTAATTTGTTTCCTCAATTTGTTTGAACCTAAATTCGAAGCCCTTTTTTAATTTCATTGATTCTAAATTCGGTTAATATAGTC	T	C	TTTTGTTGTTAAAAGATTGATCTATTGTCGCGCATAAGTCAAATACAAGCAATAAAACGTTGCATAGGGGCAACAACTCGAGTCGTTTCGCAAGGACTTC		NA	NA
Ca-II-SNP724	233.94	Ca-*desi*-LG(Chr)4	6152405	GACTTAAATGTACCTCCGCCTCATTTGTTAAATCTGGTGGCACGTAACCGACTTAAATCTGCCGTATTAAAAGCCCAATTTTCTACTAGTGTGTTAGTGA	T	G	TATTTATACAATTGTTGCTAAGTTTTTGGCTAACATGTTGAAAAACATTATGCCCTATGTAATGGGTTCTTCTCATAGTGATTTTGTTGCTATTAGAATC		NA	NA
Ca-II-SNP725	235.09	Ca-*desi*-LG(Chr)4	6204689	TGATAATGAGTAACTAAGCTCTCTTCTAAGGTCTTTGGTGAATCTCGTGATTAAGATAACTTTTTACTATGTCTTAGTTTTAAATTCTAGTTTTATTTTA	G	T	GTCATCCCAAACATTTAAATAAAACAATAGAATAATGTAATTGAGATAAACAATCAAAATCTAAAATAAAACTATAATTTAAAACTAAGACAACCAAAAT		NA	NA
Ca-II-SNP726	236.24	Ca-*desi*-LG(Chr)4	6230253	CTGGGCTTTGCCCTCAGATGAGAGTCAGGACGATCCTACCGGTTCGTACCCTACGGACGGGAATGGAGATAGGAACGCTTGACTGCAGCTACGTTAGGAG	G	C	AATACAAAAGAAAGACATCTGATCCCTCTATGCCAACCTAATCTGATCATTTTACAACACAACTAAACACCCTGAGTGATCTCCACGCGCCCCGTGAGAT		NA	NA
Ca-II-SNP727	237.39	Ca-*desi*-LG(Chr)4	6316674	TTGAATTATAATTTTATTATTTGCATTTTAATTTTATTATTTATATTTTGACTCTTTCTCGCTCTGTAGGTCATAACTTGAGCTCTGGATATCGGATTGG	T	C	AAATTTGCAATTTCGGCCTAGTAAAGTTTGGAATTGGTCTTTCCTTCCAACACAAAGGTTATAGCTCAGAATTTTATCTTTCTAACGCCTACTCATATGT		NA	NA
Ca-II-SNP728	238.54	Ca-*desi*-LG(Chr)4	6331712	ATGTTATAATAGGGTGAAGTTTGTTTTTCTTAGTGATTGGTTTATAAACATCAATTATTTATGTTCTTTTTTTTAGTATTTGTGTTGCAAAACAATTTTA	G	A	AAATGTAGTTTGGTTTTTTTTTTGTTTTTTTTTTTTTTTGTCTGTTTATGCAAAAATAAACTCATATGTTAAGCTTTCATTTTTCTTAGTTGTTTGTTC		NA	NA

Marker IDs	Genetic positions (cM)	Chromosomes	Physical positions (bp)	Flanking sequences/Forward primers	ICC 12968	ICC4958	Flanking sequences/Reverse primers	Annealing temperature (0C)	Amplified Product size (bp)	
Ca-II-SNP729	239.69	Ca-*desi*-LG(Chr)4	6341251	TTCTAACCCGAAAATTTCAAACATCTGGTAGTTAATTTTATTTTATTTTTATAATTTTTTTTTAATGTTTTTGTTAATTTATTTTTTTTGAAATAGAGAT	G	C	CGACCTCTCTTGCCCATGATATGACAGCTTCTCGTGAATCAAAAATTTGATCAGTTGTGAATGCTTCAGTTAAATCTATAAATACTGGTTGTGTCTTTTC		NA	NA
Ca-II-SNP730	240.84	Ca-*desi*-LG(Chr)4	6354569	AGACCAAACCTCATCTTGGAGGTGGAAAGATCTCTGGTGATAAACTAACCAGCACCTATGATCTTGTTGAGCAAATGCAATACCTTTATGTGAGGGTTGT	T	G	TCTCGAAATGCCGGGTAGTTCCTTTATAGTTCCCGAGCTTAACTTCGGCATAAGGATCGCAACTTCCGGTGACATCCTTTGAAGGTAATTCCTTAGCCTT		NA	NA
Ca-II-SSR199	241.99	Ca-*desi*-LG(Chr)4	6361616	TTGAGATCAGAGGGTGCAAA	(TAA)9	(TAA)7	CGGAGACCAAAATAAATAAAAGG		59.37	NA
Ca-II-SSR200	244.29	Ca-*desi*-LG(Chr)4	6388052	TAAAATGGGTGTGCGAGACA	(AT)8	(AT)9	CCCATGGATTATATACGTGGAA		60.11	NA
Ca-II-SNP731	245.44	Ca-*desi*-LG(Chr)4	6455248	TTTTCATTTGATTGATGATGTCTCTTAAACTTTTCAAATCAGGTTGCTTGAACTTTAAAAGTAAAGTTTTTGTTTTTTCCAATTCCATTGTCCACTTTAA	T	C	TTCATATCTCATGACATTGTTTCTTTTGAAAAATGAAATGGCATTTTGCATCAATTTTCAAGATTGTTGGGGAATCATAAGGACCTTAGTCACATGTTAA		NA	NA
Ca-II-SNP732	248.89	Ca-*desi*-LG(Chr)4	6499244	GTATTCTGCCTCAGCGCTGGATCTTGCAACAACATTTTGTTTCTTACTTTTCCAAGATATTAAATTTCCTCCAACAAGTACACAATACCCGGAAGTTGAT	T	C	AAAAGTGCACCTGGAAAAGGTCTCATTTATGAAGATAGAGGACATACTCAAATAATTGGCTACTCAGATGCAGATTGGGCAGGCTCACCCATAGATAGAC		NA	NA
Ca-II-SNP733	251.19	Ca-*desi*-LG(Chr)4	6547630	TAATTTATCACTTTTGGAGATTAATCCTGCTATTTATTCTTTATGCATTGCTTTGACTTTGTTGATTCTTTTGACGAATCTGTCTAAAAAAATCATGAGT	A	G	AGAACAAAGCTTTCTTGAATTCTAGATCATTCTAGGCTACGAAAATGCATCAAGCTACATGGCAAATCACCAGTATTTATTACAAAGGAACAAAATTGTA		NA	NA
Ca-II-SNP734	252.34	Ca-*desi*-LG(Chr)4	6576650	CGTGATAGGTGGCACCTCGGTAAACAGTACGGACCCGTGATAGGTAGTACGTTTACGACTTACGTTCCTGCGGGAATTGCGTTTGTCCGTGAGAGACTGC	G	A	TACCAAAACACACATAAGTAAACGAGACATTAAGAGATTCCCCATTCTTAATTCTCATCTAACTTAAACCAGTGCGTAGTCTCTCACGGACAAACCCCTG		NA	NA

Marker IDs	Genetic positions (cM)	Chromosomes	Physical positions (bp)	Flanking sequences/Forward primers	ICC 12968	ICC4958	Flanking sequences/Reverse primers	Annealing temperature (0C)	Amplified Product size (bp)	
Ca-II-SNP735	253.49	Ca-*desi*-LG(Chr)4	6652484	ACTGTGAAAAATCCAGTATGAAAACTGGTGGCGATCTTCGTTGATGTAAAGATCATCAAGAAGGAGCTGGACTTGATCTTGTGTGAGTCTGACGAGTGAC	A	T	AATAGAAAGAATTGAGACTTGTTTACAGATTGTCCAGTTTCCTCGAAACCTATCTATCTAACTCAATCTCTTTCCTATTTCTTAACCCCTTGATCCTTGC		NA	NA
Ca-II-SNP736	254.64	Ca-*desi*-LG(Chr)4	6685518	TAAACCATTTTCTTCTCCCTAAATTTTCTAACGAAATTATGCATATGTGTGAGAATATCATGTAATTGTTCTTAATCATTTCCCTCTCTTGTGAGCATAA	A	G	GTGCGGCAAATGAGAAGAGGTGAGGAGTGAAAAGAAAGGTACAAATGAAAGGGAAAAGAAAAGATTGATCTAACTCTACTCATTATATTGTTTTAATTAA		NA	157
Ca-II-SNP737	255.79	Ca-*desi*-LG(Chr)4	6750199	CAAACAAGTCCATACGTATCAATTGAAGTGATCTTTAGGTTGATACAACATTCTTGGGTTTGAAGGAAACCTTAGTTTGCTTACTGATCATAGCATTTGG	G	A	ATAATTCAAATTCGACCCAAAGTTTAGAAACAAGCTAAAAATATTATTCTAATCTATAAAGAGCTTTTAAAATACCAAACTAAAAAGCTAGAAACATGTG		NA	NA
Ca-II-SNP738	259.24	Ca-*desi*-LG(Chr)4	6783507	ACCTTGTTGTTCGACGGGGCCTCAAACATAATGGGGCATGGGATTGGGGTTGTTTTAATATCTCCAAAAAAAAGGTTCATACCTATCACAGCGCGATTGT	A	G	ATCTCCATATACTTCTAGAACTTTTGCTTTCGATTCCAAAGCCGCCAAAACTCCCATGGCACAAGCTTCATATTCTGCCATATTATTGGTACAATCAAAA		NA	NA
Ca-II-SNP739	260.39	Ca-*desi*-LG(Chr)4	6805570	TTGGATTTTATTTGATATGGTTTTCGATTTTTCACTTCAATTTTAGCAATAGGGACATTGTTGGAATTTTCAGAATTGTTATTTTGCATTGCATTGTTAT	A	G	ACTAGCAGCTCTATTAAGTCTACGACGTAAATAAAATTTCCTATCTATTTCAGGTTCAAAGGAATGAAGATTCACAGAACTAGCCTTAGTCATGCATCAT		NA	NA
Ca-II-SNP740	261.54	Ca-*desi*-LG(Chr)4	6813661	GGTTGAGATGCTTGCTAGTAGAAATCCCTTTGTGGGAAAAACCTCTGCCAGCTGTGTTGATCCACTGCGATAGTACCGCAACTATTGCAAAAATTGAGAA	T	C	TAATGCGCACATGATCCACTATAACAGCTCCTTCAGAAAGTAATTCTCTAACAGTGTTGTGCTTACGACGTATTTGTCATCTCTTACCGTTGTAATAATG		NA	NA
Ca-II-SNP741	262.69	Ca-*desi*-LG(Chr)4	6852383	TCGAGGACTTAATCCACTATTTTAGTAATAGCATACAAGTATTGTTTACTCTACCTCTTCAGGCTTTTAGTATTCTTCTCTAGCCTCTCTAGATTGTCAC	T	C	TTATCCTGATAGTGGCAATAATTCTTTTACCCTTAAGAGGTACAACAACACTTATGAACCTCAACAACATGTTGTCAGAATGTTGAATAAAGGTTCTAAC		NA	NA
Ca-II-SNP742	263.84	Ca-*desi*-LG(Chr)4	6886332	GGCAAACTGGCCAGTACTATTTTCTATCATATCTGAACTTTTTGGTCTTAAATATTACTCAACATTGTACAATGTTGGGTTAATAGCAAATCCTATTGGG	T	A	AACTCCTCCCCTGGATTCCTCTTTATCCCTAATGTTTCCATTTGCCTTATGGCTTCCTTATCATAAAGATGACCAGCAACCCTCACATTTAACAAGTATG		NA	NA

Marker IDs	Genetic positions (cM)	Chromosomes	Physical positions (bp)	Flanking sequences/Forward primers	ICC 12968	ICC4958	Flanking sequences/Reverse primers	Annealing temperature (0C)	Amplified Product size (bp)	
Ca-II-SNP743	264.99	Ca-*desi*-LG(Chr)4	7022171	TAAAAGTTGAACTTTTAGTTCCCTCATTTGTAAAAG TTACTTTTTAGTTTCTCTATTAACTAATTATTTAGAT TTTTTTTAGTCTCAACTATTTTTTATG	A	G	ATAATAAATAAAATTAATTACAACAATAATAACA TAATAAGTATTTTATCCAACTCACAAGTGTCAAT TTAAGTACCATGTCATTAATGAGAATGTTACA		NA	NA
Ca-II-SNP744	266.14	Ca-*desi*-LG(Chr)4	7062809	CTTTCACTTTATCCTTTTCACCTTCTTATGAGGCTC ATGTCTTAGTTTCATTTTTGTCCACTTCCTTTTTCT TTTTATCAATCTCTTGTTAGTGCAAAAG	T	G	TAATTTCTACACTTAACAAATACAGTTAGTGGAT AGTTGTTCTTTATTTGTTTGTTTTTGTTATCATC AAAACATCAATTAGAGGATTGTAGCTAAAACC		NA	NA
Ca-II-SNP745	270.74	Ca-*desi*-LG(Chr)4	7112402	TTCGAAATGCCTGGAAAATGTTCGAAGTTGAGGG AAAGTTTCGAAGGTTCTGGGAAAGTTTCGAAACAT ACTTTCGAAAGTTTGGATCTGGGAAAGTTTC	A	G	ATATAAATAATATTTCCCATTTAGTTTCAAAAAA TTATAAATAATATATAAAAGTATGTTTCGAAAAT TTGGGTTTATCCAAACTTTCGAATGTATGTTT		NA	NA
Ca-II-SNP746	271.89	Ca-*desi*-LG(Chr)4	7235461	TTTAGTTTGACCGAATTTGGTTCAAACCGGTCTGA TATGATTTAGTCAAATTTGGTTCGAATCATCTAGT CCAATTTGGTTTGATTAGTTCTGATTCCAT	G	T	AAACGTTCCAGGGCTTGAATAGCACGCTCAATA GCCATCATAGTCACTTCCATTTTCTCCTCTGAA GTAGTTTCCCGGCCTCCATTTCCATGCAAACGC TTGTAATTTCAAACCCCTACATCATCCACCTTAA CTCACGGTATTGCCGCATCTAATTGTGAAATTG AATACCCCTTTGAATGATCTGGTAGAAACATTG AAAGGACTTCGT		NA	NA
Ca-II-SNP747	273.04	Ca-*desi*-LG(Chr)4	7268764	AACTGATTCGAGGACCTCTCAGAGACTTAGTGAAT ACATCTGCCAATTGATCATTGGAATTAACAAAACT AGTGGTGATGTCACCTGATTCGATCTTTTC	A	C	TGACATTAATATGTGATAATCAAGCAGCATTGC ACATTGCCTCTAATCCAGTTTTTCATGAGAGGA CCAAGCATATTGAGATAGATTGTCACTTTGTAA G		NA	NA
Ca-II-SNP748	274.19	Ca-*desi*-LG(Chr)4	7344673	TTTTTTTTCCTAATTTAATTCTTCCTAGTGTATGAG TCTGATGAAATCACGGTAGCACTGTGAATTTGTTG AAGTTTCAAAGTGTAGCTTTTGCCAGAAT	C	T	TACGTACCTACACATGCATATACATTGAATAAA GATAATGCTACATGGTACATGTTTGGATTAACA GCGAGAGTTTTGCTGAATCACAGTTTGCCACCA T		NA	NA
Ca-II-SNP749	275.34	Ca-*desi*-LG(Chr)4	7395192	TTTTTTTGTGATTTCGTTGTCTGAGCGCGACGTTT TTATTTTTTTATTTTTTTGTCATAGTGCGATGCTTT TGGGTTTAGATTGACGAAGTAGTTTTAGT	G	T	TTCCCATTGTTATATAATTTATTTTGGCTTCTAT AGTTATATCTAAAGTAGTATATATCTTAAATTTG AAACCCAAAAAAGTATAAATGTTGTGATTCTGG TTGCATACATATCTCAGTATACTGATTGTCATAA TACAAGACAGTCATCTAACCCTCGTAACCAGCT TGGCCCTCATGATAACTCGACTATCGCTGGAC GTTTTTTGGAACTAGGATTTCTCTTCTGTTTTTC [illegible]		NA	NA
Ca-II-SNP750	276.49	Ca-*desi*-LG(Chr)4	7578257	AAGCATTTGTAACCTCATGGAGGGGCAGATTTTG ATTATTATTTTTGTGAGGCTTTGCTCTCTAGAGTT CTTGAAAGAATTCATCTTGAACTTATCAAGG	C	T	AAAGGAAACTCAATATTATTATTCAACAACGAAA CAAAAAACAAAATGGCGCCACAATCTAGGGATT GATAAGCCAAAAAGAACGCCACAAAGATTAACA		NA	NA

Marker IDs	Genetic positions (cM)	Chromosomes	Physical positions (bp)	Flanking sequences/Forward primers	ICC 12968	ICC4958	Flanking sequences/Reverse primers	Annealing temperature (0C)	Amplified Product size (bp)	
Ca-II-SNP751	277.64	Ca-*desi*-LG(Chr)4	7632834	AATTTCTCAAACCTTATGTTCTAAAGATGCATTTCACCAATAAGTATGTGAGTGCTCAAGTGATCCACACCCCAACTGCTACTGTAGCCTCTTCTGCAAG	C	T	CCTTAAGTAAAAGGCGTTCTGCAAGTAGCTTCCCAATCTTTGAAGCTGCAGCTACATCCCGAGTAGTTTCCAAGTTTGATCTCAAGGCTTTCTCTTGTGA		NA	NA
Ca-II-SNP752	278.79	Ca-*desi*-LG(Chr)4	7672275	CCGAAGTAGGAGAACTAGAGTTCGGATGACTCTAAACCCATCGGAGAAAATCTTCATAGTTTGTCGGGGAGCCTTGTGGAGATGGTGTAGTCTGAATAGT	T	G	GTGAGCATTGTCAACAGCTTGGACACACTATTGATCGTTGTTGGAAGTTGCATGGTAAGCCTTCGCCTTCAATAAATGCAACTCTGCTTGATCCTTCTGC		NA	NA
Ca-II-SNP753	279.94	Ca-*desi*-LG(Chr)4	7834534	TTGATCTCTTAGTATCTATCACTGTTTATGAACCAACAATACTATCATTTTCACTCAATCTCATTTTTCTCCCTTGATCTGTCTTCTCTGAATTTCTATG	A	C	AGCCATACGCGATTTTCACTCAAATTTGTAAGTTTCCGACCTATAACGATCTTTAAAGTGAACTATGACTATTAGGATCATATCACATATACAATTAGAT		NA	NA
Ca-II-SNP754	281.09	Ca-*desi*-LG(Chr)4	7965918	TATACAATATGATTTTGAATCTGTTATGAGGTTATTGAATTTGGATTATTATTTTTGCTATAAATAAAAAGCAGAAATTGATTTTTGTGTGTGTGTTCTT	A	G	AGGACACCACAATCAAACAAAAGTTTACATCTACAAATTTTACCTGAACAACATCCACATAAAAAATTGTTCTCCAGTCATTGCCAACAACTTAATGCAT		NA	NA
Ca-II-SNP755	282.24	Ca-*desi*-LG(Chr)4	8112704	TTGCTTCATATATTTGAATGAAAAGTGTGACTTTATCTTTATTTCGCTCTTTCGACACACTCAAAATGAATCTTGGCACAAACTTATACTTTGTCATATT	A	C	GAGAGAGCGGCGGCGATTTTCTGCGACGGCGGTGCATCGGAATCTGTGGCATCGACGTAACCGACGGTGTAGAGTTGATCTAAAATTTGGTCGACAATTGAGTGTTTGTCCGATTTCTCCATGCTCTCTCTCTCGCTGTGATTTAGCAAAGCAGGACTCTCT		NA	NA
Ca-II-SSR201	283.39	Ca-*desi*-LG(Chr)4	8205928	TTTAGGCCGCGACATTTATT	(AT)7	(AT)6	TCACCGATAAATATAAGTAAAACACGA		59.59	NA
Ca-II-SNP756	285.69	Ca-*desi*-LG(Chr)4	8290399	CTCCATTCAAACGAGTTTTAGATATGCCAAAATTTACAAATTTCTGGTGCTTTATTGTCTGAGTTGGCTAGTAGGTGGGATGGACGTAGTGGGGGATTTA	T	A	GAAAAATGTTACACATTTGAATTTCAGTGAACATTACTCTATTATGTGTGAAGACTTGAATGGTTTTTGTAGAGATCCTTGGCATAAATTGAAGGCTACTCTTAGGCGTGATTATTGCAATAGTCCTTGGCAAACTGCTGCTTCTGTTGCTGGAATTCTACTACTTGTTCTCTCTTTACTTCAATCAGTTTGTTCTGTCTTGCAAGTTGTACAGCAATATAATGACTCCTCAGA[illegible]		NA	NA
Ca-II-SNP757	289.01	Ca-*desi*-LG(Chr)4	9740035	GGATGAGGACGGGTTATAATTATCGTGAGCTTGGGCATTTTGGATGACTAGGGGTATCCGGATGAGTGTTGATGGTGACAGTAATGAACTTACTCAGCGT	C	T	AAGGGCTGTTGAGGTAGAATGCAATTGTTGCCATTGTAGGTCTTTCATTTGGATCTTCTTGAACACATAACAAGCCAATGTGGACGTACTTGATTACTTCTTCATGGGAATAAGTTCCTTCTATATTAGGATCCAATAGTTCAAGAGGCGTTTGCTCTGCCCATTTTATCCAAGCGTATCTTCGGATGTCATCAACGCACTCTGAGTCAGTAGAACAACCTTTCCTCTTT[illegible]		NA	NA

Marker IDs	Genetic positions (cM)	Chromosomes	Physical positions (bp)	Flanking sequences/Forward primers	ICC 12968	ICC4958	Flanking sequences/Reverse primers	Annealing temperature (0C)	Amplified Product size (bp)	
Ca-II-SSR202	290.03	Ca-*desi*-LG(Chr)4	10865571	TGACGATGGAATCATTTAGTGG	(TA)15	(TA)14	ATTTCCTCTTCATTTATAATCCATTT		59.82	226
Ca-II-SSR203	291.05	Ca-*desi*-LG(Chr)4	11096813	TGTCACACATCAATGCTCCA	(TTA)18	(TTA)19	TCAGCATCAACCATCCTTCT		59.66	NA
Ca-II-SNP758	293.09	Ca-*desi*-LG(Chr)4	11420245	GTTTATGTCCAAACTCGATATGGTCATTAGGAATT<br>AGTCAAGTTGATTTTCAAGTACTCGAAGAGGTACA<br>ATAGATCTTGGTATCATATTTGATTGCGGT	C	T	AGCATACACAGGTTAGCTGCAAATTGTTACAAC<br>CACACAGTAGATGACTAGCGCTATCATCTTTGT<br>CTGCACAGCAAGTTACCTGCTATATGCATTAAT<br>TCGATCCAAGATGATTATGACATGAGAACTGCT<br>CACAATTGTCATCTGGTGGAGGCTTGAAAACCT<br>CAGAAACAAGTGCACCGCATCAA		NA	NA
Ca-II-SNP759	294.11	Ca-*desi*-LG(Chr)4	11444487	CCTGGGGCGGTTTTAATCAACCGCCGTCGTCGTT<br>TAGGGCATAAACGTGTTTTATTTTTCCAGATTTTG<br>TAAGTTCATTCCTCACTCTCTCTTTCGTTTA	A	G	TCTGGTGAGGCAACATAGCAATTGTGTTATTGA<br>ACAAAAGGCGTGGTATGTTCTTACTAATGTGAA<br>ACAAAAAGGGACCCGTAATCCCTGGGAAGACA<br>GAAAACTAACATAGTGATGAAATTGAAGTCTGG<br>AAATGTCTCTGTAAAAACACTAGCAGGCAGCTG<br>GACAATGTCATTCTCCTCAAAATAAATGAAACA<br>CAGTTATATTGCTAGTAGTTGCTGACTTCTATTT<br>[illegible]		NA	NA
Ca-II-SNP760	295.13	Ca-*desi*-LG(Chr)4	11568614	AATTGAGTCATTGGCCAATACTTATTTTATATGTTA<br>TTATCTTTTATCCATCTTTTGTTTGTTGAATTGAAA<br>GACTTGAAGTTCTATATCTGATAATAAT	T	C	CAAGGAACACAAAAACATTACAAAGGCATTTGT<br>GATTGTGTGAGTACAATAGTTAAAGAAGAAGGA<br>GCTCATGCTCTATTTAAGGGTATTGGGCCAAGA<br>GTGTTGTGGATAGGAATAGGTGGTTCAATATTT<br>TTTGGTGTTCTTGAGAAGACAAAGAAAATTCTT<br>GCTCAGAGGCAACCCCAAACTGATGCTCAGAA		NA	127
Ca-II-SNP761	296.15	Ca-*desi*-LG(Chr)4	11777085	GAGCTGCCTGAAACCAGTTCATCACAACTATTGTG<br>AGGGCCAAGCCAAAAGTGTATCCTAAAAATGCACT<br>CTTGAAATATTGGGGCTGTTTTCCTCTAGA	T	G	GAGGGTGCAGATGAAGCAGCTGAGCCTCCCCT<br>TGATGATACTGATTCTGCAATGGCACATCATAA<br>CAAAAGTTTAGCTGTAAGCCAATGGACACAACT<br>TTACAAATTAGCTATCTGTAGAATCTTGAGAAG<br>CTGTTACAAATCC		NA	114
Ca-II-SNP762	297.17	Ca-*desi*-LG(Chr)4	12182021	AATCGCTCGTAGGAAAATATTTACCGACGGACAA<br>GCTGTGGGACACACTTACCTAGGGATTGTCCGTG<br>GGTAATATGACCCACTGAGTATCTGTGGGTAA	T	C	GGCACAGTTGTTTATTATGATGGGCAGATGAAT<br>GATTCGCGGCTTAATGTTGGATTGGCTTGCACT<br>GCTGCTTTAGCTGGTGCGTCAGTGCTGAACCA<br>TGCAGAAGTGGTATCCTTACTGAAGGATGATG<br>CTGGAAAACGGATAATTGGAGCACGAATTCGA<br>GACAATTTAACTGGTGAGGAGTTTGA		NA	NA
Ca-II-SSR204	298.19	Ca-*desi*-LG(Chr)4	12587450	TCACAAGAGACTCCCTGCATT	(TAAT)6	(TAAT)5	TCAGAAGTTTCAGAACTCTTGTCAAT		59.86	NA

Marker IDs	Genetic positions (cM)	Chromosomes	Physical positions (bp)	Flanking sequences/Forward primers	ICC 12968	ICC4958	Flanking sequences/Reverse primers	Annealing temperature (0C)	Amplified Product size (bp)	
Ca-II-SSR205	300.23	Ca-*desi*-LG(Chr)4	12811509	AGCCCTTTAAGCAGCAACAA	(TAT)6	(TAT)5	GGATGTTGGGATCAAAGTGAA		60.02	NA
Ca-II-SNP763	301.25	Ca-*desi*-LG(Chr)4	12970386	CCTTATCTCCTTTTTATTTTTCACCTGCACAACACTAATTATTTTGTGAAGTTTTTATTGTTACATTCTAACAAAGAACATTATGTCTTTATTAAATCAA	T	C	CAATGTGTCAAATTTCATCATCATCTGTCACCTTAAGTCCCAAACACCAAGGCCTAGTTTTGACTCCACAATCAACTGTCCTTCTAAGAGACATACTAATTTCATGCCATATTTTTTTACACTCAATTCCATTCCTATCAAATCCCATATAAACCATTTTTTCACCTATT		NA	NA
Ca-II-SSR206	302.27	Ca-*desi*-LG(Chr)4	16332808	AAAAATTATAAAAACAGGGCATAGAT	(AT)10	(AT)12	ATAATATTTCACCAAGGTAGGTTTTT		57.17	NA
Ca-II-SNP764	304.31	Ca-*desi*-LG(Chr)4	16481864	GGAACAAGCTGAGTTAAGTCAGCTACAATCAACTGCAGTTGCTTCACTACCTAACTCTAATGGTTCACGGATTCCTCCGTGTAAGTCTATTATGCCCATC	T	C	CCTTGGAGGAGAGATTTGCCTCCTAGTTCTTAATAAAAAAGTTATTTCTTAGGAATCCAATTCTTGGGTTCCTAATAATTGGTATGACTCGGATTTTCGAGGAGTGCCAGTGTTCAAGGAGACGGAAGCCGAACCAAATGGTGAAGTTGCCAACATTTACAGGGATAGACCCCATTGGGCGGCTTGCAAGGGCTGAAAGATTCTTTGTGAAGCATAAAACTCGTGAGTTT[illegible]		NA	NA
Ca-II-SSR207	306.35	Ca-*desi*-LG(Chr)4	18058917	GTGCATGTACGCCACGTAAC	(TC)9	(TC)8	TCCCTTTCAATCTCCACAGC		60.07	NA
Ca-II-SNP765	308.39	Ca-*desi*-LG(Chr)4	18170443	GACTTTGTAAAGATTGCTCTATCTCCCAACAAGCCATCACTGCTTCCATTGCATGGACTCTGACATGTTGTTGTAGTTGTTCTTTGAAGGAACACAGCAA	T	G	GACATAGGAAGGAACATAACCCAACAAAACATATGTGTGTGTCTGAGAGACAGAGGAGAGGACAACAAAATCCTTACTATCACTCGTCAGAAAGAAGCACAACCAGAAATCTTGCGTACAGATAATAATAACAACCAATAAAACACAAATTACACTAGATGCCATTATTGCCATTACTCTCCTCCTCCTCCACAAAGGCATTCCTTATCTTCCCACGCAGCATGTCACAG[illegible]		NA	NA
Ca-II-SSR208	310.43	Ca-*desi*-LG(Chr)4	18680223	GAATTATTATTTCACGTATGATGTGC	(AT)9	(AT)10	TGAATGAGTTGAAGTAAATGCAGC		58.09	NA
Ca-II-SNP766	312.47	Ca-*desi*-LG(Chr)4	18833427	ACGACATGTGTGTCGCTGTCCACCGACTTGTATCAGACATCAAACACGTTTTCAATCTAGGTGTTACATAGGTTAGGACTTCCACTAATAGACTAGTCAT	C	T	CTGACCTCGTTCAAATCTAAGGTTTCATTTCATCTGCTGTGATCTGGTCCTGCGTCAGTACTCGTCTGCTCACAGCTGTCCAAAACGACGCTCCTCTCAGAAGCCGTGTGAGGTGCTCATGTCACTTTACTGTCACTTTCAGAT		NA	NA

Marker IDs	Genetic positions (cM)	Chromosomes	Physical positions (bp)	Flanking sequences/Forward primers	ICC 12968	ICC4958	Flanking sequences/Reverse primers	Annealing temperature (0C)	Amplified Product size (bp)	
Ca-II-SSR209	313.49	Ca-*desi*-LG(Chr)4	18897679	TGGGGTGTGAATGTGAGAGA	(AATAA)26	(AATAA)27	CAACAGGAATTTGCAATCACA		60.09	NA
Ca-II-SNP767	315.53	Ca-*desi*-LG(Chr)4	21255529	CCAAGGCTTAACAGATTGTTCTTCATGTTTGGCACATACAACACATCACATATGAATGATTGTTTGTCATCTCTCCTTTGAATCATCACTTTTCCTACTC	T	C	ACCCTCTCAAAGAAAAACTAGACATCAAACTTAAGTTAGAATAATTAAAAAAATACAAAATTACTCTTAGTAAGCACTCTCAAATAACAAACAAAAAAATTACCCCCTTAAAACAAAACTGTAATCTTTCAATTGTATTTGATCATTAACATCAAGGTAGATCTGAATTCTGTTGATGAAAATGAAAATGGATATATACACATATTGACATACATGTGATATTTGTCCTCAACTCTA[illegible]		NA	NA
Ca-II-SNP768	316.55	Ca-*desi*-LG(Chr)4	21675078	GATGATGTCGCTGAGCCTGCACAGATGGAGGAGGATGTCCCTGAACCTACACAGATGGAGCAGACTGAGGAGCAGTATGTTGAGCCTGCACATGATGATG	G	A	GCTTGAGTTCACAAAATCATATTTAATAATAAGTTAGCCGCATCATAAATATAAATAGGTGGAGTAAAGCTAAAGTGGCTACAGCTTTTACTTGTTAGAAAGGGTGAGAATAATAGTACATGGCTTAGGAGGGACTACATTACACTGTTGGTTCATTCCATTTGCAGGGTCATACAAAACCCACCAGATATTTCCAGGATAAAGAAAACAACCTGAGAATGCAACAGTGTT[illegible]		NA	NA
Ca-II-SNP769	0.00	Ca-*desi*-LG(Chr)5	180	ATGAGTGTTAATAATTCTGTAAAGCTTTTATCAGACCATCCATTACTCGCTTTTAAGTTGTACAACTTTAATATCACCGACAGTCTTGTGAATTTAGTAC	G	A	GTTGATGAGATCACAAAAGCAGTCGAAGAAGATCTTCGACATTGTCCTAAAATGTTTGAAAAGTTTGTTGAGTGATGCAGAGAAAGAATTATATAATGGT	NA	NA	NA
Ca-II-SNP770	1.21	Ca-*desi*-LG(Chr)5	563	TTTTAAACAAGGACAAACGACTCTATTTGGATCTTTTGCATTCTCTGCAAACTCAACAAACTCTTTCACTCCATTTTCATACTCTTTTGACAATCGATTG	C	G	TTGGTTTCTTTTTGAAACTTGTTGATATGTTTTGAAAGCTTATTATTTTTGTAACTGCATTTATATAGGTCGTATAATATGGATAGGAAATGGATGTCTG	NA	NA	NA
Ca-II-SNP771	3.59	Ca-*desi*-LG(Chr)5	124382	CCCCAATCGTTTCTCCTTCACAAAGGAGCTTTCCTTTCAGTCAGAATATCAAACGGAGTTCGCCCAATGGATTATATTACTCATAGGCAGTCAAACTCTT	C	A	TTATCAACTCGAAGACACTCTTTGAAGCAGAGAAATGAAGGTAACCTCCAAGCATCTCAATACCCTCACCATTGCTGGTGCTGAAGGGAATCCTGACATG	NA	NA	NA
Ca-II-SNP772	4.78	Ca-*desi*-LG(Chr)5	217556	TCTTAACCCATTACAACGGGGTAGTTTGAAGATGATCGACAACAACTTGATATATGCCTTTGTTGAAAGATGGCATTCTGAGACATCGTCATTTCACATG	T	C	GTTACTCTATCTACATCGGGTGGATTGTAGAACTCACCCCTAATAGGCAATCCGAGAAGATTGGCAACATCATCCAAAGTAATCGTCATTTCACCAAATG	NA	NA	NA
Ca-II-SNP773	5.97	Ca-*desi*-LG(Chr)5	264577	CCTCCAAAAACGACATTGCAAATCCAACAAACGACTTCCTCAACAAAACCTCACTCTTCCCTTCATTTGTTCCCTTCCTCCTCTTCGCCAACGCCACGAA	C	T	AGCGTCAGAAAGCGGCGTTGTTGCTCGCGGCTTCAATTGTTCGGCGTGGACCGAGTTTAGCTTCGGAGGTGGCAAAGATATTTGATTTCAAGGTGGCTGG	NA	NA	115

Marker IDs	Genetic positions (cM)	Chromosomes	Physical positions (bp)	Flanking sequences/Forward primers	ICC 12968	ICC4958	Flanking sequences/Reverse primers	Annealing temperature (0C)	Amplified Product size (bp)	
Ca-II-SNP774	7.16	Ca-*desi*-LG(Chr)5	350477	TATAAGTTGGTTAATTGATTCTTATATACTACTTAATTTTTAACTTTATAGGAGTTCCTTGATATTCCTGCAATCCCCTTTCTTTGCTCTCCTCGTCTTA	T	C	AAATCACTAATGTTTGTTTATAGTGTAATATAGTGACAAAATGATGAGTTATTTGTTTATAATTTACCATACATAGTATTGAGAATCTTGAGAAACCAAC	NA	NA	NA
Ca-II-SNP775	8.35	Ca-*desi*-LG(Chr)5	408429	CCTAGCCTCATAGATTTCTTCCTTTTGAATTAAAATCATAGATTTCTTCCATCATATAGTGATGCCAATTCAAAGTTTTATGACTTGCGATACAATGGCG	C	A	GGCCTTTTATTTACTAAAGTTGTGAATACATCATATACCACATGATGATCCACCTGGCCCGCCATAAAGAAAAGCTTGTCGATAGTAGGATCCTTGATAG	NA	NA	NA
Ca-II-SSR281	9.54	Ca-*desi*-LG(Chr)5	434482	TCATATCACTCGATGCACGG	(TC)6	(TC)7	AAAAATGTACTGAAGATATTTGTTGTG	60.65	111.00	NA
Ca-II-SNP776	11.92	Ca-*desi*-LG(Chr)5	459827	TGACTTAGGAGGCCTAAGCCCAAATTTTAAGAATAATCACCATTTTTACTTCTTTTGAACATTTAATTTTTTCTGCTCTATTGATCTGTTCTTCTTTCTA	C	T	GAATTAAAAAATGGCAGAATTTAAAAAACAAAAATTGAAAAAACAATAAAAAAGTAAATAAAGAGAGAAAAAAAATTTACAAAAATTGATATAGTTCACC	NA	NA	NA
Ca-II-SNP777	13.11	Ca-*desi*-LG(Chr)5	494008	TGTTGACACTCATAGTTTCTGTATTGCTGCTGGGATGTCTTGATTAACCATTTTACACATTTGTGATCATAAATTATGCGGAGCATTGATATCATGAATG	G	A	TGAAAGTGGTGAGGAAGGAAAACTAATAATAAGCATAATTAAGAATGTAACTGACAGATCATATGCAGCACATAATCAACTTTCACTGCATTTATGAAGG	NA	NA	NA
Ca-II-SNP778	15.49	Ca-*desi*-LG(Chr)5	576575	GTGCCTATGACTCTGAATTCAGCTTATGCACTACTTCTAGCTATCACAAACTGTTTCTTGCTTCTCCAAGTAACCATGTTTCCCCACACGAATGAGCAAT	A	T	TAGAGGTCTTCATTTCAGAAAAAATACAAATAGGAGTATCGAAGTATACACTGACTCAGATTGGGCTGGATGTACATCAAACAGAAAGTCAACTACTGGT	NA	NA	NA
Ca-II-SNP779	16.68	Ca-*desi*-LG(Chr)5	585987	AATGAAGTAAATGATTTATTTATATTTTGCTATAACAACTATATATGCATGCATATTTGCTCCGTGTTTGTTATTTGGAGATGACCCTTACATTTGACAG	A	C	AATCTAGTAAGCCGGATCACAATATTGTTCCATATCTTTTGCCATGCCATAAAAACAAAATGGCACTGGATGACATGGAACATATACAATTTGGGTTGGGTGACCAGGTTGTTCAATATACACACAAAAAAAGAACCCAGATTTCTCACCACTATGGAATCTTCAATCACCATTATTTGTGCAATCTGATTTTCTCTGTTTAGCAGCAAGTCGGGGCCTCAGCATTTCTAT[illegible]	NA	NA	NA
Ca-II-SNP780	17.87	Ca-*desi*-LG(Chr)5	613252	GTTTTGGGTCTTTGGTGGTTTCTTTCTGTTTTTATTAAGTTGGATTCAATGTGTTTAACTTAGGGTTTGTCTTGAAGGTTTTATTTTTCATTTTATATGT	T	G	GTTACAAATATGAATAATTCACACAATCAAACTAAACAAGACAAATAAATAACCAAAATTAAGAACAGTGAAACCAAAATTAGTATTTCCAAATATATTA	NA	NA	156

Marker IDs	Genetic positions (cM)	Chromosomes	Physical positions (bp)	Flanking sequences/Forward primers	ICC 12968	ICC4958	Flanking sequences/Reverse primers	Annealing temperature (0C)	Amplified Product size (bp)	
Ca-II-SNP781	19.06	Ca-*desi*-LG(Chr)5	717330	ACTTTATTTACCTTTTCTTTGTTGTGGTATTCATTTTTTTTGTAACATCAATTAATAGACTTATTAAGAAAGTGAATTAGAGAATTGGATCACTTCATAT	G	T	GAACGCATGTTCAACATGTAATCTTAAGTGATTTTATTTACAATTTTTGTATTTTCTACCAGAATATTTTTTTTAAATAGTAAAAAATAAGATAATGGTC	NA	NA	NA
Ca-II-SNP782	20.25	Ca-*desi*-LG(Chr)5	1367823	AATGAAGTAAATGATTTATTTATATTTTGCTATAACAACTATATATGCATGCATATTTGCTCCGTGTTTGTTATTTGGAGATGACCCTTACATTTGACAG	A	G	TATCTCGGAGCCTCCAACTGCATGTTATTGTACTTTCTCGCATTTTCTCCTATTAGGTTGATTGTGATATTATTCGCTCAAATAATACTATATGTGACGT	NA	NA	NA
Ca-II-SNP783	21.44	Ca-*desi*-LG(Chr)5	1419904	CATAGTGTTGAGAGAAACCCTTAGCAACAAGACGTGCTTTGTAGCGCTCAATTGACTTATCTGACTTAGTTTTGATCTTGTATACCCAATAGTTCTTATG	C	T	CTGCGATTGTTGATCCTCCTCGTTACCCCTCTCGTCAACTTTGTCTGAGCCTTCTTCCTATAAAGAGGTTGTTCTTGATCATCTTTGGCAGCAAGCTATG	NA	NA	NA
Ca-II-SNP784	22.63	Ca-*desi*-LG(Chr)5	1474370	ACTTGCTTTGCAATTCATGGATTCATTTCATACCTATCTAACCCTCTCTCATTCTTTGTCTTCCACACTTCGACAGGTCAAATTCAAAACTTTAACCTAA	C	T	ATGAAGTAAAAGTTGAATAAAAGTCGATGCAGTACAAAACAGTGAATGGAATATAACAAAAAATTGAAAGGGCTTAATTTAATGATGATAATCAAATTTG	NA	NA	NA
Ca-II-SNP785	23.82	Ca-*desi*-LG(Chr)5	1492078	AATGTACATATCCTTGACCCTATGAGGCGATAAATTCTGAAAGAATTGAACTTGATCAGTTTCTTCAAGTGTTTGAGTCTCTTTAGGAGACTCAACTTCT	A	G	TCTGAGACTAATGTTTATAATTGGTTTCAGAACAGAAGAGCTCGTTCCAAGCGCAGGCAATTGGTTTCTACAATAAATCAAACAGAGCCAGAGGCTGAGA	NA	NA	NA
Ca-II-SNP786	25.01	Ca-*desi*-LG(Chr)5	1523220	TGTTGTTATGTTTGCCTCTTATATGAGTAGCCAACCATGCATTAATTGCAACTACTCTTGGTAGATAGTTGTGTCGGTTATATGTTACATTGAGTCTTCA	A	G	GCTAAAAGAGAACTAGAGGAAACCACACATATACATAATGGAGCAAGGGATGTAATTATAAAAGAAGCTAAAAGAGAACAAACTAACATGCGGAGAGTTG	NA	NA	NA
Ca-II-SNP787	26.20	Ca-*desi*-LG(Chr)5	1562648	TTGACACGTCCGTTGAATATTTGCTTCTAATTATTCTTTTTATAAATGACTTTAATTTTTTTAATTGTAGCGGATCAGTGCATACCAACTTAATTTTTAT	G	A	CTGTTCCCTAGTTTATTTTTTGGTAAAGATAGTTAATAAATACTAAGTACTGATTTAGTGAGCCGGTCCATTTAATCCATATTTCACATATACCGGACTA	NA	NA	NA
Ca-II-SNP788	27.39	Ca-*desi*-LG(Chr)5	1699761	AAGCATTATTTCCTAATTTCCATTCAAAAGAAACCATTGCTTCCAAGGAAGAATTGGAACCATTTTCTTCGTTTTCTTCTCGCATGGCTGCACTTGATTT	T	C	AAACATGTAAACGGTGTAGAGCATACCTTCGTCCAGCTAATATTTTAGCCATGTTACCATTGTTATTGGTGACAAATACATCGCTTTCATCGCAAACAAT	NA	NA	NA

Marker IDs	Genetic positions (cM)	Chromosomes	Physical positions (bp)	Flanking sequences/Forward primers	ICC 12968	ICC4958	Flanking sequences/Reverse primers	Annealing temperature (0C)	Amplified Product size (bp)	
Ca-II-SNP789	28.58	Ca-*desi*-LG(Chr)5	1777675	TTGGTGATAGATAGGTGATTCTAGTGGTGTAGTAGTTGTATTTTGTTTGATTTAAAATTATGAAACATAAGTTTTTGGAATTGGATCAAGAATCCCAATT	C	G	CTTGGAAAATCAGATGGCAGTTGCACTCGGTGGAAGGTCAGTGTACCATGCAATACACAGACATGCTTATTACCTTATTCGATTTTCGATCTTGATTTTTGCAAATTGCAAGTTACTCTTGCATCACAATTATGCACTACTTTAAATCGGTTGCTTGGCCTACTCCCGAGGGGATGTATAAGAACTGTTGTTTTAGTATTGGCCTACTC	NA	NA	NA
Ca-II-SNP790	29.77	Ca-*desi*-LG(Chr)5	1800042	ACTCAATTTTTCTGTGGTTTAGTCTTTAGTCCCCACTCCTACCCCATTCTTTTTAAAATATAAATGACGCGTTCAATAATTGAAAAATGTTATCATTACT	T	C	CTGCGCCCCTTTCTTATTATTGCATTGCACATGTGGCCAGCAACCACTTTGTAACTCTGAGCTACGCAGCAAATCAGTTACTACTTCCGAATCTCTGATT	NA	NA	NA
Ca-II-SNP791	30.96	Ca-*desi*-LG(Chr)5	1991486	TTTCAGGCCTTGCAGGCAGCATCAAAGCTCAGAACCCTACTTGGAGTCCCTCTGCAATCAGATCAGCAATCATGACCTCTGGTCAGGCTTTTCATTTTTA	T	C	AAATCAATTGACTTAAAACGACCAATAATTTTTTATTTTAGAAATCATTTTAGTATTCAAAATTACTGATATTTGGAGACTAAATTGATGATTCATTTGT	NA	NA	NA
Ca-II-SNP792	32.10	Ca-*desi*-LG(Chr)5	2003951	TTGGTGATAGATAGGTGATTCTAGTGGTGTAGTAGTTGTATTTTGTTTGATTTAAAATTATGAAACATAAGTTTTTGGAATTGGATCAAGAATCCCAATT	T	C	TGCATAAGATACCAACAAAGAAAACAACAAAAAACACACCACAAAAATCCTTGCACAAAAGGAACACAAGAAAACATCATCATCCACCAGTCATATTAAA	NA	NA	NA
Ca-II-SNP793	33.24	Ca-*desi*-LG(Chr)5	2027873	CTTCTTATTTTGTGCAGATAAATTTATATTATTTTCATAATTCCCTCATGTTTTCCATGTAAATTAACACATTAATTCTATTTTATTAACAACTTTCTCT	C	T	AATTATTTTATTTGATGTTATTAATATAATTTACTATTTTTTAATTATAATTATTTACTATTATTATTTTTATTACTTTTTATTAGATTCGGATAAAAAT	NA	NA	NA
Ca-II-SNP794	36.66	Ca-*desi*-LG(Chr)5	2423599	TTTGAGCTAGTGGAAGAATAACCTGACCTCCAAAACCTCCTGCCCTGGCCCCTGGCAAGGTACAGGGACCGGCTTTAATGGCAGCAGCTGAGTAAGCATC	C	G	CGGGGCACTACTTATCCACGCCACAACCAGAAATTCAGCTGAAAAATGCAAAAACGAGCACAATAATCACCTCTACGGTTCCCTCCTTTTTTTTATCCACGTGTCTCACCCTCCTCATCTCAATTCACAAATAACCATCACACCGTCTTTATTCTTCGACCGTCGTCTCGCATTGCAATTTTGCGAAATATAAACCTCTATTCCATTCTTGTAAAGGAATAATACTAGTATT[illegible]	NA	NA	NA
Ca-II-SNP795	37.80	Ca-*desi*-LG(Chr)5	2597436	TATTCTTTAGTTGTGTTGTGTATCATTAAAAATGTACTACAGTTAAAGTAGGGAACATGGTGGCTTCTAACATGGACTACCGTCCATGATACACTAGGCA	C	A	GTAGAGAATGGTGACACAAGGTTGAGGATAGAATCAAAAAAATATAATGTGGACAGACTATAATAGAAACAATGTTCCTGCTTAATCTAATGGTCAAAAG	NA	NA	NA
Ca-II-SNP796	38.94	Ca-*desi*-LG(Chr)5	2763336	CATGTGTTCTGCATGGTCTAGATCATATGTTTGCTTTTTGTTTGCATTTTCTCTACCATGTAGACGAGTGGGGAATATGTTTGTGCATAATTTTATGTGA	A	T	ATAGCTGAAAATTTTCATGTTGACCTAAATAAATCATTAAATTCCCTGTTCTGTCAATTGAGCTATGTTGCAATGCTACAAAGTATTCCAATAACAATTT	NA	NA	NA

Marker IDs	Genetic positions (cM)	Chromosomes	Physical positions (bp)	Flanking sequences/Forward primers	ICC 12968	ICC4958	Flanking sequences/Reverse primers	Annealing temperature (0C)	Amplified Product size (bp)	
Ca-II-SNP797	40.08	Ca-*desi*-LG(Chr)5	2789253	GAATTTATTTGTATATGGACCATTTGGCCCAACCTAAAAGGCACGTTGAAGGAAATGGGGCTGACCCCAAACCTTTTAACCATATTTTGGGCCTCCCGTA	C	T	CCATGGGTTGAGTATACAAATATGAAATTTAAGAAATTTTACGGAGAAGGGGGTATAATTGTGAGATAGATGATAAATTTTTTAACATAAAAACAGCAGA	NA	NA	NA
Ca-II-SNP798	41.22	Ca-*desi*-LG(Chr)5	2958738	GCATTTTTCCTTTCAACTACACTACCAAAGCCTGCTATATGGCATTGATAGTACCTCACATCACAATTGCTTTGCAAAGTTGTTTTGCTGCATTTCTTTG	A	G	CCATGAACTTCACTCACTGCAACAAATGATAAACTCTTATCAGTTCCCATAATTCTGCAATGCATATTGGAATAACATTTGAAATTACACAGAAGAACGC	NA	NA	NA
Ca-II-SNP799	42.36	Ca-*desi*-LG(Chr)5	3010287	CTCTATTTTGTACCTAGGATCCAAAACACCAGCAACCCTCATTCCTTCATGAACCACATTCTAATATTTTTCAAATTTTCCCAACATATTCTCTGCCATT	G	C	AATATAAAAATCAACAGAGTTAGCGTATTCGAGCTACTTAAACTTAGTGATGAGTGATAACTTTTCCCAAATTTCTTTTTAAATATTTTAATTGATTCGAATTATTCTTTCACAACAACACAAATCCTATAGCTACTAACTCATGATGTTTGGGTGACTCCAGCTTTAATTCTTGACAGGGTTGTGACATTAATTAATTTAAGAGTGAAATTAAATTATCTCTATCTAAATGGA[illegible]	NA	NA	NA
Ca-II-SNP800	43.50	Ca-*desi*-LG(Chr)5	3024118	AAATGACTATATATTTGTCTTGCCGCCAATTCCAAGTTGCTCTTTGGTATTTTTTTCAAGGCACTCTAATCCCAGTGTGCTTTCTACCTTGTCTCAATTT	A	G	GTCCCATCGACTCAACCTTGGTTTTGGTTGGACGAAGGGACTATCATTGACTATTCACATTGTGCTGAGACAAATGACTTGAAAGTTGGCCAATAGAGAC	NA	NA	NA
Ca-II-SNP801	44.64	Ca-*desi*-LG(Chr)5	3052692	GCATGGTTTTTGAGAAATTGTTAAGGAAGACGTAATTACCGAGCACCGTTGGCGATTCTGTCGATAGCGACCGTAGAGAAGAATGACGATCGGGAGCTCT	A	G	CGGTACGTACCATCACTTTATTTCTCTATTTTAATTTTATCTTTTTACTTTTCTTAATTAATTAATTCAATATTTCATTTATTCTGTATTGTATAGATTA	NA	NA	NA
Ca-II-SNP802	45.78	Ca-*desi*-LG(Chr)5	3134685	TTTGAGCTAGTGGAAGAATAACCTGACCTCCAAAACCTCCTGCCCTGGCCCCTGGCAAGGTACAGGGACCGGCTTTAATGGCAGCAGCTGAGTAAGCATC	A	C	TTTCCTTTCATGCTCTTTTTCTGTCCTTGTAGAATGTTCCTCCAGCTATTCTTCTAAGATTCTTGAGGGAACACAGATCAGAATGGGCAGATACAAGTAT	NA	NA	NA
Ca-II-SNP803	46.92	Ca-*desi*-LG(Chr)5	3153611	CTAATTCAGTTAAAATGGATCAATTGGGATTTTTCCACATGTAGTTAGAGACTCAGAGCATCTCCAACGGTAGCTAAAATGAGTTCGTCCGTCAGGTGTA	C	T	AAACAAATTTGATTTGGAGGCACTATATTGGCTATGTAAGTTTAGCAATTGTGATTATTTTTGCATGTGCGTTTGTGGTTGGCTTTAGCATTGGGGTGAATGTGAATGCTATGATGGACAGCCTTCATACGTTGTTTACCGTAGATCAGTGCTTCAAAATCATCTTAAATTCTAACGAATATCAACTATACTTCTCGAAATAGTCCTATTGTACTTTGTGGGTGGGACAATG[illegible]	NA	NA	NA
Ca-II-SNP804	48.06	Ca-*desi*-LG(Chr)5	3233511	CGAGATCTGCTCACGCCCAGCCCTCTTTTGTGCTCTGACTACATACAGAATCTTTGAACCATGTTGTGATCCATTCATTGCTTCCATTGCCCTTTGCATC	G	A	CTTCGTTTCTCTTCAAGACAGCAATTCATGCAATTGAGCTGAAGAATCATTCTACCTTGAATGGAGAAGCGACAAGGGTCATGTGGTCGTGTTGTGATCC	NA	NA	NA

Marker IDs	Genetic positions (cM)	Chromosomes	Physical positions (bp)	Flanking sequences/Forward primers	ICC 12968	ICC4958	Flanking sequences/Reverse primers	Annealing temperature (0C)	Amplified Product size (bp)	
Ca-II-SNP805	49.20	Ca-*desi*-LG(Chr)5	3310181	GAGGGGGACAACAGCTGGAAACAATTGCTAATAC CCCATAGGTTGAGGAGCAAAAGGAGGAATCTGCC CACGGAGGGGCTCACGTTTGATTAGCTAGTTG	A	G	TCAGTAAAGAAGCGAACTATTGATTATCCTCTC GTCCCAGTGTGGTTGATCACCCTCTCGAACTA GCTACTGATCATCGCCTTGGTAAGCTATTACCT CA	NA	NA	NA
Ca-II-SNP806	50.34	Ca-*desi*-LG(Chr)5	3319950	CAGGCCAGGCCAGGCTTTGAAAGGCCTGAGCCT GGCCTACGATTTATTTTTTAGGCCTAAGCCTGGCC TACGGCCTATCATAGGCTTTTTTTTCGGCCTG	G	A	TGCAACTTTGAGGTGCAGCGAGAGATGTTTCG GCAATTGGGATGGATTCTGTACTCTACAAGATG TGGACCAAATGCTATGGATGCAATTGAAGAGCT TATGCAAATTTTTGTTGACCAGTGTGCCTTCAT TGACTACTTCAAGAGTCATAGTCTTGCTATTAT AGATGTGTGGATTAATGGCATAAATTCGCTCCC TGTGACAACTCTC	NA	NA	NA
Ca-II-SNP807	51.48	Ca-*desi*-LG(Chr)5	3364304	TTAGATATTGTGCAAATACACGGTTGTGCGACCTG GTTGACACATACACTGTTCTTGCATACACTCTTTG CTTAAAACACTGTTTATTTGTGCGGTGAGA	A	G	AATAAAGCAACATTCAATAACCCTTAAAGGAGT CAAATTAATTTATAGAAGTTTCTATAGCTGGGC TTGTGTCCCAAAGCACATTAGACATACGTTTAC A	NA	NA	NA
Ca-II-SNP808	52.62	Ca-*desi*-LG(Chr)5	4406861	TTTTCGGAAACGTGAGTATCGTTTAGGGTGAACG GTCTTTGAGTTTTGATAATTTTGTTCTAATTGTTTT GTGTCGTTTACTTAATTTCAGCGTTAAGAT	G	T	AACTATTAAACATGTTTGGTACCTTCATGATAAC TTCATACACATCATCAGCAATCAAAGGTGCCTT CTTCCCAGATCTATGGTTGAAATGATTGTACAT	NA	NA	NA
Ca-II-SNP809	53.76	Ca-*desi*-LG(Chr)5	4475969	CTCTATTTTGTACCTAGGATCCAAAACACCAGCAA CCCTCATTCCTTCATGAACCACATTCTAATATTTTT CAAATTTTCCCAACATATTCTCTGCCATT	T	A	CACTAAATATTCAACTTCCAATCAATATTTTCCT AAAATTTGTATGATCAAAATTGCAATAAATGAGT GGATTAAATCCTCTGATGCATTGATAAAAAAA	NA	NA	NA
Ca-II-SNP810	54.90	Ca-*desi*-LG(Chr)5	4512060	TGTACAGATGCACACACTGTGGAACAAGTTCAAA GTCTACCCCTATGATGCGAAGGGGGCCATCTGGC CCAAGGACACTTTGCAATGCTTGTGGGCTTTT	C	G	TAAAAAGGTTGAGGTCTACTTCAACAATTTATG GACACTCGCATCTCTCAATTCTTCAGCTTACAC AAAAACAGTGATGGATCAACAGTGGCAAGTTGA GAGAAAGGTTCCTTGTTGGTCACATTTCATCAA ACCTAGAGATAGATGCAAGTCACCTCTGCCTCA ATATGTGGAGACACATCATGTTGCAGGATATCC TATTCTGTCTGATCCTTACATGTTTGAAGTATCA [illegible]	NA	NA	NA
Ca-II-SNP811	56.04	Ca-*desi*-LG(Chr)5	4539668	TCTGTGTATCACTGAGGCAATTAGTAGAACTGCAT CCCAATGACAAACTTACAGTGTTTATATCTGGATT GGCAGTTACAACTTGTTTTTCAGCAGGAAG	G	A	TTTGTAACACTGATTTCTTCAGAGAAATTTTTTA AAAGTTTCCATGCTTTAATATATTTCTATTTACT GCAGGTGACATGGAATTTAAACAGAATAAGTT	NA	NA	NA
Ca-II-SNP812	57.18	Ca-*desi*-LG(Chr)5	4643427	ATTGAATCCTCCAAATTAAGTATTCATTGAAAAGG TCCTTAACCTTGTTATGGTTTCCTCCATTGATCCC GATGCATGCATCCGTATAAATACATAAGCC	A	G	AGAAACCAATTACTGAACCAAAGTACATTTGCA AATGCAACAGAAACAAACTCTTTGGAGTATAAA CTATTTTTTACAGCATCTGGCATTATTATGCAGA	NA	NA	NA

Marker IDs	Genetic positions (cM)	Chromosomes	Physical positions (bp)	Flanking sequences/Forward primers	ICC 12968	ICC4958	Flanking sequences/Reverse primers	Annealing temperature (0C)	Amplified Product size (bp)	
Ca-II-SNP813	58.32	Ca-*desi*-LG(Chr)5	4658083	TTGCCTTATACTGATATGCTGGAAAATAGTGCATC TTTTGTTTTTAAGCTTTTGGCAAGGGGGCATAATT TGCGTTTTTCTTTATATTCTATCATCTTTC	T	C	CAACACTGAATTCATGTAAAGAAGCTTGTGTAA GAAACTGTTCTTGCCTTGTGCTTTTCTATGAAA ACAGTACTGGTAGTTGCTTTCATTTTGATCAAA CTGGTAGCTTTTCAAAAGATTTTAAAGGTAGCA ATGGTGGTTATATTTCATATATGAAGATTTCAAC TGATAATGTTGAGAATGATGGAAAGAATAGTAG TGGAAAAAAGAACATGCTGCTAGTTTTTCTCAT [illegible]	NA	NA	NA
Ca-II-SNP814	59.46	Ca-*desi*-LG(Chr)5	4925461	GGTTTTTGTCAGACATTTGTTACCCTTGCGTGGCA TAAGATATTTTGTAAGATGTTCTGGAAACTTCTCT GCTTCGTCCACTTATCCATTCAAATAAATG	C	T	CAATGGTCCACAACTAAAATGGGCTGAGTGAAA GTCCAAAGCACAAGAAAAACAATTTGTAGTTGA TGCAAACAACAAAGTCAAAAAACTGGTGTTAAG T	NA	NA	NA
Ca-II-SNP815	60.60	Ca-*desi*-LG(Chr)5	5234822	TGTACAGATGCACACACTGTGGAACAAGTTCAAA GTCTACCCCTATGATGCGAAGGGGGCCATCTGGC CCAAGGACACTTTGCAATGCTTGTGGGCTTTT	T	C	TTTTCCAATTTCAACTGCGCTAGATAGTTGAAA TATGAACAGGAGGTTTCCCACCAAAAAAAAAAA CAGAAGGATGACAAATACTTACCCTATTTGCCC A	NA	NA	NA
Ca-II-SNP816	61.74	Ca-*desi*-LG(Chr)5	5261100	CATAGTGTTGAGAGAAACCCTTAGCAACAAGACG TGCTTTGTAGCGCTCAATTGACTTATCTGACTTAG TTTTGATCTTGTATACCCAATAGTTCTTATG	G	A	ACAGTTATGTTGACATTTTATAGGGTTTCTTCCT GTTTTTCATTTTGCGGGGAGGTAAGGCCAAAGT TTTTCGTGTTAATGGTTAACGGATATCCACGTG GACATCTGCTATGTACTCCTTTGAATATAAATAC CATGTG	NA	NA	NA
Ca-II-SSR282	62.88	Ca-*desi*-LG(Chr)5	5265717	AACATGGTTCACTTAATTATACGATTC	(TTA)5	(TTA)7	AGACAAAAACGGGCTAGTTAAA	58.11	164.00	NA
Ca-II-SNP817	65.16	Ca-*desi*-LG(Chr)5	5280375	ACTTGCTTTGCAATTCATGGATTCATTTCATACCTA TCTAACCCTCTCTCATTCTTTGTCTTCCACACTTC GACAGGTCAAATTCAAAACTTTAACCTAA	C	T	CGGGTCAGGAAAAATTACATACTTTCCCCATTC AGCTGGAGGATTCCACGCTGACAAAATCGTAT CAGATTTGGCATTGGGAAGACAGTTTTTGAAAA CCAAATACTTTGTTGTAAACCGAGAAACACCAA CATCCTTAT	NA	NA	NA
Ca-II-SSR283	66.30	Ca-*desi*-LG(Chr)5	5290196	CTAACCAGGATCCGGAGGA	(AT)8	(AT)9	GAGACGGGATAATAATTGAAAATGA	60.01	164.00	NA
Ca-II-SNP818	67.44	Ca-*desi*-LG(Chr)5	5302276	GAACAGACAGCAATCTCTTTTAGGCCACCAGATCT CTGTACAAATTTAGCATTTTGCATATCAAACAATG CCACCTTTGTCACTGGCCGATTTTCCTCCA	T	C	TGTCTCTCTTGAAATCTTCCAATTCAGTTTGTG CAGGAGCAAAGAGAAACAATCAACACAGATTGA GCTTGAAAGAGCCAGATCTATCTATCAGGAATA T	NA	NA	NA

Marker IDs	Genetic positions (cM)	Chromosomes	Physical positions (bp)	Flanking sequences/Forward primers	ICC 12968	ICC4958	Flanking sequences/Reverse primers	Annealing temperature (0C)	Amplified Product size (bp)	
Ca-II-SNP819	69.72	Ca-*desi*-LG(Chr)5	5312055	ATTTGCAAAATTGCCCTCCAGTCTTTATCCTTACGGTAAGTATGTTATAAGGATGACATCTATTGTTTATCTCCATATCTAGTTTAAGTACGAGGGTCGA	T	G	GGGAAAACAAAAACCCTAGAAAGGGAAAGGGAAAGGGGAATTGATATTTGAAAGCGAGGTGATCGGAATTTGATTCTTCAGATCGGATCCGTGAGATGGTCGGTGATCGAGGAGAGTGTGTTGTTAGAGGGAGAAATCATCGTCTGCTACGCGGGTACAGCTTTGGGTTTGGGAAACGTGCTGCGACACTCAAG	NA	NA	NA
Ca-II-SNP820	72.00	Ca-*desi*-LG(Chr)5	5337897	GATTGACTATTAAGTTGTGACCTTTAGATTTGGATTAGATGCATTCAAAATACAAATTTGAATTCTCTACAGCCGACTTTATGGTGCAGCTGGCTGTAGA	C	T	TTTTGAAGAAACAAAATCCTCTACATCCGATTTTATGGTGCAGCCAGTTGTAGAATGTTAGATTAGGACAGAGAGAAACTTTATCTCTCTTAATCTAATG	NA	NA	NA
Ca-II-SNP821	74.28	Ca-*desi*-LG(Chr)5	5409164	ATCCATAATTATATTTTATGAAGTTACAAGTCATACTGTAAGACCCATAATTTTAAAGTACCATTTTTTGTATTTTTGGTATATTTTGGATTTTGGCTCG	A	G	TTAAATTCAATTTATTTATCGACGAGAAATTCTAATTGGCATCGAAATATCTTTTTGATTATAAAACTCCAAAATATTAATAACTTTGGCTTAAAAGCCT	NA	NA	NA
Ca-II-SNP822	75.42	Ca-*desi*-LG(Chr)5	5425955	GGCTAGGGTTATGGGTCGTGTACTCCACAGCCTTTTTAAAAATAGATTTCACAGTGATTCTTTTTCTTAAGCAGAAGATTGAATGTTCTTGAACAATCAA	A	G	TCAAGGTTTTGAAATACACTTTTTACATTTGTTAGACGATTTCGGAATTTCATTTCAACAAACTTGTCTGGTTTGTAATTGGGATATCCATTTTCTCATT	NA	NA	NA
Ca-II-SSR284	76.56	Ca-*desi*-LG(Chr)5	5468760	CCGGAACCGAGTCTCAAAT	(TG)10	(TG)9	AAAAGCAGCATGGTCAGTTG	60.06	171.00	NA
Ca-II-SNP823	77.70	Ca-*desi*-LG(Chr)5	5481759	GAGCAACGTCGCCTTGTTGCATCTTCAGAACATGTTGTGTATCAAAGGCAACATCTCGGAGCAAGTTGTAGTCTTTAGAACGAGTGAAACTTGAGCTTCA	G	A	TTAAAAAATAAAAGTAATTGAATGTAACTCATATATATATATATAGCATGCCTTTGAAGCGCCTACTATAGTAACTCTGAAGTTTCTATTTGAGTTGCTT	NA	NA	NA
Ca-II-SNP824	81.12	Ca-*desi*-LG(Chr)5	5611691	ATTTGCAAAATTGCCCTCCAGTCTTTATCCTTACGGTAAGTATGTTATAAGGATGACATCTATTGTTTATCTCCATATCTAGTTTAAGTACGAGGGTCGA	A	G	AAATAGGTGATGTACTTGACATGAGTGACACACTGGTCAAAGTGGTTCGAAAAGAGAAAACAAATAACAGCAATCACATCAAAACTTAATAAAATAGAAA	NA	NA	NA
Ca-II-SNP825	82.26	Ca-*desi*-LG(Chr)5	5650676	TTGATTATTTCTTACTCGAATTCAATGTTTGGCAAGTGCAGCTGCACGGATTGAAGTATATGAGTGGGTGGAATTTCTATTGACTCTACTGGGATGAGAA	G	A	ATACCAGTACCTAGAAAAATGTATTGCAGTAAAATCCAATGACTGGGACACAATTCAACCTATAAATAAATTACTACACCCGCTAATCAGTTATTCTCAA	NA	NA	NA

Marker IDs	Genetic positions (cM)	Chromosomes	Physical positions (bp)	Flanking sequences/Forward primers	ICC 12968	ICC4958	Flanking sequences/Reverse primers	Annealing temperature (0C)	Amplified Product size (bp)	
Ca-II-SNP826	83.40	Ca-*desi*-LG(Chr)5	5686299	CAGGCCAGGCCAGGCTTTGAAAGGCCTGAGCCTGGCCTACGATTTATTTTTTAGGCCTAAGCCTGGCCTACGGCCTATCATAGGCTTTTTTTTCGGCCTG	A	G	TTAGGGTTTAAAGGATGTTTGCTTCATATTTTTTAAAAATTATTTTAAGTAGGCTTTTAAATAGGCTTCCAGGCCAGGCCAGGCTTTTAAAAAGGCCAGG	NA	NA	NA
Ca-II-SNP827	84.54	Ca-*desi*-LG(Chr)5	5973615	CACCTTCTGGGAGTTGGGGATTGCAGCTAGAATAGTCCGAAGTCCAGCCACAGCATTTTTATACCGAAAACGGGCTTCTTCAAGAGCGCCACCAGCACCG	C	T	ACTTCTCCTTCCGCTGCTACCTCTCCCAACGCTCCTTCCTCAAACGGAGACGCCAATTCTGATAGCTCCAGCCAACACGCCGACGGCGCTGCTCCCGGTG	NA	NA	NA
Ca-II-SNP828	85.68	Ca-*desi*-LG(Chr)5	5992600	CATATCTTTTCTTTCTCTATAATTTTTTTTCTCTCTATTTCTCTTCTATTTTGTATCAAGGAACCACCTCCATGATACACATGTTTTGAAATTTAGTTCT	T	C	GTTAATGCATGATTGCATAAAGCACTTGTTGGATTTTGGGTTGGAGCATGTTGCAATAATTTGAACAAGTAGAAGATGAAGATGGATATGAGTATACGAG	NA	NA	NA
Ca-II-SNP829	86.82	Ca-*desi*-LG(Chr)5	6134976	TCGAGGTTAAGGTTCTACATTTCAGTGAGGCACACACCCTTGGTGCTTTTGACATGCTTTTAGTGCCCCTTATTGATAACTGTAATTAAAAATCCCGTAG	T	A	TAGCTTTGGATCCTCATTTGAACAAAACAGGGGACGTTTGGCACATATGTATTGAGGATCTTCCTAGAAGCAATGTTCTTTATGGATATCGGATAGATGGACCTCAAGACTGGGGTAAGGGGCATCGATTTGACAGAAGCATTGTGCTTGTTGATCCTTATGCAAAACTTATTGAAGGTCGAAGACATTTCGGGGATATTAGCATGAAGTTGTCTAAGTTTCTAGGCACA[illegible]	NA	NA	NA
Ca-II-SNP830	87.96	Ca-*desi*-LG(Chr)5	6333200	CCCTTCAAGGACGACGTGAAATCTTTGATACTATAATTCGAGACTCATTGTCCTTTTAAGGTATCTCGTGACTCTTTCGATAGTGTGTCAATGCTCCATA	C	A	TATGCGAGCATCATTGACAGCCTCAAGTATGTCACTGATTGTACTAGACCCGACATTGCCTTCGTCGTGGGATTGTTGTGCAGGTTTACTAGTAGACCTA	NA	NA	NA
Ca-II-SNP831	89.10	Ca-*desi*-LG(Chr)5	6509887	GGCGCTTGCACTCTATCATTTAACTTTGGTTCAGAGTAACCGAGTCAAGCTTGTGAAACTTGGGATGGTACCCACGCTTATTTCGATGGTGAAAACGGCG	G	A	CTCACCAGACACTCCACCGCATTAGCGTCCAGCATCGCCGTCCTTCCCTCCACAGACATTGCCAAATTGCACAGAATCAGCAAAACCCTACTCGCCATTG	NA	NA	NA
Ca-II-SNP832	90.24	Ca-*desi*-LG(Chr)5	6543566	TTAGTTCCAAGTGGAGAAAATTCTTGGGATGGTTTAGTTCCAAGTGAAGTGTACTTTTGATGGTGGAGTATGATTGTCGGTAAGACAATGGAAGCGGAAG	C	T	CCCAATATATAGCCTTGACCTTCTCTTTATTCTCCCACACTAAAGGATGTGAGACTTGCAATCAACCCCAACAATCTCCACCTTGATTGTAAGAGTTCCA	NA	NA	NA
Ca-II-SNP833	91.38	Ca-*desi*-LG(Chr)5	6596225	CTAATTCAGTTAAAATGGATCAATTGGGATTTTTCCACATGTAGTTAGAGACTCAGAGCATCTCCAACGGTAGCTAAAATGAGTTCGTCCGTCAGGTGTA	A	G	AAGATAACCATATCAAAGTACCGTTCCTCCGTGAGAGAAGGGTGATGACAACTTGACACATCTCCAACAACGAACTCACCAAAAACTGAAGGTCACGTAA	NA	NA	NA

Marker IDs	Genetic positions (cM)	Chromosomes	Physical positions (bp)	Flanking sequences/Forward primers	ICC 12968	ICC4958	Flanking sequences/Reverse primers	Annealing temperature (0C)	Amplified Product size (bp)	
Ca-II-SNP834	92.52	Ca-*desi*-LG(Chr)5	6675851	TTATACGATTTTACGTGTATATACGCACACCCAATTATTATATCATTCTTTTATGCGTTTGACTATTATTAAATGTGTACTTAATTATATTTATTTAATT	T	C	ATCATAATAAGTATATCAAAATAAAGAAAATCAAACGCAATATTACCACTATCTCAAGATAATTATTTATAAAGTAAATAATTAAAATAAAATATATTTA	NA	NA	NA
Ca-II-SNP835	93.66	Ca-*desi*-LG(Chr)5	6815620	TTGCCTTATACTGATATGCTGGAAAATAGTGCATCTTTTGTTTTTAAGCTTTTGGCAAGGGGGCATAATTTGCGTTTTTCTTTATATTCTATCATCTTTC	A	G	ACTTAGATATCAAGTCAAACAACTTAAAGAAAAGGTGAATTGATAGTTAAAGATATTCTAACTAAAACAAGAACAATAACGTTCAAGAGTAAGTTACAAC	NA	NA	NA
Ca-II-SNP836	94.80	Ca-*desi*-LG(Chr)5	7026863	GCGTGAGGATGGGTTCTGCCTGGCCTAACAAGCTGAATGACAGTTCTGTAATTATGATGAAAAACCTTGGATCCTTTGAAAAATGATTTCCAACGGTCAT	A	C	TGCTTCTTGAAAACTTTAAGATGCTTGATCTTTTTATGCAATTGTGTTGTGTGTAATCCAATGGAGAGCTTGTTAGCATTTTATAGAGATCCAAGCCCTT	NA	NA	NA
Ca-II-SNP837	95.94	Ca-*desi*-LG(Chr)5	7061392	AACCAGCACAGGTGGTAAGGTAAGTGTGCTTGTATCTTTGTTCTGCTTTAAGCCAATTTTAATTGTGGATCGCAACTCAAAATTTAGTGTTGTCAAGAAT	C	G	TCTTCCATTGTCTAAATTGTTTTGATTGAAGTTATATTGCACCGATGGTGAAAAGTTGATTTCCTTGTTTGGTATGAGAATGCCTTAAGGTTATATTGGGGAGTAATATAAGCTATTATGGGGGGATGTTGGGTTTATATGGATGCATAAGAATGTTGCACGTTTTGTTGTAAAATATATTTTTGTGGGCGTCA	NA	NA	NA
Ca-II-SNP838	97.08	Ca-*desi*-LG(Chr)5	7119562	TGGCTGAAACAATTTTTAGCCGGACTTTACTTATCTCTAGGCCAAACTCCAGATTACTAGGGTTCTTTCCCCTAAGAACCGGAGGTTTAATACCAAACAA	A	G	CTAATAAGTACTATACAATATAGATTCTGGTTAATTACGTAGCCCTGAGTTGCAAATGGGATGCACCAGGATGGAACCTGATGCTGGTATTTAGTATTTA	NA	NA	NA
Ca-II-SNP839	98.22	Ca-*desi*-LG(Chr)5	7177410	CGACCTGCCGGATTCGAACCAGCGACCTAAGGATGACAACATGATTTTTCCAACTACAGTCCTCCGCTCTACCAACTGAGCTAAGGTCGGTTCTGTTATG	A	C	ACAGAGTTCCACTCTCAACACTGCTTACCTCTCTTCTCCAATGAACAAAAACACCTCACGGAAGCGTGCGGCGGCGGCACCGGCACCGCCGCAACAACCACCGACGAAACAGGCACTGTCTCAGGAAGACGAGTTTCTCGACGAAGACGTGTTCCTGGACGAAACCCTCATTTCGGAAGACGAAGAGTCCGCAATTCTCCGTGACATCGAGCAACGCCAGGCCCT[illegible]	NA	NA	146
Ca-II-SNP840	99.36	Ca-*desi*-LG(Chr)5	7236249	CCTTCAGAGGACGGAGACAAGGAAGGCTGAGGGATGGCTGGAAATGTTGGAGGAAGATTCAGCATAGGTGGTTCAAGACCAAGCTTTGGGGCAAGCTGAT	A	T	CTTACAAGAATATTCCCAAAAATTTTGATGCTCTACAAACTATCATTGCGCGCCAGGCCCATCAAGATAAGGAACAGGCAATTTTGCGTGACTGGATGGA	NA	NA	NA
Ca-II-SNP841	100.50	Ca-*desi*-LG(Chr)5	7253490	TTCGATTTCATCCCACTGACGTTGAGCTGGTTATGTATTTTCTGAAGAGGAAGGTTATGGGTAGAAAATTCCCTTATGATGTCATTGCTGAACTTGACAT	T	A	TGAGAAACATAAGTACTATTATAATACTCTACCATCCAAAGAAAAATAACCACTCAGTGTCCAAATCCAAACCTGGTAGATCCCATGGAGCATACTTGTA	NA	NA	NA

Marker IDs	Genetic positions (cM)	Chromosomes	Physical positions (bp)	Flanking sequences/Forward primers	ICC 12968	ICC4958	Flanking sequences/Reverse primers	Annealing temperature (0C)	Amplified Product size (bp)	
Ca-II-SSR285	101.64	Ca-*desi*-LG(Chr)5	7263577	CGGCGACGATAAGAAGACTC	(TC)14	(TC)15	GCATTCGCATTCACATTCAC	59.98	145.00	NA
Ca-II-SNP842	102.78	Ca-*desi*-LG(Chr)5	7268725	AATATTGTTCCCAGGATTGATCTTCGTTTTTCAAAACTGTTTCAAGATCAATCTGCGTTTTCCAGAACTGCTTCAAGATCAATCTGCGTTTTCCAGAACT	A	C	TTTATTTTACAACCGTTGGATTTTATATCAGCTCAATGTCAGTGGTCTTCACGGTTTATGCTTTCTTATATGGAAAACTTTATTTGTCACTGAGTGGAGTTGAGGCTGCTATAGTCAAGTTAGCTAGAAGGAAGGGAGACGATCCTTTGAAGGCAGCAATGGCTTCGCAATCCCTTGTTCAAATT	NA	NA	NA
Ca-II-SNP843	105.06	Ca-*desi*-LG(Chr)5	7292924	TTCAGGGAGAAGAAACACTTTCCGTTTATTTGGAATACGTCTCTGGAGGTTCTATTCATAAATTACTTCAGGACTATGGGGCCTTCAAGGAGCCTGTTAT	C	T	GCTTTAAATAAATACAACATCAATAGCAGAATTACCTATGCACTGTATTTCTCCCGTGAAGATAGGCAAGTCCAGAGACAATCTGTCTGGTATAATTTTG	NA	NA	NA
Ca-II-SNP844	107.34	Ca-*desi*-LG(Chr)5	7336211	TTGACTTGTCTATGGAAATCAGGAAACTGTAGATGTACTGTTGAAATTTTGTTTTTGTTTAACTGCATTTTATTCCTCAAACTGCTGCAGTTTACGATGT	C	A	TGTTTCCAACAAGCATCTTGATGCAGTCCTGATTTGTTGAATAGAGGTCTATTTCCTTAGCCCATACTTCAGAGAGATTTGTAAATGTATCTCGCCGAGT	NA	NA	NA
Ca-II-SNP845	108.48	Ca-*desi*-LG(Chr)5	7359768	CTGCCTCTTCGCCGTCTGAGTGCGACGTTGTTTTGTATGTTGTCTTGATGCAACATTATTTTTTCCCATTTTGTTGCGGTGTTTGTTTGATTCATATTGA	G	C	GTGATATAATTAAAGTGTTTGAAATATTCATGCATTTGGAGTTTTAACGTTGAAGATGTCAAAATAATTGCTTGAATTTATAATGGATAGAGGTTAATTT	NA	NA	NA
Ca-II-SNP846	109.62	Ca-*desi*-LG(Chr)5	7440439	TTGTATCCCCTGATAATTGATTGGTTGAAATAATGCTATTAACGATGTATATATTATACTGATAAATTTGTATAAGATTGTATTTGTTCGTTTGGGCGAG	G	A	TACAATATCACATATTAATTCACCCGAAACTAGCAGATACATTCATGACCATAATATCCACATCATTCATTATTAACATTCTCATTCATTATTGACATTG	NA	NA	NA
Ca-II-SNP847	110.76	Ca-*desi*-LG(Chr)5	7498913	ACATTGTTCTTTTGATTGTTTACTCCCAGGTGAAAGGTTTGGATGTCGATGCTCTATACGTATCTCATGTCCAGGTCAACCAAGCTCAAAAGCAAAGACG	T	A	ATATATAAGATTAAGAATGTAACAACAATATACATACATTAAGACATAAATAATATAAGCACAACATACGATTGATTCTTCCGTGAGCACGGTATGTACG	NA	NA	NA
Ca-II-SNP848	111.90	Ca-*desi*-LG(Chr)5	7636893	ATCACATACCCTATCCTCCGTCCTATCATACATGAATCTGCAACCTGGTTTAGGCCCTGATCCATGAAACACCAATGAACATCTTTTCCGGGTTTGGACT	T	C	GCGGTGAAATTCAGCATCAACACCATGCCTGATGTAGAAAATGGAAGACAAGAACTTAATCATGAGAATGCAGAGAATGACGAGTACAGTATTGTTACGA	NA	NA	NA

Marker IDs	Genetic positions (cM)	Chromosomes	Physical positions (bp)	Flanking sequences/Forward primers	ICC 12968	ICC4958	Flanking sequences/Reverse primers	Annealing temperature (0C)	Amplified Product size (bp)	
Ca-II-SNP849	113.04	Ca-*desi*-LG(Chr)5	7749351	TTCGATTTCATCCCACTGACGTTGAGCTGGTTATGTATTTTCTGAAGAGGAAGGTTATGGGTAGAAAATTCCCTTATGATGTCATTGCTGAACTTGACAT	C	T	GTCACGACATGAACCTCCATATGATCCTCTTAGAGTCAAATCATAAGCGCCGTATTATATTGCAGAATACTATCTTTGACAGCAAGTTCTTCAAAAGAAGACAGTCTCAGGTAACCAATTTCATGTTCTCAATGCTGAATATTTGGAGAAGCATTTTCCAGCCTGGTTTTTGATAATGAAGTTCCTG	NA	NA	NA
Ca-II-SNP850	114.18	Ca-*desi*-LG(Chr)5	7800119	TTTTTTCAAACCTTATATTTTAGAAGGCTTGGCCTATGATGGATCGACTATGTTTTTGGTTATTTTAGGATCTTGCACTTTGATTATTTTGATGCTTGCA	T	A	ATACACATACAATAAATATCAAAGTTGTAAATTTTATCATAAAATACTTGCATGCATGAAAATAATCAAACTTGTAGAAGGCTAAAATAACATAAAATAT	NA	NA	140
Ca-II-SNP851	115.32	Ca-*desi*-LG(Chr)5	7834440	ACTGTATGAGAGTTATAGATATGGCTAGTTATAGCTTTAAGTAGAACACTTTTCAAGCTTGTTAGTGCATGTCTGGTTTCAATTTTGGAGGGGTCAAAAT	C	G	TACAAATCTTAACTTCAAGTTGGAATCAATTCTGGAGGCAGAATCAATTCTACTCAACAACATCCAAACATGCCAAAATCAATTTTACACATTCAAAATT	NA	NA	118
Ca-II-SNP852	116.46	Ca-*desi*-LG(Chr)5	7851698	TTGTTTCAAGTTGTGCCTTTTAATATTTGATCCTTTTGAGTGATTATTTTGCTCATATGAGAGTTGTATATTCCTAGTACATGAACTGGAAATCCATTCA	C	A	AAGATCTACTTCAAAAGATTAACAAAGACAAAAGCTAAGCATCAAGAACTAACAAGCAAGATCAAATCTGTTTTGAATGCAAGGAAAACTCATGCAAATT	NA	NA	NA
Ca-II-SNP853	117.60	Ca-*desi*-LG(Chr)5	7881404	GGGTTGAATAAGGATTTTGCTGTTTAAAAATTTTCAAGTGTTTTAAAAACTATCCTCTTGCAGAGGATGACAAGCCCGAGGATGGGTTTTTCAAAACCTT	T	C	GGTGTGAGGACTGGAAGTAGCCATCTTTGGGTGAACCAGTATAAAAACAGTGTGTGTCAATCTTATATTTCTCTTTCACTCTTTAGCTCAGTTCAAATCT	NA	NA	NA
Ca-II-SNP854	118.74	Ca-*desi*-LG(Chr)5	7952610	ACATGGCATATATAAAATGTGCAGCGAGCTTAGTACAAACTCATACCTTTCTCGGCCTTTTGGCTAAGATCAAGTGTAGTATCTGTTCTTATCAGTTTAA	T	C	AAGCAACACTTGAGAAATCCAATAGCAAGCTATTGTAGTGAAATCTCCCCCTCAACAAATTAATTTAATATCGTGTGAACCATTGGTTCACATATCAGAT	NA	NA	NA
Ca-II-SNP855	119.88	Ca-*desi*-LG(Chr)5	8013361	TAGGAGGAATGCTAGCGAAACACTCTCTAACACACACTTTCTAACCCACTCTCTTTGATTGGTTAAAATTCATTTAGGTCTCACTAAATCATGTGGGTCC	C	A	CCAAATGCTAGAAGTTTCTTGTATCTAATTAAAAAAATAATGCTAACAACACACTCACTTTGATTGGTTAAAATCCACATGGGTCCCACCAAATTCATAT	NA	NA	NA
Ca-II-SNP856	121.02	Ca-*desi*-LG(Chr)5	8089920	TTGATCATATTAGCATTGATTTCATGATCATTAATCTTTTGGCCGGGCATGCCACCAATAAAATTCAAGGATCGTGTGGAGAAAATGAGACTTGGTTCCA	T	C	TAACATTTATTATTGGTCCAAAATGAAATATATTCACTCAAAAACTAAAGTGCACATGAATATTTTATTAACATATTGTATGTATACACACAATCATAAA	NA	NA	NA

Marker IDs	Genetic positions (cM)	Chromosomes	Physical positions (bp)	Flanking sequences/Forward primers	ICC 12968	ICC4958	Flanking sequences/Reverse primers	Annealing temperature (0C)	Amplified Product size (bp)	
Ca-II-SNP857	122.16	Ca-*desi*-LG(Chr)5	8339399	TTTATTTGGTGTCAGTTTCAGTTGTGTACTCAAGTTGTCTTTCTGCATAGGTGCATTATCAATGTTCTCTTTATCCTTGTTGTCCTATTCGTACTTGTTT	T	C	CATCAAGGCAACTCAAAACTATGATCAAGCACAAAAGTTTAAAATAAGCAAAACAACATCTAGAACCAGAATAAAATCAATCTCCATATTGATGACCTAT	NA	NA	NA
Ca-II-SNP858	123.30	Ca-*desi*-LG(Chr)5	8490412	AAAGAAATAAACTTAAAACAAAAGAGTCCTTGTTTCTCTGTTTTGCCGTATTTCTCTGCTTTGCCGTGTGCTAGCTTTGACGTGTACAGTGCGTCTCCAT	A	G	ATTTTACACTGCTATGGGTTGTTGTTCTGGATTTTACACTGGTGTGGGTTGTTGTTATGGATTTTACACTGCTTTAACTCTATGTGGAGACGCAACCTCA	NA	NA	NA
Ca-II-SNP859	124.44	Ca-*desi*-LG(Chr)5	8551737	TTATTATTTCCTTAGAATTTTGAGTTTTGTTAATTTTTTATATTTTTATATTATTACGTCTTGTGTTTATAATTTTATAATTTTATTATTTTAATACATC	A	C	TAGATCCCAACCATTGTCCATTGGAGAAATGATTGATTTTTTAGTATATCTAAAAATTAATTCACTCAAAATCACAACTATTACTTTTTAAACAATCAAA	NA	NA	NA
Ca-II-SNP860	125.58	Ca-*desi*-LG(Chr)5	8598213	CGACCTGCCGGATTCGAACCAGCGACCTAAGGATGACAACATGATTTTTCCAACTACAGTCCTCCGCTCTACCAACTGAGCTAAGGTCGGTTCTGTTATG	G	A	AAGACAAATTCATTGATATCAGATATCGATTACCTGCTCAAATAAATCATTGGATTATCTTAATGCATTACATATTGTGATAATCGCATTACCTGAGGGG	NA	NA	NA
Ca-II-SNP861	126.72	Ca-*desi*-LG(Chr)5	8629542	ACTCATAGGCGAACCTCTTAAATCATCAACCTTAGATCTATCTTCTTCAGCCTTTTCCTCTTGAGGTTTCAACCGCTCTTGATAAGCAACAAACTCTTCC	A	T	CTTGAAAATCCCTAACTCTGTTAGTTTAATCCTTCTTGGGTAACTGAAACAAATAAGAAATGAAACCTTCTAGAACGAGTGAAGGATTATCTGTTAATGG	NA	NA	NA
Ca-II-SNP862	127.86	Ca-*desi*-LG(Chr)5	8685227	CCATCCTCTTCCTCCAATAATCGGTGTCAACATGAGATTGTATGCATATACTACCATTTTAATTTTTTCTAAATTTCAAAATATCGTTATCTTAGGTACC	A	G	TAAAACACATCGCACACAAGAAGGTTCTCACATATTAAGTGGGCCCTAGGTCCCCCCACAAAAACAAAACAATGTCACATTTTATATATAGAATGTCCTG	NA	NA	NA
Ca-II-SNP863	129.00	Ca-*desi*-LG(Chr)5	8772605	AACTTTAGTTAATAGTATCTTACGATACATGCAAGTCGTACCGCTTTTCGATATAAAACTGAACCCAATTGATACGTATCTCTACTGTGTATCTCACTGG	T	C	AGGGGTAAAGCACTGTTTCGCGCAGGTCGCGAGAGCGGTGCCAAATCGAGGCAAACTCTGAATACTAGATATGACCCCAAAATAAGAATGGTCAAGGTCG	NA	NA	NA
Ca-II-SSR286	130.14	Ca-*desi*-LG(Chr)5	8857368	GAGTCAAATTAACAAAGAAGCATAGA	(AG)6	(AG)7	AAAATGCCCACAAGAGCAAG	57.47	124.00	NA

Marker IDs	Genetic positions (cM)	Chromosomes	Physical positions (bp)	Flanking sequences/Forward primers	ICC 12968	ICC4958	Flanking sequences/Reverse primers	Annealing temperature (0C)	Amplified Product size (bp)	
Ca-II-SSR287	131.28	Ca-*desi*-LG(Chr)5	8858941	TGCCCAATATCTTATATCCAGG	(AT)10	(AT)13	TCCATTGATTATTGCTTTTCAA	57.99	218.00	NA
Ca-II-SNP864	132.42	Ca-*desi*-LG(Chr)5	8895007	AACCAGCACAGGTGGTAAGGTAAGTGTGCTTGTA<br>TCTTTGTTCTGCTTTAAGCCAATTTTAATTGTGGAT<br>CGCAACTCAAAATTTAGTGTTGTCAAGAAT	T	G	ATAAGTGAACATGATTATAATTTTCTAATTCGTT<br>ATAAATGAAAGAGCAATTAATAGGACAATCTAT<br>ACTATATCTCTTAGTCTCATCCTAGTTGGAAGA	NA	NA	NA
Ca-II-SNP865	135.84	Ca-*desi*-LG(Chr)5	9147463	CCATTCTTTTCAGCATCTTGTATTCACCATATCAA<br>GGTTTATTCTTTATTTTATTCACCATATGTAGTTTA<br>GCATTTTACAAAAGTTGGTAATGTGATAG	C	T	TGACAATATAGAGTCCACTCACCACCATCAGCG<br>AATCATCTAATCCTCCACTCCAGTGTTGTTTGA<br>GAACCTACATTGTCAATCATCTTTCACCATGGC<br>CATTCGAACCCTATTCACTAGAAAAACCCTAAC<br>CTCTATTCTCCGCAATGATCCAAACCCAATTGT<br>TGCAGGCATAGCAGCATCAACTCAATCCCGTG<br>ACTTGCATGTCTTCACGCTACCCGATCTCGCTT<br>[illegible]	NA	NA	NA
Ca-II-SNP866	136.98	Ca-*desi*-LG(Chr)5	9152669	GGCCTTAAAATCATCTCCTTTATAATCAGTGCTGG<br>AGCAAGAAGTGATCACCTTAACCAGATTCCGATG<br>CCTAACATTTTTCAAAGTTTCACATTCTGCA	G	A	GTGTTTCTAATAGTCATGATTTCTAAATTGTTTT<br>TCCTTTATTTTTGGGTTCAGTTACCTGTCTAGC<br>CAGTAGTTCCAGAAAGCTTGGTGAAGTTAGTGA<br>AGAAATTAAATTTATTCCTCATTTGCATAGCTAT<br>GGTGGTGGTAGTGTTGGAGGATATGGTAGTGG<br>AGGACCTAGTTTTGGTGGTGCATTTGGAGGTG<br>GATATGACCATGGAGGATCAAATGGCGGAGGA<br>[illegible]	NA	NA	NA
Ca-II-SNP867	138.12	Ca-*desi*-LG(Chr)5	9317619	TGCTTAATTGCACTTTTGGTCCTCCTATTATAGGT<br>GAAAATTAAAAGTAGTCCCTCCATTTTGTTTATCC<br>CCAGTTTTAGTCCCCAAACTGAATTTCAGT	C	T	ATCAGAAATAATATAATAATGAGTTTATTCCACA<br>TAAGCGTTTGCCACGTCACCATTTTATTGACAT<br>GTCATTTAAAAAATAACAACTCATCATGATTTG	NA	NA	NA
Ca-II-SNP868	139.26	Ca-*desi*-LG(Chr)5	9393004	AGGTGGTTTGGTTACAGGTGATACCAGTGAATGA<br>ACATGGGTTTTGGTTTGGAGCCAATCATGGAGAA<br>GAGATGGGTTTGGGAGAGTTTCTTTGAAGTTG	G	A	ACCACCACCACCTCTCTCTTTCTCCTCCTCTCT<br>CTATTTCTCCTCCACTTATCACCATCCCACTCTT<br>CCTCTCAAAAAGACCCAACTACTTCACAACTTA	NA	NA	NA
Ca-II-SNP869	140.40	Ca-*desi*-LG(Chr)5	9415820	TTTGAAAAAAACTGTGTTTTCTGGAAAATACGGGA<br>ACGGTCGTACAGGATCGGGATGTGACAGTGGTGC<br>AAGATTAGGGCAATTCGCGGCCGCGAGCACC	A	G	GTGTCATAATCACATAATCTCTGTCATCAATTCC<br>GGAATCCCGGTGCAGTAGACAGACTTACTGCG<br>ACGCCACCAGCCGTGGAAACGCCTTCGACGGG<br>TG	NA	NA	NA
Ca-II-SNP870	141.54	Ca-*desi*-LG(Chr)5	9447895	GCTTTGTCTCTCTCTCTGAATAGCGCCACTGTGAA<br>GAGAGATTTTAGACAGAGAAAAAGAAATGAAAGAA<br>TCTCGTTTCCCTATGCATATGTTTGTTGGC	G	A	TAGTTTGAATGAGATGATTAGTAAAGTAAAAAC<br>GGCACCGTTTTTAGCCATTATTGTTGTCCTTTC<br>CCTTTAGTACGAAAGTGAAAAAAGCCCAAACTC<br>T	NA	NA	NA

Marker IDs	Genetic positions (cM)	Chromosomes	Physical positions (bp)	Flanking sequences/Forward primers	ICC 12968	ICC4958	Flanking sequences/Reverse primers	Annealing temperature (0C)	Amplified Product size (bp)	
Ca-II-SNP871	142.68	Ca-*desi*-LG(Chr)5	9467589	TCGAGGTTAAGGTTCTACATTTCAGTGAGGCACACACCCTTGGTGCTTTTGACATGCTTTTAGTGCCCCTTATTGATAACTGTAATTAAAAATCCCGTAG	A	C	TGTTGCAAAACATAGTCCCACGGATCAGATAACTTTTTAACAACATTCAGACTATTTTACTTCCCTTATAATTATTCATATTACATTATGTGGCTGAGCT	NA	NA	NA
Ca-II-SNP872	143.82	Ca-*desi*-LG(Chr)5	9486442	TACCATTGTTTATTTATTACATTATGTAAGCCATAACATTTCTTTCTATCTCCATCTTCTGCCTCACACTTATATTCATCATTTTCTCTCACACCCTCTT	A	C	AACATATGTTGAAATCAAGCATAATTATTGACAAAGCATAAATGGGGGTCAATAAAGATGATTGAAGATGTTGAAGTTGTTGAAAATGAAGGAAGAAAGA	NA	NA	NA
Ca-II-SNP873	144.96	Ca-*desi*-LG(Chr)5	9533963	TATTTCCTTTTTCTGTCTTGTTACGGTTATTTATTTTCCTCTTTGAATAAAAGATCAAGCATTGAAATTGAGGTTTGGTGTTGTTGAACTAAATTTATTG	T	G	TGAAATTTGCTTGAATCCTAAATAAACGTCGGATAAATTTTACTAATCGATCACCCAAAAAGAACGTTCTATCCATGTGCTTTTCCTCTCATGCTATTTC	NA	NA	NA
Ca-II-SNP874	146.10	Ca-*desi*-LG(Chr)5	9591040	TCTTAGATCGGTTCATTAAGAGGTTTCCAATATATATTTTTTTATAACAATGATACACATATACTCCTAATTTTTTTGTACTCATTCAACATTTCATTTT	C	A	CACCTATAGAAGTGTATACACTTTGAAAAATAAAGAGATAAAAAAAGTAACGTGCTAAATATAATATATTGATAATGTGATAGAAAGAGAGACATAAAGA	NA	NA	NA
Ca-II-SNP875	147.24	Ca-*desi*-LG(Chr)5	9616056	GTTGTTTTGTTTGAACGCTATATTGCATCAATTTTTGAAACACTTCTTTTTCGTTAGTTATGGTAAATTTGGACTTTTCATTGCAAAGTATTTGGATTCA	G	C	GTTCCAGAACTTATTTTGCTCAATTAAGTTTTTTTTTCTCAAGCAGAGGATTGAATCTTCTTGACTAATCAAGAATGAGAAAATTTGTATCCCAACAGGT	NA	NA	103
Ca-II-SNP876	148.38	Ca-*desi*-LG(Chr)5	9639080	GGCCTTAAAATCATCTCCTTTATAATCAGTGCTGGAGCAAGAAGTGATCACCTTAACCAGATTCCGATGCCTAACATTTTTCAAAGTTTCACATTCTGCA	C	G	GTTTACAAGGGTGTGTTCAACATCAGCACTTGTGAAAATCAAACCACACTTGCTGTTAAAATTCTGGACCTACAACAAAGCAAAGCATCTCAGAGTTTTA	NA	NA	NA
Ca-II-SSR288	149.52	Ca-*desi*-LG(Chr)5	9669602	AAAAATGACCCAGAAAACTTTATTT	(AC)8	(AC)7	TCAAAATCTTCGTCTCACTTTGA	57.85	127.00	NA
Ca-II-SSR289	150.66	Ca-*desi*-LG(Chr)5	9669764	AAACCCCAGAGAGAAATGAAAA	(AG)12	(AG)11	CAAAAAGTCAGTGCAGCGAA	59.11	164.00	129

Marker IDs	Genetic positions (cM)	Chromosomes	Physical positions (bp)	Flanking sequences/Forward primers	ICC 12968	ICC4958	Flanking sequences/Reverse primers	Annealing temperature (0C)	Amplified Product size (bp)	
Ca-II-SNP877	152.94	Ca-*desi*-LG(Chr)5	9849475	AATATTGTTCCCAGGATTGATCTTCGTTTTTCAAAACTGTTTCAAGATCAATCTGCGTTTTCCAGAACTGCTTCAAGATCAATCTGCGTTTTCCAGAACT	T	G	AAGATAGGTATCAAACATCACAAATCAGATCAAAGAAGCAGTTCTGGAAAACGAAGATTGATCTTGAAGCAGTTTTAGAAAATGCAGATTGATCTTGAAG	NA	NA	247
Ca-II-SNP878	154.08	Ca-*desi*-LG(Chr)5	9929528	TTAACTTCCTTCTCTCTCTCTCTCTCTATATATATATATATATATATATAGTTAGGGATGGGTAGGGTTTGGGTAGGGTACTATAGTATCCGTTCTCATA	C	T	TCAATTATCTCAAATAATATTGTCATATGAGTTGCAACTTCACAAAATATTATTATGGATATTTAAATATATACAGTATGAATATTTTTTTAAATCGCAG	NA	NA	NA
Ca-II-SNP879	155.22	Ca-*desi*-LG(Chr)5	9941575	ATGAGATTATGTCTAATTGTCATCCCTACTTTTGCGCTATTTGTTGTTGTCTACTTCTTACATTTAGGGGCGGAAATATGTCCATTGATTTATAGGAGTT	C	G	TTTCCAATTGAGACATGGGTCCACAATGAAATGAGAAAAATATGGTGTAGTAAGTATATGTTTAATAATGAATGACAAACTACAAAAAAAACTTGGCTAT	NA	NA	NA
Ca-II-SSR290	156.36	Ca-*desi*-LG(Chr)5	9946606	CCGGCTTAATGAAAGAGGAA	(AT)9	(TA)9	GCTTCTGCTATGCTCTTCTGG	59.29	138.00	NA
Ca-II-SNP880	157.35	Ca-*desi*-LG(Chr)5	9988382	CTAATTCATCATTTTCTTCTTTGAACATTGCCTCCACTTTATATTAGAGTTAGACATTACATTACATATTATCATTTCAAGATGGCTTTTCAAGGGTCTC	A	G	GCTTGCTTTTCAATGCTATGCTTTAAATTATGAACCCCTAAAAAGTTCAATAATTAATCTATCAAGGGCTTATAATATGAATGAGTGTATTTTTGTCTTA	NA	NA	NA
Ca-II-SNP881	159.33	Ca-*desi*-LG(Chr)5	10001294	TATCCTAACCCGTATCATGCAAAGCTTAGAAACTTTATAGTTGATGATGAGATCCTATGATGCTCTCCACTTACAATAAGATCCTATGATCTTGCTACCC	T	C	CTAGTGTAAATATTACAAATCAAATCCTTATCTTAACAGAGCAAGGAGAATATTGCTTTGTCAGGTTGTCAAGATTGATATTTTGATCAAGATCAGGAAG	NA	NA	NA
Ca-II-SNP882	161.31	Ca-*desi*-LG(Chr)5	10104591	CCTCTTCACTTCTATTATAATCATTGAATTTGGCTCCTCTAAAGTGAGTGAATCAATGGTTGATCATCACCCATGATTGGTCATGCATGTACACATAATA	T	C	AGTGAGCAACTCACTTTAGAGAATAAAGTGAGTAATATCAACCATTGATGATCAAATGAATGGTTGATATTATGTGCACATGCATGACCAATCATGGTTG	NA	NA	134
Ca-II-SNP883	162.30	Ca-*desi*-LG(Chr)5	10115516	CCTTTACTATTACTAGGTCTAGGACTAGGACTTGGACTTTCTTCTTTTCCCCTCCTTTCTCTTCAACCAAATTTTACTCCCTTATGGATTTTTCGATATA	C	A	TTCGTCGTCTGGTGGTACTTTTTGGACAATTGTGGATTCTAGAATTGGCTTTGAATTAGAGTTTTTCTTCTTGGCGTAGAAATTAAGGAATTTTAGGGGA	NA	NA	175

Marker IDs	Genetic positions (cM)	Chromosomes	Physical positions (bp)	Flanking sequences/Forward primers	ICC 12968	ICC4958	Flanking sequences/Reverse primers	Annealing temperature (0C)	Amplified Product size (bp)	
Ca-II-SNP884	163.29	Ca-*desi*-LG(Chr)5	10554811	CTCAAATCTATCCTCGAATTTGGATACTTCATCCT CTAATTTTCATATAGACACATCTTGACTCATAATTC TTCTGGACTTCTTCCAAGAGATTTTATGT	G	A	TACTTAACAATAATAAAGATTTGAAAAGCCTAAT TCATAAATATAAATATGATTGCCCTTAAGTCGAT GATGATCATCGCAATATGTTGCATCTCAATCA	NA	NA	NA
Ca-II-SNP885	164.28	Ca-*desi*-LG(Chr)5	10563588	ATGAGATTATGTCTAATTGTCATCCCTACTTTTGC GCTATTTGTTGTTGTCTACTTCTTACATTTAGGGG CGGAAATATGTCCATTGATTTATAGGAGTT	C	T	TGTGAGGCTGTCTATACATATGAATTTTAATGTT TAAAACATTAAATAAAAACAATTTATATAAAAAA CTGAAAAGCTACATTAATAAGCTGACTTTGCTA TTTCACCAGAAGAGTAAGAGACAGAGCCTCACA TCTTTTCAATTTTACAATTTTAACCACATCAGTA GTTTAATTTTATCAAAATGATTCAAGCACTTGAT CGTGTGGTTTCCTGAAAACGCTGGAGTGATTCT [illegible]	NA	NA	121
Ca-II-SSR291	165.27	Ca-*desi*-LG(Chr)5	10581198	AAAAAGTCTATTAGGTCTGATAGGTCG	(AT)9	(AT)10	GGAACGACTACTTAGTGATTGCCT	58.87	140.00	NA
Ca-II-SSR292	166.26	Ca-*desi*-LG(Chr)5	10615669	TCTTGTAAGCTTTAGCCGGA	(AT)11	(AT)12	CTCCCACTCAAAAGTTTGTTCA	57.81	183.00	172
Ca-II-SNP886	167.25	Ca-*desi*-LG(Chr)5	10689098	CTAATTCATCATTTTCTTCTTTGAACATTGCCTCCA CTTTATATTAGAGTTAGACATTACATTACATATTAT CATTTCAAGATGGCTTTTCAAGGGTCTC	T	G	GTCCTATCGTTCCTCCACTTTTTGCAATAATGT AATCAAAACCCCCCCGCTGGCTGTTACTGTAAA GAAACTCAGGTTTTCACAAGCTCTAGTTTCCTT CCTTGGAGGTCATGTTCCTCCATTTATCCTTGA GATCAACAGGAGTGCGAGTATTATCAAACACAC GACAACCAAATTCTAAAATTTTTCTCCAAGGGA TATTCTGATTTCCCGTTGAGAACTTCAACACTC [illegible]	NA	NA	NA
Ca-II-SNP887	168.24	Ca-*desi*-LG(Chr)5	10700325	GAAGGGTTCCAGCACCAAGAACAAGAAGCCACGA TGGATATTTCCGTGCAGGAATTTCCCTTGGAATCT GATTGGTCCGTACAGGATATTCAATTGGCTC	T	A	CTGGCGCCATACCCATTTCCTTGTATTTTGAGC TGTTCTTCCTCTGGTTCTGTATTTCAGTACCTC TGACCCTCATTGGAGGATTTATGGGAACAAAAG C	NA	NA	183
Ca-II-SNP888	169.23	Ca-*desi*-LG(Chr)5	10814276	GAGTATCAAAATTTAGGTAGAGTCATTTCTTCCTG AAATATGCCAACCCTCAGCAACTTAATTCTCTGTT AATTTTTTCCTATTTCATTCAAAGGTCTCC	G	A	TTCATATGTATCAAGAAAGTACCCCAAATTTTCA ACAACAAAAAATCATGTGTTATCATCATGTTTAT AAACATTAACTCTTAAGTATATTGTGATGTTT	NA	NA	NA
Ca-II-SNP889	170.22	Ca-*desi*-LG(Chr)5	10901334	CCATTCTTTTCAGCATCTTGTATTCACCATATCAA GGTTTATTCTTTATTTTATTCACCATATGTAGTTTA GCATTTTACAAAAGTTGGTAATGTGATAG	T	C	ATCTTGGTAAACTTGTTGACCCTAGGCTTGGTG AAAATTACCCTCTTGACTCAGTATTTAAGATGTC ACAGCTTGCCAAAGCTTGTACACATGAAAATCC TCAACTTAGGCCAAGCATGAGATCAATAGTAGT TGCCTTAATGACACTTTCATCTGCAGCTGAGGA TTGGGATG	NA	NA	242

Marker IDs	Genetic positions (cM)	Chromosomes	Physical positions (bp)	Flanking sequences/Forward primers	ICC 12968	ICC4958	Flanking sequences/Reverse primers	Annealing temperature (0C)	Amplified Product size (bp)	
Ca-II-SNP890	171.21	Ca-*desi*-LG(Chr)5	11006689	CTGGACAGCAGTGTAATATCAGTCTCCAGATTGATAAATATTCTCCAGCTTGTTACACTTGATCTTCAGCAGCAAATATTTTTCTCCAGAATGACCAGAC	T	C	GTCCAAAACAGCTTGGTGAATGATTATACATTTCTATAGAAATAAGGAGATCAATGCTGAAATTCTGCATAAATGAAGATTGATAATCAATCTGGAGACT	NA	NA	NA
Ca-II-SNP891	172.20	Ca-*desi*-LG(Chr)5	11029004	CACTTGCCACCACGAAACCCACATGTGCAGTTCTCTCTGGCTACCGTTTTCGAGCGCCGACCAGATCAGGTGCCCCTCTGCCACATTCTGAGGTGTTTAC	C	T	GAAACTGAAGGCACCAGAAAAAGCAGCAACCGATACAGTGTCGGACATGACAATCTATACGAAGGGAAGATGAAAGGTTGCGGAAAAATCACCAAAAGAT	NA	NA	NA
Ca-II-SNP892	173.19	Ca-*desi*-LG(Chr)5	11107994	GGCCAGACCAGGCTTTGAAAGGCCTGAGCCTGGCCTACGATGAATTTTTAAGGCCTAAGCCTGGCCTACGGCCTACAATAGGCTTTTTTTTCGGCCTGAG	T	C	CATATTTTTTTTTAAATTATTTTGTTAGAAAAATTATTTTTAATGTGAATAAGGCTTTTAAATAGGCTTACAGGTCAGGCCATGATAGGCCGTAGGCCAG	NA	NA	171
Ca-II-SNP893	174.18	Ca-*desi*-LG(Chr)5	11131133	CACTTGCCACCACGAAACCCACATGTGCAGTTCTCTCTGGCTACCGTTTTCGAGCGCCGACCAGATCAGGTGCCCCTCTGCCACATTCTGAGGTGTTTAC	T	C	GTGGACCTAGTTGGCGGTTATTATGATGCTGGTGACAACGTTAAGTTTGGGTTACCAATGGCATTTACAACTACATTATTAGCATGGAGTGTCCTTGAATTTGGAAGCTCAATGCAAGACCAAATTGAAAATGCTAGAACTGCCATCCGGTGGAGCACGGATTACCTTCTTAAGGCGGCCGTCACAACCCCCGACACATTATATGTTCAAGTTGGAGAGGCTAACATG[illegible]	NA	NA	102
Ca-II-SNP894	175.17	Ca-*desi*-LG(Chr)5	11184670	TATTGGTAATTAATCTAATTTGAAATTTTCATTTAAGGTTTTTGTATGAGAAGTTGGTGTGCACGCACAAATTGTCAAATAGATATTCATTCTTGTCTCC	T	G	GTGCAAAGAACAATTTATCATTCTCTATATTTCCTAATAAGATATCTACAATGTATTTCTTTACATTCACTAGATCAAGTTTACGCATCGAAAGCTTATG	NA	NA	109
Ca-II-SSR293	176.16	Ca-*desi*-LG(Chr)5	11185022	CCATTGCACAACAATTACAACA	(AT)10	(AT)9	GAGCTCGAAAAACTTGTAAAGCA	59.39	168.00	149
Ca-II-SNP895	177.15	Ca-*desi*-LG(Chr)5	11199544	CGCAGCGGTCTTTGCTCCAAATATATGACACCTTCACAAATTAATTTACTTCTCACTTAGTGTCAAAACTTTTTAGTTTTATTTTTTATAGATTGACGTA	C	T	TTACACAGTAAAGGGAATGAAAGTTCTTAATACCATATATTATTAGGAACTCTACTATCTCATTATTATTGTAATAACGATCACACTAGTAATAGATTTT	NA	NA	144
Ca-II-SNP896	179.13	Ca-*desi*-LG(Chr)5	11366972	AATTTTACATTGCGTTTTGTTTTAGTGCAGTGCTGAATTTTGGTGAGAAATCAACATGACATATATAGAGTATTTCAGTCATATCTGGAGTTGTAGATGT	T	C	ATTTTTCTCCTCTTTAGAGTCTGCATTTGGTTCCGGTGTCTTCATGAAAGTTGTAGCTATGGATCTTAGCTTTGATGTGCACTTGGTTTGACTCCAATTG	NA	NA	142

Marker IDs	Genetic positions (cM)	Chromosomes	Physical positions (bp)	Flanking sequences/Forward primers	ICC 12968	ICC4958	Flanking sequences/Reverse primers	Annealing temperature (0C)	Amplified Product size (bp)	
Ca-II-SNP897	180.12	Ca-*desi*-LG(Chr)5	11452105	CCATTCTTTTCAGCATCTTGTATTCACCATATCAAGGTTTATTCTTTATTTTATTCACCATATGTAGTTTAGCATTTTACAAAAGTTGGTAATGTGATAG	G	T	AGAAAAAAATGTGCGAGATTGCAGTCATTTAGGCATTTAATCAAACAACTGATATACTCATGAAGTTCAAATTTCAATTAAAGTAAATAAAAACCTATTA	NA	NA	157
Ca-II-SNP898	181.11	Ca-*desi*-LG(Chr)5	11514809	GAATTGTACTGTACTCAATGGTCAATAAGAAATATGTTACCTGATGTTTTAGAGTAGCTAAGCTCTTGGTATGCATTGGAGATTGTGGAATCACACCATT	G	T	ACTAATACTTTCTACTCCATCTAGTCAATATTTCATTTCAAATTCTTGTCGATTTTACTTTCCAGACCATATTGTGTGGGCTAATTGGAATCCCTCCTTC	NA	NA	163
Ca-II-SNP899	182.10	Ca-*desi*-LG(Chr)5	11563778	CACTTGCCACCACGAAACCCACATGTGCAGTTCTCTCTGGCTACCGTTTTCGAGCGCCGACCAGATCAGGTGCCCCTCTGCCACATTCTGAGGTGTTTAC	C	T	CCCGGTTCAGGGTGGTCTTCAATGTTGTAGTATTAAATTAACAAAGAGATGGATGGAGTGTGAAAGAGAAGTTTGAGAGTCCAAATCGACTGCCTTGGTGCGTTATGTGTGTGTTTGTGTGCGACACGTCATGAAAGCTTTAGGATTTATATATGATGTAACATCGAGGAAGATATGATAGTTG	NA	NA	146
Ca-II-SNP900	184.08	Ca-*desi*-LG(Chr)5	11567462	AAGATAATGTTTAAAATCAAGAATCCACCTTGGAAGCTCTTTATATGATTCCTCCTAATTGTCGTATATTCTCTCAATGGCCTTTTGTTTTGCAATCCAT	A	G	AGCATCTTACAAATGGTAACAACAGATCCCACAATATCAGTTTCAGTATTGATTGCACATATACGATCTCGGTACACATATACCACTACTTATAGAAAAA	NA	NA	119
Ca-II-SNP901	185.07	Ca-*desi*-LG(Chr)5	11592149	GAACAATTTTAAGGGTGAGTCAACTTTTATTTTCAGTCCGTCCAAGAGTTTATGAATGCATAAAAGTTCACAGATTCCTTGAGCCATTGCTCTGAATTCA	T	G	GATAGAAAATCCACCACTGGATATTGTGCCTATGTTTGAGGAAATTTAGTTACTTGGAGAAGTAAGAAACAAGGGGTTGTTGCCCGAAGCAGTGCAGAAG	NA	NA	160
Ca-II-SNP902	186.06	Ca-*desi*-LG(Chr)5	11629355	CACTTGCCACCACGAAACCCACATGTGCAGTTCTCTCTGGCTACCGTTTTCGAGCGCCGACCAGATCAGGTGCCCCTCTGCCACATTCTGAGGTGTTTAC	C	T	ATTTTACTCGAAGAAATAGAGAAAACATATATGAGACGTGTATGAGAAAATACTACATAATTTCTCATTTCTGCTGTCAAACTAGCTTCTTTGCTTAACTCTTAGTAGTCTCCTCTGAACATCGAAACAGGTAGCCCTGGTTTTCCGGACTCTAAAACCCTTCGTTTTCCCGCCATACACCTCAACGGCCCAAACCCTAGCTTCCACGAATACACCGTGCAACCTCCATTAT[illegible]	NA	NA	208
Ca-II-SNP903	187.05	Ca-*desi*-LG(Chr)5	11842074	GAAATAGAGACTGAGTCCTATTAGAAGTCAAGAAACTGGGAGTAAGTTGTTTAATTCATCGTTGTTCATTAATTGAGGATGGTTATTGTAACTTGTTTTT	A	G	TCAATGTTAATGTCGAAATTTTCAAATAAAAATCTTAATTTAATATTTTTAATCTTAATTAATGAAAATAAAAAATTAGTATTTTTTAAATAAATAAAAA	NA	NA	133
Ca-II-SNP904	188.04	Ca-*desi*-LG(Chr)5	11915491	AAAACCAATATGTAGTTACGTCCAATCACTTCCATTTTGTTAAATTATATTTAATATCTATGTGACTGTGTCTATGTTGTGTTCGATGTCTACGTCAATG	C	T	GTAGTATAATTTAATTAAGGCAAAAAAATTCATGAAGTTCCTCAGCAATGGGTCATGTGCAGAAAGCATCTCTTGCTTTGAAACTCACCAACATAACTAA	NA	NA	105

Marker IDs	Genetic positions (cM)	Chromosomes	Physical positions (bp)	Flanking sequences/Forward primers	ICC 12968	ICC4958	Flanking sequences/Reverse primers	Annealing temperature (0C)	Amplified Product size (bp)	
Ca-II-SNP905	189.03	Ca-*desi*-LG(Chr)5	11926984	TGTGGTTGCTAAAGTAGTGTTTAAAAAACAATTAAAAAAGTGTCACTGGTGGGTGTAATTGAACGTATTTTGCTGTTATTCTTTTTCTTTTTTAAGTGAC	T	C	GCGTCAGTTGAAAAGTTAGTGGCCAATTAAAAAATAAAAGAATTAATTAAAAATAAAATACACTACACCACTACTTACGTTTCACCGTTGAGAATTTAGT	NA	NA	184
Ca-II-SNP906	190.02	Ca-*desi*-LG(Chr)5	11993105	TATCCCTTGGTTATTTAATCTAACCGGCATCCACATTGATCATACCACATGTACTCCAGAAAACTATATTCGCTAAGTCCCAAAGGATGCAGGGTTTTTC	A	G	CCTTCAAACTTATGTTTCTCATTTTAAAGTCAGTAATCAATCACCCTGAAAAAGGACGCATTTTATTAGGTACAACCTGGTTGACTACTCTCTCATCAAC	NA	NA	143
Ca-II-SNP907	191.01	Ca-*desi*-LG(Chr)5	12035418	GAGGTTTATCATATCCTCTCAATTTCTTGATATAGGCAAAAAATCATATCAAGCTCGCATTCGAGTCTTTCAATTCTCGTGTATATCATCAGAGATTTTA	C	T	TGGAAAAATACTCGCGTTCGCTACGCCTAGCAGCAATCATATCAACATTTTTGTTAATATCTGCTTGTGATCTATTGACAACACCAACCCAGGGATACTTTAACCTATAAGCTCTCCCCTCCAGCATTTCAACAGCATCAGTACCC	NA	NA	140
Ca-II-SNP908	192.00	Ca-*desi*-LG(Chr)5	12052770	AAGTTTATACTATTAATATAAGTCTTATGAGTTTGTTTGCAGATTTTTTTAATTATATTAACTTTATTTTTACCCCATCAGATGTTTATTTTAATATCTG	A	G	TAAATTTTTTTAGTCCGATTGCAAGGTCTAAATATAAATAGTAAGTATGAATCATAGTTCTAATACTTTAGAAATTTATTTATTTTCTATATTTTGTGCT	NA	NA	156
Ca-II-SNP909	192.99	Ca-*desi*-LG(Chr)5	12117536	GAGGTTTATCATATCCTCTCAATTTCTTGATATAGGCAAAAAATCATATCAAGCTCGCATTCGAGTCTTTCAATTCTCGTGTATATCATCAGAGATTTTA	T	C	ATTGTGAAACTCAATGGATATGTATAAGAATTGACAGACCAAAATGTGAGTTTATGGTTGATGACCCTAAAGTAGATTGCATTATGAAATTATTCTTACT	NA	NA	146
Ca-II-SNP910	193.98	Ca-*desi*-LG(Chr)5	12150221	CAAAAGAGAGAATGCATGATGTTTGATCACGGAGTTCAGTTAATTGTGTCAAAGTCTCCGCCTGTAGAGTGGCTGCAATACCTTTGTATAATGATTCAAG	A	T	TATGGCTCATAGTAAGTGATAAATGGATAAGTCGCTTGAGCTTAAATGTTGTAATCTGGACCGTAGTATTTAAATACAAGTTGTACACTGTAAATCCTAA	NA	NA	138
Ca-II-SNP911	194.97	Ca-*desi*-LG(Chr)5	12171539	TTCTCTTTTGTTTCTCTTATACCAAATAATACTTCAAATCTATTTTTTTTTTCTTTCATATTTTTCTATCTTATCACACTCTCTCACTAATTTCTCTCTT	C	T	CCAATATGTGGGCCGGAGCCAATAATTAGAGGGTTTGTCAAATAAATGGACAACACTATTCTTTTCTTTTCAAGTGTGATCGGTTGTAAAGAGAAATTGG	NA	NA	151
Ca-II-SSR294	195.96	Ca-*desi*-LG(Chr)5	12287423	CAATATAATTGTGGAGGATGATAGTTT	(AT)20	(AT)15	GTCACCTATGCCAAATCGGA	58.06	112.00	177

Marker IDs	Genetic positions (cM)	Chromosomes	Physical positions (bp)	Flanking sequences/Forward primers	ICC 12968	ICC4958	Flanking sequences/Reverse primers	Annealing temperature (0C)	Amplified Product size (bp)	
Ca-II-SNP912	196.95	Ca-*desi*-LG(Chr)5	12294571	TCTTTATATTACTTTATTCTCTTCTACTTTCCTTCTCTTCTCTGCCACCTGACTTTATCTCAAGACGAAAATCTGAGTAATATTCTGAACTATTCATGAA	A	C	TCTTCTTCTTCTATATTTTGTCAAACTCTCATTTTGTAGTTGCATCATAGCTACTTATGTATGTGGGAGCTATTAATATTTTAATAATATTTGTTGTGAT	NA	NA	157
Ca-II-SNP913	198.93	Ca-*desi*-LG(Chr)5	12433033	CCACCAATGCCTAGACCTCTTCCTGTTTTGATGGATGGGATTACATTACCATGGTTGCAATGGGAGACAAACAATAGCCCTTCTTCTCCTATGACAAACC	C	A	AAAATAGCACAGAAAATGATAATGATGATGGAAGGGGTAAAACTATGCGCACATTAGAACAAATTGTAGACACATGTTGCATAACATCCCGTATGTATCAAAATGATGTAGATTTTTTTAACATTGATCCTATGTTCGAAGAGGTGTTGGGTACAGG	NA	NA	126
Ca-II-SNP914	199.92	Ca-*desi*-LG(Chr)5	12466514	GTTGAAATCCTATTCAAATTCAAACTAAGGATTCTTGTGGTTCTGGAGGATCCAATTACAGGAGAACCGGGAACGAAGAGCTTCTCCCCCCCTTTTTACG	T	C	CACATTAGTTATTTGCTCTCAGCTTGAACAGTTAAAGGTCGGAGAAGTGCAATTACTCATTCTTAACTTAAAACCCACATTCTTAAGACTAAAGAGTCTG	NA	NA	210
Ca-II-SSR295	200.91	Ca-*desi*-LG(Chr)5	12483205	TGGAACAGGTTTTCTAGCTGC	(CA)8	(CA)9	AACGATTGGTGCTCCAGACT	59.51	124.00	147
Ca-II-SNP915	201.90	Ca-*desi*-LG(Chr)5	12510260	CCACCAATGCCTAGACCTCTTCCTGTTTTGATGGATGGGATTACATTACCATGGTTGCAATGGGAGACAAACAATAGCCCTTCTTCTCCTATGACAAACC	A	T	ACAAATGCCTTTCCTTTGTAAACAATTAAATAGTTCAGTGATAACCTGAGTGGAACTTCCAGTTGCACTATTCGGAGTCGATGTCAAAAGTGTCTTATTC	NA	NA	101
Ca-II-SNP916	202.89	Ca-*desi*-LG(Chr)5	12520180	AGGAGATGGAAAATTGGAAATTTTCTTTCCTTTCCTTTCTTTTTCTTTCCTTTGTTTTTCCTTTCTTCCTTTTTCCTTCATTCCTTTATATACTATTTTC	C	T	TTTAACAAATATCTAGATTTTGAGATATTTGTTAAGTTATATTTATATATGTTTGTTTGGAGGGAAAAAGAAAGGAAAACTAGAAGGAAGGAAAAGGAAA	NA	NA	217
Ca-II-SSR296	204.87	Ca-*desi*-LG(Chr)5	12567943	AAATAGTCGTAGAGAAAGGATGCAA	(AT)10	(AT)11	TTCTAAAATTGAGGAATATGACAAAA	59.73	208.00	148
Ca-II-SNP917	205.86	Ca-*desi*-LG(Chr)5	12619537	TTTGTATCTATCAGCTCTGCACCTACTACGGGACCCACGATGTGGTGTTGCAGAAGAGCCAACTCAACCATCACACCGACAAAGACGTAATAGACCTCGC	T	C	TGTCTTGGTGGTTCAAATTGTTGTTGTGTTGGGGTGAAAGAAAATGAATGACGATGTGGTTATGGCGCTTCATTTTCAATAGAAACAGGGTGAGTCTGTA	NA	NA	154

Marker IDs	Genetic positions (cM)	Chromosomes	Physical positions (bp)	Flanking sequences/Forward primers	ICC 12968	ICC4958	Flanking sequences/Reverse primers	Annealing temperature (0C)	Amplified Product size (bp)	
Ca-II-SNP918	207.84	Ca-*desi*-LG(Chr)5	12688787	TCTTAGATTTTAATTTTATTTTTTTAAATAAATTTTTATTTTTATTTTTATTTTTTTTGTTATTTTCTTAGTTGTTTACTCTTGTTGTTCTTGGCTCATA	A	C	CTATTGTACTTTATTAACCAATAATGTTTACATTATTAACTAGAAAAAGAAATACAAAAAAAAAAAACCAGAAAACTGTGTATAAGCCAAGAAAACCGTT	NA	NA	163
Ca-II-SSR297	208.83	Ca-*desi*-LG(Chr)5	12944505	CGGAGGTGGTTCTTGATGAT	(GTT)5	(GTT)6	GTCATGATCCTCCTCTTCGC	59.93	168.00	209
Ca-II-SNP919	209.82	Ca-*desi*-LG(Chr)5	12950426	ACTGAAGGGGTAAGAACGAAGCATGAGTCTCTTTTATTTTCTCCAAGTAGCCGAATGTCTTTCTAACAATAATGTCTCGACCAGGATCAAGGTTGTCCAA	G	A	AAAACTAACTAAACCCTCACTCCCAATTTTCATTCATCACCAAAACTCACAAACAAAATAAAAACAATTTTTTTCTTCAACTGAAATAAAACAAAGAAGAAAAAAATCCGAACAACAATGGCTCCGAACTTGGAATGTCGCATGTACGAGGCAAGGTACCCAGAGGTAGACATGGCAGTAATGATACAAGTGAAGAACATCGCCGACATGGGTGCTTACGTGTCACTTCTCG[illegible]	NA	NA	175
Ca-II-SNP920	211.98	Ca-*desi*-LG(Chr)5	13042726	CGTCTTATATTTGGTATTTATTCTCTCTCTTTTAGTATTCTTAAAAGTCTTTAAACTATGTTTGGATTTGGTCACCACCTTTAAATTGAGTTTTCTAGCA	A	C	TGTCATTTCGAAACTTTTGAATATTCAGAAAATTTGCCAAGTTAAAAGATTAAAATTGTATTTTTATATGGATTGGGGTGGCAGTTTTATGTAGGGGTGC	NA	NA	189
Ca-II-SNP921	213.15	Ca-*desi*-LG(Chr)5	13086919	ACTGAAGGGGTAAGAACGAAGCATGAGTCTCTTTTATTTTCTCCAAGTAGCCGAATGTCTTTCTAACAATAATGTCTCGACCAGGATCAAGGTTGTCCAA	C	A	CATCTAATGTGTGCCTAAGGTTGTTTCGAAACAGAACAAAGGATCCAAGAACACATAATATGCCATCATGTGATGAGGTTGCTGCTTTAATAGTTGGTGA	NA	NA	145
Ca-II-SNP922	214.32	Ca-*desi*-LG(Chr)5	13163827	TGCTCTGTAGTACGCAACATTTTCAGATGTTTGTTTTGCAGTCCATCAAACATCTGTGACATTGAAGATATAGACTCATCCATATCTCGTTTACAAATGT	T	C	ATTTGTTATACTTATTATATTATTATTACGATAATATTCTTATTTAATTATTTGTATTTTTTTAAATTATTTACAGACATGGATTTATGAGCATTTCCCT	NA	NA	162
Ca-II-SNP923	215.49	Ca-*desi*-LG(Chr)5	13175432	ACTGAAGGGGTAAGAACGAAGCATGAGTCTCTTTTATTTTCTCCAAGTAGCCGAATGTCTTTCTAACAATAATGTCTCGACCAGGATCAAGGTTGTCCAA	C	T	TTGCAAGATGGTAGAAATGTCCTGTGAGGAACATGATAAAGCAGCTGCGAAGAGCCAATTTATCACACACACAATAGGCAGGGCATTGGCAGAAATGGATGTCAAACCCACCCCTATTGACACTAAGGGCTTTCAGGCACTTG	NA	NA	112
Ca-II-SNP924	216.66	Ca-*desi*-LG(Chr)5	13648236	CATGTTCAATTCCATTCTTGACACAAAATCCTTGAATTCATAGGATGTATATTCTCCTCCTCCATCTATCCTTAGCACGTTGACAACTTCTCCACTTTAA	T	C	TAAGTATTTCATAACCTTTGTTGATGAATTAAGTAGAATGTTATGGATTTATTTGATTAAAACCAAAGATGAGGCATTGGACAAATTCAAGAACTTCAAG	NA	NA	160

Marker IDs	Genetic positions (cM)	Chromosomes	Physical positions (bp)	Flanking sequences/Forward primers	ICC 12968	ICC4958	Flanking sequences/Reverse primers	Annealing temperature (0C)	Amplified Product size (bp)	
Ca-II-SNP925	217.83	Ca-*desi*-LG(Chr)5	13652905	TTGATGCAAATGGATTCACCTTCCAAAACCATCTCAGATTTCATGGCTTGGTAGATTTTCTCCCATGCTCTTTGGAAATCTATCCAAGACTCATTAGAGC	G	A	GTGTTCTGTGTTTGTTTGTATTAATTAATCTTGATCCTGTTTGCATTATTGGTACTTGAAAGCCTGCTTCGGCGTAAATAAATCATTGTTCTTCTTCCTTTTTCTGTTTTCCCCCTATGCAGTAATCACTCGAGTATCATGCTTTTGAACC	NA	NA	139
Ca-II-SSR298	219.00	Ca-*desi*-LG(Chr)5	13691148	AATTGGTTGCTAAGCCGTGT	(TAA)9	(TAA)10	TGGGGAGAAATAAAATGGGA	59.64	182.00	192
Ca-II-SNP926	220.17	Ca-*desi*-LG(Chr)5	13702262	GGTGGTTGTTTCTAGATTAACTTGTTCCTACAATTATAACTTCCTCGGTATTTATTGAACCACCAAGTGTTGCTTTTATCATCAGTTTGTTGAGCATAGT	G	T	AAACGTCTACATATAGCACACATAACCTAAAATGAATACACAAAAACTTCAAGTGTTATAAATATAATTACATCGAGTAAATTATTTGTTTGAGAAAATC	NA	NA	131
Ca-II-SNP927	221.34	Ca-*desi*-LG(Chr)5	13715122	ATTTAATGGTTGGATATATATTTTCTTGGTATAAATAGTTAGAAAGTCTACATCTTGGTCATGCTCCACCACCAACTTGTTTTCACCTTCTCCCTTGAAT	A	C	AGAAGGATTAATTTTTGGTTATGCTTATGAATTAATTGTCTCACAATCACAACCAACTCTATAGGTATTTTTCCTAAAGTAAAATGGTAGTGAGAGAAAA	NA	NA	172
Ca-II-SNP928	222.51	Ca-*desi*-LG(Chr)5	13739071	CCTTGTTATACACCTTGAAAAAGATAGTGGCTGTTGTATCTGGTATTTACTGAATTTTTTATGTTTTCCTAAATTAAAAGTCCTTTTCTCTTTTGCAGGC	C	T	GGGAAAAGAGCTCCTGTGAATAAGGTACAACTATTGGGAAAAAAGAAGTAAAAAAAAAAAGACCAAACATACCAAGTAGCTTCAGAATGTCAGTAAATGT	NA	NA	134
Ca-II-SNP929	224.85	Ca-*desi*-LG(Chr)5	13768754	GAATTGTTGTGTTGCCTATGAATCCATGTTTATGTTGTGTTGAACAAATCTTTAAATTATCATCTTATTATCATGATGTTTTATGTATGGTTAAAATCAT	A	G	AAAGATAAAATGTTTATGCTCCCCCTCAATCATATGTGTAATAATTTTTAAAACTAAAAATCATTACTCCCCCTAAATGTATGCCAAAGTGTTGAAATAA	NA	NA	198
Ca-II-SNP930	226.02	Ca-*desi*-LG(Chr)5	13790950	CTCAAATTTGGGTGATCATGAGTTGAGTTGTATAATCTCCATTTTAGATGATTATGTAGAGACTTATGGTATCCCCAACCATGTCCTAGTGAGAGGAGGG	C	T	AACCCAACCTAATATGTGTATCACCCTACAATATTATCTCACATCTAAGAGTTACAAGGATGATAACACTCACATCTCACAACAACAAATAATCACCCAC	NA	NA	126
Ca-II-SNP931	227.19	Ca-*desi*-LG(Chr)5	13824239	AATCTTAATTTATGACAGATTTTATTCAACAGCTCTTTCATGACAGTTGGTCCCTTTCCAACGGCCACATGAGCTGTCCTAAGTCTTCTTGGAGTCTATA	G	A	AACTATAAGAAAGATTTTGAGACACTTGAGTATGATCGAAAAGATTGATCTTTTGCTTGGTGCTAATTGTTGTGTTTTGTTCCTCAACTTGCAGCTGCAT	NA	NA	138

Marker IDs	Genetic positions (cM)	Chromosomes	Physical positions (bp)	Flanking sequences/Forward primers	ICC 12968	ICC4958	Flanking sequences/Reverse primers	Annealing temperature (0C)	Amplified Product size (bp)	
Ca-II-SNP932	228.36	Ca-*desi*-LG(Chr)5	13913861	ATTTATATTTATTTTTTGTCTTTCATTTCAATTTTCTCACATGTTCCATCTAGTGAAACAACAAAGGGGGCGTATTGATACTCAATCAATCTTTACTTTG	T	A	AGAGGCATCAATTTTTGGGAATTTGAAGAGTTTAATGAAAGCCCAAAATAAAATTAAAAATTAAAATGAAAACAAAATAAGTTTATCATGTCTTGTAACC	NA	NA	204
Ca-II-SNP933	229.53	Ca-*desi*-LG(Chr)5	14047868	TTTACAATTATGGTTTATTTTTTATAACTAGCATTTTCATCTTGTTAAGCTTTCTATGATCAGGAGCATCTTTTTTGTTTGTTGGCTCAGAGCAAGAAGC	C	A	AGGCAGAAGCTATTGAAAGTAAACACTTTGACCAAAAACTTAAAAAAACGTGCATAATACAATCAACAAAATACTCATAGAACTCTAACCTTACATTATA	NA	NA	201
Ca-II-SNP934	230.70	Ca-*desi*-LG(Chr)5	14048205	TTTTTTAAAACTCGAGTTTGTTGTTTATGAACCAATGAATAATTTCTAATTCTTATGTTTGTATGGTTATGTGTATATGATATTGTGTGTTTGTATGGTA	C	T	CATTTTCAAACAGAAAATAAAATATCAACATACAGTTATTTAGAAGATAACTTTCTTGTTTGGCACCAGTATGCACTTTATGAGATACTGTGATATATGC	NA	NA	205
Ca-II-SNP935	231.87	Ca-*desi*-LG(Chr)5	14063987	TTGATGCAAATGGATTCACCTTCCAAAACCATCTCAGATTTCATGGCTTGGTAGATTTTCTCCCATGCTCTTTGGAAATCTATCCAAGACTCATTAGAGC	A	C	GTCCTGATAGCCTGGAGAACTCCCTAAAAGTCATGGAAATTTTCTTCCCTTTAACTTCAGACTCAAATCCAAAGTCTGTGAGTGTGAAGTTTGAGTAGAA	NA	NA	149
Ca-II-SNP936	233.04	Ca-*desi*-LG(Chr)5	14087402	GTTTCAGTGATTTGGGATGTTATTAGGTTAGATCTTCAAGTCCATTTTAAAAAGATGCACTGTCAGTGTAAATAGTTTTACACAGTCGGCAAATCATGAC	C	T	ATAAATGAATTTAATTTATATACACTGTCAATTATAACCGTTACATCATTAGCAATGTTTGATTTCTGACAAAACTACTTATAAAGTCACCCACATGATT	NA	NA	143
Ca-II-SNP937	234.21	Ca-*desi*-LG(Chr)5	14100567	ACAAATGTGACCAATGGTCTTCCAGGAAATCCACGTTGTTGGACTTGCTCTTCAACTGTCGGCTCCTCAGCTTTCTCATAGTACATTTGATCAGACTCCT	G	A	ACCCATTGCATCTCATAGACAAATGAATGGAGCTAACCATCCACCTCATCAACATCGTCGTAGGCGTAGTTTCAGGTCTCACGAACTTGATGAAGATGTT	NA	NA	147
Ca-II-SNP938	235.38	Ca-*desi*-LG(Chr)5	14165429	AAATCAGTATTAGAAAAATATCGATTGCGCATTCGTTAACCGTTGGATCGGGTTGATTTTTGGACAGCAAGTTCGCAACATTCAGATCCACGTTTTCAAC	A	G	TCTAGAATTATGCTTCACAAATTATTGTCTCACTCATTTCACACCTGCTGCTGTGCACGCACGGTTTCTCCCTCTCTGAATGTCATTTCGCTGATCAGAC	NA	NA	157
Ca-II-SNP939	238.89	Ca-*desi*-LG(Chr)5	14222770	GCACGCACGCACGCACGCACGCACGCACGCACGCACGCACGCACGCACGCACGCACGCACGCACGCACGCACGCACCCACGCACGCACGCACGCACGCACGCAC	G	A	CCTAATTTGCACTTTAAGTGCTTGAACATTACACTCCTAATGTGTGTGTGTGTGCGTGCGTGGGTGCGTGGGTGTGTGTGCGTGTGTGCGTGCGTGCGTG	NA	NA	171

Marker IDs	Genetic positions (cM)	Chromosomes	Physical positions (bp)	Flanking sequences/Forward primers	ICC 12968	ICC4958	Flanking sequences/Reverse primers	Annealing temperature (0C)	Amplified Product size (bp)	
Ca-II-SNP940	240.06	Ca-*desi*-LG(Chr)5	14259428	TGAGTGAAATCAATGTTTTATGATTTACTTGTTATGTTGAGTTTAAGTCATTAGATTACTCATGTCTAATGGCTTAAAGTATTTGAAAGATTTGTGGCTA	G	A	TGCCAGTATGCTAAACATAGAAAATCCTCTCGGCAAACTATACTTCAATTTAATATAATACTATTTCTTCGTAGGCTATATTCAAATACCTCTTCTACCT	NA	NA	181
Ca-II-SNP941	241.23	Ca-*desi*-LG(Chr)5	14270809	CAAATCCATGATATCAGGTATTTTTAATTTGAGAAAGTGATTTGAGGCTCTAATAAATTGTCATTTGTGATCCTCTTGAGTTTGATTATTCAGTTGCTCT	C	G	GAAGTTTTGAATTTCTATCCCGGGTCTTTTGTAAGTCTTTAATAGAAAAAAAATGTTTGAAAACTTATTAGAGCCTCAAATCACTTGCAATAATTAATTA	NA	NA	192
Ca-II-SNP942	242.40	Ca-*desi*-LG(Chr)5	14297283	GATTTTGTTAAATCAGTCTCAGAAAAACATCGATTGCGCATTCGTTAACCGTTGGATCGGGCTAATTTTTGGACAGCAGGTTCGCAACATTTAGGTCTTC	G	A	ACACAATCACTGAGACAAAGTAAATTAGGGATAACCTGGTGCTGTGCGAGCACGATTTCTCCCTCTCCAAATGTCGTTTCGTCGATCCGACCGTTGACGA	NA	NA	174
Ca-II-SNP943	243.57	Ca-*desi*-LG(Chr)5	14322733	TGGCCCTCTCCTCTCAATCCTGCAACGCATCGTCACGTATCAATTAGGGAGCTTACATCACGTTGATAGCCCTCTCCTTCTACGGATGACATTTCTCATA	T	G	TGGGAAATCGATGTTATTGTTATTAAATCGTTAACTGCGTTAGGTGCACCTAGTTACCTCGTGTTGATTAAGGAGAGTTACCCGTTAGATGGAGCCGCCC	NA	NA	141
Ca-II-SNP944	244.74	Ca-*desi*-LG(Chr)5	14412697	AATAATGCAAACATGCTATATGTAAGACCCATAATTTTAAAGTGTATTTTATGTATTTTTGTGTATTTTGGATTTTGGCTCAGAGGCTTTTTAGCCAAAG	A	T	GTAAAAGATTCACCGCAGTTAATTTAATTTATTTATCGACGAGTAATTCTAATCGGCATCAAAATATCTTTTTGATCATATAAACTCCAAAATATTATTA	NA	NA	145
Ca-II-SNP945	245.71	Ca-*desi*-LG(Chr)5	14425204	AAAACTTGTCGCTCATGCAGGCGATCCAGAACCTACCAATCATGAAGCTGATGCCACTCATGCAGCTGATGGCGCTGATCCCGCCGATGAAGCAGTTGAC	G	A	TTTGTATTGCTCGATGCTGCAATAAAAAATAAATTAAATATGAATAGTTAAAATAAATTAAATATGACTAAATAAAATATGACTAATTAATATACCGTTA	NA	NA	125
Ca-II-SNP946	246.68	Ca-*desi*-LG(Chr)5	14463140	TATATATATTACCTTACAATGTGTATATTTGTAGTGTGTACATATATAAAATATTACAATCCTTTTCTTGTCCTTTTTATAACATTTTTTTCACCTTTTT	A	T	GGCCCAATGTCGATCCTACTAAATCCATCCTTGTATATAAAACCCAAAAAAAAAAAGTGATATAATTAAATGAAAGTTCGTAAAAAATAATTAACACAAT	NA	NA	100
Ca-II-SNP947	247.65	Ca-*desi*-LG(Chr)5	14497965	TTGATTTGTGTTATCATCGTTTTTCTCCTTTGCATTACACTTGCTTGTCCTCTTTGTCTTCTGTTTCTATTCCTAAAACTACAGGTGAAGCATTATCCCA	T	C	ACCCGACTAAAGACTTCCCATGAGGTAAAGGAACCAGTTCCCAAGTACCACTGCTTTGTAGAGCACACATCTCATCAAGCATTGCTTGCCTCCACTCAGG	NA	NA	103

Marker IDs	Genetic positions (cM)	Chromosomes	Physical positions (bp)	Flanking sequences/Forward primers	ICC 12968	ICC4958	Flanking sequences/Reverse primers	Annealing temperature (0C)	Amplified Product size (bp)	
Ca-II-SNP948	248.62	Ca-*desi*-LG(Chr)5	14640464	ATTGTGATGAAATGACGGTGAGAAGGAATTAGCAAGAGATGAATGGTTTTAGGGTTTCAGATGGGTTGAGAAAGAAATGTTTTAGGGTTTTAGGTGGGTT	G	A	GAGTTTGAATTTTAGCGACTAAACTTTCAGTGCTAACTATTTTTTAATTACATAGTTCTATTTTTCTCACCACCACACATATCAGTTCTTCTTTCTTTCT	NA	NA	101
Ca-II-SNP949	249.59	Ca-*desi*-LG(Chr)5	14700126	AAGCTAGTCCAAAAGACGATTTTCCTTTGCCTCACATTGATGTCCTAGTTGATAGCACTGCTCAATATTCTCTTTTCTCCTTCATGGATGGTTTCTCAGG	A	G	TCTTCAATCCAAATGGCATCACTTTGTAACAGAAAGTCCCCCATTGTGTAATGAATGTAGTTTTCTCCATATCTTCGGGAGCCATCTTGATTTGATTATA	NA	NA	216
Ca-II-SNP950	250.56	Ca-*desi*-LG(Chr)5	14721876	TCAAATTACCCCTATTGGTTGTCCCAGATTTTTCTAGAACTTTTGTGCTTGAAACATATGCTTCTAGTAAAGGGATAGGAGCAGTTTTAATGCAAGAAGG	T	G	ATTTCTGGACTGCCTGTACCAATGCCATTAATTCCCTCTCATATACTGATTTCATTTGTGCTCTAGGTGAAAGACCTTTACTCCAAAAAGCTAAAGGTTT	NA	NA	201
Ca-II-SNP951	251.53	Ca-*desi*-LG(Chr)5	14749055	TATCTTATTGACTTTTTTGGTTTTCTATTTATGTTTACTGAATTGATTAATTATAGTTTACTGAAACTTTTTTTTTTCTCCTGATTTATTTGTTTTTCTA	A	C	ATAAATAGTAATAAAAAATAACATTAATAAATAATAAAATATATATAGGCCGACCTTTCAGGCCTAATAGACAAACCAACAAACCAGAAAAACCATTAAA	NA	NA	168
Ca-II-SNP952	254.44	Ca-*desi*-LG(Chr)5	14772066	AATACTAGTCAAGAAAAAGATGGATGCCATATGTAGGGATGGGAATAGGCTAGGCCGTCCGACAGGGGCCTATGGCCTGGCCTACTTATGGTCTGGCCTG	G	A	TCAGGCCTAATAGGCTTCTTTATAAGCCTGAGCCTGATCTATTTAAATAAATAGACTTTAAAAATAGCCTGAGCCTGGTCTTTTTATTAAATAGGCCAGG	NA	NA	142
Ca-II-SNP953	255.41	Ca-*desi*-LG(Chr)5	14795209	TTTATACAATCAGTATGCGTCCAAAATATATCTCTCAAAACATCTGAATTCTCCCGTCTTCTTGTCCAATACACATAATTTTCTTGTTGTATTAACTTCA	G	A	GAGAAATAAAGCTAATCTCTCAATTCCTAGTCAAATATATAAAGCAATGAGTACTTATCGAGCTTCATTGAGAGGTTCGTACACAGAAATGCAGCATGTG	NA	NA	218
Ca-II-SNP954	256.38	Ca-*desi*-LG(Chr)5	14837148	GGTGGTGGGTTGATTATCGTCCGATGTCGCTTGTTGTTAGTGATCGAGTATGTTTAAGGTTAAGGTTATGGGTTGATGGGAAGAAGTATGATGGCAGATT	C	T	TACAACTCTTCCTCGTTTTTGTCTATATATTGAAACAAAAATTCAATAAGAGAGGGGAGAGAAATCAGACGATAAAATTGGGGATAATATCAAAGAAGAA	NA	NA	152
Ca-II-SNP955	257.35	Ca-*desi*-LG(Chr)5	14883642	TTTTAAATTTTTCTCAATTCCCTTTTATGTCTCTATAGGTTGTATTTATTTATTTTTAATTTAGTCATTTTATTTATCTCATTAGCCACTTTAACTTTTA	C	A	ACCACATCAAACTATTATGCACACATTAACATCTTAAGGGTCACGTTATACTTTTTTAACGAACTTATAAAAAATAATATTTGGGTAAGTGGATTATAAC	NA	NA	102

Marker IDs	Genetic positions (cM)	Chromosomes	Physical positions (bp)	Flanking sequences/Forward primers	ICC 12968	ICC4958	Flanking sequences/Reverse primers	Annealing temperature (0C)	Amplified Product size (bp)
Ca-II-SNP956	258.32	Ca-*desi*-LG(Chr)5	14900249	ATAATTTGAAACTGATTTGACTGAGTCTGTTACAGCCTTGCTTAATCAGTTGTGTCTTCTACTTTAAGGGCCTTTGAAGCTTCTCACTTAATCTTTGATG	T	C	TTCTGCATAATTACAGTAGCGTTCGGTTTACTTCTGCAATGGCTTATAATCTTTCTCCAAACTATTTTGGCTAGATTCAAGGCTCCAGATCGAAGCTGTA	NA	NA
Ca-II-SNP957	259.29	Ca-*desi*-LG(Chr)5	15075698	CCACACTACGGTGCTAGTGAACTAATTTGGAAGCTGCGTTGCGAGCGAAGATTTTACCGAATTACTCGCGGTACCGGAAAACCACGTTAAAGCTAACGCT	A	G	TTTCCCCCAAATCAATACTCAAACCCTAACTAGATCCTTTCCACACAATCACAGATAAGTCCCTAAATGCAAACTAAAAGTTTGGAAGGAGCCCTTACCT	NA	NA
Ca-II-SNP958	260.26	Ca-*desi*-LG(Chr)5	15107815	GTTGACTGGTTGTCATAGTAAACAGATGTTGGTTTGCTAAAAGTAATGTGAAGATCTTTGAAGAGGTAGTGCAACCATTGTATTTCACAGGTTAGGTTGG	T	C	ATGTTATGATGTCTTCTCAAGCTCACCTTTAATCTCTTGGAAATCAAAGAAACAGTCCAATGTTTCAAGGTCTAGTTCTGAGGATGAATATCGCGCATTG	NA	NA
Ca-II-SNP959	261.23	Ca-*desi*-LG(Chr)5	15129853	TTTGAGCTGAAGTACTAGGCTTGTGAAGGAAATGATGAGTTTTGTATCCGAGGGAGGAGGGATCATGGGTGCAGGGTCAGAGGGAGGAAGAATAATGTTT	G	A	AGAGGAAGAAATGGAAGGTACATTCAAACTATAAGCAACTTATTTATGTCCCCCACATTGAACCTGAACCAAACTCATTTGTTACCCTCTCCCCAACCAA	NA	NA
Ca-II-SNP960	262.20	Ca-*desi*-LG(Chr)5	15656274	ATAATTTGAAACTGATTTGACTGAGTCTGTTACAGCCTTGCTTAATCAGTTGTGTCTTCTACTTTAAGGGCCTTTGAAGCTTCTCACTTAATCTTTGATG	C	T	CTCTAATATCATCTATTTCATATATAACTTGTAGTGCAGGACAGTGCAATAAATTCAATGATTCAATGCTTTGTAATTTGTTTTGCACATGACTTGGGAACACATTCAAAAGTTGGTCACATGATGTGATTTCCAATTTTTTTAATTTGCCAAAGGAATTTTGAGGAAGTTGGTTTGGCCATATTGACTTCAAGTTGTCCATTTGAGAAATCACTAATGTCTCCAAG	NA	NA
Ca-II-SNP961	1.88	Ca-*desi*-LG(Chr)6	566924	AGAGATCACACAATGATATATCCTGGCTCATCCAATCTGGGTTACGTCCAGTCCTCACAACCGTGAGATTTTTCACTAAGTGTTCAAATCGGAGAATGTT	T	C	CCTTTCAAGGCTAAAAGGTGATTGTAAAAATCCTTTCTAGGTTGAAAGGTGAAGATTATAACAGATCAAGGTTGATCTGGACTGGTGGTGTAAAAACCAA		NA
Ca-II-SNP962	2.82	Ca-*desi*-LG(Chr)6	593723	CTAGTTTCTGAAAACCCACTTGGTTCAAATAATGTTCTTCTTTGGTCTAGGTACTAGATCCCAAACATCATTCCTCTTAAATTCTTGAGAACCTGCAAAA	C	T	AACATACAGGTGATATCTTTCTTTAAATTTAATTATTGTTTGATATTATGTTTATTGTGATAACAAGAAGCTTGATCTTAAAAATTAAAGCTAGATAAGT		NA
Ca-II-SNP963	3.76	Ca-*desi*-LG(Chr)6	615409	GCCTTCGTATTGGTTACCAATGTGTAGATCGACATGAATAAAAGAATGTTCATCTTCAGATTGGTGCGTAGATCGGCAAGAATGAAAGGATGATCGTCTT	T	C	CTAATTTTCTTCATTCCTATTGATCTACGCATTAGTAACCAATTCGAAGACGATCATTTTATTTTTCATCCATGTCGATTTACACATCGGTAATCAATCA		NA

Marker IDs	Genetic positions (cM)	Chromosomes	Physical positions (bp)	Flanking sequences/Forward primers	ICC 12968	ICC4958	Flanking sequences/Reverse primers	Annealing temperature (0C)	Amplified Product size (bp)
Ca-II-SNP964	4.70	Ca-*desi*-LG(Chr)6	627292	ATCCTGATTTTGAAGAAATGTGTGATGATGTGGAACTTAATTTTGACGCTATGAATGACGAAGTTGAACCTGTTATGATTGATTTGACTGATGTGTTTAC	C	T	CAGTTTCAGATCTAAAAATCACAACAACAATTCCATTTTCCCTTCCAACATTTCTAGCCCATTTCAATAATTCATCTCGTGTATCAAACATCATGCCGGT		NA
Ca-II-SNP965	5.64	Ca-*desi*-LG(Chr)6	671394	GATTGTAGAGTAACTCTTTTGACAATCATTCCAATGTGCAAGTGTAAATTGTACAATGCATTCTCTATCATTTGTTTTTTGTTTTAGAATACCCCCATAC	T	C	ATAGTACAAGCAATCAAAAAACAAGTCAAACAAATACCTTGCTTGCATCTAGCTGACCCATTACCCCAAAAAATTGTTGAAACAGATGCTTTAGATATCG		NA
Ca-II-SNP966	6.58	Ca-*desi*-LG(Chr)6	737931	TGATATTTGGACGAAAACGGAAAAACTTTTCTGGAATAGTTATCGATCACCAATGGCAAACGATAGGTTGTTGTTGTGTTTCCACTTTGGTTTTAGAGCT	A	G	GGTTTTATTTAAAAATAAATAAAGGGACCAATGGGTTTGACCATGGACTCTAATTGTTAATAAAATAAAACAACCAAATAAAAAAATGGAATTGGATGGG		NA
Ca-II-SNP967	7.52	Ca-*desi*-LG(Chr)6	745730	TCTATTGTTCCTTTCAATGACAAGGTGCGATGCATTGGTTTGATATATACGTAGAAACAAGAGATTATCCTGTATGTAATTCTTGCAATTGGGATATAAC	G	A	TTATTTCAGATACATAAAATTAAAAGACTGACACACTATTTGTTTACTTACTCCATGGATAGCAAACATTACCCTTTGTTACCATGCATGGTCCTGATTG		NA
Ca-II-SNP968	8.46	Ca-*desi*-LG(Chr)6	749404	CATTCTTATACAATATTTTCTATATCCTAGATTATTCGCTTTCGTAAAAAATACAATGTCTATGTTTAATTATCGCGAAATTTCTCTTGTTATGTGCATT	A	G	CGTTTTTCAATTTACAATGAACGATTAACTCTAAATCTCCTTTAAACTTGTTATAAATTTCCATGTCCAATCCAACAACATTATACATCCACATTAATGT		NA
Ca-II-SNP969	9.40	Ca-*desi*-LG(Chr)6	779347	AACATCCTCTTGAACTTATTCATATTGACCTTTTTGGTCCCATCAAAACTCCAAGTCTTAGTGGTAAAAGATATGGTTTTGTTATTGTTGATGATTTCTC	A	C	ATTCATCTCAACAAGTGTTTGTATTGAAGAGGGTTTCATCTCTGTAGTTATATCTGAAAAGGGTCTTGACTTTGTCAAAGATATTCTAATAGACCAAGCTATATCATCTATAGTTCTTTCTCAGCTTCCACAAATTGAGAAATCTGTTCAAGTACCTCTTGTTGGAAAAGCTCGTGTGATTCTTTCTGAGATCACAATCAAAGATATCCAAGTCAGTTCTTCATCTGTTGAAA[illegible]		NA
Ca-II-SNP970	10.34	Ca-*desi*-LG(Chr)6	810210	ATCATGTTTAACGCTCTATAATACTCCATTTGAAAAGTTGTTGTATCTGATCTTAAGACCAGATACATCAACTTTTCAGTTATCATTTTTGCTTATATTT	C	T	CTTGTGCGTCTATCTACTTTTTGCTGAATACAAGTCGTACTCAACGTGTTTAAATTAGTTGCATAGGGAAATGGTGGTTTGTGTTATACTATTATGGAAT		NA
Ca-II-SNP971	11.28	Ca-*desi*-LG(Chr)6	829507	CTAGCCCTCGCCTTTATTATTACTAAGATAAAAGTAAATCTCATTCAATTCTTCAATTTTTTTACTTTTTACCGAATTCTCGTTCGGATCTTAGTTTTCG	G	A	AAAGTCCACTTTCAAACATTACATGAACATTTCAGAAAACAAACATTATAAGAAAGAAAATGCTCATCTGACATTTTTTTTCCGCATCTCAAATACATTA		NA

Marker IDs	Genetic positions (cM)	Chromosomes	Physical positions (bp)	Flanking sequences/Forward primers	ICC 12968	ICC4958	Flanking sequences/Reverse primers	Annealing temperature (0C)	Amplified Product size (bp)
Ca-II-SNP972	13.16	Ca-*desi*-LG(Chr)6	839044	TAATTTGTACTATTTTGGTTTTATAAACTCGTATCTGTTGCCATGGCCTAGATCAACCTCTAAAATATCATAATGCCTTGCTCTATAATTTTATATATGG	C	T	AACTATATGTCAACTTAAATAATTTTACCTAAAAGATACTCACACTTGTCACTTTTGACGGTGTTAATTTTTTCAGTTGAAAACTAATATTAAAAACAAT		NA
Ca-II-SNP973	14.10	Ca-*desi*-LG(Chr)6	880921	TCACGTTTCCCGTTTAGTACTACGTTCCCCACTTTCCCTCTCACTTAATACTACGTTCTCCATTTACCCATTTAATGTCACGTACTCCACTTAATATCAC	A	G	TTTTCTATCAAAATGGTCTCCCTCTCCTTCTCTCCTAAACCCTTCCCTCTATATCCTAGATGGAATGCGTCCGTTTTGTTTTATTCATTTTTATGGGTAA		NA
Ca-II-SNP974	15.04	Ca-*desi*-LG(Chr)6	907028	AACATCCTCTTGAACTTATTCATATTGACCTTTTTGGTCCCATCAAAACTCCAAGTCTTAGTGGTAAAAGATATGGTTTTGTTATTGTTGATGATTTCTC	C	T	CAATTTTTGATCCTTTTTCGTTTTGAATCTTTTTGCAAAAATGGTGGAACGCCTCAAAGGCTTCATCTTTATGTTTTAAAAATAAAACTCAAGTAAAATG		NA
Ca-II-SNP975	15.98	Ca-*desi*-LG(Chr)6	1324148	TTGAAATTGTTAGGTTTAAGCTGTCACCACTTGTTTCAGCATATGGTTTCTCTCCTACTACCACTATTGCATATGAAAAGTTGTTTGACTTTACATAGTC	G	T	ACACTGCTCAACTGATATTCACAGGTACAACAATTCTCGGTGCTATAAAAAATACAGTTGACAAGGACACCAACATAATCTACAAGGAGTATCCAGATCT		NA
Ca-II-SNP976	16.92	Ca-*desi*-LG(Chr)6	1336769	CTTGTTATTACTTGAACTTATGCAATTTCCCTGAGCATTGACCTTTCTTTTATAAGCGTTTTAGAGCAATTAATAATGGTCAAAAGAGTTGTTGGTAGTG	T	C	CAAGAACGAAGAAAAATGCACAGTCAAACATATTTCATAAACTGTTCAAAGGCTGAAATATTTTTATTCGTATATATTGATTTTGGTGAAAACTGACAGA		NA
Ca-II-SNP977	17.86	Ca-*desi*-LG(Chr)6	1350303	CATCCACCGGCCATTCTTTATGACTTGTCATATGATTAGAACAACCATTATCAACAAATCAGAATTGGGACGAAGAATGTTCTTCGTTTGTGGTAGCCAT	T	C	GTTGATGAATGTTGGGCTGGAAAAGAAGGAAAACAAAAGAAAGAAGAAGATGAAGCATGTGTTGCTCAAGAAGATGACTTAAATTAGAAACAATCTTGTT		NA
Ca-II-SNP978	18.80	Ca-*desi*-LG(Chr)6	1363682	TTAGTCCAAAGTAGGTTGAAGACTTCGATGAGAGTTATGCACCTCGGCCGCTACCACTTTCCTTGTGAATTTTGTTGTTTAGCTGAAGTGATTACGACTT	A	G	AAGTTGGAAGTATGTAAGAACATTTCCAACTTGGACAAAACAATATAAGAAAAAGAAAAATACAATGAAACTAATGAAAGAAAATTATAAGAAAAAAATA		NA
Ca-II-SNP979	19.74	Ca-*desi*-LG(Chr)6	1399426	GCTCAACAGAAATGGGAAGTTGAAACACTCACAGCCTTCTGAACAGCCTCATTAGTCACTCTTGGAGAGGACTTTCTTGAAGCCTGAGCTGCAGCATTAG	A	C	ATTAACCCTTCTTTCCTTTATCCTATATCAGAGAAAACTATATGTTTCATTCCTGTTTGTTTCACTATTTTTATGGTTTCATTTGCAGGCCTTCTTCTCT		NA

Marker IDs	Genetic positions (cM)	Chromosomes	Physical positions (bp)	Flanking sequences/Forward primers	ICC 12968	ICC4958	Flanking sequences/Reverse primers	Annealing temperature (0C)	Amplified Product size (bp)
Ca-II-SNP980	20.68	Ca-*desi*-LG(Chr)6	1448279	TGGGGAGTTAATTGGTGTATTCCTTCACTTCCATGTATTTATAATTCAACACAGTGTTGTCAATAGCAATTATAGTGCCGCTATAGTGTTATAGCGTAGC	G	A	AAAATTTCGCTACGCTATAGTGCTATAGCACTATTATACCTGCTATGATGACACTTTAGTACTAAATAGCATATTGCAGAGCAATAGCGATTTCAAATTT		NA
Ca-II-SNP981	21.62	Ca-*desi*-LG(Chr)6	1455782	GCATTTTTTTCCTACCTTCAAAAGGAGTTATTCCTCTTCATAACCATCCAGGAATGACTGTTTTCAGTAAGCTTCTATTGGGACAAATGCACATTAAGTC	A	T	GTTAATTGAATTTGATGATATGAATAGAGATTAGAAAATACACACATTGAGATGGTTGTTGCAACAAATTGTGAGAGACCTCAGGATCAACCCAATCATA		NA
Ca-II-SNP982	22.56	Ca-*desi*-LG(Chr)6	1471831	CCAGCTTATAATTTTGTTATCTCGCTTGTTTTTTACTTAAAAATGATACATTTTTATTGGCAATATATCTTTTGATTCATAATAAGATTCAATTCACGAT	C	T	TCAAAATTAATTTAGCATGCAATTTAACATCGGTTGTCTATGTCATAGGCCATAGAATCAAATCGAGATTCAAATCATGAATTAGAAGACCTATTTTTCG		NA
Ca-II-SNP983	23.50	Ca-*desi*-LG(Chr)6	1498906	TTCTTGGTTTTACTTGTATGCAGAGTACAAGGATACAAAGCATAGTCACATCAGTGAATATATGTGCTTTGATGTTTGTCATAGTAGCTGGTGGTTACTT	A	G	TTAAAGATACCAAAAATATATATGGAGAAGGGGTATTGAAAAATAATGATGTAAATTACCCTGTAGGTAGTTGATATCCAATCCAACCAGTTTTGAATCC		NA
Ca-II-SNP984	24.44	Ca-*desi*-LG(Chr)6	1558305	CAATATGAATAATATGTATCTAAATTTGAGATCTTTGTAGTTGTTCCAAGCGTATAGGGATAGTTGAGTCAAAATGAGTGCTTTGTGGATTCTCTGCATA	A	G	CATATGAAATATTTATAGAAATATATATAACTCTATATGTTTTGCAGGTTCGATTGGCTATGGCATGGCCACAAGCATGTGTTTCTGCTTACTATGTTGC		NA
Ca-II-SNP985	25.38	Ca-*desi*-LG(Chr)6	1558359	GCATTTTTTTCCTACCTTCAAAAGGAGTTATTCCTCTTCATAACCATCCAGGAATGACTGTTTTCAGTAAGCTTCTATTGGGACAAATGCACATTAAGTC	A	T	GTTCAATTTTAGTACCAATTATTTCAACCTTTAGAGGCTTTGCTAGGTCAACAAAACTTGTTGGTGGAGATATTGATGAGTTCATGTTTTCATGTGTTAGTTGTTATATGGGAATTGCATCAATTCTTGTGAGTTGTGGAAATAGATGGGGTTTTGTGCATGTTGGAGTTTATAATAAAGGGTTTGTGCAAGCTTCTAAGGATACATGGGACATTTTTAATAGGGTTGGATT[illegible]		NA
Ca-II-SNP986	26.32	Ca-*desi*-LG(Chr)6	1559819	CCAGCTTATAATTTTGTTATCTCGCTTGTTTTTTACTTAAAAATGATACATTTTTATTGGCAATATATCTTTTGATTCATAATAAGATTCAATTCACGAT	C	T	GAAGAAATCAAAGCAATGACACCAATTGCAAGACTTATTGGAGATTCAATGTACACAAACATAATAGCCATTGAACATGTAAGCAAAGGGCTAATCCAAAATGCTACTTTGATGGCATTTTTGGTGTTCTTGAAAGTGAACCATTGCCAAATGAAACCAAG		NA
Ca-II-SNP987	27.26	Ca-*desi*-LG(Chr)6	1593638	TACGATATGGTACTGTGTTCGTCCAATCTCTACCACCACCCCTCTGACTTCGCATCATCAAAATTAACTCATTAATGAGTGGCATGCCATCAGGTAATAT	T	C	TTTTTAATTAATTTAATTCATTATTGATATTTAATTTATTTTATTTTTTATTACAGCGTCGAGCAATACAAATTGTTGAAGAGCTGATTGGCCGACAACT		NA

Marker IDs	Genetic positions (cM)	Chromosomes	Physical positions (bp)	Flanking sequences/Forward primers	ICC 12968	ICC4958	Flanking sequences/Reverse primers	Annealing temperature (0C)	Amplified Product size (bp)
Ca-II-SNP988	28.20	Ca-*desi*-LG(Chr)6	1606173	CATGCTTCTCGTCCTCATACGCAGCAGCTCCAAG<br>CGTGGAATTCAGGGCACCAGGATCGGTGCTCTGA<br>TACCAGTGTTAATACTGGTATCAATTAGGAAT	G	A	CTTTCTCTACAATTCCTTGTAATTCTTCTATTCA<br>ATCAATAATACAAAGAGATTAGGGTTCATTGAT<br>CCTAATCCACCATTCAGTTTCAATTACGCTATT		NA
Ca-II-SNP989	29.14	Ca-*desi*-LG(Chr)6	1740238	CTTGTTATTACTTGAACTTATGCAATTTCCCTGAG<br>CATTGACCTTTCTTTTATAAGCGTTTTAGAGCAATT<br>AATAATGGTCAAAAGAGTTGTTGGTAGTG	G	A	TGTTGATGGTGTTTCTGGTTTTCATATTGATCC<br>TCTTAATGGAGAAGAATCAAGCAACAAAATTGC<br>TGATTTCTTTGAAAAATGTAAAGTGGATTCAACA<br>CATTGGAATATGATTTCTGTGACCGGATTGCAG<br>CGCATTAATGAAAGTTATACTTGGAAGATCTAT<br>GCAAACAAGTTGCTGAATATGGGAAACATTTAT<br>ACCTTTTGGAGACAAGTGAATAATGAACCAAAA<br>[illegible]		NA
Ca-II-SNP990	30.08	Ca-*desi*-LG(Chr)6	1840672	CGGTTGTTGTTGCTCTCAAACCGTGTCAATAATAC<br>CAAATTCTCTATCTCTCTCTCAAATCTTAGATCGAA<br>TTGATGTTTAGAATGTTGAAAATGGGTTT	C	G	TATATTAATATGAATTCATTTTTATTACAATAAAC<br>ATATATATCTAATAATTTGACATAATTTCAGGTA<br>GGACCCTATAAAATTTTGCATATTCAATCTC		NA
Ca-II-SNP991	31.02	Ca-*desi*-LG(Chr)6	2035499	TCTATATATTCTACATGAATCTATTATAACAACCAA<br>CATTTGGTCAGGGCAGGCTTTGATTTTTGTGTCAA<br>TTACACTTCATATGGAATATTCTTCATTG	G	A	ACATACTTGTTTGATGAACAACAACAACAAAAAT<br>TGATCCATACTTGATAGTACTATAGTATTGGCA<br>TAACAAAGACATGAAATGAATCTTGTAAGCCGT		NA
Ca-II-SNP992	31.96	Ca-*desi*-LG(Chr)6	2058649	GCCTCTAAACACTATTCGTGGTCCCCATGGATTG<br>CCCTTTCGCTTTCTTTATAGATAGAGTTATAGATG<br>ATACAAAAAATGACTTCAACCTAGTATTTAC	C	T	AACAAATAATTGAGAAGTGTGAGTATTTACTTAT<br>AAGATTTTTTAATCTCAACTTTCAAGTTACGTAA<br>GACATTTTTGTTTATGCTGAAAAAATTTGAAA		NA
Ca-II-SSR328	32.90	Ca-*desi*-LG(Chr)6	2160721	TCTTAAGAATCACTCGAATATTGAAA	(TAT)16	(TAT)29	ATCTAAATTTTGTTATTTTTAAATGGGATTCACA<br>CATATAACGACGATAATAACAATAACAATAATAA<br>TAATAATAATAATAATAATAATAATAATAATA	GGGATTCACA<br>CATATAACGA<br>CG	57.75
Ca-II-SNP993	33.84	Ca-*desi*-LG(Chr)6	2161729	CGATTTGTCTTCTTCTTCTCAAAGAGAAGAGCTTA<br>TTCGAAAAGTTGCTTCTGCTTTCAACGGCAAGCTC<br>AACATACTTGTTAGTCATTTCTTTTCTATA	T	A	TCTTATAATTTATGCTAAAATTGTTAAATATAAAT<br>AACTAGTTTAGGGATTTGAAGCAATTTGAAAAT<br>TTATAATTTTTATGATTTATGCTAAAACACAC		NA
Ca-II-SNP994	35.72	Ca-*desi*-LG(Chr)6	2203748	CTTGTTATTACTTGAACTTATGCAATTTCCCTGAG<br>CATTGACCTTTCTTTTATAAGCGTTTTAGAGCAATT<br>AATAATGGTCAAAAGAGTTGTTGGTAGTG	T	A	GATAAAACAAGTAGTAGAAGAAGTTGGAGTTGG<br>GCAAGTTGTCCAAGTGATTACATCAGGCGAAG<br>AACAGTATGCTGTTGCTGGGAAAAGGCTGACT<br>GATACTTATCCTAACCTTTATTGGAGCGCGTCT<br>GCAGCTCATTGCATTGATTTGATACTTGAAGAT<br>TTTGGAAATCTTGAGTGGATTAGTACAGTAG		NA

Marker IDs	Genetic positions (cM)	Chromosomes	Physical positions (bp)	Flanking sequences/Forward primers	ICC 12968	ICC4958	Flanking sequences/Reverse primers	Annealing temperature (0C)	Amplified Product size (bp)
Ca-II-SNP995	36.66	Ca-*desi*-LG(Chr)6	2208003	CTACAAAATCAGACAAGTATTGTTGTAGTGGGGAATTTGCTGATCCAAATATTTGCAAGCCAACTTTGTTTGCTCGTGTTTTTAAGACAGTTTGTCCTCG	T	A	GTGAAAAAAGAACTGATCATGCAGGGCAAAATGTAATGGCATAACGTTTTGCCAAACAAGTCATAAGGGAAGTTGAATCATCATAAGCATAACTATAAGC		NA
Ca-II-SNP996	38.54	Ca-*desi*-LG(Chr)6	2235926	CTTGTTATTACTTGAACTTATGCAATTTCCCTGAGCATTGACCTTTCTTTTATAAGCGTTTTAGAGCAATTAATAATGGTCAAAAGAGTTGTTGGTAGTG	C	T	ACTGAAACATATAAGAAGTAAGTTGTGAATGGAACATTGGAGAAGATACAGAAAAGGAAATTAGCAACATAACAAATTATAAAAGAAACTTATTTCGAGA		NA
Ca-II-SNP997	39.48	Ca-*desi*-LG(Chr)6	2348827	CTCTTCTGCCAACTTTTTTCTTTCAGTTTCATTATGTGCATCACGCTTTTCCAATTCAATTTTCCGTGACTCAAGCTCCCTTTTCTGAGATTCCAGTTGC	G	A	TTTTATGCAATGATTCCTCTGTATTTAGAGATAAAGAAAATACAGACAAGTGCTAAGGATCACTTCCAAAGGATTTTTAACGATCACGAAAAGCTTAAAT		NA
Ca-II-SNP998	40.42	Ca-*desi*-LG(Chr)6	2376749	CACTGTTTTCTCTTTCTTTCGAGGGTTTTTTTCTTTTTCTCTTTCTCTTTCTTGGAGTGTCGTACGACTGCCATATTGAGAGTGTAATTATAGAACGGTT	T	C	CGATGCACTGCCCAAATTGTGCAAGACAGACTATCAATCATGAAGTCCCCAGGATAAAACAGTCCAGATACTTTGTATTGGAGTTCTATTGCACTGTAGA		NA
Ca-II-SNP999	41.36	Ca-*desi*-LG(Chr)6	2408161	CTTGTTATTACTTGAACTTATGCAATTTCCCTGAGCATTGACCTTTCTTTTATAAGCGTTTTAGAGCAATTAATAATGGTCAAAAGAGTTGTTGGTAGTG	A	G	TTGGAACCGGGTGATGTACTTGTTCGTGTCAATGGTGAAGTAAGTTTGCTTGATGGAAAATCCCGCCCCCTTTCTGGAAAAAGAATCCTTTCCATTTTCTTTTGACAGGACTACAAGCTTGAACACTACTTCAAGTAGCATTCCGAAA		NA
Ca-II-SNP1000	42.30	Ca-*desi*-LG(Chr)6	2565245	GGTGGAGCCTAACGTGCAGTTGGTTGTGATTCCGATAGAGGAAGCAACAAAGTCGGGTCCAGCTGCTTCTTCATCCTCAGTGGATGCAGCTAGTATGTCC	A	G	ATCAATAAGAATGTATCTGAAAAAGTTTATGCATTTTTTTAAAATGTATTTTGACAAAAGTAATGAAGTTTTCTTTACCTTTATCTTCCCTCCACCATTA		NA
Ca-II-SNP1001	43.24	Ca-*desi*-LG(Chr)6	2624838	CTTTTTCATTTTAAAGATAAACCATTTTTCCTTGATATTTTCCCATTTGATTTCGGGTACCCTCCTTTTGAAGCGTTGCAAGACTGACTCTGCCTTTTTT	C	A	AGTGTAGTAAACAAAGCTCATCTATTGTAAGGATACAAGTTAGACATTTTAACCCTCCTAACAACTTAAAATTGACAAGAAAATATAGAAACAGCAAAAT		NA
Ca-II-SNP1002	44.18	Ca-*desi*-LG(Chr)6	2648414	ATCGAGATGCTCTAATTTTTGCCGAGGTCGCTTTTTCAGGTTTTCAACCTAGCGAGCCCATTTTTCGATCATCATTTTTCCTAAGCATAATATTTTTTGA	T	C	CGAAGGACCATATGTTGTGAAAAAAGCCTTCTCTGGTGGAGCTCTAATACTCGCAGAAATGGATGGAGAGGACCTTCCACTTCCAGTATATTCAGATGCA		NA

Marker IDs	Genetic positions (cM)	Chromosomes	Physical positions (bp)	Flanking sequences/Forward primers	ICC 12968	ICC4958	Flanking sequences/Reverse primers	Annealing temperature (0C)	Amplified Product size (bp)
Ca-II-SNP1003	45.12	Ca-*desi*-LG(Chr)6	2657388	CTCATGCAGCGGTCTTTTTGCTCCAATTGCACTACACTTGATGAGATGAATTACCAACTCCTTCTTGAATTCCTTCCTTTTCTATCTTAAACTCTTTTTG	T	C	CCCCACTCCTGCCTTAACGTTCCCACGGCTTAACGTTCCTCGTAAAAGAAAAAAAAACAAAAAGGGACGTCTTCCATTCAAAAACACGTTGTGCGATATA		NA
Ca-II-SNP1004	46.06	Ca-*desi*-LG(Chr)6	2661720	TGTTGGGCGTGTTACTTCATTGGCATTCAGTGAGGCTTCTGCCTTTGGTCCTTTGCAATCCCTGGCTAGATGTCCGGGCTTCTGGCATTTAAAACATACA	C	T	CTCCATTTCAAATGATAGACATAGGACTTTAGAATCAAAGCCATACTGTTTCGTTGTGGAGACCCAAGACACCTTACGACTAAGTGCCCTCTCAACGGCA		NA
Ca-II-SNP1005	47.00	Ca-*desi*-LG(Chr)6	2720364	GTGTGAAGTAGAGGTTGCATGTACCTCACTTTTGAATTCTTCAAGTGATTCACCAGAGATATCTTGATGCAGAAACCTCTTTACAGCAACTTCCTACAAA	T	C	TATTTCACTTGTCAGTATCAGTACTTTTATGGGTAATATTGTATTATGCTGTAAATAACAGATTTTAGTATGTATATTTTTAGCATGTTTCTGCTCTTTG		NA
Ca-II-SNP1006	47.94	Ca-*desi*-LG(Chr)6	2725154	ACTGAATGCAACCAATTCGAAAGCTTTTTTCGAGTAGTAGGCGATGAAGATGAACCATCATTGGACTTGTTGTTCCTTTCTGCAGCAGACCCTTGTACAT	C	T	AGAGGTTTTCACTTCTTTAACATTCAGTACTGTTACAACAAGGTGAAAAGTTATGAAGAACATTCTTAAGAAGCTTCATATAATGTCTAATCAATCAGAA		NA
Ca-II-SNP1007	48.88	Ca-*desi*-LG(Chr)6	2742457	AAATCAAGCTCTTGCGCTTGCTACGTCATCCTGATATTGTAGAAATTAGGCATATTATGCTTCCTCCTTCCCGCCGAGAGTTCAGAGATATCTATGTTGT	C	G	GACCCCGAAGAAGTTGGTACAAGAAAAATTGGTGGTGCTCAGGAGTAAGATCATCATTTGCCTTAATGACTTGATGTAGATCAGATTCCATCAACTCAAA		NA
Ca-II-SNP1008	49.82	Ca-*desi*-LG(Chr)6	2771903	CATATGAGCACAAGGCTCCATCTTATGAGTACAAGTCACCCCCACCTCCACCACCTTACATTTACAAGTCTCCACCACCTCCACCCTATGAGCACAAGTC	A	T	GTGGTGGAGGTGATGGTGATGGAGGAGGTGGTGACTTGTACACATAGGGTGGTGGGGGTGATGGTGTAGGAGGCGGGGGAGACTTGTACTCATAAGATGG		NA
Ca-II-SNP1009	50.76	Ca-*desi*-LG(Chr)6	2775649	ACCAGTGTTTGCACGCCACCCCTACGCACACATTACATGTTATCTCATGCACTATTTAACAAAAACTCTTATTTGTTTTCAGGTTATAGTTTTCTTCGAG	T	A	AATATCGTAGCTCAAAGATGATAAATTCAAACGTTTATTAAAAGAATATGGACAGGAAGCTAGATGTTTCACTTTTCTCCAAATGGAGCTTGCCTTTATT		NA
Ca-II-SNP1010	51.70	Ca-*desi*-LG(Chr)6	2844907	ATAGCACATAATACCCCATGAAGGTAGCCCTTATAGGTAGGGGCAACAATTGGGTGGTAAAGTTTACTTTGAGAATCATTATTATTTGCATGGTTAGTGG	A	G	TGGTTTACACCCCACAAAATGAGTCATAAGTAGCCACTATGATCACCAAAACCAGGATGATAATTCTCAATCATCTCTTCATAACAATCAACCAAAATCC		NA

Marker IDs	Genetic positions (cM)	Chromosomes	Physical positions (bp)	Flanking sequences/Forward primers	ICC 12968	ICC4958	Flanking sequences/Reverse primers	Annealing temperature (0C)	Amplified Product size (bp)
Ca-II-SNP1011	52.64	Ca-*desi*-LG(Chr)6	2931598	TGAGTTTATTTGAATATTTGAGTTATGTATTTGATGAATACATGAATTTTCTTAAAATTGGTATTGATATTTATGGATTTTGGTGAATTTTCTAAATTTA	T	A	AGTCATTTTTTTTTATTTTTTATTTGTTTATTTATTTAATTTTAAGTAACAATCTAATTATTGATATCTTAAAATGTCAATAATATTATCATTTGTTTAC		NA
Ca-II-SNP1012	53.58	Ca-*desi*-LG(Chr)6	3080317	GAAAGAATTGTGAATTGTTTACAGATTGTCCAGTTTCCTCGAAACCTATCTATCTCTCACAATCTCTTTCCTATTGCTCACCCCTTGATCCTTGTAGTCA	C	A	AACGACTGTTAGAAATCCAGGTAGAAACTGGTGGCGATCTTCTTGGGTGAGAGGCCTCATCAAGAAGGAGCCGTACTTTGATTTTGTGTGAGTCTGCTGA		NA
Ca-II-SNP1013	54.52	Ca-*desi*-LG(Chr)6	3532503	GCTCGCAGCGTCTAGGATCAGCAACAAGAAGAGTACGGTCATAACGAACAAGAATGTCCTTGATCTCCTTCTTACTCTGTTCATCCACATATTTCTGATA	G	A	GTTTCGCCGGCGTTGACATGAGGATCAGAGTGAAGGGAGGAGGACATACTTCTCAGATCTATGCCATCAGACAGAGCATCGCAAAGGCCTTGGTTGCATA		NA
Ca-II-SNP1014	55.46	Ca-*desi*-LG(Chr)6	3542345	ATTCTTCTCCACCGGTTGCTTTGTTCGGTTCAAGGAAACTCTACCTTATGGAATGAACTCTTATGGACTCGTTCCATTGGTTTGATCTTTCTTTACCCTT	T	G	ACAATTGAGTGCTTCATCAATTAGTTGTGCATCGGAAGTCACAAATCTTGTGTAGGAATTGGAACTTATTGATGAAGAAGAAGTAGCGTCCTTGAAATGA		NA
Ca-II-SNP1015	56.40	Ca-*desi*-LG(Chr)6	3557055	GTGTGAAGTAGAGGTTGCATGTACCTCACTTTTGAATTCTTCAAGTGATTCACCAGAGATATCTTGATGCAGAAACCTCTTTACAGCAACTTCCTACAAA	T	A	AAGTACCACCAAAGTCAGGTGACTTGATCTCTAGTCTATTACAATAAATGATAATATGATCTATTACATCTGTTTATCCCAACTCCACAAAATTAATAATACAACCAAAATAATGCCTTCGAATTCACAAATTAATGGGAATTAACTACGTTGCTACCCCCAACCCAACCCATAAAAAACATAAAATCTCACTCAACATTCAGCAACGCTGCATTATATCATATTAGACCGTTTG[illegible]		NA
Ca-II-SNP1016	57.24	Ca-*desi*-LG(Chr)6	3591192	ATGTGGCCGACACAATGTGTTTCCTAATGCTCCTAATGCACTTATGGCTGTAAATCTATAAAATAATTGAGTCAATTTTTTCATCTACAGGGTTCAATGT	T	G	GAACAAACAAGCATAAATAGAGACAAAAGAGCTGCTGTACCTTGTCCTGACCCCCGACATCCCACACAGTGAAGCTGATGTTCTTGTACTCCACGGTTTC		NA
Ca-II-SNP1017	58.08	Ca-*desi*-LG(Chr)6	3613223	TATTCTTTTTACATTTTTTTTGTTTGAAAATATTGCAAATTTATTTATTAGTTTTTAAATCCATCTTCTGAAGCCTTTCATTTCATTTCACTCTTTTATC	C	T	TGCCATTGTTGTTGTTGTGTTTTTTTGTTGTTGTTGTTGTGTTGGAATGAAAGATGAAGAGAAGAAGAAGAAGAAGATAATGAAAAAGGGATAAGAAAAT		NA
Ca-II-SNP1018	58.92	Ca-*desi*-LG(Chr)6	3712916	CAGAATTCTTTATACACATCAGAATTCTTTTCATCTTTAGATTTCTCTTTTACAATCTAGTCCACCATATATGACCATATTATGGGTCAACCATGACTGT	T	C	TGCATAAAATTTTTATGCAACATATCCATATCGATCTATATCATCAAGTACATATTAATGTGGTGGCCATTTGAATTTAGTCACATTATATGTTAGTGAC		NA

Marker IDs	Genetic positions (cM)	Chromosomes	Physical positions (bp)	Flanking sequences/Forward primers	ICC 12968	ICC4958	Flanking sequences/Reverse primers	Annealing temperature (0C)	Amplified Product size (bp)
Ca-II-SSR329	59.76	Ca-*desi*-LG(Chr)6	3786461	TCTTGCACAACTCATTTCGG	(TAT)15	(TAT)17	GTAAATCAAACATAGGCTAATTCCTCTTACATTTATCAAACAAAATAATAAAGATAATTTAAATAAAATGAAGAGAAAAAATAAACACATAAAAATAATA	TCAAACATAGGCTAATTCCTCTTACA	59.84
Ca-II-SNP1019	62.28	Ca-*desi*-LG(Chr)6	3998825	AGCATCATCCATTCCATAGCTAACAGTCCCATCTCCAATTCCCTTCTCACCTCGCTCAAGCTCCTCCAATAATCTGAAATTCCTAGGAACTGCATTGATA	G	C	TGTCAATGTAACCTATCATTGACTACATTCATCTGATATTGTAACTGTTCTATTGCACACATAGATTTGTAATTTTAACTTATCTTGTTAAAATTTTGGT		NA
Ca-II-SNP1020	63.12	Ca-*desi*-LG(Chr)6	4052251	CATCCAATATAAAACGTCTATCACATTATTCTTTTTATTTTTTTTTAAGTTGTATAATTTAAATATCATAGCCTTGTAAAAAAGGTTTTTTTTTTTTTTT	G	A	ATTTAATTGAATTAAGTAATTGGACATTAATCTCATTGCCTAGCAAAAACATAACAGATTATTTAAGATTTCGTTTCTGTCCTTCTCACAAGGTTGTGTG		NA
Ca-II-SNP1021	63.96	Ca-*desi*-LG(Chr)6	4077783	TTCGCGGCCAATGCGATCACTGACAACAAGACCAGCATTCGTAACATCACGAATCACAATCTCGTCTTCCCACGACATTGTGAATTGGAGAGCTGCAATT	A	G	CTCTCCACGGTCTTCTTTCTCTCCCTTTCCCGCTCTCTCCCTAATTTGATTCTCCATTTGTTTATCGGGAAACAAAAGAGGGCTTCAATTTGATTCCACC		NA
Ca-II-SNP1022	64.80	Ca-*desi*-LG(Chr)6	4132081	GTGTGAAGTAGAGGTTGCATGTACCTCACTTTTGAATTCTTCAAGTGATTCACCAGAGATATCTTGATGCAGAAACCTCTTTACAGCAACTTCCTACAAA	T	C	ATTTATTGCTGGTGAAGCAAGACGTGTCTTCCAACTTGGGGCCATCAGAGCTCAAGAATCACTTGGATGAACTTGGAGATCAGTTATTTAGTGGGACATGCAATTTCCTTCCCAAAAATAAAGACCACAAACACAAGGTACCGTCCGCTTTCAATGTCTTCAACGACTCAACATCCGTTTGTGCAAAAGATGGAATAACGGTG		NA
Ca-II-SNP1023	65.64	Ca-*desi*-LG(Chr)6	4135764	TATGCGAGTTTTTTTTTTTTTAAAGGGTAGAAAAGAGGAAAAGGAAATTGATTTTTTCTTGTTTACATATTTGATTCCATTATTTGCATAAGTCATAGGT	T	C	CCTTCGACAAGCCACTTTAATGAATTTAATTAAGGTTAAACCATATGGTAATAACCAAGACTCATTGTAATTCAACAAGTCCTATTAAAAGTAACAGTCA		NA
Ca-II-SNP1024	66.48	Ca-*desi*-LG(Chr)6	4260700	GAGCATAAAATAATTTATTGCAGAAGAAGATGGTTTTGGTCTCCAAATATGTGTTATCTATTTCTCTATTACTAACTATTATCGTTGCTTTGCAGTTTGA	T	G	TTTTATCTTTACATTGAACACCAAGCTGTAAGCCACCTGTTAAGTTGTTATTAATAAAAACGGTTACTCTTGGTTTTTGGAGCAGATTGCTTTTAGAGCC		NA
Ca-II-SNP1025	67.32	Ca-*desi*-LG(Chr)6	4331449	TTGTTGTGGGGACAGGTGAGGCAGGAGAAGGAGTAGGTTCAGGAGAGTTACGCAGTGGAGAGGTTGGTAGTGGTGATGGTGTATCCTCAGGTGGAGGATG	G	A	ACATTAAACAGATGAAGATTCAAACTGATGACCGTCCTGTCTCAAAGAAAAGAAAAACTCGTCCTTCCTCTGTTTCTCAAAACTCTGAAGATGAACCATC		NA

Marker IDs	Genetic positions (cM)	Chromosomes	Physical positions (bp)	Flanking sequences/Forward primers	ICC 12968	ICC4958	Flanking sequences/Reverse primers	Annealing temperature (0C)	Amplified Product size (bp)
Ca-II-SNP1026	68.16	Ca-*desi*-LG(Chr)6	4403765	CCACATACAACTCCATAATGAAATGTGATGTTGACATCAGGAAAGACCTGTACGGTAACATTGTCCTTTCAGGAGGTACAACCATGTTCCCTGGCATTGC	T	C	AACCACCAATCCAGACACTGTACTTTCTCTCAGGGGGTGCTACCACCTTGATCTTCATGCTGCTTGGGGCCAATGCAGAAATTTCCTTGCTCATTCTATC		NA
Ca-II-SNP1027	69.00	Ca-*desi*-LG(Chr)6	4407916	TTGTTTTGATTTTATGCAATGGATGTGTTAAGATTGTTTGTTATGGAAATATTTAATGGTTTAATATTTGAACATTTGAATTTCGTGAAATAAAAGTTGT	A	C	AATTGGATTATAATAAGATATGATAACATATATATCATTTCCCCTTTTCTATCTCGACTAGTAAAAAATTATTGCAGTTAAATTAAACTTGTATTTGAAA		NA
Ca-II-SNP1028	69.84	Ca-*desi*-LG(Chr)6	4421550	ACAAGAATGATTCATGGATGATTCTTGATAAAACGGCTTCAATGGCGTTAGAAACGGCCGTCTTTGAGTTTCTATGCTAGGGCACAAGTCTCCCAAGTTC	T	C	CGCGCGCCTGGAGCGCACCCTGCACGCCTGGGGCGCGTGACGCGCATCAGCAATCATAAAAACGCAAATTTTTACTGTTTATGGATCTTTTTGACTCTTG		NA
Ca-II-SNP1029	70.68	Ca-*desi*-LG(Chr)6	4459719	AGCTTTAGCAATGCCCCTTCCTGTAGGATTTGTTAATGGAAAAACTTAGGTCAAGTGTCTAAGATATTGTTACTGTTTTCCCATAAAATTACCCAGTAGA	T	C	ACAGAACTTAATATACTGTGTTTAGTACTATTTTTACTCTTTTGTTTTTCACTTTTCAACTTCCAGCAAGCAGATCTCAATGGGTGCACCGGATGAAACC		NA
Ca-II-SNP1030	71.52	Ca-*desi*-LG(Chr)6	4472854	TCCCTTATCTTGAATTTTTTTGAAGATGTTGATGTTATAGGTTGGATAATTAGTCTGTTTGGATTAGCTTATTTTAATTTATCAAATGGCATAAATATTT	G	A	TTTTACATGTTATTCATCTTAGAAAATTTATAAAAATTAGTTAAAAATAGCTTATGAATATATCATAAACTGTTTCTACAAATTTTCTCAAACAATCTAA		NA
Ca-II-SNP1031	72.36	Ca-*desi*-LG(Chr)6	4485447	GATATAATTTTCCAATTTTACTATTCTATTTATGAATGCTGAGACTTTATCATTGTTATTTTCTTTTCAAATGTTTGCGCATGAAAGATTTGTGCAACTG	T	C	CAGAAACATCTAATATAATAAAAAGAGAAAGCAACAGAAAGTTAATGGTATTATGAATTAACAAGAGTTGAAAGACAGAGTGTGAGGGAGAATATGGTGATGATGGTGGTGGTGATGATAGGGTGTAGATTCTTTGTGAATTCGCCAACGGAGATGAAGAAAATCATCAACGGAACAAGGTAGCTTGAGGGGACCATGAGCGTTGTAGCCATAAACATCGTAAGCTTT[illegible]		NA
Ca-II-SNP1032	73.20	Ca-*desi*-LG(Chr)6	4514928	ATAGCTGTAACAACTGCTCCTAAGAAGTTACCTTTTGAGTTCAGAGCACTTGAAGCTTGTATTGAATCTGCTTGTTCTGTTCTTGAATTTGAGGTATGTA	G	A	AACACAAATCATATATAGAACTCATAATAAACATACAATAAATTAATATCAAAGATAATTTTATATATAAATGAACAAAATAAATAATGAAATTATTACA		NA
Ca-II-SNP1033	74.04	Ca-*desi*-LG(Chr)6	4528042	GGTGTCAACTGGCAAATCATTTTCAACACGCACCGTTGTTATTGTTGCCTTCACGCTTGTGATCATCAATACAAGAACAATAGCCAACACATTATTCATT	G	A	CAAACTTGTTGGCCATGGATTATAAATAGACAATTTAGAGTATAGGATAATGCATAAAACTTGATTTTTAAAATTGTGTAGAGTTGAGTAAAAAAAATGA		NA

Marker IDs	Genetic positions (cM)	Chromosomes	Physical positions (bp)	Flanking sequences/Forward primers	ICC 12968	ICC4958	Flanking sequences/Reverse primers	Annealing temperature (0C)	Amplified Product size (bp)
Ca-II-SNP1034	74.88	Ca-*desi*-LG(Chr)6	4567143	TTTGGGAAGGTTCTTGCCTTTCAACATACTCCTAACCATGTTCATTATGGTTCTGTTCCTCCTTTCAACAACACCATTGTGTTGAGGTGTGTAAGGAGCA	A	G	GGAAAACAATTGAAGATACTCAGAACTGATGGTGGAGGGGAATTTACTTCCTCTGAACTTGAAAGATTTTGCACTGAAAATGGTGTTGTGCATGAAGTGA		NA
Ca-II-SNP1035	75.72	Ca-*desi*-LG(Chr)6	4683999	GGTGATAGAATTCAAGCAACAGTGCGAAAAACTCTTATTCCTCGATTTGAGACTACTATTCGTGAGGGAGTTGTGTACAACTTTCGTTCTCTCGGTGTTG	C	T	GGCTGTGACCAAACTGGTTTCTAATTCTCGGACCATTGTACTATTTTGCAAATTCAATTTGAATTGATTTCCGGTTGTTCTATATGCTCCAGAGTTAGAC		NA
Ca-II-SNP1036	76.56	Ca-*desi*-LG(Chr)6	4756280	ACTGCTTCTCCCCACAGAGCTTTGGGAAGGTTCTTGCCTTTCAACATACTCCTAACCATGTTCATTATGGTTCTGTTCCTCCTTTCAACAACACCATTGT	A	G	CAGAACTGATGGTGGAGGGGAATTTACTTCCTATGAACTTGAAAGATTTTGCACTGAATATGGTGTTGTGCATGAAGTGACTGCTCCTTACACACCTCAA		NA
Ca-II-SNP1037	79.92	Ca-*desi*-LG(Chr)6	4763248	TCCTCTTTTTGTATGGGATAGATATCAAACAATGCTATCAAAGGCGGACTATGGCGGCCAGCCTAAAATTCGCCAAATAAACATGGCATTGCTGCCATGG	A	G	TTCCACCATGGTGGCACCATGGCCTTATGTCGTGGTGGATTTTGTGTCAGCCGCCATACGTTGCAAGTCTGAAACAGTTGTTATATCAGCCATGTTTCCG		NA
Ca-II-SNP1038	80.76	Ca-*desi*-LG(Chr)6	4767445	TTATGGTTCACCACCACCTCACCTACAAAGCCTTACAAAAGCTCCATATTATGGTCCACCGCCACCAGATGCACATATCCTTGCAGAAAATGTCGACTTC	A	G	ACTTAGGAGTTGTGCATTGAGTCCAAAATAAATTAAAGAAAATATCGCGTTTAGTTAAGTGACCGGGGATTTGTATTTCAGGCAATGAAATAACATAATA		NA
Ca-II-SNP1039	81.60	Ca-*desi*-LG(Chr)6	4782646	GATATAATTTTCCAATTTTACTATTCTATTTATGAATGCTGAGACTTTATCATTGTTATTTTCTTTTCAAATGTTTGCGCATGAAAGATTTGTGCAACTG	G	C	ACTTTTCGCGGATAATTCAAGTCGTTGGCTAATAGCAGATCAAGGTGTGTCAATCATTTTTTTTTCTTCCACTTTCACTGTAGTGTTTAGGAAGGAATTA		NA
Ca-II-SNP1040	82.44	Ca-*desi*-LG(Chr)6	4787645	AAAGATGATCCATATACCATATTTGTTGCCTTTTTATTTGTTAGTAGACAGAGTGTTAAAATGACTTAAATAATTTTTCACTTCCCACTCTCTACCTGTA	G	T	CAAATGTATGAGGAAAAAAGTGAGTATAAAAAAATTTCAACTTTATATATAGTAAAAAAGGATTAAAAAATATATTTTTCAATATATTGAAAGGTGAGAC		NA
Ca-II-SNP1041	83.28	Ca-*desi*-LG(Chr)6	4792295	GACCCGCTATCCCAGCCTGAACCGCTCTAGGGCTTTTTAAAAATGCAGATTTTGTTTTTCTTCCATTTTTCATTATAATAAACCCACGAAGGGGTTGGTT	T	C	AACGGAGCAAGAAAAGAACAAAAACAACAAAGCAAGAGCGATGGTGACGGAGCAAGAATGACCAGAGGAAGAACGAAAACAATTTCTTTTTCATGATGTC		NA

Marker IDs	Genetic positions (cM)	Chromosomes	Physical positions (bp)	Flanking sequences/Forward primers	ICC 12968	ICC4958	Flanking sequences/Reverse primers	Annealing temperature (0C)	Amplified Product size (bp)
Ca-II-SNP1042	84.12	Ca-*desi*-LG(Chr)6	4799707	TTCTTCATGCTGAGGATTAATACTAATAAGTAACTTTGTAAGTTGAAGCTGTCTGTGGATGTCATTGTCAACTATTCCACAACATCTTCTCTTACGCATC	T	G	AACAACAAAGTTTCAAGTTAGCTGCTAGTAACTCAGTAAAATGATGAAACAGCACTCAGTGTCAAGGTAACATAATTTCAAAATGCCAAAATATAATGTA		NA
Ca-II-SNP1043	84.96	Ca-*desi*-LG(Chr)6	4807428	AGCAAGTCCTCTATGATTTGCATTTTCAACAAAATATCCAGCTGCTGTGGAAGCTGTGTGAGTTCCATGGCCAATAGCATCTCTAGCTGAGAGATATTCT	A	G	TGTCAAGTAGGACAACACTTCAACTCAACAAACTGCAACAAGAAGATAATTGGTGCTAGATGGTTCCTGAAAGGAATAAGTGATCACACTAATCACACAT		NA
Ca-II-SNP1044	85.80	Ca-*desi*-LG(Chr)6	4811236	TGCAATTTTATTCTATTCAATTTTGTTTATTGTATTAAAATTGGCTACACTCCACGTGGAAATTTTATTTATAATTTTCTGAATCGTACATAAGTACATT	G	C	CAAAAAAGAGAAAAGAAAGAAATGTATTTTAATTTGATTGATGTAATGTTACGCAAATTACACGTTGGAAATATCATGTGTTTAACATTTGTCTAACACT		NA
Ca-II-SNP1045	86.64	Ca-*desi*-LG(Chr)6	4818263	ATAATGGAGGTTTAGAAGGGATGGTACTACTTGATTGGGTTTGTGAGAAGGACATGCCATTAAATATTGTTGAAGTGCCTTCATTGTCTTGCCACCTTTG	T	C	CAGTGAATGTTTTGGGTCCCCTTGTGGAGCTATGGAAACCTATCACCCATACAGAAGAATACAGTTATGGCATTGACAATCAGAGTCTTCCTCAAGCACT		NA
Ca-II-SNP1046	87.48	Ca-*desi*-LG(Chr)6	4822927	ACGCTCTTCGTTGACTAAATATTTTTTTTCAATGAAACCAGTTTTCAATTTCTTTGTACATCCTCATTGTGATTTTTCACTTAAGTCTTCATTCTCCTCC	T	A	TCATACTCATGTGTAACGTAAAACATACTCATGTCTACATGATGGATATGAAGGGCCAAGGAAAAAAAAACATAATAAGTGGTATCAAGATATTCAACCA		NA
Ca-II-SNP1047	88.32	Ca-*desi*-LG(Chr)6	4930794	AGTAACTGAAGTTGACGGTTTAAATGACCTGTAATTTTGGGTTGATGAGTATATGATGCTGATTGAGTTTGTTAGGCAGGTTCATCCAGACATATGGTAC	T	A	CTAAATGTCTCCTGATATCACCGGGGGGAATCTTTGATGAATGTTTTTGTTTGACACAAATTACATCTTGACCTCCAACAGACATACCTACTACTATGTG		NA
Ca-II-SNP1048	89.16	Ca-*desi*-LG(Chr)6	4939660	TGGATATTGAGTCAAATTTCAAACTAATAATATGCTTATTCGTGTTTGGCTTTATGATGCAGGTGTATTTTATTAACCACAGATACGACGGACTTCAGCT	G	C	CCTCTAAAAGGTTAAAGGTTGAAGGATACAAGAAAAGGAAGGAGATGAAGTTAGAAATCCACACAAGTTGATGAACTCCAGGAACACTTTGGAGCTGCCA		NA
Ca-II-SNP1049	90.00	Ca-*desi*-LG(Chr)6	4996054	TGCATTCAACCTTCAATGTACATTTTTCTTGGAATTCGATCATCTATATGTTATTGTAGCAGTTGTCCATGACCACTTGTGCTGTTGTTTCATGGCAGGG	C	A	CAGAAAATACTAAATACTATTATTATGCTTATCTGTAATCATGTTCTAAAGACTAACCTCACTAGTGTCTCTGTAATGCACGAGAACAATATGGTCAAAT		NA

Marker IDs	Genetic positions (cM)	Chromosomes	Physical positions (bp)	Flanking sequences/Forward primers	ICC 12968	ICC4958	Flanking sequences/Reverse primers	Annealing temperature (0C)	Amplified Product size (bp)
Ca-II-SNP1050	90.84	Ca-*desi*-LG(Chr)6	5007515	CCATTTCTGGCATTTGAATATCCATGAAACATGCG<br>TCGAACTGATGAGGTGGCTTCAGCGAAGAGATAG<br>CATCTTTTCCACTGCTAACACAAACCACATC	T	A	TGTCTCTTAGCCATCTTCTTTTTGGGAGGAAAA<br>TTCTTATTGTTGATGACAATGGCGTGAACCGCG<br>CAGTAGCAGCCGGTGCTTTGAAAAAGTATGGA<br>GC		NA
Ca-II-SNP1051	91.68	Ca-*desi*-LG(Chr)6	5010930	GCTCCAAAGTATCCATTCTTTTAATAATCCAACCTT<br>GTTGCTTCTCAAATTGTTCCCTTTCAGAATGTAAC<br>ACTCTTACAGCATATGCAACACCACTTGT	T	A	GGTGATAGTGTCAACTCAAGTGCAGAGATGAAT<br>CTAACTTGTAAAACCTACAGAGAACATTTGCTA<br>GGTACACAGAAAGGACAGCTTTTGAGAGGCCT<br>CT		NA
Ca-II-SSR330	92.52	Ca-*desi*-LG(Chr)6	5013080	TTGTTTAGCAGTGAAATTAACGGT	(TA)10	(TA)11	TATGAACACAAAGCATATAAAAGTTGTGATTTCT<br>GCTCAGTGGAAGTGGAACCATACCTTTCTCTTC<br>TCTACATTATTAACTCTACCTTACTAAAAGTAT	TCTGCTCAGT<br>GGAAGTGGAA	59.19
Ca-II-SNP1052	95.04	Ca-*desi*-LG(Chr)6	5022799	GATTAACTATGAGTATATTCTATCTCACTTGCACT<br>CAACTATTAAACCTCATGTATTCCTGCATGTGTAA<br>ACATGTGTGATCAGTGACCCATTATAACTC	A	G	TTTAGAAAGATATGATGTAAGCTCAATGTATTAA<br>TGTTGGGAAAAATCAGTTGACCCGACCGAAGT<br>CGAGCCACTCGAATAGGAGAATTTGATAGTTG<br>GA		NA
Ca-II-SNP1053	97.56	Ca-*desi*-LG(Chr)6	5054753	TTCTCTCACTTTGTTGCCCATTTCTTTTCTTCCATC<br>GCTTCCTTCTTCTAAATTCACTCCACTACTCCTTC<br>GAAGATCTAAAATTGGAATTTCACTCTCA	G	A	AGTGTCTAGTCACTGTATATATATATAGATGTAA<br>CTTGTGAGAGAAAAATACAACTACAATCCACAT<br>ATCGAAATACGGTAGAAGAAAGAAAAATGACTA		NA
Ca-II-SNP1054	98.40	Ca-*desi*-LG(Chr)6	5069429	TAATGTGGGTCACTAAGATAATTGAGTCCTAATTG<br>GTAAAGGTGGTCTGACCAAACCCCAATCAGTGTC<br>ATGATTGTTCTCCTTTACCTGTTATTCCATG	T	C	TAATAAGTGCATTTTATACAAAATATTTCAAGTT<br>TTGTACAATATCATTAATTAGATCATGCATGACC<br>TTATTCACGGTGGGACATTTAGATTATTCCAG		NA
Ca-II-SNP1055	100.08	Ca-*desi*-LG(Chr)6	5557511	GATTAACTATGAGTATATTCTATCTCACTTGCACT<br>CAACTATTAAACCTCATGTATTCCTGCATGTGTAA<br>ACATGTGTGATCAGTGACCCATTATAACTC	T	C	CGAGAAGAGTTGAGTGCAAAATGAAGGGACCC<br>GAAATATCAAATTGAGAGAAGGCAGTAGTAGTC<br>TCTGCAACAAACAATAACACAATATGGCTTTCT<br>CTGCAACAATTACTTCTACGCACTATTCTCCCC<br>AATTCCTTCGCGTTCCCATCTCCGTTCCACTTT<br>CTCAAACTCACACATCACTCTCTTTTCGGTCTT<br>CAACACCAATCCTCAATCGCCGAATTCGTACAC<br>[illegible]		NA
Ca-II-SNP1056	100.92	Ca-*desi*-LG(Chr)6	5624131	TTCTCATGCAATTTAGTTTAGTGACTTCTATTCTCA<br>CTGACGATCATTCTGCATGGCAATTTGTCCCAAAT<br>GCATTGGCCCAATCTTTGTCCCAGATGTA	G	C	AGTTGAACAAAGCAAGATTCGTCCTTCTAAAAA<br>CTGCATTTGGAAACTTCAAACCAATAATATTAG<br>AGATGCGTGCTTCAAGGAGAGAAAGACAATGC<br>CA		NA

Marker IDs	Genetic positions (cM)	Chromosomes	Physical positions (bp)	Flanking sequences/Forward primers	ICC 12968	ICC4958	Flanking sequences/Reverse primers	Annealing temperature (0C)	Amplified Product size (bp)
Ca-II-SNP1057	101.76	Ca-*desi*-LG(Chr)6	5663010	GATTAACTATGAGTATATTCTATCTCACTTGCACTCAACTATTAAACCTCATGTATTCCTGCATGTGTAAACATGTGTGATCAGTGACCCATTATAACTC	G	A	AAAGTACAAAAAACTCGTGATTGTGTTCTTTATCTTTCTCTTCTTCATCTTCTCTATCTAAAACAACTTCTATTAGCTTCTTCATTCCACTACCAATTTTTCTCTTATCA		NA
Ca-II-SNP1058	102.60	Ca-*desi*-LG(Chr)6	5722880	GAATTTTCGATTTGTTGTTGTGTATGTTTAGGAAATGATGTTTCTAACTTGATTTCATCTTGTTTATATTTATCCTCATCGACCAATTCTTGCAGATCTT	C	A	TTTTAGGAATTACTGAGTTACTATAATGATAATTGATAACTGAGGAGTTGAAGTGTTGTACGAGGTGCGAGTATTAGTGATTGAAGATGAAACAACTATC		NA
Ca-II-SNP1059	103.44	Ca-*desi*-LG(Chr)6	5737988	ACTTCATGCACTTAGAATTTTCTCCTGCCTCTCCAACATAGAGTGGATAATACCTAGATAACTCCTCAAACTTAGTGGCATACTCCACTACTGACATATT	C	T	CAATTACTTGGGCTATATTCAAGAACATGTTCCTAGTCAAGTATTTCCTTAAAGACATCCGCTATAGGAAGGAAATGGAATTTGTCAAATTGGAACAGGG		NA
Ca-II-SNP1060	104.28	Ca-*desi*-LG(Chr)6	5748573	TTTCACTAATATAATCGATTTGGCAATGAGCTAATTTCTCACTGTAACTCAGACACTTAATCAAATCGATTTGCCAATCGACTGATTTGTCCCTGTAACT	A	C	ACTAATCAATTAGTTAATCGATTGCATGAATGAATTAGTTCATCATTTATCAGAAACATATTGCATAATCGATTGGCACATCGATTTTAATTAAATATCT		NA
Ca-II-SSR331	105.12	Ca-*desi*-LG(Chr)6	5762498	CGTTTTGAAAAATCGGTGGT	(TG)7	(TG)6	GTTCCTTATTCTCCCTCTCACGTTCCCTCCAGAACATGATTTCCAAACCCTCCAGAACTTTTTAACAATAACAAACAACTACAAATTCAAACTCAAAAAT	TCTCACGTTCCCTCCAGAAC	59.84
Ca-II-SNP1061	105.96	Ca-*desi*-LG(Chr)6	5766119	AAAAGGCTTCTCTCCATGCTGCCCATTTTTTGAGTATTTTTACTTTATTTCAAACCGCTTGAGTATTTATTAGCATTACTAGGGAACACACAAGTATTTC	T	G	AACAAGTGGAGAAACCGTTAAAAAAAAGTGCAGAAAGAGAATCCTCTTTTTCTCCTACTATTCAACCTAACGAAATAAAAGAAACCTTCTAAACATAAGT		NA
Ca-II-SNP1062	107.64	Ca-*desi*-LG(Chr)6	5794645	CCACTAGTTTATAGAGGACAGCTTATAGCGTGTGAACTCATATGCCCAAAATAGAGCTTCTTGGTAACCATTTTTTGTTCGGAACCTACAACTGTTTCAA	T	C	TGCTATAAAAAGATAATTTTAATTAATATATACATTTTTTCAATTAAGATGATGAAGGTGAAAATGAGAAAAGATGTGATATGTTATAAGCTCAAACTCT		NA
Ca-II-SSR332	109.32	Ca-*desi*-LG(Chr)6	5830798	GGAGACCTTTCAATTTCGATG	(TAT)11	(TAT)10	ACCAATTTTATCAGATGGTGGAAAAGGTGATACAACCTGTTACAGAAAGAAGTTGAGATAGTTTTACAATTAAAATCACAACTTAAAAATAAATTAATAA	TCAGATGGTGGAAAAGGTGA	58.63

Marker IDs	Genetic positions (cM)	Chromosomes	Physical positions (bp)	Flanking sequences/Forward primers	ICC 12968	ICC4958	Flanking sequences/Reverse primers	Annealing temperature (0C)	Amplified Product size (bp)
Ca-II-SNP1063	110.16	Ca-*desi*-LG(Chr)6	5856064	CTTGATAATGAGTTGTTAATCCTCTAACCTTTATTTTAGTCCAAGAGGACCTTGTTGTTTTAGTCTATACTTAAATATATGATTAGTCCTTATAAATATG	A	C	CAGCCTTATTTCAAGCACAGAGCAAGTCACAAACCTCAATGTGGACAACGGTGGTACTTACTTATCAATGAAACCAACAAACAATAATCACACCTATGACCTTTGAGTTGGAGCAGTTTGAACATACGACTAAAGCAAACCCTGATATCACTATTTTACGTAATGAGCAAAAAAATTTCACTTCAAA		NA
Ca-II-SNP1064	112.68	Ca-*desi*-LG(Chr)6	5970165	ATACTGTTTTGTGTGTTGAGCATAACTATGACTCTATCTGATGTTGTTGGAGGTTTGTTTATGTAACTTTGGTGAGCTTTGATTTTAAGGCTTTGAGCTA	A	G	ACCTGGTTTAGCCATTTTCAACTACTATCCCAACCATCCAATGAGGTCACCACCAACACCTCCACCATCTCCACCAGCTTGGGACAATGTTGATTCAAGG		NA
Ca-II-SNP1065	113.52	Ca-*desi*-LG(Chr)6	6096482	GCATTAACTGCATTTTCCATAATGTGACAGTCATTTGTCTTAGACTGGTTTTAGATCAAATATTTGTACTATTATTTATATAGATTCTAGCTAGGTTTGA	A	T	TTCAATATGAAAAATGAAACACCTTTTCTATACATGGGACAGAGGATACCTTCTTTCTTATTTTATATCACCAAACTGCATGTGATGAGAGAACTCAATT		NA
Ca-II-SNP1066	114.36	Ca-*desi*-LG(Chr)6	6118607	GTGTATGAATTTTCCTAAATTATCATTTTGTATATCAACGTGTACAATTCTGAGTTTTCATCTTCCAGGAGACTTAGTATTATTAATTAAGGGTATATTT	G	A	ATCAATATTCAATAGTACCTTTGTCCCAATTTATTAGCCAAAAAAATTGTCTCTAAATATAACATTCATTTCAAATTCCTTAATATATTATTATTTTTTTC		NA
Ca-II-SNP1067	115.20	Ca-*desi*-LG(Chr)6	6123476	ACCTTCTTCTTTTGTTCTATATCCACTAAATTCCACTTTATCCTATGATAAATGTGCCCCTACTTACAACAGTTTTTGTCTTAGTGTTTCCTCCATCAAA	G	T	ATAATAGTCCAAGTGTTGGTGGTCTGCAATGCCTCTAATTCATAATGCATGGCTTGCTTGCAACATTCATGCTTAGAGGATTGAGTATAGGTAAAGGATT		NA
Ca-II-SNP1068	116.04	Ca-*desi*-LG(Chr)6	6149552	TGATAGTGGAAATTTAAAAAGAATCGGTTATTCGCTTGAATATTGGTGTCATTTCAATGTTCTATGTCTTCTCACCCTAATCCTTTTAATACCTAAAATT	C	T	TGAAAAAAGAGTGTTCTTAACACTTCTCTCAAAGTAATAACACCTTAATTTATCTCAAAGTAATAACACCCTAATTTAACTCGCAAACGAACTCAAAGAA		NA
Ca-II-SNP1069	116.88	Ca-*desi*-LG(Chr)6	6270775	CATTCCTCGTACCTTAAATAGTTTTGTCCTCGAAACTGGCATCAGAGAAAAGTTGAGTATTGCTCTCTCATTTTGTCTTCTAGTTCCCAAGTGGTGTCTC	T	C	ATCTTTTGAAGTACCTCCGGCAAGAATTGAAGATAGAAAAGTCAAGTTGCTTCGCAACAAAGAAATTCCCTTGGTGAAAGTAGTTTGGAATCAAGCCACT		NA
Ca-II-SSR333	117.72	Ca-*desi*-LG(Chr)6	6378408	TTTTAGTAGTCGTGTGGCAATG	(AT)6	(AT)7	TATTCAAATAATACAATCGGTCACTTATTGTAAAACATTTATAATATAACTAACAAAAACCGTGAAAAGAAACATTCATAATACTCTAATATTTTGTATA	AACAAAAACCGTGAAAAGAAACA	57.92

Marker IDs	Genetic positions (cM)	Chromosomes	Physical positions (bp)	Flanking sequences/Forward primers	ICC 12968	ICC4958	Flanking sequences/Reverse primers	Annealing temperature (0C)	Amplified Product size (bp)
Ca-II-SNP1070	119.40	Ca-*desi*-LG(Chr)6	6386917	CTTGATAATGAGTTGTTAATCCTCTAACCTTTATTTTAGTCCAAGAGGACCTTGTTGTTTTAGTCTATACTTAAATATATGATTAGTCCTTATAAATATG	A	T	CTTCAATACAAACACAAAATTCACCCCTCATAAAAAATGGATTTCAGTAGCAATGAGagTGAAGAAagTGAActGTATCATGCTCAAATCCATTTgTACAAACATTtATAtAGTTTTATAAGCTCTGCTGCTCTCAAATCTGCTGTGGAACTTGGCATAGCAGATGcAATCCACAACCATGGAAAACCAATGACTCTTAATGAATTAGCTTCATCTTTGAAACTTCACCCTTCTAAAG[illegible]		NA
Ca-II-SNP1071	121.08	Ca-*desi*-LG(Chr)6	6402258	CATGCTATTGGGTTTCCTCGCATTTCTCCTTCTAGGTTGATTGCAACATTATTTGGTCAAGTAATACTATATGTGACGTCCTATCAAATGTGGTCACGTA	C	T	AGTTGAATAATTTTGGACGCTCTGATTCAGTGACCTATTATGTTACTAACTAAGGTGATGGATATTCATGCATGCATTGGTGGTCTTAATCTAGTCGCGA		NA
Ca-II-SNP1072	123.60	Ca-*desi*-LG(Chr)6	6611519	CCTGCTTCTCTCAGGAAGGATGCACTCTTCGTGATCTCACGCAATGGCTTGTTAGTGTACCTCACAGTGTTGTACACCCATGCTCCGGCCACCGCTCCTA	A	G	ACCAATAACAGGAGCATCAATGAATCCAGCTAGAAGTTTAGGACCTGCATTTGTACACAATAAATACAGAGGAATATGGATATATTTGGTGTCACCGATT		NA
Ca-II-SNP1073	124.44	Ca-*desi*-LG(Chr)6	6699208	AGCTGGAGTTTTTAAAGGTATGTGTCAAATTCTTGCAAATGTATGCCATTGCACTTTTGGGGGACTAGAATAACCTGTACTTTGTATTTTACTGCTCAAA	C	T	AAAGTTGACAAAAAATTGCAGTTTACCTTTGTTCTGATACTTGTAAAGCCACCATTATTTGCCGTTGAAACAACACCTGGAATACACACAAGACTCTATT		NA
Ca-II-SNP1074	125.28	Ca-*desi*-LG(Chr)6	6719297	CTTGATAATGAGTTGTTAATCCTCTAACCTTTATTTTAGTCCAAGAGGACCTTGTTGTTTTAGTCTATACTTAAATATATGATTAGTCCTTATAAATATG	C	T	GACTAAAATAAAAAAATAAGAAACAAAAACAAAAATATTTACATAAATCAAAATCAAACAAATTCTTTTTTAAATTTCAGTGATTAAAATTAAAAATAAC		NA
Ca-II-SNP1075	126.12	Ca-*desi*-LG(Chr)6	6725031	TTTTGAAGTATGAACGTTTCCCACCACTAATGGCTTAATATTCATGTCGCTCTGCATCTAAATCATCTCAAGACTATTGTCATTGATATGGGTCTTGTCA	G	T	AGGTCTTTTAGTTTAAGATCAAAAATTACTCAACTTCAAAATGAGGGATTTAAGTTTCAAAATTACTCAACTTTCGTCAAGTTTCAAAATTACTCGACTT		NA
Ca-II-SNP1076	126.96	Ca-*desi*-LG(Chr)6	6735402	TATCTTCAATTTTTCTAGTTTTTAATATATTTATTCTTGGTATTTTTTAACTTCTGGATACAATAATTTGTTTTTTTTTAAATAATATTCTTTTGAAGCT	C	T	GGGTGAGAATTGAGAAAGCATGAGAAAATAAAGGAATTGAAAATGTCAAATATCAAATAAGAAAAATTATTTTGTGGTTTAAGAATTTGAGAGTGTTCAA		NA
Ca-II-SNP1077	127.80	Ca-*desi*-LG(Chr)6	6741902	GAGGAACGGGTGGCACGGAAAAGGGAAGAATGGTTAGGACAGAGGTTTTTCTTTCTCCGTCGGTTCGAATTCGCACGGAAAACGAATTTGAGGAGATGCG	C	T	TTATATTTTTGATTGTATCATAAAACTTGTCTATTTTAAAACTCTGTTTAGAAGACGCATTATTTTTCCTTATGCTCCCTCGTCTATCGCAGGCGAAGCT		NA

Marker IDs	Genetic positions (cM)	Chromosomes	Physical positions (bp)	Flanking sequences/Forward primers	ICC 12968	ICC4958	Flanking sequences/Reverse primers	Annealing temperature (0C)	Amplified Product size (bp)
Ca-II-SNP1078	128.64	Ca-*desi*-LG(Chr)6	6744604	CCAAAGCGAATATGCTGTTGTATCTTCTTTCATATGTAGCACTAGCAGTGAGCACCACTGCACAAGCAATACGTCTTGCATTCTTTTTTTGGTCAACATC	G	C	TATGTGAGTATATATTCATATTCATAGAGATCGAATCTGATCTTTTTGTGGGGAGAAGAAATTCCTTGTATAGTTGATTATGTAGGTTGAAAAGAATACA		NA
Ca-II-SNP1079	129.48	Ca-*desi*-LG(Chr)6	6753701	AATGTTTATACTTGAGATTAATGAATCTTATTGTACAAGTATGGTGAATTAGCATGCATCGGTGTATGTCGTGTATCTCAGATTTGAGTCATTTTATTGG	G	A	TCTTTAAAGAGGGGTACAACAGTTGCCTAGTGCTTATATACTTTGTCTTCTGTCATGAGCAAGCTGTTGCCAGTATGCCATCCATGCATTATGGTCTCTA		NA
Ca-II-SNP1080	130.32	Ca-*desi*-LG(Chr)6	6764579	TATTTCTTAAATTATTTATAAAGTTATTTATTATCTTTTAAAATGTTTACAAAGTTACTTTCTATCTTTTTTTCTCTCATTAAGATGTTTTATCTTTTAA	T	C	TGAAATCTTATATATAAGAATGAACATAATTCTACATTAACTTGTCAATAAGCATTTAAAATTACTTGTCAACATTTTAAAGGGATAATTTTGTAGACAT		NA
Ca-II-SNP1081	131.16	Ca-*desi*-LG(Chr)6	6783139	AGTGTGATGACAGTTTTTATGATTCCATCAAGCCTCAATTTATTCTTCTTCCTGGTCACATCAATAGATATCTGAACAGGAGTTCCATTCGGATACTCCT	T	C	ATACTGCACTACTTTGGAGAGATTAGGTCTAGGAAATCTTTCAACTGATATTTCGAAAAATAAGGCGTCTGTAATGGGATTGCGATTTACGAAAGCAGCG		NA
Ca-II-SNP1082	133.68	Ca-*desi*-LG(Chr)6	6790995	TGGTTTTCTTTATTATGAGAAACTTTCTATGAGAGATTTCATGGTAATTTCATTGGTGGGTATGTAACGGGCTTTTGATATTCTAGAAATAGAATATTTT	G	A	TCAAAATAAGGACTTTATTGATATAGACATGAGTTCATTTGCTAAGACATGTAGACAACATGTACATGATAACGCATCACCTAACTGAAATCGAAAGAGA		NA
Ca-II-SNP1083	134.52	Ca-*desi*-LG(Chr)6	6800800	TAACTTAACTAATTGGTTAACTAAAATGAGGTAGTTATAGCGTGTTCACAAACAGTTTCGAATTATTTGTGCAACCATCGTCATTTTGATTTTTTGTTTA	A	G	GCAAAAAACTAATAAGCACCTCAGTTCGGCCAGCCAATTAGTTATGTTACAGATGAGCCATGAGTGAGTTAGATCTTGAGTTGGGGAATGTTGGAATAAA		NA
Ca-II-SNP1084	135.36	Ca-*desi*-LG(Chr)6	6815781	TCTTTTTGACCTTATGAGCAATTCCATGGCGTTTCTGAGATCACCCATTTCGCATAACTTGCAAATTTTAGCATTTTGGTCAGCGGTTACGTTGTGAGTG	A	G	TTCATCTTCTTCAAACAGCCTCGTACACCATCTCTGCTACATTCCAAATTGAACATTTTTTGTCGTAGCAGTGTCGGCGTCTCTTCATCTTTAGCCAACA		NA
Ca-II-SNP1085	136.20	Ca-*desi*-LG(Chr)6	6825600	ACCCAAATCGATCATGTTTGGGTTGGGTTAAGTTCTAAACCTGTATTTTTTCATTTAACCCGAACTGACCCAACCCGATCAAGAACGGGTTGGATTGACG	G	A	ATTGAAATTTTATAATTTTTGAGACATTCAAATATTAAATATATTTTCATTCAATATATTTTCCCGTAAAATAAAGAACATGTTATTATTTATGTATGAC		NA

Marker IDs	Genetic positions (cM)	Chromosomes	Physical positions (bp)	Flanking sequences/Forward primers	ICC 12968	ICC4958	Flanking sequences/Reverse primers	Annealing temperature (0C)	Amplified Product size (bp)
Ca-II-SNP1086	137.04	Ca-*desi*-LG(Chr)6	6839524	TGTCATATTTAGTGTCAACCCCCGATACCGACGCTAGTTAGCGTTGGTGTAGCGTCGGCTGAGTCGACGCTAAGTTAATTTTTTATTTTTCTATATTAAT	T	A	GGCTCGTGTTCGAATTAGAATCCGATCTTACTAATATGATTTTATTAAAAAATTTAATATTTAAAAATCATAAACCTAGCGTCGGCCCGTCGACGCTAAC		NA
Ca-II-SNP1087	137.88	Ca-*desi*-LG(Chr)6	6843142	GCATGGAAGTTCCATCCTCGGTGGGATTCTCTGTCCTATGCTCCCCAAAATCAAAAGAAAAAAATATATTAAACTATTATCCTTGCCTATCCATTCTGTC	C	T	GAGTGTTTTTTAAATTTCATTTTATACAATTTAGGATTTTTGGTGTAATAAAACACATGGCAATGTGGGAAAAAGTAAAAGGTGACAAGGACGAGAAAAT		NA
Ca-II-SNP1088	140.40	Ca-*desi*-LG(Chr)6	6850697	AACTTCAGTCACAGCTCCTTTTTTCCTCCAATCTATAGAAGCAGGAAGGTTGTCACAATCATCATCATCATCTTTAAGTTTTCTATCATCCCAATTAGAT	G	C	AAGAGAAACTCACCTTTGCAACATCGTTTGGGTCTCAACAAGTTTGGTGATATGAGTCCTGAGGAGTTTAGTAAAACATACTTATCAGAGATTCAGATGA		NA
Ca-II-SNP1089	141.24	Ca-*desi*-LG(Chr)6	6853416	CATGTAACATTGCATCTCGTACTTTTTGGTTGCGATTTCTCAAAAATGATCTGTATTGATCACATGTTAGGTAATTTGAACCTGTAATTCGATGTGTGAT	C	G	CAAAAAAATTTTTGAAACATTGACAGTTTCTAGAGCCAATTCTTGAAAGTAAAGCAACTATTCATTAGTTTAATTTCCTTTGAATTGCAAATACCAATTT		NA
Ca-II-SNP1090	142.08	Ca-*desi*-LG(Chr)6	6866046	AAATACTAACGTTCCTATCATGAGAATGAATATGGCCACAATTTTTTTTTGGGTATGGCCTATTTTTTTTATAATTAATATAAGTTTACATTAGTTGGGT	T	A	TTACAAGACTTTTAATTGATTCACACTATTGATGTAAATAATTTTATAACGTATATCTTCTTTAAGTCTTGAATTCTATCTTAATTTAATGAACTACATA		NA
Ca-II-SNP1091	142.92	Ca-*desi*-LG(Chr)6	6872681	GGATCCCATTACCATCTTTCACAACTACTACAAAACTTAGCTACTTTCTTCCATTTTTCATTATTACCATTTAACATACACTATCTTGTATCACATCTCA	A	T	TACAAACCAAATAATATTTGTTTGTAGTGGTGGGTTAATTACCTGAAAAATGAAGAACAAAAAACAAGGAAGGGAATAAGAATATGTAGTGTGTTGAGAT		NA
Ca-II-SNP1092	143.76	Ca-*desi*-LG(Chr)6	6875034	CTGGAGGAGCTACATCTTCTGCTGGGAGATTGGTGATTGAATATGGTGTTTTGAAGGTATAAATCTGTCAAAAGTCTCATATTTTTTTCACTCTTTTGAT	A	G	GGTAATGACCGCTGTGAGGCCAAACAGCCTGTTTAAAAGAAACAATCAAATCATTCTTAGCCGAACACATAAAATCGAAAAAACTAGCCACAAATGAGTC		NA
Ca-II-SNP1093	144.60	Ca-*desi*-LG(Chr)6	6886340	CATCCACTTTACATTATTATATTGGAATCAACAAATAGTTTCCAAAAATAGTTTGGTAAACTTGTTCTTCTTCTTTCATTTATTATATTTTCTTAATCTG	C	T	GAGGATATTTGCTCTTCAATGTTATTGTGACATAGGGATATTAATTTATGACCAATATACTCCTCCGTCCCTAAATAATTATCATATTTGTTTATTTTAT		NA

Marker IDs	Genetic positions (cM)	Chromosomes	Physical positions (bp)	Flanking sequences/Forward primers	ICC 12968	ICC4958	Flanking sequences/Reverse primers	Annealing temperature (0C)	Amplified Product size (bp)
Ca-II-SNP1094	145.44	Ca-*desi*-LG(Chr)6	6896141	TATTGCATTTAATAACTTTTTAGCATTGTTCTACTTAGAATTTTATATCTGGTTTATGCTTGACTTAAGTAAAGTTCTTCACTTATAACTTCATATATCA	C	A	ATATACTGGAGCACTTCCTCCAACACCTCCTAAAGAACAACTTATGGACAGGTAAGTAACCCACATAAGATATATTTCTACATGGAAGTTTCAATTTCCT		NA
Ca-II-SNP1095	146.28	Ca-*desi*-LG(Chr)6	6901525	GGATTTCATACCTATTTACTTTCCTCCTTTTTTTGGTTTTAGACATACCAAATAAAAAGAACTTGGATGTTGGAGCAAACCTTTTCATTGGCAATCTAGA	T	C	ACTCAATTTCAAAATTAAAAATCCTAATCATTAAAAATGTTGTTACATATCAAGTACATTTGAATTTATTCAAAAGAAAAAGTTGCATATTTGGTGACTC		NA
Ca-II-SNP1096	147.96	Ca-*desi*-LG(Chr)6	6904308	CCTCCGCCGTCGCCGCCGCAACAACTCCCGCCGTCACCGAAGCTCTAATCGAAAAAAACGAGGAGCAAATCATCACCAATTCCACATCCCCCGGTCCTTC	A	C	AAATAGACTTGAATGCGCGAAGGTTGAATCTCTGGATAGCGATAAGAACGCGAGGATCGGAGCAATTTAGGGTTCCGATTAGGTCATCACATGAAGAGGA		NA
Ca-II-SNP1097	148.80	Ca-*desi*-LG(Chr)6	6909604	AATTTTATTTGTTTGGTTTTTATTATGGCTGTGTCGAGACATCATATAACACTACAAGGAGGAGTTCATTAGATGACATATGATTGTACTAAGTAATTTT	C	G	AACTATTATTTTGGTGTAATGTAAATTGAGTGAAAATACACAACCATCATCATTTAATTTTTGAGAATTTCAAAATTGATATTTGAAAAAAAATACCTTT		NA
Ca-II-SNP1098	149.64	Ca-*desi*-LG(Chr)6	6917240	TTTCTTTGTTGTTTTCAAACTGGTACCAATCATTTCATCGCTCACTTGTTAGGACAAGGAGGACTATGAGCTTTTACTTCGACAGGTACCATGATCCGTT	G	A	TGTGAAAATTTTATATTATCTTGTCGGGAAATTCTACAGCAAACCATTATGATAGGTGGACCACAGTGCACCAGTACTTGAAAATCACTTGAACCTCCAA		NA
Ca-II-SNP1099	150.48	Ca-*desi*-LG(Chr)6	6924077	GGTATTGTTACTTTTTTTAGCTTATACTGCTTGACGTTAATGCTTATTTCTGATACTTCATTATCCAATTATTAGCAATGCTTAAAAATGTCTTCTCTCA	T	G	TAAGTAAAAATGGTAGTTCAAAAAGTTAATGTATCTGGTTAGAAATTAAAATCAGACACATCAACTTTGAGTTAACTTTTTGCTTATATTTTATGAAAGT		NA
Ca-II-SNP1100	151.32	Ca-*desi*-LG(Chr)6	6927099	ACTCTCAAACAACTTCGACACCAAGTCATGTATGTCACCTCTCATCACTCTCAAACTCCCTTATGAAGTTGTTTTTTCTTTTTGTTTTAATGTTTTTGCG	G	T	TGACAGCATTGACTGTCATTTGCATGTCAATTGCAATTGCATCACCCTTTGAGAAATTGGCATCTATTGCATTCGCCACCAACCTTAAAATAATATAATA		NA
Ca-II-SNP1101	152.16	Ca-*desi*-LG(Chr)6	6938851	TTTGGATTTTTTTTATGTACAATAGTATAGTTATTGTAATCTCTAATTTTTTATATATTGTTATTTTTATCACTAGAGCAGTCAATTAGTAATGTATTTT	C	G	GCAGAAATTTTGGATATTAAATGCAAATTCATATCTATAATGCTAAAGAAATTGCACATTTTCGATTTAGAATATAATCATATTCTATTAATATTTATTT		NA

Marker IDs	Genetic positions (cM)	Chromosomes	Physical positions (bp)	Flanking sequences/Forward primers	ICC 12968	ICC4958	Flanking sequences/Reverse primers	Annealing temperature (0C)	Amplified Product size (bp)
Ca-II-SNP1102	153.00	Ca-*desi*-LG(Chr)6	6947535	TCCAAGTCGTGGTTCTCGCCGGTGGCGTCTCCAAAAAACTCCTTCCCCTCGTTTCAAAGGTACTCTCATTTTCAATCCCTTTTCTTCTTTCTTATTTTTC	C	A	AAAGCCTTCGGAAGCTCCTGCGAACCAAAAAGAAGAAATGTTTGAATGTAATTGCAATTCTTATTTTTCCATTCCCATTTTCATCACCAAAGCAAACAGA		NA
Ca-II-SNP1103	153.84	Ca-*desi*-LG(Chr)6	6957538	ATTTCTTTAACTAACTCTTCAAAATCTTTACTTTTCACGTTTGCTTCAAATAAAAGGATTTTAATTGTTCTCTTTATTTTGGGATTGATTAAATATCTGA	A	C	GTAGTTATAATCAAGTGGTTAATAAGCTTCGGCTAAGGTAAAATCGATTAGGAGTATTTCTTTCTTTGTTTCCAGTTGATAACATAATATCACAAAAACG		NA
Ca-II-SNP1104	154.75	Ca-*desi*-LG(Chr)6	6965550	TTCTTTGAACACCGAGGTTAAGGGTTGTGTTGAAAGAGAGGGATTTGGAAGAAGGGGTTGAGGCATTGGTGTAGAGGAAAGAGGAGGTGATGGTGGAAAC	C	A	ATTTGAGTGCTAGTTACTACTAAGAGATAAAATGGTCTACTACTTTTGAACTTAATACATGACAGAAAATTATGACTTTGTATATGCAGTTGACCCCATCTAGTAGGAAAAGGCTTAGATAATAGGTATTGCTGTAAAACTTAATTCACTAATGCCGTTGTTTCTTGTTGATCCATGAACCAAATTGCGAGTGAAAAACATAAGCTATGACATGACAACAATCTAGCATTTT[illegible]		NA
Ca-II-SNP1105	155.53	Ca-*desi*-LG(Chr)6	6968625	ACGGGTCTTAGGTTAGAAATTATGATAGCTGCAGCAGGAGTCCTAGCAGATACAACACTGCCTGTTGCTGGAAACTCCAATAAGGCATTAGCTGACTTCA	T	G	TCATAGTGCCACTGTTATGTGGACCGACAATGATGGAACAAGCAGCCCTGGTTTTGTCGCTGAGAGCACTGGTCACCAGAGGAGCAGTCGACTACTTCGT		NA
Ca-II-SNP1106	156.23	Ca-*desi*-LG(Chr)6	6975916	GTGAATGTGTGGCTTTTGTGTAATGAAATGCTTTGACCCTAAAAAAGCATAAGGTGTAGCGTTTTATAAGTTTGCACGTCATGCATTTAGCATCGGCTCA	T	C	ATAAATGAAACTAAGAAGATCTTTAGTATATTTACCTTGAAAAGTGATGATTTAGCCAAATTGATAGAAATATCTATGATATGTGAATTTTGAATACTCT		NA
Ca-II-SNP1107	156.93	Ca-*desi*-LG(Chr)6	6982306	AACTTAAGATTTCTTAAATGGGAAGAAGCCCTCTTTTATTTTTTAGGGTTCTTTATTCAAGATTTCATCTTATATGTATATTGTTATCTCAGCATTCATT	A	G	TGTTTGGTTAACAAGGACTTGATTATATTATTATTATTATTATTTAGTAGGGCTCTCTGAAAATTTTAGTATTACAGTTAGAATTTAAGAAGCCAGCATA		NA
Ca-II-SNP1108	157.63	Ca-*desi*-LG(Chr)6	6988975	TACCATCCTCTTCCTCTCTGGTATTTTCTCTCTCTTTTTTAAATTGTTTCAAATTTCATTATTTATTTAATTGGTCATTTGTTTATTTATTTTTTGGATT	G	T	TTAACATTAGCTCTAACGTTTATATAATAAGCACTTCACTAAGATGTTTATCCAAACATGCCCCTAATCTAGTGTTCAAAAAACTTTAACCTAAAATCCA		NA
Ca-II-SNP1109	158.33	Ca-*desi*-LG(Chr)6	6995984	TTTTTGTCTATGTCCCAAATTGATAACATTTTACTTGAATTTTCCAGCAATGGTCTTGTTGCAGAGCTTGATAACATGGTTAAATTGGCAGAAAAGTGCA	A	G	GTTTCAAAACATATTATGTCGTATAAAGAAAAACATAGAATCACCGAAGCACATAAAAAGGTAAAAGACTCACTTTACAACCTCCATAGGAACCTGAATG		NA

Marker IDs	Genetic positions (cM)	Chromosomes	Physical positions (bp)	Flanking sequences/Forward primers	ICC 12968	ICC4958	Flanking sequences/Reverse primers	Annealing temperature (0C)	Amplified Product size (bp)
Ca-II-SNP1110	159.03	Ca-*desi*-LG(Chr)6	7015537	TTCTTTGAACACCGAGGTTAAGGGTTGTGTTGAAAGAGAGGGATTTGGAAGAAGGGGTTGAGGCATTGGTGTAGAGGAAAGAGGAGGTGATGGTGGAAAC	T	G	CTACTCCTCCCCCAATAAATATCGCATTCCCATCTCTCAAAATTACAAAAGTCGAACAGTGTTAGTTGCTTCACAGAATAAACCAACATAACATGGCTTC		NA
Ca-II-SNP1111	159.73	Ca-*desi*-LG(Chr)6	7022331	GGTTGTGATGGTTCGTAGGGTTAGGACAACGTATTAAATTTGTTTTTATTTATTTAAAGTGAATGATTTTTATTTAAAGCGCAAGCCTTTTACAGCGCTT	C	T	GATAAAGAGAACAAAAAACTAGCGGTGTTGGTGTTACCATATTCCATCACACTCACACCTATCTCTTTAATTTCTCTCACTTTTTTAGAAACTCTTTCATCATCATGGGTTCCAAAAGTGTAACAACAAATCCATCACTCTTCTTTTCTACCTTGATTTTCCTCGTCCTTATGTTTGGTCTTGCAAACTCAGATGTGAACCAAGACAAAGCTGAATGCACGGACAAACTCC[illegible]		NA
Ca-II-SNP1112	160.43	Ca-*desi*-LG(Chr)6	7028534	CATCCGAATGAATCCTTCCAAAGGGAGTGTGAAATCTCAGGTAATATTTAACTGTTCTTTTTGACTCTGGTGCAACATATTCATTTATCTCTATGGATTG	C	T	AATGCATACATGTCGTATTTGCAGTTAAAGTCTTATCAGCAGCAGTGGTAACAAACAAATCAAACAATAAGGTTGTGACATGCAATTCCAATAGCTTCAC		NA
Ca-II-SNP1113	161.13	Ca-*desi*-LG(Chr)6	7040121	TTCCACGTGGTTCAGTGTTGAATGCAACCATTCGTCTTGCCATGTCTCCTATTTTAGGTAGAACCATTTTTAATGTATAACGGTGCATAAAACGGAACAT	A	G	TGACTACTTCTTCATCCCTAGAGAAATCTTATGCAATTCTAATGCACAAACAATTATGGAATTTAATCAAAATACTCTCTTATTATATCACATTATATCA		NA
Ca-II-SNP1114	161.83	Ca-*desi*-LG(Chr)6	7082685	GGTTGTGATGGTTCGTAGGGTTAGGACAACGTATTAAATTTGTTTTTATTTATTTAAAGTGAATGATTTTTATTTAAAGCGCAAGCCTTTTACAGCGCTT	G	A	CCACCTTATGTTACATGGCTTTTAAAGCGCTTTTTGTGAAAGCGCTGTAAAATTCATGCGCTTTCATATAATTAAATGACCTATTAGAGCGTTTTTTATA		NA
Ca-II-SNP1115	162.53	Ca-*desi*-LG(Chr)6	7097471	TCCTTTCTTTTATTCAGTTTATGTTTTTTTCTTTCAATATCATATTTCCTTCGCAATTAAACAATGTAATGTAACCAATTAATCACTTCTTATAAGGTCT	T	C	GCTGCAACCAAGAAGCCATCAAAACATAAATGGAGAGTAATGGATGATAAATACCCCACAAAACCAATAAGGGTAACAACTGAATGAAAGATGAACAAAACAATTTGTGACTAAGACAATTATGAGAAATCTGAATGAAAGATGAGGGTAATCTATGTACCACATATACCGACACAAATACCAGAGACTATATTGACACTGACATGTCGGCACCGGTAGTAATTTGAGAAA[illegible]		NA
Ca-II-SNP1116	163.23	Ca-*desi*-LG(Chr)6	7219012	AAAAGTATCATCTTTATTATAGTTATAGCTTATATATTGTGTATCCTATCGTGAAGCAAACTAATCGTGAAGCTGTTGGGGTTGATTGCAAGTCCCACAT	C	T	TTCACATGGAAGGTGGTGTACCGGAGGCTGATATATCTGCTTTCAGAGAATGCATGTCTCTTTCATGGAAAAATCCTTATGTTCTTCGTCTTGCTTTTTCTGCTGGTATTGGTGGCCTTCTCTTTGGCTACGACACTGGGGTCATATCTGGG		NA
Ca-II-SNP1117	163.93	Ca-*desi*-LG(Chr)6	7231361	CAAGAGCCATACTCACAATGGCTTCCTGTATTATCAACCCTTTCTCTTTTCAATTTACATTTTTTCTGGTTCATTTAACAAACCAGTTTTAAGATTTTCA	A	G	TAATGACTTAACTACTATGACCAATTACTTGTTCATTTTTGTTTTTAAATTTTGAAATATAAAACTATTTCTACTTATAATTAGTGAGTGATAGAGAAAA		NA

Marker IDs	Genetic positions (cM)	Chromosomes	Physical positions (bp)	Flanking sequences/Forward primers	ICC 12968	ICC4958	Flanking sequences/Reverse primers	Annealing temperature (0C)	Amplified Product size (bp)
Ca-II-SNP1118	164.63	Ca-*desi*-LG(Chr)6	7288581	GGCCTAAGCCTGGTCTACGGCCTATCATAGGTTTTTTTTCCGGCCTGACTTGACCTTTTTATAAGTCTAGCTTGACCTGACCTTTTTAAAAGTCTAGCCT	A	G	ATAGATATGTACATTGATATTGGTATGTGGAGAGCATATGGTATGAGTAGAGCATTTAAATGTTTTAATATGAATAAGGGTTTTAAACAGGCTTCCAGGT		NA
Ca-II-SNP1119	165.33	Ca-*desi*-LG(Chr)6	7337556	ACATTGTTGAGACGGCACTTGAAATTTGCTGATGATCTACAACTTGTTGCTGCTGTTGTGATTTTTGTTGTTGTTGTTGCTGCTGCTGCTGCTGCTGCTG	C	T	CTCAATCTCAGCCTCAAACTCAATCACATTTCCAGCAGCTTCTTCAGCATCAGCACTCATTCACTAATCAAAATCATCATCATCTACAGCAGCAGCAACA		NA
Ca-II-SNP1120	166.03	Ca-*desi*-LG(Chr)6	7366952	TAATGTGTTTGTTATTTGCCATTTAATTTTGATAAGGTATGATTTTTGTATGTTTTCTTTTTAAAGTGATATGTTATGATAACAAGTATTGAATAGATAA	A	T	TCAATACATCATTAACTAAACCATAACACTTTTTACATTGCATTGTATCAACATAGAATTAATGTTCACTCATGTTGCCTATGAACCACATCGTTCTAAC		NA
Ca-II-SNP1121	166.73	Ca-*desi*-LG(Chr)6	7435189	AAAAGTATCATCTTTATTATAGTTATAGCTTATATATTGTGTATCCTATCGTGAAGCAAACTAATCGTGAAGCTGTTGGGGTTGATTGCAAGTCCCACAT	C	T	ATACAAGCTTAAAGTGCTATTGCTGACTGGTGCATTTGAAAACAAATGACCAAAACTCAATATATAGCTTTGGCCTTCTCCTTATTCTCCCACACTAGAG		NA
Ca-II-SSR334	167.43	Ca-*desi*-LG(Chr)6	7446833	TGTCTTATAGCATAGCAAATGTGGA	(AAG)12	(AAG)11	AGTCTCACCATAATGTTTTCACATGCATTCTGAGTCTGAAATGTTGAGATGCATTTTGCTTTTTATAGTCAAAATGAATTGTCTCAGAAGGGGGGATTTT	TGAATTGTCTCAGAAGGGGG	60.06
Ca-II-SNP1122	168.13	Ca-*desi*-LG(Chr)6	7470585	CGATGGAATACTTATGTTTTGTTGTATCATAAGATCGTATGCACCCATGTTGTGTTGTGTCATATGAATGTAAAACAAAGTTTAGCTTATTTGTGATATA	A	T	GACAACTGCTTTTTTCATCAGGTTTTCTTGGACTCATTTTGGAACAATAAACTTCAAAACTAAAAACTAGGTATCGAGGTAAACAGGGAACCTATGCAAA		NA
Ca-II-SNP1123	170.23	Ca-*desi*-LG(Chr)6	7628458	TCCCATTGCAAGCGTAAAGATTCGACATGTTCATCGTGAGGTTGGTTCTATTGTTACTTATTATCTCTTCTGATTGTGTCGGGCCACTGGGTAAAAGTAA	A	C	CTGTGGAGCTTAGGAAGCGTTAGACTTTGACACACAACTTTTGAGTGAAGAGGCTGTTTAGGCGGATACTGCTGCCACAATCAAGTCACAAGGCATCAAC		NA
Ca-II-SNP1124	170.93	Ca-*desi*-LG(Chr)6	7634446	TCCTTTCTTTTATTCAGTTTATGTTTTTTTCTTTCAATATCATATTTCCTTCGCAATTAAACAATGTAATGTAACCAATTAATCACTTCTTATAAGGTCT	C	T	ACGCTTCCAACCTCATATAGTTTCCACAGTTTGTGAAACTAATGACACTTTTTGAATTACATAGCATGAGATAATTATGAAGATCGAGCTTGGTAGCTGT		NA

Marker IDs	Genetic positions (cM)	Chromosomes	Physical positions (bp)	Flanking sequences/Forward primers	ICC 12968	ICC4958	Flanking sequences/Reverse primers	Annealing temperature (0C)	Amplified Product size (bp)
Ca-II-SNP1125	171.63	Ca-*desi*-LG(Chr)6	7653975	GAGCCTATTTCTTTTATTGGTAGATGAATAAATAT<br>GTGCTCCAATTTTTTTTCTGGATTGATGATCCATA<br>AATGGTTCACCATTCCTTTGGTTTCATGTT	T	C	CTGCAGATTCAACTGAAAGACTAGAGGCAACTA<br>AAGAATTTGTTAAATTCTCGATTGATGAAGGAG<br>AGTTCAGTCAATTTGATTCATTGCAAGATAGCT<br>G		NA
Ca-II-SNP1126	172.33	Ca-*desi*-LG(Chr)6	7686474	GATGATTTCTTTAGGGCTTCTTCATGTAGTTCTAC<br>ATATGGTAGACTTGTTGGTGATACGTATGGTAAAT<br>TTGTCAAAAGTTTTTACAATATAATTGGTT	C	A	GTGTAGAGTCTAATTTAAAAATAATTGAAGTAA<br>GATAGAGTTGTTGTGCAAGCTTAATGCACAACT<br>TTAAAGTCCAAAGTTCAACCCATCCGCATAACA<br>A		NA
Ca-II-SNP1127	173.03	Ca-*desi*-LG(Chr)6	7774516	TTAAGCACCACAACACCCCCAGAAAGCTTGGCTA<br>ATCTTTCCTGTAATTTTTCCTTGTCATAATCTGAAG<br>TGCTATTTTGGATTGCCGACCTAATCTGTA	G	A	AGATTTCATTTTACCATTGTTAGTGTATTTTGCT<br>TTCATATCAAGAAAGGTTTGATTGTTCATTGAT<br>GTTTCTGACTTGTTTCTTGTTATGAATTTTTAT		NA
Ca-II-SNP1128	173.73	Ca-*desi*-LG(Chr)6	7788148	TTATTGTTTTTCTATTACGTTGAACTATTTTCAAAC<br>ATTGTGCCTATTTTGTGAAGGAGGAGAAGATATTT<br>TTTTCAAAAATTGTGATTTTCTTTTAATG	G	A	AAGATATTTGGTGCCCTCAGGGAACTCTAAAAA<br>ATAAACCTCGTGATAGTTTTATTTTTACTTGCAA<br>AAATATTAATAATTTATTCATTATAATCAAAAT		NA
Ca-II-SNP1129	174.43	Ca-*desi*-LG(Chr)6	7794972	AGCAGCTGACTGGGTTAACCATCGATCTCCTGAG<br>GCACTTAGTTTCTTTCTTCTTTGGTCTCTGGATAG<br>TATTCTTGCCGACTTGGGTAGCCAGCAAACA	G	T	ATAAAAAGAAAAAATAAATAAAACAAAAGAATTA<br>GACTAGTCTAAGTTACCTGAGATTTTGAAGTTA<br>CTTGTTGCACAGCCTTTTTGGAACCCTTGGACA		NA
Ca-II-SNP1130	176.53	Ca-*desi*-LG(Chr)6	7829769	CTTAAGCCTTTTCACTGTGTGTGTGTATGTATATA<br>TATACAGACCATCTTTTTCTTAATTAAATTATGATT<br>ATATTTTTGTAAACTCACCCTTCTCTCCA	A	C	CATTCAAAAAGACCCTATATCCCATTTCAAATGA<br>TAACTATTAAGCTACATATCATGAAAATAACGTC<br>CTTTAATTAGACTCTACGTCTACTTTTTCATT		NA
Ca-II-SNP1131	177.21	Ca-*desi*-LG(Chr)6	7833350	TGAAACTTCTTTGTTTCTGTATTTTCGGAATATGA<br>GTGTGTGACTTTTTATAATTGTTTCTATTGATAATA<br>CTTATCTAAAAATGTATCCATCATCTTAT	T	C	ATGAATCATAGTTCTAATATTTTAGAGTCTATTT<br>ATTTTCTATATTTCGTGCTTCAGATATTAGAATA<br>AATATCTGACGAGATAAAGTCATCTCTATCAA		NA
Ca-II-SNP1132	177.89	Ca-*desi*-LG(Chr)6	7853727	GACTCTATGAAGTTATTTAAAACTTTTCCTTTTGTT<br>TTGCTGGTGGATTCAACATAGAAAACCAACATGTG<br>TCGTTTACCTTTGCTTGAGTTTGTTCGTG	T	C	CAATAAGTCATGACACATCTCTACAATCCAACT<br>AACATTATCTTTCTTTTCAAACTCTATATAAGCA<br>AATGCTACTAAATATGTCTCTTCAATAGACGTG		NA

Marker IDs	Genetic positions (cM)	Chromosomes	Physical positions (bp)	Flanking sequences/Forward primers	ICC 12968	ICC4958	Flanking sequences/Reverse primers	Annealing temperature (0C)	Amplified Product size (bp)
Ca-II-SNP1133	178.57	Ca-*desi*-LG(Chr)6	7863456	CTAGAGCTTGCAATCAGTTTGACTGGCGTTATTTA GTGAATTATTTCGTGCATGACTAGTTCAGTAAGTC TAATTTTAAGCGAGGAAACTCAAGAGTTGT	G	C	TTCCCACTATGTTATAATTACATCTCCTATAAAA GCTTTTATTAATCAACTAATATATTGACCTTTAT TTATTGTGGTTGAATATTGGTCCAACAAGTTT		NA
Ca-II-SNP1134	179.25	Ca-*desi*-LG(Chr)6	7876356	AATACAAATATGAATGTGATATCTTTCATATTTGTC CGTGAATTTATGTCTTCCTTTTTTTTTTTATTGTTGT TGCGTTCACAAAATTATACCTCTAGAGC	T	G	ACGAAAAAAAACTAAATTAGTATAAAAACTACTT ATTTAAATAAAAAAGAAAAAAGATTTGATAAGTG TTTCTAAATAAAAAAAAATAGTATGGAGAGAC		NA
Ca-II-SNP1135	179.93	Ca-*desi*-LG(Chr)6	7880374	AACCCACCTACCTCTTTTAATCAAATAGAATGATG CTTAACACACAATCACTCTTCCTTTGTTTACTCGG CCTGTACTTCATTTGCTAAAGTAATTGATC	G	A	TTATAAATATAGTAGTTAATCATGTTCACTTTAT GTGTTTGTGCTTGAGTGTTTTGCATGGAAATAA ACACTAGAAATTTTGTTTAGGACTGATAATATG		NA
Ca-II-SNP1136	180.61	Ca-*desi*-LG(Chr)6	7895882	AGCCGAAAGCATTTTACATCCACTACCTCCCTAAC ACACTTATTCTAAATCTATAAATACTTTATCTATTC TTAATTGGACTCCGGACCCAGTTTACTGC	A	G	AAAAAAAGTTCGGTTTAGTTCAGTTTAAAAACA GTTCGATTGAGTTCTACAATTTAGATCAATTTTT AATTTTTTTAACACCCCTATCCTAACTATGCAT		NA
Ca-II-SNP1137	181.29	Ca-*desi*-LG(Chr)6	7917856	TACATTTTTTTTTTTAGTTAGTGTGAACGGATGAAAA ATAAAAAACCAGCCGTTGCTTGAATAATAGCTTCT ACGCGTGCTGTGTTGTGCTGCGCTGAAAA	T	A	CACAAAGACAACTAATAAGGTTACTGTTTTTGTT TTTGTTTTTTTTTTAAGAATAAGGACAGTAGTTT CTAATCAGAAAATAAAAGTTGATTTGGTTTGG		NA
Ca-II-SNP1138	181.97	Ca-*desi*-LG(Chr)6	7936608	ACTAAGAACGATTTCGATATAGTTATTTATCGATG ATTCTTGTTGATGCAATGAAACATTTTTCTCGAAC AACCGGGGAATAATATTGTCTTATGTGTTG	G	T	AATCTTGCTGATTAATATGATTTTTCTCTTCTTG ATCCACTGATTTATAACATGATTCACTCACCAAT TAGATTGTCTATTGGATTGTGATAAAAATTTC		NA
Ca-II-SNP1139	182.65	Ca-*desi*-LG(Chr)6	7943468	TTTTCGCCTTGTGCAGATTTTATGTGGATAATATT CCTATTCGTGAAGTTACGCGAACAGAATCTATGG GAGGAGACTTCCCCGCCAAGCCTATGACTCT	G	C	CAAGGTCGGAGAACTTAGCAATATATGGTGCAT ATTTGTAATTTACTCTGTATTTTCCACCATTGGT AGCCCAATCGGATGCATCCCATATTGTAGCATA		NA
Ca-II-SNP1140	183.33	Ca-*desi*-LG(Chr)6	7958054	AACCTTGTTACCCTTGTTAACAAACCCTAATTCTT GTGCGTGCAATTTTCCGTTCCTTCATTCTCACGCC GCAGTCGCTGCACACACACACCGGAGCCAA	A	G	TTGGTGTGGAAATCGAGTGTCATACGAGAGTA ATACCTCTCGATAACTTGACGAGAGGATTTCTT CACTGTCTTTGTGCGTACTCGTCCCATGGTTAC GG		NA

Marker IDs	Genetic positions (cM)	Chromosomes	Physical positions (bp)	Flanking sequences/Forward primers	ICC 12968	ICC4958	Flanking sequences/Reverse primers	Annealing temperature (0C)	Amplified Product size (bp)
Ca-II-SNP1141	184.01	Ca-*desi*-LG(Chr)6	8006246	GAGATTCAAATTGTAGAATAGCAAAATGTTTCCTATAGTCACTAGTCATTGTCTTTCTAGACTCTGTGTGAACCATTTGGAAGAAGTGGTATTTTGCTCT	A	G	CTGCTTAAGCACAGGTCTCCCTTTAAAGTCAACCGAGCCATCTCCTGTGTATCGCTTACTCTCTTCATCCTTCAGATAAAAAATAACCAATAGTTCATCA		NA
Ca-II-SSR335	184.69	Ca-*desi*-LG(Chr)6	8126601	CAAACAAAGTCATGATGCAAAA	(AT)12	(AT)14	ACAAAAATAAATTGATGAGAAGAAATAAAATAAGGCATATCTATTTGTTTTGTTGGGTTTCACACACAAAAAACACAACACGAAAAAAAAAAATAATATA	TGTTTTGTTGGGTTTCACACA	58.72
Ca-II-SNP1142	186.73	Ca-*desi*-LG(Chr)6	8407081	TGACTGAATTGGCCCTGCGTTTGCGAAATCGTAGTTGTTACAAGTTAATGAGTCTTGAAGTTTAAGTTTGGGAGGAACCTCACTCTGAAAGGTGATATCG	A	G	TATTTGATTTAATTTTAAAAACTTACTAGTACTTGTAAGGTTTTCATACACAAAGTCATTTCAAATTAAATAAGTAAAATACAAATTACAATCCTTATTC		NA
Ca-II-SNP1143	187.41	Ca-*desi*-LG(Chr)6	8455952	GTGCCTCACATTGAGCATTATGGTTGTATGATTGACCTTCTAGGAAGGGTTGGATACTTGGATGAGGCTGAAAATTTGATCTTCTGTGTTGTCGCTCTGT	C	T	GAACTAAACAAAACTAAATCATATCAAACCAAACTAAACAAAGAGAGAAGCAACAAAGCAAATCACTAGAGAGATTAGCAATCAAACAGAAGAAGCCACA		NA
Ca-II-SNP1144	188.09	Ca-*desi*-LG(Chr)6	8709503	TTAGTCCAAAGTAGGTTGAAGACTTCGATGAGAGTTATGCACCTCGGCCGCTACCACTTTCCTTGTGAATTTTGTTGTTTAGCTGAAGTGATTACGACTT	A	G	AAAAAATGCCTGGCCTTACTATTGGAGACACTATTCCTGATCTTCAAGTTGATACAACTCAAGGCAAGATCAAACTCCATCAATTCTGTTCTGATACATGGACCATTCTCTTCTCTCATCCAGGTGATTTCACCCCAGTTTGTACCACGGAACTTGGTAAAATGGCTCAG		NA
Ca-II-SNP1145	188.77	Ca-*desi*-LG(Chr)6	9229603	GCTCAACAGAAATGGGAAGTTGAAACACTCACAGCCTTCTGAACAGCCTCATTAGTCACTCTTGGAGAGGACTTTCTTGAAGCCTGAGCTGCAGCATTAG	T	C	AGTTAAATCCTCTTTTTGACAATATTATGTCATGTGTTACAGATAGTTCAAATTACAATATCTTGCAGGCCATATCAGAGGAGTGATGCAAAATTCCATCTGATGTTCGAATCCAATATCAGTCTCCATGGAAAGTGTCAACACGGAAATAAGGGAAGAAGAAGTATTTACCTGACAAACCAACATCAATTGACTGTATGCAATGACTCCAACACT		NA
Ca-II-SNP1146	189.45	Ca-*desi*-LG(Chr)6	9392890	TGGGGAGTTAATTGGTGTATTCCTTCACTTCCATGTATTTATAATTCAACACAGTGTTGTCAATAGCAATTATAGTGCCGCTATAGTGTTATAGCGTAGC	T	C	TGTAGCCATGGCGGATTACTGGTGTATTATTTGAAATGAAAGTCCAACATAAGAAAAATAACTAAGAATACAATCACAACATAGTTCACGTGCTGTATTATTAGTAGGTCGTATAAGCACAAGTTTTACCGTTACTGGATTTTATGCAACAACT		NA
Ca-II-SSR336	190.13	Ca-*desi*-LG(Chr)6	9802459	TTTGTTTTCATTCAGTGACACG	(AAC)12	(AAC)11	TTTCCTTCATTCTAACCTCTATTTTGCTTTTCCTTTATGTCACTGCCACACCGTTTTCACAACTCATACTGTATACAAATTATAAGGAATTTTATTTTAG	TATGTCACTGCCACACCGTT	58.73

Marker IDs	Genetic positions (cM)	Chromosomes	Physical positions (bp)	Flanking sequences/Forward primers	ICC 12968	ICC4958	Flanking sequences/Reverse primers	Annealing temperature (0C)	Amplified Product size (bp)
Ca-II-SNP1147	192.17	Ca-*desi*-LG(Chr)6	9923291	GCATTTTTTTCCTACCTTCAAAAGGAGTTATTCCT<br>CTTCATAACCATCCAGGAATGACTGTTTTCAGTAA<br>GCTTCTATTGGGACAAATGCACATTAAGTC	T	C	AGTTTTCTGTTTCTGAATTTTCAGGTACATTGG<br>CAGACTCATAAGTAGATTCTCAATTTGTGAGAT<br>GAGCAGTATTCTCTTGATTTTGGCTATAGGAAT<br>AAGCATAGGCTTTGTGGTTGGAGTTGGTTTAG<br>CATTTTCAATTTTAGTCATATTCAGGTGTGGAA<br>GGAAGAGAATAGACGTGGAGAAGAATGGTCCT<br>CGGAGAACCAAGTCCGTTCCTGTTCATGTCAAA<br>[illegible]		NA
Ca-II-SNP1148	192.85	Ca-*desi*-LG(Chr)6	9955114	TCTATATATTCTACATGAATCTATTATAACAACCAA<br>CATTTGGTCAGGGCAGGCTTTGATTTTTGTGTCAA<br>TTACACTTCATATGGAATATTCTTCATTG	A	G	GACCGATCCTGCAATTCCTTATTAAGATCTCTT<br>GTATCATTAAGGCTCTCAACAATGATTGCTTTC<br>TCCATTTCAAGTCGTCTATATGCCTCGTTAGCT<br>GATTTGAGGTCTGATTGAAGACTACTATTGCAC<br>TGTTGTAGGTTCAAATTATCATCAATTGCTACC<br>AACTTTTCTGACTTTTCCTTTGCATTTATCTCTT<br>CTAAGAAAGAAATTGTCT		NA
Ca-II-SNP1149	193.53	Ca-*desi*-LG(Chr)6	10232264	GCCTCTAAACACTATTCGTGGTCCCCATGGATTG<br>CCCTTTCGCTTTCTTTATAGATAGAGTTATAGATG<br>ATACAAAAAATGACTTCAACCTAGTATTTAC	C	A	CCACATTTCTAACCTCATCTTGCAATTTTTGCAT<br>CACAATTGGGTGCTTTAGTAGTTCAGCCATTGC<br>CCATTCTAGGACTGTGTAGGTAGTGTCAGTACC<br>TGCAGCAAACATGTCCAGTATCAAAGCCTTTAT<br>AGCAGTTCTATCAATTGGGAATCCAACAGCATT<br>AGTCTTTTGAACAGAAAGCAAAACATCCACAAA<br>ATCACTATGCTCCTCATCACTATCTTCACCACT<br>[illegible]		NA
Ca-II-SNP1150	194.21	Ca-*desi*-LG(Chr)6	10782792	CGATTTGTCTTCTTCTTCTCAAAGAGAAGAGCTTA<br>TTCGAAAAGTTGCTTCTGCTTTCAACGGCAAGCTC<br>AACATACTTGTTAGTCATTTCTTTTCTATA	T	C	AATTACAATGGCAAGTAGACAACATAGATGTAA<br>CCAATGTGGAATACTACTAATGGTTCCACAAGA<br>AAGCCAAGCCTTTAAGTGTTCAATATGCAATGG<br>CATAACCTATTTTCAATCCACAGGTCCATTGAA<br>TCAAGCCTACA		NA
Ca-II-SNP1151	194.89	Ca-*desi*-LG(Chr)6	11431448	GCCTTCGTATTGGTTACCAATGTGTAGATCGACAT<br>GAATAAAAGAATGTTCATCTTCAGATTGGTGCGTA<br>GATCGGCAAGAATGAAAGGATGATCGTCTT	T	C	GCTGAATCTGTGAATAGCAATGAAATTTATTAT<br>CTGCAAAGCTTAGCAATCTCAGTTTTGAGTCTT<br>AATACAACAAAAACTTCGTCATCGAAGATTCAA<br>GCCTGACAATTGCAACCAAGTACCAGCGATAT<br>GTATATAATGTTAGACATGGCTAATTGGTCACA<br>CAAAGCTGTCACAGGTACATAACTAGCATGGTT<br>TTTTTGTCACATCTTTGAAATTATGGGGGCTTT<br>[illegible]		NA
Ca-II-SNP1152	195.57	Ca-*desi*-LG(Chr)6	11443399	ATCCTGATTTTGAAGAAATGTGTGATGATGTGGAA<br>CTTAATTTTGACGCTATGAATGACGAAGTTGAACC<br>TGTTATGATTGATTTGACTGATGTGTTTAC	A	G	TTTTAATCCTCTTCCCAATTCCCAACCTCCCACT<br>TTGTCTTTTTCTCCTCTTTCTTTTTTTCCCCCTC<br>TAACTTCTTCTCCTGCATTCTTTCTTCTATCTAA<br>ACCCTAGATTTCAAATCTGGGGTTTTCTTTTCT<br>CTTCAGATATGATTATACCAACTTTCACCTTCTG<br>ATTTTCCTACATTTTGCCCTATATATTTCCCATT<br>TTTTCTCTGTCTGCACTGTTTTAGTTCTAAAAAA<br>[illegible]		NA
Ca-II-SNP1153	0.00	Ca-*desi*-LG(Chr)7	7906	CGTTTAACTTCTCGCTCTGTCTAAAACTCTCATGC<br>TTCAATGGCGGCAAGCTCTTCCTCAATCATATGTC<br>TCATATGTTCCAATGTTTCAATGCAACCTA	G	A	TCAAACTAGCCTACTACAACCATGTCGTCGATG<br>AAGTTGAAGTTGTTCTCAGCTAGGTTTGCGAGA<br>CCGACTTTTAATTCGTGAAGAATCTCAAGTTGA<br>T	NA	NA
Ca-II-SNP1154	2.10	Ca-*desi*-LG(Chr)7	26916	CTAAGCATTACATTTGACCCAGTGGTGACTGCAGA<br>AAACCCCTAGATACAGGAAGTGGATTTGTGGTTG<br>CAACTTTAGAGTTTGATGACTCATTCGCTTC	A	C	CAGCACGTGGCAGAGGCATGCACACTAGGGCC<br>TCAGCTTGGCTAGATTTACACATATGCACATAT<br>TCCAGGAAGACTTCTTTGTTGTCTTATGCAAAT<br>AT	NA	NA

Marker IDs	Genetic positions (cM)	Chromosomes	Physical positions (bp)	Flanking sequences/Forward primers	ICC 12968	ICC4958	Flanking sequences/Reverse primers	Annealing temperature (0C)	Amplified Product size (bp)
Ca-II-SNP1155	3.04	Ca-*desi*-LG(Chr)7	32994	TAGACCTAATATTCTATTTAGTGTGAGTGTGTGTCAGATTTTAGTTTGATCCTAAAGAAACCATACTTGGTTTATATATCTGAAAGGCACTACTAACCTT	G	T	TAGTTTCCTCTGGTGCTTTTCCTTTCTACCTTGTCTCTCGTATAGTCAGCATTGTAGAAACCAACTAACATGTACTCAGAGGATGGCTAAAAACACAAAC	NA	NA
Ca-II-SNP1156	3.98	Ca-*desi*-LG(Chr)7	41554	CTGCCTAAGATGATAGGCGCCCTGTTGGACTTATTCTCTCCCTCCACGTCCAGAATGTAAAAATCTGCAGGAAATGTCAATTCATTAACTCGAAGAAGTA	C	T	TATTTTTAACTCTCTTGCTCTTGGACCTTTACAGAGTATAGGTGTTACCATTCAATTGGCGAACAGGAGCAATGCTCATCCTGTTGGACTTATGGAGGAT	NA	NA
Ca-II-SNP1157	5.86	Ca-*desi*-LG(Chr)7	86750	AAGGACATAGATGTTATGTTGTTGGTGGTAACTCGAAGATGATGGGTATGGATGGATTAACGTTATACGAATAGGCGATTTCATGTAAGGATCTAAGTGG	C	A	AATTTCAGGGTATTCACACCTCTTTGACAGTTATCAAACTTCATCCAGTCTCATCCTGAAAACTATCAGGCGAACATCTAATATTCTTCTTAAAAAGCTC	NA	NA
Ca-II-SNP1158	6.80	Ca-*desi*-LG(Chr)7	102757	TTACAAGCAAAAGATCTAATTTTAATTAAATCACTTTACTTGGAGTTAGGTAGAAAAACTTTTGTCTCGTCTTGGTACAGTTCTAAGTTTGAGGTGGTGG	T	C	GAAGATGTTATTCATCAAAGTACACAAACTGATTTTTTTTTCTTTTTCTTTTTTATTTATTTATTTATTTTTCTTGATAATTTTTATTTTATTTTTGAGA	NA	NA
Ca-II-SNP1159	8.68	Ca-*desi*-LG(Chr)7	155116	ACTCCATCGAAAGTTTCGACTCTTCCCCGAAATACGATTTGAGTCTTTGTCCATTGACCTTGAAGTTCCGATCGGAGTGTATATCTTTCAATTCGACGGC	A	C	AACTAGTTCTCCTATTCAACTCAAGATTGAAGTTGTTTCCCGGAAAACTAAAGTCAAGATGGTCGGGCCCTTTGAAGGTAACCAAGGTATTTCCACACGG	NA	NA
Ca-II-SNP1160	9.62	Ca-*desi*-LG(Chr)7	196707	GGACCTGCACCTGAATCAACAGCAGGACCTACACCTACACCTGCACCTGAACCTGCACCTGAACTAACAGCAAGGCCTGCACCTGAACCTGCACCTAATG	G	A	TACCTCATGCAGTGATGATGGGTTGGATTCTTGGACATTTCCAGATTCTAGAATGGCCTTTTCTCTCCTTGAATATGCCAGATTTTTCCCAAATTTTCCA	NA	NA
Ca-II-SNP1161	10.56	Ca-*desi*-LG(Chr)7	253246	TTTTCTTCTCTTTATCTTCATCTTTTTCCCTTGATTGATCTCTCTTTAGGAACCTGCAATTTCTTTTTATGTGACCCGTTTTGCCACAATGATAGCATAT	C	A	CAAAGTCACGGGGGAGAAGTCAATCTAGAAACTTCCATAGACGCGACAACTCTGAAAGTTGTAGCAAGTCAAGAAGCAAGTCGAGGACTAGAAAAGAAGT	NA	NA
Ca-II-SNP1162	12.44	Ca-*desi*-LG(Chr)7	268136	TCTAAGTACACTTCCTTCTTTTTCACCATGTCAAACAAGATTGACTTCAAAATATTGAAACTTTTTTTTCATTTACATGGCAATTTTACCTGTCAATGGA	T	G	CTGTTACATACGTAACATTCACCAGGTTAAATAGAAATGTTATAAAGTCTTCGTTCTCTATCAAGAGTGAAGAATGTTCAAGAGCTACATCAATGAAACA	NA	NA

Marker IDs	Genetic positions (cM)	Chromosomes	Physical positions (bp)	Flanking sequences/Forward primers	ICC 12968	ICC4958	Flanking sequences/Reverse primers	Annealing temperature (0C)	Amplified Product size (bp)
Ca-II-SNP1163	13.38	Ca-*desi*-LG(Chr)7	272276	TTAAATACGTTACTCATATCCCACCAATAAATGTGTCGCTGCCTTCTGCTTTTTCCACTCAAAATCTCAGTACACTTCCTTCTTTTTTCACCATGTCAAA	T	C	AGTGAATAATGTTCAAGAGCTACATCAATGAAACACCCATTGACAGGTAAAATTGTCATGTAAATGAAAAAAAAAGTTTCAATATTTCAAAGTCAATCTC	NA	NA
Ca-II-SNP1164	14.32	Ca-*desi*-LG(Chr)7	276790	GGATTTAAGCCAGGTTAAAAAAAGTAAAAGATGTCGTGTATTTAGCGTTTTATCTTCCTGCTCTCATAACATCTGCGCTCTAAACCTCTTCTTTCCATTC	C	G	AATCAAACATGTCTTAATGAATTGATGAAGTGATGATGATAAAACGGCAACGGTTGTAAAGTCGAATCCAAGTTGTAGGTGCTTCACGATACAGAGGTTA	NA	NA
Ca-II-SNP1165	15.26	Ca-*desi*-LG(Chr)7	333608	CTCTTTTTTCACTTTTTTTTTAAAGTCATTATTCAGATTTATATTGTTTGATTAGAATTACCAAACTTTCTGTTCAAATCAATTAACCAAGTTTTTCAAG	G	A	ATTGTGAACAATTTGGATAATGAATTTGAACTAAAAACTTGGTCATTCTAATTAAACAAAATATAAATTTGGTTAATGACTTAAAACAATATGGTTAATT	NA	NA
Ca-II-SNP1166	16.20	Ca-*desi*-LG(Chr)7	366332	ATCGAAACCCGGGTACACCCATTTTAGTAGAAACTGCCCCTAAGCCGGTAGGTCTCGTTCAAAAGCCACTTTAGTTGATGTGTCCCAAACCATGTGCTCG	A	G	TTAGTGTGTTTGGGTGTGTGTTACACTTTTCTTCTCATGTCCCTATGTAAACTAACCAAAAATTTCAGCAACTTGCGCATCAACGATGACAACATCATTA	NA	NA
Ca-II-SNP1167	17.14	Ca-*desi*-LG(Chr)7	370425	TTGCACCTTGGTAATTAGTGCTGATCTGTGAGTGATTGCACTCTAGAATGTGCTGATATGTGAGCGATTGCACTCTAGAAAATCGTGTTGGTCTGTGAGC	G	A	ACTGACCAATATTAATTACCAAGGTGCAATCTCTCATATATCAACACGATTTACTAGCGTGCAGTCGCTCACAGACCAGCACGACATACACGAGTGCAGT	NA	NA
Ca-II-SNP1168	19.96	Ca-*desi*-LG(Chr)7	425722	AATGGTGTCCGGAATGCCAATTACCATGCAAGCATGCATCCTGAACGCGAAACCTTTACATCGTTGGAACCGCTTTCGCCGCCGGAATTGGTGGTCTTCT	C	A	CACATAAAACTCTGATCAAGTCCAATAGCATCAACATCATAAATGAAGCTAGTAGATATTGAATTGAATCTACATAGACCAGACCTGTATCGTAGCCGAA	NA	NA
Ca-II-SNP1169	20.90	Ca-*desi*-LG(Chr)7	465928	TATAATTATCGAAATGTTCAACAACGTTGCTCTTTTTGTATAATTTAATTATTTATGGTTCATTGTACCCACAAATCATAATTTTACTGTGTCTTTTTCC	A	G	ATATCACTTAACCACATTGACATAAACATCTTCTCAACTTCACTTATCAGATGTTACTTAAACTTCACGTTGAATTTAAATGTTATAAGTTGATTTATTT	NA	NA
Ca-II-SNP1170	21.84	Ca-*desi*-LG(Chr)7	468231	CGAGATTGACATTGATCAGGTATGTTGGACACCATATGAGGAGCATCGGGTGAAGTGACCGTTTGAATAATATTTTTTATTTTGAGGGTGGATACGTTGG	G	A	GTCGTCTGACCCATTATCTCTAACGGTGAGCTAGAAATGGTTTGTACATGATCGTATTGACGCAATATTCTGTATAACAAATGAACATACATTTTTGAAC	NA	NA

Marker IDs	Genetic positions (cM)	Chromosomes	Physical positions (bp)	Flanking sequences/Forward primers	ICC 12968	ICC4958	Flanking sequences/Reverse primers	Annealing temperature (0C)	Amplified Product size (bp)
Ca-II-SNP1171	22.78	Ca-*desi*-LG(Chr)7	470261	TTCTCAATATTTATTTTCTCCCCTTGTTAGCTTAGACTATTCCATTAAGAGCATCATCTTTAGGAATCATCATTATCTCATCAAGTATGAGGTACTTCAA	T	A	GTGATAATAAAAGATGGATGCATGGTATGATATATGTTTACGTGATGATAATGATGATTTTTGATTGATGATAGTGATGTTATAAAATTGTGATGAGAAT	NA	NA
Ca-II-SNP1172	23.72	Ca-*desi*-LG(Chr)7	500019	TTATTTTAGTGATTAAGTAGGAATTTTTCGTAATTTTACAGTGCGTTGTGTTTTGGTGCAGTGCTGAATTTTGGTGAGAAATCAACATAACATATGTAGC	T	G	TCTGGTGTCTTCATGAACATTGTAGCTATGGATCTTAGCTTTCATTTGCACTTGGTTTAACTCCAATTGGACATCTACAACTCCAGATATGGCTGAAATA	NA	NA
Ca-II-SNP1173	24.66	Ca-*desi*-LG(Chr)7	502609	TGCATCTGGGTTCCTTAATTGTTCATACATGCATGTTGATTTTCTGAGATTGAATAGGTGTTAATGTTGCATATATGTGAGTTGTTCATGTGTTTTATGA	T	C	GTGGTTGTGTCATTTTAACAAACAGTTGGAGTGCAGTCATGTTTTGTGGGAATTAAGGTGCAAAAGTGGTTGTGTCATTTTAACAAACAGTTGGAGTGCA	NA	NA
Ca-II-SNP1174	25.60	Ca-*desi*-LG(Chr)7	618120	TATTTATGTTTATGTAGTTAGCTCCTTGAGTCTCGAAAAGGAATCGAGACCGTTGGCACCGAGTTAGCGGTGGGGAGAACTCTGATATATTAATGTTGTT	A	G	TAATTATGAACACATTAATTTCACCAAACATGAATCACCAAAATTTCCAAACATTAATCACCCATATTCAATCTCCAAAATTTTCAACCATAAATTACCA	NA	NA
Ca-II-SNP1175	26.54	Ca-*desi*-LG(Chr)7	642019	GATGACTAGTAAATAATTCTATGGTTTGCTCTAGAACTAGGAGTCGTCTCAGATTCATGTGCAGGATTGAATTTCATTTAGTTGATTATTTGATTTCGTC	C	T	CGGAGAATCTTGGGTTATGTTGTGTTGTTGGGTTCCATGTCACTCTTTGTGGAGAACTTACAATATTAGTGCCTCTAGTCGTCACTCGCGTCGTCTCTCTTTTCCCCTAATCCTGAAAATAAGCTGCATGGACGCCGGCGGCTCAGATTCCAATGGCCGCGAATACAACACCGCCGACGAGATGTGGAGTGAACAAGCCGGCGACCTCAATAAGAAGACTCTCTGGTA[illegible]	NA	NA
Ca-II-SNP1176	28.42	Ca-*desi*-LG(Chr)7	684110	TTCTTCTTTTCGGTGTGTATCACTCTCATGTTTGTTGTTCTATTATCTATCCTTATTTATATTTCACTGTTAAAATAAAAGGATACATTATATGAAGTTT	A	G	AAACAAACGTAAACATATCCAACAAAGCAAGCCTCATTTCAAAAAAAAAAACTCAGTTTCAATAAATCAACCTAAACAAAATTTATTTTTAATCCTAAAA	NA	NA
Ca-II-SNP1177	29.36	Ca-*desi*-LG(Chr)7	765277	TGCAATTCTCTTGTATACAATTTGAGTTTTACAATATGAATTTAAAATTCATATTGCAATTTATTTTTTAGGGACATCAATTTCAAGTTTGCATTGGGCT	T	C	CAAAACGTGAGCTTATAGTTGATGACCATAAATTAAATTACACTATGAAATTCTTATTCATATTGTAAAACTCGAATGATGATATACATAAGAATTGATG	NA	NA
Ca-II-SNP1178	30.30	Ca-*desi*-LG(Chr)7	770412	CACCTTAGAAACTACCATGCCTATTCCAGAAACTATACTGCTATCCTTTAAATCTTCATCAGACTCCAGGGGAAGACCCTAACTTTTTTACCATCTACCT	A	C	TTGGTTAAGGCATGGATTTGGTTAGATCATGTCTTGTGGCCTCTGTTCATAGAGTCTGAAATTGGTTGCAAGAGAAATGTTCTGAGATATATATTATATG	NA	NA

Marker IDs	Genetic positions (cM)	Chromosomes	Physical positions (bp)	Flanking sequences/Forward primers	ICC 12968	ICC4958	Flanking sequences/Reverse primers	Annealing temperature (0C)	Amplified Product size (bp)
Ca-II-SNP1179	31.24	Ca-*desi*-LG(Chr)7	845234	ACTGATGTCAGTATGTGATTGAAATGAAAATTATTTGATTTGCTGGGAAATAGCATGCTGCAAGGGATCTGAATATATGGTAGTTATGTTCTATGTAGTT	T	C	TATTCATATACATATTCATATATTGACAACAACACATATTCATATAACTACATATTCACAACTTTTCAAATGTGCCCTATTCATATACATATAACTAACA	NA	NA
Ca-II-SNP1180	32.18	Ca-*desi*-LG(Chr)7	870350	TCTTGTGTATAAATAGATTTGTTGTCAATCTGCTCCATTATTCCAATGATTTTGAAAGTGTGGTTTTGGTTGACAACTCAGTATTTCTATGGCCAATTTT	T	A	CTAGTGCTAGATTTTGGAATGTCCTCTTCATATTTCCAGTAAATCTTGGAGTTAATTGATCAATTAAAAGGTTTGTATTAAGGCAATCCAAAACAGTTAA	NA	NA
Ca-II-SNP1181	33.12	Ca-*desi*-LG(Chr)7	925631	CAAAGACTATGTTCGGTCTACTAGCTGTTAAATATAGGAAGGATCCAATCATCCCTCTATACTCTTTTTCAGATATTGGAGTTCCTTCATCATCTTTGTG	G	A	TCTTCATATCTCAAGAAAAATACACCAAGGATCTACTCAACAAATACAAAATGAGTTCAGCCAAAAGTATGGGAACTCCTATGCATCCATCCTCCATACT	NA	NA
Ca-II-SNP1182	34.06	Ca-*desi*-LG(Chr)7	929639	ATTGACACACACTGTTTTTATACTGGTTCACCCAAAGATGGCTACTTCCAGTCCTCACACCCTTGTGAGATTTCACTAATGTTCAAACAGATCAACCTCT	A	G	TCACACTGTAATTTCCACGTGAGGTACACTTGGAAATATTTGAAATCTGATTGTAAGCTTGGTGAAGCTTGAGAATACACAAGTTGTAATCATCCTCGGT	NA	NA
Ca-II-SNP1183	35.00	Ca-*desi*-LG(Chr)7	944691	GATGACTAGTAAATAATTCTATGGTTTGCTCTAGAACTAGGAGTCGTCTCAGATTCATGTGCAGGATTGAATTTCATTTAGTTGATTATTTGATTTCGTC	A	T	ATGCATCACAACTGGAATGATACGGTAAAATTAAATTTTAAAGTAAATGGAATAATATATAAACCAATTATCATTGAATCCCAGATCCAGAATAAGAAAA	NA	NA
Ca-II-SNP1184	37.82	Ca-*desi*-LG(Chr)7	975862	TAACTTTTTGTTTGTTTTCATCAAAACATTAAATTGAGATTTTGTCTCAACAATCTCCCCCTTTTTGATGATGACAAACATGTTAATTTAGATTTTAATT	T	G	GGGAGCTTTGTTAACTTGGACGAAAATGGAGAAGAAGTATAACATAGATGAATACAAAGGCCAAACAAGATTACCCAATTTTGCAATTCCAAAAAAATATGAACTTCACTTACTACCTGATTTTTCAGCATGCACTTTTTCTGGCACTGTGCAAATTACTCTTTCTATCAAAGAAAACACCAAGTTCATTGTCTTAAACTCCTTGGAA	NA	NA
Ca-II-SNP1185	38.76	Ca-*desi*-LG(Chr)7	1003445	TACAAATCTTACATCCTCCAATATTTGTCAACTCGCCAAAATTCTGATGTGGCACATTCCAAATTCATCTTCTATTCCAATATGTTTTATTTCTTCAATA	C	T	TTTTTAAGTGTATTGATGAGTCATTGAGTGATAAGTTAAAATTAAAATTATATAATGTATGAAATAATTGTACTTGGGCAAGTGAAATGGTTTGCATTAT	NA	NA
Ca-II-SNP1186	39.70	Ca-*desi*-LG(Chr)7	1045780	TCTTAGCATTTAGATATGATGCCCATTATAGTTGGTGATATACTGATTTATCAATTTTGTGTACGGCTATTGTGTTTGATGTCTAGCGATGTTCATTTGA	G	A	CAAAAAGAATGAATTAACATTTAGATCAAGAAATTTCCCTAGTTCTGAAATACATATAGAACTGAATGCATCATTATTTTAATACAGCCCTAAAGGAAGA	NA	NA

Marker IDs	Genetic positions (cM)	Chromosomes	Physical positions (bp)	Flanking sequences/Forward primers	ICC 12968	ICC4958	Flanking sequences/Reverse primers	Annealing temperature (0C)	Amplified Product size (bp)	
Ca-II-SNP1187	40.64	Ca-*desi*-LG(Chr)7	1066575	TCGTGGATTGCCATATCACTCTTGAATCACAAAAGTAGTCCTCCCATTTTATTTCTCCCAAGTTTTGGTCCCTCAAACAGAATTTTGGTCTAAAACTTGA	C	T	TTGAATTAAGTTACACCATCATCAAGATCTCGTGGTGTAACAATTGTACCTGAAATCTATGATCATAAATGGTGTGGTGTGACTTAAAAAATGACATTTC	NA	NA	NA
Ca-II-SNP1188	41.58	Ca-*desi*-LG(Chr)7	1090742	CAAGGGCTTCGTGGGTCCTTCCAAGCAACACCACTAACTCGAGCTTCGTGGGTCCTTCCAAGCAACACCACTAACTCTAAATCCCCGCAAGGATCTGTGG	A	G	TGATTTTGGACGCTCTGGTTGAGTGATCTATATTGTTACTAACAGAAGTGTCTACATTCATGCATTCATAGTTGTGTGGTGCGTCCATAATTGGTTGGAG	NA	NA	NA
Ca-II-SNP1189	42.52	Ca-*desi*-LG(Chr)7	1095355	TAACTTTTTGTTTGTTTTCATCAAAACATTAAATTGAGATTTTGTCTCAACAATCTCCCCCTTTTTGATGATGACAAACATGTTAATTTAGATTTTAATT	C	A	ACAAAGGGGGAGAATTATTTTAGATTTTATGTTGGTTCAAATATATGAAGCATATTCTGAGGGGGAGTTTTTAAGCTCTCTTTGATTAAAATTAGAATCA	NA	NA	NA
Ca-II-SNP1190	43.46	Ca-*desi*-LG(Chr)7	1451148	TTCTTAAAATCTACACTTTTCAAACCCCACCAACAATCTCCTCAACCATTTTTTCATACATTCTTCAACCGTATCACTGCTCCCTCTCTAGATTTGTTCT	T	C	TAAAGATAAATGAAAAAATAAAATAAAATTAAAAACTATATAAAATAATTTAAAAAAATCAGAATAGATTTCGGTATCACACAGATCTAGCTAATCCCTC	NA	NA	NA
Ca-II-SNP1191	44.40	Ca-*desi*-LG(Chr)7	1468684	TAATTATCTCCATTTGTTGATTCCCTTTAGATGATAGTGTTGAATATAATGTCTTTAATTTATGATATCATGTGTCTAGTTTTCATTTGAGTATCTCTAA	T	C	AAGTTGGTCACAGCACTTGTTCCCCTGAATTTAATTGCTGCAATATCATATGCTTCTGCAGCCTCCTCTTGTGTGCCTATAGGATTGTTATGGTTCACCA	NA	NA	NA
Ca-II-SNP1192	45.34	Ca-*desi*-LG(Chr)7	1483940	ATTATGATGAAACATTTGCTCCGGTAGCCAAAATGACAATTGTTCGCATTGTACTCTCTATAGTTGCTTCTAATGGGTGGACTCTTTATCAAATGGATGT	G	C	AGCGTTTGAGCTTGCACACACCATGAGATGAAGAAAATAGACCTTGAGGAGGAGTCATATAAATATCTTCAGTGAGATCACCGTAAAGAAATGCATTCTT	NA	NA	NA
Ca-II-SNP1193	46.28	Ca-*desi*-LG(Chr)7	1503761	AATGATCATTTTGATCATTACCCTTATTCTTTGGTTCAAAATGTTCTAAACACTTGTTGCATTTGATTCTTGAGACAATATTTGACTTGTTTGGTTCTCA	T	C	AGAGAACATTCTCCTTTTTCAAAGGTGAATATTGGTTTAAGAGTTCATGGGTATTGTGCCTACATTGTGTTAGCACTTTTCCAAATAGGTTTATAAACTC	NA	NA	NA
Ca-II-SNP1194	47.22	Ca-*desi*-LG(Chr)7	1617972	TTTTCTGGTTGACCAGATTTTTTAATTTGTTGACTAGATTCTTGGTGGAACTGGAACTACTAGAAATCGAATTGGAAATGTTAGTCGGAATTGAAAATGA	T	C	AGTGAAATTGAAGCTAGTGATTGGGAATTGGTTCAAAATGATAAATCAATTGTTTCCGTTATCAACGAATTGAATTCTCATTCATCAATTATCAGATCCG	NA	NA	NA

Marker IDs	Genetic positions (cM)	Chromosomes	Physical positions (bp)	Flanking sequences/Forward primers	ICC 12968	ICC4958	Flanking sequences/Reverse primers	Annealing temperature (0C)	Amplified Product size (bp)	
Ca-II-SNP1195	48.16	Ca-*desi*-LG(Chr)7	1649012	TGTAACTGATGATCATGTGCCTCACCATGATGAGC<br>AGGTTCCAGTTGAGCCACCAGTCGAGTTTGAGTT<br>GAGAAGATCTACTAGAGAACGTCAACCTTCT	A	C	TTTAACCACTTTTCTTTATCAACATTTGTAATAG<br>GTTCTTGATAATACTCTGGCTCTCCACTATCAG<br>TGATCATCACATACTCATGTGGAGGATATCTTT	NA	NA	NA
Ca-II-SNP1196	49.10	Ca-*desi*-LG(Chr)7	1698235	GAACTGTGCTGCAAATATTTCTGCATGAAAAGTCG<br>TAATAAAATGTCTAAAGTTTTCTTAAACTTTGCTGC<br>GGATTGTATTCAATTTTCCTGCGGATTTA	G	T	GTTACCTGCGGATTTGCCGGCAGAACCTTATC<br>CCCACGTAAAACCTTCGTGGATAATTGTTACCT<br>GAGGATTTCACTATCCGCAGGTAAATTACCTGC<br>GG	NA	NA	NA
Ca-II-SNP1197	51.92	Ca-*desi*-LG(Chr)7	1721640	GCTGTTGTAGGGTCATATAGGGTCACATAGCGTT<br>TGCACATAATGCTTACAACCCTTTTACAACGCTTT<br>TTGTAAAAGCGCTGTAAATTGAAGCGCTCTC	T	C	TTCACAAAAAGCGCTTTAAAAGCCATGTAACAT<br>AAAGTGGGAGCGCTACATTTTACAACACTTGTG<br>TTTAAAGCGCTCAAAAAGCGCTGTAAAAGCTAT<br>G	NA	NA	NA
Ca-II-SNP1198	52.86	Ca-*desi*-LG(Chr)7	1776493	ATCAATCTTTATAACTTTAGGGACATATTTGTATAC<br>TCAGTCATTTTTTTTTATCTCAGACTTCCAAATTGG<br>TTTAATTTAATGGTATATATCAACAGCT	C	T	AAGCAAAAATGAGAGTGCTAGGATTAGAATATT<br>TGACCTTGTCTTTCTTAAAAGAACACAATTAGC<br>ACAAGACTATAGATAATTCATTGTCATCTTTAGT	NA	NA	NA
Ca-II-SNP1199	53.80	Ca-*desi*-LG(Chr)7	1791302	TTAAACATAATTTTCCAGATTTTTAACAACTCGAAT<br>TTCTCCCTGAAATTCCCGATTCTCCGTTTACAGAA<br>TTCTCGTTATTTTGCATCGTTCTTCTTCA	T	C	TTAAGGAAAATTACCATAAATCATAAAACCAATC<br>AGTGGATAATATAGCCACTTTTTACACGATCGT<br>TTTGGCGTTAGTTTCACTCAAATCGGACTTACA	NA	NA	NA
Ca-II-SNP1200	54.74	Ca-*desi*-LG(Chr)7	1842420	ACTCGCGTTTTGGAGCATAAACCCTAAATTTGCAG<br>TTCCAACTCTAGCTCGTGATTCGATCAAGTTCATC<br>CACTTCATTCCCTCTTCTTGATCATTGTTG	T	C	AAAAATAAAATTCAATTTAAAACCCAAATTCAAA<br>AAAACCGTAATCGAAAGAAAGAAAAAGAAAAAC<br>TAACCAAAGAAATTATCAGAGAGAATGAAGAAC	NA	NA	NA
Ca-II-SNP1201	55.68	Ca-*desi*-LG(Chr)7	1855882	TCTAAGTACACTTCCTTCTTTTTCACCATGTCAAAC<br>AAGATTGACTTCAAAATATTGAAACTTTTTTTTCAT<br>TTACATGGCAATTTTACCTGTCAATGGA	T	G	ATTCATCACTTGATCTACAATAGTCTCAGACAA<br>CAACTTTTTAAGCATCTTAGAACACTTTCCAAG<br>CTTCCTTCTTTTATTCTCATCAATTCCATAACCA<br>ATGCAATCAACACATTTCCTTCCTTGAGGCATA<br>GATCCCATTGCTCTTATCACACAGTTTCTACAA<br>TACTTTGCACTACAAACA	NA	NA	NA
Ca-II-SNP1202	56.62	Ca-*desi*-LG(Chr)7	1868303	CATTGATTGTAAATGAAATTTTAAAGGCATATATTT<br>CAAACCTTTATTGGAATCAATGTGATGTTATTTGA<br>CTTTTGCTTCGACATTGTAGCACCTCTTT	T	G	AATATTCATTTATTTCTTATATATATATATATAAT<br>GTGTTAGTTAATGCTATAATAATCACATTTATTT<br>ATTCGATAGTGCTTTACAAGTTTTTCGAGCT	NA	NA	NA

Marker IDs	Genetic positions (cM)	Chromosomes	Physical positions (bp)	Flanking sequences/Forward primers	ICC 12968	ICC4958	Flanking sequences/Reverse primers	Annealing temperature (0C)	Amplified Product size (bp)	
Ca-II-SNP1203	57.56	Ca-*desi*-LG(Chr)7	1916451	GATTAAATTTAGTTTGATTAATACCATGATGATCTGATGGATGATACTCTATATTATTCATTTTTCCTCGGTATTATATTGGAGTCAGGCAAAACATCTG	G	A	TTATTCATCAGATCATAATGGTATTAATCAAAATGCATTTATGGCTAGAACAAATAATTTTTCCATGATTGGATATTTGAATCAAACAGATTTGGCATGT	NA	NA	NA
Ca-II-SNP1204	58.44	Ca-*desi*-LG(Chr)7	1922382	CAATCTTCCTTTTTTTTTACAACAATTTCCCCCATTTTTTTGACAACATTTTGTTATCATAAAAGTGTGCAACAAACTTCATTCTTGTACTCATTAATTG	A	G	TATTTATGATATTTTTAAAATTCATTTTGAGATGATAAAATAAGTCAAATTGGTCAAATTTACTATGTTTTTAGTGGAAAAAATATCAAAATTTAATATT	NA	NA	NA
Ca-II-SNP1205	59.32	Ca-*desi*-LG(Chr)7	1938849	CAAAGCCTCCTCCTTTGTTTGATGTTTTTCTCTCAATTTATAGACTAAAATGATGAATCTTTATATTCATTAGATAAAATTGAGTTCTAGATTATTACTT	G	A	CCCTAAAACAACTACAATTTTTACCATGTATACAAGAAATAAAAAAGTGCATTCTCTCTTTTCTGGGTTTACCTTAGGAAATTCAATTTTTCTTCAAAAA	NA	NA	NA
Ca-II-SNP1206	60.20	Ca-*desi*-LG(Chr)7	1949027	TTCTATTAAGTTTGAGATTACTTTAGTGGTTGATTTTTTAGTTTCCTTTTATTTTTTGTTTTTATTTTATTATTATTATTATTATTATTATTATTATTAT	A	T	TTTATTCGAAGATTAAAAATAAGCAAAAAATACTCAAAATATTGAAACAAAATAAAACTCAAAATCAATTATCAATAATGATAATAACAATAATAATAAT	NA	NA	NA
Ca-II-SNP1207	61.08	Ca-*desi*-LG(Chr)7	1958630	GAGTCTTCGTCGTCTGGCAAACTCTCGAAGGAATCATCATTACTCAAACTTCCTGGGAAGTCACTTTCAAGGCAGATGTCCTCATCAGGAGAGGGTTCAT	T	C	TGAAAAACAACACAACAATTCTTTTAACCAAACGGGGTTGAGGGATGAAGGAAGTGTGAGAAGGACACCTAAAAAGCAAAAGAGTAAAAGAAAATGTTTC	NA	NA	NA
Ca-II-SNP1208	61.96	Ca-*desi*-LG(Chr)7	1962522	ATGAAATAATAATGTTTTGGTATTAAAGTGGGGGAAAATTGGAGTTTGTTACATGGTTGTGGTTTGTCATGTGAGTATGATCTAAAACTTCAATGTAGGC	T	A	GAGGAGATGGCCTACCCTGTTGAGAATTGCTGCATGCATCACTTTCTTCAACCTAAATCTATAATGAAATCTTCAATTGAATAAACTCGTCTTATTGCTT	NA	NA	NA
Ca-II-SNP1209	62.84	Ca-*desi*-LG(Chr)7	2036771	AAAATTCACCCCACACTTTCTTAATAAGAAGGTTGGTGGTCAAAGAATATTTTGCAACACAGCTGTTTTCTTATTCGGCTCTAGACTGGCCAGCACGTGA	C	T	CCAATGCACAGCAGCCAAAACTTTTTGTTGGAATGATTCTTATCCTCATCTTTGCTGAAGCTCTTGCCCTTTATGGTCTCATTGTTGGTATTATCTTGTC	NA	NA	NA
Ca-II-SNP1210	63.72	Ca-*desi*-LG(Chr)7	2042539	TCAATTTCCATACCAAGGATTCTCTTAGCTGCTCCTAAGTCCTTCATTTCAAATTCAGAATTAAGTGATTTCTTCAAACTGTTGATAGCATTCATATCAG	A	C	CAATAGAAGCTCTTATGACTGTTGTGTCTATTTCAAAAAAGAAGGTTCAATCAACTTCATATATCTATTACTCTATGTAGATGATATGTTAATAGCTAGT	NA	NA	NA

Marker IDs	Genetic positions (cM)	Chromosomes	Physical positions (bp)	Flanking sequences/Forward primers	ICC 12968	ICC4958	Flanking sequences/Reverse primers	Annealing temperature (0C)	Amplified Product size (bp)	
Ca-II-SNP1211	64.60	Ca-*desi*-LG(Chr)7	2075045	TAATTTTCTGAGATGGCTCAATGAAAGTCCTTCCTACATAGTGTGACCTTATCAGCCCTTGCTGAAAAGGAACACCAGCTTTTGCAGCATAACCAAGTGC	T	A	CTGTTTACGAGTCTCGTAGACGTTTCGGGGAGATACTTGCTACCGAGAGTCCTGTTGATTGTGATGTTGTCATAGCTGTTCCTGACTCTGGTGTTGTTGC	NA	NA	NA
Ca-II-SSR373	65.48	Ca-*desi*-LG(Chr)7	2084209	GGATGCAACCTCATAAAGTTATAAAAA	(AT)9	(AT)10	TAAACTCATTTGACCAGCGG	60.05	181.00	NA
Ca-II-SNP1212	66.36	Ca-*desi*-LG(Chr)7	2091776	CAATCACAATGTTATAGATTTATTGAAACATTTTCTTTCAAAATTGGGTTTGTCTCTTCACGAACCCATTCTTTTTTCAATGGTACCATCATAATATCAG	A	G	GTTGAAGAATTTTCTAGGTTTGCTTCTGTGTTGCTGAATAAGTTTTCTGGATATATTTTCTTTTGTTTTTCTTGACATATAGACCCACAGGAAGCCAAAG	NA	NA	NA
Ca-II-SNP1213	68.12	Ca-*desi*-LG(Chr)7	2127007	GATCGCCGGTTTGGCGGTCACCTCCACATAGTTGTGAGGGGGAGATATGTTGGGTTTTGGGCTCCCTTCCTATGTGGAGAAAGGCCCAATATGTGCAAGT	T	C	TTGCTCTTCCTCTCTCACTTTTCTCTTTGTCTCTCTCTGAATTCAGCTCTTTCACACCCACTAATCTGACCTCTCTCCACTTGGATAAGTGAGACACATG	NA	NA	NA
Ca-II-SNP1214	69.00	Ca-*desi*-LG(Chr)7	2163724	AGATGGATTTTTTCTTCCATGGACCAAACCTTTTTTTCATTTCTTTCTTCTCGCCCAATACATCAGGAATGTCTTGTAAGTCTTTTAAAATTCTTGTTGT	G	A	TGGGTCATCGAGTCTTTTTGGATTCAAATCATGTTTGGAGATCAAATGCAATATCTTTTGATGGAAAACCAGAATATAGGTCTCCACCCTCCTTGTTAGA	NA	NA	NA
Ca-II-SNP1215	69.88	Ca-*desi*-LG(Chr)7	2190171	AAGGGACAATAATTCAAAGTCTTCTTTCTTTCTCTTCATTTTTTCCTTATTAAAACCCAATAACTTCAATAATTCTAGCTAATTCATTTTTGAGGTAGTT	C	A	TGCAATAAGTATGATGCAAGATCTAAGAAAAACATGGTAAATGATAATGTAGTACTAATAGTTTGATTATAAGTTCATTGCAATAAGAACAGTAATTAAG	NA	NA	NA
Ca-II-SNP1216	70.76	Ca-*desi*-LG(Chr)7	2196332	TCAGAGAAAAGTTCCCTTATATCTTCATTGGTTACTCCACGGTCCAAGTTGGAAACTTACAACTTTGTGTCGGCTTCTACTCCTTCCTGCAGCTCTAAGG	A	C	TTCTGCTGCTGCCAAGGAGGAGGAGAAGAAGGATGAATTGATGGTATAGTCTATGTTACTGATCCTGGATTTGCTATGCAGAATGAGGTGTTTGAGGATA	NA	NA	NA
Ca-II-SNP1217	71.64	Ca-*desi*-LG(Chr)7	2204432	GTTATGAATAAGTTATTGGTTCACATTGTTCCTAGAAATTGTACAACTGTGTCTGTAATCATCTGTCATGATACTTGAGGTGTTAGCTCAGTGATAAGAG	C	T	GACAGATGGGTTTTTTTTATTAATCAAAGTGATACTAATAGCCACTTTACAATTGTCCCTCAGGGGATTTGAATTCGGTATCTTGAAGTTACAAGATTAA	NA	NA	NA

Marker IDs	Genetic positions (cM)	Chromosomes	Physical positions (bp)	Flanking sequences/Forward primers	ICC 12968	ICC4958	Flanking sequences/Reverse primers	Annealing temperature (0C)	Amplified Product size (bp)	
Ca-II-SNP1218	72.52	Ca-*desi*-LG(Chr)7	2227761	TCACCTTGTATTTTATTTTATTACTTTTTTGTTGTCTCACTCGATCATTTTAATCATCCATCTTTGTGTAATTGTGAAATATTTTATTTTAAATTTGATA	G	T	ATGAATTTTGGATTTCGATTTTGAAACCAAAATATTAATCATTATGATTACTCTATTAATGTTAATAACAGTTTGAATGTTTGTAAATTAAAATTCTAAT	NA	NA	255
Ca-II-SNP1219	75.16	Ca-*desi*-LG(Chr)7	2230354	ATGTGATGAAGAGTCATCAATATTCTACCTATTTGCTAATTTTTTTTCAAAATATTTATTGTTGTTAGTCAGAGTATTATTTTTGTAGGCATATTTAATA	A	T	TCTTTTGAAGACACAATAATAAGTCTACTATGAAACTAAATATGGATATTTTTTTTTGTTTTTTTTTTTGGAAAGCCACTATACTTTCATGTTGTAGATG	NA	NA	NA
Ca-II-SNP1220	76.04	Ca-*desi*-LG(Chr)7	2262437	TTACCTGTCCAATCACTTTAGCCATGGCTGGATCATAATCTGTAAAGATTGTTTTTGGTTGTTGGTTTCCCATACTCTCCAAAAATGCTTTGAACAACCA	A	G	AAAATTTGATTTGTGCCCCCTTTGTTGGTGTAAATCATCATTATCAAAATGTCATGTTTGGTTGTGCCTTGTTATTTGATGATACTGAAGTTTCTTTTAC	NA	NA	NA
Ca-II-SNP1221	76.92	Ca-*desi*-LG(Chr)7	2282511	TACAATCCCTAATAAGTTGGACACTCTGAATTTCAGATGTATGCTCTTCTTTCCTCTTCGTCAACTCTATCATCTATAACCTCATGAAATGAAATATTAT	A	T	TAGTTTCTGTATGCTGTTTTTAATGACCCTGTTATTACTTTTTGGTTAGATGGAAGTAGGGGACACTGAATTTGAACAAATTAATAAAGAAGAACAAATT	NA	NA	NA
Ca-II-SNP1222	77.80	Ca-*desi*-LG(Chr)7	2353501	CTTCCTTCAAGTGGATGGCATGGTCATGCCTTCTAGAGGGTGGTAGCCCTTGAGGATTTTGAAATACCTCCTGAAATTGCTGCAATAACTCCTCTAACTC	C	T	ATGGGGCATTCATGCAAGTATTGTTGAAAGAAGGTGAAGGGTTGCTGATGCAAGGTGAGATAGCAACAGAACTTCAGCAGGAGAAACAGCAGGTCTCAGG	NA	NA	NA
Ca-II-SNP1223	78.68	Ca-*desi*-LG(Chr)7	2355113	TTTTTTCATTCGAGCTTCATGGAGCTCCATTCGCATCTTCAATATTTCACGATCTTTCCTCAATTCCTTGAACTCATTGTTCCCGTTGTCTTCGTTTCTG	G	A	ATCACTGGTATCAGAGCACCGATCTAGGTGCCCTGAGGGAGTGGAACCACCATACGAGCGTTGCTACAATCGTCTATGATTTTTACCAGAAGCATGGCAA	NA	NA	NA
Ca-II-SNP1224	79.56	Ca-*desi*-LG(Chr)7	2417406	ACTGTGATCCATGTTGGCCTGCTGCTTTATTCTTTGACATTAACAAAAATATTGAAATATTTTACAGGTGAGGATTGGGAAGAATGGTGTGGAGGAAATT	C	G	TGGGAGGCAAATTTGATTCCCTTCTCAATAGATGATTTGAGTTCAGACTTGAGGTTTTCAAGGCCTTGTTGCTCGAAATCTGAGAGAGGACCTAAGCCAA	NA	NA	NA
Ca-II-SNP1225	80.44	Ca-*desi*-LG(Chr)7	2427737	GTATTAGCACGATATGATGAAGAGGGTGCAAGAATTTATCGAGGCTTGTGATACTTGTCAATGTCAAAAATATGTTGCAACCACTCCAATCGGCTTGCTG	C	T	ACCACCACCAAAATAGCTTCATAGTTATTACTTTTTGGCAGACTAGTAATGAAACCCATCAAGATTTCGCTCCAAAACAACACTGGAATGGGAAGTGGCT	NA	NA	NA

Marker IDs	Genetic positions (cM)	Chromosomes	Physical positions (bp)	Flanking sequences/Forward primers	ICC 12968	ICC4958	Flanking sequences/Reverse primers	Annealing temperature (0C)	Amplified Product size (bp)	
Ca-II-SNP1226	81.32	Ca-*desi*-LG(Chr)7	2503483	ATGGCAGTACATCATCACATCTTCCTCTATCATCTAATACACATGCCCTCAATAAATCTTCCAGGGACTGATTCGTTCTCTCAGTCTGTCCGTCCGTTTA	T	C	AGTATTGTATCAGACCGTGACCTGAAATTCACGTCGCAATTTTGGGAGCGTTGCATGAAGCTTTGGGAACGAAACTAAGATTGAGCTCAGAATATCATCC	NA	NA	NA
Ca-II-SNP1227	82.20	Ca-*desi*-LG(Chr)7	2525183	TTGTTTCTTCATAAATAAGATTTAATGAATGACAATTAATTGACCATTTTGTTGATGTAGGCGTCACTTCCTAATTCTTTAATGTTTGTACCATTTTCTC	C	A	TCTGTTTTGTGTGTTTAATGCATTTTAAAGTTTCTAAAGCTTTAAAAATAATGTGATGAAGCAAATGGAGAGCAAAGCCATTAAAAGCATTCAAATAGAA	NA	NA	NA
Ca-II-SNP1228	83.08	Ca-*desi*-LG(Chr)7	2545091	GGTCATTGGAAAACACATTTTCTGAAATTGGGGAGAGCACATAAGAAGAATTTTAGGGGGCCATCGTCCAATGTTGTTGCTTCTGTTCCTCCTACCATTG	G	A	CATAGCATGTGATTGAGTGGCAAGAAGTATTTGAAGCTGTTCTGCAATATCAGATATTTGGGAAGCAGTCTCAGATGAGTATACAGAACCAGAACTAGAG	NA	NA	NA
Ca-II-SNP1229	83.96	Ca-*desi*-LG(Chr)7	2570445	CTAATTTGGATACACAAATTCAACATATACAATAGTAACATTACTTGATCTATTTTTTTGCTGACTCTGCTCTTTCTCTGCCCCTGAACAGCTTGTCTTC	T	C	AGAATAGTACCTATAAGGTCTATGCAGCATACACCTACTGACATTATTGCTGCTTCATTGACAAGGTTTCTTATATCTGCTCCAGAGAATCCAACAGTAC	NA	NA	NA
Ca-II-SNP1230	84.84	Ca-*desi*-LG(Chr)7	2574968	TTTTCTAGAGTATCCCTTCCTACCTTCATCAGTAATATCATCACCAAATACAACATGTTCAGCACATTACCGCAGTGTATCTCATGCCATGCTTCTTGTT	G	T	ATGGACGGGGGAATTGCATGAATGATGAGACCGCCTAACCAAAAGTTTCCCTCTTATGATTGTGAATCTTGATTGACTGACAGATGAAACTGATTCAGAG	NA	NA	NA
Ca-II-SNP1231	85.72	Ca-*desi*-LG(Chr)7	2652130	ATAGTGCAATTAACATTGATGGTGATCTCCTAAATCTAGTATTGTTAGTAGAAGCTGAACCTGTATCTCTAGAGGAAGCACTGGCTCACCCTCATTGGAA	T	G	ACACCCACTTCACTTCTATAGCCTTCTTATTTCTTGGCAAGCTTACCATTTCCCAGGTTTTGTTCTTGTGAATTGATCTCAATTCCTCATCCATAGCATC	NA	NA	NA
Ca-II-SSR374	86.60	Ca-*desi*-LG(Chr)7	2717077	GCCACACTAGAGTCCTCGCT	(ATTT)9	(ATTT)7	GTGACTGAAGAACCGCACAA	59.63	180.00	NA
Ca-II-SNP1232	88.36	Ca-*desi*-LG(Chr)7	2734808	GGAAGCTTCCAATGGCAGTCAACGGGTGGACTCTAGTGCTAGTCGACAGGTCGAGGCTGGAAACATGATTCAAGGTGTACTGGCAGCCTCCAGTGCCGGT	A	C	GAAAGTGGTGCACTATCTACATTAGTGCGTGTATATGGATCACATGGGAGAATATTGAGATCAACTGGGGCGACTCCCACATTTCCAGCCTCCACCTGTC	NA	NA	NA

Marker IDs	Genetic positions (cM)	Chromosomes	Physical positions (bp)	Flanking sequences/Forward primers	ICC 12968	ICC4958	Flanking sequences/Reverse primers	Annealing temperature (0C)	Amplified Product size (bp)	
Ca-II-SSR375	89.24	Ca-*desi*-LG(Chr)7	2813514	CACAAACAAAATAGATAAACAAACAAA	(AG)7	(AG)6	TTGACATCTGGTGTGGATGAA	57.94	110.00	NA
Ca-II-SNP1233	91.00	Ca-*desi*-LG(Chr)7	2821353	ACTTTTAAGATTTTTATAGAGATGGTGTAGATCCA CTATGATTGGAAGGCCATAACCCATGAAGGTTGA ATTTGATGGGTACATTGGGATTGCATGGCAT	C	T	TTGTACTACTACTTAGCACTAGCTATGGTGCTT ATGACTATTACAAGTTGGCCCAACAATGGCCAA CAACCTATTTTAGACATTCATCTCAAACCATAAA	NA	NA	NA
Ca-II-SNP1234	93.64	Ca-*desi*-LG(Chr)7	2900339	TTAAACTTTATCTATCTTTTTCTTCTTATTCACAATC CATAAAATGCAACAATCTTAGGGGCCGACTAATTT GTGCAAATATTTTTTTTGCTTTTAAAAG	C	T	TATTGATAAAGAAATTATAGAAACGTTTATTTTG AAAAAATATTTTCTATTTTTATTTTTTAAATTTAT GTTGATTTTACTTATTAATAAGAAGATAAAA	NA	NA	NA
Ca-II-SNP1235	94.52	Ca-*desi*-LG(Chr)7	2931394	GGCACACATGGCTACAATCTCTCATGATTGAACTA CATGTTCCTTTTCACACTCTTATATGTTGTTATGTG ACAACCTCATTGCTGTTCTAGTTTTGCAT	G	C	AATTCAATTCACATGTTGGTCACATGAAGGACA ACTAGAGACTTAGACAGTACCTGTTTCAAGGCA AAATGAATGTCCTATTCCATGTGCTTTGTACAA G	NA	NA	NA
Ca-II-SNP1236	95.40	Ca-*desi*-LG(Chr)7	3057424	TCGTGGATTGCCATATCACTCTTGAATCACAAAAG TAGTCCTCCCATTTTATTTCTCCCAAGTTTTGGTC CCTCAAACAGAATTTTGGTCTAAAACTTGA	A	G	TTGATTCAGATTTCAATCCAAATTTATCATGGCC GAAGAAGGAGATAATGGGTCTCTAAAATCTTCG CCATTGACACCGTATGAAGAAGCGTTGGAAGC CTTGTCATCTTTGATTACTAAACGCACTCGTGT TGGTGATGTCAATATGGAGGAGCGATTCAGCG TGCTATTCGAGTACCTAAAGATGCTTGAATTGG AGGAGGCGATTTCCAATTTGAAGGTTATCCATG [illegible]	NA	NA	NA
Ca-II-SNP1237	96.28	Ca-*desi*-LG(Chr)7	3176106	TATGTGCTTGACAAGAATGTAATCTTGCTCCTACG TATCTCCCGGTGCGATGGAGAAATCAAAGCTACG TAGTTATTGAGTAAGAAATGTTGATGTGTTG	G	A	TCGTATTTCAAAATATATTCTTTTTCCAGTCAAA CAAATTCATTTTCCTTTCTCGTCAAAAACATTTT TTTATATAACAAAACCCATTTTCTTTTAAAAA	NA	NA	NA
Ca-II-SSR376	97.16	Ca-*desi*-LG(Chr)7	3377852	TCTAACATTTGGTCACGCCA	(ACC)5	(ACC)6	AGGTGGGTGGTGATAATGGA	60.11	101.00	NA
Ca-II-SNP1238	99.59	Ca-*desi*-LG(Chr)7	3392731	TCCACATTAAATTTGCATCTTCAACCTTTTTTTGGT AGGTTTTTTTTTCTTTCATATTTTTAGAAGGCTAAT GGTCGTGTCGATTATCTAGCCAATTTAG	A	C	AGTGAGGTAGATAAGCCCCCTTACATCATAGG CTAAAATAAGTTTTAAAGAATGAAACTAATCGTG CACAAGAGTCCAATTGAAAGACTAGATTGAATG A	NA	NA	NA

Marker IDs	Genetic positions (cM)	Chromosomes	Physical positions (bp)	Flanking sequences/Forward primers	ICC 12968	ICC4958	Flanking sequences/Reverse primers	Annealing temperature (0C)	Amplified Product size (bp)	
Ca-II-SNP1239	100.40	Ca-*desi*-LG(Chr)7	3397685	CATTTGCGCTATCATATTTCTCTATGTAGATGACTTACTCATATTTGGTTCAAACATTCATGCTGTAAATATTGTGAAATCATTGTTGTGTAACAACTTT	G	A	GTAGGTTTACAGTCAAAGTATTTGTATTTCTTTAGGATCTTTTCTGACCTAGTAATCTTGATTCCAACGATTACACTCGTTTCTCTGAGGTCTTTCATAT	NA	NA	NA
Ca-II-SNP1240	101.21	Ca-*desi*-LG(Chr)7	3400244	TTCCAATGGAGAGCTTGTTTGCATTTTATAGAGATCCAAGCCCTTGATGACCGTTGGAAATCATTTTTCAAAGGATCCAAGGTTTTTCATCATAATTACA	A	G	CTTGCATTCTGATTAAAGGGAAAAAACAAGGACATGTCAAGATCAAAGCTATGCTGCGAGAGGATGGGATCTGCCTGGCCTAACAAGCTGAATGACAGTT	NA	NA	NA
Ca-II-SNP1241	102.02	Ca-*desi*-LG(Chr)7	3414861	GATGGTGAGGCATCATCAAATAATGTTGATGAAGTAGAAGTCACTAATACTCCTCTGATTTGTGGCCTTCCAGATGATATTTCTCTTTTGTGCCTTGCAA	C	G	TTTATGCTTTCTGCGATAAAAAAACCACTCGTCACTGCAAACTAAGTCCCTCCATCTTTTCGAGACAGCCTTTAGAACTGAATGATATTTTCTAGGAACT	NA	NA	NA
Ca-II-SNP1242	104.45	Ca-*desi*-LG(Chr)7	3467534	CAAAGACTATGTTCGGTCTACTAGCTGTTAAATATAGGAAGGATCCAATCATCCCTCTATACTCTTTTTCAGATATTGGAGTTCCTTCATCATCTTTGTG	T	G	GCTCTCTGGTAATTATTTCCCGTTAAAAATTCTTCACCAAATTTGTTAAACTAAAAAACCTAATCCTCTTCGGTAGACCCCCGTTACACCCCCAATCCTCTTCGACGCTGTTTGCTTAAACCCGTTGACAACCGCTTGTGAACACGTCCGCGATTCCAATTTACTTTGCGGCGAATTTCTCTGACTCTCGTTTGTCACGAATCAACTTGGATTGGAGTTGCACTCAACTCAAC[illegible]	NA	NA	234
Ca-II-SNP1243	105.26	Ca-*desi*-LG(Chr)7	3474237	CCGTTAGAGTTGTGCCAATGTATCATCACGGTACTGTCAGGAATAAATATATCACGGTTAGTGGTGGACTCTATCTAATAAACTCTTGAGTTCATCGTTG	G	T	TTTTTGAAACAATTTTCTTTTTCTCTTTTTGGTTTGTTTCTTTCTACTTATGGAGCCCACAATGCCACATCATCTTGAAATAACTTATTCATTTTTTACT	NA	NA	NA
Ca-II-SNP1244	106.07	Ca-*desi*-LG(Chr)7	3502394	TCCTATTAGCGTTGGCTCTCGAAGCGTCATTCCTTCTTGGCGACATGGTTTCCTATAAAATCATTATCAAGTAGAGTCTTAACACTACTTCAAATAATAC	C	T	ATAGGTTAGGAGTTGTTATCTGATCATGTGTCGGTTTAGGTGACTTGAGTTGTCAAGTCCAATATGATTATTATGAGTTGTTATAGTCGGACGTTAGTTT	NA	NA	NA
Ca-II-SNP1245	106.88	Ca-*desi*-LG(Chr)7	3521319	AGAGTTCTTGCAAGTGTTATGCTAATTTCGATTGTCATGTTTGAATTGAAATAATCATAGTATTTTTTATTGATTGCTTAATTTCTAAGAAGATTGTAGA	C	T	TCTTAAGTACAACAAGTCATTGATTGATAATACTCAGAAAAAGAATGCTACTGATCCAGCATTGAACAGTCCAATGTCATGAACCAGAGTATGCAGCCAC	NA	NA	NA
Ca-II-SNP1246	107.69	Ca-*desi*-LG(Chr)7	3524538	ACTACCGACCCCTTGAAAAATAGCCTTGTCCTTCAAGGCTGAAATATGCATCAAGTATCCTTCCCTTCTTGCTGCCAAATCTTTCCTCTGCACCAAAACT	T	A	GGAGAATTTCATTTTCCTATGAAAGTTACTACTTATGAGTATTGCTGGTATTGGTTTATCCACCTTTCATGGCTGGCTCAATTTTCTATTTGATAGAAGC	NA	NA	NA

Marker IDs	Genetic positions (cM)	Chromosomes	Physical positions (bp)	Flanking sequences/Forward primers	ICC 12968	ICC4958	Flanking sequences/Reverse primers	Annealing temperature (0C)	Amplified Product size (bp)	
Ca-II-SNP1247	108.50	Ca-*desi*-LG(Chr)7	3536403	ATTGTTATAATCAACTTGTTAGAAGCAACTACTCAAGTTCCAAACCTTTGTATTCAATCCCAATGGTGGTGTTATTGTGCCTTCAACATTTTGGAATTGT	T	C	CCTGACCCTCCAAATCATTCTACTCAAACGGTCTGACAACTTCAAACTTTTGAGGAACACACACCTCGACTCTACCAACCAAGTCTTCTCCACATAATCT	NA	NA	NA
Ca-II-SNP1248	109.31	Ca-*desi*-LG(Chr)7	3551690	ATATATATATATATATATACTCTAATTTGGAACCGTTGCGAATGTCTTTAAAAACTAGTGCATTTTACTTTTATTTCAATTTGGGTTCAGGGTGTTACAT	A	T	ACTATTAGACGATTGACTATTAGACGACTGACATTTGACTCACATTAAACTGACACTCAACCAACTCGGCCTGATTGGACAGACCTGCTCTGATACCACT	NA	NA	NA
Ca-II-SNP1249	111.74	Ca-*desi*-LG(Chr)7	3554353	TTCTTTTGTCTGATTGTTGACTAAGTTTAATTTAAGTGTCATTTCTATTGTTGTTACTGATTGTATTAGGCAATTAAATTCATCATCACTAGTTGATTTG	G	A	TATTTAACTTAGAAAACCAATTAAGTATAATCCATAATTCAATTTTTTTTTTCAACTCAATTAAATAGTCAAAATAATATTTCAAGTAAGTAAAGGATAG	NA	NA	NA
Ca-II-SNP1250	112.55	Ca-*desi*-LG(Chr)7	3603706	ATTTAATCACAATAATATCATTTATTTATCGATTTTTTTTAGTTAAAGTGTGGGTAACGGGTATGGGTACGGGCACTTAAGTACCCAGAGGGTAAGGGTA	C	T	GGCAATGGGTAGGGTACTACAGTACCCGTCCCCATACCCGCGATTTAAAAAAATACCCATACCCGTGCCCGTACCCTCTTGGGTATCAACTTTAATACCC	NA	NA	NA
Ca-II-SNP1251	113.36	Ca-*desi*-LG(Chr)7	3670182	TTGTCCATTTCAGGATTTATCCTATAATTTATTCTATTCCTATAATCCTGGTTCTTGATCCGAACTTCTTCCCATCTGGTCAGAAACCTGTCCTAAGGAA	T	C	CAAATATCAGCCTGTTCCTTGTGAATATGTTTTTCAATAAATTAACCGGGGTTAAACGAACATTCCCTTCTCCGGGTCTCGCTTTCTGAACGGCGCTCCA	NA	NA	NA
Ca-II-SNP1252	114.17	Ca-*desi*-LG(Chr)7	3697179	TTTTTATACTGGTTCGACTATCAATTCGATAGTCTACATCCAGTCCCGAAATCACTTACCGATTCCAGCCTTTACTAGAAATGATTTGAGTATGTTTACA	T	G	TGATCTTGCTAGAGCTTGGTGATTAGTTCTTAAGGATAGATTGGTTGTTGTTGTAGATTGATATTGAGTTAGATGTATAGGCATTGAAGTGCTGGAAAGT	NA	NA	NA
Ca-II-SNP1253	114.98	Ca-*desi*-LG(Chr)7	3702391	TTTTATGCACATTTAAGTATTATAAATGATGTGCTGATATGGCTTGTGCTATCTGATGACAATTGCATACCATCTTAGAAATTGGGTTGAGCCTAACTCA	G	A	GGGTGTGTGTGTAAGAGTCCCACATTAGATGAGATATTATGACCTCAAAATGAGTTTATATGGTGGAGGCACCCTCACCTTAGCAATCAGTTTTGTAGGG	NA	NA	NA
Ca-II-SNP1254	115.79	Ca-*desi*-LG(Chr)7	3729362	ATGTCAAGCATATTCCGGCTTTGTAATTTATTTTGTATTTCTGCTATTTGTAAGTCTTTGTTTTGATAATTTTCTTGGTCATTTCTATTATCTAATTCTT	C	A	TTTAAACTATAGAAATATGGCCCAATTCCTTAGAACCTTGAGCGAACTAGGATCCAGAAGAAGAGATAAAACCATGATACTTTCTACTTCATCATCTATA	NA	NA	NA

Marker IDs	Genetic positions (cM)	Chromosomes	Physical positions (bp)	Flanking sequences/Forward primers	ICC 12968	ICC4958	Flanking sequences/Reverse primers	Annealing temperature (0C)	Amplified Product size (bp)	
Ca-II-SNP1255	116.60	Ca-*desi*-LG(Chr)7	3815555	ATTGCTTCTAACCTTGCTACTGGGGCGAATGTTTCATCAAAGTCAATCCCTTCTTCTTGATTGTACCCTTGAGCGACCAATCTTGCTTTGTTGCGGACAA	G	C	CGAAAGAACCAAAGTATGGGAACTTGTTCCTAATCTTGGAAATAAACACATCATTGGTACACAATGGGTGTTTAAAAATAAACTTGATGAGAATGGTATA	NA	NA	NA
Ca-II-SNP1256	117.41	Ca-*desi*-LG(Chr)7	3823467	TTAAGCAACCGGGTCTCTCACCCGACCCACAAAAATCTCACGCTGTAACTTTTCTGCAACCGAGAAACGCACGATTTCAGCGCTTCTACGATTAAGCACG	A	G	CATAAACAGCATTACACTAAAGCACTTATATCATAAGCACTTATCATATAACTGTATATTTCGATTGACTGTGAGTGTTTGGCAAAATTACAGAGGATCG	NA	NA	NA
Ca-II-SNP1257	118.22	Ca-*desi*-LG(Chr)7	3845687	TTTTTTTACAACATCCAGAATTAAGGGTTAAAGTCGCCTTTGGGGCTGCCTGACTGATTTTACCCCGTCCTCCAGTAGTATCCTATGCATACACATGGAT	G	A	GCCAAACTTGAAGATGAACAGGAAGAGAAGTTGTTGCAGATTTTGAGAAAACACATGAAGGCAATCGGATGGACCTTGGCTGATATTCCTGGTATTAGCC	NA	NA	NA
Ca-II-SNP1258	119.03	Ca-*desi*-LG(Chr)7	3849555	ATTTTGACTGTCGTACCCGTATAAGGAACTTATACGGACTTCTATTCCTTGATTATTTTTCTTCTGATTTTCACGTGAAATTTTGGAGCAATCCGAGACA	A	G	AAGACCAATTCCGAACTTTGCTGGGTCTAAATTGCAGATTTCATAATCTGGATTTTTATAATAATTTTACAAAAATCATATTAACCCTGTTTTTAAGCGA	NA	NA	NA
Ca-II-SNP1259	119.84	Ca-*desi*-LG(Chr)7	3859205	GAAGGACTCATGAATGATTCTTGATAAAACTGATCCAATGATGTTAGAAACGGCCGTCTTTGAGTTTCTATGCTAGGGCACAAGTCTCCCAACTTCCCAA	G	T	CCAGGCGCGCCTGGAGCGCGTTTCCTGAGCTTGGGGCGCGCGACGCGCATCAGCAACCATAAAAACGCAATTTTTCACTGTTTTAGGGATCTTTTTGACT	NA	NA	NA
Ca-II-SNP1260	120.65	Ca-*desi*-LG(Chr)7	3870719	TGGAAGAAGTCTTTGATCAACTTGGTTGGACTTATTTCTTGCGTATCAATGAACCTCAATATCCTCACCTTGTTAGAGCTTTCTATGCTGCATCAAATGG	G	A	GCTCCATCATCTCAAATATCTAATATGTGGCACAAGGAGGCTGGGTTGATTTCCATATGAACCCCTTTGAGCACAATGTTGAAACCTTATTTCCTTTTGA	NA	NA	NA
Ca-II-SSR377	121.46	Ca-*desi*-LG(Chr)7	3913998	CACGAGTAAGAAAATGAGCACAA	(TA)10	(TA)11	CCATCCTACAAACAAAGCCC	59.46	144.00	NA
Ca-II-SNP1261	122.27	Ca-*desi*-LG(Chr)7	3917259	AATTTTGATCGCATGTTTTAATTTTATTTTCACTTTGTTTTTCTTCACTACACATAAATTTATTTGTTGAAAATCGATAATGTTCTTATCAATAACCTAT	T	C	AAGATATAGTTTACTATCTGATTTATAGTAAAAAAAACTATATTCTTCTTTTTTTTTTTTTTTTTTGCAATAATAGTAACAAACTCGAGCTCCCTAATAA	NA	NA	NA

Marker IDs	Genetic positions (cM)	Chromosomes	Physical positions (bp)	Flanking sequences/Forward primers	ICC 12968	ICC4958	Flanking sequences/Reverse primers	Annealing temperature (0C)	Amplified Product size (bp)	
Ca-II-SNP1262	125.51	Ca-*desi*-LG(Chr)7	3940097	TAGTTGATAAAGCAATGGTACATTGTTTTTTGCATGACCAACTTATGAGAGATTTACCAAGAAATTGGCAAGCTCCACTTGTGTTTTTCCTTTCAACTTT	A	C	TCTTTAGATATCTTTTTGGCACCACCAATCTTAGAATCTAGTATAAAAAATGCTGAAATCTTGACCTCATAGCTTATTGTGATGCTGACTATGCGGGAGA	NA	NA	NA
Ca-II-SNP1263	126.32	Ca-*desi*-LG(Chr)7	4002075	TTTAATCCATGACAAGTTATTCCTCAGGGTGAAAATTTTTAGGTCGAAAATTAGCCAGTGTTTTTGATGTATATAGATAATTTTTAGTGGATCTTGACTT	T	C	TCGAGGTCAATCTGTATCTCAGAAATAGCTCGCCTCACTTCAGAACGAACTGTTTCATATATATCACCAACTCCTGGCTGATTCCTTTGTACGCACTGCA	NA	NA	NA
Ca-II-SNP1264	127.13	Ca-*desi*-LG(Chr)7	4075651	AAATTCTTCTTCATCTTCTTCACTATCATCTACAACAACAATAGATTATTTGTAACTAATCTATTTGAGGAAGTGTTCTGTTTTGGATGAGGTTAAACTT	T	C	AATCCAAACCTAAAATGAAAGACAAATAAAAAAAAAATCTAAAACCAATGTAACACTTCCAACAATATATTTTCTAAACAAATTAGCAAACAATCTCCGC	NA	NA	NA
Ca-II-SNP1265	127.94	Ca-*desi*-LG(Chr)7	4081355	TTAAATAAAATATTAATGTCGCGGCCTAACTTTGGCTTTTTCCTTTGCTATTACTACTCTTGTTTTTTCCTTCTTCTTTTACGGTGTTTTTTTTCTTAGT	G	A	AACTTTATAAATACACCTTATCAAATTTCAAAAAGGGGGGGATCTGAAAAATTAACATTGCTGATAAAAAACGCATAGAAAGAAGGAGAAAAAAATTGGA	NA	NA	NA
Ca-II-SNP1266	128.75	Ca-*desi*-LG(Chr)7	4096273	TCGTCTTCGGATTGGTTACCGATGCGTGGATCTAAAGAAATTAAAAATGATCGTCTTTGGATTGGTTACTGATGTGTAGATCTATAGAAAAAATGATCGT	A	C	TACCTTCAAGATTGGACCAATTACGATCTATGCATCGGTAACCAATCCGAAGACGATCACTTTCATTTCTATAGATCTACGCATCGATAACCAATCCGAA	NA	NA	NA
Ca-II-SNP1267	129.56	Ca-*desi*-LG(Chr)7	4105738	TACTTGCCAACTCTCTTATGTCGATTTTCAACACTTCAGAAATTGCATCAAAACTTGGTGGTTGCCCAGTATATCGATAAGGATTTCCTTTCTCTAGGGA	A	G	GGCGTTGGCTCAGTACTACGACCCTCTCTTAAGATGTTTCACCTTTCAAGACTTTCAGTTGGCCCCCGACATTAGAGGAATTTGAGCGAATATTGGGTTA	NA	NA	NA
Ca-II-SNP1268	132.80	Ca-*desi*-LG(Chr)7	4131862	TGTGTCATTTATTCCTTTAACTCACTATCATTTATCGATGCAGGTTGTCCTTACTTGTTTCAAGTCTATGACTTGGGCTATAGAAACTAAGAACTCAAAC	T	C	AAATTTATTGATAAAACAACTTTAAATGACACTTGAAAAGGAAAACAGTAGTATCTACACATACATACCTTCATGTTGATCACTGCAACTTTACTGGAAG	NA	NA	NA
Ca-II-SNP1269	133.61	Ca-*desi*-LG(Chr)7	4168119	TTTGTGTGGGGCGACATACAAAGTGGTCATAAGGTCCATCTTGTTGGTTGGGACATGTGTTGTCAACAAAAAATCAATGGTGGATTAGGTGTCAAGAAAC	C	A	CCTCCCATATTTACTAATAAGAACCTTGCACCAAAGATCATCAAGGTTTTGTAATAGATTCCACCATATTTTCATGAGAAAAGTCTCATTCATACGATGA	NA	NA	NA

Marker IDs	Genetic positions (cM)	Chromosomes	Physical positions (bp)	Flanking sequences/Forward primers	ICC 12968	ICC4958	Flanking sequences/Reverse primers	Annealing temperature (0C)	Amplified Product size (bp)	
Ca-II-SNP1270	134.42	Ca-*desi*-LG(Chr)7	4191368	AAGATCCCTAAAACAGTAAAAATCTGCGTTTTTATGGTTGCTGGTGCGCGTCGCGCGTCCCAGGCGCGGAAAAACGCGCTCCAGGCGCGCGTTGGCAGAG	G	T	AAATGCAAAATACATCAGACAGCAGATGCTGTGCGCCTGGAGCGCGCCCTGCGCGCCTGGAGCGCGCCCTGCGCGCCTGGAGCGCATTTCTCAGGATCGG	NA	NA	NA
Ca-II-SNP1271	135.23	Ca-*desi*-LG(Chr)7	4201107	GATCATACTGCCAGATGTCCTCTAGCATCTCTTTCAAAGTTTCTTCACTGGGATCTAGCATCTCTTTCAAAGTTTCTTCACTGGGATTTTCGGCAGTATC	C	T	AAGAAACGGGAGAAAGCTCTACTCGACAAGAACAAAACTCTGAAATAAAAATTGAGCGACAATTTGCAAAAATAAATTTTGAAGAAAACCCCATGGATAT	NA	NA	NA
Ca-II-SNP1272	136.04	Ca-*desi*-LG(Chr)7	4241579	CGTATGCATAACACCATACGTCTTTAAGGCCTCTTTCTCCTACAAATCATCTTCAACATGTTCAATTTTCTTTCCCCATCACACTTCTTCAAAACCATTG	G	A	GCGTGTTGGTTTCAGATCGACAATGGTAGCTATGTTAAAAAGAGTAACGGGGAGCATACCATAAGGAATGTGAAAACTATCATTTGAGGACCTCTAAATG	NA	NA	NA
Ca-II-SNP1273	136.85	Ca-*desi*-LG(Chr)7	4253650	TGACAAGTTTTGGTTATTTTTTTGGAGATATCATTCAATATTTCAATTTGGTTTCCTGAAAAGGTGGGATCAGTACTTTTTAAATGAAAAGAATGCTATT	T	G	TGCTTACAGAGAAGATAGAAGAACAAAAAGGTTCCATGCTTTCAAACAAAGGGACACAAAGAAGTAAAAAATATTCTCTTGACATAAAAGTAGTGATTCA	NA	NA	NA
Ca-II-SNP1274	137.66	Ca-*desi*-LG(Chr)7	4325967	CAACATCAGTCAGGTATGAAGGTAAGGTTTAGGTTTTTCTTGATGAAGTCAAAGCTATCTTCCGGTAGTGGTTTGGTGAATATGTCAGCCCATTGGTGTT	T	C	ACATATCTTACATTCTAGAACTAAACACATAGAGATCAGACACCACTTCATTAGGGACTTTGTTCAAAAAGGGGTACTAAACATTCAATTTGTAGACATT	NA	NA	NA
Ca-II-SNP1275	138.47	Ca-*desi*-LG(Chr)7	4360750	AATGAGGAATGGAGTGCATTTGTTTCCAAATGTTTGGACCCCGCATTTGTCGTAAGTGATTAATTTATTAGCATTTGTGTTTTATTATTCATATTCGTAG	A	C	CGTGATTTTTTGTATGGGTGGGTTGGATTGCTTGCCCTTTCGCGATTTGCCTTACTAATATTTTATATAAATAAAATAGCAATTGTGTAAAAATAAATAC	NA	NA	150
Ca-II-SNP1276	139.28	Ca-*desi*-LG(Chr)7	4367175	TCTCCGTTACGTAAACTTCATACGCTCGTTTCTACGAACCCTACTCTCCTACCAACTATTCGTATTTCTGCGTTGTTCTTTTTTGTTCGTATTTCTGCGT	C	T	GTAGTCTAAGTACGTAGCAGTATAAGTTCAACATGCATTTTAGTGACAACAAAAAATTAATAAATACAATACCTGAAAAATAGAAAAACAGTAAGGTTAC	NA	NA	NA
Ca-II-SNP1277	140.09	Ca-*desi*-LG(Chr)7	4376143	TCTCCCCTTTCTTTATGTCACTGATATTTATGAGCGGTTATGTACCTTTGTCCTTTCGAATTTTTAGAGCGGATGTTATGTCGTATTTTCGTTGATTTTT	T	G	AAGGATTACGACACAACAAACCGCTTTAAAAGACTACGACACACAAACTGCAACAAAAGACTACGATACAAAAACCGCTCATAGTGGTATTAGGACCACT	NA	NA	NA

Marker IDs	Genetic positions (cM)	Chromosomes	Physical positions (bp)	Flanking sequences/Forward primers	ICC 12968	ICC4958	Flanking sequences/Reverse primers	Annealing temperature (0C)	Amplified Product size (bp)	
Ca-II-SNP1278	140.99	Ca-*desi*-LG(Chr)7	4395021	GGAGCATGTCTAGAGAGCTGTGTAAAACGAGCACTATACTCTGAAACTGTCATACCCTCCGTTTGCTCCAATCGCTCAAACTCATGAGCTAATCCATCTC	T	C	GTTTGATATAGTGTCACGTGGTAGACAAGTGGGGTCACCTCCGTTAGCATGGAGAGAGTTTTCTCAGTTGTTCATGGCTCGTTTTCTTCCAGAGAGTGTT	NA	NA	NA
Ca-II-SNP1279	141.89	Ca-*desi*-LG(Chr)7	4496371	TCACAAAGTGATAGATTCAATATATTTTTGGCTTTTTGCCTGAATTATATATGACACCTCTTCAATCTAAACCAGGTTTTGATTTTAATTTGAACTGCAC	T	C	CAAACTAATACTTACATGAGCATCATAATGTTTGGAAGTATCATCTCGTCTATTAGGATCCAAATTCTTGTTCAAGTTTAACCAGTCAAACATCCTCCTC	NA	NA	NA
Ca-II-SNP1280	142.79	Ca-*desi*-LG(Chr)7	4506937	ACTTTAATTGTCATATTGAGAGTGACTTTAACTGCCATATTGAGGGTGTAATTCTAGAACGGTTTTCTATGGTGCAGTGGAACTTCGATACAGTGAATCT	A	G	TGAGTCGCGGACTAGGTCGCTCTCTTTAAGACGTTTCACGGCACTGCCCAAATTGTGCAAGGAAGACTATCAACCACGAAGTCCCCAGGAAAAAACAGCC	NA	NA	NA
Ca-II-SNP1281	143.69	Ca-*desi*-LG(Chr)7	4516212	TTTCCATATTGGTCTACCTCTCCTCCTTTTTGAAGTGGTTTTAGGTGGTTCAATAGATGGCTGTGTAGTTTGTTTTGTAGGTGGCTCAATAGATTATTGT	G	T	ATAGGGAAGCCACAACATAGTTGCCTAAAACTGCTCCAAAAAATAAGGGAGATCGAAAGCCAATTATGCACCATCTATTTAACCACTTGCAAAATAGCTA	NA	NA	NA
Ca-II-SNP1282	144.59	Ca-*desi*-LG(Chr)7	4520667	GAGGCCTTGCGACTCACATTCGACGCCAATTGTCCGTCTTGAACTCTGGTCTTGTAGGGTAGCATTACCATTAGTAAAAGTAATAATAAAGTCATGGAAC	C	T	GGGGTTGGCACTATCCAAATTCTCCATTCCTTTTCAGTTGAATATGTTCTCTATGTACCAAGATGCCCTTTTCGTTTACTTTTCTAATTTTGATTAACTC	NA	NA	NA
Ca-II-SSR378	145.49	Ca-*desi*-LG(Chr)7	4528657	TTTCCTAATGAATTCCCGCTT	(AT)16	(AT)11	ATTTATATGAAAACCCTTGATTTGA	59.92	124.00	NA
Ca-II-SNP1283	147.29	Ca-*desi*-LG(Chr)7	4561463	ATGTTACATTGTTTGTGTTTGATTGGTTTAACCCTTTGTTTGTGAAATCGTAGTTAGTACAAGTAAGAAAGTCTTGAAGTTTGGGAGAAGCCTCTAATAC	G	A	ATTCCAAATCTCATAAAAGGCTAAATGTAGAATAATATTTCTAAGGTGAGATTAGCAAAATTTGTCAATTTATTCATGAGATGATGAAAAATTATTAACA	NA	NA	NA
Ca-II-SNP1284	148.19	Ca-*desi*-LG(Chr)7	4571592	CAACGGATTGTGAGCTTTGAATTTATGATTTGACCAATCTCTTCAGAATTAATTATACTTTGATATGAACTCTTTAGAGGCATGATGAGTTTGTACACAT	T	C	GTCCAAAGACTGCCTCTAAAGAATGACTATGAAATTGAGTCTGAAGATTGCCTCTGAAGACTGAATTTGAAGCATGCCTCTGAAGCCCTTGCATGTCTCT	NA	NA	155

Marker IDs	Genetic positions (cM)	Chromosomes	Physical positions (bp)	Flanking sequences/Forward primers	ICC 12968	ICC4958	Flanking sequences/Reverse primers	Annealing temperature (0C)	Amplified Product size (bp)	
Ca-II-SNP1285	149.09	Ca-*desi*-LG(Chr)7	4629079	CAACCGTGAGATTTTTCACTAAGTGTTCAAGCCAATATCGTTCTTGATTTTCATAGATCAATCTTGATCTTTACACAATTTCTACCTTTTTACCTAGAAA	T	G	TTGTTAAAATAAGCTTGAGTGTTTTGTAAAAATCCTTTCATGTTTTGAAAGGTAACTCGCAAAATCCTTTCTGAACTAGTAAGGTAGAACTGAGGAAATC	NA	NA	NA
Ca-II-SNP1286	149.99	Ca-*desi*-LG(Chr)7	4638355	ATCTTTTCTATTCTCTAACCATATTTCCAATCTACACTCATCGCTCTGTTTAATACGTCGATTTCTTTATAATAGCCAAACTTCAACTCAGTTTCATGTC	A	G	CAGAAGGTGACGATGCGATGAAACACGATTGTGAAGAAGATGGCCATGAAACTGAGTTTGACTACGGCGATTAAACTGAGTTTCACGATTGTAAAGAAAT	NA	NA	NA
Ca-II-SNP1287	150.89	Ca-*desi*-LG(Chr)7	4641911	TCTTACTTCCTCAATAGAGTCAATAAAACCTCTAAATCGATAGCATGTGAGGTAGGGTGATGAGTGTGTCTCTTTTTATCATGTTTTTATATTGGAACTA	A	G	TATGAATGGTGCAAACTTTGGTAAATACATAGATAAAATCTAAAAAAAAGTAATTAAAGTGAGACATCTTCCACTTTAAAATCTATTTTGTATGAATAAG	NA	NA	143
Ca-II-SNP1288	151.79	Ca-*desi*-LG(Chr)7	4650844	TCCGCGATCTGCTATTTAGTACAAATTGTTGTCAAATAGCCGCTATTGCGGCACTAAAGCACTGTAGCATAGCAGAATTTGAACAAACCGCTATTTTCTG	C	T	TGCCAAATGTGTTTTTACGTTTTTAATGAATTGGCAGTTGGCAGGAGTAGAGAGAATAAACAGGTGATAAGGCATGAAGCAGTGTTGTCAATTGCGGATC	NA	NA	NA
Ca-II-SNP1289	152.69	Ca-*desi*-LG(Chr)7	4684570	GTGAAAATGGGTGGGTAGGACCACATATTTGTAGTTGTTGATCTTGTTAAGTTGAGAAATTTTGCTCAGTGAATCGGTTTGTGTAACACCCCGATTTTTA	A	T	GTATTATATATGATATAATTATTTATGACTCTTGTCGTTTTGCGTTACGACATATTATTTTATTAAGTTGTTTTTTAAAAAAAGACATGCACTCGCGTTG	NA	NA	NA
Ca-II-SNP1290	153.59	Ca-*desi*-LG(Chr)7	4708534	TGATTCGAAGCAGGTTATCACCTTTCTCAAGAAGAACATATTCGCTCGCTTCGGCGTGCCTCGAGCCTTGATTAGTGACGAAGGCACCCATTTTCTAAAC	C	A	TGCCTGTTGGAGACTTCAACTTGCCCACTTGTTTGTGGATGGTATGGGGTTGCCACTCTATGACGCACATTATATTTCTTTAGAAGGTATTCCATATTCC	NA	NA	NA
Ca-II-SNP1291	154.49	Ca-*desi*-LG(Chr)7	4710789	TCCCATTTGACCAATTTGTGAGTTACGTGAGGGGAAAAAGGGTACCATTCACACCTTAAGTCATCAATGAAATCTTTGGCCTGCCGAACTATAGTGATTG	G	C	CATCTCTATTTCGTACCCAATCTGTGTCTGGTCTGCATAAAATGCGTAGGATTTCCTGACTGCTTGGTCTTATATGTTGTGTTTCCATAGCTAGAAAGCG	NA	NA	NA
Ca-II-SNP1292	155.39	Ca-*desi*-LG(Chr)7	4718160	TTCTTAAAATCTACACTTTTCAAACCCCACCAACAATCTCCTCAACCATTTTTTCATACATTCTTCAACCGTATCACTGCTCCCTCTCTAGATTTGTTCT	C	T	AAAGGATGTAGAGCTTTCGAAGTGAGTTTTGTGACATTTGATAGCTGGAGCAAGTGAGCTGGAAGTAGAGAAAGCAGAGAAGCCTGAATAACAGGATCATCACAAGCACTGTCTATTAATCTTCCACGAGCAAGTAAACAAAGCAAATGAACCTTGTGCACAAGTTCAGCCAATTCCTTGTCTTCAGCAGAAGCTCGTCGTATTTGTTTCTGTACAGAAGAAACAGGGG[illegible]	NA	NA	NA

Marker IDs	Genetic positions (cM)	Chromosomes	Physical positions (bp)	Flanking sequences/Forward primers	ICC 12968	ICC4958	Flanking sequences/Reverse primers	Annealing temperature (0C)	Amplified Product size (bp)	
Ca-II-SNP1293	156.29	Ca-*desi*-LG(Chr)7	4721837	ACAAGCTCTTCGTCGGTGGTACCAGCTTCTTTCATCTATTTTAACTCGTCAAAGGTTAAATAATCGTTATAATAACAGTTTGGCGTCAGAGCTATCAATC	A	G	GTATCACATTTGTCCATCTGATTGTGTTTGTGAGTTTTATTCTTGTCATTACTACCACAAACTTTGGCATTAGACACATTATCATTCATACACTGGACAT	NA	NA	NA
Ca-II-SNP1294	157.19	Ca-*desi*-LG(Chr)7	4777755	AGAGTTAAGCATACTAGCAACAACTAGTTCAATAAGAGTTATGTTCCTTTTTTTTCTTTCAACTTTACCATTCACTTCAAGTAATTATGGTGCAGTTGTT	C	T	TAACTGAAATTGAAAATCAATTTAGTATAAAGATTAAGAAGTTCCAAGTATGATTTTAGTTTATTTAATGAGTTTTATAAGTTGTCTAAAATTATACATG	NA	NA	NA
Ca-II-SNP1295	158.09	Ca-*desi*-LG(Chr)7	4875181	AAGCAAACTAATTAACAATCCCTCCTTTGAACTTAGCTCTCTGAAGAGTTCAGTTCACCAACGCTCATCCCAAGTGCTTCTCGAATCTTCTGAAATGATG	C	T	TCATTTTCTAAGGGACTTAACCAAAGAAGGTGTCATCGAATTGGTTTATTGCAGCACAAAAATGCAGCTAGCAGACATGATGACAAAGGCGTTAAAGTTG	NA	NA	117
Ca-II-SNP1296	158.99	Ca-*desi*-LG(Chr)7	4928513	TGGGTAGAAAATGCGGATGTGTTGATTGCTTTTTAAAGAAAATGCGTTCGGTTTCAATAAATTAATTTTGATGAGAAAGTTTGGTTATTTATTTGGATTT	G	T	TCCTTTTAATAAAATAAGACCAACTGAGATGCGACATCTTGTCAGTGATAGTTCAAGACGCTTGAGTTGTGACAACTTGGTGATATTAAACTTTCTTAAC	NA	NA	NA
Ca-II-SNP1297	159.89	Ca-*desi*-LG(Chr)7	4955329	TTAATTAGGTCTCCATCATTTTGGCTTCACTTGCTTGGCTCTTTAGTTAATTAATGAATTATTTGAGGTAAATGAGGTACTAATTCATGGATTATTTGAG	A	G	ACAAAGACTTATCAACTCTCAATTTATTTTTAAAAAAAGCTAACAAAAACCAAAAATTTAATCAAGAATCCACATGAAAATCATGTAATGATCTCATTTA	NA	NA	NA
Ca-II-SNP1298	160.79	Ca-*desi*-LG(Chr)7	4960519	CTATTGAATTTACTTATTTCCAATTTCATAAAATTTATGCAAATTTCAGTGTATATCTGACCAGAAGCTAAAATTGATTTGTGTCGTTTTCCCATGGTTG	T	G	CCAACCTTGTGGAGTCGATCAATGCAGTATTAAAAGGCTCGCACAACCTTCTCATCACTGCTTTGGTCCAATCAACTTATTTTAAACAGGGACACAATTT	NA	NA	NA
Ca-II-SNP1299	161.69	Ca-*desi*-LG(Chr)7	4991975	AAAATATTTGTTTCAATCGATTTGCCAATCGATTGTGTTTAAAACAGGAGCTTAGCTAATTTCATGTTAATCGGAGTGCCAATCGATTTGGTAGTATTTT	T	C	ATCGATTGGACAATCTATTGTGAGAATTTCAATCATGTTAAGTTTGGTATCAATCGATTGGGAAATCGATTAAACTAAACAATTGGTAAGAACTTTTCAG	NA	NA	NA
Ca-II-SNP1300	162.59	Ca-*desi*-LG(Chr)7	5014988	GTTCGGAGGATCCTGGTGTTGCTGTTGGAAATTTGGAGGCAGTTGCTGCTGCTGGGAGTATTGAGAGTAATTTTGTTGCTGCTGGAATAGTGGTTGTTGA	T	G	CAACAACAGCAGTTTCAACAGCAACAACAATTTCAACAACAACAATTTTCATTTCCTCCTGTGTTTCATCAGCAGTTCCCGCCTCGACCTCAATACTTGC	NA	NA	NA

Marker IDs	Genetic positions (cM)	Chromosomes	Physical positions (bp)	Flanking sequences/Forward primers	ICC 12968	ICC4958	Flanking sequences/Reverse primers	Annealing temperature (0C)	Amplified Product size (bp)	
Ca-II-SNP1301	163.49	Ca-*desi*-LG(Chr)7	5019768	AATGAAAGTCCTGAAGGTAAACAGGAGGATTTTTTGGCCTTGTTGATTTGCGAATACTAGGTGGTTTAAAAGGCTCTTCATGTGCATAATTATTTTCATC	G	A	TTGGCACTTTTAATTTCCTTCTTGAACCTATAAAACTAGATTTTGTCCATAGTAAATTGAGTAATTCTAATTTGAATGATATTAATGTCATGCCTACTTG	NA	NA	NA
Ca-II-SNP1302	164.39	Ca-*desi*-LG(Chr)7	5068949	TCCCTATCAATGCAGTCATAAGCTTTGTTGATATCCAGTTTCAATGCGACGTCACCAACTCTACCATGGGTCTTACTTTTCATGTAATGAACAATTTCAA	G	T	CTTTCAAACAGGTTAAAACATGTTCTACATAAATGTATATCTAATTAACCAATCAACTTTTGTACCTAATAGGTTCATCCTTGACAATGCAATGGCAGCC	NA	NA	NA
Ca-II-SNP1303	165.29	Ca-*desi*-LG(Chr)7	5130849	TCCTTTTAAGAGTTCATCCTCTTGTCTGTATATCCTCCAATTTTGAAGACTTGATGGATTTAGTCATATTTTTCTCCTCTTTATCTTAATGATGAGTAAC	C	G	TCTCTCTCAAATGGTTTTAATAATAACAAAATTGTTTAAAAGATTATGATTGTGGTTAATGACTAATATGCGCTTTTGAGTGAATTCATATTAGAAAACA	NA	NA	NA
Ca-II-SNP1304	166.19	Ca-*desi*-LG(Chr)7	5164973	CTCTATTTGTTTATTTTTCTAATATTTGGTATCATAGCTTTTGATTCGGTCTAAGTAAATAGAGTTCTTAGTATGTTATGTGGTTGTAGTTTTGTCTGAT	A	C	AGATGAGTCAAAATGACCACTTAATAACTAAATATGTCCTTACCTAGAACGTTGTCTAAACAACACTTTTACAATATAACATTTCTTTCTTGATGTGGAA	NA	NA	NA
Ca-II-SNP1305	167.09	Ca-*desi*-LG(Chr)7	5167898	TATAATTTAGCAACCTATAGAATTGCCGTGAAATTATTTATATCAAGATTGTATGTCGTCTGATTTTATGAATATTATGTTGATTGTTACTTTGTGAATG	G	A	CACTACTCACAATATCAATCAAAAAAATAAAAGATCAAACAATATCATATTCAAGTGATATCACAATTTCATTCAAGTAGCAAGAATACTCCAGTACATA	NA	NA	NA
Ca-II-SNP1306	169.79	Ca-*desi*-LG(Chr)7	5206401	TGACTGCATTTCTTCATTCATAGCTGTCATCCAAGTTTCCTTGTCATTATTGTTAATTGCTTTCATGTAATTGTTTGGTTCAACTATTCGAAGATCTTCA	A	G	TCTATATTTGTTTGTTTTTCATTTTCATTTTTATTTTCTATATCAAGCCTCCAATCAGATATGGGCAAGCATATTTTAATTTGTTATGCTTTAAGTGTTG	NA	NA	NA
Ca-II-SNP1307	170.69	Ca-*desi*-LG(Chr)7	5244567	TATGTGCCCCGGTCACACCTTCGTAACTTCAAATATCCGAATTTGTTGAAGTATACCTAATGCGATATTCCAACCACCCCATGAATTCGTTATTCCTAAA	T	C	GTCTTATTAGTCTAGGATTGATTCCATTTGATCCTTCTAACCCAGTTCGATCTTGATCCAATGTGGAGATAAGGTGTGTTCGTTATACCCTTCATGACTC	NA	NA	NA
Ca-II-SNP1308	171.59	Ca-*desi*-LG(Chr)7	5257100	TTCCTTTACTCGTTTAAAAAAATCGCTCGTATTTTTGATCTGTTAACTATACTTTCATTAGTCAAATTGGTCTTGTCAGTTAGTTTTTGACAAAAAAAAC	A	G	TATTTGATGAATTTTTAGGGTTTTTAGATTGTAGGAGATTATAACTTAATAAGTTTATGAATTTGAAGATGAGTGCGATGAAAGAAATGACAAAACTTAA	NA	NA	NA

Marker IDs	Genetic positions (cM)	Chromosomes	Physical positions (bp)	Flanking sequences/Forward primers	ICC 12968	ICC4958	Flanking sequences/Reverse primers	Annealing temperature (0C)	Amplified Product size (bp)	
Ca-II-SNP1309	172.49	Ca-*desi*-LG(Chr)7	5451631	TTCGGCTGCCTTTCTAGCAACCAGCCACAATGCTACAACCGGCCTCAAAAGCACCACCTTCGCTGTGTGTTTTTCCAGTGACTTTTTTTCTTCTTTCAAA	C	A	GATGCGCACTAGAAAAGAGGCTGTTGGTGTTGGCACAGCTGTAACAGAGTAAAATAAAAATAAAATAAAAACACTGACATTTGGTGCGAAACACAGTTGT	NA	NA	NA
Ca-II-SNP1310	176.09	Ca-*desi*-LG(Chr)7	5534873	TCTTCAATCTGTGTTTATCTTCAATCGGATTTATCTTACGGTTCAACAATTTACAATTTATGATTTACAATTTTGTTATTCAAACTCATCGATTCAGATT	T	C	CCTGAGCTTGAAGATGAGAAACTCCATATTTATTTTTTGCTTTTTCATTATCCTTTCTCTGAAAATTAATCCCTAAATCGCTGAATCTGAATCTGAATTT	NA	NA	NA
Ca-II-SNP1311	176.99	Ca-*desi*-LG(Chr)7	5605819	CGCTCTCTCAGAAGCAAATCAAGCCCTCTCTATCACCCCCAAGGATCCTTCCGTCCACATTCTTCGGGCCCGAGCCCTTTACATAGCGGGTCACCGCGCC	A	G	TTCATCTCAGCCCTAAACACCAACGCGTCGGCACGCTCTGTCGGATTGAGAAACTTCGCCGCCGGAAGAGATAGAGCGGAATCGAGGGATCTGATGGCAG	NA	NA	NA
Ca-II-SNP1312	177.89	Ca-*desi*-LG(Chr)7	5624378	CCATCAAATTTTTCTGCGAACAACATCGTCATCTACAGATCAGGTCATGTCTGAAAAGCGCGTCGCCAAAAGTTGTCACACGCACTCTCATGCGCTGCTA	A	C	GAGATTCTGCACACAATGGAAGTGGTTGTGTCGGAAAACTTTTCCAGCAGCGACGACAGACAGCAGCAGACGGCGGTGGCAGATCTACTTGCATGAGAGA	NA	NA	NA
Ca-II-SNP1313	178.79	Ca-*desi*-LG(Chr)7	5627374	AAAATCTTCTACGGATCTTAAATGTTTAGCCCATTTATGCTCCCGCACATCCCCGAATTCCCCAAGAAATCATAGTCATGGTTTGTCTTGGTGAGGGAGC	A	G	ATCCTATACTTGTACTGCTGCTATTATCAACAAGATGTGTCTCCTAACAACATCGTCAAGAAGACATTCAATACAAGAATCAAATACAAATATCCACTTC	NA	NA	NA
Ca-II-SNP1314	181.49	Ca-*desi*-LG(Chr)7	5659652	TGGATTAATATTTAGATATTTTTCATCATTCCTAGGCTTATTCTAATCCATTTAAGTTTTTAACAAATCTTGTGCATCCAATAAGTGTGTTTGTGTCATT	G	A	CAATGACACAAAAACACTTATTGGATGCACAAGATTGGTTAAAAAACCTAAATAGATTAGAATAAGCCTAGGGAGGACGAAAATGACCTAAATATTAATC	NA	NA	NA
Ca-II-SNP1315	182.39	Ca-*desi*-LG(Chr)7	5712266	CCTCTAATGTAACTGATTCTAAGGATGTCCATGGATGGTCATAAGTCTTTGAGAGATGGTTGTATGTCTTTCTTTCTAATATGTGACTATTGGAATTTGG	T	C	TTCTCATCAATAATAGATCAAGAGAATAATAAGAATCTCTTTTATAAATCATATTCTAGAAAGTTACCTAACAAGTAAAAAAATGTGTCAACATGTTTAC	NA	NA	NA
Ca-II-SNP1316	183.29	Ca-*desi*-LG(Chr)7	5722546	CAAGGGCTTCGTGGGTCCTTCCAAGCAACACCACTAACTCGAGCTTCGTGGGTCCTTCCAAGCAACACCACTAACTCTAAATCCCCGCAAGGATCTGTGG	T	C	TTTTCAAAAGAAAGTTATCTATATGTGTGTGGCAACATGTGTGACAATACAGTACCTACTGATCCTAATCACATGGTTGAAAGAAGCCCAAGAAAATTGTATATGCCCACAGATTTTTCACATTTTCACAGCAAAATTCATTAGCAGAGAAAGCTTTTAA	NA	NA	NA

Marker IDs	Genetic positions (cM)	Chromosomes	Physical positions (bp)	Flanking sequences/Forward primers	ICC 12968	ICC4958	Flanking sequences/Reverse primers	Annealing temperature (0C)	Amplified Product size (bp)	
Ca-II-SNP1317	184.19	Ca-*desi*-LG(Chr)7	5799441	ATACCTATACCCATTATTTTTTAAAAATACCAATTATACCCTTAATTTTTTTAAATTATTTTATTAATTCAATTTTTTTTTATTCATTTCATCTTTTCTG	A	G	GCCTGGTATTTGAGATAAATATTATCTCTACAAAGTCCCACTATTAATTGAAAAAAGTTTTTCGAAAATAAAATGTGTACTGAAAATCTTTTTTGTAGTT	NA	NA	NA
Ca-II-SNP1318	185.09	Ca-*desi*-LG(Chr)7	5805369	ATAAAGCACAGTTGTCAGTACACTTTGACCTTTTGGTTCAGGCCAACCGTCACCATAACTTATACTTATCATCGTCAGCTTCGTTTTGAAGTTGGAAGAA	C	T	CGAAGCATAACGAGTTAGATTATTATTAGGAAACAGTAATTAAAAATATGCATAGCATAGGATACATACCATTGACTTGCCGGAGCCAAGTTCCGTGCTC	NA	NA	NA
Ca-II-SNP1319	185.99	Ca-*desi*-LG(Chr)7	5859634	TAGATTTCGTCATTTATTTATTTTGATGGTCTTTGTTATATAATATGTTATTAATTTGATTTACGTATGAGTTAAAAGTATTTAAATATTGAATTGTTAT	G	A	ACGTCGTTAACGATTTAATAATTATAACAACTATTTCCCAAAACACGTATTTAAAATCATCTCTCATTTCATCGTAATTCATGGAAAACATATTCAATCG	NA	NA	NA
Ca-II-SNP1320	186.89	Ca-*desi*-LG(Chr)7	5865212	CCTTCCCTGTCTTCATTTGGCCCTTTATCAAACATATGAATGCTTGGTCCTTCTTAAAAACAGAGTTCTCTGATGTATCTGAATAAATTTGAATCAAATC	A	C	TTGTGTCTGACTGTCATCCAAACATTGTTCGATGGCATGGGGTGGAGCATGATCAAGATTTTATTTACCTTGCACTGGAACGTTGTACTTGCAACTTAGA	NA	NA	NA
Ca-II-SNP1321	187.79	Ca-*desi*-LG(Chr)7	5880777	AGCTGGGTAAATATTAAATTTTTGTTGCTTCTGGATGCAGGCTGCAAGGATATTGATGATGCACTCCACTGCTACGCTCTTCCAAATGGAAACTTTGAAG	T	C	TGCCACTTTAATCCACACACACACACAAATATATATATAGATTATCGATACATGTATCATCATTAAAATAGAAATCCTTAGACCAAGGATACGAACTCCA	NA	NA	NA
Ca-II-SNP1322	188.69	Ca-*desi*-LG(Chr)7	5885422	CTAGACTGTGTATGTGCTAATCTGATAATTGTTTTTTGGTTCTTGCGTAGGCGTCATCCAACTCCAACCAGAGAGATGCTTGAACCCTTGCTACGTGTTG	C	A	AAAAAAGAAAAATAGAAATAAAATATTTACCACAGCATGGTCAAGAGAATCAGCCAATGCTTTTGACGAGGAAACATCCAAGTTCAGGCCAACTGCAGCA	NA	NA	NA
Ca-II-SNP1323	189.59	Ca-*desi*-LG(Chr)7	6099337	GTCAATCCAACCCCATGTGAAAAGTCGGAAGCCGCCTGCCAACTCAGAGTTATATGACCTTTTACTAACTTCCTTTTGGCTGAAGTCTAAACTTTTGTGA	A	G	TTCCCTTGCTTCTTTTTCACTAGTTGGTTTTTCCCTAATTGTTCCTCGATATACCACAACCTACAGAACATTTATTTGTAGCCACATAAACATTAACATG	NA	NA	NA
Ca-II-SNP1324	190.49	Ca-*desi*-LG(Chr)7	6148005	CTGGTATGCTCTCCCAAAACATACTTTATCTTAAAGAAAACTGAATTTCTTCCAATGTTCTTCCCTTAGTCTCAGGTACCCACATTGATGTAAATGCAAC	G	A	CATGATTCATGCATCTCTTGTATAATTGTCAATATAATGCTGACATTGTTTCATTGTCTATAGGGACATTCACAATCTACACAATCGTAGCCGCTTTCAC	NA	NA	NA

Marker IDs	Genetic positions (cM)	Chromosomes	Physical positions (bp)	Flanking sequences/Forward primers	ICC 12968	ICC4958	Flanking sequences/Reverse primers	Annealing temperature (0C)	Amplified Product size (bp)	
Ca-II-SNP1325	191.39	Ca-*desi*-LG(Chr)7	6192916	TTCATCATCTTCTTCATCATCATTGTCCCTCTTCCTCTTGGGGTTTGAAGAGGCAGGGGGCAACACCTCCTGAACATCATCGTCATCATCATCAACTTCC	T	A	TATCTTGTTCAGCCAATTGGACAGCCCGAGACTCGCAATGGTGGGGGTGCTGACATTGATGATGGGGAAAACCTGGATGATGAAGAGGAGGAAGAGGAGG	NA	NA	NA
Ca-II-SNP1326	192.29	Ca-*desi*-LG(Chr)7	6202937	TGGCATTTTCTTTTTTGTGGGTGCTTCTTTTTCATTTAAAGACTAAATCATAACAAGTGTGTTGGTTATATGACTTCTATTTTTAGGAAATTTTATACAA	T	A	TGTTAAATCTTTTGCTGTTAAAATAGGAATTAATATTAAACAAGTTGGTTTTGATATTAATTCATCACTTAGGAAAGAATTAGATAATCTTGTTGATAAT	NA	NA	NA
Ca-II-SNP1327	193.19	Ca-*desi*-LG(Chr)7	6248248	TTTTCACTAGTGAAATAATTTTTTTTAAAACATTTTTCCAATGTTCCTTTTCTTTCACAATTAATCTTTAAGTCATTTTATCAATAATTTGACCCTTTTT	C	A	AAGAGCTTGACATAGAGATTAGTGAGAGACATAGAGAGCATGAGATAAGCATGGATCATGTTAAAAATATGACAACTTGGTATGAGTTGTGTCAAAGGCT	NA	NA	NA
Ca-II-SNP1328	194.09	Ca-*desi*-LG(Chr)7	6283725	TTCGTGCTCTTGGATTCCCCCTTTTTTCTCAGATCCAGTTGCTGAACTCATCTATAAATAGAGAGCTCACCTCACTTGGAAAAGCACACCAAAAGCTCTC	T	C	AGTCGATTGACTGACACTCAAATATAGCACGGAGGCTACTCAAGAAAATAAGAAGAAGAAGAAGAATATAAGGGGGAGAAGTGAGAAAAGCCTCAGACAC	NA	NA	NA
Ca-II-SNP1329	194.99	Ca-*desi*-LG(Chr)7	6286042	TCGATGGATCTCATTTTACCAATAATGGGAGCTTTGTTTGTGGTGGGTTGATTCATAATCAACATGGTAAATTTGTGTATGCTTTTATGCGTAAAATTAC	C	T	TCATATTCAATTAACACATGTTGCATGTATTGAGATTGAACAACTTGGAGGTCTGTGAGGATTGTCCATAATTCAATTCAGTTGCAAGAGTATTTGAAGA	NA	NA	NA
Ca-II-SNP1330	195.89	Ca-*desi*-LG(Chr)7	6405935	GAGCCATCAATATAGGTTAAACACCATTAATGTTCTTCTTCATAACTTCCATAGGGGGTTTAATCCATTCGTTTTTATTAGGGTTAATCAAGTTCTACTT	C	T	TACCGCCACACCAAAGTACAAAGATAAAGAGTGATTTGTGGCATTGCATTTCTAACCAAGTTGACTTTATTCATAACTCTTTGATCAAGAGTAAGGTTTT	NA	NA	NA
Ca-II-SNP1331	196.79	Ca-*desi*-LG(Chr)7	6426365	GAGTTTTGTGGTGCATTGTCATTATCTAGCTCTTGTAAAAATTATTTAGGAGTTAGAGTTTATAATGTTACAAGTTGCATTTAGACAAGCAAGGCATGAT	C	T	GTTTCATTTAATTTTTCATCATCTACAAAAATATTATGGATAAAATAGTCTCCATCTTCTCCATTATAATTTCAAGACTCATAACCACGAGTTCTTGCTT	NA	NA	NA
Ca-II-SSR379	197.69	Ca-*desi*-LG(Chr)7	6459484	CATTTTCGTCTCAACCATTCA	(AT)12	(AT)11	TGGAGACGAGGAGCATACCT	58.61	134.00	NA

Marker IDs	Genetic positions (cM)	Chromosomes	Physical positions (bp)	Flanking sequences/Forward primers	ICC 12968	ICC4958	Flanking sequences/Reverse primers	Annealing temperature (0C)	Amplified Product size (bp)	
Ca-II-SNP1332	199.49	Ca-*desi*-LG(Chr)7	6475773	AAAAAAGTTTTTACCGGAAATTTCAATCCTCTTGAAATTTCCGGTACACTGTGTAATTTTTTTTTTTAATTTTGTTTATTGAATTTTGTTAGTGAGGACCA	A	G	TTCCTCATTTCTCTTTCTCCTTTTTTCAACAGCAGCTCTCCTATTTGATTGTGTAGGAGGCCGAACTCGCGAATAATCACCACCGTCAGATTTTCCTCCT	NA	NA	NA
Ca-II-SNP1333	200.39	Ca-*desi*-LG(Chr)7	6530072	GAATTCTTCCACTGCATCTCAACAGAGACCAGTTGTTGATTGGTGGGCTGAAGATCTAACAGTTCTCAGAATTGACATTTTTCAAAGAGTTCTAGTTGCA	T	A	CCTTGTATAGTAAAGGATCTTACCAAACCTCTGAGAGATTTCTGTGAATAAAGCATTATAATAGGACCAATACCACACTGTTTAAACCCTCTTGCCATCA	NA	NA	NA
Ca-II-SNP1334	201.29	Ca-*desi*-LG(Chr)7	6565557	TCTTAGCATTTAGATATGATGCCCATTATAGTTGGTGATATACTGATTTATCAATTTTGTGTACGGCTATTGTGTTTGATGTCTAGCGATGTTCATTTGA	G	T	AAATTATGCATATTTGGTACTACAATCTCATCATTTTATTTCCTCCATCTGCCAAAAGTTTCAAATAATGGGTCGATTTAATCTTATACTAGTTTTTTCACTCTCATTAAATTTGTGTCTTATTCCATATCTTACTTTAGCTCAACTTAGTACAAACCATTATGCTAAAACTTGTCCCAATGTTGAAAGCATTGTTAGAACAGCAGTTCAAAACAAATTCAAACAAACTTTTGTTAC[illegible]	NA	NA	NA
Ca-II-SNP1335	202.19	Ca-*desi*-LG(Chr)7	6598127	ACAAAAGGCAAGTTACCTCCTCTATTTCAGGTTCATAACAATACTCCATGACCTTCTTTGTTACAGGCCTTTTGTTTCTTCCTGAACTTGAAGATTTTGA	T	C	TATTATGCTATGCCAGTGATAGCAATTTCTTGCCCATTTTGAACATGATGGTTTGATTATTTTTTTTTTCTTCTTCTGAAATGGTTGAGGATAATGATGG	NA	NA	NA
Ca-II-SNP1336	203.09	Ca-*desi*-LG(Chr)7	6627339	CTTCGGGCTTCTGATCTGACATTTGGCTTTAGCTGTTGTTTCATCACCTTCCAGTTAATACTGCATATGCCCTTCTTACCATATAAGTGGTAATATGAAC	C	T	TGTGTCCGAGTTCTACTACTTTCTGCACTTCGAACTGGATTGAAGTATGGATGGGCCTGCGTAGTATTTAAGATTTGTTAACCAAACATTCTATAAAATG	NA	NA	NA
Ca-II-SNP1337	203.99	Ca-*desi*-LG(Chr)7	6722262	ATCGCACTTTTCTCTATTTGATAAAACTCTGGCATACCGATCTATTCGAATTATTTTTATGACCCTTTACAATGGAAAAAAATCTCTTTTAAGACCTTTC	C	A	AGATTTTAGGAACTCATTAATGATTTTGTGATTTATGATGGTACTATTTGATACAAGAAATTTAGATGCTCTTGATTCTAAAATGATTGATTTTTAGTTA	NA	NA	NA
Ca-II-SNP1338	204.89	Ca-*desi*-LG(Chr)7	6729740	ATTGACACACACTGTTTTTATACTGGTTCACCCAAAGATGGCTACTTCCAGTCCTCACACCCTTGTGAGATTTCACTAATGTTCAAACAGATCAACCTCT	T	C	CCCACCCCTCCTTTTCCCTTAAAAACTCAACTCCTAGTCATATCCTAAAACAACTTGCAATATCTAACCACAATCGAGTTCAGAGTGTAACAAAGTCTTATCCTCCTCTCTCATTTCCTTCCATGCTTCCAATATTTGATATGCCGAAGGCTTGTCAATCACTCCATCGTCAACATCATCATCTTCATAGCTTTCATCATCTGAGAGCTCATCATCCGAACCATCCGAAGACTT[illegible]	NA	NA	NA
Ca-II-SNP1339	205.79	Ca-*desi*-LG(Chr)7	6747829	TAATTATCTCCATTTGTTGATTCCCTTTAGATGATAGTGTTGAATATAATGTCTTTAATTTATGATATCATGTGTCTAGTTTTCATTTGAGTATCTCTAA	C	T	GTAAATTACTCACAACAACAACCCCCAAAATCTACATTGTCGTGAAGATGGATATTCTCACTGATCATCAATGCAGTGCACCGTACTTCTGCTCTTGAAACTAAATGATCACTCAATCTTCTGCCAAGATAGTCAACCACAAACATTACCTAGCATCAGCTTT	NA	NA	NA

Marker IDs	Genetic positions (cM)	Chromosomes	Physical positions (bp)	Flanking sequences/Forward primers	ICC 12968	ICC4958	Flanking sequences/Reverse primers	Annealing temperature (0C)	Amplified Product size (bp)	
Ca-II-SNP1340	206.61	Ca-*desi*-LG(Chr)7	6769512	CCAGTAAATTCTCTCAATCTTGTTCCCCGGATGATGAATCACAACCGTCGCCGCCTCCGCAGCTTGGCAATTCCCCATTTTTCTTGATGGTAGAGTTTGA	G	A	TTTAATTTGCACTACTCTCTCTTCCATTATCTATCTTTTTCCTTCTATTCTATCTTATGTCAGAATAACACAGTTTCAGCAAGTTTATTTTTAATTGCCA	NA	NA	NA
Ca-II-SNP1341	207.43	Ca-*desi*-LG(Chr)7	6786915	CTGTAACTTATTTTTGATTAATTAGATTTTTTTAATGACTTTAGTTTTAGTTAGAATTTGTGTTTTTCTTATCTTGTTGTGTTTAGGTTAAATAATGTCA	C	T	GTGTGATCAAATTGAGAAACTCCAACATTAAAAATCAAATTGAGAGACTCAATCTCAAATTATTACAATAACACAAAGAATAGCAACAATCATTCCATAT	NA	NA	NA
Ca-II-SNP1342	208.25	Ca-*desi*-LG(Chr)7	7087458	TTTTCTTCTCTTTATCTTCATCTTTTTCCCTTGATTGATCTCTCTTTAGGAACCTGCAATTTCTTTTTATGTGACCCGTTTTGCCACAATGATAGCATAT	G	C	AGTGTTCAGTACCTCGTGGGGTGACAAGGAAGGAATTATGGGCATACAAGGAAGCCAAGTCACTTGGTGTTGGGAAGCTTTCGACATTAGATGAACTGATTTTTGACAGCCAGCAGTATTGGGATGCATTTGTGCTTTTATATGAGATGCTTGAAGAATATGGTACCCATTT	NA	NA	NA
Ca-II-SSR380	209.07	Ca-*desi*-LG(Chr)7	7091911	ACCCCGTATTTTCTTTTGCC	(TG)6	(TG)8	ATAGGACCAGCAGCACAAGG	60.18	187.00	NA
Ca-II-SNP1343	211.53	Ca-*desi*-LG(Chr)7	7160146	TACAAATCTTACATCCTCCAATATTTGTCAACTCGCCAAAATTCTGATGTGGCACATTCCAAATTCATCTTCTATTCCAATATGTTTTATTTCTTCAATA	G	A	CAATTGCACAAAGAGGGAAAGTGTCAGACAAAACCTAGCAGCAGCTTGCAGTTGTACCCACCATGAAACAGAGAAGTGCCACCTTCTATATCAATAGTATATCAAATGACGATAAGGAAGGTACTTCCAATGTTGCCAACCCTTGTGAGTTGGGGCTGATGCACCATGGGGCCATGGGTGTCTAGAAAAGAGAGCACACTAGTTACTATTCATATACAGTGAATGATG[illegible]	NA	NA	NA
Ca-II-SSR381	213.17	Ca-*desi*-LG(Chr)7	7492313	TCCTTGGTGTCCAATCCTTT	(AT)9	(AT)10	GTCTGAGGCCGATGAGGTAA	59.38	203.00	NA
Ca-II-SSR382	130.37	Ca-*desi*-LG(Chr)7	7874736	TCTATAGCCGAAAATGACAAACT	(TAA)5	(TAA)7	GGGTTTACCGTGCATCACTC	57.21	146.00	NA
Ca-II-SNP1344	213.99	Ca-*desi*-LG(Chr)7	8206701	ATTATGATGAAACATTTGCTCCGGTAGCCAAAATGACAATTGTTCGCATTGTACTCTCTATAGTTGCTTCTAATGGGTGGACTCTTTATCAAATGGATGT	C	T	GTGTGAGTTGAATGTTTTAAGTTGCTTGTCAGAAAAAAGTTGGACTATCTTACTTTATGCAGTTCATAACAATTTTGCTATTGGGTTTGATTAAAGCAGAACTTTGCAGGCATTGATGTGAATAATATGTGACCTCTAAA	NA	NA	111

Marker IDs	Genetic positions (cM)	Chromosomes	Physical positions (bp)	Flanking sequences/Forward primers	ICC 12968	ICC4958	Flanking sequences/Reverse primers	Annealing temperature (0C)	Amplified Product size (bp)	
Ca-II-SNP1345	0.00	Ca-*desi*-LG(Chr)8	42890	ACGATCTTGTTACCGCCTTTCGTGTCTAGAATATGAACGTCGACATTTTTATCGGCGGTGCCGGAGAATACCATAACTCCGTCGTTCGCCCTAAAAACCT	C	T	TTCCTGAAATCACTGGATTCATTGTAATAATTAAATGGGTAATGTAACGGGAACAGTGGCCGCGAAATTCGCGTTTTTCCCGCCAGAACCACCCACATAC	NA	NA	NA
Ca-II-SNP1346	0.85	Ca-*desi*-LG(Chr)8	99835	TTACCTGAACAACATAAGTAACAGCACATGAATTATCTTGAAGCTTCTCTACTACCCATCCTGATTGTAGCAATAGTCCTCTTATTGCATTATTTTGCTT	C	A	TGTTTCAAAGTCCACATGATGAAGTCCAAACCTTATACTGTATCCATGATTCCACTCAAAGTTATCTAGAAGGGACCACAGAAAGTACCCCCTCACATCTGCTCCTTTCCTTATTGCTGTTGCTAGAGAATCCAAGTATCCACTCAAATATTCCACTCTTTTGACATCGTTCAATACATCTTTACTTGTTGGGTAGGAATTCTCAGTCATCCCAAATCCATTTTCAGTGATG[illegible]	NA	NA	NA
Ca-II-SNP1347	3.52	Ca-*desi*-LG(Chr)8	131020	AAACATGGGAAGTGAATTTGCCTGTTGAGGAAGTGCCTCCTGAACTTCCGGAGCCAGCATTAGGTATAAACTTTGCTAGGGATGGCATGCAGGAGAAGGA	A	G	GAAGCACAACAGTGTGCCCAAATATGTTGATCTCAAGAACTGGGATGCTCAATTTGTGGATGTCGACCGTGAAACTCTGTTTGATCTTCAAACTTCTGCAGGGTACATGAGAATTGATAGTCTGCTTAAGCTGGCATGGGATAAAATAGATAGCATGATAAAAGACAAGACACCAGAGGAAATTGCTCAGTTCTATGTCAGTTGAATTTTGCTTGAAACTCATGTTACAG[illegible]	NA	NA	NA
Ca-II-SNP1348	4.41	Ca-*desi*-LG(Chr)8	156792	TAGAATCTATTCTCCTTTGTGCTTGTTGGTGACCTGATTTTATTTAATTGCAGATTTTTGTTGGCAATTTGGATCCTAATGTCACAGATGATCATTTGAG	A	G	CTAACTGATTAATTTACCTGTCAGCAAATTGGACAAATCCGCATCTCTTGCCTGCTGGAATCTTAACATGAACTAGCTCACCGTACTGCCCAAAAACTTG	NA	NA	NA
Ca-II-SNP1349	5.30	Ca-*desi*-LG(Chr)8	218174	TACACTCATCAGAGGATCAGCTTCGTGTGCATAAGAAGCAGACCTCGTTCTTAACACAGTTGACAGCAAAAACACTGCCTAAAGGTCTCCACTGTCTTCC	A	G	ATATTGCATAATGGTAAAGTCCAGGGTCTTCTAACTTCTCTTGATTGGGGAATTGTTGCTGGGAAGAATTCAAATTATAATATTCAGTTGTAAGGCGCAA	NA	NA	NA
Ca-II-SNP1350	6.19	Ca-*desi*-LG(Chr)8	219169	TTGATCCTCGTGCTTGTGGATGGGCATATGGTATGAATGTTTTTGATTTAGTAGGATGGAAGAAGCAAAACATCACAGAGGTGTACCACAACTGGCAGAA	A	T	CAGCAATGGAAATTTGAAAGAAACAAAGACACTGCAGATGCTTTTCACTTAATTATGTGATGTTTTGTTTTGGAAAAGTAAACCAAAGTGTGCTTACCAG	NA	NA	NA
Ca-II-SNP1351	7.08	Ca-*desi*-LG(Chr)8	237547	CTTAGTTCAAATATGTTTGATTACAAAATTTTGTAGAAGTGCATGACCTTCTAGCTTTTACCACTAGTTGGTTGTTGGTGAAGTACATGCAATCAACGAT	C	T	TATACTTGTAGAGAGAATTCCACAACGACTGGTTTACCTAAATTGAACTCCTTTTATTAATGCATCATTTTTCTGATTGAATTACCCAAATTGATGTTTG	NA	NA	NA
Ca-II-SNP1352	7.97	Ca-*desi*-LG(Chr)8	238144	ATGATGTATTTATACTAGTGCTTTTGCTCATGGCATTTGACAATGACAACATGACTCATAAATATATTTCTACACTTTGACCCGTTGAATGACATTTTGA	A	G	ATTTATGTATAATTATTCATAAATATGTTTGTAAGAAAATCAAATTTCATACCAAAGGTAATAAAAAAAATCAGTTGAAAAAACAAACATTGATGTAACA	NA	NA	NA

Marker IDs	Genetic positions (cM)	Chromosomes	Physical positions (bp)	Flanking sequences/Forward primers	ICC 12968	ICC4958	Flanking sequences/Reverse primers	Annealing temperature (0C)	Amplified Product size (bp)	
Ca-II-SNP1353	8.86	Ca-*desi*-LG(Chr)8	244409	TCTCATTAATTAAAATTTTCTCCCGGTACCATCTATCATACTACCCCTTATCCTTATTTGTGTTTCTACAATATTTGATTTTGTTTTATTCTTAATTAAT	G	T	ATGTTTATCAAGAAATGTAGTGCCCAAATAGTAGAAACTTTAACTCAATCGGTCTCATCTCATTGACTATCATTGTAATAGCCAAATACTGTATGTGTTC	NA	NA	NA
Ca-II-SNP1354	11.53	Ca-*desi*-LG(Chr)8	252008	TTACCTGAACAACATAAGTAACAGCACATGAATTATCTTGAAGCTTCTCTACTACCCATCCTGATTGTAGCAATAGTCCTCTTATTGCATTATTTTGCTT	A	C	CGTGTTTTAGAATCATGGCTTTAAAGTTAACTGAATGAATGAATTTGTAGGTAGTAGCAGTTGCTTCATTGCCAAAGGAGATAGCTGCTGGATTACATCC	NA	NA	NA
Ca-II-SNP1355	12.42	Ca-*desi*-LG(Chr)8	307458	AAACATGGGAAGTGAATTTGCCTGTTGAGGAAGTGCCTCCTGAACTTCCGGAGCCAGCATTAGGTATAAACTTTGCTAGGGATGGCATGCAGGAGAAGGA	C	T	GTTCTTACCTTTCATTCTTACCAAATCCAAAGCGGGCACCAAAGTAGAAAGCAACAGCTAGCAGCCATGAGTCACTGTGAACTGCAACCAGCGATAACCA	NA	NA	NA
Ca-II-SNP1356	13.31	Ca-*desi*-LG(Chr)8	392134	GTTTTCAAGGACTATATTGTCTGGGCTGACATTGCATTCTGGAACATAACATGATTCTTCGAGCATAGATCTGATTTTCTCATTTGATGTCATAATGTCC	C	A	GCTTGCATGGATGTGACTGATAGTGATCACAAACCTGTTCGATGTAAGTTCAATGTGAGAATTTCTCATGCTGATAGATCAATTAGGAGAAAAGAATTTG	NA	NA	NA
Ca-II-SNP1357	14.20	Ca-*desi*-LG(Chr)8	395111	GCACTCACTCACCAAAGAGATATAAGCAAGCCAGCAAGCTGCCTAGAACCCATACGCTCAAAAGCTTTTCCTTCTTCCAAGGCCTTCCCTATTGTATCCA	G	C	AAGAACAATTTTGTGTAATGTGTATAGTCTATCCATGCATTTACTGTCTATATAACGATTGTAGGTAGGTCTAGAAGGAAGTGCAATGGGGCAGTGGTGG	NA	NA	NA
Ca-II-SNP1358	15.09	Ca-*desi*-LG(Chr)8	396980	CCAACATTCCATGTGCCAATCAAAATTCTGATATTGTGTCGTCTTGTATAAGTAAGTTCCTTTGAGGCCAGCTCTGATCTTATTATGCTGTCAACAGGGC	C	A	TGCTCATAATAGTCCTGTGCTTAAATTGGCTGTTGGTAACGGTTCTGTGTATAGCTTGGCAACTCATGGTGGCATACGCGGATGGAATATTGCATCCCCA	NA	NA	NA
Ca-II-SNP1359	15.98	Ca-*desi*-LG(Chr)8	397569	CTTGATCCTGCTGCACCGACGACATGTCAACTCGATTTTCTGGCTGACCGTCGATGTTAAATACTTTCAAAAGGTCCTTTGAACGGGCATCCCTTTAAAA	T	G	TGGTATTCTATTTCAAACTTCACTTTTCCTGCTACTAGTTTAGTTTAGTCTTTGTGTTTCCTTCTACTCCAACTTATATATTAATAACTTGCAATCCATC	NA	NA	NA
Ca-II-SNP1360	16.87	Ca-*desi*-LG(Chr)8	406836	AGGGTTGTCCTAAGATGTTGGGTGTAACATCCTGCTTTTTCGAGATATTATCTAACGAAATTCTAAACGTAGAAATACAAATTTGTTTTTTATTTATTTA	T	A	TTACTTTTAGTAATTGTAAATATTTTGATTCATTATTTTTGAAAATATAGCTAAAAATATTATTAGTTAATTTTAAGATAAATGAGTATTTAAGATAATC	NA	NA	NA

Marker IDs	Genetic positions (cM)	Chromosomes	Physical positions (bp)	Flanking sequences/Forward primers	ICC 12968	ICC4958	Flanking sequences/Reverse primers	Annealing temperature (0C)	Amplified Product size (bp)	
Ca-II-SNP1361	17.76	Ca-*desi*-LG(Chr)8	509122	TGCGCGTCACGCGCCCCAGGCGCGCGTTGACAGAACTGAAACACGAGAAATGCGCTCCAGGCGCGTGTTGACAGAACTGAAACACGAGAAATGCGCTCCA	A	G	ACTTTCATGAAGACACCGGGACCAAATTCAGACGCAAAAGATGAGAAAAATGGAAAATACATCAGACAGATGCTGTGCGCCTAGAGCGCGCCCTGCGCGC	NA	NA	NA
Ca-II-SNP1362	18.65	Ca-*desi*-LG(Chr)8	513632	ACATAATTTTTTCCATTTAGTTTCTGGATTTCAACCTTGAGGGTATTAGAAGTTCCCTCATGGCCAAAAAAGTTGGAAGAACCATTCTGGCTGCTTAGGC	C	T	CCTTATGGGTAGCGGCGACAATGACGATGGTTTGGCAAAAAAATTAGACGTAATCAACAACCCTGACAATTTAGACAGCGTCATAAGTGGCAGTGGCAGC	NA	NA	NA
Ca-II-SNP1363	19.54	Ca-*desi*-LG(Chr)8	571310	AGGGTTGTCCTAAGATGTTGGGTGTAACATCCTGCTTTTTCGAGATATTATCTAACGAAATTCTAAACGTAGAAATACAAATTTGTTTTTTATTTATTTA	A	G	CTTTAAACCGCAAAAGTTATCTAATGGGATAAAACAATTTCATTTTGCCTAGGTAGTATGTTGAGAATGGAATCTGTTGAGCACATCTGGTGCTCGTGGGTATTAATTGAATGGAATGTTCTTTTTGTTTTTGTAAAGTAAAAATGAAGGTTGAATTATACTTTTGGTCCCTTAAATT	NA	NA	NA
Ca-II-SNP1364	20.43	Ca-*desi*-LG(Chr)8	582713	TCTGCCACACTACATTTTCCTAATTTCCTTGATCTTAATTTTCTGTTCATTAGATGATCCATTATATTATTATCTAATTAATTAAGTAGTACTCAAATGA	T	A	ACCTAATTCCCATTCTTCAAATTACTAATTTAGTCATTGGAGCAAACAAATCTCATCTAAGGTACATTCATTCATTAACCTAGCATTAATTTCACATTAA	NA	NA	NA
Ca-II-SNP1365	21.32	Ca-*desi*-LG(Chr)8	926918	GACTATCGGCGATCTTCCATTTTTTTATTTAAATTTTAGTTTTCCACGTATTCCCTTCACCAAAGATAATTGTACGAGGCACTCTTAAATACTAAAAGGT	A	G	GTTTATCTGATAAATGTTGAATTTACCGAAAAGAGTTAATTTTCCACAAGTTGGGACGTGGCGAAGTATAGTTTAAATAATTTCTCATGAGAAATCTGTC	NA	NA	127
Ca-II-SNP1366	22.21	Ca-*desi*-LG(Chr)8	1082099	TTTATTCTTTCTGAATGTACTCGACTTTAGCTTGTTATTATAGCTTTTTCCTGTAACAATGTAGTTGCTGGCTTCATACCCGGCTGATTATATCATATCA	G	T	ATACGCAATAACATATTTTATAGAGTCAATTTTCAAAGAGAGTAACATTAAAATGACTAATAAGTTATTCACAAAACCCCTTCAAAAGCAAGATAGATTA	NA	NA	NA
Ca-II-SNP1367	25.77	Ca-*desi*-LG(Chr)8	1371017	GGGGGACATCAATCCAAAGTCCATCTTTAAGGAGCTGAAGTCCACCAACCTTGTCATCTTGGAAAAGTAAAATGATACCACCAGCATCTGTGTGGGCTCT	C	A	GGTATTTGAAGAAGGTGTTTTATGGATCCAAGGGACCAAATTTTGGAACAAAAGTTAGCAACTACCCTCCTTGTCCTAAGCCAGAGCTAATAAAAGGACT	NA	NA	NA
Ca-II-SNP1368	26.66	Ca-*desi*-LG(Chr)8	1384864	TTAAACAAGCTGAAGAAACAGTTCTCAAGCTAGACTTGGATGGGCTTGTTGTTATTGGTGGAGATGACTCAAACACAAATGCATGCCTCCTTGCTGAGTA	T	C	AAAGACTACATGATGCCCTCCTCCCCCGCAAAAATAAAGACTGCCCTGTTCCTAGCCATAAATCATGCCGTAATAAAATTATATTTGCAAAAAACCTGAA	NA	NA	NA

Marker IDs	Genetic positions (cM)	Chromosomes	Physical positions (bp)	Flanking sequences/Forward primers	ICC 12968	ICC4958	Flanking sequences/Reverse primers	Annealing temperature (0C)	Amplified Product size (bp)	
Ca-II-SNP1369	27.55	Ca-*desi*-LG(Chr)8	1388006	ATATGTTCCTTGAACTGTATATAATTTATGGCATGTACCGTTAATGCTGCTTGTACATGGTCTGACCTTGTTCACATTTTTATGTTAATATAATGCAGAT	C	A	TGCAATAGTCTGAAGAGTTTTCTACGATAGTATACGTACAGTGGTAATATTTTCCTGTTGATCGAGCATCAATCATAACATTTCCAATCATTTCTGCATA	NA	NA	NA
Ca-II-SNP1370	28.44	Ca-*desi*-LG(Chr)8	1390716	TAAAATTCCATAGCATTATGAATTTCATTTATTCCAATGTTTTCTTTTATGTTTTGATGTTTATGAATTGTGTGATTTTCTGTGCAGGTAAATTCAAGCC	T	C	TCTACATGGGTTAAATATAGTTCTCGGCTTGTTACTTTTGAGGAGTAAAGAATACATAAATAGATATACCTTCAAGCTCCACCATTGCTTTCTTAATCAC	NA	NA	NA
Ca-II-SNP1371	29.33	Ca-*desi*-LG(Chr)8	1391715	CTCTATGTTGTATTGGAGAAACACTGATGATCTCGATCTTTACTGTAGGTCCGATTCAATTCACTGGCCCAGGATCTGATGCAATTAGTCACACTCTACT	C	T	TCAACAGAAACACGAGCCCTATATGATCTCCGGTTTATTATATTTGCCACCATCAAAACGAATACTCATAGCACGCCTAAGCTTGCGCACCAAGCTCCAA	NA	NA	183
Ca-II-SNP1372	30.22	Ca-*desi*-LG(Chr)8	1427874	ACCATATCAGTAAAGGAGAATATGATCCCTGATGTTATTATTATTATTTTTATTTCTTTTTGTCCAGACTTAAGATCTTTGTACCTATAGCGTTCCTAGC	T	G	TTGAAACTGAGAGTTTATCAACGTCGCTGGAAGTTATGTTTTTTATCCCAGCCGTTTCTAGCCCAGTGCTTGTCCAGTTGACGGGAACCAGCATCGCCCA	NA	NA	NA
Ca-II-SNP1373	31.11	Ca-*desi*-LG(Chr)8	1441739	TCGGAGAGAACATGATGTTTTCCTTTTCTATATGTGGTGAGTTATTGGTTATTTATGTTGCTTTTTTATCTATGATGGAACAGATAAAGAAGATGATAGT	T	C	CTATAAGGAAAAATGAAGTAGCTTTCTTCATGCAGCATTCAGCCAATCAAGCACAATTGAGTAAAGCTTTTAGGTCTTTGACAGCTTGATCAACCGCATA	NA	NA	NA
Ca-II-SNP1374	32.00	Ca-*desi*-LG(Chr)8	1465861	AGGGTTGTCCTAAGATGTTGGGTGTAACATCCTGCTTTTTCGAGATATTATCTAACGAAATTCTAAACGTAGAAATACAAATTTGTTTTTTATTTATTTA	A	G	CAGCAGATAAAATAATGAAAGTAACAATTGTTTTCAATAGATTTGCTAGTGGACTAACCGAAAGGATGCCACGAGTAAGATTTGGTTATGCACACGTGGTGAACAACAAATATGATGAGTGGAAAATGTATGCTATAGGAGGAAGTGCTAACCCAACCATTTTAAGTGAAGGGAACTTTTTTATAGGACCAAATGACCAAAATGCAAAACAGGTTACCAAGAGGGGAGCCA	NA	NA	NA
Ca-II-SNP1375	32.89	Ca-*desi*-LG(Chr)8	1467895	AATTGAAGAACAAGTACTCATTTTGAGCTGAAAGTTTGAATCTTTTGCTTGTGTCTAAACTAAATGGTCTTGTGGGTCGGTAATTATCACCATCTTGACA	A	T	CACAGAAACTCGAACTCAGAACCCCTTCTGGTAGATACCTTGTTCATAACATTACCTATTATGATCCACACATTGTTGTCACTGATCCATTCATGTGGAA	NA	NA	NA
Ca-II-SNP1376	33.78	Ca-*desi*-LG(Chr)8	1478675	TTGAATTGGTGCAAAAAAAGATTACCAAAAAGAGATGGATTCTGAATTGGTGCTAGGGCTTCATACTGGTTTGCAAAGTTTTTTTATTGCTGTGCTTTGA	A	G	GATATCATAAAGCAAGCTATCAAATAACCAATTGCAGGAAATAAAAGAAGAAGAAAAAAAAGACTTTGAGAACAGAGAAAAATGGAAAGTCAATTTCAGA	NA	NA	NA

Marker IDs	Genetic positions (cM)	Chromosomes	Physical positions (bp)	Flanking sequences/Forward primers	ICC 12968	ICC4958	Flanking sequences/Reverse primers	Annealing temperature (0C)	Amplified Product size (bp)	
Ca-II-SNP1377	34.67	Ca-*desi*-LG(Chr)8	1478723	CAATTGGTATTTGGCGGTTATGTTTGACAAAGTAC TAGCTACTAGTATGTCTACATTGGTAAGAGATACC TTAGATGGTTTGCATTTGTTTTACTCAAGA	T	C	CTTCTTCTTTTATTTCCTGCAATTGGTTATTTGA TAGCTTGCTTTATGATATCTTATTAAATCTCGAA CCTATTTGGAAAATGAAAAATGATGTTACATGTT CAATTCAGCTAATCAAGGTAGAGTGCTTCATTT GCATGATCCCTTGATTTATGGTTTAGAATATAG CTATTTTTATACACTTTACACCAGCAACAAATAT CAAGTTGATTTCATGTGAATGTATTTTGCAAGG [illegible]	NA	NA	NA
Ca-II-SNP1378	35.56	Ca-*desi*-LG(Chr)8	1485942	TGGTGGATTCTTGACCTAAAGTGTGGCTTTGCAAT ATGAATTTTGTAATCATGTGAATTTGAACAGTATG CTTGGATGTCCTTAAATTTAACCATGAGAA	C	T	TCATATTGAAGTTGCATTTAGAATTTATTGCCAT CCATACTATCCCAATTTCATACGAATAAATTATT CATATTGCAAGACACGTTTTGGGTCAAGAGTC	NA	NA	215
Ca-II-SNP1379	36.45	Ca-*desi*-LG(Chr)8	1486798	TGGGTGTTTCTTGTCAAGGCGTTGTGTGCGGGCA GGACATCATGGGAATTCATGATAAATTGAATAAAA TTTGGTTACTAATGTGACCAGTTAGTGGTTT	T	C	ACATAATATTCCAATTTTATATGAATAAAAAATT CATGTTACAAGCCAATTTTATTTTGTTAAGACAA CTACCAAATAAAAAAATTTGATCACCAACACC	NA	NA	113
Ca-II-SNP1380	37.34	Ca-*desi*-LG(Chr)8	1487952	ATTTGATATTCGAGACTAGTAGATTGCCTTCGATA AATCAAGATGATGATATTGTGTTTCAATCACCTTA GATTTGAAATTTGATCTTGTGATGATGATG	G	T	ATTTAGGACCCTTTATACTCTAAATATTAAATTT TACATCTAAGGGACAATACCATCATCTCAAATT TTGGTTTTAAGATCACATGTTCATCAAGGTCCA	NA	NA	161
Ca-II-SNP1381	38.23	Ca-*desi*-LG(Chr)8	1488017	CCTTAGATTTGAAATTTGATCTTGTGATGATGATG GTGGACCTTGATGAACATGTGATCTTAAAACCAAA ATTTGAGATGATGGTATTGTCCCTTAGATG	T	C	TTAGGAACATCTATGTTCAATTATCAATGTCGT CAAGGTACAATAACATCACTCTAATTTATCGAAT TTAGGACCCTTTATACTCTAAATATTAAATTTT	NA	NA	180
Ca-II-SNP1382	39.12	Ca-*desi*-LG(Chr)8	1491840	GGTGCCAAGGGATATCAATTTCATACTACACAATA GTCGCTTGGGTTGGAAACTGTGAGTGGAATTGTT ACATCACGTGATTTTCTGACCTTTATTATGA	T	A	CACCAAATCCTTGATTTATCGAGGCTATAAAGT CCAGGGTCTACAATCATTGGCTTCCCTCTCTTA TTCCTGAAATAAAATAAAATTGGATATAAAAAG	NA	NA	186
Ca-II-SNP1383	40.90	Ca-*desi*-LG(Chr)8	1492057	AGTTTGGCTACATCTTTTCAACTCTACACAGGTTT TATTTCCCTTCAATCTCTCACCTTATTCTGGATTTT GCTAATGTAGTATGTGTTTGCAAGTGCTT	C	A	TGATTCGAAAAAAGAAAAACATAACATAAAAGG TAGGAAAACATGCCTGTAATGAAAGGAACAAGA GGAAGCACTATGCGTGTAATGAAAGGAACAAG AG	NA	NA	156
Ca-II-SNP1384	41.79	Ca-*desi*-LG(Chr)8	1493698	ATGTGGTTTGCAATGAGTTTTTTATTCATATGAAAT TGGGATTGTATGGTTGGACGACCCTAAATTCAATC ACGAGATGCATTTTTGACTCAAAATGTGG	C	T	AGCCACATTTTGAGTCAAGAATCCACCATCTCA TGATTAAATTTAGGGTCATCCAAGCTTACTATC ACAATTTCATATGAATACACAATCCATATATCAA	NA	NA	174

Marker IDs	Genetic positions (cM)	Chromosomes	Physical positions (bp)	Flanking sequences/Forward primers	ICC 12968	ICC4958	Flanking sequences/Reverse primers	Annealing temperature (0C)	Amplified Product size (bp)	
Ca-II-SNP1385	42.68	Ca-*desi*-LG(Chr)8	1496943	TGCGATGAGTTAGAGTAATGGTATTGTACTTTGATGACATTGATACTTGTGCCAAGATGGTTCTAAATTCAATCATTTAGAATGACATAGTTGTGTCTCG	A	G	ATCAAATTTTGCATACAACTTTATCGAGACAATTCAATATCATAGCAATTTATTGAATTTAAGAGCATCTAGACTCATATCAATTTTGTATTCGAAGTCA	NA	NA	160
Ca-II-SNP1386	43.57	Ca-*desi*-LG(Chr)8	1501529	TTTGTTTGGTGCATTTGCTTTATAATTATTATTATTATTCCTATTGTTAGGTTGTTAATATTGTTAAGTTAGTTATATGTTGTTGAAGTTTGTTAAGTTG	A	G	GATTCAATGAAAAAAGTTGATTACAATATGAGCATGAAACACTTTATACATTCATCCTAATTATACTTACAAATCTTAACTAATTTGTAACTAATACTAA	NA	NA	112
Ca-II-SNP1387	44.46	Ca-*desi*-LG(Chr)8	1526844	TACTCACTGGAATTGCAGCTATAGGTTCTGTTTTTGTAGGAAATAATGGATCAATTGCAGGTATAATTGCTATGATGGCAGGTTCATTAGGTGCTGCAGT	G	T	AAGTGGACTCAACTGTTTCTTCCAACAGTTTGAAAAATCCACCACAGTTTCTATACATTTCAAAAACCATACCAACTTGTCCACCATGCTCAAAAGCATT	NA	NA	148
Ca-II-SNP1388	45.35	Ca-*desi*-LG(Chr)8	3401303	TGTAAGTGGAGTATCCGTACGGACACACTTATGATTCTTGTTGTGATGCTGCTATCTTTTTCTTCGGGTTGATGTAATTGGCTCTTTGTCATTATTATTT	G	A	AGACAGAAAATCATGCATCCACACCATCATACTTTCCATATATTAAGATGTCAGGAATATATATACACTCACGGACTTGGCTTGGTAATTGTAATCAAAT	NA	NA	177
Ca-II-SNP1389	46.24	Ca-*desi*-LG(Chr)8	3563996	CTTTTTGGAGTATCCTTCTGGTATAATGCCTTTATACAGGTAAATAAGTACTCATTCTGTCCCTTTTTTATAAGCCACTTGCTTTCATACAAATATTCGC	A	G	ATTTGAAACGCTTTCGACGTATCCAACAAAAGCCATGTTTGGAATTAATGGATGGATAGTTCCCCTGTCATGATTAGCATGTATCAAATTATCAGTTTCT	NA	NA	130
Ca-II-SNP1390	47.13	Ca-*desi*-LG(Chr)8	3670804	CAATTGGTATTTGGCGGTTATGTTTGACAAAGTACTAGCTACTAGTATGTCTACATTGGTAAGAGATACCTTAGATGGTTTGCATTTGTTTTACTCAAGA	G	T	CTATTGTTAGAAACACCTTTAAGATTGTTTAGTCCAATCTGAGATGGTATGCTTCTTAAACCATTTTCATCATCTTCAATTTTATTTTGCGAAACATCTC	NA	NA	214
Ca-II-SNP1391	48.02	Ca-*desi*-LG(Chr)8	3683992	TTTATACCATTGTCTGGGATACTGTTTAAGTCCATATATTGATCTTTGAAACAAACATGTCATGTGTTCCTTTCTAGGTGCACCATAACCCTCAGGTTGG	A	C	ATTATACTCGCCTTAGTTGCTACCTACAACTTGGAACTGGAACAAATGAATGTCAAGAATGCTTTCTTACATGGTCACCTAGAAGAGAAAATCTATGTGA	NA	NA	126
Ca-II-SNP1392	48.91	Ca-*desi*-LG(Chr)8	3684044	AAACATGTCATGTGTTCCTTTCTAGGTGCACCATAACCCTCAGGTTGGATCACATAGATTTTCTCTTCTAGGTGACCATGTAAGAAAGCATTCTTGACAT	T	C	AATTGACTTCAAGAAAATATTTTCTCCAGTTGTGAAGCACACTAGTATCAGAATTATACTCGCCTTAGTTGCTACCTACAACTTGGAACTGGAACAAATG	NA	NA	133

Marker IDs	Genetic positions (cM)	Chromosomes	Physical positions (bp)	Flanking sequences/Forward primers	ICC 12968	ICC4958	Flanking sequences/Reverse primers	Annealing temperature (0C)	Amplified Product size (bp)	
Ca-II-SNP1393	49.80	Ca-*desi*-LG(Chr)8	3710991	GCAGGTTTGACTTCCCTAGTAAAGCTCTATTCTGA CATGTGTGTCTTGTGCAGTTCTGAGTCAGAGACC TTGCCTCATTTCTTCCACAATGACAATAGCC	C	T	TTTTACTACCAAACTCTGGATGGTGAACTTGAA GAGACTGATTCAATAAATGCCAATTCTCTCTAG AAATGCTAAAAAGCTTATTCCAAAGATAACTCA C	NA	NA	100
Ca-II-SNP1394	52.47	Ca-*desi*-LG(Chr)8	3728139	ATAATCCTTCTTTGAAATAGCTTTTTGACTTTATTG GTTTAGCATTTAAATCAATTATGTCCTTTTTAGAAA GTACTCTTGACGTTTCTTCATTGATCTT	A	G	GTTTTCTGCACAATAACAACAATACTGCATCTT CTCTGAAATAGCCTGTAATTCATCTCCAACATT CTCTAGCTACACTCAAGACAGAGAATGTACCAT C	NA	NA	210
Ca-II-SNP1395	53.36	Ca-*desi*-LG(Chr)8	3742949	TCTTGAATTATGCTTTGGGTGGATCACCTGTTTTG TTTTTATATGAGGACCATCTTTTTATTATTTTCAAA TAGAATTTAATAGAAGTTAAGACTGAGGG	G	A	AAACTCAAGGTGATTCTAAATATATACATATCTT ATGAGTGCAATATATTTCCTATCACCAACTTAGT CAACTGAAGATATTGTTCCCTTTGGATATTTT	NA	NA	205
Ca-II-SNP1396	54.25	Ca-*desi*-LG(Chr)8	3747818	ATACAAGACCTTCGACAATACCATTTGTACCTTCT AGTCCTTCTATGCATGTATCTGGATTCACAAGAAC AGCACTCAGCCATGTTCTTAGATCAGAGCT	C	T	GTGTTATAATTTCCTCTGTTTTTATTTATTATTCT TATTTCCTCTGTTATTGTAGCTAACAAATTCGTC ACACAGGGAAAGATAACAGTACTGGAAATCT	NA	NA	125
Ca-II-SNP1397	55.14	Ca-*desi*-LG(Chr)8	3753564	TTTTTACTATTAACTTGGTTGATGTGAATATATTCA CATATTTATCTTTTTCATTTTTATAAATTGCGTTGA ATTTGTATTTATATATGATGTACTCCAT	G	C	GAATTAGTGGTTTATGTTGTCAGTAAGTGTAAA ATTGTTTATCGACAATGCACTATTATTATTTTTT TGGAAAAAAAAACGATATTAATTCTTTCAAAAT	NA	NA	158
Ca-II-SNP1398	56.03	Ca-*desi*-LG(Chr)8	3787620	GTGTCATCTCTGGCTAAGTGCATATTTGGTTTCAC AATGAGATTGGAAAAATCACGGTGACTCATCGTGA TTTTGCCAATCTCACCATGAATCAAACATG	C	T	AAAAGGCTCTATTATTGTTCTACATACCACTTTT TGGAATCGGTAGATCCAAGATGGTTAACAACTT AACATGGACTCAAATATGGATATTAAACTTAGT	NA	NA	175
Ca-II-SNP1399	56.92	Ca-*desi*-LG(Chr)8	3788385	TAGTTTTTAGGTTTATCATTATTTTGAATTTGGAAG TGTTGTTGTCCAATACTGCTCCTGATGGAATAGAA AATAACTTTGTTTTATATGCAGTACTATT	C	A	TGCTGCGACCAGGTAATTTCTTGTATATGCAAA TAATTCCAGGGTACGTTTATCTGGAAATTTTTG CTTCTGTTATGTACTGGCTGAGATATAAGGAAA T	NA	NA	145
Ca-II-SNP1400	57.81	Ca-*desi*-LG(Chr)8	3806673	GTAGATAGTATCAGAGTCGTTAATTTGATGTGACC ATACCTTCCAACAATTGGTGTTTTGATTCTCGTGT AGTAAATTATTGTGTCAAAGTCTCTTGTTT	A	G	TTCAAAACATTGTCAAAAACCCGCTTACAAATG TATCAAGATACATGTATACGTAGTAAAGTCGAT TCATCAACATTTTTATCATTCGATTATACTATAG	NA	NA	188

Marker IDs	Genetic positions (cM)	Chromosomes	Physical positions (bp)	Flanking sequences/Forward primers	ICC 12968	ICC4958	Flanking sequences/Reverse primers	Annealing temperature (0C)	Amplified Product size (bp)	
Ca-II-SNP1401	58.70	Ca-*desi*-LG(Chr)8	3811946	TCCAGAATATTCAGACAGAAATCCCCTCCTTATTTTCGTGGGAAATACTTTTTCTTCCTTCTCATTACAATCTCAACCATGGAGTTTATAACATGCAACA	T	G	CTACACTAGTAACAAGTCTTACAAGCTAATTTGTCATAGTAGAAAAAGAGCACTTCCCTATAAGTGAAATTGACTTTCCAATTGACAAGAAATATATATA	NA	NA	169
Ca-II-SNP1402	59.59	Ca-*desi*-LG(Chr)8	3823729	TTTGCCTTTCTATATTTTATTTTTTTGTTCCTTTTTTCCCCCTTATGATGAATGCCTCAAAAGGGATTTTATTTATGAAATAGGACTTGAAAATCAACGT	G	C	CAAAGTTCAAACTAAAACTAAAACTTTCTCAAAGCCTAGAAGAACTTTCTCATAGCCTAGAAGAACACAACATTTAAAATTAACAAAATTGTTGTGTTTA	NA	NA	145
Ca-II-SNP1403	60.48	Ca-*desi*-LG(Chr)8	3868129	CAAATGACCTATTTACGTATCACATGGCCTACCTAAGCTCTCTCTTCTCTTTTTGTTTTTCAATTTTGAACTGTAAATAGTATAAATTATTTGGATACAA	G	A	ATGGGGGATGATACATGGACACAGTTATTTCCTCATCATTTTGAAAGATCCTATCCATATCCTTCTTTTAATGTCAAAGATCTTGATACGGTTGATAACGGATGCATTGACCATTTATTTCCATCCCTTTATGAAGACGATTGGGATGTTCTTATTGCACATTTTCTCGGAGTGGATCACGCTGG	NA	NA	210
Ca-II-SNP1404	61.37	Ca-*desi*-LG(Chr)8	3903353	TTGTTTGCTATTTAATTTTATCTCTTTGGGGAAAACAATGTTACTTACTGTGATAATGTGAAGGGAGGGACCCTAATGCAGAACTGTAAATTCTGAGCAC	G	T	TAACACCTTCATCCAACATTAATTAAATCATTAAAATCAATTTACATCACTGCAAAGCCAAACAATTCTTTGTCGACTGCCCTACCCATTACAATTTGCA	NA	NA	187
Ca-II-SNP1405	62.26	Ca-*desi*-LG(Chr)8	4050848	CTTCCCCTTTGTCGGGTCTTCCTGAGTTCTCTCATCTTGGCTGGGGGCACTGGTTTACATTGAGAGACCTAGAACTTGCAACAAACAAGTTTTCGAAGGA	T	C	AAACTTACAGATTATTGAGTAGCTTCTTAATAGCCACTGGATTCCCATTGATCAGCTGGCCTTGATAAACAACTCCATATCCACCTTCACCAATAACATT	NA	NA	246
Ca-II-SNP1406	63.15	Ca-*desi*-LG(Chr)8	4051597	GAAACTACTCAGTACTGGTTTATATAATTTGGATTCTTGACCCTTAGAAATTCCCTGTTAATTTCAGGTTGTTGGTTTATGAGTATGTTAACAATGGAAA	T	C	AATGATCTTACGCTTTAGCTGTTCCAAGAAGAATTTTAATCCGAGCCTCCCATGTGAGAAAACCATATTGCCGCATGGCTCCATGAAGCCATTGCTCTAA	NA	NA	181
Ca-II-SNP1407	64.04	Ca-*desi*-LG(Chr)8	4054628	GTGGTTCCTTTGGTTGAGCAGAGCGAGTATTGAGTAGAGGCTGTAAAGCTTTGACAATGATGCTCATATTTGGTCGAAACTCAGCTTCATATTGAACACA	T	C	ACTACATAATTATTTGAAGGTAATTTGTGTTATAATGTAGTTTTTATTGATTTGTTGCACTTGTTGTATTTTTGCAAAAGATGGCTGCTGTTGCTGCACT	NA	NA	171
Ca-II-SNP1408	64.93	Ca-*desi*-LG(Chr)8	4099504	CCGACTGCTCTCGCAGTTCATATGAGTTGCAGCCACCTTTCTATTTGCAGTGCCCTGTGATATTTTGTAGGAGAATAACATCATCAAACTTCAAGCACCG	G	A	TTGTGTGGTCACCGTCAATGATGTTCTGAGGCTTTTGGTACAGGTGGGGTTCTGTGGTCAAATATATTTGCAATTTGAACTTACCTTTATATTTTATACA	NA	NA	138

Marker IDs	Genetic positions (cM)	Chromosomes	Physical positions (bp)	Flanking sequences/Forward primers	ICC 12968	ICC4958	Flanking sequences/Reverse primers	Annealing temperature (0C)	Amplified Product size (bp)	
Ca-II-SNP1409	65.82	Ca-*desi*-LG(Chr)8	4193971	CACTGAGCAGGAGGAGGATGTCCCTCAGCCTCCACAGATGGAGTAGGTCGCTGATGATATTCATGATACTTCTGCACATGCTGATGATGCTGATGAGTCG	G	A	CGAGCCACGTGATGCTCGTACTGTGTTAACACACATGTCACCGTCGGTCCTCCGGGAAATTCGGTCTGCTGATCCTGATCATCAATATCAGCAGCATCAT	NA	NA	170
Ca-II-SNP1410	66.71	Ca-*desi*-LG(Chr)8	4194075	ATGCTGCTGATATTGATGATCAGGATCAGCAGACCGAATTTCCCGGAGGACCGACGGTGACATGTGTGTTAACACAGTACGAGCATCACGTGGCTCGGAG	A	G	AACATTTATATAAATATATAATTAATTAAATAAACTTAAACACAAATAAACAAATAATTAAATATAAAAATAAATTAACATTTACCTCTCTTTCCCATAG	NA	NA	168
Ca-II-SNP1411	67.60	Ca-*desi*-LG(Chr)8	4195630	TCCGTGATGGATTATCAGTTATATTAATACCTGATCAGTGGTGAATTTGTCGGTATAATCTATTATATTTCTTGAGGCAGGAACCTCAACTCTACTCATA	T	C	GTTACACAGGTTCGAAAGTTTCGAAAATTTCAATTTTTTTTTAAATTTATTATTATTTATACATATTATTATTTATATTTATTACTATTTTTTAAATTAG	NA	NA	135
Ca-II-SNP1412	68.49	Ca-*desi*-LG(Chr)8	4228643	AATTTCTTTGTACCTTTTTATAAGTGATATCTTTTTTTATTTTTTAACTATATTAATTATTTATTTACTAACGTACTTCTAATTAATTTACATATTTTTT	G	A	TGTTGTATTAAATTTTTTAACAATTGTTTTTACATGATTATTCTTGACAAGAGATAATTAGTTAAATTATATTATATTAAAATATATTTATTGTTGATTG	NA	NA	144
Ca-II-SNP1413	69.38	Ca-*desi*-LG(Chr)8	4252710	TTGGTGGAGCAACAATGCATGCATTTAGAGGTGGAGCCCCAAAATATGCCATTGACATTCTAGATTTGTATGAGTTTGTCATTGCCCTATGTCTCACGCT	T	C	ATTTATTGAAGAATGGTGGAAAAGGTTGGAACTAATAAAAGGAGGTGATTGATTATCTGTTGTGATTTGCATTACAGGTTATGACAAATGGAAGATTTGT	NA	NA	206
Ca-II-SNP1414	70.27	Ca-*desi*-LG(Chr)8	4257596	CTTTATATTAAAATACTTTTTTTTTCTCTTATTTATAAGTCGCTTTTGTAAATGGCGTATTCCTTAAGAAAGTTCGAGTTTGCAAATGCGACTTTCTTGA	T	G	CCAAAATACCATTTGTAAAAGCAATTTCTTTAAGATTTTTTTTTTTAATTTTTGTTTAAAATTAACAAAGAAATTGTCTAATTTTTTTTTTAAAGATTTC	NA	NA	
Ca-II-SNP1415	71.16	Ca-*desi*-LG(Chr)8	4324943	ACGCGCCGCCGCCGCCCGCCCGGCTTGGCTCGGTCCGGTCCGGGTTGTGGTGTATTAAAGTTTTTCAGATGTTTGCCACGTCAAAACCCTAAAGCTGAGT	A	C	GAGCACGAAGTCCATTAACTGGCCACCAGGAACGTGCCTCAGAAATAGGAAACGTGATTGACAAGGTTCCTTGACCGGGCAAAAGTCAAAACGTGGGAAA	NA	NA	
Ca-II-SNP1416	72.05	Ca-*desi*-LG(Chr)8	4325065	CCCGGTCAAGGAACCTTGTCAATCACGTTTCCTATTTCTGAGGCACGTTCCTGGTGGCCAGTTAATGGACTTCGTGCTCTTGGATCCCCCCTTTTTTCTC	A	T	AGGGGGAGAAGTGAGAAAAGCCTCAGATATGGAGAGCTTTTGGTGTGCTCTTCCAAGTGAGGTGAGCTCTCTATTTATAGATGAGTTCAGCAACTGGATC	NA	NA	

Marker IDs	Genetic positions (cM)	Chromosomes	Physical positions (bp)	Flanking sequences/Forward primers	ICC 12968	ICC4958	Flanking sequences/Reverse primers	Annealing temperature (0C)	Amplified Product size (bp)
Ca-II-SSR424	72.94	Ca-*desi*-LG(Chr)8	4335277	TTTTCAAATATCCTTTTCATTGATAC	(TAA)16	(TAA)18	GGCCAACCATAGATAAAATATACAA	57.04	239.00
Ca-II-SNP1417	74.72	Ca-*desi*-LG(Chr)8	4369989	CAAATGACCTATTTACGTATCACATGGCCTACCTAAGCTCTCTCTTCTCTTTTTGTTTTTCAATTTTGAACTGTAAATAGTATAAATTATTTGGATACAA	C	A	TCTACAAGAAGCGTATCTATGAATATATATCTCTTTTATTATTCTTAGTAGCTTTCTATTATTATCTTACGACTTTACTCTTGTGTGTTCTAATTTTATT	NA	NA
Ca-II-SNP1418	75.61	Ca-*desi*-LG(Chr)8	4370215	ATTTCGTTCTACATTCTTTATCTTTAATAAAATATTTATTATTTTTAGATTTTTGACTTTTGATTCTTAATAAATCTTATTAAGTTTTTTCTTTGATTAT	G	A	GCTTTCTTTTTTGAATAATTTTGTTTTTGTTGTCCAATAAGTTTTTTTTATCTTTCAATGTAAATGGAGTATACATAATAAGTATAGAATATAGTAAATC	NA	NA
Ca-II-SNP1419	76.50	Ca-*desi*-LG(Chr)8	4415284	ACTAACAGTTTTTTTGTCTGAATCGTTCGGTCTTGATTTTGATTCTCTACATGTGTGATTGCACGATCTTCATCTTTATCCTTCGCTTCTTCTTCGAGTA	A	G	TCCTATCTCCGTTTCCTCTTCGATTTCTTCATCCTCCAATGAAGTTTGTGTTGCGAACAAACCAATTGTTCCGGTAGCAGGAAATGAACATGAACTTGAA	NA	NA
Ca-II-SNP1420	77.39	Ca-*desi*-LG(Chr)8	4446531	AGTTATAGGCCTTCTAAAAGGGCCCGAGCTGATCACAGGTCGTCTGCACCAAGCGATGATGATCTTGATGGGATGAATAGTTCACCTGGTAGGTCACAAC	A	G	TCTTTCTAAAAAAATAAAAATAAAATTCAATGTTGAAAGTACCTCATATTGATCATCGTCGTTTTGATCAGTAGTTGGGTTATCCTCCCTTGAGTGTCCA	NA	NA
Ca-II-SNP1421	78.28	Ca-*desi*-LG(Chr)8	4515504	TTTTTAAGTAAAAAATTTTGTTTTTTTAGGGAGGTCTAAAGCGCTTGCTTTGGTAGCCTTACCCTTAAACCGGCTCTACAGAATGAGCCGCAGATAACTT	A	C	CAGCTCTGTCGTGGACTTCCTTAGTGAAGACATAACTATTATTGCGACAAAACTCATGTTGAATAAAGTGAATACAGGTATCAAATAGTAACCATATTTT	NA	NA
Ca-II-SNP1422	79.17	Ca-*desi*-LG(Chr)8	4521706	ATAACACGATCAACATGGCCTTCATCTCCCCCCTCAACATTGCGCTCAACTTCCTCCTCAACAGTTTCATCAACAAAGCTCTCAACTTGACCCACTCTCT	T	C	GGAAGAAGGCGCCCAGAAGTGACGAGCAGTCGCGGTCGAAGCTTGGCTCTAGGCAATAGGCTAGTAGTAAGAAGTTGAGGTGTCTGAAATTGCAAGGACG	NA	NA
Ca-II-SSR425	80.06	Ca-*desi*-LG(Chr)8	4611654	TAGATGAATTGCAATGCCCA	(GA)9	(GA)8	CTTTTGCATCGCATCAGTTC	60.04	140.00

Marker IDs	Genetic positions (cM)	Chromosomes	Physical positions (bp)	Flanking sequences/Forward primers	ICC 12968	ICC4958	Flanking sequences/Reverse primers	Annealing temperature (0C)	Amplified Product size (bp)
Ca-II-SSR426	81.84	Ca-*desi*-LG(Chr)8	4714629	TGGAGTACTTTAGATACACAAAGGTC	(TAA)19	(AAT)18	TTCAGCTAAATAAATTTGAAGATGTTG	57.22	129.00
Ca-II-SNP1423	82.73	Ca-*desi*-LG(Chr)8	4930608	ATGCTGCTGATATTGATGATCAGGATCAGCAGACCGAATTTCCCGGAGGACCGACGGTGACATGTGTGTTAACACAGTACGAGCATCACGTGGCTCGGAG	A	G	GAAGAAACCCATTAAGTAGCTGCTGCGGAAGCATTCTGCATCTCCACAAATATCAAAGAATAGGTTTCGCAGCAGCACACTGATAATTAAATTAAATAAATAAACAAATGGCTCTCTCTCTCTCACAAAGTAACCGTCTCTGTACCCTTCTTGCTCAATCTCTCCCGCTGTTTCATCGGCGAGGGTTTGCAGCCGCTTCTGATGCTTCAGTGAGAATTGAATTGGGTAG[illegible]	NA	NA
Ca-II-SNP1424	83.62	Ca-*desi*-LG(Chr)8	5010723	ATTGACTGCTTCAACAAAAACATCATCTTTGTCATCATCACCGCCTTCCTCCTATGCGTCATCTTCTTCATCAGCAGTTACCTTAGCAATAGGTCTCGGA	T	G	GTTAGCGGCGAAGACGAAACTACTGAGCCCGCCATGCCCATTCTCATTTATCTCGATGCTAAATCCACCGTTCAACAAGAACAACCGTTCGATGAACAAT	NA	NA
Ca-II-SNP1425	84.51	Ca-*desi*-LG(Chr)8	5062175	TATCTTTTGTGGGTATAAAGTTGTTAGAATTTGCAATAGATCTTTTCTCTTGTCTTTGTTTTGTTGTTTCTTTCTACCATTGACTTTTCTTGATCCCATT	A	G	ATTATTTGCATATATACATATATAAAAAGTTGTTATTTAATTTCTTCATGTGTAATACTAATACACGAGTCTAATAATTAGTTGATTAATATATAGGCAA	NA	NA
Ca-II-SNP1426	85.40	Ca-*desi*-LG(Chr)8	5111434	CTTTGCAACCCACGGTGGTCTCCTCTTCAAAATCTAAGATTTGGTACAAGAGAAGAGCTTCGTCCATCCATCATCGCCGCCGCCCTTCTACCTTACCAAC	G	T	CAGCAATAAGAAGATGGGGGATATACTGTCATTCTATTATGGGAGATTTTACAAGTCTGACAAACATCAGAGATGGGCTGGAAGTAGGAAAGTGAGAAGCAGGAAATACATTTTCGGGCAAAAGATTTTCACTGGACCAAGGCAAC	NA	NA
Ca-II-SNP1427	86.29	Ca-*desi*-LG(Chr)8	5125571	AAATGAACCATCCATCTCAACACCATTTTCGTCTTTGGAGATTTTAAATCCTGGGACCATTGGATTTTCAACAGAATTGCTATGCTCCATTCCAAATCTG	T	C	TCAGATTTGGGTAAGATGAGGTATTTTCTTGGTATTGAGGTAGTTCAGTTTGATGGCGGCATATTCATCAGTCAACAGAAGTATGTGACAGAAGTAGTGA	NA	NA
Ca-II-SNP1428	87.18	Ca-*desi*-LG(Chr)8	5125998	GCTTTGTTAAGCTTATATACCTTATGCTCCTCATCCTTTATTTCATAACCCCTTGGCTGCTCCACGTACACATTCTCAACTAACTTCCCATGAATAAAGG	A	C	AGAAGTATACGCTCCTGTAGCTCGTATGGACACGGTTCGAATGATTTTGGCACTTGCTGCACAAAGAGCTTGGTGTGTGTTTCAGATGGATGTGAAGTCA	NA	NA
Ca-II-SNP1429	88.07	Ca-*desi*-LG(Chr)8	5199404	TTTATTAGACACATAAGGTGGATAATCTTCTCTTATATAGATTTTCTCTTTATATAAGAGGTATAGATCAAACTTCTGACCACTTGCTTAAGGTGTTTGA	G	C	GTATTTGAAGAAAATAGAACGACAATTTTCTTATTCTTATATACAATGTGATATGATTTGTAGGAGACTCTCAAATTAAATATATTTGAGTGGTAAGGGA	NA	NA

Marker IDs	Genetic positions (cM)	Chromosomes	Physical positions (bp)	Flanking sequences/Forward primers	ICC 12968	ICC4958	Flanking sequences/Reverse primers	Annealing temperature (0C)	Amplified Product size (bp)
Ca-II-SNP1430	88.96	Ca-*desi*-LG(Chr)8	5205782	GGAAGAAGCCCTTACCTTAATGTTAGCTTCAACGTGGTTTTCCGATACCGCGAGTAAAGCCGGTAAAATCTTTGCTCGCAACGTCGCCTCCAAATTGGTC	T	C	TTCTTTTTTTTTTTTTTTTTGTCATTGTGTGAGGGGACATTTTATTTTACAATAATGTGTTATTAGCCTTATGCTAATTTCATTTCATACCTACCACAGTTTTGGACCTTTGGTTTCAATTTTTCCCCTTTTTTGATGAGTAACATAGATGGTATAAGGCTGTGTTAGTTTGCTATAAAT	NA	NA
Ca-II-SNP1431	89.85	Ca-*desi*-LG(Chr)8	5236445	GGGAACCCTTCCTTAAAAGCTAGCTACCAAGAGGGAAGAACCTGGTCAATTAAATACTCCGCTGAACATCCCATACTACTCGATGTGGGACTTGGGCACC	C	T	CTTCAAATCAAACATGCCATCACAAGTGGTACATTGGTATTTGTAGCCTAAATGCTGGTTGGTTTCATTGTATCAAATTGTTAGGGAATTGGGTATTATG	NA	NA
Ca-II-SNP1432	90.74	Ca-*desi*-LG(Chr)8	5462785	GTTAACATCACTATACACATATTGTAATAATCTTTGTCATACTATTGAATACTATCGATATATCTCTCATTTTATTTTCTGTTTTGTTGGGTTCAACTAT	G	A	AATCGATGAAAATAGATAAATCAAAACTGTAATTGAACCATTAAAATTTTGAATTATTTATTCAAGATGAATGTCATAAATCTGACGCTATGTTGAGAAG	NA	NA
Ca-II-SNP1433	91.63	Ca-*desi*-LG(Chr)8	5519042	TAATTAATTTGTATTTGATATTGTTTTTTTTTCTCTTATACTCTTATCTAAGAGTATTTTGCGAAATAGTTCGAATATTTACTTAAGAGAATATTGATGT	C	A	ATAATTTAATGTGGAAAAATATATTTGTATAATGAAAACTGTAAAATAACAATGAATTTTTATAGAACATAGATATTTCTTAAAGGTTCCACAACTCAGG	NA	NA
Ca-II-SNP1434	94.30	Ca-*desi*-LG(Chr)8	5573153	ACAGAGTAACTTGACGGGAAAATTTGTTAATTTCTTAGGTTGTGAACAACAGCTTGAATCCTGTTTGGAATCAAACATTTGACTTTGTAGTTGAGGATGG	A	C	AAAAAAGGCAATCTTAGTAACTAATTCAATATGGTGCAAAATTTATTACCTTTCCAAAAGTGTCGTGGTCATAAACTTCAACAAGTAGCATTTCATGTAA	NA	NA
Ca-II-SNP1435	95.19	Ca-*desi*-LG(Chr)8	5612410	GAGAATTTTCATTTTAGTCTAGTTTGGAGTTTATTTCTTATAAAGTGTCATTTATTAATGATGTGAAATTTCTTTAAGTATGTGTGACATTGTTGATTTG	A	G	GACTAAAAATAGTGGACCACAAGATCGGTTACAACCTTCTTTTTTTCAATTTTCATTGATCAAATGTAAATTTATGCTTCTAAAATCAACTGTTTTTGGT	NA	NA
Ca-II-SNP1436	96.08	Ca-*desi*-LG(Chr)8	5663720	CTTTGCAACCCACGGTGGTCTCCTCTTCAAAATCTAAGATTTGGTACAAGAGAAGAGCTTCGTCCATCCATCATCGCCGCCGCCCTTCTACCTTACCAAC	G	C	TAGGGAGGGAGAGAGACAGAGAGGGAGGGGATGAAGAGATAAAAATAAAAAACAATAGAAAATAAATTGCCATCAAACTAACAAAAAAGAGAGAATCCAC	NA	NA
Ca-II-SNP1437	96.97	Ca-*desi*-LG(Chr)8	5676823	AGAATTTGCCATTGGTACGACATGTTGCTTTTTTCATTCATTTCTTTCTGCATCAGTCGTCGTCTTACAAACTCCATCAATTATATAAATGAACCAATTA	A	G	ATTTTTATTGCGATTGGATATATACATTTTTTTAATTAAGGTTGCCAACTCAATGGTAGAATTCTGGATTTGCAACTTATTTTTTACACATTTCTATGAA	NA	NA

Marker IDs	Genetic positions (cM)	Chromosomes	Physical positions (bp)	Flanking sequences/Forward primers	ICC 12968	ICC4958	Flanking sequences/Reverse primers	Annealing temperature (0C)	Amplified Product size (bp)
Ca-II-SNP1438	97.86	Ca-*desi*-LG(Chr)8	5705748	TCTAGGGTTGGTTCTTAAAACACTATTTAGTATAGTTTTTATTTTGTTTTTGCTTCTTTTTTTTTCCTTCTTTTGTCTATGCAAGTTTTTTTTTAATAGA	T	C	TTGGGATATGAATGAATTACATGCCGGAAGTCTATATTTCAATATCTAACATTAGTATTTTTTTCTCCCTGTAAAATATATACTAACTATAATAATATTC	NA	NA
Ca-II-SNP1439	98.75	Ca-*desi*-LG(Chr)8	5715980	CGAAATCTTCCCTCTTTGCCCTCAATAATATCTGAAAACGATTTGCAAACTTTATTATGATTGTCCCTCATTGATTGTCCGCGGATTCCATTATCAAGAA	A	G	ATTAATGATATCATCTAGTAATCGAAAAATACCTATTCGTAACATGTTTATATTTTGTTATAGAATGAGTATCTATTTAATTTTCTTAATAGAAATTACA	NA	NA
Ca-II-SNP1440	99.64	Ca-*desi*-LG(Chr)8	5741859	ATAAAAGCAGTTTGCTTAATGTATTATTACATTGCTTGGGGTATTCCTCTCTGGTTTACAGGTTTGCAGAAGCTTACCACATTAAATGTTGAAGGATGCA	G	A	AGAACAAAAAAGTTGATGTCTAAAATCAAGAAAAAATTAAATAAGAGAATGAAATGAGAATTTTTTACCAGAAATATACTCAAAAAATGCAGCAGTAATA	NA	NA
Ca-II-SNP1441	100.53	Ca-*desi*-LG(Chr)8	5885427	CATCACTTCACCTCATCAGTGCATGCCCCACATTCCTGGCTTCCAACTTCCAACTATACACTCTTTTCATTATATTTATTCTTTATGGAAATGGCAAATC	C	T	TTGCGTCTCAGTTACAGAAGAGCTTTTTTTACCTTTGATTCCAATTCAAAACTAAACAAGGCTAAACTAATTCAATCCTTGTGTCGGAAATTTCCGGAAT	NA	NA
Ca-II-SNP1442	101.42	Ca-*desi*-LG(Chr)8	5919381	TCTCTAAGTTGCTTTTAAAAGCCCAAGAAAACCATACCATCTATGCTATCTAGATGGTGTATTATGCCCCAACCATCTCTCATTTTTTCTATGTTGTTTT	T	C	GTTTTAAAATAGAGACTCTAAGAAAAGTCACTTTTACAATTTAGGATAAAGTCTTCATTTAACATTTGACCTTTTTCCAAACTCTATCAATCAACTCTTT	NA	NA
Ca-II-SNP1443	102.31	Ca-*desi*-LG(Chr)8	5943582	TCCTTTTGGATTAATTTATATCCTTTTCTCATTCTTTCAATATAGAATTTATTTCCACGTTTTCTATCGAGATTAATAATAATAAGCATATCTGTTACAT	C	T	TTTTGTTTGTACACCGACGGGAAAAATTTAATAAGAAACTCATCCATAGAAGTAACTCCTCCTGTTAACCATGGCACATGCATCAATTATCCAATAAATG	NA	NA
Ca-II-SNP1444	103.20	Ca-*desi*-LG(Chr)8	5966950	AAATTATTGGGAATAAGAGTAATACTTTGTTTTGGAAGGATTCATGATTGATTGGTGGGGTCTTGCGTGAGAAATTTAGTAGATTATTTAATTTGTCTTT	G	A	ATTTCTCCTCCCACGCAAACAACTGTCTTCGCCATCTCCGTCATTGAATACCCTCCCGTCGTAACATCTCTACCATAACCTCTACATTTTCCTCTTGTTG	NA	NA
Ca-II-SNP1445	104.09	Ca-*desi*-LG(Chr)8	5967238	GATCATATTCGGTAAATGAGGCATATTCTGTATTACGGGAGGAGTTTGTCGCTAGTATCAACACACCTCAATTTCAAGACCTAGTGTGACATCCTTTTGT	C	T	TTGATCTTCCGCAAAAATGATACCTCGACGAAATAAATTTTCTTTTGTAGGCAACCTATGTTTAAACAACCTCCACACAAATAATGATATTTTTTGCTTG	NA	NA

Marker IDs	Genetic positions (cM)	Chromosomes	Physical positions (bp)	Flanking sequences/Forward primers	ICC 12968	ICC4958	Flanking sequences/Reverse primers	Annealing temperature (0C)	Amplified Product size (bp)
Ca-II-SNP1446	104.98	Ca-*desi*-LG(Chr)8	6009417	GCGTGTGAGAGTGCGTGTGACGACTTCTGGTGACATGTTTTCAGGCGTGGGCTGATCTAGATAGAACGAAGCTTATGGAGAGATTGTTCTCTGGAAAAGC	T	A	AAACAACCGTTATTTGTTTTTGTCAACCCTTTTTCAGCACTCGTTTCAACATTTTCTGTCCAGATCCACTGCTATCACCATCGTCACCCTTGTTTCCGAC	NA	NA
Ca-II-SSR427	105.87	Ca-*desi*-LG(Chr)8	6012940	ATTGTGGCACTAGTGGAGGG	(TA)11	(TA)9	TCCATGTATGGTTTGTATGGTCA	59.99	182.00
Ca-II-SNP1447	107.65	Ca-*desi*-LG(Chr)8	6026460	TTGACATTAATTACATTTGTGGTCCCAACTAATTGTGGTCTCCCTTTATACCTTATGGTGGTCAGATGAGGTAGAAATTTTTTAGATCATTAGTGATGGA	A	T	GGAATTGCGAATGAGAAGGTTGGAATCTGAAGTAGCATCTAAATCTAAACCCTTGGAATCGGGTGGAATACCTCTTGTTCGAAGTGAGCGTCTGATGACTACAGGGGTTTCCGTTTTAGGTTTTTTCTCCGATTTCACACTG	NA	NA
Ca-II-SNP1448	108.54	Ca-*desi*-LG(Chr)8	6094120	TAGCTTGAGTTTCAAGAATGATGATTGTGTCCTCGTTTACTTGGGCCTATGGCTTAAGTTCTGCACAAAGTTCATGTCAGTGTAGAGTTTGCGGGTCGAG	A	G	CCAATGTGAACATTAAAAAAGGAAGTAGTGAAAAAGTTCTATTGTTGATCAGAGATTTCTCTATGAAAAATGAATCGGGTTTTGAAAAAGAGGAAGCAGT	NA	NA
Ca-II-SNP1449	109.43	Ca-*desi*-LG(Chr)8	6234529	ACACTTGAAGATACATGAGCAGAACTATCTGATTCGTTATTTAGAATTTAGAGTAGTAGTTTTCTCTCTCTCGAGATTTGGAGTCACTATTACTCCAAAT	G	C	AATGCAGTACAATCTGATAAACTACAAGCATAATCAATACTACCAGGCAAATAATGCAAATCAGTAACATCAGGATCCAATATACACCATTGTTTTCCCAAATATTTCACACCTTCAACAGCTACAAGACCTTTATTATCACT	NA	NA
Ca-II-SNP1450	110.32	Ca-*desi*-LG(Chr)8	6328459	ATGTCCCTCATCATCGTCTTTAATTTCTCCTTGAAGCTGTCGGTCGAATCCTCTCTAGGGGATTCATCGCGAATGATGTAGAAGTATCCGTGTCATGTGT	A	T	TTTTAATTAATTATAATTTTATAATTAATAGTGATTTATTTCTTTTAATTTTTAATTTTTCAGTGGACGCACGAGGAAGAGACAGCAAAATCATACGAAT	NA	NA
Ca-II-SSR428	111.21	Ca-*desi*-LG(Chr)8	6423497	ACAGCTAAGCAATGAATGCG	(AG)11	(AG)12	CTTCCTCCGGTCCATTGTAA	59.09	167.00
Ca-II-SNP1451	112.99	Ca-*desi*-LG(Chr)8	6440897	AGTATCTTTGTTAAGAAGCTCTCTTTGTGTGGCTTTCAATTCACTCAAAACATGCGAAAGCTGATCCCGAACTTTCACAGTTTCTAATTTAAGATCAGTC	T	A	ACTTTGTTGAAAAACTCTGATATTGAAAGAAACAAATATGTGAACGAATGTTCGGAGGGTAAAGCTAGCAAAGACGAGCTTGAATCAATGACAAAAGAGA	NA	NA

Marker IDs	Genetic positions (cM)	Chromosomes	Physical positions (bp)	Flanking sequences/Forward primers	ICC 12968	ICC4958	Flanking sequences/Reverse primers	Annealing temperature (0C)	Amplified Product size (bp)
Ca-II-SNP1452	113.88	Ca-*desi*-LG(Chr)8	6469017	ATTTCCTACGAAAAAATGTGTTTTTTTCCTTAGTCTATTGTGGGTGTTCTTTGTTTTTTGGGATCTAGCTTTCTCATCATATAATGTTCAATTTTGCTAG	T	C	CAGTTACACCCCAAAAAAATAATCTTTTGAATGTGAATTAGTATTTTTTTAACTAAGTTCGGGTAGGTTTCTTGAATTAAAATAAAATAAAACGAGTCTT	NA	NA
Ca-II-SNP1453	114.77	Ca-*desi*-LG(Chr)8	6491984	CCATTTTGTTTCTCCCTGTTTTGGTCTCCCAAACAGAATTTTGGTCCAAAACTTGATGAAATTTCATTTTTTTTGCATTTATTTAAGTCACACAATGCCT	T	C	AATTTTAGTCCAAAACTTGATGAAATTTTATTTTTTTTAAACTTGTACTACACCATTTATGGTCATATATTTCGGGTACAATTGTTGCAAATGAGATCTT	NA	NA
Ca-II-SSR429	115.66	Ca-*desi*-LG(Chr)8	6529039	GCTCGTATATTTGTTTGATTTTGG	(AT)7	(AT)11	AATAAGTTGCAAATCTTCATAGGATAA	59.02	142.00
Ca-II-SNP1454	117.44	Ca-*desi*-LG(Chr)8	6641248	GGAAGAAGCCCTTACCTTAATGTTAGCTTCAACGTGGTTTTCCGATACCGCGAGTAAAGCCGGTAAAATCTTTGCTCGCAACGTCGCCTCCAAATTGGTC	C	T	TGAGATTTAAACGCAAACCCCTCCGTTTCTTCTAGATCCAAGCTTCATTTCGCGAAGAGTTAGAAGGGAAAGTTGCTCACTACCACGCCACGGTGCTAGG	NA	NA
Ca-II-SNP1455	119.22	Ca-*desi*-LG(Chr)8	6673883	TCATTGGATTAATATATCTGGATTATGTCCTCTGAGAAGATGTAGTTTGCATATGATTGACGGTAATATGATATCTGCTTTTGCTGAGAGATGGCACGCA	G	T	GGTGTGAAAAACTCTCCAGTACATGAGATGCTCAAAAGACCCCCGACGTCGTCAAGAGTAATAGTCATTTCACTGAATGGCAGGTGAAATGAACTAGTCT	NA	NA
Ca-II-SNP1456	120.11	Ca-*desi*-LG(Chr)8	6674022	ATTACTCTTGACGACGTCGGGGGTCTTTTGAGCATCTCATGTACTGGAGAGTTTTTCACACCTCCTGCAAATGTCAATGAAGATTTGGCAATTGTTGCTG	T	C	AAAAAATACCTCTTTCAACCACTCAAAACTATACGAGGCCCCTCTATTGGTCCTAGTCTCAGTGACAGCTTCATCATATGCAACTCCCAACAACTCAACA	NA	NA
Ca-II-SNP1457	121.00	Ca-*desi*-LG(Chr)8	6675197	ATATGGGTGGTCCTGAAGATCAGATGTGTGTCTTATTAGAGAGAGCTTTATACATTTCTAGAGGAGGGGATTATCAGTAGTATTATAGTTGTATTATTCA	A	G	ATAATAACTCTGAATAATAAATAACTAACTAAATAAGTCTGAATAAGTCGCTAATACAATAAGTCTGAATAACAAATAACTCGCGAATAATAAAATAAGT	NA	NA
Ca-II-SNP1458	121.89	Ca-*desi*-LG(Chr)8	6716647	CACGATAAATTACAACTCAAGTTATTTCATCTCATGCTTGTTTCCTAATTCGATCAGCTTACCGACAGATTCACGAAGCCTCTTGATCCTCAACCGTTCA	T	C	TTAGCGCAACTCATATATAACTCTAAAAATATTCTCTAAATCATTTTTCTTTCAATTTATTAAGTGCATGTCAATAATGTCTAGTTTTGACGACAAATAC	NA	NA

Marker IDs	Genetic positions (cM)	Chromosomes	Physical positions (bp)	Flanking sequences/Forward primers	ICC 12968	ICC4958	Flanking sequences/Reverse primers	Annealing temperature (0C)	Amplified Product size (bp)
Ca-II-SSR430	122.78	Ca-*desi*-LG(Chr)8	6719856	AAATTGTTCTTTAAGTTCTGCACTC	(AT)11	(AT)10	CAATAATCTGTTAGGGTCTTTTGACA	57.43	124.00
Ca-II-SNP1459	124.56	Ca-*desi*-LG(Chr)8	6871710	TTGACATTAATTACATTTGTGGTCCCAACTAATTGTGGTCTCCCTTTATACCTTATGGTGGTCAGATGAGGTAGAAATTTTTTAGATCATTAGTGATGGA	C	T	AAGAGTTGTACATGAGAGGATAAGATAACCAAACATGCACTAAGGACAAAAATAATCCCGTATGGGCTAGTTCATAGGACACAAACTAATGGATCTATAT	NA	NA
Ca-II-SNP1460	125.45	Ca-*desi*-LG(Chr)8	6879863	GTCTGAGCCTTGCTTATGATTAATTTTTTAGGCTTAAGCTTGGCCTACGGTCTAATATAGGTTTTTTTTCGGCATGACCTCTTTAAAAGTCTGGTCTAGC	A	T	TGGGGGGGACTACTTTCGCGATTCAGCTAAAATAGGGGGACCAAAACTGCAATTAAACGTAAATATTTTAATATGAATAAGACTTTTAAATAGGCTTCCA	NA	NA
Ca-II-SNP1461	126.34	Ca-*desi*-LG(Chr)8	6883558	TTGCTTCTTCTTTGGTGACATCCCTCTTGTCCCCCCCGGCTGTCCAACCACCTCCTGCGATTGTTGATCCTCCTCGTTACCCCTCTCGTCATCATCTTCA	A	G	TCAACTAAAAAAAACGAAAACGTTTAATGGAATGCATCTTTGTGAGCAAATCAGCAATCTGTAAAGAAGAAGAGACAAACGGTAGAGTAATGATTCTATG	NA	NA
Ca-II-SNP1462	127.23	Ca-*desi*-LG(Chr)8	6890882	GATTCCTCATGCATGTGAATATGTGATTTTTAATTGTAAATTAGTCTTCATAACAGTGTTTTTTTACTGTTGAAAGTTGAAAACTATTTGGTTAATTACT	T	C	TTCAAAGGAAAGTTCCTTGATTCAATTCCAAAAGTATCAGCTGTAATTGTGTCAATGACTGTGACAAAATCCAAAATAATAAAAACAAATACAAATATGT	NA	NA
Ca-II-SNP1463	128.12	Ca-*desi*-LG(Chr)8	6900162	TCCACGCATCGCATGCCATGTTGTTTCTTTTTGTCTTTTTCGTTTTATTGCAACATATTTGAAAGGAACAACAGAATGTAATTTGTACGAAGCTTCTTTA	G	C	AATTATACTTTAATTATTCATGACCAAATTTTAAATTATTAGAATCATATATATAAAAGTGTGTGTGTGACCATGTCCCAAAGTAACAAGATGGATGCCA	NA	NA
Ca-II-SNP1464	129.01	Ca-*desi*-LG(Chr)8	6901022	TCAAGAGCATGTTGATTGGCTCTACTTAAATTATGGTAATCCTTTGTCTTTAAGTTTAGTTGCGATCTTTCTCTAATTTTCTATATAGGTTTGAAAAACC	T	C	CTTAAGAAACGCCATTGACATTTATTAATTTCTTTTTAGAAATATTAAAAGACACTTTTTGAAACGTCAATGAGATTATTTAATATAAAATTCAGCACAA	NA	NA
Ca-II-SNP1465	129.90	Ca-*desi*-LG(Chr)8	6906044	GACACTTTGTTTGTTGGGGTCGTACTTAAGATTTTGGGTCTCTTATGCGATTGATAAATTTATATCCATGTCTAAATTGTAGATAATTTTTAACATAAAA	G	A	TACATGTTCTAGTAAATAGATACATGTATAACTAATTTTGCTATAATTCAATTTTGATTTTGGTTTAATATGAATTTTTCAATACTTATATAATATTTAA	NA	NA

Marker IDs	Genetic positions (cM)	Chromosomes	Physical positions (bp)	Flanking sequences/Forward primers	ICC 12968	ICC4958	Flanking sequences/Reverse primers	Annealing temperature (0C)	Amplified Product size (bp)
Ca-II-SNP1466	132.49	Ca-*desi*-LG(Chr)8	6914175	TTTGGTGGTTCGTTATGCTCCCCACTAAGATAGTTACATCATCTACGAAACCCAAACTCATTTGCCCTACTTTATAGTCAATTGATTGACTCGTTATAAA	A	T	TTTTTACATTTAAAATGTATAACAATTAGACTCCACATTTCTATTAGATGTGTCAATGTTATGGAGAAAGTAAGTTTACATTTTGAAAAATAAACTCAAT	NA	NA
Ca-II-SNP1467	133.34	Ca-*desi*-LG(Chr)8	6919957	TGCAGTTTTGGTCCTCTTATTTTAGTTGAATCGTAAAAATAGTTTTTTTTTTTTTCCAGTTTTGATCTCCGAATAAACTTTTGGTCCAAAACTTGATGAA	T	G	CGTTTATTTAAGTCACATCATACCTCAAGATCTCATGACGCAACAATTATATCTGAAATATATGATTATAAATAATGTGGTGTGACTTGAAAAAATGAAA	NA	NA
Ca-II-SNP1468	134.19	Ca-*desi*-LG(Chr)8	6931424	TCCTTTTTTTTTTATTTCCCTAAATGCCCTAATTTAAAAACTATTTCTCTAAATGTCTTATTTTTTTTTAAAGCAGGAAGTCGTTCTTTGAGGAAGCGAC	C	T	CAAGAAATAATTAATGGTATCATTTGCACTTAGGATATGAAAAATTGGAGGTTTTTTTTTTATCAATATATCCCTTTTTATTTATTTATCGTCTTCAAGT	NA	NA
Ca-II-SNP1469	135.04	Ca-*desi*-LG(Chr)8	6945723	TGCGGCACCTTTCTATATTATGATTCAAGTTTAGTGTTTACGACGTACAATTTGTGTTTAAATAGTTCAGGCTAGTTTACAAGATTATGAGTCATACTTA	C	T	AAAATAATAATATCTTTTCAAAAATATATTAAAATGTTCAAAAAGTTAGAAGAACAAACACGTACAAATTTTTATCTTTAAAGTATGACTAAGGATTGTC	NA	NA
Ca-II-SNP1470	135.89	Ca-*desi*-LG(Chr)8	6954083	ATACGCTGCGTGGCAACATAATCAATACACAGTGGATGCTTTTATACACTGTGTGGCCTCATTTGACACAACCATTATACAATTTCTACACTTCCTTGTA	T	A	CACAATGAGGTTTAATCATATGCAATCTTAACAATGAATAGGCGAAAATACTAAGAAATAAACTACTTTTATTTTAAAGAGTTTACAATTGTTTCAAAGT	NA	NA
Ca-II-SNP1471	136.74	Ca-*desi*-LG(Chr)8	6955563	ATATGAGCCATACTTAATATCTCAGATATAGTTTAAAAAATTCATTGTGTACATATTTGTTTTCCCTAATTTTTCATTTTCCTAACATGTTTTGTTTATG	G	T	AATGCTTGGAATAATACTCTTTTTTCTATGCTCTTAAATTATGCTATAGATTCAAAAGGATGAAATCTATCTCTAGAAACTATAAAAAAAAATCCTGTTGG	NA	NA
Ca-II-SNP1472	137.59	Ca-*desi*-LG(Chr)8	6957824	AATCATCACTGTTTTGGGAATTCCAGTGACCAACACTGATCAAAATCAAAACTTGTTGAGTTGGGGTCTGTGGGCTATATCTATGAATGTTGATTTATGA	G	C	TTTTGTTTCTTGTTTCTTGTTCCCTTTACTATAACTAAGCAAATTATGTTTTGTCTTCTTTTTTCCTCAACGAAACTACTTTAATAAATTTCCTACTTTC	NA	NA
Ca-II-SNP1473	138.44	Ca-*desi*-LG(Chr)8	6960219	GAGTTACTTATGATAGGGGATTTGAAATGGCTATTTGAAGATCCAGTTCATTTTTTACCTTTACAGTTTATTTTTTTTAACTAAAAGCTATTTGCGCATA	T	C	TCTGTTATCCGATACAGTACCTTTAGAAAAACATTTATCGAAAGAGCTCAATCATATATAAATAAATTACAATTAGTTCAAAAGATTAATCTCTATTATT	NA	NA

Marker IDs	Genetic positions (cM)	Chromosomes	Physical positions (bp)	Flanking sequences/Forward primers	ICC 12968	ICC4958	Flanking sequences/Reverse primers	Annealing temperature (0C)	Amplified Product size (bp)
Ca-II-SNP1474	139.29	Ca-*desi*-LG(Chr)8	6961782	AGAACACTTGTCTGAGTTGTCAGTGTTATACGAGTGTCGTACAAGTATCAAACGCTGACACGTGTTAGACGGGAAACAGCCTAATCTCGGAGATGTCTGA	G	A	CTGTTTATCTCTCCCAACAGGTTAGTTTTTTTCAGTTTGATTGAACCCCTTTAACCTGTTTGATGAATTGCCTGATATGACACATTAAATCTAATGGCTG	NA	NA
Ca-II-SNP1475	140.14	Ca-*desi*-LG(Chr)8	6964897	TCATCATGGAGTTCAACACACTCCCAAAAAAAGGATTTTAGAGCAGGAAGGCTGAGTTGTATCCAGAATCACTGATTGTCTTGCTCGTGGTCACCAGGTC	C	T	CAATGGCCACGCACCTCGTGGTGTTGCAAATAGGAATGCATTAGCAGTCTTGTTTGAGTCGTTGCTACCGTGGGTAACTTACGAGGATAGGGAGGATGGT	NA	NA
Ca-II-SNP1476	140.99	Ca-*desi*-LG(Chr)8	6968877	TTTAGAACCTGGTGTTCGGGTTCAGAATCGTGGTCGTTGAGGAGATCGAAGGGGTTGATTGAGGAAGGTGCAGTTGAACCTTCTTCTTCCTCATCATTTT	C	T	AACTCACAAAACGATGTCGGGTCGATTGGTGAAGAAGATTCTGAACAAACAACAACACTTTCCTCATCAACTCATCAAAGAAGAAGAAGAAGAAGATGAT	NA	NA
Ca-II-SNP1477	141.84	Ca-*desi*-LG(Chr)8	6971496	ACACTTGAAGATACATGAGCAGAACTATCTGATTCGTTATTTAGAATTTAGAGTAGTAGTTTTCTCTCTCTCGAGATTTGGAGTCACTATTACTCCAAAT	T	C	ATAATTTAGGTATTCCATTCATCTATATTTCCTCACATTCAAATCCTTTTGATTGAGCATGCAATGTAAACTTTTATGATAGTTGAATACTTCAAATTGT	NA	NA
Ca-II-SNP1478	145.24	Ca-*desi*-LG(Chr)8	6972137	TCAGATTTAAATATGCCTTTGCTCCTTAAAAATATATCACTTTTTGATGTTTGTCATCCTAAGTTTTTTTGTTTGAATTTAGTCACTAAAATAAGAGACC	A	G	TTAGGGTTTTTTAGAAAATTCGCCTTCTTCCGTGCCGGTGCAGTTTGCTAACTCTCTTTTTGTATGTAGCTTTTGATATGATATTAAGGACAAAAACCAA	NA	NA
Ca-II-SNP1479	146.09	Ca-*desi*-LG(Chr)8	6978739	CGCACATTGTGCACCGCGATATAAAGTCGAGCAACATACTCCTTAACGAGAAGTTTGAAGCACGTGTTGCCGATTTCGGGTTGTCGCGGTTGATTCTTCC	A	G	TGTACACATCTCCTCTCAGAGTAGCCACCCATGCTTGTCCGTACTCCGGAGGAATGTAACCTAATGTACCTACAAGTTCTGTTGTAACATGAGTTTGATA	NA	NA
Ca-II-SNP1480	146.94	Ca-*desi*-LG(Chr)8	6979516	ATATCTTGTTAAGAATACAGCATTTTATCAATTCTTCTTGTTTTACTTCTCAATCTTAATACAGCAATGCTACAACTTACTGACTTTTATTATTGCCATT	C	T	GAGTCTGAGCTATAAACACAAAATTTGACATGTCTTTTCCTTTTGAAATTAAGCTATACATACTAACTATAATTAAAAAGGGATTCACATTTAGTAATGT	NA	NA
Ca-II-SNP1481	147.79	Ca-*desi*-LG(Chr)8	6982315	GTCAGATGGCATATCTACGGATCCTTGACAATCACCTCCATTCTCTCCGGAAACAAACTTGGAACATAATTCTAATTTTTTTTGTTCAAATTTATCCTGC	G	A	TATTTATTTAGCCCTTGTTCTTTTGTCGCATCTTTAATTGAATTGGCTCTACCTTTACCTTTTCTAACAGTTATTTATTTTCTGATATATTCAAATGTGA	NA	NA

Marker IDs	Genetic positions (cM)	Chromosomes	Physical positions (bp)	Flanking sequences/Forward primers	ICC 12968	ICC4958	Flanking sequences/Reverse primers	Annealing temperature (0C)	Amplified Product size (bp)
Ca-II-SNP1482	148.64	Ca-*desi*-LG(Chr)8	6984120	TCACAATTTCTTTTGTTTATTTCAGACTTTCTTTTGTACTTTAAGGACTCCCAATGTGCCTTCAGTTGAGCCCATACATCCTCTTCCATCCAAATTGGTT	G	T	GCCACCTCAGCATAAAGACATGGTGCGGCGTAACTTTGAGAAAAGGGGTTCAGCTAAAATGATTCAATTAATGCAAGAAGCACGGAAAAATTTAGATCAA	NA	NA
Ca-II-SNP1483	149.49	Ca-*desi*-LG(Chr)8	6990174	TTAGTTGGGGGATATATGGTGGATCTATAATTTTTTTGATTTTGTTGGAAATTATTTTATGGAGTTTTGTGGGATATTGAAGAGTTTGATGGTTAGAATC	A	G	ACACAAAATATGGTAAGTTAGTTATGCATTTTACTAAATTCACACTATAAAATTGTAATAATTTACAGACATTTTTTTAATGTTGTGTAAAAAGATGCAA	NA	NA
Ca-II-SNP1484	150.34	Ca-*desi*-LG(Chr)8	6993303	AGGTATCAGAATTATGTTTTTGAGAAGTCATCAAAGTATTTACTAGATACTATCAAACCTGTGAGTCGTCTACCTTCCCTTTGGATTTTTGTGGATGGTT	G	A	TTGTAAACTGGATAGCAAACTATACACATGAAATATAAACAAATTGTTGGCAAATCTATCATGGATTATTTTTTCCCCACGTTCATATATAAGGGTGAAA	NA	NA
Ca-II-SNP1485	151.19	Ca-*desi*-LG(Chr)8	7004534	TACCTCGTGTTCCTATGAATACCATGCAGTATTCTGCTCCTAACAACAGTGGCCTTCCGATGCCTCCACCAAGACCACCAGGCATGCACATGATGCCATC	T	C	TCATATGATGCATTGATGGATGCATTGGAGGTGGTTGACCAGCCATCATTGAATGCTGTCCAGAGGACATTTGCATTGGAGGAGGGGGCGCTGGCCGAAC	NA	NA
Ca-II-SNP1486	152.04	Ca-*desi*-LG(Chr)8	7009241	TCTCAAACTTTCCCATAAATGACTCTCCTTGTATTGACGAAGATACAAATTGTTTTCTTTTAATTTTGATTCAGACTTTGTACGTAAGAATCTACTATTG	C	G	AAAAACTTATTAGTTAATTTTTTTTTACAGTTAATTATATGTAAGTTATATACTTATTTTTTAATATACATTTATTAGTAAGCTAATTAACAAATGAGGGG	NA	NA
Ca-II-SNP1487	152.89	Ca-*desi*-LG(Chr)8	7012940	TTACTTGTTTCAGGGATCTCTCTTTCAATTCTTTAACAGGAGACCTCCCTCAAACTATGAACTCACTTTCAAGCATAACCACCATGTAAATGTCCAACCT	T	C	AAGGACATTAATGGTTCCTGTAAGTTGGTTGTTTTGTAGATACCTACGCACACGTAAACAAGTCAACTTAGAATGCAATCAATACCAAATAATTAAACAC	NA	NA
Ca-II-SNP1488	153.74	Ca-*desi*-LG(Chr)8	7023172	CAATTATTTTCATTGGTACGATATCTTTCATTTATTTATTAGAGAACATTTTTTCGTGATAAATTTGATTTATTTGAATTTCGTTCATATGATTTTTCAT	T	C	TAAGTTAGTATATACAGTCTCCAACGATTATCTACAACATACCTATCAATAATATTAGAAAAAGACAGAAATATAAAAATATGAGAGACTAAATCAAACT	NA	NA
Ca-II-SNP1489	154.59	Ca-*desi*-LG(Chr)8	7024894	AATATCTCAATTCAGACTAGACGAGAATATGTATACTTACCCAATGTGGTTCTTGAGAACTAAGGCTTTCATTTTTAGAATCTTTATGCTCAGTTTCCTT	G	C	TAAGAATCTGCTCGGTTTTATCTTCAATTTCTCAGGGAAGTGAGAAGCTGCTTCAACTAGTCTCAGATAGTTGCAAGTCTTCCAACACTTATAAGGATAA	NA	NA

Marker IDs	Genetic positions (cM)	Chromosomes	Physical positions (bp)	Flanking sequences/Forward primers	ICC 12968	ICC4958	Flanking sequences/Reverse primers	Annealing temperature (0C)	Amplified Product size (bp)
Ca-II-SNP1490	155.44	Ca-*desi*-LG(Chr)8	7029788	TTCAAAACTCCTAACCTTGGGTTAGATCTTTTACTTTTTGAGATGAATAGTATAATCGGACATCTTATCAATTTTTTGTGATGCACATTATCATATGATT	G	A	ATTCTAAAAAAAAAAGAATACTAAAACAAATCATTTACAATGCAATTACAAAAATGATTTTAGATAAGAAGATGAATTATATATGTTTATAACAAGTGGT	NA	NA
Ca-II-SNP1491	156.29	Ca-*desi*-LG(Chr)8	7036172	TATGTTTGGTATCGAACCTCTGCGAACTACAAGTAATATTTGTCTAAAATCACCATCTATTACAACTGGAGTTCCACCAAATGGTTTGTGTTTGTTGTCT	T	A	TCATGATGACTTAGTCCTTAATGTGGCCTTAAGTGGTATTACATCATTGTTACTACCAGGCGGTCATACAACCCACTCTACATTAAATATTCTGTTGGAG	NA	NA
Ca-II-SNP1492	157.14	Ca-*desi*-LG(Chr)8	7037315	TCCATAATTTCAACGCTATTGCAATGAAGCACCAATTAACCTTTTGCGTGTTAACTTCGTCCATTCTATCGTATTTGGATTCATTATGTTGAAATATTAC	T	C	TATTTTATAACTTTATTCTATTTAGTGTGTTATTGATTCTATTGTTCGGTAAAAAGGTCCTACATATTATGTTATCTTTAACACCACTACACGTTTAGTT	NA	NA
Ca-II-SNP1493	157.99	Ca-*desi*-LG(Chr)8	7043758	TACACCAGAATTTGTGGATACATTTGGTACATTTTACTCTTTAGTATGTGCAAGACATTTTTGGACTTCTTTGTTGTCAAAACCTAACTCTTACAAGATA	G	T	TTGTTAGATAAAAAAATGCCTTACAAGTTATCATTTAACATCAATCTTATAAAAAATCTGGTTTGATATGTTTGCCTTTGTGTGTTTATTTGTACTATTT	NA	NA
Ca-II-SNP1494	158.84	Ca-*desi*-LG(Chr)8	7044735	TTGGTTTCTCATTGGCTGTTAACATTAATTTGTAACTTGTTCGTAAACTTTAGATCGAAATTGAATTGAATTTTATTATTTGCATATTGACCGCTGTTTG	C	T	TTTTATTCCAACATAAAAGTTGTGACTTTGATTTTTAGCTTTCCAACGCCTGCTTGCATGCGTCAATATGATATGCAGAACTCAAGTTATGACCTACAGA	NA	NA
Ca-II-SNP1495	159.69	Ca-*desi*-LG(Chr)8	7045300	GGAATGCTTTATGCTATTTTATTTTGATTAATTTGCTGCCATATATATGTAGGTTTATATTCGCCGTATATTACTTGTTATTGTGTTTTTGTGTTTATTC	C	T	CAAGTTTAATTTTTAATTCGTGACTCTATTCCTATTTGACTATAGAATTGATCAAACAAGTGTAATTAATCCCATGTGAATTCGGTTTCTTTGACTGGAG	NA	NA
Ca-II-SNP1496	162.24	Ca-*desi*-LG(Chr)8	7047467	ATTAGAAATAAACTGTGGACTATAAGTGGGTGTTCAATGTGATTGCCCCTACACCCACTGAATATGAAAAATGCTTTCTTCATGTATGATCCCCCCAGCT	T	A	CTTTACTGGCTAGGTGAATTTTCAAACCATTCCCTGAAAGACTATTTCAAGCCACAGAGAGACTTCTCCAATTTGCAAATCTGACCCAAAATTATCTTCA	NA	NA
Ca-II-SNP1497	163.09	Ca-*desi*-LG(Chr)8	7049649	CGTATTTGATTAGTGCTCGATGTTTTGTAAAAAGACCCAAAATATATCATTCAATGCCTTGCTTGCATTTATTTAGCGTTTTAAGCCAAAATATTAGTTG	C	T	GTCTGCCATCACCATTGGCTTTTGTTTTCCTTTGTTAATGCACCTTGATCCATGAATGACTTTGATTCTATTTCAGGCTCCTCTTCTTCATCAGATATGG	NA	NA

Marker IDs	Genetic positions (cM)	Chromosomes	Physical positions (bp)	Flanking sequences/Forward primers	ICC 12968	ICC4958	Flanking sequences/Reverse primers	Annealing temperature (0C)	Amplified Product size (bp)
Ca-II-SNP1498	163.94	Ca-*desi*-LG(Chr)8	7061028	GGCGAGATCATATTTTCCAGCTCAGATTTTGACTTTGGTCATGCGCAACCTGGTTGCATTTAAAGGGAAAATACCCTATTTTCTAAATATATTCTTCATC	A	G	AATATCAACACTCATCTTCATTAATCTCATCGCCTCCTCCATCATTTCCATTATCTCCTCCATCATCATCGTGAGAGGTAGAGAGGATGGTTGTGAGTCG	NA	NA
Ca-II-SNP1499	164.79	Ca-*desi*-LG(Chr)8	7062955	TTATGATGAGGGTATGGAATTAGCCCCGCACAAACTCCTAAGATAGTGAAAGGCTCAATCTCAATATGAGTAGTTTTTGGTGTCGCATCTCCTTCATATA	G	A	ATTTCTTGTTTATTTTGTTTTTGAAATAATATTCGTGACAACCAGTAATCTCAGTTGCCTCACCAGTAATCTCAATTGCCTATTTTCTTTATAGATTGCT	NA	NA
Ca-II-SNP1500	165.64	Ca-*desi*-LG(Chr)8	7066214	ACAACAATCCATTTCAATTAGCCGTGTGTGATCTATGGGGCCCTACACCAGTTAAGTCCTCTGGTGGCTATATGTATTTTCTCACCTGTGTAGATGCTTA	C	T	ATTTTACAACTGAATTGAAGTTCTACCATGGCTTTGAATTGTGTAAATTGTGCTAGAGTATCAGACTCCAACTTTAAAGGATAAACCAAACAAACCTAGA	NA	NA
Ca-II-SNP1501	166.49	Ca-*desi*-LG(Chr)8	7067157	TTCTGACCCAAACTCTGCATCCAGACCTGAAGCACGTGAGTCCAAACTGGACGCATCTGTCTCCAGACCTGAGGCATCTGCACCCAATCCTGTTCCCATC	G	A	TTCATGCTGCGAGTTAGGAGCAAAATGGGTTGCAAACGGGGTAGTATAATGCCATCCTTAGCTCTAGTCCCCATTGGGTGAATGTTCCTAGGATGTATCC	NA	NA
Ca-II-SNP1502	167.34	Ca-*desi*-LG(Chr)8	7068247	TCTCACTTGTGAATCGGTTTGGAGTCAAGTAAAGGCACTCTATACTAACGACACACAACGTCTCTATGGAGTTTGTCACCGCTTGTTGAATATCATTACT	A	C	ACTGGATTACTTGCAGCAAGAGGGAGAAACTTATTAAAGTAATGAAGTGCGCTATGAACGATGCCAAGATATGCAGAAATAGAGCCATCAAGTGTCTTCG	NA	NA
Ca-II-SNP1503	168.19	Ca-*desi*-LG(Chr)8	7677137	GGGGTGAAGATATTGATATGATATGTATACAATGTATTAAGTTTTTAACTCAGTTGTTTGGTGTAGAGAAGGCACCCTACAGCTCTCCCAGCACCCATGG	A	C	AATTTTTTTATATATAATGAAAATAAACAACATGGTGACTAGGTAGCTTATCATTTTACTCCCTAGGCCAAGCCAGGTTCAGTTTTCTAAAGCACTTGAA	NA	NA
Ca-II-SNP1504	169.04	Ca-*desi*-LG(Chr)8	7749615	CTCAGTTCGGAACAACGCTCTCAACGTCTAGAAGTACAATGAGACTTGGTGAATGTGTCTGGCTGTTGATCATGTGCAGGATTGGGATAGACAGGGAGAT	A	G	TTTACAGTGGCACCTATTTCTTCTGTGCTGATCTCATGCCGTACACTCACACTAAACATAAGCTGGACTTGTTTTTGTCAGGAAGAAATTTATCAACAAG	NA	NA
Ca-II-SNP1505	171.59	Ca-*desi*-LG(Chr)8	7770919	AATTTTGATTACTTGCAATTTCTTCACGGAAAAGCATTTTTTTATTCGTTCATAACTTTGTAATAACTCTTTTATATTGTATGCAAGATTTAATTTATAT	A	C	ACCCTACAAAAACAACAGCATCACACATATCACATTTTTAAACATCAGATTAAACATTTTTAAATCAGAAGGTTGTGATGTGATTCACACTATCAGTGTA	NA	NA

Marker IDs	Genetic positions (cM)	Chromosomes	Physical positions (bp)	Flanking sequences/Forward primers	ICC 12968	ICC4958	Flanking sequences/Reverse primers	Annealing temperature (0C)	Amplified Product size (bp)
Ca-II-SNP1506	172.44	Ca-*desi*-LG(Chr)8	7879595	GACACGGTTTCGTGAGTTCCTAATTCTCTGGTTGGATTGATGGTTTGGATTTGCAAAAAATGAATAGAATGGTATGAAACATGATTCCATTCCCTACCAC	C	T	ATAAAACAATAAAATAGGGGTAAAGAAATAAATATTTAATTAAGTTGCTCCATTCTATTGGATACATTCCAAATTGGAGGGAAGAAAAATAGGAGGAGAG	NA	NA
Ca-II-SNP1507	173.29	Ca-*desi*-LG(Chr)8	7943790	TGGTGGCTTTTTGAGTTAGAATCCCTTCACCGTTAAAAAACTACCCAAGTTTGGCAGCACCATCTCGGTTCTCTGGTTTTTGCTATGTGTCTTTCGCTTC	A	G	CTAAATCAAGGGAGCGTTATGGAAAACAAAGACAGTGTACATTGTCACTAATCTGAAAAAATGCATAGCTATGTGGAAAGCAACGAATGACTATGCAGTG	NA	NA
Ca-II-SNP1508	174.14	Ca-*desi*-LG(Chr)8	7944361	TTGTCCTGAACTCCTCTAAATTGTCAATAGAACAAATATTTCTTTGCCAATACTATTATGGTATGCCATTTTTAATTGTCATGGATGCAAAGCTTTTGCT	T	A	TTAAATAAAATTCAAGCAAAAGCTACAAAAAATAAGCATCATAAATAACATCACTAAATTAACAAAGCAAGAAGCAACCCTACAGAAACCATTTCCACAC	NA	NA
Ca-II-SNP1509	174.99	Ca-*desi*-LG(Chr)8	7945632	AACTTGTTTTAATTTTAGCACTTCAGAGTGGCTGTAAATTTATGTTTTAACTTTACAAAATTAATCAAATGTATTGCAACTGCTGAGTGCAATGGTTCTT	G	T	TCCCATCGCTGGTGAAAGTTGCATCGTTCACATAAGATGTATGGCCACGAAACTCTTTCAACATCTTTCCAGATTTGAGGCCATGGATTCTATATTACAT	NA	NA
Ca-II-SNP1510	175.84	Ca-*desi*-LG(Chr)8	8060043	GCAAGTTCATTATTAGGGTTCCTACAAAGGATTAGAATAATGACACATTCAATACTTGAATTCTCGGTGTAGATGCTACGTAGTTGGTTCTCATCCTTCA	A	G	GTTCCTACAAAACTAGACTCGAAAAGCTTGAGAAAGAAAATATAACCATCTTAAGTTGATCCTTTTTTTGACGAATGCTCCTTTATTATACTTGTAGTCT	NA	NA
Ca-II-SNP1511	176.69	Ca-*desi*-LG(Chr)8	8086999	AAAGGCGCTTTAATGCCATCATTGCAACATACTTACCTGGATGGAGTCAATGGCTGATCAGAAAGGCCCATGGCTTAAGTTTGTGACCTCCATTGCACTT	T	A	AGACTTGACTAAGAAGGGGCCGCGCGAACGCAGGCCCCAACAATAACAAATTATGATGTAGGCTCCACCACTTGGGTAGACCTTAGGTGAGCACCCCTCC	NA	NA
Ca-II-SNP1512	179.18	Ca-*desi*-LG(Chr)8	8111791	ATTTTCTTGGTGCTTTTTTCCTTCTCCGTGATTATATTAATATTTCCTCTGTCCCTTATTATAAAATCTACTTAGAAAATTCACAGGTACTAGAAAAGTA	G	C	ATTTAATTTACCGTTGGAAAACGTAAATTCTCATTATATCAAAAATTGTAAAGGTAATTATGGAATAGTAGGACATATTTTGACAAATTTAATGCTACAA	NA	NA
Ca-II-SNP1513	180.01	Ca-*desi*-LG(Chr)8	8140661	AAGAAGTTAATATTAGCATTAGTTGAACTTTCTACACTTGTTGTTGGACTTGTGAGACTTCACATTGAAGACCACACATATTGTGTTGGCGCTAACATGG	A	G	AGTTGAATGTGAGAATGTCCAAAATGCAACGAGCCCGGACAACATTTGTGAGCACGAAATTACAACAGGAGCTCTTATGTCACATAGGGCCTCTCTTCCT	NA	NA

Marker IDs	Genetic positions (cM)	Chromosomes	Physical positions (bp)	Flanking sequences/Forward primers	ICC 12968	ICC4958	Flanking sequences/Reverse primers	Annealing temperature (0C)	Amplified Product size (bp)
Ca-II-SNP1514	180.84	Ca-*desi*-LG(Chr)8	8141174	TAGTGGTGTGTTATCTTAAAAGTTTTTGTTAGAAGTTTGTAGCAGAAATCCTATGGTGGGATTTGTACATTTTCTGACAATAGTAAAAAAATCTCTTGTA	A	G	GGACAGTGTAGGACAATGAGTAAATGACAGTAAATTAATGAACACATAATATTATCCTGGTTTCTTTTGTGACAAATAGTAAATCCAGTCCTTTTGCACA	NA	NA
Ca-II-SNP1515	181.67	Ca-*desi*-LG(Chr)8	8376853	ACTCATACACTTGTGGATTTTTTGCACAATAGCTCACTTGAACTCTCCCCAATTGATCTATAAATCACTTATTGTTGTTAACATTACTTTCAACAAGCTA	C	T	TAGCTTTTATTTTGTTTGTCTCAATTTATGTTTTGTTAGTTACAGTTAGTTATGGTTCTAACTAATTGGAGGGAATGGATAACTATATAAGGCATTGCTT	NA	NA
Ca-II-SNP1516	182.50	Ca-*desi*-LG(Chr)8	8449855	ATTAGTTTTTTAGCCTCTATAACGGTTACTTTATATTTTTCTTATTTTTTACTTCATTCCCTTCAAAAAAAAAACTTCAGTCCTGCATTTGAAACCTACG	C	T	AAAATCAAAGAGAGAAGGCTTGAACTTTGACACTCATAGAATATTGTTCGAAAAGAAATTTTTGACAGTTGGTTATTGTATTATGAAAGAAAATAAACTG	NA	NA
Ca-II-SNP1517	183.33	Ca-*desi*-LG(Chr)8	8522269	TTGCATGGTGATTTAAGAGATATTAGATTTGATAAAACCTTGAATTCTTTCTCTTTGAATGTGGTGTTTATGTTGGAAGGTATTCTAGGATGCATTGAGG	G	A	TCTAAGCCTCACTCTCAACAATGTTTCATCATCATCTTCTTCTTCTTCTCCTTCTCCACTTTTTTCCACTTACAACAAAGTCACATCACAAACAAATGAA	NA	NA
Ca-II-SNP1518	184.16	Ca-*desi*-LG(Chr)8	8579338	GTCAAACATAATTTTTTAATTTTTACGCTTAAATATACAATTGGTCCTTACAATTACTCTATTTTTATTTTTCCACAGTTTTTTTATTTTTGTCTTTGTA	G	A	AAGATTAAAATTAAATATTATATTTATTGCAGTGATTTGTTCCAATTAAAACTAATTTATATAGACTAAAACAAAAAGAGCAAGGATAAAAACAAAAAAT	NA	NA
Ca-II-SNP1519	184.99	Ca-*desi*-LG(Chr)8	8645587	GCATATCCTAAGAAGCTCTTGGAGCATATGCTATTTTCTTGATCAGCTCATTATAAATTCATCTATATAGAGAGAGGAGCTATCTTGCTTAGTATTAGTA	C	T	TGCCAAAGGAAAGAAGTCCCGCAGCTGGTTTGCTTAGCATCAAGTGAATCACAATATCTCACCGACTCTTGTGGATAATTTGTTCTGTTCAACTTTCTTG	NA	NA
Ca-II-SNP1520	185.82	Ca-*desi*-LG(Chr)8	8779980	AGTCTTTTAAGTTTTGTTGTTTCATGTTAGTCTATTAGGTTCCAAAACTTTAGTATCTTGAGTTACAAATATTATACACCTCCTTTTTGTGTCTAACATT	G	A	GTTATAAACAGAACATTTTAATCTCTTAAGTTACAAAAAATTAAACAAACATTTTAGTGTCTTAAGTTACAAAAATTAAACATTTTGATCCTTTAACTTA	NA	NA
Ca-II-SNP1521	186.65	Ca-*desi*-LG(Chr)8	8784040	TGATGAAAAAGGGTATATTATGCGCTTGTACATGTTTGAGCAATCATGTGTTGATACTAAAGTGTTTGTTGATTATGTGAAAGAAACTTGTTGACTCCAC	A	T	ATTCAACAATATATAATAAAATTACATTACATATATGTTAGTCGTTGTGTTCCCCAAATGCATCACTTGATTTGTCCATGCTTCAACAAATCTTTCCTTA	NA	NA

Marker IDs	Genetic positions (cM)	Chromosomes	Physical positions (bp)	Flanking sequences/Forward primers	ICC 12968	ICC4958	Flanking sequences/Reverse primers	Annealing temperature (0C)	Amplified Product size (bp)
Ca-II-SNP1522	187.48	Ca-*desi*-LG(Chr)8	8912416	TAGCTGTTTGTTGTCGTAACAACAGACGTCTGGGAGGTAGTCATAAATCACCACGGGTGCAGCACTACCCCATGCATATTTATCTATACCGTCTAAATAT	T	C	GATTGTGCTTCTTGAGCATATCTGTTGAATTTAGTAGGGAGTACTATTTTCGCGGATAAGAGTCATACTCTAGTTGATGCGAAATACCTTCCTTTATTCA	NA	NA
Ca-II-SNP1523	188.31	Ca-*desi*-LG(Chr)8	8963019	GGTGTTTGCATCTGTAGCATTTTTTCCAACCGTACTTTTGCCATCTACACCGTCTATGTGGGTGGCTGGAATGACATTTGGTTATGGATTTGGGTTCTTG	C	G	TGTGAGATTGAAGAAAAGGCATATACTTCAATTTTATGATGGAAGATTGAACCAATTATAAAAGGGAGTGATACACCCACAGCTGCTGCAGATATTATAA	NA	NA
Ca-II-SNP1524	189.14	Ca-*desi*-LG(Chr)8	9007562	AATTTTGATTACTTGCAATTTCTTCACGGAAAAGCATTTTTTTATTCGTTCATAACTTTGTAATAACTCTTTTATATTGTATGCAAGATTTAATTTATAT	T	C	AAACAAGGCGGGTCAGCTCCACCTCCGACTGCCGGGGCCCCACCGGGTAAGAAGCTTCTTGACATCTCCAAATTTATTTTCCCTTTGTTAACTTAGAACTTTAATTCCACAATTAGAAATGCATTCACATTATGTACCCAAAAAAAACTGCATCCAC	NA	NA
Ca-II-SNP1525	189.97	Ca-*desi*-LG(Chr)8	9031119	GACACGGTTTCGTGAGTTCCTAATTCTCTGGTTGGATTGATGGTTTGGATTTGCAAAAAATGAATAGAATGGTATGAAACATGATTCCATTCCCTACCAC	T	G	CCAGCCTATCTTTAGCTTAAGAGGGAAGAGAGAACTTCTAATAAAATAAAATAATTATAGAAAGAAGGGACCGGAATACGCTAATATCCTAAACTAATTTGTCTTATTTTATTAGAAAAAGTATGACACGCATATGCACTACCAATACTAAAACACTCATCCTCTCCTAACCAGTACTCTCTGAAAAGAAAATAACATTATCCACATATACATCTTGGGCTGAGCTGGCCCA[illegible]	NA	NA
Ca-II-SNP1526	190.80	Ca-*desi*-LG(Chr)8	9879668	GCCTTTTCTATCAAAATAGTCTCTCTCTCTTCTCTACGGTAGATCCCAGGATGATAATTCTCAATCATCTCTTCACAACGATCAATCAAAATCCCCCAAT	T	A	CCTTTCCACAAGCCATTAGGGTTTGACGCTCTAACCAACCGAGCTAAAAAGAACAATGGGTGGTAAAGTTTACTTTGAAAATCATTATTATTTGCATGGT	NA	NA
Ca-II-SSR431	191.63	Ca-*desi*-LG(Chr)8	9887641	TACCCGGATTTGCTGAACAT	(TAA)18	(TAA)20	TTACGGATCCGGATTTTATGA	60.33	205.00
Ca-II-SSR432	192.46	Ca-*desi*-LG(Chr)8	9906628	GCAATGTGTCGGATGCATTAT	(TA)13	(TA)12	TTTGCAAAGGCGATTTTCTT	60.75	193.00
Ca-II-SNP1527	193.29	Ca-*desi*-LG(Chr)8	10009717	TGCTACGTTTTTCAAGGGCAGTATGCGTTCATGTAGACTAAAACTTAAAGGCAGTTTTCGCATTTTGCCCGGTGGAAACTCAGAGTGTTTATGCTATGAG	C	T	ACATCCACAGTTTAGAATCCACATGAAACATGTGGCATACAAGCAACAAATTCCAGCTCATAAATATGTGGTCCCAAATAAATGCAAGTAAAAGATAGTC	NA	NA

Marker IDs	Genetic positions (cM)	Chromosomes	Physical positions (bp)	Flanking sequences/Forward primers	ICC 12968	ICC4958	Flanking sequences/Reverse primers	Annealing temperature (0C)	Amplified Product size (bp)
Ca-II-SNP1528	194.12	Ca-*desi*-LG(Chr)8	10136505	TTTCTTCTTTTGTTTTTTTTTTCGTTGATTAAACCAAATTTTAGGGCTTCTACTTCATTTTCACTCTTTAGTTTATGCATGTTGAGTTTGTTTGTGTGCA	T	G	ATATTAGAAGAAGCATAAAAAAAAAGCTTGAAACAATTAAAAAAATGGTAGCATGTCATTGATGGAGCAATATATATTATATTCATTATCATGTAAGTGA	NA	NA
Ca-II-SNP1529	194.95	Ca-*desi*-LG(Chr)8	10138754	AGTATCAGTACTACAGAGAAGTTGTAAATAAATCTCTTCGCTGTCAACAGTGCCATAGGCCCTTCGTTGCATATATCCTGGATGTGCAAGGTTCATCACG	G	A	CAACATGCGAGTTGCCTTGAGATCCAACATCCACCTTGGAAGTGCCGTGATTCAAACCATCTTTCTGCTGGCCAAACGCTTGCTGACTCGAAGTAGTTGT	NA	NA
Ca-II-SSR433	195.78	Ca-*desi*-LG(Chr)8	10224265	CAAGAATGGTAGCTAGTAGAGGGAA	(GA)15	(GA)17	GGAAGGTGTTTTAGTGGGTG	59.41	133.00
Ca-II-SNP1530	196.61	Ca-*desi*-LG(Chr)8	10225098	ATTGAATATCCACAAGGATCAAATGCAAAATCACAAATGAACGCATTTGGGTTAATTTCGTTTATTCCGGTAAGCTCCGTCATTTCTTTTCCGGCAGAGT	C	G	CAATCAAAGTGATATCTTCACCATGGAAATCTGCATGACAGAACTCGACCCGATTCTAGCTCGCAAATTCTTCCGCCGACCCGGAGACGGAAAAACCGGC	NA	NA
Ca-II-SNP1531	199.10	Ca-*desi*-LG(Chr)8	10247387	GTTGGCTAAATTCAAAGCAACCCAACTCAGTGATCTACATTTGTTTTGGAAGCCTTTGCCATTTCTCTGATAACCAGTTGTATGAGATTGCAAGCGCGAT	A	T	TATTTCTCTGTTCAAATGCCTTTGGCAACCACTTTTCGTTCTCATCCTCATTCCCTTTCTTCTCGGGGACAACCCATATGTATTGGTGCTCTGATGCTTT	NA	NA
Ca-II-SNP1532	199.93	Ca-*desi*-LG(Chr)8	10252427	CAACACGCGCACTTACTTTCCGCGTGATCCTCACTTCACCCCGGATAATGCTCATTGGATTACCACTTGGCAGTTACCACTTACCTTGTGAGCTTTTGTT	A	T	TTTTTAAATTTGTGGAGTATAAATACAAATGTGAGGTATAGGAATTTTTTTAATAATTTTTAGTGAAAAAAGTTTTTTTAAAAAAGGGCAGTCTGATGA	NA	NA
Ca-II-SNP1533	200.76	Ca-*desi*-LG(Chr)8	10512305	TCTTGTAAAGTTCAATCATTAATAGTAAATTAATCTTGTTTGCTTGTCCGTGACGTAGGTCTCATCGATTGAGACTGAACACGTAAATCTGTATGTTATT	A	C	GAAAATGGAGAACGTTTTGGATTTTTCTGCTCAAAACAGATAATGTTTTAAAACAATTTTCACAATCAAATTAAAGAAAATTAAAAAAAGGATAAGGGAA	NA	NA
Ca-II-SNP1534	201.59	Ca-*desi*-LG(Chr)8	10532681	AGCCTCTTGAGAGGGAGGTGTTGGTTACCGGAGGGAGAAAGGACGATCTTTGCCCTCATGCTTGTGTCTTGCAATGCCATCCGGGTCCATGTCCTCCTTG	T	C	CACACTGCTGACCACAGGTAAGATCAGACTGTCGATCGGAGCATCGGGTGGCAATCTTTTTCTTCCCACAAGGGCACAAACGAGGAGGAGCAAATGCTTT	NA	NA

Marker IDs	Genetic positions (cM)	Chromosomes	Physical positions (bp)	Flanking sequences/Forward primers	ICC 12968	ICC4958	Flanking sequences/Reverse primers	Annealing temperature (0C)	Amplified Product size (bp)
Ca-II-SNP1535	202.42	Ca-*desi*-LG(Chr)8	10539192	TTACAATGTACGTGAACTCAGTTTAAGTACGTTTACGTGAACTCAGTTCAAGTACGTTTATGTGAACTCAGTTCACGTACCACCAATGAGTCTCAAACTC	T	C	CGTGAACTCAGTTCACGTACATTTTAAAATTGTAAAAATTTCAGGCAGTACGTGAATTAAGTTCGCATAAAGGTACATGATTTAAGATTGTGTAATTAAT	NA	NA
Ca-II-SNP1536	203.25	Ca-*desi*-LG(Chr)8	10541585	TTTTCAAATCTCATGCTTGTTATGTGTTGTACATTCGTTCGGTTGCTAGGGAAAACAATTTTCATGTTTATTAACCATGGTCCTTCAAAAATGATAGTTC	G	C	AGTCACATCCACACCTTGCGAAGGTAAAAAAAAGTAGGACAATTTCTCAACATTTCAAATTTTCATGAATATTACAACTCAATTTTGAAACAAAATGGA	NA	NA
Ca-II-SSR43	94.41	scaffold00032	297366	AAATGGGTAAATAAAAGCTTGGAA	(AT)12	(AT)15	CAATTGTTGATAGAGTCGTGCT	59.37	170.00
Ca-II-SSR17	79.47	scaffold00046	81914	AAATCGGATGGTGGTGCTAC	(TC)7	(TC)15	AAATTGAAGGATTTGGGCAG	59.82	147.00
Ca-II-SSR36	200.30	scaffold00046	231127	TGCTCTTCAAAAGTAATTGTTCC	(TTATT)9	(TTATTT)8	TTCAATCACATCATTTTCAGGTG	57.64	109.00
Ca-II-SSR7	102.71	scaffold00055	43281	GAAACTTTCATGGTATAAATTCACAG	(TTC)6	(TTC)7	TCCAACACCATTTCCCTCTC	57.45	124.00
Ca-II-SSR25	199.54	scaffold00078	152204	CAATGGCCACTTCAACAATG	(TCT)7	(TCT)6	TCTTCCCATTCCAATGTTCTG	59.96	101.00
Ca-II-SSR34	168.31	scaffold00079	201569	TCTTTTGAAGGTTTTGCACTCA	(AAG)17	(AAG)13	GGCTCAAACCCTCTTCCTCT	59.90	139.00

Marker IDs	Genetic positions (cM)	Chromosomes	Physical positions (bp)	Flanking sequences/Forward primers	ICC 12968	ICC4958	Flanking sequences/Reverse primers	Annealing temperature (0C)	Amplified Product size (bp)
Ca-II-SSR44	97.73	scaffold00079	359228	CAGGGAACGACCTCCTACTG	(AT)7	(AT)6	TTGTTTAGGTAGAGTTGGGAGTAACA	59.72	156.00
Ca-II-SSR18	88.60	scaffold00119	87150	CACAATTTGGGTCGACGG	(AT)8	(AT)7	TCTGGACATCCTTGAAAATTAGG	60.94	125.00
Ca-II-SSR35	190.42	scaffold00119	210194	TGGAACCTTGTGCCTTTTAG	(TATT)12	(TATT)11	CAAAGTGTTTGTTAAATAGATGGGA	57.88	137.00
Ca-II-SSR1	1.90	scaffold00122	14473	TCGGTTCCAACAAATTACGG	(TG)11	(TG)10	TGCAAATTCTCCATCACCTG	60.72	141.00
Ca-II-SSR40	172.06	scaffold00122	273687	AACCCGACACTTAACCGATG	(TTC)5	(TTC)6	TTTGAGAAGGGGTTGAGGTG	59.85	163.00
Ca-II-SSR6	96.90	scaffold00126	38794	GGGCTTTAAATTGAGTGAGGC	(TA)8	(TA)7	TTTGGAAGTTGATTCTGAGCC	60.09	150.00
Ca-II-SSR31	106.86	scaffold00126	178029	AAGCAATGAGTCTTCCACCG	(AATTTG)6	(AATTTG)8	CCCTCTCTCCTCCAAATTCA	60.26	139.00
Ca-II-SSR42	86.11	scaffold00143	295903	GGAATGATGAAGTTACAGGGGA	(TA)8	(TA)9	CGTGACTGGCTCACAAGCTA	60.19	166.00

Marker IDs	Genetic positions (cM)	Chromosomes	Physical positions (bp)	Flanking sequences/Forward primers	ICC 12968	ICC4958	Flanking sequences/Reverse primers	Annealing temperature (0C)	Amplified Product size (bp)
Ca-II-SSR2	47.97	scaffold00156	26854	TTGTTCATGATCTTGAGAAGTGTTC	(TA)19	(TA)15	AGCTAGGGGCATATTAGAGGA	59.69	171.00
Ca-II-SSR33	146.71	scaffold00156	199922	TCACCATTTGTGAAAGTTGGA	(AG)12	(AG)11	CATTTGCACTTCTGCTCTGC	59.01	153.00
Ca-II-SSR38	143.11	scaffold00156	246641	AAATTCACTCTCTTCCCTCTTCA	(ACA)7	(ACA)8	CCTTGTGAATTTCCAATGGTG	58.52	193.00
Ca-II-SSR9	137.71	scaffold00157	54110	GCTCCGTTTCCACTCAGGTA	(TC)8	(TC)7	GCAAATTGAGGGAGGAAACA	60.26	108.00
Ca-II-SSR8	128.44	scaffold00158	47686	TGAAATATCGATCAAATCACGTAAA	(AT)10	(AT)9	TTTCTGAAAACTGTTGAATTCTTATTT	59.77	113.00
Ca-II-SSR26	70.47	scaffold00167	153407	TCCGTCAAAGATGAATGTCG	(AATA)6	(AATA)7	CAAAGTGAACAATTAAAGACAAAAACA	59.65	181.00
Ca-II-SSR27	85.28	scaffold00167	162678	AGTGAGGGGGAGATTTGTTG	(TAT)9	(TAT)8	CCTTCACATGGACCTAGTCCTAA	58.99	158.00
Ca-II-SSR29	93.58	scaffold00173	170626	CCTCTAAGGTCAAACCCCTTG	(TC)13	(TC)14	GCATAGCAGCAAACAAGCAA	59.98	100.00

Marker IDs	Genetic positions (cM)	Chromosomes	Physical positions (bp)	Flanking sequences/Forward primers	ICC 12968	ICC4958	Flanking sequences/Reverse primers	Annealing temperature (0C)	Amplified Product size (bp)
Ca-II-SSR39	147.61	scaffold00185	252811	GGAGCACTCAAACAACTTCCA	(TAA)25	(TAA)24	TCAATGTAAGGACCAGATGAATG	60.28	200.00
Ca-II-SSR23	164.56	scaffold00192	136270	CGAGACAACATAACCTTTCGG	(ATA)6	(ATA)5	GCAAAACAATGTAATTCATATCTCA	59.62	194.00
Ca-II-SSR28	87.77	scaffold00198	169684	AACCCTGTAGCTGCTGATGC	(CT)11	(CT)12	ATTTTGCCACACTTTAGATTATCA	60.43	107.00
Ca-II-SSR10	159.31	scaffold00216	55316	GGTGTTATGCGTTAAGGTAGCA	(AAT)19	(AAT)22	CGTCCTCGTCACCTAATCGT	59.21	182.00
Ca-II-SSR30	104.37	scaffold00216	171314	TGGTCCATGGTACACCACTC	(AT)8	(AT)9	TGTAGGTGCTTCATAAATGCCA	59.23	100.00
Ca-II-SSR16	76.77	scaffold00225	81661	TTATATAAAATTCGATATGCAGGATCT	(AT)15	(AT)16	TCTCAATTCAAACTTCCACTCAA	57.91	145.00
Ca-II-SSR3	65.07	scaffold00229	29109	AATGGAACACCGCCAGTAAG	(AG)9	(AG)15	TGTGGCTAAGCTGCTTGTTG	59.99	170.00
Ca-II-SSR20	106.03	scaffold00233	99420	GAACGAGAATGAACATAGAACGAA	(GA)12	(GA)13	CGGCTGTTACTTCACCGTTT	59.67	148.00

Marker IDs	Genetic positions (cM)	Chromosomes	Physical positions (bp)	Flanking sequences/Forward primers	ICC 12968	ICC4958	Flanking sequences/Reverse primers	Annealing temperature (0C)	Amplified Product size (bp)
Ca-II-SSR11	184.34	scaffold00252	56127	CTCGAGGGTGACCATTGATAA	(TA)14	(TA)11	CCGTCGTATATAGCCGGAGA	59.94	149.00
Ca-II-SSR37	108.52	scaffold00258	245622	CCAAAACGCATGTGTAAAAA	(AT)8	(AT)6	TTGAAAAACTGAACGACACCA	57.24	167.00
Ca-II-SSR41	109.35	scaffold00258	287444	CCCTCAGTATGCCAGTCGAT	(AAG)6	(AAG)7	CACTTGGAAAAGCACACCAA	60.10	148.00
Ca-II-SSR5	84.45	scaffold00270	33338	GCTATGTATCCACTTGCTATGTTTTT	(TA)10	(TA)8	GGAATGATTAGAGATTGTTCTTGTATT	59.14	138.00
Ca-II-SSR22	144.01	scaffold00278	135415	CAACGAATGTATCGCTTCAGA	(TC)9	(TC)10	CGAACAACCTCAGTACCAGAGA	58.93	153.00
Ca-II-SSR4	73.17	scaffold00289	32825	CATTCTAACACGTATCCAAATGAT	(TA)7	(TA)9	AGGCAATTGCACCCACAA	57.20	106.00
Ca-II-SSR19	100.22	scaffold00292	95837	TGAAGAATTGATACACTTACCTCTTTT	(AATA)5	(ATAA)5	TTGACAAACCAATTGAGGTTTATTT	58.09	118.00
Ca-II-SSR13	205.62	scaffold00307	65051	ACATTTTAATTATCTTGACACCGTT	(TA)8	(TA)7	TGCTCCACGAGATATTGAGGT	57.34	156.00

Marker IDs	Genetic positions (cM)	Chromosomes	Physical positions (bp)	Flanking sequences/Forward primers	ICC 12968	ICC4958	Flanking sequences/Reverse primers	Annealing temperature (0C)	Amplified Product size (bp)
Ca-II-SSR15	56.07	scaffold00307	75928	TGGTTCGAAGTTCAAATCTCC	(TA)12	(TA)11	TCATCCCCTCGTTATAAACTTCTC	59.16	102.00
Ca-II-SSR12	198.02	scaffold00316	57060	GGAGAGGGGGTTTATCAATCT	(TC)7	(TC)9	CGCACATCCATTTAAGCAGA	58.40	174.00
Ca-II-SSR21	131.76	scaffold00316	118750	GCATACCCTTTTGTTGACGG	(TGT)5	(TGT)5	TCTCACTTCAACTTTCTCTTCTTTCA	60.37	117.00
Ca-II-SSR14	207.90	scaffold00325	68230	GGGTTCGGGTTCAGAATTTTA	(TAT)7	(TAT)8	GAAATGACCCAGCCCAATAA	60.17	127.00
Ca-II-SSR32	135.08	scaffold00327	178985	CCATATGGCATAATCGTAGAAGA	(TA)14	(TA)13	ACAAAAAGAATAAAAGAGACAACGA	58.19	162.00
Ca-II-SSR24	188.14	scaffold00331	139676	TTGGGATGTGCCTTCAATTT	(CA)6	(CA)7	TGTCACTCGTTTCTTAAAACTTGC	60.31	179.00
Ca-II-SSR114	167.14	scaffold00333	180215	AAAAACAACGGGCGTAACAT	(TA)11	(TA)10	GGAGGAAGAAAATAAAGAATTCCA	59.38	129.00
Ca-II-SSR112	109.84	scaffold00335	139785	TTGGTATTTGAAGCCCACAA	(AAAT)5	(AAAT)7	TCGATCCTTTTATCGTTTGTTTT	59.01	100.00

Marker IDs	Genetic positions (cM)	Chromosomes	Physical positions (bp)	Flanking sequences/Forward primers	ICC 12968	ICC4958	Flanking sequences/Reverse primers	Annealing temperature (0C)	Amplified Product size (bp)
Ca-II-SSR81	159.22	scaffold00337	22733	TCGAGTCAACCAAAATAACCG	(AG)12	(AG)6	CTTGCCTTGCGAGAAGAGTC	59.98	197.00
Ca-II-SSR83	173.30	scaffold00346	28936	ACAAGTTTTAATGGTAGATCAATCTCT	(AT)11	(AT)13	CCTTTCTCCTATTTGCAAGAAATC	57.25	107.00
Ca-II-SSR107	5.12	scaffold00346	110316	TTTTAAAGAGATTTGCAGCGG	(TA)8	(TA)7	TCAAGCATTTTGTGTGAGTGTG	59.50	100.00
Ca-II-SSR105	108.93	scaffold00358	107460	TTCAGAATTTTGGTTTTTGACA	(TA)14	(TA)15	TGACGTGGATGAGTAATTTCG	57.35	135.00
Ca-II-SSR88	184.95	scaffold00360	50340	TTTAAATAGGTCTGGGTCAAACTT	(TTA)10	(TTA)12	AACGACTAATGAGTGATTGCCT	57.48	192.00
Ca-II-SSR101	100.74	scaffold00360	93243	TCATTGTTGATATCGCGCTC	(TA)8	(AT)7	TCCATTTGAGAGACCAACAGTG	59.80	185.00
Ca-II-SSR90	146.15	scaffold00368	61125	CCCACAACCCAGTACCTGAA	(TC)13	(TC)14	ATGAGATTTTGATGGAGGCG	60.80	145.00
Ca-II-SSR78	86.45	scaffold00370	15189	CAATGGCCGACCACTTATCT	(GA)8	(GA)9	TGTGGTCCATGGTTCAAAAT	59.96	127.00

Marker IDs	Genetic positions (cM)	Chromosomes	Physical positions (bp)	Flanking sequences/Forward primers	ICC 12968	ICC4958	Flanking sequences/Reverse primers	Annealing temperature (0C)	Amplified Product size (bp)
Ca-II-SSR73	228.70	scaffold00370	17175702	CAAACTCAAGTTAAGGAGAACCG	(AT)12	(AT)11	CTAACTATCTCAAACTTCCTCTTGC	59.35	101.00
Ca-II-SSR87	224.35	scaffold00381	47571	GCCATGCATTCCGAAAATAA	(AAT)12	(AAT)11	GGGCTTCACGGGTCCTATTA	60.78	191.00
Ca-II-SSR92	77.65	scaffold00381	62796	ATCCATGCAATTGGACACAA	(AT)9	(AT)7	ACTTCAAACGGTGCCCTAAA	59.78	164.00
Ca-II-SSR103	135.71	scaffold00384	102322	TATCGCGCTGAAGAGGTTCT	(TTA)8	(TTA)9	TCACAGAACCTAAACTTCACCAC	60.12	156.00
Ca-II-SSR109	82.05	scaffold00385	116670	CCAAGCTGTTGGAGGAGAAG	(TTA)29	(TTA)27	TCCAAACAAAATATACACATGTTCAA	59.98	182.00
Ca-II-SSR80	129.62	scaffold00394	21061	CAGTTATGCACCATGTGCTC	(GA)9	(GA)8	TGATGTCCTCAACAACAGGC	57.70	102.00
Ca-II-SSR100	83.81	scaffold00398	92449	TCAAGTAGGCATTTCCTCAAGA	(TA)9	(TA)10	CGCCACTGTCCACAAGTTTA	58.97	181.00
Ca-II-SSR93	82.93	scaffold00401	64508	AGGTGCAAACATGAGAAGGG	(ATAA)8	(ATAA)9	CGGAATCCGATTGCTTCTAC	60.11	155.00

Marker IDs	Genetic positions (cM)	Chromosomes	Physical positions (bp)	Flanking sequences/Forward primers	ICC 12968	ICC4958	Flanking sequences/Reverse primers	Annealing temperature (0C)	Amplified Product size (bp)
Ca-II-SSR77	80.29	scaffold00407	9006	GTGGAAGCTGGAGGAGTTTG	(TTG)5	(TTG)6	CCCAACACTGTTTCATTCTTGA	59.84	135.00
Ca-II-SSR84	198.25	scaffold00409	31592	GGGACTACTTTCGCGATTCA	(AT)9	(AT)8	TTCAGTGTCTCGAACCACAC	60.21	114.00
Ca-II-SSR97	161.86	scaffold00414	74911	TTCTTCTCTTTTCCCTCGGC	(AT)9	(AT)11	TGCCTTATGGGTGAAATGGT	60.82	123.00
Ca-II-SSR99	78.53	scaffold00423	83002	CCCCATATCCAAAGTAGCTCA	(AC)9	(AC)6	TGAATTGGGTGCTGATCAAA	59.05	126.00
Ca-II-SSR89	180.34	scaffold00438	54674	TGCAGTCAAAAGTCTGTAACACAA	(TAA)8	(TAA)9	GCCACATTATTGAATGAATAGATAAAA	59.89	148.00
Ca-II-SSR116	175.06	scaffold00446	258085	AAAGTTGAGTGTGAAGGAAATCATC	(AT)12	(TA)11	TGCTCCCAAAACACGATTTTA	59.93	212.00
Ca-II-SSR94	94.37	scaffold00448	66428	AATCCACCGGCTCTTCTCTT	(CT)7	(CT)8	TTGTTCGAGTTTCAGAGCGA	60.21	159.00
Ca-II-SSR106	137.45	scaffold00448	107965	AATGGTCCAATGATATTAATAGATGTT	(AATA)11	(AATA)12	ATTAAGTGGGTATGGGTGCG	57.54	196.00

Marker IDs	Genetic positions (cM)	Chromosomes	Physical positions (bp)	Flanking sequences/Forward primers	ICC 12968	ICC4958	Flanking sequences/Reverse primers	Annealing temperature (0C)	Amplified Product size (bp)
Ca-II-SSR108	13.20	scaffold00451	113441	TGGCAACGACTGGTACAAAA	(AT)9	(AT)8	GTTGGGCCATTAATGAAAAA	60.15	192.00
Ca-II-SSR113	143.54	scaffold00476	142835	CCTTTTTGTTGAGTTAGAAGTGTGC	(AT)8	(AT)11	AATTTACCACATGCATCCCC	60.57	142.00
Ca-II-SSR91	11.18	scaffold00481	61602	AACATTGTTGAGAACACACGAT	(TA)10	(TA)9	GTTACAAACTGATGATGTAACACGC	57.11	100.00
Ca-II-SSR104	164.50	scaffold00488	103491	GCGACACTGCACTTTCAGG	(TTA)11	(TTA)10	GGACCAAAATTCTGTTTGGG	60.61	139.00
Ca-II-SSR96	133.10	scaffold00491	73070	TCGTTCAACAAACAAATTCTCA	(AT)6	(TA)11	TTGGGTGTTGTATCAGTGCC	58.32	154.00
Ca-II-SSR115	174.18	scaffold00514	244293	TGTTCTACGATAGACAAGGAAGAA	(GA)9	(GA)11	TCTATTCTCATTTCACAAATTATTTCA	57.30	163.00
Ca-II-SSR98	12.19	scaffold00517	82863	TTCGCTCAAAAGGAGAAGGA	(ATT)9	(ATT)8	TGGGTTGGATTGTATTTGTATG	60.07	186.00
Ca-II-SSR82	172.42	scaffold00534	24252	GACAACATAGCCATACTCCACG	(AGA)6	(AGA)5	CGCATGGTTTGCACATCTCT	59.52	168.00

Marker IDs	Genetic positions (cM)	Chromosomes	Physical positions (bp)	Flanking sequences/Forward primers	ICC 12968	ICC4958	Flanking sequences/Reverse primers	Annealing temperature (0C)	Amplified Product size (bp)
Ca-II-SSR102	108.02	scaffold00539	93728	TCACAAGAGTGATGTTCGTTG	(TA)13	(TA)12	CCGTCATTACATAACAATTGACC	57.29	205.00
Ca-II-SSR86	220.27	scaffold00555	37885	TCAGATATGGTAATGCTATGGATTTT	(GA)13	(GA)18	AGCATGTCCATGCAATAATAACT	59.26	180.00
Ca-II-SSR111	105.29	scaffold00557	136653	AGGGAGAGGGAATTGCAAAG	(AG)12	(AG)14	TGAACTCGTTTCATCGTTGG	60.57	168.00
Ca-II-SSR74	0.00	scaffold00579	1298	TTTTTCTCTGTTGCAGCTTTGA	(ATAA)5	(ATAA)6	ATCGGCCATAAAGTTGGAGA	60.17	141.00
Ca-II-SSR79	99.83	scaffold00580	18617	CAATTGATCGCAATTCTGCT	(AT)16	(AT)18	ATACACTATTTAAACCAATGGACAAA	58.87	106.00
Ca-II-SSR95	104.38	scaffold00592	70266	TTTTGCCTTTTCCATCATCA	(TTA)6	(TTA)7	TGGATTGAAAAACCATGCAA	59.09	194.00
Ca-II-SSR76	75.01	scaffold00596	4454	CGGTGAATCCTTTACGGAGA	(TA)7	(TA)6	TTTCCTTTCTGTTTTCCTCCAA	60.07	135.00
Ca-II-SSR85	202.05	scaffold00598	36659	AGGTCAGTAGTTTGGCGTGT	(AT)12	(AT)10	AAATGCAGGAGTTTGTTTGAA	57.32	134.00

Marker IDs	Genetic positions (cM)	Chromosomes	Physical positions (bp)	Flanking sequences/Forward primers	ICC 12968	ICC4958	Flanking sequences/Reverse primers	Annealing temperature (0C)	Amplified Product size (bp)
Ca-II-SSR110	89.97	scaffold00604	129503	CCCAGATCAAAATCAAAATCAA	(TC)8	(TC)9	AAAGAAAGGTTCTCGTGTTGTTG	58.92	169.00
Ca-II-SSR75	63.78	scaffold00611	2144	TTGTTGATACTTTAAATGCAAGACA	(GA)12	(AG)6	AAAACAAGTGTTAATCAAGAAGCA	58.03	102.00
Ca-II-SSR144	6.75	scaffold00612	230414	AGCAACGGAAAGGATACACG	(ATT)21	(ATT)31	TGACATTAGTAGGTGTCAACACAGTT	60.13	252.00
Ca-II-SSR145	11.23	scaffold00620	123255	TGTACCAATTTCATGCCATTTT	(AT)13	(AT)12	TCTCATGTTTCCCAACCACA	59.23	178.00
Ca-II-SSR146	5.63	scaffold00626	23909	CCTCTCAAACAAATCCTCTCG	(AT)8	(AT)13	ATAAATCGATATGTGTAGAGCATCA	58.92	111.00
Ca-II-SSR147	16.83	scaffold00628	46633	TGCACCAAGACCAGCTTATG	(TA)10	(TA)9	CTTCATGATTTGATCAATGGCT	59.86	154.00
Ca-II-SSR148	36.46	scaffold00630	91885	CAGCTACGTGCTTCATAGATCC	(TA)12	(TA)11	AGTCCACAAAGTGCAAGGAAG	59.02	139.00
Ca-II-SSR149	38.40	scaffold00630	109187	AAAGACTGAAGAAGGTCCTTTGA	(TA)7	(TA)6	TTCGTCCCTCGGATCATAGA	58.57	159.00

Marker IDs	Genetic positions (cM)	Chromosomes	Physical positions (bp)	Flanking sequences/Forward primers	ICC 12968	ICC4958	Flanking sequences/Reverse primers	Annealing temperature (0C)	Amplified Product size (bp)
Ca-II-SSR150	42.28	scaffold00634	141913	GGGCTAAAGCCCAAATTACAC	(TA)13	(AT)12	GCAACCGTAATATTGGATGGA	59.86	110.00
Ca-II-SSR151	49.07	scaffold00659	119412	TGGTGCATGTAAAATTAATTAAAAGA	(AT)6	(AT)7	TGCACCATAAAAAGCATGACA	58.28	155.00
Ca-II-SSR152	73.31	scaffold00670	160289	CAATTTCAAAACAAATACTAAAAGTGA	(AT)9	(AT)8	CCGACAACGTCTCATACATTC	57.32	190.00
Ca-II-SSR153	88.75	scaffold00703	31447	TTCATGTTGGATTGGTGTTGA	(AG)12	(AG)13	CCTTCTTCACCATCCACTTCTC	59.81	100.00
Ca-II-SSR154	103.25	scaffold00719	39896	TGTGAACTCCCTTCACCCTT	(TTC)6	(TTC)5	CCAAACCTAGCCGTGACACT	59.55	127.00
Ca-II-SSR155	112.75	scaffold00724	106748	TAAGCAAATTTGGCAAGCAA	(TAT)13	(TAT)9	GTATCCTTCCATTCCCCCTC	59.47	197.00
Ca-II-SSR156	114.65	scaffold00731	60987	ACACATCGTGGTTGCATACG	(AT)9	(AT)8	TTTAACACTTTCACCCTCTCATTG	60.46	100.00
Ca-II-SSR157	153.61	scaffold00747	54731	GCAAACAAGAACCGAAAACA	(TTA)10	(TTA)9	TGTTGACACCTAATTTTGTCCG	58.80	168.00

Marker IDs	Genetic positions (cM)	Chromosomes	Physical positions (bp)	Flanking sequences/Forward primers	ICC 12968	ICC4958	Flanking sequences/Reverse primers	Annealing temperature (0C)	Amplified Product size (bp)
Ca-II-SSR158	126.05	scaffold00760	51907	TTACATGAAAAATAAAAACAAGCAA	(TC)8	(TC)9	AGCAATGGAAAGGTCCCATC	57.26	190.00
Ca-II-SSR159	162.25	scaffold00777	19305	TCATGTGACCATACAAAGCATTC	(TAT)26	(TAT)27	TCAAGCTGCAAAGAAGAAACC	59.88	260.00
Ca-II-SSR160	168.01	scaffold00792	53958	GTGGTGAGGTTAGGTGGTGC	(TAT)23	(TAT)24	TGAGGGATACATGAGGGACC	60.43	246.00
Ca-II-SSR161	135.55	scaffold00795	12245	GAGATGGTGAATCATAATAACCTAAAA	(AAT)19	(AAT)20	ATTATTCATCCCGTCCCAAA	58.16	138.00
Ca-II-SSR162	186.25	scaffold00795	94285	TGTCTCCAATCCTATAGCCAAA	(TA)7	(TA)6	CTTGTTCATCAATAGAATTACCTACCT	58.72	100.00
Ca-II-SSR163	195.24	scaffold00807	516	ATGGCACTGCATCTGAAACA	(ACA)5	(CAA)5	CCAAATTAAGCCTTTGGCTC	60.27	108.00
Ca-II-SSR164	201.30	scaffold00825	55018	GGCTAGGTTAGAAATTTTACATATTGA	(TC)8	(TC)7	CCCAACTTGTTGACATTATCAAAG	57.68	154.00
Ca-II-SSR165	208.37	scaffold00825	90232	GGGCTTGCAATGTACAAGAA	(AT)7	(AT)8	ATGCGTCGGTGCTAGCTAAA	58.77	146.00

Marker IDs	Genetic positions (cM)	Chromosomes	Physical positions (bp)	Flanking sequences/Forward primers	ICC 12968	ICC4958	Flanking sequences/Reverse primers	Annealing temperature (0C)	Amplified Product size (bp)
Ca-II-SSR166	225.54	scaffold00840	67415	AAAAACCACAACCAAAAAGTTTATTC	(AAT)25	(AAT)22	CCGAGTTTCGAACTTTAAATTGTAT	59.97	205.00
Ca-II-SSR167	230.59	scaffold00851	103140	ATTGGAGAAGCATTTGTCGG	(GA)16	(GA)17	TGGCATTTAACTTGGACGAA	60.07	182.00
Ca-II-SSR168	237.66	scaffold00852	13148	ACACAAATTGCTTTCGCAAC	(AT)11	(AT)12	CGAAATCGAAATCATTGTTCA	58.82	187.00
Ca-II-SSR169	252.67	scaffold00885	82619	GCAGCAGTTTTTCTGTGCAA	(TTAT)7	(TTAT)9	TGACTATGTGAGTTAAAGATTCCAATG	60.18	121.00
Ca-II-SSR170	258.31	scaffold00888	7232	CAAATTCAAAACAAATAACCATGT	(ATA)19	(ATA)16	TTTTAATGGATGAAATCGAAATTCT	57.18	170.00
Ca-II-SSR171	256.43	scaffold00907	141878	TCTTTTGATTTAATTTTGGAGTGTTTT	(TA)10	(TA)8	TGGATGGAGTTAGTTAGACTCTTGG	59.76	159.00
Ca-II-SSR172	10.11	scaffold00908	148256	GGTCTTACATTGTGTTTTCAAAGAA	(ATT)16	(ATT)17	GGGTTCAACTTCAAAAGCCA	58.71	128.00
Ca-II-SSR173	20.19	scaffold00912	14526	TACCCACACCCCTCAAATGT	(TCT)7	(TCT)6	TGTGTTGAATGAAGATGATGAAGA	60.09	124.00

Marker IDs	Genetic positions (cM)	Chromosomes	Physical positions (bp)	Flanking sequences/Forward primers	ICC 12968	ICC4958	Flanking sequences/Reverse primers	Annealing temperature (0C)	Amplified Product size (bp)
Ca-II-SSR174	33.55	scaffold00928	35611	TGGTGTGTTGCTATACATGCC	(TA)9	(TA)10	CTCCAACCAATTATGGACGC	59.48	135.00
Ca-II-SSR175	34.52	scaffold00965	32759	CCCTTTAAAACAATTAAGCATCG	(ATT)20	(ATT)15	TGACGTGGAAACATTTGTGG	59.13	152.00
Ca-II-SSR176	41.31	scaffold00978	49512	TCAAAACTCAACTTCGCCATT	(AT)10	(AT)9	GGTTTGCAAAATAGTGTGGC	59.74	134.00
Ca-II-SSR177	46.16	scaffold00997	30906	TGCAAACATTGATTTTGTAATTTG	(AT)9	(AT)8	GCATTTTAGATCAATTTGCTTTTG	58.99	176.00
Ca-II-SSR178	72.28	scaffold01004	239200	GTAGGTTATTTTCACATCAATGTATCA	(AT)11	(AT)12	GAAAAGTCCAAAAGCAACCG	57.68	111.00
Ca-II-SSR179	91.45	scaffold01018	64137	TCTTTCAACTACTGACTTCTGCTCA	(ATT)22	(ATT)28	GAGACAATTGTGTGGAGGTTG	59.76	206.00
Ca-II-SSR180	105.15	scaffold01040	84732	GCCCCTTTGAGTCTTGCTCT	(AAG)12	(AAG)11	TTCAACCTACCATGGGCTTT	60.90	181.00
Ca-II-SSR181	110.85	scaffold01046	10884	GGGTGACACTGGATTGGGTA	(ATAACA)6	(ATAACA)5	TGACTTTGGCAAAACCATCA	60.63	167.00

Marker IDs	Genetic positions (cM)	Chromosomes	Physical positions (bp)	Flanking sequences/Forward primers	ICC 12968	ICC4958	Flanking sequences/Reverse primers	Annealing temperature (0C)	Amplified Product size (bp)
Ca-II-SSR182	113.70	scaffold01046	92052	TTTCACTCGGTTTTAATCTCCA	(TTA)5	(TTA)15	TCATTTGATGGAGATTGGTTG	58.74	172.00
Ca-II-SSR183	125.10	scaffold01086	69905	TTCTCTCATACATTGTCTCTCTCTTTC	(CT)8	(CT)9	GGTGATTGAAGTTTTCCAACAA	58.73	151.00
Ca-II-SSR184	127.00	scaffold01090	109221	AATATCGAAAATTACCGTGGAA	(TA)12	(TA)7	TTTCTTAATCATTTGTTTCAGAGTCTT	57.60	100.00
Ca-II-SSR185	168.97	scaffold01092	90916	TGGAGGGAGGACATACACTTG	(AT)19	(TA)16	TGTCTCTCTTTCCCTCGTTG	59.97	115.00
Ca-II-SSR186	136.50	scaffold01093	56567	ATGGCCGTGTCCAATAAAAA	(ATTC)5	(ATTC)6	GCTTCAATATGCGGGTCATT	60.19	141.00
Ca-II-SSR187	170.89	scaffold01109	52905	CGAAGAGACATGACTTTGTCCA	(TA)11	(TA)15	TTTAGAGCTGATCATTCAAAAAGA	60.29	219.00
Ca-II-SSR188	185.29	scaffold01121	18387	CAAATACACTTACATTCAATTCTTTCA	(AT)9	(AT)8	CTGCCTACTCATGTTTCAACTTT	57.95	200.00
Ca-II-SSR189	194.23	scaffold01133	48159	AAGTGGACATTGGGATTGGA	(AT)10	(AT)11	TGATTCCCCAACAATCTTAAAAA	60.17	140.00

Marker IDs	Genetic positions (cM)	Chromosomes	Physical positions (bp)	Flanking sequences/Forward primers	ICC 12968	ICC4958	Flanking sequences/Reverse primers	Annealing temperature (0C)	Amplified Product size (bp)
Ca-II-SSR190	203.32	scaffold01134	88246	TGTTGTTGTGGGGTTGATTG	(AC)11	(AC)12	GAGAATGTTGCGTGGTCCTT	60.26	139.00
Ca-II-SSR263	203.19	scaffold01155	53620	TGACTAAATCGAGAGTTTAAACAGGA	(TA)11	(TA)9	AGCTTGGCAAACCTATCGAC		59.75
Ca-II-SSR245	148.53	scaffold01203	35498	GCAGATGTGTGCGACTTTCT	(AT)7	(AT)8	TTGGGCTTATGTGCAATATAAAAA		59.04
Ca-II-SSR210	0.00	scaffold01218	897	TCAGACATTGACAACAGAACCA	(AT)13	(AT)11	GTGGTGGGTGATTCTCACAA		59.19
Ca-II-SSR280	309.41	scaffold01236	103282	CCAACACTTTGATGTTTGTGAA	(TTA)9	(TTA)10	GCGGAACGTATCAAAATCTAACA		58.58
Ca-II-SSR267	101.34	scaffold01245	57148	CGTCTAACTTTAATTGGAATTGTTC	(AT)7	(TA)7	AAATATTTGTTTAAGCCACCAAAA		57.56
Ca-II-SSR229	314.51	scaffold01280	20397	TCCTTTTCTTTTCTCCCCAAA	(AT)13	(AT)12	TGCGGTGAATCTTTTACGAA		60.03
Ca-II-SSR249	33.58	scaffold01291	38908	TGCAATGAACAACAATTTTCAA	(ATA)6	(ATA)7	AGCTGCAACAGCAAGCCTA		59.10

Marker IDs	Genetic positions (cM)	Chromosomes	Physical positions (bp)	Flanking sequences/Forward primers	ICC 12968	ICC4958	Flanking sequences/Reverse primers	Annealing temperature (0C)	Amplified Product size (bp)
Ca-II-SSR219	243.14	scaffold01321	6929	ATCGGTTGGAATCACTTTCC	(AAAT)12	(AAAT)14	ACATGGGTCAAGCCCAATAA		58.84
Ca-II-SSR231	127.96	scaffold01327	21087	CCTGATGAAGAATACATTTTGTGC	(AT)9	(AT)10	TTTGTTGTGTTCTCTACCATTTGTC		59.90
Ca-II-SSR271	231.64	scaffold01330	60713	TGTTTAAAATGAGTCACGCCA	(TA)6	(TA)7	AGAGCATGGGTCAATCTTGG		59.22
Ca-II-SSR256	131.59	scaffold01332	45869	TGAATTGAATTGAATGAAACCC	(GAA)11	(GAA)9	CCAACGCTTTCTTCTTCCAC		58.79
Ca-II-SSR262	192.69	scaffold01335	53353	TGTACAATGACATGGGAGACTCTT	(AT)9	(AT)10	AAAAATGGGTCAACTATTTGGATT		59.93
Ca-II-SSR226	303.29	scaffold01347	15322	TGTGTCTTGAGGAAATTGATGA	(TA)8	(TA)6	TCCTTTTACTATAAAGTTTTCCGCAT		58.20
Ca-II-SSR265	61.41	scaffold01364	55137	TCAGTTCTCACTTGATACGCTC	(TA)13	(TA)12	GAACTTTCTTGTGTTCTATAATTGCG		57.23
Ca-II-SSR211	11.80	scaffold01381	3060	GTATTGCGCGCAGCTTTTAT	(TA)11	(TA)12	TCTTTTGATTTCTTTTGTCATTCA		60.38

Marker IDs	Genetic positions (cM)	Chromosomes	Physical positions (bp)	Flanking sequences/Forward primers	ICC 12968	ICC4958	Flanking sequences/Reverse primers	Annealing temperature (0C)	Amplified Product size (bp)
Ca-II-SSR274	287.99	scaffold01385	68176	TTGGAGAGTGTTGCTGGTTG	(TA)11	(TA)13	CGAGCATAATTTTCTTTTCATTACC		59.87
Ca-II-SSR278	160.41	scaffold01385	76539	CGCTGTAACATGTACACCCAA	(TG)8	(TG)7	TGCCATAAAAAGCTAAAAAGCG		59.52
Ca-II-SSR215	94.08	scaffold01404	4084	CATCCAGTTCGACGATATGC	(TAT)9	(TAT)15	AGCTTCAAGTGGAAAATAAACATT		59.11
Ca-II-SSR225	159.42	scaffold01420	15047	TTCCTTTGCCATTTATGTTTTG	(ATA)5	(ATA)6	TAGCATCACATCATTTTATTCTTGT		59.02
Ca-II-SSR232	147.32	scaffold01425	22480	TCGTGAATTGCTGCAAGATT	(TA)8	(TA)10	TGGAAAGGTACACCTCCACAT		59.43
Ca-II-SSR218	224.19	scaffold01427	5868	CTGAACCAGTCTCTCGTCACA	(TC)6	(TC)20	GGCTGCATTGCAAATAACTG		59.03
Ca-II-SSR276	204.24	scaffold01453	70656	TTTGCTTCGACACTGTAGCAC	(TA)15	(TA)10	TGCACGGCAATTACAACAAT		59.14
Ca-II-SSR275	193.74	scaffold01480	69857	TGAAGACCATCCTACAAACAAAG	(AT)11	(AT)13	CACGACAACGCAAGTAAAAA		58.30

Marker IDs	Genetic positions (cM)	Chromosomes	Physical positions (bp)	Flanking sequences/Forward primers	ICC 12968	ICC4958	Flanking sequences/Reverse primers	Annealing temperature (0C)	Amplified Product size (bp)
Ca-II-SSR223	292.07	scaffold01498	12514	TGAGGTAAATAAACCGACTCCAA	(AT)13	(AT)15	AATTATTTGTTGTTAAGGCGAGTT		59.89
Ca-II-SSR244	130.38	scaffold01508	34787	GGAAATCCAACCTCATCTTCA	(CTT)19	(CTT)22	TGAAATTGCTCTTTTCACCAGA		58.97
Ca-II-SSR269	132.80	scaffold01522	57922	GGAAGAAACAACCTTCTCACCTT	(TAT)8	(TAT)5	CAACAAGGTCGATCTCCTCA		60.03
Ca-II-SSR272	256.94	scaffold01526	63824	AATATTTGCATTGAGGATGACTTG	(TTC)5	(TTC)6	TGACCAAAAACCACCCTCTT		59.42
Ca-II-SSR260	258.09	scaffold01529	51454	TGATTGCTGATGGCACTCAT	(TTA)27	(TTA)17	CCGGAATGTGGATCATGC		60.23
Ca-II-SSR255	98.92	scaffold01544	45590	TTCCTTGTCTCTCACTGCCC	(CT)11	(CT)12	CGGAGAAGAAGCTGTTTTGG		60.39
Ca-II-SSR266	89.24	scaffold01547	56961	TTGGCTTAAACAACACACCAA	(TA)15	(TA)16	TGGTACCGACGAACCGTAAT		59.12
Ca-II-SSR279	305.33	scaffold01565	90966	TTAAACCATTTAAATTTGCAACAA	(AT)15	(AT)14	TCATGATGTTCGTTCAAGTAATGA		57.85

Marker IDs	Genetic positions (cM)	Chromosomes	Physical positions (bp)	Flanking sequences/Forward primers	ICC 12968	ICC4958	Flanking sequences/Reverse primers	Annealing temperature (0C)	Amplified Product size (bp)
Ca-II-SSR241	85.61	scaffold01576	31865	CATCATTTATTGAGAGTTGGGTAGTT	(TG)8	(TG)9	TTAAGTCTGGCCCATCAAGG		58.97
Ca-II-SSR220	178.23	scaffold01589	8052	TCGGTCTTTCATGATCTCACC	(TA)10	(TA)11	GAAGAGAAAGAACCCCACTAGG		60.07
Ca-II-SSR248	247.74	scaffold01618	37514	ATTTTCATGTTGTAGATGAGATCAAT	(AAT)25	(AAT)26	GCTTACAAGTTGCAACCCAA		57.31
Ca-II-SSR270	152.49	scaffold01619	58432	CGACAAATAATTTTATTCGGGGT	(TA)11	(TA)13	CAATTGAATTTTAGTTTGGGGA		60.30
Ca-II-SSR268	102.55	scaffold01632	57696	CGTTAATTCATGGGATGGTG	(TAT)5	(TAT)6	AATGCTCGTAACATACACATCTTT		58.70
Ca-II-SSR237	19.06	scaffold01638	30068	CCAATCAACAACCCTTCGTT	(TC)11	(TC)9	AGAAACTCCTGCACCGTCAT		59.83
Ca-II-SSR277	269.59	scaffold01647	74883	TGTGAAATGGATTGGGCAGT	(TA)6	(TA)11	TTCTCGTTTTTCACAATAAATATCAA		61.31
Ca-II-SSR251	202.14	scaffold01649	40919	ACATGATGAGAGAAACCCGC	(AG)7	(AG)8	GCCATCCATTTATTGCATTTG		60.08

Marker IDs	Genetic positions (cM)	Chromosomes	Physical positions (bp)	Flanking sequences/Forward primers	ICC 12968	ICC4958	Flanking sequences/Reverse primers	Annealing temperature (0C)	Amplified Product size (bp)
Ca-II-SSR273	250.04	scaffold01689	63911	ACTTAAAATGAGGGGTGAAACTT	(TA)11	(TA)10	GGCTTCAGAAATAATTCTCCACA		57.38
Ca-II-SSR253	86.82	scaffold01716	43992	TCACAGAAAGCCAAAAGAAGG	(AG)9	(AG)8	TCTAGGGTTGTGATTTCTAGGTCA		59.48
Ca-II-SSR216	126.75	scaffold01788	4719	TTAAGTTGAAGAAAGCGTGTTG	(TAT)10	(TAT)9	GGGGCAGCTGTAATACCTCA		57.34
Ca-II-SSR243	96.50	scaffold01803	33820	TTGCATCGCATTTGTTCTTG	(AG)6	(AG)7	GGGCTTCTTTGAAGGAAACAT		60.79
Ca-II-SSR212	49.31	scaffold01838	3309	AAATGCTCATAAATCCATGTCTGT	(AAT)15	(AAT)12	TACATTGCCTGCAACGAAAC		58.96
Ca-II-SSR224	299.21	scaffold01855	13824	CTTGGTTGTCATCTTGATTCTCA	(AT)7	(AT)9	CCAATATATAGGACACCGACACA		59.23
Ca-II-SSR240	50.52	scaffold01886	31675	TTTCCTTTCTTTTTCCCTCCA	(TA)10	(TA)8	TATTTTGGATTTTGGCTCGG		60.03
Ca-II-SSR258	230.49	scaffold01910	48198	GAAAACAGCTTAAATCATTTCACTCA	(TA)13	(TA)14	TTGTCGATGAATCACAAAACA		60.07

Marker IDs	Genetic positions (cM)	Chromosomes	Physical positions (bp)	Flanking sequences/Forward primers	ICC 12968	ICC4958	Flanking sequences/Reverse primers	Annealing temperature (0C)	Amplified Product size (bp)
Ca-II-SSR264	268.44	scaffold01927	54554	AGAAAAGCATAATTCGGACAAT	(TAT)27	(TAT)24	TGCAGTATGATTGTTGATCAAGTG		57.00
Ca-II-SSR259	246.59	scaffold01928	49480	CCACATGCCCCCATATATTC	(ATA)15	(ATA)10	AACAATGGTCTACGGTGGATT		59.86
Ca-II-SSR238	201.09	scaffold01951	30269	TTCACAAAACAAAATAAAGAATGAGA	(AT)14	(AT)15	TACGCGGGTGTCTTCAGATT		58.42
Ca-II-SSR235	185.34	scaffold01955	24240	GAGGAATTATTGTGATGATTGATG	(TAT)9	(TAT)8	CAAACCAAAAGTTAAACACACCA		57.52
Ca-II-SSR234	179.22	scaffold01970	24170	CGATGTTGAAGAGGTTTAGCTG	(TA)9	(TA)10	TGAAGTGGTATATCTTAACCCAGGA		59.04
Ca-II-SSR221	284.54	scaffold01989	8513	CAGTGTTACCTCATGGAACCAA	(TA)14	(TA)12	ACGCAATTGCCATGGTTTTA		59.89
Ca-II-SSR239	267.29	scaffold02000	31067	TTGAACAGCTACCGAATATGGA	(ATA)16	(ATA)13	CCTATTAGGTCTGACAGGACGA		59.61
Ca-II-SSR233	226.29	scaffold02037	23187	TTTCCATCTTCTGCCAATCC	(AT)9	(AT)10	AATTTAATTTGCTCTTTATTATGCAGT		60.01

Marker IDs	Genetic positions (cM)	Chromosomes	Physical positions (bp)	Flanking sequences/Forward primers	ICC 12968	ICC4958	Flanking sequences/Reverse primers	Annealing temperature (0C)	Amplified Product size (bp)
Ca-II-SSR252	60.20	scaffold02048	43471	ATCATCAATCATACAACATCACG	(ATT)31	(ATT)20	TGTTGTGACATTTAGTGTCGAATG		57.38
Ca-II-SSR217	141.27	scaffold02082	5575	TTCTCTTATTCTTCAATGATCCTTAGT	(TA)14	(TA)15	TGATTTTCAAAAACTCAAGAAGACT		57.42
Ca-II-SSR250	191.64	scaffold02087	40378	TCACCATCAGGACTCTGCAA	(AT)13	(AT)12	CCAAAAATTTTCAATTACTTGATCC		60.40
Ca-II-SSR213	81.98	scaffold02104	3371	CACCCTCTGTATGGCAGTCA	(AAG)7	(AAG)6	TCATTTGGAAAAGCACACCA		59.70
Ca-II-SSR228	311.45	scaffold02109	17748	GGAGTTGTTGGCGAGTTGAT	(CT)8	(CT)9	GACAACTATGTGGCGCAGAA		60.12
Ca-II-SSR236	32.37	scaffold02116	26571	AACATTGTCCTTGTCTTAGTCTTGG	(AT)6	(AT)7	TGAAAATCATTAGAGAGCACTTCA		59.98
Ca-II-SSR227	307.37	scaffold02164	15661	ACGTGGCATAATCAGACGTT	(AAT)11	(AAT)12	GCTGTCTGCAGAGTGCATTT		58.10
Ca-II-SSR261	286.84	scaffold02243	52608	TGAATTATTTGTCCTTACCATCTCTTC	(TA)7	(TA)6	AATTAACTCTCAAAAACCCGACTC		60.14

Marker IDs	Genetic positions (cM)	Chromosomes	Physical positions (bp)	Flanking sequences/Forward primers	ICC 12968	ICC4958	Flanking sequences/Reverse primers	Annealing temperature (0C)	Amplified Product size (bp)
Ca-II-SSR222	17.85	scaffold02281	9375	AAATAACTCTTAATCATGACCATCCA	(AT)13	(AT)17	GGGAAAGTGGCGAAAGGATA		59.25
Ca-II-SSR257	149.52	scaffold02331	46651	GTGGACGAGAAGAGGTTGGA	(GA)13	(AG)12	CCGGAGGAGAGTGATTGTTC		60.24
Ca-II-SSR214	91.66	scaffold02336	3789	TGGAGCGTTTTCTCTGAACA	(AT)14	(AT)16	TGAAGTTAAAGTCGAAGAAATGACA		59.57
Ca-II-SSR246	228.39	scaffold02408	37253	ATGCAAGCCGAGCATTTAGT	(TTA)10	(TTA)11	TCGGAAGGATACTTGTGATGTG		59.87
Ca-II-SSR254	97.71	scaffold02430	44672	CTGGCATCACTGTCAAGGAG	(TA)13	(TA)14	GTGCATCATGGTTGTTGGTT		59.42
Ca-II-SSR242	109.81	scaffold02435	31937	AATTGGAGATCGGTCGTGAG	(GA)15	(GA)14	CACCAAAACCGCATAAAACC		60.07
Ca-II-SSR247	184.29	scaffold02437	37521	TGTTTCTCTGTCTTCAATTTGTTG	(TC)8	(TC)9	TCTCCCATAGCTGCACACTG		58.48
Ca-II-SSR230	108.60	scaffold02462	20792	TCTCGAAAGAATTTGATCTCCA	(AT)10	(TA)11	GAATATACCCGATCTACAATTAATGC		58.90

Marker IDs	Genetic positions (cM)	Chromosomes	Physical positions (bp)	Flanking sequences/Forward primers	ICC 12968	ICC4958	Flanking sequences/Reverse primers	Annealing temperature (0C)	Amplified Product size (bp)
Ca-II-SSR319	70.86	scaffold02487	32478	TTTGAATCCACCCACACTAGC	(AGA)5	(GAA)5	CGTGAGAGGAAAGAAAGAAAGG	59.99	105.00
Ca-II-SSR327	160.32	scaffold02501	119889	GGCCAGATCGATGAAATGAC	(TTC)7	(TTC)6	TTGATTGTCTCTGATTTTACAAGGA	60.44	125.00
Ca-II-SSR322	106.20	scaffold02520	35644	AAAGAATTGTCTTTTGGTCATTTT	(AT)8	(AT)9	TTATTCAAAATGAGACTTTTGCTTT	57.45	141.00
Ca-II-SSR324	134.70	scaffold02575	38694	TGCTGCAATGAGTCTCCTTAGT	(AT)9	(AT)11	GGAGACCGAATTTTGAATGAA	59.15	172.00
Ca-II-SSR307	151.80	scaffold02585	12978	TGCGTTACGGGTAAAATGGT	(AT)9	(AT)10	TTTCCTTTCTTTTTCCCTCCA	60.24	118.00
Ca-II-SSR309	158.34	scaffold02606	21574	TCTCACTAATCCAAAACACCAAAA	(AAT)24	(AAT)26	TGAACAACTCAAATAAACAATCTTCC	59.91	267.00
Ca-II-SSR302	68.58	scaffold02620	5627	TGGTGGTGAACTTTTGTGGT	(TG)8	(TG)9	TCTTGAGAAGTTGTGCACGG	58.88	123.00
Ca-II-SSR318	14.30	scaffold02667	31666	GAGAAAGAAGAACGGTTGTTACC	(AAG)7	(AAG)9	TTCCCTTCTTTCCCGAATCT	58.38	129.00

Marker IDs	Genetic positions (cM)	Chromosomes	Physical positions (bp)	Flanking sequences/Forward primers	ICC 12968	ICC4958	Flanking sequences/Reverse primers	Annealing temperature (0C)	Amplified Product size (bp)
Ca-II-SSR314	203.88	scaffold02674	27650	CTACAAATGATCCCCCAAGC	(GA)20	(GA)21	CCTAACAATAATACGGTCATTTGTTCT	59.39	237.00
Ca-II-SSR306	35.52	scaffold02688	9765	CTTTTTAGTTCCCGTGTGCC	(TTC)5	(TTC)7	GAAAATGGAGGTTTGCGTTC	59.61	141.00
Ca-II-SSR310	236.55	scaffold02701	21753	TGATCAATCACCTTCTCGGA	(ATT)21	(TAT)20	TCCCATGAAAGAGAAGCTCG	59.17	222.00
Ca-II-SSR308	183.09	scaffold02705	19900	GGACCAAAACTAGGGGGAAA	(TA)8	(TA)9	CGACGAGTTTCACCAAATCA	60.16	190.00
Ca-II-SSR305	34.38	scaffold02714	9100	GAGACTACAACAAAACCCAAAAA	(AT)13	(AT)11	CTCGAGTTCGAATCTGGGAC	57.43	191.00
Ca-II-SSR312	178.14	scaffold02799	23183	GAACTTGGGTAGAACGGTACTATTT	(TA)11	(TA)10	CATCCAACAAGGACGGAGTT	58.25	158.00
Ca-II-SSR299	2.40	scaffold02862	544	GGCTGCGGTGAATCTTTTAC	(AT)8	(AT)17	TCCTTTTCTTTTCTCCCCAAA	59.71	189.00
Ca-II-SSR316	210.81	scaffold02874	29955	TGGGGCTGTTGTTTTAGAGG	(GA)12	(GA)13	CACCACAAATTTGCGCTTTA	60.10	170.00

Marker IDs	Genetic positions (cM)	Chromosomes	Physical positions (bp)	Flanking sequences/Forward primers	ICC 12968	ICC4958	Flanking sequences/Reverse primers	Annealing temperature (0C)	Amplified Product size (bp)
Ca-II-SSR303	78.84	scaffold02887	6803	ACTCTCACACCTCCCCCTCT	(TG)11	(TG)12	CAAACCACCATTTTCTGTGTG	60.11	109.00
Ca-II-SSR317	223.68	scaffold02920	30731	GGGGGAAAACGTTTCCTTT	(TA)8	(TA)7	TGAGATGTGAGTGAAGTGATGTG	60.15	133.00
Ca-II-SSR323	133.56	scaffold02923	35666	TTAAAACAAACGCACCCATT	(AT)11	(TA)9	AAATTGATATGTGTAGAGCATCATGT	58.07	167.00
Ca-II-SSR320	73.14	scaffold02932	33000	TAGTGCATCAAGGCCATTTG	(AAT)14	(AAT)13	AAGTTTAAAAGTTTAATTGGGGCA	59.69	162.00
Ca-II-SSR321	79.98	scaffold02984	33584	TCCACTATTATGGCGAAAAGTT	(TA)10	(TA)7	ATCATGTGGAAACAAACGCA	57.85	140.00
Ca-II-SSR326	253.47	scaffold03073	40474	GTTTTGGGGAATTTGAGGGT	(GTG)5	(GTG)6	CTCCTCCGAAACCAAAAACA	60.03	149.00
Ca-II-SSR304	103.92	scaffold03176	8432	GGAGATTGACACGTCCCGTA	(TA)13	(AT)12	TGTTCTGTCGAGGAAGAGGAG	60.92	147.00
Ca-II-SSR311	237.72	scaffold03215	22172	CCGTATCTGAGGCTTTTCTCA	(TTC)8	(TTC)5	CCCTCTGTATGGCAGTCGAA	59.46	106.00

Marker IDs	Genetic positions (cM)	Chromosomes	Physical positions (bp)	Flanking sequences/Forward primers	ICC 12968	ICC4958	Flanking sequences/Reverse primers	Annealing temperature (0C)	Amplified Product size (bp)
Ca-II-SSR313	197.94	scaffold03300	23828	AGGGCAGATGTAAAGTTGCG	(AG)13	(AG)14	CCCTTTCCTCCACTCTACCC	60.27	181.00
Ca-II-SSR300	10.73	scaffold03309	5226	GATTGAAACTTTGTTCCTAAAGTGA	(TA)14	(AT)15	GTTAAAGGTTTCAAACTTCATGACT	58.03	140.00
Ca-II-SSR325	252.50	scaffold03391	39428	TCAACCTTGTTTATTTAATTCCACC	(AAT)13	(AAT)10	AGTAGTTCATCATGTGACAAATTAAAA	59.59	200.00
Ca-II-SSR301	64.02	scaffold03401	5376	ATTCGTTTGGGTTTGGATTT	(TA)9	(TA)11	AAAAGGACGATGGGTTTGC	58.28	117.00
Ca-II-SSR315	206.85	scaffold03419	27662	TCCAGTTTGGAAGTTTGGAAG	(AAT)24	(AAT)27	CAGTGATCAAATAAATATCACATTGAA	59.20	183.00
Ca-II-SSR355	139.56	scaffold03420	8229	CCCTCCAACACGTGGACTAA	(TAT)6	(TAT)7	AATTTTGATTGGTCAAAATGCAATTAGTTAACTT<br>GATTGATTTGTTGGACCTAAATTAAATTGGCTA<br>ATAAAGTATCTTAATAATGATTTGATATTATAA	TTTTGATTGG<br>TCAAAATGCA<br>A	60.94
Ca-II-SSR351	175.83	scaffold03480	7425	TTCAAACTCCCTTCCTTTAAAAGTT	(TA)13	(TA)12	TTTATTTTATCTAAATAAATGACGGAAATAGATT<br>AAGTTGTCTGTAAGAGGTCTATGGTCTGACCTG<br>ACATTTTTAATAAAAAGACTACGCTCATATTTA	GGTCTATGGT<br>CTGACCTGAC<br>ATT	59.94
Ca-II-SSR361	78.24	scaffold03542	16118	CCGTCGAAATATTTTCCTCTTC	(TA)10	(TA)11	TAATAATACTAGTATAATTTTTTAGAGAAATGTT<br>TTGTTATGAAGTGTATTTGTAAGTAACATCTAAC<br>ATTTTTAAATTGTAATTATTTACTCTCTTTTA	GAAATGTTTT<br>GTTATGAAGT<br>GTATTTG	59.12

Marker IDs	Genetic positions (cM)	Chromosomes	Physical positions (bp)	Flanking sequences/Forward primers	ICC 12968	ICC4958	Flanking sequences/Reverse primers	Annealing temperature (0C)	Amplified Product size (bp)
Ca-II-SSR370	79.08	scaffold03589	26039	CTGGCTATGACAAAGTGGCA	(AGA)5	(AGA)6	CAAACATTCACCTTATTCCTAAACCCATCCACTTATCATTTGACGTGAAAAGAAAGAATCTAAAAGAAAAAAAAGAACACTTAATCACTCTCTAACTTCT	CCTTATTCCTAAACCCATCCA	59.86
Ca-II-SSR369	37.60	scaffold03648	25093	AGGCCTGACAGTCCAACCTA	(TTA)14	(TTA)19	TTATTACTTCAAACAAATATGGAATGACTACTGAGTGATTGCTTAATTGATAAACTTTAAAATATGAAATAATATTATTAATAAATAATAATAATAATAA	GGAATGACTACTGAGTGATTGCTT	59.72
Ca-II-SSR371	99.24	scaffold03653	35219	TGAAGATGAAGAATGTGTTGAATG	(AAG)12	(AAG)11	CCCTAATTTACTACCCACACCCCTCAAATGTTTTAAAAAAATTAATATTTTCAAATTACCCTCCCCTTCTTCCTCTTCATTAACCTAACTCATTTTCTTT	TACCCACACCCCTCAAATGT	59.13
Ca-II-SSR366	169.53	scaffold03665	21120	CATTGATCTGTCATGATGCCTT	(AT)7	(AT)6	GAGGAACACTAAAGCCACTATCGAGCTTGGTTACAATGAATTGGAAACTATTTTTGTTTTTGCTTCTACTAAGCAGCTTTGATATAATGATTTTCCCATA	AGCCACTATCGAGCTTGGTTA	59.96
Ca-II-SSR360	191.49	scaffold03723	15777	TTCTATTTTCACTAAGTCGCTCCA	(TTA)27	(TTA)23	TTGTATTATAGGTTATAAAAAAATTGTTGAGACTTTGGGATGACAGTGGTAGATATATTTAAGAGCGGAGAGGTCACGACAGCCCCAAAATTTTATTTTT	TTAAGAGCGGAGAGGTCACG	59.47
Ca-II-SSR368	0.94	scaffold03772	22132	CCCATCCATAAAAGGCAGAG	(AG)15	(AG)16	CATTTTAGTTAGTCTTTTGACCTTTAAAAAGTAATGACTATTTTTGTGATGAAACGAAAACATCTTAAAGTTATTTCTTACAGGTTCCTTTTCGTCGCTC	TTTCTTACAGGTTCCTTTTCGTC	59.52
Ca-II-SSR349	132.00	scaffold03877	3759	AGTCATCATCATCACGCCTC	(TC)10	(TC)11	GGGTTTTTGAAAACTAATAGAAGAGAAAAAAACGATTAAGACATGGAGAAGAGAGAGAAAGAAAAAAAATTAAAAGAGAAAGAGAAAAAAAATTAAAAGA	CGATTAAGACATGGAGAAGAGAGA	58.62
Ca-II-SSR367	0.00	scaffold03924	21916	TCTGACGGCTATCAGCTTAGATT	(AT)10	(AT)9	TAAATATTTAGTTTTTTTTATTATACAAGAGATCGCATGTTCAATTTAACTTAAATAAAATTTTATCGCAACACTAACTATTTAAAAATATTTAATAAAT	CAAGAGATCGCATGTTCAATTT	59.56

Marker IDs	Genetic positions (cM)	Chromosomes	Physical positions (bp)	Flanking sequences/Forward primers	ICC 12968	ICC4958	Flanking sequences/Reverse primers	Annealing temperature (0C)	Amplified Product size (bp)
Ca-II-SSR353	77.40	scaffold03950	7589	TCAGGCCTATCAGACCAACC	(ATT)11	(ATT)15	ATGTCAAAAATTGATGTAGATTAGATTGCTTAAATTATAGAAAATTTTAGCTAATTAATGATTAAACACAAAATGTGCAATTTAATATAATAATAATAAT	AATGATTAAACACAAAATGTGCAA	60.07
Ca-II-SSR342	118.56	scaffold03965	2194	TCACTCCTCAAGTGGAACCC	(TTA)33	(TAT)31	TTTGCATTAGGATTTCCCCCTTTCCTTCCTTTTATTTATTTATTTTCACATGGGTCAAGCCCAATAAATAACTTTTTTTTCCCTTTTTTTTCTTTCAATA	ATTTCCCCCTTTCCTTCCTT	60.09
Ca-II-SSR338	60.60	scaffold04027	1150	TCCAAGTTTTGAGGCTAGTGG	(AT)9	(AT)8	AAATTAGAGGAGCATATCCTTTATACGAGATAAAAGAGAACCACCTCAATAATATGCTCAATTCTTACCATACAAAACCTCTTTATCATTGTTTACAAAA	CGAGATAAAAGAGAACCACCTCA	59.36
Ca-II-SSR348	138.72	scaffold04158	3631	CTAGCCTGGCAAGCCTATCA	(AT)10	(AT)11	TTGAATTGTGACTGAATTGAGAATTTAACAGGGATTTTATAAATTTGGAAATGTTGAATTTATAGAGGATACTCTGCCGAAATTTCGAGTAAAAAGTATA	TGAATTTATAGAGGATACTCTGCCG	60.50
Ca-II-SSR356	132.84	scaffold04194	10213	TCTTGAATTGGTTGATCATCTTG	(CAT)5	(CAT)6	TTTAGAATTTTCAATAAGAGAACTCTATTAGTCAAAGAATCAATGCATGTATCTTTTGATGAAACTAACCCCTTGAAAGAGGACAAAAACATTTCTTATG	ACCCCTTGAAAGAGGACAAAA	59.08
Ca-II-SSR345	190.81	scaffold04214	2954	CTGCGATTGCATCCTTATCA	(ATT)13	(ATT)11	ACTTTGATTTCAAAAATTCTAAAGGTAAACAATGCAACAAATAAGAAAGAACTACATTCTATATCATTTCAATTTCCTCAATTATCAATTTGAGAAAAAT	TTCTAAAGGTAAACAATGCAACAAA	59.79
Ca-II-SSR364	111.00	scaffold04224	19662	TGGACAAAAGGGCTGTAAAA	(TA)6	(TA)7	ACTTATTTTCTGCTTATATTTAGCTTGATCTTAGTGTTTTATACTAAGTTCATGTAATTTAAGTGTACATTTAAACTGATCATGTAATTTAGCATTGATA	TTTTCTGCTTATATTTAGCTTGATCTT	58.27
Ca-II-SSR365	122.76	scaffold04292	19923	TTGCTTCTAACTCCATGAGCA	(AT)10	(AT)7	TGGGATAGCAACTTTCTAAATTTGGGGTGGTGATTCTCAGAAAAAGAAAATAAAATAATAAAAATATAAAAAGAAGAAAAAGAAAATAAAAAAATAAAAA	TTTGGGGTGGTGATTCTCAG	58.66

Marker IDs	Genetic positions (cM)	Chromosomes	Physical positions (bp)	Flanking sequences/Forward primers	ICC 12968	ICC4958	Flanking sequences/Reverse primers	Annealing temperature (0C)	Amplified Product size (bp)
Ca-II-SSR339	93.36	scaffold04309	1576	GCACACATTTATTCCAAAATCG	(AAT)9	(AAT)10	GACATCCGTTAAAATACGGTTACCTATAACTAA TTGTGCAAAATTAATATTAACTTAAGTATATTTA TTTATCCTTATAATTAAATATGAATAACAATTA	CATCCGTTAA AATACGGTTA CCT	59.37
Ca-II-SSR346	61.44	scaffold04352	3139	ACACCTATACTGCTCAAATATTCTTTT	(TA)12	(TA)15	AATATATCCCAGTGACTTTGAAAATGGCTGAGA TGAGTGATGGGTTGGGGTTTTAATTTTTTATGA GCACCAAATTTTTAAACTTAAAAAATAATATATA	GGCTGAGATG AGTGATGGGT	57.35
Ca-II-SSR362	96.72	scaffold04413	16586	TCGCTGTTAAAAATCGTAGTGC	(AT)7	(AT)8	TGGATCCCTTTTACTTACAATGTTATTTATTTAA TAGATTTAGCAGTGTTTTTTATATATAAAAAATA AAAACAAAAAATTATATATATACACCCAAATA	GGATCCCTTT TACTTACAAT GTTATTT	59.45
Ca-II-SSR363	108.48	scaffold04423	19170	TGTTGAACTTTTGAACGTGGA	(AT)10	(AT)11	TTAATTAAAGAGTTAAACAAACTATTTGACCACA TGTCTTAAGTTACTATCTAAACCGATTATTAACA ATAGTTGATAATTCATAAAAAGAAAGACTATA	AAACAAACTA TTTGACCACA TGTCTTA	59.21
Ca-II-SSR372	111.84	scaffold04453	139717	AATTGGGGACACAGGGAATA	(AT)20	(AT)12	CTTTTACATTTCAAATTAGATTTCAATATCTTCA TGAAAATTGTAACTATGCATGTTAGATTTCATTT ACAACAAATGTGAATCTATTTGAACATCTACA	TGCATGTTAG ATTTCATTTAC AACAA	59.10
Ca-II-SSR354	95.88	scaffold04477	7666	AAAGTGTTTCAGACTAAAATGCCTC	(TA)11	(TA)13	GTCGTCAATATATATCATATCAATTACATCACTT TTATTAATTTATATCTTTCTCTAAGTATATACATT TAATAGGGTGTGCATCAAACATCTAATATAT	AGGGTGTGCA TCAAACATCT	59.31
Ca-II-SSR352	12.22	scaffold04583	7538	TGCACATAGCAATTACAGATGG	(AAT)27	(AAT)29	CAAGTTATGGGATCCAGGAGGATACATTTTTTC TACGTTGTATGTCTTTCACCTTGAGGACAAGGT GATAGTTTGGGGGGATATTGTTAGACTTATTAT T	ACAAGGTGAT AGTTTGGGGG	58.73
Ca-II-SSR343	175.13	scaffold04585	2415	ATTGTTGGCAGGTTGTGGTT	(AT)16	(AT)11	CGATTGTAGTGGGGCTAATCCAAAAAAATTCAC CCACGTCTTTATATCTCGCAGAGTAAATTGTTG TAGCTTTTTACCATGAGATTAAAATATATGAAAT	CCCACGTCTT TATATCTCGC A	60.28

Marker IDs	Genetic positions (cM)	Chromosomes	Physical positions (bp)	Flanking sequences/Forward primers	ICC 12968	ICC4958	Flanking sequences/Reverse primers	Annealing temperature (0C)	Amplified Product size (bp)
Ca-II-SSR359	186.05	scaffold04590	15529	CCCCAGTTTTGGTCTCTCAA	(TAT)26	(TTA)24	TTTCCATAAAAATCTCAATCTTTTACGGACATTTTAACTTTGTATAAGTATTAAAAATTTAATACAAAAATATTAAAAGTTTAAATGAATATAGTCTAAC	AAAAATCTCAATCTTTTACGGACA	60.08
Ca-II-SSR337	34.78	scaffold04616	429	CACGTTCAAGTATATTTTGAAGCAA	(TAA)14	(TAA)12	AATACTAGCCATGCTGCTAAAACAAGGATAAGTATAATGTAAAAAATTTGAGAATATATATTTTAAATTTTACTTTTACGATTTTCTTTAAAAATTTATC	GCCATGCTGCTAAAACAAGG	59.64
Ca-II-SSR347	94.20	scaffold04869	3258	TGCGATTCAAGAAGGAGATTG	(TA)8	(TA)9	TCCTTTATTTCAGTTAGATCACTTTTTGAGTATGTTGAATTGGAGTTGTTAAAATACTATGCGATATCATATTTGAACATAAATAAGTTAGAATCAAATA	TTGGAGTTGTTAAAATACTATGCGA	60.34
Ca-II-SSR358	168.83	scaffold04875	14016	TCTGGTTGTTTATTAATTCATCTTTG	(TTA)8	(TTA)7	ACAAAATCCTCCCCCCTCGTTTAACCATTAAGTAAACAAGATTGAATTTAGTGTTAATGCTAGTTTCACACAATTTCACACCCGCATATACACAATACAC	CCCCCTCGTTTAACCATTAAG	57.89
Ca-II-SSR350	120.24	scaffold04961	5959	CAGATCAAAGGTTGGATCAAAAA	(AG)12	(AG)11	CACTTCAATGCTCCATTGTCACTTGCTCTTTTCTCGGATCTCTCTCTCACCCTTTCATCTTTTTCTTCTTCCTCTTCTCATTCTCTTCACAATTTGGTAT	TGCTCCATTGTCACTTGCTC	60.33
Ca-II-SSR340	106.80	scaffold05077	1646	ACCCACGAAATGTGTGTGAG	(CT)6	(CT)9	CCGACAAAAGAACCATGTGGGACCCAACATACTTCCAAATCTAACAGAGAATCAACCATGTGGGACTCTAACATCCTTTCCATTGTGATTGCATAGAGAG	CAAAAGAACCATGTGGGACC	59.44
Ca-II-SSR357	121.92	scaffold05186	12370	TGATTGTTGTTGTTGGTGTCTG	(AG)8	(AG)9	CATTCATTTATACCTTTTATTTTTTTCTTTTTCCAGTCCCTAATCTATGTATCTATCAATTCTTCAATATACAAATAAAAACCATTCCATTATTTATCTC	CCAGTCCCTAATCTATGTATCTATCAA	59.51
Ca-II-SSR344	185.37	scaffold05190	2785	CAAACGATTCATTCGAGGCT	(AT)7	(AT)8	AATATCAAATATCTCACATTTATTAAAAATAAATCGAACATCTCAATAATATCAAATACCACATATTTAACGAAGTTAAATCAATCAACACCTTATAAAT	CGAAGTTAAATCAATCAACACCTT	60.21

Marker IDs	Genetic positions (cM)	Chromosomes	Physical positions (bp)	Flanking sequences/Forward primers	ICC 12968	ICC4958	Flanking sequences/Reverse primers	Annealing temperature (0C)	Amplified Product size (bp)
Ca-II-SSR341	147.12	scaffold05191	2017	ACGGTAACGCTAAAAATTATCAA	(ATA)22	(ATA)29	AAGCGCAGCTGTTGCGCTTCAGGCACACAGTG TAACACCCAATTTCTTATTATATTTTAGTAATAT AATATTGATAATTTATTATTATTATTATTATTAT	GGCACACAGT GTAACACCCA	57.15
Ca-II-SSR383	73.40	scaffold05272	9743	CCTCATGTTTAGCGTTTGTTGA	(TAT)6	(TAT)7	CAACGACGAATGACGATGAC	60.17	154.00
Ca-II-SSR384	50.04	scaffold05576	4036	GGTGAGAGGGCTCATCAAGA	(AG)6	(AG)7	ACAGATGTTCCAGCGCTCTC	60.35	143.00
Ca-II-SSR385	110.12	scaffold05581	5773	TCTTTTAATTAGAGTATGCTTGAAACG	(CA)12	(CA)13	TTTTCAACTAAAGACGGACTCG	58.69	103.00
Ca-II-SSR386	91.88	scaffold05583	56098	AGAATCGCGAGAAGGATGAA	(TA)12	(TA)13	TGAATTGCGGCGAGAGAATA	59.92	184.00
Ca-II-SSR387	102.83	scaffold05624	1140	ACAGGTTGGGAATCATTTGG	(AT)14	(AT)12	CAAGGTGTGGTGGCATTGTA	59.65	168.00
Ca-II-SSR388	123.89	scaffold05741	8345	GGTTTGGAAAGTGATGGGTG	(TA)8	(AT)8	CTCTGTCCGTGTCCTTGCTT	60.21	131.00
Ca-II-SSR389	167.99	scaffold05896	797	TTGCATTTTCTCATCTTCTTGC	(TTA)25	(TTA)33	CCGCAAAGTAAACTTGGAGAA	59.48	196.00

Marker IDs	Genetic positions (cM)	Chromosomes	Physical positions (bp)	Flanking sequences/Forward primers	ICC 12968	ICC4958	Flanking sequences/Reverse primers	Annealing temperature (0C)	Amplified Product size (bp)
Ca-II-SSR390	179.69	scaffold05921	6372	ATCATGCTTTATGGCTGCTT	(AC)7	(AC)8	CCACTTGAGGGAAATTTTGG	57.48	127.00
Ca-II-SSR391	212.35	scaffold05945	121055	TTTAGTGTTTGTGTATGTGCGTG	(TG)6	(TG)7	GCATGTTATAAAGAACACACACCC	58.74	135.00
Ca-II-SSR392	175.19	scaffold05946	1181	GGCTTTTATAGAGCTTATTGACAAA	(TA)10	(TA)9	TTGAAAAAGCTTTGTTTGAGTACC	57.51	155.00
Ca-II-SSR393	131.18	scaffold05983	2502	CCTGGCACCATAGACCTCTT	(AAT)27	(AAT)22	TCAATTTGTTAATTTGACTGCATTT	59.16	204.00
Ca-II-SSR394	27.48	scaffold06002	3449	TGCACAGAAAAGAAAATAAAAAGG	(AT)8	(AT)9	TCAACATTGGAATTTGCCTG	58.94	142.00
Ca-II-SSR395	19.02	scaffold06035	8254	TCTTGCAATGACCAAAACCA	(TA)7	(TA)8	GATCCACTACATGGTCATTATAACA	60.09	146.00
Ca-II-SSR396	18.08	scaffold06182	582	CCATACATGTTTCAAGTTTCAACG	(AT)17	(AT)15	TTTGATAGAAAATTTTGAATGTCCTC	60.65	155.00
Ca-II-SSR397	4.92	scaffold06315	3095	TGTGACACCCTAAATGCCAA	(AT)10	(AT)11	CGCGTTATTATGTTTGATGGAA	59.96	191.00

Marker IDs	Genetic positions (cM)	Chromosomes	Physical positions (bp)	Flanking sequences/Forward primers	ICC 12968	ICC4958	Flanking sequences/Reverse primers	Annealing temperature (0C)	Amplified Product size (bp)
Ca-II-SSR398	1.16	scaffold06342	3787	CGATATATAATCTTAATTGGGTCTCA	(AT)10	(AT)9	CACAATTGAGTTTTATTTTGGGAA	57.04	132.00
Ca-II-SSR399	35.94	scaffold06438	4342	CCATATCACAAAAACTGCACG	(AT)11	(AT)12	TTTCCATTAAACTAGGAAAAAGTGG	59.10	148.00
Ca-II-SSR400	74.28	scaffold06441	5645	TCCGCTGCAAATTTAAAAGAA	(TA)15	(TA)13	CCAATTGGTAACTGTGACACC	59.85	100.00
Ca-II-SSR401	11.50	scaffold06839	2320	TCACCATTTAAAAATACAATACACCAA	(TA)11	(TA)12	TGGTGAAACCTCTAACGGAAA	59.85	185.00
Ca-II-SSR402	7.74	scaffold06885	4049	GGGTAAGCAAAATCAGTCACG	(AT)12	(TA)11	TGACTTTTCAAATACGTTGTTTGTT	59.62	125.00
Ca-II-SSR403	67.24	scaffold07041	5574	TTATGTCGCTATCATTCAATGTG	(AT)13	(AT)15	CGTGCAAGAACAAGGTTCAA	57.76	171.00
Ca-II-SSR404	87.48	scaffold07062	1520	GCGACCATTACAACAATCCC	(AT)7	(AT)12	CGACACTGTAGCACCCCTTT	60.20	191.00
Ca-II-SSR405	90.12	scaffold07242	5503	CGCGGGATGTATTTTACCAA	(TA)12	(TA)10	TTGAAGTCAATGCTCAAGTGC	60.69	143.00

Marker IDs	Genetic positions (cM)	Chromosomes	Physical positions (bp)	Flanking sequences/Forward primers	ICC 12968	ICC4958	Flanking sequences/Reverse primers	Annealing temperature (0C)	Amplified Product size (bp)
Ca-II-SSR406	98.78	scaffold07311	1902	GCACATAGTTTCGAACTTGCTCT	(TA)11	(TA)9	AATGCCAGTGGAGTCACACA	59.97	129.00
Ca-II-SSR407	123.08	scaffold07533	7350	GTGAAATAGCAGCACCGACA	(AG)10	(AG)11	TCACCTCCTCCAAAATCACC	59.87	138.00
Ca-II-SSR408	168.89	scaffold07615	6328	GGCTTTTCTCACTTCTCCCC	(CTT)9	(TCT)8	TCCTCTGTATGGCAGTCGAA	60.19	108.00
Ca-II-SSR409	180.59	scaffold07735	4651	TGTGTTTCCGAAGCATACCA	(TA)6	(TA)7	AGAAATGGGCGCATAACTGT	60.11	139.00
Ca-II-SSR410	210.71	scaffold07987	3854	TTTGCCTTGTGGATTCAATTT	(AAT)18	(ATA)15	CAATATCGATTGCACTCATTTCA	59.44	158.00
Ca-II-SSR411	173.39	scaffold07993	3308	CGGTTGGTCTCACCAAATCT	(AT)12	(AT)14	CATCATGAAACAATTTCAAAAGGTA	59.97	141.00
Ca-II-SSR412	97.97	scaffold08095	3404	TCCTAGAGTCAACGAATCTCCA	(TA)12	(TA)11	TGTTGGAACACGTCACATCAT	58.93	131.00
Ca-II-SSR413	124.70	scaffold08140	6452	TGCGGTGAATCTTTTACGAA	(AT)15	(AT)10	TTCTATTTCCTTTTCTTTTCCCC	59.30	144.00

Marker IDs	Genetic positions (cM)	Chromosomes	Physical positions (bp)	Flanking sequences/Forward primers	ICC 12968	ICC4958	Flanking sequences/Reverse primers	Annealing temperature (0C)	Amplified Product size (bp)
Ca-II-SSR414	146.39	scaffold08236	758	AGATTCGCAGGCAGTTGAGT	(AT)8	(AT)7	GCGCGCAAGCTATACTCCTA	60.02	156.00
Ca-II-SSR415	198.59	scaffold08475	299	TTCAACAATATTCACAAATCCTCAA	(TA)8	(TA)7	ATTTATCCGCTGCAAATTTTAT	59.78	139.00
Ca-II-SSR416	209.89	scaffold08482	3815	CTTTCCTTCCCTTCCATTCC	(CT)9	(CT)10	TCATAGCAAAGTTTAGCAAACAAAA	59.88	130.00
Ca-II-SSR417	174.29	scaffold08490	4849	CCTTATAGGGGATGGAAAATGA	(AT)9	(AT)8	TTTTGGCGATTGTACCAACTT	59.19	136.00
Ca-II-SSR418	131.99	scaffold08492	3756	TCCACTTTATCCATCATTCGG	(CTT)14	(CTT)15	CCAAACACGAGAAGAGGTTGA	59.77	170.00
Ca-II-SSR419	36.88	scaffold08497	3479	TGAATTTAAAGATTTGTTGGAAACTT	(TTA)17	(TTA)15	CATGAAACATACTCCCTTCAATC	58.70	172.00
Ca-II-SSR420	50.98	scaffold08547	414	GCATTCTCGCTGTTCCTTCT	(AT)9	(AT)7	CACTCATTTGAGCTCGGAAA	59.58	166.00
Ca-II-SSR421	110.93	scaffold08648	6145	TCGTTAAATGTGTGGCATTTG	(TGA)5	(TGA)6	CCATTAATACCGTCATCTCAGACA	59.48	176.00

Marker IDs	Genetic positions (cM)	Chromosomes	Physical positions (bp)	Flanking sequences/Forward primers	ICC 12968	ICC4958	Flanking sequences/Reverse primers	Annealing temperature (0C)	Amplified Product size (bp)
Ca-II-SSR422	92.76	scaffold08666	3070	CGGATACCAAAAAGTATCACCAA	(AAT)23	(AAT)20	TTCTGTGTGATTGGGGTGAA	60.12	180.00
Ca-II-SSR423	103.64	scaffold08707	2537	GCAACTGTTTCTTGGTCTTCG	(AT)7	(AT)8	CAATTTCCATTTCCACAAATCA	59.91	140.00
Ca-II-SSR464	144.39	scaffold08854	4837	TTGATACTTGATTGATTCTTGATCTT	(CA)9	(CA)8	TGTTGCTCGTGTACGTCAGAT	57.55	144.00
Ca-II-SSR443	169.89	scaffold08907	692	TTTGAACGGTTGACGAATGA	(TTA)32	(TTA)31	CGTGGAATGTAACACCTAGTCG	60.09	189.00
Ca-II-SSR460	10.64	scaffold09135	3338	TTGTAGAGTCCAAAACTTGGCA	(TA)9	(AT)11	TGTTGGAGTTGTGTTGATTCTT	59.79	133.00
Ca-II-SSR453	143.54	scaffold09202	2058	TCGGGATATTAGTAATGTTCTGTTTG	(AT)9	(TA)9	CATCATTAGAGGATGGTCGAAA	59.73	148.00
Ca-II-SSR440	116.55	scaffold09234	574	CTGTTTTCAATGCCTTGCCT	(CA)14	(CA)13	GAGATGTGAGTGAAGTGATGTGTG	60.25	152.00
Ca-II-SSR445	9.75	scaffold09449	813	CCTTTGCTTTTCCTTCCTTC	(AT)11	(AT)9	GGAATGAAATGATGGGGGTA	58.05	178.00

Marker IDs	Genetic positions (cM)	Chromosomes	Physical positions (bp)	Flanking sequences/Forward primers	ICC 12968	ICC4958	Flanking sequences/Reverse primers	Annealing temperature (0C)	Amplified Product size (bp)
Ca-II-SSR459	2.63	scaffold09742	3299	TTGGGGGAGAATGTATAAAATGA	(TA)10	(TA)9	TTTTCCCTTCAAGCTGCAAT	59.58	148.00
Ca-II-SSR439	112.10	scaffold09754	527	GGTTTGGAAAGTGATGGATGA	(TA)7	(TA)6	TCCAGTTTTCCTCCGTTCTC	59.78	171.00
Ca-II-SSR441	123.67	scaffold10234	718	CCGCCAAAATCCAAAGTTTA	(TA)12	(TA)13	ATCGGGTGTTTTCACGAGAC	59.94	163.00
Ca-II-SSR458	198.27	scaffold10438	2543	TCCTTTTAGAGAATTAAATTGTGTTGG	(AT)13	(TA)12	CAACACATAGTAAAACCAATGTGTAAA	60.16	144.00
Ca-II-SSR463	51.58	scaffold10813	4131	CAAATTCTTTTCCCATTTGTTCA	(CTT)5	(TCT)5	CAAAGCAGAATGTCCCTCAA	60.20	128.00
Ca-II-SSR465	118.33	scaffold10990	112130	AATGGGGAAAGTAGGGAACG	(TTA)27	(TTA)26	TTTAGGTGTAGAAATAATTGTTGATGA	60.18	203.00
Ca-II-SSR442	177.52	scaffold11736	734	AAAGGATCAAATCGGGAGAT	(AT)7	(TA)10	ACCAGGTGTTGCATTTGACA	57.53	157.00
Ca-II-SSR462	24.88	scaffold11762	3896	CGCTGTAGAGAACCTCCCAC	(TTC)17	(TTC)19	CCGAGCCAAAATCCAAAATA	59.87	179.00

Marker IDs	Genetic positions (cM)	Chromosomes	Physical positions (bp)	Flanking sequences/Forward primers	ICC 12968	ICC4958	Flanking sequences/Reverse primers	Annealing temperature (0C)	Amplified Product size (bp)
Ca-II-SSR461	93.41	scaffold11876	3439	GCCAAAGTTAATAATATTTTGGAGA	(AT)6	(AT)8	TTTTCCTTTCTTTTTCCCCC	57.18	110.00
Ca-II-SSR456	178.35	scaffold12317	2469	CATCGAGCTGCAAAGAAAGA	(AGA)11	(AGA)15	GCTACGGCACTATATGAGGCT	59.30	144.00
Ca-II-SSR447	23.99	scaffold12437	992	CATCACAACCATAAACAACAACA	(AAC)12	(AAC)8	AAAAGGGGAGTTCAGCGACT	58.38	169.00
Ca-II-SSR436	80.95	scaffold12901	236	TGTATGTGAATCTTGCCTCGTC	(TA)16	(TA)14	TGGATTTGCACACAACAAGAA	60.14	222.00
Ca-II-SSR435	73.83	scaffold13062	139	TATGATCCGTTGTCGCACAC	(ATA)18	(ATA)17	CGTTTTGCTCATCTGTTAAATCA	60.55	202.00
Ca-II-SSR437	23.10	scaffold13160	285	AAACTCTGAGGTTTAATTGACTTGTAA	(TAA)13	(TAA)14	CTGATTTATGTGGAGTGCGG	58.14	204.00
Ca-II-SSR450	160.54	scaffold13471	1289	ACTCCCTCTTGTTGCTAGAAAAT	(TA)8	(TA)9	ATTGGATTAGTGAAATTCATTGTTT	57.70	198.00
Ca-II-SSR434	1.74	scaffold13784	101	GAAGCAAAAGAGGAGGAAAGG	(TTA)27	(TTA)29	ACACGTGTCGGACATCAAAT	59.47	198.00

Marker IDs	Genetic positions (cM)	Chromosomes	Physical positions (bp)	Flanking sequences/Forward primers	ICC 12968	ICC4958	Flanking sequences/Reverse primers	Annealing temperature (0C)	Amplified Product size (bp)
Ca-II-SSR449	142.69	scaffold14000	1152	AAAAATTTACGATGCAATGAACA	(TA)7	(TA)8	TGAGCTCTTGTTGAATACAATTATCA	58.60	199.00
Ca-II-SSR444	197.44	scaffold14143	773	TTTAGGATTTTATTTGTGGGGG	(AAAATA)5	(AAAAT)5	TATCCAATCATGCCAGTCCA	59.13	151.00
Ca-II-SSR455	131.64	scaffold14352	2439	GGGATCTTTTTGGCTGAAGA	(TTG)9	(TTG)5	GGTCGAAGTTGAACATACCGA	59.24	100.00
Ca-II-SSR446	92.52	scaffold14449	909	CGAACATCAAGCAATTTTACGA	(TA)7	(AT)6	TCAAATCACACTAAAGAGATACGAAAA	60.13	140.00
Ca-II-SSR457	170.74	scaffold14543	2519	AAGGGGAATGAGTTTTTGCC	(TA)8	(TA)9	GATTGAGAAGGGAGTGATGTGA	60.30	141.00
Ca-II-SSR451	130.79	scaffold15923	1495	TGAAACCAAGAATTATGTTTTAGGG	(AT)8	(AT)9	TCATACCAGATGCTCTCCACA	59.70	146.00
Ca-II-SSR454	161.39	scaffold16191	2082	GCAAATTCTTAAAAAGCGGTG	(TA)18	(TA)17	CGGATATCCTAAAGCCTTGTCA	58.93	115.00
Ca-II-SSR448	40.01	scaffold16819	1116	TGCTGACTGTTTGGTTCGAG	(GA)12	(GA)10	CTTTTCCCCAAAAGCTGGAC	60.03	118.00

Marker IDs	Genetic positions (cM)	Chromosomes	Physical positions (bp)	Flanking sequences/Forward primers	ICC 12968	ICC4958	Flanking sequences/Reverse primers	Annealing temperature (0C)	Amplified Product size (bp)
Ca-II-SSR438	106.76	scaffold16843	435	TACATCTCCAAATGCGACCA	(AAT)15	(AAT)13	CACTACGTGTAAAAATGGGCAA	60.07	134.00
Ca-II-SSR452	50.69	scaffold16975	1903	CCTTGCACGACGAGTACAAC	(AT)14	(AT)11	CACTGTAGCACCCCTTTCGT	59.37	181.00

*Ca-IISSR (***C****icer*** a****rietinum* **I**CC 4958 and **I**CC 12968 **S**imple **S**equence **R**epeat) and Ca-IISNP (***C****icer*** a****rietinum* **I**CC 4958 and **I**CC 12968 **S**ingle **N**ucleotide **P**olymorphism)

Supplementary Table S2: Phenotypic variations of three quantitative agronomic traits determined in an intra-specific chickpea mapping population (ICC 4958 x ICC 12968) using diverse statistical measures

**Traits**	**Years**	**Parental genotypes**		**F_4 mapping individuals**		**Heritability (H^2%)**	**Coefficient of variation (CV)**	**Least square difference (LSD)**	**ANOVA significance (P)**
		**ICC 4958 (Mean ± SD)**	**ICC 12968 (Mean ± SD)**	**(Mean ± SD)**	**Range**				
Pod number per plant (PN)	2012	101.6 ± 2.2	46.7 ± 2.3	59.8 ± 12.6	37.1-109.4	75	21.1	24.8	< 0.0001
	2013	110.5 ± 2.5	50.8 ± 2.4	65.0 ± 13.8	40.4-119.0	76	21.2	27.2	< 0.001
Seed number per plant (SN)	2012	137.2 ± 2.1	54.2 ± 1.8	71.8 ± 15.5	43.9-137.2	72	21.6	30.6	< 0.001
	2013	146.4 ± 2.0	57.8 ± 1.9	76.6 ± 16.6	46.8-146.4	70	21.7	32.7	< 0.0001
100-seed weight (g) (SW)	2012	35.4 ± 2.2	20.8 ± 2.1	28.1 ± 4.2	20.4-43.0	89	14.9	8.0	< 0.0001
	2013	33.8 ± 1.6	22.6 ± 1.5	29.0 ± 4.3	21.1-44.5	87	14.8	8.2	< 0.0001

Supplementary Table S3: ANOVA-based summary effects of three quantitative agronomic traits measured in an intra-specific chickpea mapping population (ICC 4958 x ICC 12968) across two experimental years

**Traits**	**Variance**	**Degree of freedom (*df*)**	**Mean square (MS)**	**Significance (P)**
Pod number per plant (PN)	Genotype (G)	189	348.3	P < 0.0001
	Environment (E)	1	3967.6	P < 0.0001
	G x E interaction	189	0.62	P < 0.001
	Error (e)	380	174.5	P < 0.001
Seed number per plant (SN)	Genotype (G)	189	515.4	P < 0.0001
	Environment (E)	1	3316.8	P < 0.001
	G x E interaction	189	0.54	P < 0.001
	Error (e)	380	257.9	P < 0.001
100-seed weight (g) (SW)	Genotype (G)	189	36.2	P < 0.0001
	Environment (E)	1	138.4	P < 0.0001
	G x E interaction	189	0.01	P < 0.0001
	Error (e)	380	18.1	P < 0.0001

Supplementary Table S4: Genomic distribution of SNP and SSR markers physically mapped on eight *desi* chickpea chromosomes

**Chromosomes**	**Size (Mb) of chromosomes (pseudomolecules)**	**Number (%) of SNP and SSR markers mapped**	**Average map density (kb)**
Ca-desi-Chr01	14.79	205 (12.4)	72.1
Ca-desi-Chr02	17.30	207 (12.5)	83.6
Ca-desi-Chr03	23.38	219 (13.2)	106.8
Ca-desi-Chr04	22.09	211 (12.7)	104.7
Ca-desi-Chr05	16.30	210 (12.7)	77.6
Ca-desi-Chr06	11.48	201 (12.1)	57.1
Ca-desi-Chr07	8.46	202 (12.2)	41.9
Ca-desi-Chr08	10.57	202 (12.2)	52.3
**Total**	**124.37**	**1657**	**75.0**

Ca-desi-Chr: *Cicer arietinum desi* chromosome

Supplementary Table S5: SNP and SSR marker-based comparative mapping between the chromosomes of *desi* and *kabuli* chickpea genomes

**Chickpea *desi* linkage groups**	**Number of chickpea unique loci**	**Chickpea *Kabuli* chromosomes**								**Total**
		***Cak*Chr1**	***Cak*Chr2**	***Cak*Chr3**	***Cak*Chr4**	***Cak*Chr5**	***Cak*Chr6**	***Cak*Chr7**	***Cak*Chr8**	
*Cad*LG01	197	**156 (64.19%)**	8	15	4	16	24	13	7	243
*Cad*LG02	183	19	**98 (38.74%)**	6	25	17	68	17	3	253
*Cad*LG03	179	29	13	**101 (39.29%)**	14	17	21	22	40	257
*Cad*LG04	192	17	26	12	**137 (53.31%)**	18	20	18	9	257
*Cad*LG05	169	13	10	5	8	**153 (71.16%)**	13	9	4	215
*Cad*LG06	176	14	47	3	13	8	**132 (54.32%)**	20	6	243
*Cad*LG07	157	18	15	13	27	17	40	**103 (42.56%)**	9	242
*Cad*LG08	197	28	3	6	20	25	9	**173 (64.31%)**	5	269
**Total**	**1450**	**138**	**122**	**60**	**111**	**118**	**195**	**99**	**83**	**1979**

Supplementary Table S6: SNP and SSR marker-based comparative mapping between the chromosomes of *desi* chickpea and *Medicago* genomes

**Chickpea linkage groups**	**Number of chickpea unique loci**	***Medicago truncatula* chromosomes**								**Total**
		***Mt*Chr1**	***Mt*Chr2**	***Mt*Chr3**	***Mt*Chr4**	***Mt*Chr5**	***Mt*Chr6**	***Mt*Chr7**	***Mt*Chr8**	
*Cad*LG01	28	10	**18 (22.22%)**	16	6	11	6	7	7	81
*Cad*LG02	35	10	12	20	**36 (25.35%)**	31	6	19	8	142
*Cad*LG03	34	9	8	16	13	14	4	**31 (29.80%)**	9	104
*Cad*LG04	28	**19 (27.53%)**	7	10	6	7	7	9	4	69
*Cad*LG05	29	17	22	**49 (24.87%)**	21	33	15	20	20	197
*Cad*LG06	25	8	5	10	14	**18 (21.43%)**	2	9	18	84
*Cad*LG07	21	10	16	21	**28 (18.92%)**	28	4	25	16	148
*Cad*LG08	31	4	8	6	**21 (30.00%)**	5	5	6	15	70
**Total**	**231**	**68**	**78**	**99**	**60**	**129**	**49**	**95**	**97**	**895**

Supplementary Table S7: SNP and SSR marker-based comparative mapping between the chromosomes of *desi* chickpea and *Glycine* genomes

**Chickpea linkage groups**	**Number of chickpea unique loci**	***Glycine max* chromosomes**																				**Total**
		***Gm*Chr1**	***Gm*Chr2**	***Gm*Chr3**	***Gm*Chr4**	***Gm*Chr5**	***Gm*Chr6**	***Gm*Chr7**	***Gm*Chr8**	***Gm*Chr9**	***Gm*Chr10**	***Gm*Chr11**	***Gm*Chr12**	***Gm*Chr13**	***Gm*Chr14**	***Gm*Chr15**	***Gm*Chr16**	***Gm*Chr17**	***Gm*Chr18**	***Gm*Chr19**	***Gm*Chr20**	
*Cad*LG01	29	8	2	0	6	3	8	4	5	15	7	2	6	**16 (12.16%)**	3	15	7	7	1	6	2	123
*Cad*LG02	25	3	13	1	**20 (11.56%)**	11	12	6	5	6	0	12	7	3	20	6	11	13	7	6	11	173
*Cad*LG03	27	10	**12 (9.98%)**	9	3	7	0	2	8	9	8	5	9	5	6	8	2	2	4	12	2	124
*Cad*LG04	29	0	**10 (12.99%)**	3	4	1	5	3	5	2	6	2	7	6	4	1	1	2	4	9	2	77
*Cad*LG05	27	15	14	11	**17 (10.37%)**	10	13	2	7	2	7	2	1	4	7	9	8	12	5	3	15	164
*Cad*LG06	17	2	**7 (13.21%)**	0	1	9	3	2	**7**	2	0	4	0	4	2	1	2	0	4	3	0	53
*Cad*LG07	18	3	1	0	2	1	1	3	4	4	4	3	1	2	1	0	2	**4 (10.00%)**	2	0	1	40
*Cad*LG08	21	2	6	5	9	4	**15 (14.15%)**	12	5	3	1	1	2	3	1	12	2	7	6	8	2	106
**Total**	**193**	**43**	**36**	**29**	**25**	**46**	**42**	**34**	**46**	**43**	**33**	31	33	27	44	52	35	43	33	47	35	860

Supplementary Table S8: SNP and SSR marker-based comparative mapping between the chromosomes of *desi* chickpea and *Lotus* genomes

**Chickpea linkage groups**	**Number of chickpea unique loci**	***Lotus japonicus* chromosomes**							**Total**
		***Lj*Chr1**	***Lj*Chr2**	***Lj*Chr3**	***Lj*Chr4**	***Lj*Chr5**	***Lj*Chr6**	***Lj*Chr7**	
*Cad*LG01	8	2	0	4	1	1	2	**5 (33.33%)**	15
*Cad*LG02	13	11	3	6	5	3	6	**14 (29.16%)**	48
*Cad*LG03	17	**13 (39.39%)**	3	5	3	5	0	4	33
*Cad*LG04	10	**7 (41.18%)**	6	2	0	1	0	1	17
*Cad*LG05	13	5	**6 (21.43%)**	3	5	1	2	6	28
*Cad*LG06	9	3	2	4	**10 (37.04%)**	3	2	3	27
*Cad*LG07	10	2	**5 (27.77%)**	2	2	0	2	5	18
*Cad*LG08	19	2	1	0	**10 (47.62%)**	2	3	2	21
**Total**	**99**	**25**	**15**	**26**	**16**	**16**	**17**	**21**	**207**

Supplementary Table S9: SNP and SSR marker-based comparative mapping between the chromosomes of *desi* chickpea and *Cajanus* genomes

**Chickpea linkage groups**	**Number of chickpea unique loci**	***Cajanus cajan* chromosomes**											**Total**
		***Cc*Chr1**	***Cc*Chr2**	***Cc*Chr3**	***Cc*Chr4**	***Cc*Chr5**	***Cc*Chr6**	***Cc*Chr7**	***Cc*Chr8**	***Cc*Chr9**	***Cc*Chr10**	***Cc*Chr11**	
*Cad*LG01	11	0	3	2	1	0	0	1	**5 (27.77%)**	3	1	2	18
*Cad*LG02	17	2	5	2	2	0	3	0	0	3	2	**11 (36.66%)**	30
*Cad*LG03	17	4	4	7	0	1	1	**8 (21.05%)**	3	1	2	7	38
*Cad*LG04	18	4	**5 (16.66%)**	4	2	0	4	1	3	1	2	4	30
*Cad*LG05	17	3	3	3	0	2	1	4	2	1	1	**10 (33.33%)**	30
*Cad*LG06	14	0	**6 (30.00%)**	3	1	2	1	0	3	1	3	0	20
*Cad*LG07	6	1	1	0	1	0	0	0	**2 (25.00%)**	0	1	2	8
*Cad*LG08	13	0	1	3	0	0	0	1	3	1	1	**8 (44.44%)**	18
**Total**	**113**	**14**	**17**	**24**	**7**	**5**	**10**	**7**	**14**	**11**	**13**	**15**	**192**

Supplementary Table S10: Chickpea genotypes utilized for evaluating polymorphic potential of SNP and SSR markers

Sl. No.	Genotypes with Accession No.	Cultivar types	Geographical origin
1	ICC7571	*Cicer arietinum Kabuli* landrace	Israel
2	ICC16796	*Cicer arietinum Kabuli* landrace	Portugal
3	ICCV1	*Cicer arietinum Desi* variety	Western India
4	ICCC37	*Cicer arietinum Desi* variety	Southern India
5	ICCV88202	*Cicer arietinum Desi* variety	Northern India
6	ICCV93952	*Cicer arietinum Desi* variety	Central India
7	ICCV93954	*Cicer arietinum Desi* variety	Southern India
8	ICCV91106	*Cicer arietinum Desi* variety	Central India
9	ICCV92944	*Cicer arietinum Desi* variety	Central India
10	ICCL87207	*Cicer arietinum Desi* variety	Central India
11	ICC4951	*Cicer arietinum Desi* landrace	Central India
12	ICC8933	*Cicer arietinum Desi* genetic stock	Northern India
13	IC296131	*Cicer arietinum Desi* variety	Northern India
14	IC296132	*Cicer arietinum Desi* variety	Northern India
15	IC296133	*Cicer arietinum Desi* variety	Northern India
16	BGD112	*Cicer arietinum Desi* genetic stock	Northern India
17	BGD1103	*Cicer arietinum Desi* genetic stock	Northern India
18	Pusa256	*Cicer arietinum Desi* variety	Northern India
19	Pusa547	*Cicer arietinum Desi* variety	Northern India
20	BGD72	*Cicer arietinum Desi* genetic stock	Northern India
21	IC244160	*Cicer arietinum Desi* variety	Northern India
22	IC244217	*Cicer arietinum Desi* variety	Northern India
23	SBD377	*Cicer arietinum Desi* variety	Northern India
24	Pusa2024	*Cicer arietinum Desi* variety	Northern India
25	Pusa1103	*Cicer arietinum Desi* variety	Northern India
26	ICC14402	*Cicer arietinum Desi* landrace	ICRISAT
27	ICC5135	*Cicer arietinum Desi* landrace	India
28	ICC13524	*Cicer arietinum Desi* landrace	Iran
29	ICC13219	*Cicer arietinum Desi* landrace	Iran
30	ICC13187	*Cicer arietinum Desi* landrace	Iran
31	ICCV89314	*Cicer arietinum Desi* variety	Eastern India
32	ICCV96970	*Cicer arietinum Desi* variety	Central India
33	ICCV94954	*Cicer arietinum Desi* variety	Central India
34	ICCV10	*Cicer arietinum Desi* variety	Southern/Central India
35	ICCV93958	*Cicer arietinum Desi* variety	Southern India
36	ICC4958	*Cicer arietinum Desi* landrace	Central India
37	ICC3512	*Cicer arietinum Desi* landrace	Iran
38	ICC8950	*Cicer arietinum Desi* landrace	India
39	ICC6263	*Cicer arietinum Desi* landrace	Russia & CISs
40	ICC283	*Cicer arietinum Desi* landrace	India
41	ICC1431	*Cicer arietinum Desi* landrace	India

Sl. No.	Genotypes with Accession No.	Cultivar types	Geographical origin
42	ICC4918	*Cicer arietinum Desi* breeding line/cultivar	India
43	ICC1205	*Cicer arietinum Desi* landrace	India
44	ICC1882	*Cicer arietinum Desi* landrace	India
45	ICC1356	*Cicer arietinum Desi* landrace	India
46	ICCX-810800	*Cicer arietinum Desi* variety	Northern India
47	ICCX-820065	*Cicer arietinum Desi* variety	Western India
48	ICCV90201	*Cicer arietinum Desi* variety	Northern India
49	ICC1392	*Cicer arietinum Desi* landrace	India
50	ICC3362	*Cicer arietinum Desi* landrace	Iran
51	ICC2969	*Cicer arietinum Desi* landrace	Iran
52	ICC12037	*Cicer arietinum Desi* landrace	Mexico
53	ICC1052	*Cicer arietinum Desi* landrace	Pakistan
54	ICC11284	*Cicer arietinum Desi* landrace	Russia & CISs
55	ICCV2/ICC12968	*Cicer arietinum Kabuli* variety	Southern India
56	ICCV92311	*Cicer arietinum Kabuli* variety	Central India
57	ICCV95311	*Cicer arietinum Kabuli* variety	Southern India
58	ICCV96329	*Cicer arietinum Kabuli* variety	Southern India
59	ICCV95334	*Cicer arietinum Kabuli* variety	Central India
60	IC411514	*Cicer arietinum Kabuli* variety	Northern India
61	Pusa5028	*Cicer arietinum Kabuli* variety	Northern India
62	IC449069	*Cicer arietinum Kabuli* variety	Northern India
63	Pusa5023	*Cicer arietinum Kabuli* variety	Northern India
64	Pusa2024	*Cicer arietinum Kabuli* variety	Northern India
65	IC 296376	*Cicer arietinum Kabuli* variety	Northern India
66	IC 244243	*Cicer arietinum Kabuli* variety	Northern India
67	ICC5003	*Cicer arietinum Kabuli* variety	Northern India
68	PhuleG0515	*Cicer arietinum Kabuli* variety	Central India
69	BGD1105	*Cicer arietinum Kabuli* genetic stock	Northern India
70	ICC8740	*Cicer arietinum Kabuli* landrace	Afghanistan
71	ICC16524	*Cicer arietinum Kabuli* landrace	Pakistan
72	ICC13523	*Cicer arietinum Kabuli* landrace	Iran
73	ICC12328	*Cicer arietinum Kabuli* landrace	Cyprus
74	ICC7272	*Cicer arietinum Kabuli* landrace	Algeria
75	ICC11764	*Cicer arietinum Kabuli* landrace	Chile
76	ICC15697	*Cicer arietinum Kabuli* landrace	Syrian Arab Republic
77	ICC8261	*Cicer arietinum Kabuli* landrace	Turkey
78	ICC15333	*Cicer arietinum Kabuli* landrace	Iran
79	ICC10755	*Cicer arietinum Kabuli* landrace	Turkey
80	ICC10885	*Cicer arietinum Kabuli* landrace	Ethiopia
81	ICC9137	*Cicer arietinum Kabuli* landrace	Iran
82	ICC15406	*Cicer arietinum Kabuli* landrace	Morocco
83	ICC8151	*Cicer arietinum Kabuli* landrace	United States of America
84	ICC14199	*Cicer arietinum Kabuli* landrace	Mexico
85	ICC12824	*Cicer arietinum Desi* landrace	Ethiopia

Sl. No.	Genotypes with Accession No.	Cultivar types	Geographical origin
86	ICC12537	*Cicer arietinum Desi* landrace	Ethiopia
87	ICC9643	*Cicer arietinum Desi* landrace	Afghanistan
88	ICC8621	*Cicer arietinum Desi* landrace	Ethiopia
89	ICC9895	*Cicer arietinum Desi* landrace	Afghanistan
90	ICCV95332	*Cicer arietinum Kabuli* variety	Central India
91	ICCV95333	*Cicer arietinum Kabuli* variety	Central India
92	ICCV92337	*Cicer arietinum Kabuli* variety	Central India
